# Effect of Mass Azithromycin Distributions on Childhood Growth in Niger: A Cluster-Randomized Trial

**DOI:** 10.1001/jamanetworkopen.2021.39351

**Published:** 2021-12-30

**Authors:** Ahmed M. Arzika, Ramatou Maliki, Maria M. Ali, Mankara K. Alio, Amza Abdou, Sun Y. Cotter, Nicole E. Varnado, Elodie Lebas, Catherine Cook, Catherine E. Oldenburg, Kieran S. O’Brien, E. Kelly Callahan, Robin L. Bailey, Sheila K. West, Travis C. Porco, Thomas M. Lietman, Jeremy D. Keenan

**Affiliations:** 1 Carter Center, Niamey, Niger; 2 Centre de Recherche et Interventions en Santé Publique, Birni N’Gaoure, Niger; 3 Programme National de Santé Oculaire, Niamey, Niger; 4 Francis I. Proctor Foundation, University of California, San Francisco; 5 Department of Ophthalmology, University of California, San Francisco; 6 Department of Epidemiology & Biostatistics, University of California, San Francisco, California; 7 Carter Center, Atlanta, Georgia; 8 London School of Hygiene and Tropical Medicine, London, England; 9 Dana Center for Preventive Ophthalmology, Johns Hopkins University, Baltimore, Maryland; 10 Institute for Global Health Sciences, University of California, San Francisco

## Abstract

**Importance:**

Mass azithromycin distributions may decrease childhood mortality, although the causal pathway is unclear. The potential for antibiotics to function as growth promoters may explain some of the mortality benefit.

**Objective:**

To investigate whether biannual mass azithromycin distributions are associated with increased childhood growth.

**Design, Setting, and Participants:**

This cluster-randomized trial was performed from December 2014 until March 2020 among 30 rural communities in Boboye and Loga departments in Niger, Africa, with populations from 200 to 2000 individuals. Communities were randomized in a 1:1 ratio to biannual mass distributions of azithromycin or placebo for children ages 1 to 59 months. Participants, field-workers, and study personnel were masked to treatment allocation. Height and weight changes from baseline to follow-up at 4 years were compared between groups. Data were analyzed from June through November 2021.

**Interventions:**

Participants received azithromycin at 20 mg/kg using height-based approximation or by weight for children unable to stand every 6 months at the participants’ households. Placebo contained the vehicle of the azithromycin suspension.

**Main Outcomes and Measures:**

Longitudinal anthropometric assessments were performed on a random sample of children before the first treatment and then annually for 5 years. Height and weight were the prespecified primary outcomes.

**Results:**

Among 3936 children enrolled from 30 communities, baseline characteristics were similar between 1299 children in the azithromycin group and 2637 children in the placebo group (mean 48.2% [95% CI, 45.5% to 50.8%] girls vs 48.0% [95% CI, 45.7% to 50.3%] girls; mean age, 30.8 months [95% CI, 29.5 to 32.0 months] vs 30.6 months [95% CI, 29.2 to 31.6 months]). Baseline anthropometric assessments were performed among 2230 children, including 985 children in the azithromycin group and 1245 children in the placebo group, of whom follow-up measurements were available for 789 children (80.1%) and 1063 children (85.4%), respectively. At the prespecified 4-year follow-up visit, children in the azithromycin group gained a mean 6.7 cm (95% CI, 6.5 to 6.8 cm) in height and 1.7 kg (95% CI, 1.7 to 1.8 kg) in weight per year and children in the placebo group gained a mean 6.6 cm (95% CI, 6.4 to 6.7 cm) in height and 1.7 kg (95% CI, 1.7 to 1.8 kg) in weight per year. Height at 4 years was not statistically significantly different between groups when adjusted for baseline height (0.08 cm [95% CI, −0.12 to 0.28 cm] greater in the azithromycin group; *P* = .45), and neither was weight when adjusted for height and baseline weight (0.02 kg [95% CI, −0.10 to 0.06 kg] less in the azithromycin group; *P* = .64). However, among children in the shortest quartile of baseline height, azithromycin was associated with a 0.4 cm (95% CI, 0.1 to 0.7 cm) increase in height compared with placebo.

**Conclusions and Relevance:**

This study did not find evidence of an association between mass azithromycin distributions and childhood growth, although subgroup analysis suggested some benefit for the shortest children. These findings suggest that the mortality benefit of mass azithromycin distributions is unlikely to be due solely to growth promotion.

**Trial Registration:**

ClinicalTrials.gov Identifier: NCT02048007

## Key Points

**Question:**

Do biannual mass azithromycin distributions, which have been shown to decrease child mortality in sub-Saharan Africa, promote child growth?

**Findings:**

In this placebo-controlled cluster-randomized trial with longitudinal anthropometric monitoring among 2230 children from 30 communities in Niger, mass azithromycin distributions did not have a statistically significant effect on height or weight. However, among the shortest quartile of children, administration of mass azithromycin resulted in statistically significantly taller children.

**Meaning:**

This study found that biannual mass azithromycin distributions did not impact growth, suggesting that the mortality benefit associated with them cannot be attributed to growth promotion.

## Teaser

This parallel-design double-blind trial investigates the effect of mass azithromycin distributions on child growth in Africa.

## Introduction

Mass azithromycin distributions have been shown to decrease childhood mortality in sub-Saharan Africa, even when not given for a specific indication.^1,2^ Several theories exist to explain this observed mortality benefit. One hypothesis posits growth promotion as a mechanism, given the estimation that approximately half of childhood mortality in resource-limited settings is related to malnutrition.^3,4^

The Macrolides Oraux pour Réduire les Décès avec un Oeil sur la Résistance (MORDOR) trial^1^ was a cluster-randomized, placebo-controlled trial undertaken in Malawi, Niger, and Tanzania that demonstrated decreased mortality among preschool children treated with mass azithromycin distributions. Concurrent with the MORDOR trial, smaller trials were conducted at each site to assess for potential causes of decreased mortality, including childhood growth. Just as in the parent trial, cluster randomization was used to account for the possibility that antibiotic intervention could have spillover effects on members of the community who were untreated.^5^ Unlike previous trials assessing the effect of mass azithromycin on childhood growth, MORDOR was placebo controlled and followed a longitudinal sample of children. We hypothesized that height and weight gain among children aged 1 to 59 months would be greater in azithromycin-treated communities than in placebo-treated communities.

## Methods

### Study Design

MORDOR^1^ was a placebo-controlled, cluster-randomized trial conducted in Malawi, Niger, and Tanzania that found decreased childhood mortality in communities treated with mass azithromycin distributions. Because close monitoring of child health outcomes and any resulting treatments could affect the mortality outcome, we performed detailed health assessments in a separate clinical trial from December 2014 until March 2020 in which a smaller number of communities were randomized to the same treatment groups as in the parent trial. This parallel-design double-blind trial report presents the results of anthropometric measurements from the Niger site of that smaller trial. The methods were not changed after commencement of the trial. The trial was approved by ethical review boards at the University of California, San Francisco, and the Niger Ministry of Health. Oral informed consent was obtained from children’s guardians at each study visit. This study is reported following the Consolidated Standards of Reporting Trials (CONSORT) reporting guideline. The trial protocol is available in Supplement 1.

### Study Eligibility

**Figure 1.**
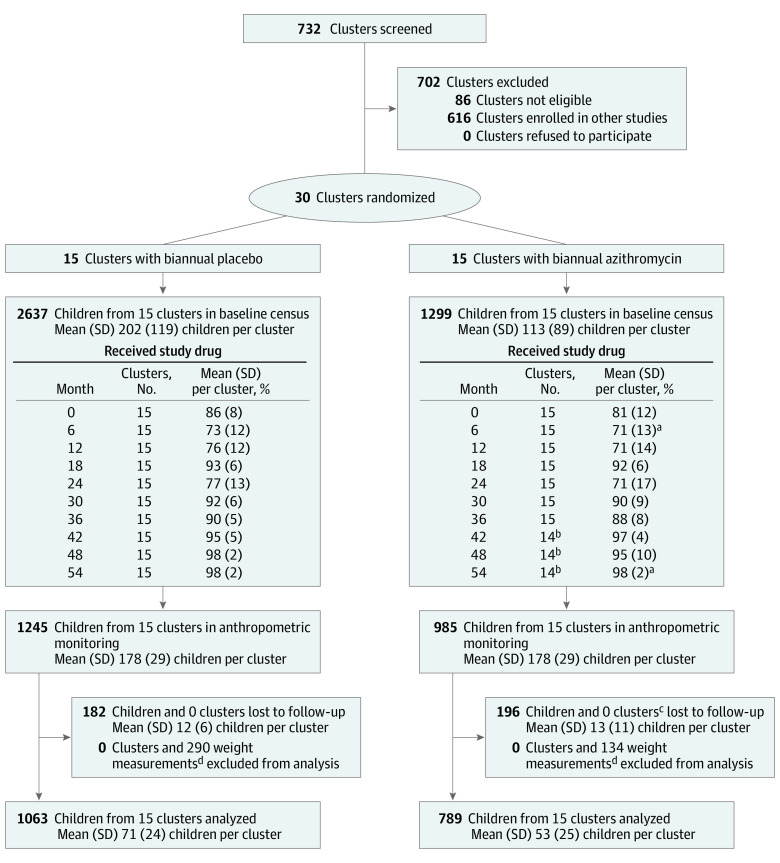
Study Flow Diagram There were 30 clusters drawn from the same pool of eligible communities as the main Macrolides Oraux pour Réduire les Décès avec un Oeil sur la Résistance (MORDOR) trial and randomized to biannual mass administration with azithromycin or placebo for 5 years. A random sample of children was selected from the baseline census for anthropometric monitoring and followed annually for 5 years. ^a^Data were missing from 1 entire community owing to technical problems; this community was known to be treated. Population and drug coverage estimates omit this community with missing data. ^b^One community refused participation after month 36 and was not included in estimates of population or drug coverage. ^c^One azithromycin cluster refused to participate after month 36, but follow-up data from earlier points was included in analyses. ^d^Measurements from a malfunctioning scale were excluded from 3 communities in the azithromycin group and 5 communities in the placebo group at the month 24 study visit.

The randomization unit for MORDOR-Niger was the “grappe,” a government-defined geographic area termed “community” for this report. Grappes in the Boboye and Loga departments with a population of 200 to 2000 individuals on the most recent (2012) government census were eligible for enrollment. Of 732 eligible communities in Niger, 30 were randomly allocated to this trial (Figure 1). All children aged younger than 60 months from each community were eligible for enrollment.

### Randomization and Masking

Communities were randomized in equal proportions to 1 of 6 treatment letters, with 3 letters corresponding to azithromycin and 3 to placebo. The randomization sequence was created in R statistical software version 3.1 (R Project for Statistical Computing) by the trial biostatistician (T.C.P.). A study coordinator (A.M.A.) enrolled the communities and assigned interventions. Allocation was concealed by randomizing all communities at the same time. Study participants and study staff were masked to treatment allocation.

### Eligibility for Monitoring Visits

A door-to-door census was conducted every 6 months during the study, as described previously.^1^ Anthropometric monitoring was performed at separate study visits at months 0 (ie, pretreatment), 12, 24, 36, 48, and 60, with monitoring visits attempted within 3 months of the preceding census. Anthropometric outcomes were collected in repeated cross-sectional random samples and a longitudinal sample. For repeated cross-sectional samples, 40 children aged 1 to 59 months per community were randomly selected from the most recent census prior to each annual monitoring visit. For the longitudinal sample, a group of 80 randomly selected children per community was chosen prior to the baseline visit, and these same children were measured at all subsequent visits. The longitudinal sample offered increased statistical power owing to repeated measurement of highly correlated anthropometric outcomes. However, children born after the baseline visit could not enter the longitudinal cohort, and thus this sampling strategy could miss longer-term growth effects of azithromycin on the youngest children. The repeated cross-sectional samples were incorporated to allow observation of the youngest children at follow-up visits.

### Anthropometric Assessment

A portable stadiometer (Schorr Productions) was used to measure standing height or recumbent length for children unable to stand. A Seca 874 floor scale (Seca GmbH & Co) was used for weight measurements. Scale calibration was monitored with standard weights. Height and weight were measured in triplicate, and the median value was used for analyses. Weight measurements were not possible in 8 communities at month 12 owing to a nonfunctioning scale. Mid–upper arm circumference (MUAC) was measured for referral purposes. Interanthropometrist agreement was excellent (eTable 1 in Supplement 2).

### Intervention

The study drug was administered every 6 months at the participant’s household. All children aged 1 to 59 months on the preceding census and weighing at least 3.8 kg were offered treatment. Azithromycin and placebo powder for suspension were provided by Pfizer in identically appearing bottles. Azithromycin was dosed at 20 mg/kg using height-based approximation or by weight for children unable to stand.^6^ Placebo contained the vehicle of azithromycin suspension. Treatment at months 0, 6, 12, 24, 36, and 48 was distributed after that study period’s monitoring visit, usually within 1 month. Treatment at months 18, 30, 42, and 54 was distributed at the time of the census. Guardians were instructed to contact a village representative if their child experienced any adverse events within 7 days of receiving the study drug; the representative then informed the study coordinator (A.M.A.). A formal adverse event survey was performed for children aged 1 to 5 months and is reported separately.^7^

### Outcomes

The 2 prespecified primary outcomes were height over time and weight corrected for height over time, assessed at the 48-month study visit. Weight was adjusted for height given the strong association between weight and limb length.^8^ End points at months 24 and 60 were also prespecified. We purposefully did not select *z* scores as the primary outcome because caregiver-reported ages were known to be imprecise, and calculation of anthropometric *z* scores was thus subject to considerable misclassification. Instead, the height-for-age *z* score (HAZ), weight-for-height *z* score (WHZ), weight-for-age *z* score (WAZ), and MUAC *z* score (MAZ) were calculated according to the 2006 World Health Organization (WHO) child growth standards and analyzed as a secondary outcome in the repeated cross-sectional samples of children.^9,10^

### Statistical Analysis

The longitudinal sample was used for the primary analysis, and repeated cross-sectional samples were used in a sensitivity analysis. Length measurements were converted to heights by subtracting 0.7 cm, as recommended by WHO.^10^ The prespecified primary outcome height was compared between groups in a repeated measures analysis of the 0-month to 48-month measurements, modeled in a mixed effects linear regression that included fixed effects for time and the time by treatment interaction, random intercepts for participants and study community, and a random slope for each study participant over time.^11^ The primary weight analysis was modeled similarly but additionally included height as a covariate. Study visits with missing data were omitted from analyses. Statistical significance was determined by Monte Carlo permutation testing of the coefficient for the interaction term (1000 permutations), with a significance level of .05 for each analysis. Subgroup analyses were performed based on height quartiles at baseline, chosen as a proxy for age given imprecision in self-reported age; median community size; and sex. Intraclass correlation coefficients (ICCs) were estimated from mixed effects regression models. Cluster-level *z* score summary statistics (ie, mean, proportion of children with *z* score less than −2 and proportion of children with *z* score less than −3) from cross-sectional samples were analyzed as a sensitivity analysis in mixed effects linear regression models with treatment group and baseline mean *z* score as a fixed effect and community as a random intercept. Bootstrapped 95% CIs were computed for all descriptive statistics and regression model coefficients, with resampling at the community level to account for the clustered design (9999 replications).

We estimated that including 40 children per community and 15 communities per study group would provide 80% power to detect a 1 cm difference between study groups assuming a mean (SD) of 90 (10) cm, an ICC of 0.02, a correlation coefficient of 0.94 for serial height measurements, equal cluster sizes, and a 2-sided α of .05.^12,13^ All analyses were performed in R version 4 (R Project for Statistical Computing). Data were analyzed from June through November 2021.

## Results

A total of 3936 children from 30 communities were enrolled at baseline (Figure 1). As reported previously, baseline characteristics were similar between 1299 children in the azithromycin group and 2637 children in the placebo group (mean 48.2% [95% CI, 45.5%-50.8%] girls vs 48.0% [95% CI, 45.7%-50.3%] girls; mean age, 30.8 months [95% CI, 29.5-32.0 months] vs 30.6 months [95% CI, 29.2-31.6 months]), except that mean size of communities in the azithromycin group was smaller (113 children [95% CI, 63-163 children] vs 202 children [95% CI, 136-268 children]) (Table 1).^14^ Across 10 mass drug distributions, 85.3% (95% CI, 78.7%-91.6%) of children from azithromycin-treated communities and 87.7% (95% CI, 82.0%-93.0%) of children from placebo-treated communities received study medication. No hospitalizations or life-threatening illnesses were reported in either group.

**Table 1.** Baseline Characteristics of Study Communities From a Population Census

Characteristic	Mean (95% CI)	
	Placebo communities (n = 15)	Azithromycin communities (n = 15)
Children aged 1-59 mo, No.	202 (136-268)	113 (63-163)
Age, %, y		
0	14.6 (11.9-17.2)	13.8 (11.3-16.4)
1	14.4 (12.3-16.6)	15.2 (12.4-18.0)
2	19.3 (17.5-21.1)	18.8 (16.0-21.6)
3	23.8 (21.4-26.3)	24.6 (21.7-27.5)
4	27.9 (24.1-31.7)	27.5 (24.3-30.7)
Overall, mean (95% CI), mo	30.6 (29.2-31.6)	30.8 (29.5-32.0)
Girls, %	48.0 (45.7-50.3)	48.2 (45.5-50.8)

At the pretreatment monitoring visit, 2230 children aged 1 to 59 months, including 985 children from the azithromycin group and 1245 children from the placebo group, had anthropometric assessment performed. Height and weight were similar at baseline (eFigure in Supplement 2), with minimal between-community clustering (ICC for height, 0.022; ICC for weight, 0.035). Repeat anthropometric assessment at a subsequent study visit over the 5-year study was performed among 789 children (80.1%) from the azithromycin group and 1063 children (85.4%) from the placebo group. Analysis of baseline characteristics of children lost to follow-up did not provide evidence of differential loss to follow-up between treatment group (eTable 2 in Supplement 2). A total of 71 children in the azithromycin group and 70 children in the placebo group died during the follow-up period, among whom 39 children and 42 children, respectively, did not contribute follow-up anthropometric data prior to death.

**Figure 2.**
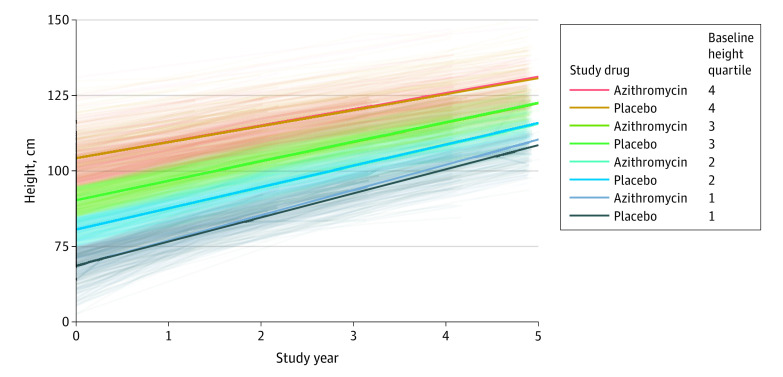
Height Over Time A random sample of children was monitored annually over 5 years in the azithromycin and placebo group. Each thin line indicates an individual child’s growth curve, colored according to the baseline quartile of height; heavy lines, mean of the treatment group in the respective subgroup.

At the prespecified 4-year follow-up visit, children in the azithromycin group gained a mean 6.7 cm (95% CI, 6.5 to 6.8 cm) in height and 1.7 kg (95% CI, 1.7 to 1.8 kg) in weight per year and children in the placebo group gained a mean 6.6 cm (95% CI, 6.4 to 6.7 cm) in height and 1.7 kg (95% CI, 1.7 to 1.8 kg) in weight per year. Estimated differences between treatment groups were small and not statistically significant. At the 4-year study visit, mean height in the azithromycin group was 0.08 cm (95% CI, −0.12 to 0.28 cm) greater than in the placebo group (*P* = .45 after adjustment for baseline height, prespecified primary outcome) and mean weight in the azithromycin group was 0.02 kg (95% CI, −0.10 to 0.06 kg) lower than in the placebo group (*P* = .64 after adjustment for height and baseline weight, prespecified primary outcome). Outcomes were similar for other prespecified end points (Table 2). Rates of linear growth over the study were related to baseline height, with the greatest growth rates seen among the smallest quartile of children (eTable 3 in Supplement 2). In subgroup analyses, children in the smallest quartile of baseline height randomized to the mass azithromycin group were 0.4 cm (95% CI, 0.1 to 0.7 cm) taller at 4 years compared with those randomized to the placebo group (Figure 2). No significant effects were observed for height or weight in other subgroups based on sex or community size (Table 3). Across all monitoring visits of the longitudinal sample, 13 children in the azithromycin group and 9 children in the placebo group had a MUAC less than 11.5 cm and were referred to a local health facility for treatment.

**Table 2.** Estimated Rates of Height and Weight Gain

End point	Growth rate				Difference^b^
	Placebo		Azithromycin		
	Children, No.^a^	Mean (95% CI)	Children, No.^a^	Mean (95% CI)	
**Height, cm/y**					
Month 24	957	7.1 (6.9 to 7.4)	704	7.3 (7.0 to 7.5)	0.07 (−0.19 to 0.32)
Month 48	1057	6.6 (6.4 to 6.7)	782	6.7 (6.5 to 6.8)	0.08 (−0.12 to 0.28)
Month 60	1063	6.4 (6.3 to 6.6)	789	6.5 (6.4 to 6.7)	0.07 (−0.12 to 0.26)
**Weight, kg/y**					
Month 24	871	1.7 (1.7 to 1.8)	664	1.7 (1.6 to 1.8)	−.03 (−0.12 to 0.07)
Month 48	1027	1.7 (1.7 to 1.8)	765	1.7 (1.7 to 1.8)	−.02 (−0.10 to 0.06)
Month 60	1038	1.8 (1.7 to 1.8)	775	1.7 (1.7 to 1.8)	−.02 (−0.10 to 0.06)

^a^ Number of children aged 1 to 59 months at baseline contributing data to longitudinal analysis. Models of later times included larger numbers of children because all study visits up to the end point were included in repeated measure models, including from children lost to follow-up before the end point.

^b^ Positive numbers indicate larger estimates in the azithromycin group; all analyses are adjusted for baseline values, and weight analyses are also adjusted for concurrent height.

**Table 3.** Subgroup Analyses for Month 48 Outcomes

Subgroup	Children, No.^a^	Height, cm		Children, No.^a^	Weight, kg	
		Azithromycin (vs placebo)^b^	*P* value		Azithromycin (vs placebo)^b^	*P* value
Baseline height, cm						
<75	397	0.4 (0.1 to 0.7)	.03	388	−0.03 (−0.11 to 0.05)	.32
75-84.9	416	−0.1 (−0.3 to 0.2)	.68	402	−0.004 (−0.07 to 0.08)	.91
85-94.9	455	−0.03 (−0.2 to 0.1)	.71	441	−0.01 (−0.07 to 0.09)	.82
≥95	571	0.1 (−0.1 to 0.3)	.20	561	−0.06 (−0.17 to 0.05)	.27
Sex						
Girls	878	0.3 (−0.2 to 0.2)	.71	855	−0.005 (−0.09 to 0.09)	.92
Boys	961	0.1 (−0.1 to 0.3)	.45	937	−0.05 (−0.13 to 0.04)	.32
Community size, No. children						
<150	882	0.1 (−0.3 to 0.4)	.71	872	0.002 (−0.12 to 0.13)	.98
≥150	957	0.0004 (−0.3 to 0.3)	.99	920	−0.03 (−0.16 to 0.10)	.67

^a^ Number of children aged 1-59 months at baseline contributing data to longitudinal analysis.

^b^ Positive numbers indicate larger estimates in the azithromycin group; all analyses adjusted for baseline values and weight analyses also adjusted for concurrent height.

In a secondary analysis, HAZ, WAZ, WHZ, and MAZ were compared between groups from randomly chosen cross-sectional samples of children at each study visit (eTable 4 in Supplement 2). Across 4 prespecified follow-up visits, 30.2% (95%CI 25.4 to 34.89%) of children were classified with stunting (ie, HAZ < −2) and 10.7% (95%CI 7.0 to 15.6%) of children with wasting (ie, WHZ < −2). No statistically significant difference was observed between groups for any *z* score estimate over 4 years of follow-up (eTable 5 in Supplement 2).

## Discussion

This parallel-design double-blind trial randomized communities to biannual mass treatment with azithromycin or placebo and then assessed for growth in a random subset of preschool children over 5 years. No improvement in mean rate of height or weight gain was observed in the clusters given mass azithromycin distributions, although height gain was greater in the azithromycin-treated communities specifically for the shortest quartile of children. The proportion of children with stunting or wasting, as assessed by WHO standards, was not significantly different between groups at 4 years.

Several previous studies have suggested that antibiotic therapy may improve growth when given in certain clinical scenarios.^15^ A randomized trial^16,17^ studying children in Zambia with HIV found that daily cotrimoxazole therapy resulted in improved linear growth and decreased mortality. A randomized trial^18^ in Malawi found that antibiotics improved childhood growth and decreased mortality when given in the setting of severe acute malnutrition, although other similar trials^19,20^ have found no benefit. A randomized trial^21^ from Burkina Faso found greater weight gain among children administered a single dose of azithromycin at 14 days but not at 6 months.

It is biologically plausible that azithromycin distributions could improve childhood growth. A broad-spectrum, long-acting antibiotic like azithromycin could treat or prevent bacterial diarrhea, a major cause of malnutrition in resource-limited settings. Bacterial infections of the gastrointestinal tract are thought to play a role in causing environmental enteropathy, and some researchers have theorized that antibiotics could ameliorate this condition.^22^ Azithromycin has been shown to alter the gastrointestinal microbiota, which could theoretically impact nutrient absorption.^23^ Infections, in general, are thought to increase the metabolic demands of the immune system, so decreasing the incidence and duration of infectious illnesses could leave more metabolic resources available for growth.

The MORDOR trial^1^ demonstrated a 14% decrease in childhood mortality in communities receiving biannual mass azithromycin distributions to children ages 1 to 59 months, with the greatest effect seen among children ages 1 to 5 months. Mass azithromycin decreased mortality despite selecting for macrolide resistance.^24^ The mechanism for this decrease in mortality is unclear, although verbal autopsies showed fewer deaths due to malaria, dysentery, meningitis, and pneumonia in the azithromycin-treated group and blood smears revealed less malaria parasitemia among individuals in the azithromycin group.^14,25^ Other than a potential direct effect on infection, it is also conceivable that children receiving antibiotics may experience less stunting and malnutrition, which could in turn convey a survival benefit. Our study’s central finding, that the growth rate of preschool children was similar in the azithromycin and placebo groups, argues against growth promotion as the sole mediator of azithromycin’s effect on child mortality. However, subgroup analyses stratified by baseline height (ie, a proxy for age given imprecise caregiver-reported ages) revealed that mass azithromycin distributions were associated with increased height gain among children in the shortest quartile; this is an intriguing finding considering the strongest mortality effect of azithromycin was among the youngest children.^1^

Several randomized studies have assessed anthropometric outcomes in the context of mass azithromycin. Studies conducted in Ethiopia, The Gambia, and Niger randomized communities to differing frequencies of mass azithromycin distributions and found no difference in stunting or wasting between the 2 groups.^12,13,26,27^ However, these studies did not measure anthropometry in the same children before and after treatment and distributed at least some azithromycin to all study communities, making it more difficult to detect an effect. Our trial may have overcome these limitations because control communities received placebo in a masked fashion and a baseline sample of children was followed longitudinally.

The null primary result of the present study could have several interpretations. The most likely explanation is that biannual mass azithromycin distributions do not have a clinically meaningful effect on childhood growth over a 5-year period, consequently suggesting that the mortality benefit seen in the Niger site of MORDOR was not due to improvements in growth. However, given our findings in the subgroup with children in the shortest quartile, it is also possible that mass azithromycin distributions do impact childhood growth but only among young children. Such an interpretation is plausible because most children developing linear growth failure do so in the first few months of life, with few ever experiencing reversal of stunting.^28^ Alternatively, it is possible that mass azithromycin affects childhood growth at a lower magnitude than detectable by our study or that any growth effects of mass azithromycin depend on an unmeasured effect modifier (eg, adequate nutrient support or concurrent infectious disease). When used for animal husbandry, antibiotics are thought to improve growth by 10% to 15%.^29^ A 2014 meta-analysis^15^ estimated that antibiotic therapy may improve growth among children who have illnesses by 0.04 cm per month, similar to our study’s results in the shortest quartile. Thus, it is conceivable that antibiotics have a small yet real effect on growth. Future studies could be designed with larger sample sizes of children under age 12 months or with individual-level randomization, each of which would increase the power to detect a smaller effect of mass azithromycin on childhood growth.^30^

### Limitations

Several limitations of the study should be noted. Coverage of the study drug was not universal, although mean coverage exceeded the 80% target recommended by many mass drug administration programs. Approximately one-fifth of children were lost to follow-up, raising the possibility for bias if the loss to follow-up was differential between study groups. The prospect of differential loss to follow-up should have been decreased by the use of a placebo and the efforts to mask participants and study personnel. Imbalance in the population size of placebo vs azithromycin communities raises the possibility of confounding, although the age and sex distribution was similar by group. Age was not part of the primary outcome, but it is important to note that precise age measurements were not readily available for many children, increasing the chances of misclassification, although not bias, for age-based anthropometric *z* scores (ie, HAZ and WAZ). Moreover, height and weight can be challenging to measure among small children. Observed interanthropometrist agreement was high, but it is still possible that measurement error resulted in some degree of misclassification; however, differential misclassification by treatment group would be unlikely. One of the scales was not functional for a period during the 12-month assessments, leading to incomplete data for this secondary end point. Additionally, the study was performed in Niger, where malnutrition is relatively common. The generalizability to other settings is not clear.

## Conclusions

This study found that biannual mass azithromycin distributions did not significantly impact growth rates in the setting of a placebo-controlled cluster-randomized trial in Niger, although subgroup analyses found a possible benefit among children in the shortest quartile. These findings suggest that the decrease in mortality conveyed by mass azithromycin distributions is likely not caused solely by growth promotion.

## Notes

Supplement 1.
Trial Protocol

Supplement 2. **eTable 1.** Reproducibility of Anthropometric Measurements **eTable 2.** Baseline Characteristics of Participants Selected for Anthropometry Stratified by Loss to Follow-Up **eTable 3.** Estimates of Rates of Height and Weight Gain Over 4 Years by Baseline Height **eTable 4.** Mean Anthropometric *z* Scores in Repeated Cross-sectional Random Samples of Children Aged 1-59 mo Over 4 Years of Follow-up **eTable 5.** Extreme *z* Scores in Repeated Cross-sectional Random Samples of Children Aged 1-59 mo Over 4 Years of Follow-up **eFigure.** Height vs Weight at Baseline

Supplement 3. **Nonauthor Collaborators.** MORDOR-Niger Study Group

Supplement 4.
Data Sharing Statement
