# Supplementary material for: Effect of Mass Azithromycin Distributions on Childhood Growth in Niger: A Cluster-Randomized Trial
Source: JAMA Netw Open. 2021 Dec 30;4(12):e2139351. doi: 10.1001/jamanetworkopen.2021.39351 (PMC8719241; doi:10.1001/jamanetworkopen.2021.39351)
Supplement: Supplement 2. — eTable 1. Reproducibility of Anthropometric Measurements eTable 2. Baseline Characteristics of Participants Selected for Anthropometry Stratified by Loss to Follow-Up eTable 3. Estimates of Rates of Height and Weight Gain Over 4 Years by Baseline Height eTable 4. Mean Anthropometric z Scores in Repeated Cross-sectional Random Samples of Children Aged 1-59 mo Over 4 Years of Follow-up eTable 5. Extreme z Scores in Repeated Cross-sectional Random Samples of Children Aged 1-59 mo Over 4 Years of Follow-up eFigure. Height vs Weight at Baseline [file jamanetwopen-e2139351-s002.pdf]

Supplemental Online Content

Arzika AM, Maliki R, Ali MM, et al; MORDOR-Niger Study Group. Effect of mass azithromycin distributions on childhood growth in Niger: a cluster-randomized trial. *JAMA Netw Open*. 2021;4(12):e2139351.
doi:10.1001/jamanetworkopen.2021.39351

eTable 1. Reproducibility of Anthropometric Measurements
eTable 2. Baseline Characteristics of Participants Selected for Anthropometry Stratified by Loss to Follow-Up
eTable 3. Estimates of Rates of Height and Weight Gain Over 4 Years by Baseline Height
eTable 4. Mean Anthropometric *z* Scores in Repeated Cross-sectional Random Samples of Children Aged 1-59 mo Over 4 Years of Follow-up
eTable 5. Extreme *z* Scores in Repeated Cross-sectional Random Samples of Children Aged 1-59 mo Over 4 Years of Follow-up
eFigure. Height vs Weight at Baseline

This supplemental material has been provided by the authors to give readers additional information about their work.

eTable 1. Reproducibility of Anthropometric Measurements
A convenience sample of 93 children (median caregiver-reported age 36 months, interquartile range 24-48 months) had anthropometric measurements performed by two different teams of study workers.

Measurement	Mean value (95%CI)	ICC (95%CI)
Height	87.2 (84.5-90.0)	0.999 (0.998-0.999)
Weight	11.6 (10.9-12.3)	0.999 (0.998-0.999)
MUAC	14.6 (14.4-14.9)	0.887 (0.834-0.924)

MUAC=mid-upper arm circumference

eTable 2. Baseline Characteristics of Participants Selected for Anthropometry Stratified by Loss to Follow-Up

	Lost at Month 12		Lost at Month 24		Lost at Month 36		Lost at Month 48		Lost at Month 60		Not lost to follow-up		
Baseline characteristic	Placebo n=182	Azithro n=196	Placebo n=77	Azithro n=65	Placebo N=96	Azithro n=92	Placebo n=91	Azithro n=66	Placebo n=240	Azithro n=171	Placebo n=559	Azithro n=395	P-value[a]
Mean age, mos	30 (16)	31 (15)	29 (17)	30 (16)	32 (16)	32 (15)	29 (17)	32 (17)	32 (16)	33 (16)	32 (16)	31 (16)	0.28
Age													
0y	21 (12%)	21 (11%)	13 (17%)	12 (18%)	12 (13%)	9 (10%)	13 (14%)	9 (14%)	38 (16%)	21 (12%)	67 (12%)	56 (14%)	
1y	34 (19%)	29 (15%)	15 (19%)	9 (14%)	12 (13%)	12 (13%)	18 (20%)	7 (11%)	27 (11%)	22 (13%)	70 (13%)	54 (14%)	
2y	39 (21%)	47 (24%)	8 (10%)	16 (25%)	20 (21%)	21 (23%)	20 (22%)	9 (14%)	39 (16%)	30 (18%)	109 (19%)	63 (16%)	0.24
3y	44 (24%)	40 (20%)	21 (27%)	12 (18%)	22 (23%)	24 (26%)	17 (19%)	17 (26%)	60 (25%)	38 (22%)	132 (24%)	110 (28%)	
4y	43 (24%)	59 (30%)	20 (26%)	16 (25%)	30 (31%)	26 (28%)	23 (25%)	23 (35%)	76 (32%)	59 (35%)	181 (32%)	112 (28%)	
Mean height, cm	87 (15)	86 (14)	87 (15)	85 (15)	87 (14)	88 (15)	85 (15)	89 (15)	88 (15)	88 (15)	88 (14)	87 (13)	0.73
Mean weight, kg	11 (4)	11 (4)	12 (4)	11 (4)	11 (3)	12 (4)	11 (4)	12 (4)	12 (4)	12 (4)	12 (3)	12 (3)	0.81
Height quartile													
< 75 cm	40 (22%)	54 (28%)	21 (27%)	19 (29%)	18 (19%)	18 (20%)	21 (23%)	12 (18%)	50 (21%)	38 (22%)	113 (20%)	89 (23%)	
75-84.9 cm	49 (27%)	44 (22%)	15 (19%)	15 (23%)	30 (31%)	24 (26%)	28 (31%)	13 (20%)	55 (23%)	31 (18%)	125 (22%)	85 (22%)	0.94
85-94.9 cm	43 (24%)	44 (22%)	17 (22%)	16 (25%)	21 (22%)	15 (16%)	16 (18%)	15 (23%)	54 (23%)	51 (30%)	142 (25%)	111 (28%)	
≥ 95 cm	50 (27%)	54 (28%)	24 (31%)	15 (23%)	27 (28%)	35 (38%)	26 (29%)	26 (39%)	81 (34%)	51 (30%)	179 (32%)	110 (28%)	
Sex													
Female	89 (49%)	92 (47%)	35 (46%)	35 (54%)	46 (48%)	41 (45%)	42 (46%)	33 (50%)	108 (45%)	86 (50%)	276 (49%)	184 (47%)	0.77
Male	93 (51%)	104 (53%)	42 (55%)	30 (46%)	50 (52%)	51 (55%)	49 (54%)	33 (50%)	132 (55%)	85 (50%)	283 (51%)	211 (53%)	

Values indicate mean (standard deviation) or numbers (proportion). Age was reported by caregivers.

[a] Baseline characteristics modeled in mixed effects regression models with fixed effects for (i) the study visit at which loss to follow-up occurred, (ii) treatment arm, and (iii) the interaction between study visit and treatment arm. The p-value from the interaction term is shown here, indicating whether loss to follow-up over the duration of the study was different between the two treatment arms.

eTable 3. Estimates of Rates of Height and Weight Gain Over 4 Years by Baseline Height

	Height, mean cm/year				Weight, kg/year			
	Placebo		Azithromycin		Placebo		Azithromycin	
Baseline height	No.[a]	Mean (95%CI)	No.	Mean (95%CI)	No.[a]	Mean (95%CI)	No.	Mean (95%CI)
< 75 cm	222	8.0 (7.8-8.3)	175	8.3 (8.0-8.6)	215	1.9 (1.8-1.9)	173	1.9 (1.8-2.0)
75-84.9 cm	251	7.0 (6.8-7.3)	165	7.0 (6.8-7.2)	246	1.7 (1.6-1.7)	156	1.7 (1.6-1.7)
85-94.9 cm	249	6.4 (6.3-6.6)	206	6.4 (6.3-6.5)	240	1.6 (1.5-1.6)	201	1.6 (1.5-1.7)
≥ 95 cm	335	5.3 (5.2-5.4)	236	5.4 (5.3-5.6)	326	1.7 (1.6-1.8)	235	1.7 (1.6-1.8)

[a] Number of children contributing baseline and follow-up data; weight values lower because of a nonfunctioning scale affecting 8 communities at the month 12 visit

eTable 4. Mean Anthropometric *z* Scores in Repeated Cross-sectional Random Samples of Children Aged 1-59 mo Over 4 Years of Follow-up

	Placebo					Azithromycin						
	No. per visit					No. per visit					Adjusted	P-
Z-score	12	24	36	48	Mean (95%CI)[a]	12	24	36	48	Mean (95%CI)[a]	difference[b]	value[b]
HAZ	550	589	569	554	-1.14 (-1.32 to -0.98)	568	568	529	517	-1.19 (-1.35 to -1.04)	-0.01 (-0.24 to 0.22)	0.91
WAZ	360	589	569	554	-1.16 (-1.36 to -0.99)	451	568	529	517	-1.19 (-1.40 to -1.00)	-0.01 (-0.26 to 0.25)	0.96
WHZ[c]	357	588	568	553	-0.66 (-0.86 to -0.52)	449	568	529	517	-0.72 (-0.93 to -0.54)	-0.02 (-0.29 to 0.24)	0.85
MAZ[d]	538	575	557	537	-1.09 (-1.29 to -0.95)	558	558	511	505	-1.06 (-1.29 to -0.87)	0.001 (-0.19 to 0.19)	0.99

HAZ=height-for-age Z-score, MAZ=mid-upper arm circumference Z-score, WAZ=weight-for-age Z-score, WHZ=weight-for-height Z-score, calculated using caregiver-reported age in months.

[a] Values represent cluster-level averages across the 4 pre-specified annual follow-up visits (i.e., months 12 to 48).

[b] Cluster-level means or proportions for each follow-up study time point (i.e., months 12 to 48) were modeled in a mixed effects regression model adjusted for the mean Z score at baseline and including a random intercept for study cluster. Positive values indicate greater growth indicators in the azithromycin arm relative to the placebo arm.

[c] WHZ values are only calculated for children between 65-120cm.

[d] MAZ values are only calculated for children 3 months and older.

eTable 5. Extreme *z* Scores in Repeated Cross-sectional Random Samples of Children Aged 1-59 mo Over 4 Years of Follow-up

	Mean proportion (95%CI)[a]		Adjusted	P-
Binary Z-scores	Placebo	Azithromycin	difference[b]	value[b]
HAZ < -2 SD	30% (25-35%)	30% (26-35%)	-0.01 (-0.07 to 0.06)	0.82
WAZ < -2 SD	23% (18-29%)	23% (18-29%)	-0.003 (-0.08 to 0.07)	0.92
WHZ < -2 SD[c]	11% (7-16%)	11% (7-15%)	-0.01 (-0.07 to 0.05)	0.72
MAZ < -2 SD[d]	19% (15-25%)	18% (12-25%)	-0.004 (-0.06 to 0.05)	0.87
HAZ < -3 SD	10% (8-13%)	11% (8-14%)	-0.001 (-0.04 to 0.03)	0.96
WAZ < -3 SD	6% (4-9%)	6% (4-9%)	-0.01 (-0.04 to 0.03)	0.77
WHZ < -3 SD[c]	2% (1-4%)	2% (1-4%)	0.0003 (-0.02 to 0.02)	0.97
MAZ < -3 SD[d]	5% (3-7%)	4% (2-7%)	-0.003 (-0.02 to 0.02)	0.77

HAZ=height-for-age Z-score, MAZ=mid-upper arm circumference Z-score, SD= standard deviations, WAZ=weight-for-age Z-score, WHZ=weight-for-height Z-score, calculated using caregiver-reported age in months.

[a] Values represent cluster-level averages across the 4 pre-specified annual follow-up visits (i.e., months 12 to 48).

[b] Cluster-level means or proportions for each follow-up study time point (i.e., months 12 to 48) were modeled in a mixed effects regression model adjusted for the mean Z score at baseline and including a random intercept for study cluster. Positive values indicate greater growth indicators in the azithromycin arm relative to the placebo arm. The numbers of observations included in the analysis are provided in the preceding table (eTable 3).

[c] WHZ values are only calculated for children between 65-120cm.

[d] MAZ values are only calculated for children 3 months and older.

© 2021 Arzika AM et al. *JAMA Network Open.*

eFigure. Height vs Weight at Baseline
Each dot represents a child, and the lines represent lowess curves for each arm. Azithromycin-treated communities are orange and placebo-treated communities are blue.

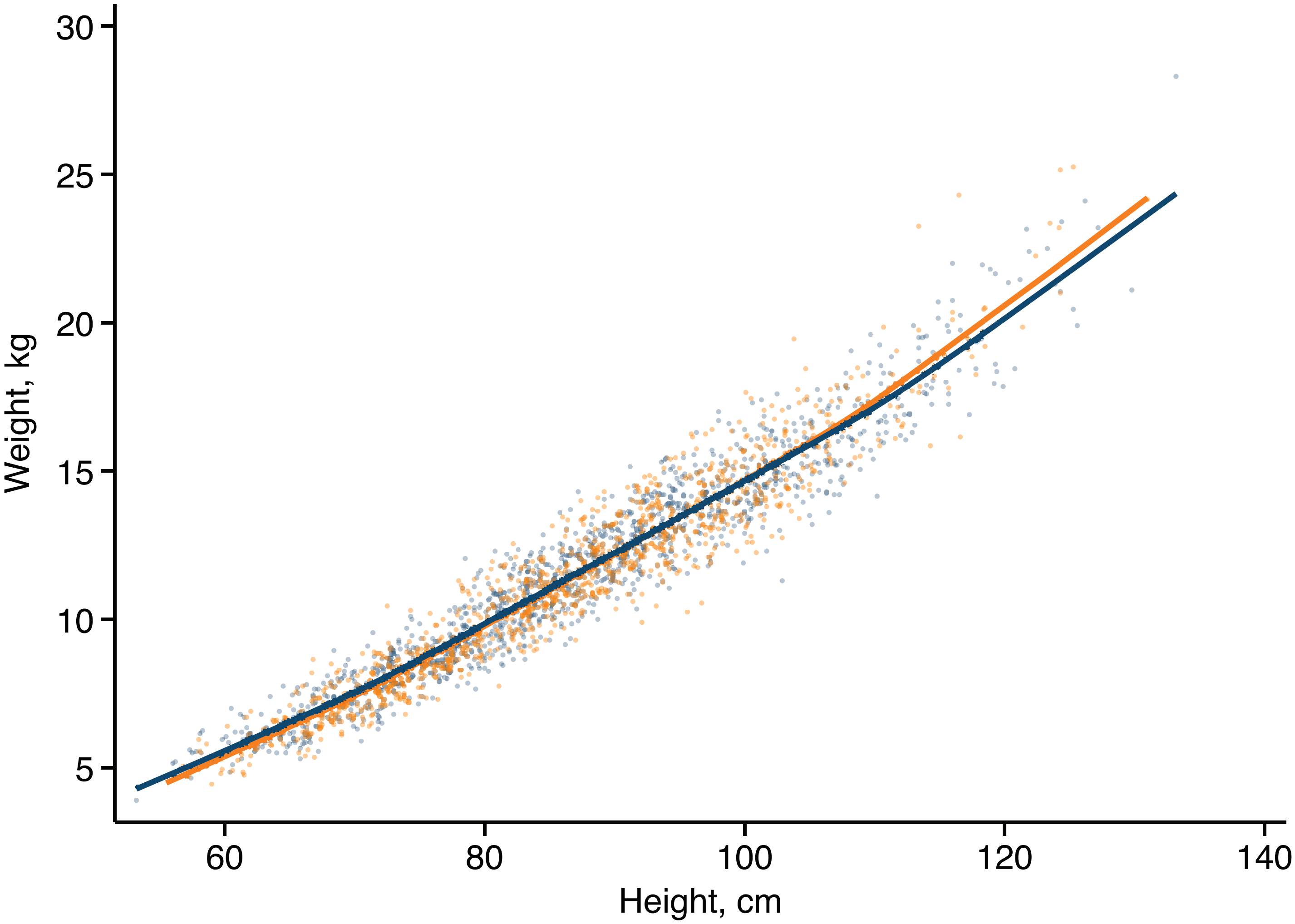

© 2021 Arzika AM et al. *JAMA Network Open.*
