# Supplementary material for: Effect of Mass Azithromycin Distributions on Childhood Growth in Niger: A Cluster-Randomized Trial
Source: JAMA Netw Open. 2021 Dec 30;4(12):e2139351. doi: 10.1001/jamanetworkopen.2021.39351 (PMC8719241; doi:10.1001/jamanetworkopen.2021.39351)
Supplement: Supplement 4. — Data Sharing Statement [file jamanetwopen-e2139351-s004.pdf]

Data Sharing Statement

Arzika AM, Maliki R, Ali MM, et al. Effect of mass azithromycin distributions on childhood growth in Niger: a cluster-randomized trial. *JAMA Netw Open*. 2021;4(12):e2139351. doi:10.1001/jamanetworkopen.2021.39351

Data

Data available: Yes
Data types: Deidentified participant data
How to access data: Supplemental file to this report
When available: With publication

Supporting Documents

Document types: None

Additional Information

Who can access the data: Anyone
Types of analyses: Any purpose
Mechanisms of data availability: Data available with the publication
Any additional restrictions: None

© 2021 Arzika AM et al. *JAMA Network Open*.

cluster_id	child_id	studymonth	arm	female	agemos	testdate	heightcorrected	heightmethod	weight	muac	monthofdeath	crosssectional	largevillage
1	2	0	Placebo	0	48	3/24/2015	96.1	HEIGHT	15.55	15.5		1	1
1	2	36	Placebo	0	80	5/20/2018	113.7	HEIGHT	20.68181818	16		0	1
1	3	12	Placebo	0	8	7/18/2016	70.7	LENGTH	7.75	13		1	1
1	3	48	Placebo	0	40	4/21/2019	94.2	HEIGHT	12.75	14		0	1
1	3	60	Placebo	0	50	2/26/2020	99.6	HEIGHT	14.4	13.7		0	1
1	4	12	Placebo	1	7	7/18/2016	66.5	LENGTH	6.25	12.5		0	1
1	4	36	Placebo	1	29	5/20/2018	84.2	HEIGHT	10.68181818	15		0	1
1	5	0	Placebo	1	24	6/11/2015	83	HEIGHT	9.1	12.5		1	1
1	5	24	Placebo	1	49	3/28/2017	98.4	HEIGHT	12.55	13.5		0	1
1	5	36	Placebo	1	64	5/20/2018	106	HEIGHT	14.86363636	14		0	1
1	5	48	Placebo	1	75	4/21/2019	108.5	LENGTH	16.15	14.5		0	1
1	5	60	Placebo	1	84	2/26/2020	115.2	HEIGHT	16.95	13.6		0	1
1	6	24	Placebo	0	8	3/30/2017	69.7	LENGTH	7.6	13.5		1	1
1	6	36	Placebo	0	23	5/20/2018	82.6	HEIGHT	9.818181818	13.5		0	1
1	6	48	Placebo	0	34	4/21/2019	89.6	HEIGHT	11.3	13.5		0	1
1	6	60	Placebo	0	43	2/26/2020	97.5	HEIGHT	13.15	14.7		0	1
1	8	0	Placebo	0	36	3/24/2015	83.4	HEIGHT	11.75	16		1	1
1	8	24	Placebo	0	61	3/28/2017	96.8	HEIGHT	15	16		0	1
1	8	48	Placebo	0	87	4/21/2019	107.1	HEIGHT	16.65	15		0	1
1	9	0	Placebo	0	48	3/24/2015	101.1	HEIGHT	15.2	14.5		1	1
1	9	48	Placebo	0	99	4/21/2019	121.2	HEIGHT	22	15.5		0	1
1	10	12	Placebo	0	23	7/18/2016	79.5	HEIGHT	10.2	14		1	1
1	11	0	Placebo	0	36	3/24/2015	92.2	HEIGHT	14.15	16		1	1
1	11	48	Placebo	0	86	4/21/2019	100.7	HEIGHT	15.8	14.5		0	1
1	11	60	Placebo	0	95	2/26/2020	124.2	HEIGHT	25.5	16.8		0	1
1	12	0	Placebo	0	24	3/24/2015	80.8	LENGTH	9.2	13		0	1
1	12	12	Placebo	0	38	7/18/2016	88.6	HEIGHT	10.95	14		0	1
1	12	24	Placebo	0	49	3/28/2017	92.9	HEIGHT	12.35	13.5		0	1
1	12	48	Placebo	0	75	4/21/2019	102.8	HEIGHT	14.9	14		0	1
1	12	60	Placebo	0	84	2/26/2020	108.3	HEIGHT	16.3	14.5		0	1
1	14	24	Placebo	0	53	3/30/2017	100.9	HEIGHT	15.75	15.5		1	1
1	18	12	Placebo	0	14	7/18/2016	71.4	LENGTH	7.85	13		1	1
1	18	48	Placebo	0	51	4/21/2019	92.2	HEIGHT	13.05	14		1	1
1	19	12	Placebo	1	11	7/18/2016	75	LENGTH	7.95	13.5		0	1
1	19	24	Placebo	1	20	3/28/2017	82.8	HEIGHT	9.45	13		0	1
1	22	0	Placebo	1	54	3/24/2015	119.3	HEIGHT	21.65	16		0	1
1	22	24	Placebo	1	79	3/28/2017	128.2	HEIGHT	24.65	16.5		0	1
1	22	36	Placebo	1	94	5/20/2018	133.2	HEIGHT	27.22727273	17		0	1
1	22	48	Placebo	1	105	4/21/2019	135.8	HEIGHT	30.3	18.5		0	1

1	29	12	Placebo	1	4	7/18/2016	67.9	LENGTH	7	12.5		0	1
1	29	36	Placebo	1	30	5/20/2018	88.3	HEIGHT	11.77272727	14.5		0	1
1	30	0	Placebo	1	54	3/24/2015	91.2	HEIGHT	13.55	15.5		0	1
1	30	12	Placebo	1	56	7/18/2016	101.2	HEIGHT	15	15.5		0	1
1	30	24	Placebo	1	67	3/28/2017	105.3	HEIGHT	16.4	15		0	1
1	31	0	Placebo	0	36	6/11/2015	91.9	HEIGHT	11.65	13.5		1	1
1	31	36	Placebo	0	76	5/20/2018	108.9	HEIGHT	17.5	15		0	1
1	31	48	Placebo	0	87	4/21/2019	113.2	HEIGHT	18.35	15		0	1
1	32	12	Placebo	0	54	7/18/2016	93.3	HEIGHT	14.6	16		1	1
1	33	0	Placebo	0	30	3/24/2015	91.3	HEIGHT	13.6	15		0	1
1	33	48	Placebo	0	81	4/21/2019	117.9	HEIGHT	21.05	16.5		0	1
1	35	0	Placebo	0	12	3/24/2015	75.2	LENGTH	8.2	12.5		0	1
1	35	12	Placebo	0	27	7/18/2016	84.4	HEIGHT	10.1	13.5		0	1
1	39	0	Placebo	0	12	3/24/2015	75.7	LENGTH	8.6	14		1	1
1	39	12	Placebo	0	26	7/18/2016	86.5	HEIGHT	11.6	14.5		0	1
1	39	60	Placebo	0	77	2/26/2020	111	HEIGHT	17.3	11.9		0	1
1	40	0	Placebo	0	1	3/24/2015	62.5	LENGTH	6.35	13.5		0	1
1	40	24	Placebo	0	25	3/28/2017	84.6	HEIGHT	12	15		0	1
1	40	36	Placebo	0	40	5/20/2018	93.5	HEIGHT	14.63636364	16		0	1
1	41	24	Placebo	1	24	3/30/2017	75	HEIGHT	8.8	13.5		1	1
1	43	0	Placebo	1	42	3/24/2015	101.6	HEIGHT	15.3	15		0	1
1	43	12	Placebo	1	56	7/18/2016	107.6	HEIGHT	17.5	15		0	1
1	44	0	Placebo	0	4	3/24/2015	64.6	LENGTH	6.7	13.5		1	1
1	44	24	Placebo	0	29	3/28/2017	83.7	HEIGHT	11.5	13.5		0	1
1	44	48	Placebo	0	55	4/21/2019	98.8	HEIGHT	15.3	14		0	1
1	45	24	Placebo	0	49	3/28/2017	103.4	HEIGHT	14.75	14		1	1
1	47	0	Placebo	0	54	3/24/2015	103.4	HEIGHT	16.05	15		0	1
1	47	36	Placebo	0	101	5/20/2018	120.8	HEIGHT	21.95454545	16		0	1
1	47	48	Placebo	0	112	4/21/2019	123.8	HEIGHT	22.9	16.5		0	1
1	47	60	Placebo	0	121	2/26/2020	128.3	HEIGHT	24.8	16.6		0	1
1	50	12	Placebo	1	9	7/18/2016	72.5	HEIGHT	7.3	13		0	1
1	50	24	Placebo	1	14	3/28/2017	81	HEIGHT	9.9	13.5		0	1
1	52	12	Placebo	0	11	7/18/2016	79.7	LENGTH	9.25	13	36	0	1
1	52	24	Placebo	0	20	3/28/2017	87	HEIGHT	11.3	12.5	36	0	1
1	55	0	Placebo	0	54	6/11/2015	110.4	HEIGHT	17.45	14.5		1	1
1	55	24	Placebo	0	82	3/28/2017	120.7	HEIGHT	22.65	15		0	1
1	55	48	Placebo	0	105	4/21/2019	127.8	LENGTH	25.1	16		0	1
1	55	60	Placebo	0	114	2/26/2020	134.1	HEIGHT	27.7	16		0	1
1	56	0	Placebo	1	13	3/24/2015	75	LENGTH	8.5	14.5		0	1
1	56	36	Placebo	1	56	5/20/2018	100.9	HEIGHT	14.54545455	15		0	1

1	56	48	Placebo	1	67	4/21/2019	107.1	HEIGHT	16.75	15		0	1
1	56	60	Placebo	1	76	2/26/2020	112.5	HEIGHT	17.85	14.5		0	1
1	59	0	Placebo	1	36	6/11/2015	84.7	HEIGHT	11.85	14.5		1	1
1	59	24	Placebo	1	41	3/28/2017	98.8	HEIGHT	14.8	15		0	1
1	59	36	Placebo	1	56	5/20/2018	104.9	HEIGHT	16	14.5		0	1
1	59	48	Placebo	1	67	4/21/2019	109.9	HEIGHT	16.95	14.5		0	1
1	59	60	Placebo	1	76	2/26/2020	114.5	HEIGHT	18.7	14.7		0	1
1	60	0	Placebo	1	12	6/11/2015	72.8	LENGTH	9	15		0	1
1	60	12	Placebo	1	18	7/18/2016	84.7	HEIGHT	11.3	14.5		0	1
1	61	0	Placebo	0	36	3/24/2015	88.6	HEIGHT	12.3	14.5		0	1
1	61	12	Placebo	0	50	7/18/2016	98.9	HEIGHT	14.5	14.5		1	1
1	61	24	Placebo	0	72	3/30/2017	104.8	HEIGHT	15.5	14.5		0	1
1	61	36	Placebo	0	87	5/20/2018	112.3	HEIGHT	17.54545455	13.5		0	1
1	61	48	Placebo	0	98	4/21/2019	113.8	LENGTH	19.15	14		0	1
1	61	60	Placebo	0	108	2/26/2020	120.3	HEIGHT	20.5	14.6		0	1
1	62	0	Placebo	0	36	3/24/2015	81.5	HEIGHT	11.75	15		0	1
1	62	24	Placebo	0	61	3/28/2017	97.6	HEIGHT	15.35	15.5		0	1
1	62	48	Placebo	0	87	4/21/2019	108.8	HEIGHT	18.95	16		0	1
1	62	60	Placebo	0	96	2/26/2020	115.1	HEIGHT	20.7	14.5		0	1
1	63	12	Placebo	0	8	7/18/2016	71.2	LENGTH	7.15	13		0	1
1	63	36	Placebo	0	29	5/20/2018	88.9	HEIGHT	11.72727273	14		0	1
1	63	60	Placebo	0	50	2/26/2020	102.1	HEIGHT	14.65	13.5		0	1
1	65	12	Placebo	1	9	7/18/2016	71.8	LENGTH	7.35	13	30	1	1
1	68	0	Placebo	1	54	3/24/2015	112.1	HEIGHT	17.2	15		1	1
1	68	36	Placebo	1	109	5/20/2018	128.5	HEIGHT	22.27272727	15.5		0	1
1	68	48	Placebo	1	120	4/21/2019	130.2	LENGTH	23.95	16.5		0	1
1	68	60	Placebo	1	130	2/26/2020	135.7	HEIGHT	26.8	17		0	1
1	69	12	Placebo	1	11	7/18/2016	70.1	LENGTH	7.15	13		1	1
1	69	48	Placebo	1	39	4/21/2019	86.6	HEIGHT	11.65	15		1	1
1	73	12	Placebo	0	51	7/19/2016	118.3	HEIGHT	21	15.5	18	1	1
1	77	24	Placebo	1	41	3/30/2017	104.9	HEIGHT	16.25	15.5		1	1
1	78	12	Placebo	0	7	7/18/2016	68.9	LENGTH	7.55	14		0	1
1	78	36	Placebo	0	32	5/20/2018	84.4	HEIGHT	10.63636364	15		0	1
1	78	60	Placebo	0	52	2/26/2020	98.7	HEIGHT	14.25	16		1	1
1	80	0	Placebo	1	7	3/24/2015	69.5	LENGTH	7.2	13.5		1	1
1	80	12	Placebo	1	22	7/18/2016	85.7	HEIGHT	10.8	14		0	1
1	80	24	Placebo	1	33	3/28/2017	91.4	HEIGHT	12.2	14		0	1
1	80	36	Placebo	1	51	5/20/2018	99.5	HEIGHT	13.81818182	14.5		0	1
1	80	48	Placebo	1	62	4/21/2019	106.9	HEIGHT	15.25	14.5		0	1
1	80	60	Placebo	1	71	2/26/2020	113.3	HEIGHT	17.5	14.4		0	1

1	83	24	Placebo	0	38	3/30/2017	104	HEIGHT	15.9	16		1	1
1	85	0	Placebo	0	48	3/24/2015	99.1	HEIGHT	16.35	15		1	1
1	85	36	Placebo	0	80	5/20/2018	116.2	HEIGHT	20.68181818	16		0	1
1	85	48	Placebo	0	91	4/21/2019	120.4	HEIGHT	23.7	16.5		0	1
1	85	60	Placebo	0	100	2/26/2020	125.2	HEIGHT	26.1	17.8		0	1
1	86	0	Placebo	1	48	3/24/2015	100.2	HEIGHT	14.9	14.5		0	1
1	86	12	Placebo	1	63	7/18/2016	109.3	HEIGHT	16.65	15.5		0	1
1	86	24	Placebo	1	74	3/28/2017	113.4	HEIGHT	17.85	14.5		0	1
1	87	12	Placebo	0	3	7/18/2016	65.9	LENGTH	7.95	15		0	1
1	87	36	Placebo	0	26	5/20/2018	82.4	HEIGHT	11.31818182	15		0	1
1	88	12	Placebo	0	20	7/18/2016	80.9	LENGTH	9.9	15		1	1
1	88	48	Placebo	0	57	4/21/2019	97.5	HEIGHT	14.8	15		1	1
1	89	0	Placebo	0	24	3/24/2015	76.6	LENGTH	8.2	13.5		0	1
1	89	12	Placebo	0	45	7/18/2016	83.9	HEIGHT	10.65	15		0	1
1	89	24	Placebo	0	53	3/28/2017	88.3	HEIGHT	11.85	14		1	1
1	90	24	Placebo	1	65	3/30/2017	111.5	HEIGHT	17.15	15		1	1
1	91	12	Placebo	1	26	7/18/2016	88.6	HEIGHT	11.5	14		1	1
1	91	24	Placebo	1	37	3/30/2017	94.3	HEIGHT	13.25	13.5		1	1
1	92	12	Placebo	0	3	7/18/2016	69.3	HEIGHT	8.1	14		0	1
1	92	36	Placebo	0	29	5/20/2018	89.6	HEIGHT	13	15		0	1
1	92	48	Placebo	0	40	4/21/2019	96.5	HEIGHT	14.4	15		1	1
1	92	60	Placebo	0	50	2/26/2020	103.5	HEIGHT	16.45	14.9		0	1
1	96	12	Placebo	0	57	7/18/2016	100.4	HEIGHT	16.3	15.5		1	1
1	98	24	Placebo	0	2	3/28/2017	59.9	LENGTH	6.55	13.5		1	1
1	98	36	Placebo	0	17	5/20/2018	75.1	LENGTH	9.090909091	14		0	1
1	98	48	Placebo	0	28	4/21/2019	79.7	HEIGHT	10.25	13.5		0	1
1	98	60	Placebo	0	37	2/26/2020	87.6	HEIGHT	13.55	14.8		0	1
1	101	24	Placebo	0	11	3/28/2017	71.3	LENGTH	7.55	12		1	1
1	101	60	Placebo	0	41	2/26/2020	98.2	HEIGHT	13.1	12.7		0	1
1	105	0	Placebo	0	24	3/24/2015	79.4	HEIGHT	11.4	16		0	1
1	107	0	Placebo	0	36	3/24/2015	85.9	HEIGHT	9.85	13	12	0	1
1	108	0	Placebo	1	48	3/24/2015	98.2	HEIGHT	12.8	13.5		0	1
1	108	12	Placebo	1	65	7/18/2016	106.3	HEIGHT	15.4	14.5		0	1
1	108	24	Placebo	1	73	3/30/2017	110.4	HEIGHT	14.55	13		0	1
1	109	0	Placebo	0	42	6/11/2015	91.6	HEIGHT	12.2	13.5		1	1
1	110	0	Placebo	1	24	3/24/2015	88.8	HEIGHT	11.65	14		0	1
1	111	12	Placebo	0	42	7/18/2016	98.8	HEIGHT	14.6	16		1	1
1	112	12	Placebo	0	26	7/18/2016	84.4	HEIGHT	11	14		1	1
1	112	36	Placebo	0	47	5/20/2018	96.1	HEIGHT	14.54545455	15		1	1
1	113	0	Placebo	0	36	3/24/2015	94.5	HEIGHT	14.35	16		0	1

1	113	12	Placebo	0	50	7/18/2016	102.7	HEIGHT	15.6	16		0	1
1	113	36	Placebo	0	84	5/20/2018	111.2	HEIGHT	18.59090909	16		0	1
1	113	48	Placebo	0	95	4/21/2019	115.1	HEIGHT	20.85	16.5		0	1
1	115	0	Placebo	1	24	3/24/2015	84.3	HEIGHT	11.45	15.5	24	0	1
1	115	12	Placebo	1	42	7/18/2016	92.2	HEIGHT	13.55	15.5	24	0	1
1	120	12	Placebo	1	11	7/18/2016	73.9	LENGTH	7.95	13		0	1
1	120	36	Placebo	1	35	5/20/2018	89.9	HEIGHT	10.68181818	13.5		0	1
1	120	48	Placebo	1	46	4/21/2019	96.6	HEIGHT	12.5	14		1	1
1	122	12	Placebo	0	54	7/18/2016	114.6	HEIGHT	19.6	15		1	1
1	123	24	Placebo	1	53	3/28/2017	105.6	HEIGHT	17.25	15.5		1	1
1	124	0	Placebo	0	60	3/24/2015	99.7	HEIGHT	13.5	14	30	0	1
1	126	0	Placebo	0	48	3/24/2015	100.5	HEIGHT	13.4	13.5		0	1
1	127	12	Placebo	0	38	7/19/2016	93.7	HEIGHT	14.65	15		1	1
1	129	12	Placebo	0	32	7/18/2016	83.2	HEIGHT	9.95	14		1	1
1	129	36	Placebo	0	58	5/20/2018	94.8	HEIGHT	13.86363636	16		1	1
1	130	0	Placebo	0	54	3/24/2015	112.4	HEIGHT	18.1	15.5		1	1
1	130	48	Placebo	0	105	4/21/2019	129.3	HEIGHT	27.55	17.5		0	1
1	132	12	Placebo	1	42	7/19/2016	98.9	HEIGHT	15	14.5		1	1
1	133	12	Placebo	1	1	7/18/2016	62.6	LENGTH	6.05	13	36	0	1
1	133	24	Placebo	1	12	3/28/2017	71.3	HEIGHT	7.55	13	36	0	1
1	134	12	Placebo	0	11	7/18/2016	72.4	LENGTH	8.7	14.5		0	1
1	134	24	Placebo	0	20	3/28/2017	77.3	HEIGHT	9.3	13		0	1
1	137	12	Placebo	0	30	7/18/2016	85.8	HEIGHT	10.4	13.5		1	1
1	138	0	Placebo	0	24	3/24/2015	72.2	LENGTH	7.95	13		0	1
1	138	12	Placebo	0	38	7/18/2016	83.1	HEIGHT	11.15	14.5		0	1
1	138	24	Placebo	0	49	3/28/2017	87	HEIGHT	12.4	14		0	1
1	138	48	Placebo	0	75	4/21/2019	100	HEIGHT	14.8	14.5		0	1
1	138	60	Placebo	0	84	2/26/2020	106.6	HEIGHT	16.4	14		0	1
1	140	0	Placebo	0	24	6/11/2015	84.4	LENGTH	10.35	14		0	1
1	140	36	Placebo	0	58	5/20/2018	107.6	HEIGHT	14.68181818	13.5		0	1
1	141	12	Placebo	0	53	7/18/2016	94	HEIGHT	15.95	15.5		1	1
1	142	12	Placebo	1	15	7/18/2016	68.9	HEIGHT	7.1	13	30	0	1
1	144	12	Placebo	0	9	7/18/2016	78.3	LENGTH	11.15	16		0	1
1	144	24	Placebo	0	14	3/30/2017	83	LENGTH	12.4	15		0	1
1	144	60	Placebo	0	54	2/26/2020	107.7	HEIGHT	18.85	15.5		0	1
1	145	0	Placebo	1	18	3/24/2015	80.5	LENGTH	10.05	14		0	1
1	145	12	Placebo	1	33	7/18/2016	90.2	HEIGHT	12.05	14.5		0	1
1	145	24	Placebo	1	44	3/28/2017	95.6	HEIGHT	13.05	14		0	1
1	145	48	Placebo	1	70	4/21/2019	108.9	HEIGHT	19.2	16.5		0	1
1	145	60	Placebo	1	80	2/26/2020	114.5	HEIGHT	18.6	14.5		0	1

1	146	0	Placebo	0	36	3/24/2015	77.5	HEIGHT	8.85	12.5	0	1
1	146	12	Placebo	0	54	7/18/2016	85.1	HEIGHT	11.65	13	0	1
1	147	0	Placebo	1	5	3/24/2015	67.8	LENGTH	6.75	13	0	1
1	147	12	Placebo	1	20	7/18/2016	81.2	HEIGHT	8.95	12.5	0	1
1	147	36	Placebo	1	45	5/20/2018	95.3	HEIGHT	12.22727273	13	0	1
1	147	48	Placebo	1	55	4/21/2019	101.4	HEIGHT	13.85	14	1	1
1	148	12	Placebo	0	14	7/18/2016	83.9	LENGTH	10.95	15.5	1	1
1	148	48	Placebo	0	51	4/21/2019	102.2	HEIGHT	16.9	15.5	1	1
1	149	0	Placebo	0	48	6/11/2015	98.7	HEIGHT	14.5	14.5	0	1
1	149	12	Placebo	0	54	7/18/2016	106.1	HEIGHT	16.05	15	0	1
1	149	36	Placebo	0	88	5/20/2018	117.7	HEIGHT	20.40909091	15	0	1
1	150	0	Placebo	1	54	3/24/2015	113.8	HEIGHT	17.65	14	1	1
1	150	12	Placebo	1	68	7/18/2016	120.4	HEIGHT	19.5	15	0	1
1	150	36	Placebo	1	94	5/20/2018	128.3	HEIGHT	22	15.5	0	1
1	150	60	Placebo	1	114	2/26/2020	135.5	HEIGHT	26.4	16.5	0	1
1	151	12	Placebo	1	10	7/18/2016	72.2	LENGTH	8.15	12.5	0	1
1	151	24	Placebo	1	22	3/28/2017	79.6	HEIGHT	9.35	13.5	0	1
1	154	24	Placebo	1	8	3/28/2017	67.5	LENGTH	6.7	12.5	1	1
1	154	48	Placebo	1	32	4/21/2019	83.4	HEIGHT	10.55	14	0	1
1	155	12	Placebo	0	54	7/18/2016	129.9	HEIGHT	25.45	16.5	1	1
1	157	24	Placebo	0	3	3/28/2017	63.5	LENGTH	7.85	15	1	1
1	157	36	Placebo	0	17	5/20/2018	79.1	LENGTH	9.727272727	15	0	1
1	157	48	Placebo	0	28	4/21/2019	85.6	LENGTH	12.7	15	0	1
1	157	60	Placebo	0	38	2/26/2020	93.9	HEIGHT	13.8	15	1	1
1	159	0	Placebo	0	12	3/24/2015	76.1	LENGTH	8.5	14	1	1
1	159	12	Placebo	0	29	7/18/2016	86.7	HEIGHT	12.55	15.5	1	1
1	159	24	Placebo	0	37	3/30/2017	93.2	HEIGHT	12.75	14.5	1	1
1	159	36	Placebo	0	52	5/20/2018	98.3	HEIGHT	16.09090909	16	0	1
1	160	0	Placebo	0	36	3/24/2015	100.4	HEIGHT	14.6	14.5	1	1
1	160	12	Placebo	0	50	7/18/2016	105.9	HEIGHT	15.65	14	0	1
1	160	24	Placebo	0	61	3/28/2017	110	HEIGHT	17.3	14.5	0	1
1	161	0	Placebo	1	36	3/24/2015	94	HEIGHT	15.45	17	0	1
1	161	12	Placebo	1	50	7/18/2016	104.3	HEIGHT	17.1	16	0	1
1	161	24	Placebo	1	61	3/28/2017	108.5	HEIGHT	18.95	16.5	0	1
1	161	48	Placebo	1	87	4/21/2019	118.7	HEIGHT	21.9	16.5	0	1
1	163	24	Placebo	0	1	3/30/2017	61.4	LENGTH	5.85	13.5	1	1
1	163	36	Placebo	0	15	5/20/2018	77.7	HEIGHT	9.363636364	14	0	1
1	163	48	Placebo	0	26	4/21/2019	86.4	HEIGHT	11.85	14.5	0	1
1	163	60	Placebo	0	35	2/26/2020	94.2	HEIGHT	13.55	14.8	0	1
1	164	24	Placebo	1	54	3/28/2017	101.2	HEIGHT	14.9	14.5	1	1

1	165	12	Placebo	0	9	7/18/2016	73.4	LENGTH	9.9	15		0	1
1	165	36	Placebo	0	29	5/20/2018	89.1	HEIGHT	12.36363636	15		0	1
1	165	60	Placebo	0	50	2/26/2020	101.6	HEIGHT	15.6	14.5		0	1
1	167	0	Placebo	1	24	3/24/2015	84.6	HEIGHT	10.3	13.5		1	1
1	167	12	Placebo	1	38	7/18/2016	87.5	HEIGHT	11	14		0	1
1	167	24	Placebo	1	49	3/28/2017	93	HEIGHT	11.9	14		0	1
1	168	12	Placebo	1	54	7/18/2016	98.7	HEIGHT	14.1	14.5		1	1
1	170	0	Placebo	0	24	3/24/2015	83	LENGTH	9.7	14		0	1
1	170	12	Placebo	0	38	7/18/2016	91.8	LENGTH	12.6	14.7		0	1
1	170	24	Placebo	0	49	3/28/2017	98.3	HEIGHT	14.3	14.5		0	1
1	170	36	Placebo	0	64	5/20/2018	105.5	HEIGHT	15.5	14		0	1
1	170	48	Placebo	0	75	4/21/2019	110.6	HEIGHT	17.5	14.5		0	1
1	172	0	Placebo	0	36	3/24/2015	81.2	HEIGHT	9.05	12		0	1
1	172	12	Placebo	0	54	7/18/2016	90.7	HEIGHT	12.15	14.5		0	1
1	172	24	Placebo	0	65	3/28/2017	99.2	HEIGHT	14.25	14		0	1
1	172	36	Placebo	0	80	5/20/2018	106.7	HEIGHT	15.77272727	15		0	1
1	172	60	Placebo	0	100	2/26/2020	121.4	HEIGHT	20.25	15.2		0	1
1	175	0	Placebo	1	54	3/24/2015	88.9	HEIGHT	12.85	14.5		1	1
1	175	12	Placebo	1	68	7/18/2016	101.8	HEIGHT	16	16		0	1
1	175	24	Placebo	1	79	3/28/2017	105.5	HEIGHT	16.15	15		0	1
1	175	36	Placebo	1	94	5/20/2018	111.2	HEIGHT	18.45454545	16.5		0	1
1	175	48	Placebo	1	105	4/21/2019	116.2	HEIGHT	20.35	16		0	1
1	175	60	Placebo	1	114	2/26/2020	119.5	HEIGHT	21.55	16.3		0	1
1	177	24	Placebo	1	10	3/30/2017	70.8	HEIGHT	7.05	12		1	1
1	177	36	Placebo	1	23	5/20/2018	79.6	HEIGHT	9.363636364	13		1	1
1	177	60	Placebo	1	44	2/26/2020	90.6	HEIGHT	12.05	14.8		0	1
1	180	12	Placebo	0	10	7/18/2016	73.3	LENGTH	8.5	14		0	1
1	180	36	Placebo	0	35	5/20/2018	87.7	HEIGHT	13.09090909	15		0	1
1	182	0	Placebo	0	48	3/24/2015	95.4	HEIGHT	13.8	17		0	1
1	182	12	Placebo	0	54	7/18/2016	103.8	HEIGHT	14.85	15		0	1
1	183	24	Placebo	0	31	3/28/2017	85.4	HEIGHT	11.55	15		1	1
1	183	48	Placebo	0	57	4/21/2019	97.6	HEIGHT	14.25	15.5		1	1
1	183	60	Placebo	0	56	2/26/2020	104.4	HEIGHT	16.1	14.6		1	1
1	185	24	Placebo	0	41	3/30/2017	91.7	HEIGHT	13.55	15		1	1
1	186	12	Placebo	0	5	7/18/2016	66.5	LENGTH	7.5	14.5		0	1
1	186	24	Placebo	0	14	3/28/2017	72.4	LENGTH	8.95	14		0	1
1	186	36	Placebo	0	29	5/20/2018	84	HEIGHT	10.63636364	14		1	1
1	186	60	Placebo	0	50	2/26/2020	97.9	HEIGHT	14.85	14.1		0	1
1	188	12	Placebo	0	6	7/18/2016	67.6	LENGTH	6.5	12.5		0	1
1	188	36	Placebo	0	26	5/20/2018	84.8	LENGTH	9.75	12		0	1

1	189	12	Placebo	1	9	7/18/2016	72	LENGTH	7.8	13.5	0	1
1	189	24	Placebo	1	14	3/28/2017	81	HEIGHT	9.9	13.5	0	1
1	189	36	Placebo	1	29	5/20/2018	88.2	HEIGHT	11.22727273	14	0	1
1	189	60	Placebo	1	50	2/26/2020	101.6	HEIGHT	14.6	14	0	1
1	190	12	Placebo	0	53	7/18/2016	103.4	HEIGHT	17.45	15	1	1
1	191	12	Placebo	1	1	7/18/2016	62.2	LENGTH	6.1	13.5	1	1
1	191	24	Placebo	1	12	3/28/2017	72.1	HEIGHT	7.95	13.5	0	1
1	191	36	Placebo	1	39	5/20/2018	80.7	HEIGHT	9.272727273	12.5	0	1
1	191	48	Placebo	1	50	4/21/2019	86.7	HEIGHT	11.85	15	0	1
1	191	60	Placebo	1	59	2/26/2020	95.1	HEIGHT	13.95	15.5	0	1
1	193	12	Placebo	1	56	7/18/2016	99.6	HEIGHT	13.95	14	1	1
1	197	12	Placebo	0	9	7/18/2016	69.8	LENGTH	7.55	14	0	1
1	197	24	Placebo	0	14	3/28/2017	80	HEIGHT	10.6	15	0	1
1	198	24	Placebo	0	29	3/30/2017	84.9	HEIGHT	13.25	16.5	1	1
1	204	24	Placebo	0	53	3/30/2017	114	HEIGHT	21.9	18	1	1
1	206	12	Placebo	0	6	7/18/2016	64.8	LENGTH	6.3	12	0	1
1	206	24	Placebo	0	12	3/30/2017	69.6	LENGTH	8	12.5	1	1
1	206	60	Placebo	0	49	2/26/2020	89.3	HEIGHT	12.5	13.8	0	1
1	207	0	Placebo	0	12	3/24/2015	81	HEIGHT	10.85	14.5	1	1
1	207	12	Placebo	0	26	7/18/2016	91.5	HEIGHT	13.25	16	0	1
1	207	24	Placebo	0	37	3/28/2017	96.2	HEIGHT	15.35	15	0	1
1	207	48	Placebo	0	63	4/21/2019	108.8	HEIGHT	18.95	15	0	1
1	207	60	Placebo	0	72	2/26/2020	114.2	HEIGHT	20.15	15.3	0	1
1	211	24	Placebo	1	31	3/30/2017	83.2	HEIGHT	10.7	14.5	1	1
1	214	0	Placebo	0	12	3/24/2015	71.6	LENGTH	8	13	0	1
1	214	60	Placebo	0	75	2/26/2020	103.2	HEIGHT	15.7	13.9	0	1
1	215	12	Placebo	1	7	7/18/2016	76.5	HEIGHT	8.15	13.5	0	1
1	215	24	Placebo	1	14	3/28/2017	81.7	HEIGHT	10.25	14	0	1
1	215	36	Placebo	1	29	5/20/2018	91.4	HEIGHT	13.22727273	14.5	0	1
1	216	0	Placebo	1	48	3/24/2015	98	HEIGHT	13.6	13.5	1	1
1	216	12	Placebo	1	66	7/18/2016	105.6	HEIGHT	15.5	14	0	1
1	216	36	Placebo	1	92	5/20/2018	114.9	HEIGHT	17.54545455	14	0	1
1	216	48	Placebo	1	103	4/21/2019	119	HEIGHT	19.9	14.5	0	1
1	216	60	Placebo	1	112	2/26/2020	123.1	HEIGHT	21	15	0	1
1	218	0	Placebo	0	24	3/24/2015	81.3	HEIGHT	10.05	13.5	0	1
1	218	24	Placebo	0	49	3/28/2017	99.3	HEIGHT	16.65	15	0	1
1	218	36	Placebo	0	64	5/20/2018	106.8	HEIGHT	18.09090909	15.5	0	1
1	219	12	Placebo	0	18	7/19/2016	84.7	HEIGHT	10.4	13	1	1
1	221	0	Placebo	1	18	3/24/2015	72.7	LENGTH	7.75	13	1	1
1	223	0	Placebo	1	48	6/11/2015	111.8	HEIGHT	17.45	14	0	1

1	223	12	Placebo	1	65	7/18/2016	118.9	HEIGHT	19.45	15	0	1
1	223	24	Placebo	1	73	3/28/2017	121.6	HEIGHT	20.75	15	0	1
1	223	36	Placebo	1	88	5/20/2018	126.4	HEIGHT	22.5	15.5	0	1
1	223	48	Placebo	1	99	4/21/2019	129	HEIGHT	24.25	16	0	1
1	224	12	Placebo	1	12	7/18/2016	73.3	LENGTH	8.15	13	1	1
1	225	12	Placebo	0	58	7/18/2016	102.2	HEIGHT	14.65	15	1	1
1	226	12	Placebo	1	11	7/18/2016	78.3	LENGTH	9.1	14.5	0	1
1	226	24	Placebo	1	1	3/28/2017	85.3	HEIGHT	10.3	14	0	1
1	226	36	Placebo	1	39	5/20/2018	93.3	HEIGHT	11.09090909	13.5	0	1
1	226	60	Placebo	1	59	2/26/2020	105	HEIGHT	14	14	1	1
1	230	0	Placebo	0	36	3/24/2015	93	HEIGHT	12.95	14.5	0	1
1	230	12	Placebo	0	50	7/18/2016	98.5	HEIGHT	14.5	14.5	0	1
1	232	0	Placebo	0	54	6/11/2015	117.8	HEIGHT	18.45	14.5	0	1
1	235	0	Placebo	0	24	3/24/2015	84.9	HEIGHT	11.85	16.5	0	1
1	236	0	Placebo	0	36	3/24/2015	93.5	HEIGHT	14.55	15	1	1
1	236	24	Placebo	0	63	3/28/2017	107	HEIGHT	18.8	16	0	1
1	236	48	Placebo	0	88	4/21/2019	115.6	HEIGHT	22.45	15.5	0	1
1	236	60	Placebo	0	98	2/26/2020	120.9	HEIGHT	24.35	16	0	1
1	237	0	Placebo	1	24	3/24/2015	78.5	HEIGHT	8.85	13	1	1
1	237	24	Placebo	1	53	3/28/2017	94.7	HEIGHT	13.65	14	0	1
1	237	36	Placebo	1	68	5/20/2018	101.5	HEIGHT	13.86363636	13	0	1
1	239	0	Placebo	0	30	3/24/2015	92	HEIGHT	11.55	14	0	1
1	239	24	Placebo	0	55	3/30/2017	106	HEIGHT	15.25	14.5	1	1
1	239	36	Placebo	0	70	5/20/2018	112.9	HEIGHT	17.18181818	15	0	1
1	240	12	Placebo	0	3	7/18/2016	96.4	HEIGHT	13.65	14.5	0	1
1	240	36	Placebo	0	27	5/20/2018	85.5	HEIGHT	11.95454545	15	0	1
1	240	60	Placebo	0	48	2/26/2020	96.2	HEIGHT	16	15.6	0	1
1	241	24	Placebo	1	31	3/28/2017	79.9	HEIGHT	10.6	14	1	1
1	241	48	Placebo	1	57	4/21/2019	93.7	HEIGHT	13.55	15	1	1
1	243	0	Placebo	0	54	3/24/2015	106	HEIGHT	17	15.5	1	1
1	243	48	Placebo	0	105	4/21/2019	125.2	HEIGHT	22.75	16.5	0	1
1	244	12	Placebo	1	50	7/18/2016	112.6	HEIGHT	15.45	13.5	1	1
1	247	0	Placebo	1	48	6/11/2015	91.9	HEIGHT	13.3	15	0	1
1	249	12	Placebo	1	4	7/18/2016	65.8	LENGTH	8.2	15.5	0	1
1	253	0	Placebo	1	12	3/24/2015	78.2	HEIGHT	10.05	16	0	1
1	253	12	Placebo	1	26	7/18/2016	87.6	HEIGHT	12.1	15.5	0	1
1	253	24	Placebo	1	37	3/28/2017	92.6	HEIGHT	13.25	14.5	0	1
1	253	36	Placebo	1	52	5/20/2018	99.6	HEIGHT	14.72727273	15.5	0	1
1	253	48	Placebo	1	63	4/21/2019	104.6	HEIGHT	16.25	15.5	0	1
1	253	60	Placebo	1	72	2/26/2020	109.5	HEIGHT	17.45	14.3	0	1

1	254	12	Placebo	1	6	7/18/2016	65.1	LENGTH	7.3	15		0	1
1	254	24	Placebo	1	14	3/28/2017	71.8	LENGTH	8.5	15		0	1
1	256	12	Placebo	1	38	7/18/2016	95.5	HEIGHT	14.1	15.5		1	1
1	257	12	Placebo	0	18	7/18/2016	88.7	HEIGHT	12.2	14.5		1	1
1	257	24	Placebo	0	34	3/28/2017	93.4	HEIGHT	14.4	15		1	1
1	258	0	Placebo	1	8	3/24/2015	72.7	LENGTH	9.2	16		1	1
1	258	12	Placebo	1	27	7/18/2016	85.6	HEIGHT	11.85	15		0	1
1	258	36	Placebo	1	50	5/20/2018	98.7	HEIGHT	15.77272727	16.5		0	1
1	259	0	Placebo	0	9	3/24/2015	71.4	LENGTH	8.4	14		1	1
1	259	12	Placebo	0	24	7/18/2016	81.6	LENGTH	10.2	14.5		0	1
1	259	36	Placebo	0	49	5/20/2018	92.2	HEIGHT	12.5	13.5		0	1
1	259	48	Placebo	0	60	4/21/2019	97.4	HEIGHT	13.3	13.5		0	1
1	263	0	Placebo	1	54	3/24/2015	113.3	HEIGHT	18.35	14.5		1	1
1	263	12	Placebo	1	68	7/18/2016	119.2	HEIGHT	20.35	15.5		0	1
1	263	48	Placebo	1	105	4/21/2019	131.9	HEIGHT	26.6	16.5		0	1
1	264	24	Placebo	1	43	3/30/2017	93.2	HEIGHT	14.4	15.5		1	1
1	265	24	Placebo	0	39	3/30/2017	90	HEIGHT	14.25	17		1	1
1	267	12	Placebo	1	2	7/18/2016	62	LENGTH	6.6	14.5		0	1
1	267	24	Placebo	1	12	3/28/2017	70.1	LENGTH	8.35	15		0	1
1	267	36	Placebo	1	26	5/20/2018	80	HEIGHT	10.45454545	15		0	1
1	267	60	Placebo	1	47	2/26/2020	92.4	HEIGHT	13	14.8		0	1
1	269	0	Placebo	0	24	3/24/2015	91.9	HEIGHT	11.8	14		0	1
1	269	12	Placebo	0	41	7/18/2016	100.2	HEIGHT	13.7	14		0	1
1	269	24	Placebo	0	49	3/28/2017	105.3	HEIGHT	15	13		0	1
1	270	0	Placebo	0	24	3/24/2015	86.6	HEIGHT	12.4	15.5	6	1	1
1	271	0	Placebo	0	18	3/24/2015	80.6	LENGTH	10.35	14.5		1	1
1	271	12	Placebo	0	42	7/18/2016	91	HEIGHT	14.25	16.5		1	1
1	271	24	Placebo	0	53	3/28/2017	96.9	HEIGHT	15.55	15.5		1	1
1	271	36	Placebo	0	68	5/20/2018	102.6	HEIGHT	17.31818182	15.5		0	1
1	272	0	Placebo	1	54	3/24/2015	112.6	HEIGHT	17.85	16.5		1	1
1	272	48	Placebo	1	127	4/21/2019	134.4	HEIGHT	28.1	17.5		0	1
1	272	60	Placebo	1	137	2/26/2020	138.6	HEIGHT	29.65	17.8		0	1
1	275	0	Placebo	0	8	3/24/2015	70.2	LENGTH	7.85	14.5		1	1
1	275	12	Placebo	0	21	7/18/2016	81.7	LENGTH	10.15	14.5		0	1
1	275	24	Placebo	0	29	3/28/2017	84.5	HEIGHT	11.3	14.5		0	1
1	275	36	Placebo	0	44	5/20/2018	93.4	HEIGHT	13.95454545	16		1	1
1	275	48	Placebo	0	59	4/21/2019	98.6	HEIGHT	14.55	15.5		0	1
1	277	12	Placebo	0	4	7/18/2016	70.1	LENGTH	6.95	13		0	1
1	277	24	Placebo	0	14	3/28/2017	73.2	LENGTH	7.9	13		1	1
1	277	60	Placebo	0	49	2/26/2020	102	HEIGHT	15.1	14		0	1

1	280	12	Placebo	0	6	7/18/2016	68	LENGTH	7.05	13.5	0	1
1	281	12	Placebo	1	8	7/18/2016	68.9	LENGTH	7.6	13.5	0	1
1	281	24	Placebo	1	13	3/28/2017	74.9	HEIGHT	7.9	12.5	0	1
1	281	36	Placebo	1	28	5/20/2018	80.6	HEIGHT	9.590909091	13	1	1
1	282	0	Placebo	1	36	3/24/2015	95.7	HEIGHT	12.7	14	1	1
1	282	12	Placebo	1	54	7/18/2016	103.7	HEIGHT	14.45	14	0	1
1	282	36	Placebo	1	87	5/20/2018	113.7	HEIGHT	16.86363636	14.5	0	1
1	284	0	Placebo	0	12	3/24/2015	69.4	LENGTH	7.7	13	1	1
1	284	12	Placebo	0	26	7/18/2016	81.4	HEIGHT	11.35	15	0	1
1	284	24	Placebo	0	37	3/28/2017	85.6	HEIGHT	11.95	14.5	0	1
1	284	36	Placebo	0	56	5/20/2018	95.5	HEIGHT	14.59090909	15.5	0	1
1	284	60	Placebo	0	76	2/26/2020	106.5	HEIGHT	17.35	15	0	1
1	286	12	Placebo	1	30	7/18/2016	88.3	LENGTH	12.45	15.5	1	1
1	286	36	Placebo	1	54	5/20/2018	103.4	HEIGHT	18.45454545	16.5	1	1
1	288	0	Placebo	0	36	3/24/2015	85.8	HEIGHT	10.6	16	0	1
1	288	12	Placebo	0	50	7/18/2016	95.7	HEIGHT	12.95	15	0	1
1	288	24	Placebo	0	61	3/28/2017	100.8	HEIGHT	14.35	15	0	1
1	288	36	Placebo	0	76	5/20/2018	108	HEIGHT	15.31818182	15	0	1
1	288	48	Placebo	0	87	4/21/2019	112.4	HEIGHT	17.35	16	0	1
1	289	0	Placebo	1	8	3/24/2015	72.2	LENGTH	8.85	16	1	1
1	289	12	Placebo	1	18	7/18/2016	82.1	HEIGHT	10.25	14.5	0	1
1	289	24	Placebo	1	29	3/28/2017	85.3	HEIGHT	11.7	14.5	0	1
1	289	36	Placebo	1	44	5/20/2018	91.8	HEIGHT	13.09090909	16.5	1	1
1	289	48	Placebo	1	55	4/21/2019	100.7	HEIGHT	15	16	0	1
1	289	60	Placebo	1	64	2/26/2020	107	HEIGHT	16.85	15.7	0	1
1	291	0	Placebo	0	12	3/24/2015	68.8	LENGTH	7.5	13	0	1
1	291	48	Placebo	0	56	4/21/2019	97.7	HEIGHT	14.5	14.5	0	1
1	293	0	Placebo	1	54	3/24/2015	97.5	HEIGHT	14.55	15.5	1	1
1	293	12	Placebo	1	50	7/18/2016	103.9	HEIGHT	15.35	14.5	0	1
1	293	24	Placebo	1	61	3/28/2017	107.3	HEIGHT	15.95	14.5	0	1
1	294	12	Placebo	1	3	7/18/2016	64.2	LENGTH	6.3	12.5	0	1
1	294	24	Placebo	0	12	3/28/2017	71.9	LENGTH	7.1	11.5	0	1
1	294	60	Placebo	0	48	2/26/2020	90.3	HEIGHT	12.4	13.5	0	1
1	295	0	Placebo	0	48	3/24/2015	95.4	HEIGHT	13.2	14.5	0	1
1	295	12	Placebo	0	54	7/18/2016	103.3	HEIGHT	15.35	14	0	1
1	295	24	Placebo	0	65	3/28/2017	108.3	HEIGHT	17.3	14.5	0	1
1	295	36	Placebo	0	80	5/20/2018	113.5	HEIGHT	18.36363636	15	0	1
1	295	48	Placebo	0	91	4/21/2019	116.5	HEIGHT	20.55	16	0	1
1	298	0	Placebo	0	11	3/24/2015	78	LENGTH	9.55	15	1	1
1	298	12	Placebo	0	27	7/18/2016	88.5	HEIGHT	12.55	15.5	0	1

1	298	24	Placebo	0	38	3/28/2017	95.4	HEIGHT	13.45	15.5		0	1
1	298	36	Placebo	0	63	5/20/2018	104.2	HEIGHT	16.09090909	15		0	1
1	298	48	Placebo	0	74	4/21/2019	110.7	HEIGHT	16.8	15		0	1
1	298	60	Placebo	0	83	2/26/2020	115.5	LENGTH	18.25	15		0	1
1	300	0	Placebo	1	48	3/24/2015	108.2	HEIGHT	16.1	15		0	1
1	300	12	Placebo	1	69	7/18/2016	117.3	HEIGHT	19.25	15		0	1
1	300	24	Placebo	1	77	3/30/2017	122	HEIGHT	21.1	14		0	1
1	300	36	Placebo	1	92	5/20/2018	126.4	HEIGHT	23.54545455	17		0	1
1	300	48	Placebo	1	103	4/21/2019	129.4	HEIGHT	25	16		0	1
1	301	24	Placebo	1	25	3/28/2017	80.8	HEIGHT	10.8	15		1	1
1	302	0	Placebo	1	48	3/24/2015	83	HEIGHT	9.4	12.5		0	1
1	302	24	Placebo	1	61	3/28/2017	97.9	HEIGHT	14.45	14		0	1
1	302	36	Placebo	1	76	5/20/2018	104.7	HEIGHT	16.59090909	14.5		0	1
1	302	48	Placebo	1	87	4/21/2019	110.6	HEIGHT	18.8	15		0	1
1	302	60	Placebo	1	96	2/26/2020	116.3	HEIGHT	20.2	14.5		0	1
1	303	24	Placebo	0	42	3/28/2017	92.1	HEIGHT	13.25	16		1	1
1	305	12	Placebo	0	42	7/18/2016	84.4	HEIGHT	13.7	16		1	1
1	306	0	Placebo	0	48	3/24/2015	96.2	HEIGHT	15.2	16		0	1
1	306	12	Placebo	0	54	7/18/2016	105.9	HEIGHT	17.55	16		0	1
1	306	36	Placebo	0	87	5/20/2018	115.7	HEIGHT	20.63636364	16.5		0	1
1	306	48	Placebo	0	98	4/21/2019	119.4	HEIGHT	22.05	16		0	1
1	306	60	Placebo	0	108	2/26/2020	124.9	HEIGHT	23.05	15.8		0	1
1	307	0	Placebo	1	36	3/24/2015	83.8	HEIGHT	11.55	15.5		0	1
1	307	12	Placebo	1	52	7/18/2016	94.2	HEIGHT	14.7	14.5		0	1
1	307	48	Placebo	1	86	4/21/2019	110.6	HEIGHT	18.9	16.5		0	1
1	310	0	Placebo	1	30	3/24/2015	85.2	HEIGHT	10	12.5		0	1
1	310	24	Placebo	1	38	3/28/2017	98.9	HEIGHT	12.75	13		1	1
1	310	36	Placebo	1	53	5/20/2018	106.8	HEIGHT	14.81818182	13.5		1	1
1	312	24	Placebo	1	37	3/30/2017	90.4	HEIGHT	13.6	15.5		1	1
1	312	36	Placebo	1	52	5/20/2018	97.7	HEIGHT	14.40909091	15		1	1
1	314	12	Placebo	1	38	7/20/2016	94	HEIGHT		13.9		1	1
1	316	24	Placebo	1	37	3/30/2017	93.7	HEIGHT	12.6	14		1	1
1	316	36	Placebo	1	52	5/20/2018	104.5	HEIGHT	14.90909091	14		1	1
1	318	24	Placebo	0	31	3/28/2017	80	HEIGHT	10.6	15	60	1	1
1	319	0	Placebo	1	24	6/11/2015	81	HEIGHT	10.25	14		1	1
1	320	12	Placebo	0	18	7/18/2016	75.9	HEIGHT	9.35	14		1	1
1	322	12	Placebo	1	8	7/18/2016	68.4	LENGTH	6.9	12.5	42	0	1
1	322	24	Placebo	1	15	3/30/2017	73.9	HEIGHT	7.8	12	42	0	1
1	323	0	Placebo	1	24	3/24/2015	85.6	HEIGHT	11.25	13		1	1
1	323	24	Placebo	1	49	3/28/2017	102.3	HEIGHT	15	14		0	1

1	323	36	Placebo	1	64	5/20/2018	110.5	HEIGHT	16.13636364	15	0	1
1	323	48	Placebo	1	75	4/21/2019	115.2	HEIGHT	17.8	15	0	1
1	323	60	Placebo	1	84	2/26/2020	122.2	HEIGHT	19.4	15	0	1
1	324	12	Placebo	0	1	7/18/2016	61.4	LENGTH	6.2	13.5	0	1
1	324	36	Placebo	0	26	5/20/2018	79.7	HEIGHT	9.454545455	14	0	1
1	324	60	Placebo	0	47	2/26/2020	90.1	HEIGHT	11.55	13.8	0	1
1	328	0	Placebo	1	12	3/24/2015	74.9	LENGTH	9.1	14.5	0	1
1	328	12	Placebo	1	26	7/18/2016	86.2	HEIGHT	11.4	15	0	1
1	328	24	Placebo	1	40	3/28/2017	92	HEIGHT	12.65	14.5	0	1
1	328	36	Placebo	1	55	5/20/2018	97.3	HEIGHT	13.22727273	14.5	0	1
1	328	48	Placebo	1	66	4/21/2019	102.1	HEIGHT	14.3	14	0	1
1	328	60	Placebo	1	75	2/26/2020	107.7	HEIGHT	15.55	13.9	0	1
1	331	12	Placebo	1	7	7/18/2016	70.6	LENGTH	7.8	15	0	1
1	331	24	Placebo	1	18	3/30/2017	75.5	HEIGHT	8.4	14	0	1
1	331	36	Placebo	1	32	5/20/2018	80.5	HEIGHT	8.681818182	12	0	1
1	332	24	Placebo	1	41	3/30/2017	101.3	HEIGHT	15.3	15	1	1
1	333	12	Placebo	0	11	7/18/2016	76.2	LENGTH	8.3	13	0	1
1	333	36	Placebo	0	35	5/20/2018	116.4	HEIGHT	18.63636364	16	1	1
1	333	60	Placebo	0	56	2/26/2020	105.3	HEIGHT	17.9	15	0	1
1	335	0	Placebo	0	36	3/24/2015	88.3	HEIGHT	12.3	14	1	1
1	335	48	Placebo	0	87	4/21/2019	114.6	HEIGHT	19.15	15	0	1
1	335	60	Placebo	0	96	2/26/2020	119.5	HEIGHT	20.5	14.1	0	1
1	336	12	Placebo	1	50	7/18/2016	103.3	HEIGHT	16.4	16.5	1	1
1	337	24	Placebo	1	12	3/28/2017	72	LENGTH	7.25	13.5	1	1
1	337	48	Placebo	1	37	4/21/2019	85.1	HEIGHT	9.75	13.5	1	1
1	338	12	Placebo	0	12	7/20/2016	78.3	LENGTH		13.5	1	1
1	338	48	Placebo	0	48	4/21/2019	99.2	HEIGHT	14.95	13.5	0	1
1	340	0	Placebo	0	36	3/24/2015	95.3	HEIGHT	13.7	14.5	0	1
1	340	36	Placebo	0	76	5/20/2018	111.1	HEIGHT	17.77272727	14	0	1
1	340	60	Placebo	0	96	2/26/2020	121	HEIGHT	21.55	15.8	0	1
1	341	0	Placebo	0	54	6/11/2015	103.4	HEIGHT	17.15	15	0	1
1	341	24	Placebo	0	53	3/28/2017	114.2	HEIGHT	21.65	15.5	0	1
1	341	48	Placebo	0	79	4/21/2019	123.8	HEIGHT	25.1	16.5	0	1
1	342	12	Placebo	1	38	7/18/2016	88.7	HEIGHT	12.35	14.5	1	1
1	343	0	Placebo	1	48	3/24/2015	114	HEIGHT	17.6	13.5	1	1
1	344	0	Placebo	1	6	3/24/2015	63.6	LENGTH	6	12.5	1	1
1	346	0	Placebo	0	48	3/24/2015	98.2	HEIGHT	15.5	14	1	1
1	346	12	Placebo	0	62	7/18/2016	105.3	HEIGHT	17.15	15	0	1
1	346	24	Placebo	0	73	3/28/2017	109.6	HEIGHT	19.15	15.5	0	1
1	346	48	Placebo	0	99	4/21/2019	118.8	HEIGHT	23.15	16	0	1

1	346	60	Placebo	0	108	2/26/2020	122.8	HEIGHT	24.85	16.3		0	1
1	348	0	Placebo	1	18	3/24/2015	82.3	HEIGHT	9.55	13.5		0	1
1	348	12	Placebo	1	33	7/18/2016	90.6	HEIGHT	12.35	14.5		0	1
1	348	24	Placebo	1	41	3/28/2017	98.1	HEIGHT	13.65	14.5		0	1
1	348	36	Placebo	1	56	5/20/2018	106.4	HEIGHT	15.27272727	14.5		0	1
1	348	48	Placebo	1	67	4/21/2019	109.1	HEIGHT	16.4	15		0	1
1	348	60	Placebo	1	77	2/26/2020	116.2	HEIGHT	18.65	14.7		0	1
1	352	24	Placebo	0	41	3/28/2017	102.2	HEIGHT	14.55	14.5		1	1
1	353	12	Placebo	0	50	7/18/2016	96.7	HEIGHT	13.35	15.5		1	1
1	355	0	Placebo	1	48	3/24/2015	113	HEIGHT	19.9	17		0	1
1	358	0	Placebo	0	36	3/24/2015	86.1	LENGTH	10.8	12.5		0	1
1	358	12	Placebo	0	50	7/18/2016	97.1	HEIGHT	13.35	14		0	1
1	358	24	Placebo	0	61	3/28/2017	102.2	HEIGHT	14.55	13		0	1
1	360	0	Placebo	1	12	3/24/2015	82.1	HEIGHT	9.4	13.5		1	1
1	361	12	Placebo	0	3	7/18/2016	63.7	LENGTH	8	14.5		0	1
1	8021	36	Placebo	1	44	5/20/2018	93.2	HEIGHT	12.85	15		1	1
1	8063	36	Placebo	0	5	5/20/2018	60.1	LENGTH	6.727272727	14		1	1
1	8063	48	Placebo	0	15	4/21/2019	73	LENGTH	8.7	14		0	1
1	8063	60	Placebo	0	25	2/26/2020	83.3	LENGTH	10.15	13.8		1	1
1	8065	48	Placebo	0	40	4/21/2019	90.1	HEIGHT	12.55	14		1	1
1	8065	60	Placebo	0	50	2/27/2020	97.2	HEIGHT	13.95	13.8		1	1
1	8078	48	Placebo	0	6	4/21/2019	70.4	HEIGHT	8.1	15		1	1
1	8104	36	Placebo	0	22	5/20/2018	83.5	HEIGHT	9.318181818	13.5		1	1
1	8106	48	Placebo	0	18	4/21/2019	72.3	LENGTH	7.5	13		1	1
1	8177	60	Placebo	1	54	2/26/2020	99.1	HEIGHT	15.4	15		1	1
1	8179	48	Placebo	1	15	4/21/2019	75.5	LENGTH	8.35	12.5		1	1
1	8203	36	Placebo	1	39	5/20/2018	87.2	HEIGHT	12	15		1	1
1	8239	36	Placebo	0	6	5/20/2018	65.8	LENGTH	7.636363636	14	54	1	1
1	8325	48	Placebo	1	15	4/21/2019	73.9	LENGTH	7.95	13.5		1	1
1	8327	60	Placebo	1	50	2/27/2020	106.1	HEIGHT	15.05	13.3		1	1
1	8346	48	Placebo	0	17	4/21/2019	73.3	LENGTH	7.75	13		1	1
1	8348	48	Placebo	0	36	4/21/2019	89.6	LENGTH	14.7	17		1	1
1	8348	60	Placebo	0	46	2/26/2020	95.9	HEIGHT	15.6	15.6		1	1
1	8350	48	Placebo	1	21	4/21/2019	77.2	LENGTH	9	14		1	1
1	8354	36	Placebo	1	17	5/20/2018	72.5	LENGTH	7.409090909	12.5		1	1
1	8365	48	Placebo	0	8	4/21/2019	64.9	LENGTH	6	12		1	1
1	8365	60	Placebo	0	14	2/26/2020	74.5	HEIGHT	7.8	12		0	1
1	8393	60	Placebo	1	24	2/26/2020	82.9	LENGTH	9.55	13.5		1	1
1	8397	60	Placebo	0	55	2/27/2020	94.5	HEIGHT	16.45	16		1	1
1	8405	48	Placebo	1	7	4/21/2019	65.3	LENGTH	6.85	12		1	1

1	8405	60	Placebo	1	16	2/26/2020	76.9	LENGTH	9.45	13.5	0	1
1	8440	48	Placebo	0	10	4/21/2019	68.3	LENGTH	7.35	13	1	1
1	8442	48	Placebo	1	58	4/21/2019	90.5	HEIGHT	13	14	1	1
1	8456	36	Placebo	1	48	5/20/2018	97.7	HEIGHT	13.95454545	14.5	1	1
1	8459	60	Placebo	0	32	2/27/2020	87.8	HEIGHT	12.4	14	1	1
1	8471	60	Placebo	0	11	2/27/2020	74.8	LENGTH	10.15	16.5	1	1
1	8473	36	Placebo	0	8	5/20/2018	70.1	LENGTH	7.318181818	12.5	1	1
1	8508	36	Placebo	1	56	5/20/2018	106.1	HEIGHT	17.68181818	15.5	1	1
1	8543	36	Placebo	0	6	5/20/2018	68.7	LENGTH	7.090909091	13	1	1
1	8543	48	Placebo	0	16	4/21/2019	79.3	HEIGHT	8.95	14	0	1
1	8551	36	Placebo	0	53	5/20/2018	79.7	HEIGHT	14.13636364	14.5	1	1
1	8587	60	Placebo	1	10	2/27/2020	67.3	LENGTH	8.4	14.7	1	1
1	8596	36	Placebo	1	12	5/20/2018	72.4	HEIGHT	8.363636364	14.5	1	1
1	8609	48	Placebo	0	50	4/21/2019	92.4	HEIGHT	12.95	14	1	1
1	8628	60	Placebo	0	20	2/26/2020	78.6	LENGTH	9.8	14.5	1	1
1	8631	36	Placebo	1	28	5/20/2018	89	HEIGHT	12.54545455	14.5	1	1
1	8660	36	Placebo	1	12	5/20/2018	72.6	LENGTH	7.545454545	12.5	1	1
1	8678	60	Placebo	0	25	2/27/2020	85.4	HEIGHT	12.4	13.6	1	1
1	8707	60	Placebo	1	10	2/26/2020	69.5	LENGTH	7.25	12.2	1	1
1	8714	48	Placebo	1	2	4/21/2019	54.1	LENGTH	5	13.5	1	1
1	8765	48	Placebo	1	43	4/21/2019	85.9	HEIGHT	12.15	13.5	1	1
1	8782	36	Placebo	0	56	5/20/2018	94.4	HEIGHT	13.36363636	14	1	1
1	8795	36	Placebo	0	19	5/20/2018	82.7	HEIGHT	10.90909091	15	1	1
1	8812	36	Placebo	1	12	5/20/2018	75.7	LENGTH	8.772727273	14	1	1
1	8812	48	Placebo	1	22	4/21/2019	84.3	LENGTH	10.7	14	0	1
1	8812	60	Placebo	1	32	2/26/2020	99.2	HEIGHT	12.45	14.2	0	1
1	8845	60	Placebo	0	58	2/26/2020	105.5	HEIGHT	16.2	15	1	1
1	8866	60	Placebo	0	59	2/26/2020	107.3	HEIGHT	14.65	13.5	1	1
1	8909	36	Placebo	1	42	5/20/2018	91.9	HEIGHT	12.13636364	14	1	1
1	8921	48	Placebo	0	9	4/21/2019	70.5	LENGTH	7.5	13.5	1	1
1	8921	60	Placebo	0	14	2/26/2020	76.9	LENGTH	9.7	13.8	0	1
1	8929	60	Placebo	1	55	2/26/2020	101	HEIGHT	16.25	15.2	1	1
1	8930	60	Placebo	0	34	2/26/2020	84.7	LENGTH	11.6	13.5	1	1
1	8935	60	Placebo	1	45	2/26/2020	95	HEIGHT	12.9	14.5	1	1
1	8939	48	Placebo	0	37	4/21/2019	90.5	HEIGHT	13.4	16.5	1	1
1	8939	60	Placebo	0	47	2/26/2020	98.9	HEIGHT	14.9	15.5	1	1
1	8947	48	Placebo	0	37	4/21/2019	89.6	HEIGHT	11.7	12.5	1	1
1	8968	60	Placebo	1	21	2/27/2020	76	LENGTH	8.65	13.2	1	1
1	9032	48	Placebo	1	21	4/21/2019	75.2	LENGTH	9.25	15	1	1
1	9032	60	Placebo	1	31	2/26/2020	82.2	HEIGHT	11.7	14.5	1	1

1	9054	48	Placebo	0	16	4/21/2019	90	HEIGHT	12.3	13.5		1	1
1	9076	36	Placebo	1	35	5/20/2018	87.1	HEIGHT	12.5	15.5		1	1
1	9078	48	Placebo	1	4	4/21/2019	61.6	LENGTH	6.35	13.5		1	1
1	9139	60	Placebo	0	30	2/26/2020	86.1	HEIGHT	12.8	15.7		1	1
1	9141	36	Placebo	0	21	5/20/2018	83.9	HEIGHT	9.954545455	13.5		1	1
1	9210	36	Placebo	1	15	5/20/2018	73.6	HEIGHT	8.045454545	12.5		1	1
1	9224	36	Placebo	1	27	5/20/2018	81.1	HEIGHT	10	13.5		1	1
1	9234	60	Placebo	1	50	2/27/2020	106.3	HEIGHT	21.35	18		1	1
1	9235	48	Placebo	0	48	4/21/2019	80.6	HEIGHT	8.9	12.5		1	1
1	9251	36	Placebo	1	17	5/20/2018	75	LENGTH	8.590909091	13.5		1	1
1	9261	60	Placebo	0	46	2/27/2020	96.5	HEIGHT	16.15	16.5		1	1
1	9270	60	Placebo	1	7	2/26/2020	64.9	LENGTH	6.55	13.5		1	1
1	9304	60	Placebo	1	14	2/26/2020	93.8	LENGTH	7.9	13.5		1	1
1	9312	48	Placebo	0	36	4/21/2019	92.3	HEIGHT	12.4	14		1	1
1	9317	36	Placebo	0	40	5/20/2018	94.9	HEIGHT	12.95454545	13.5		1	1
1	9325	48	Placebo	1	55	4/21/2019	98.9	HEIGHT	15.2	14.5		1	1
1	9357	48	Placebo	0	19	4/21/2019	78.9	HEIGHT	9.3	14		1	1
1	9357	60	Placebo	0	28	2/26/2020	75.7	HEIGHT	12	14.5		1	1
1	9365	60	Placebo	1	21	2/27/2020	99.7	HEIGHT	9.3	12.2		1	1
1	9373	48	Placebo	1	21	4/21/2019	80.7	HEIGHT	8.75	12		1	1
1	9383	36	Placebo	0	7	5/20/2018	65.4	LENGTH	7	14.5	60	1	1
1	9387	48	Placebo	1	55	4/21/2019	97.8	HEIGHT	12.7	13.5		1	1
1	9399	60	Placebo	1	20	2/27/2020	88.3	HEIGHT	10.85	12.8		1	1
1	9411	36	Placebo	0	56	5/20/2018	105.8	HEIGHT	18.55	17		1	1
1	9425	60	Placebo	1	50	2/26/2020	110.4	HEIGHT	19	15.5		1	1
1	9433	48	Placebo	1	51	4/21/2019	95.3	HEIGHT	12.55	15		1	1
1	9433	60	Placebo	1	54	2/26/2020	101	HEIGHT	14.1	14.2		1	1
1	9436	48	Placebo	1	51	4/21/2019	90.8	LENGTH	12.65	15		1	1
1	9489	48	Placebo	0	22	4/21/2019	79.3	LENGTH	9.75	13.5		1	1
1	9495	48	Placebo	0	15	4/21/2019	75.4	LENGTH	8.15	13		1	1
1	9495	60	Placebo	0	25	2/26/2020	83.1	HEIGHT	10.45	13.4		1	1
1	9500	60	Placebo	0	25	2/27/2020	83.5	LENGTH	9.7	13.5		1	1
1	9502	36	Placebo	1	11	5/20/2018	67.8	LENGTH	7.045454545	12		1	1
1	9502	48	Placebo	1	20	4/21/2019	78	HEIGHT	9.45	13.5		0	1
1	9502	60	Placebo	1	30	2/26/2020	85.9	HEIGHT	11.25	13.8		0	1
1	9512	60	Placebo	1	29	2/27/2020	88	HEIGHT	10.55	13.2		1	1
1	9527	60	Placebo	0	3	2/26/2020	61.6	LENGTH	7.5	13.5		1	1
1	9558	36	Placebo	0	35	5/20/2018	91.3	HEIGHT	12.22727273	14.5		1	1
1	9566	36	Placebo	0	55	5/20/2018	94.9	HEIGHT	13.95454545	15.5		1	1
1	9570	60	Placebo	1	26	2/27/2020	83.1	HEIGHT	10.2	13.2		1	1

2	370	0	Placebo	1	48	4/9/2015	103.2	HEIGHT	14.5	14.5		1	0
2	370	12	Placebo	1	54	6/16/2016	109	HEIGHT	15.9	14.5		1	0
2	371	24	Placebo	0	14	5/15/2017	72.8	HEIGHT	7.7	13		1	0
2	371	36	Placebo	0	27	6/9/2018	85.6	LENGTH	11.75	15		1	0
2	371	48	Placebo	0	37	5/23/2019	91.4	HEIGHT	12.8	14		1	0
2	372	0	Placebo	0	54	4/9/2015	109.2	HEIGHT	16.35	14.5		1	0
2	372	36	Placebo	0	116	6/9/2018	125.2	HEIGHT	21.5	16		0	0
2	372	48	Placebo	0	127	5/23/2019	130.1	HEIGHT	23.8	16		0	0
2	372	60	Placebo	0	136	2/17/2020	133.5	HEIGHT	25.45454545	16.2		0	0
2	374	0	Placebo	1	36	4/9/2015	94.7	HEIGHT	13	14.5	24	1	0
2	374	12	Placebo	1	54	6/16/2016	101.4	HEIGHT	14.4	14.5	24	1	0
2	377	0	Placebo	0	48	4/9/2015	104.6	HEIGHT	15.9	15.5		1	0
2	377	36	Placebo	0	89	6/9/2018	114.1	HEIGHT	19	16		0	0
2	377	48	Placebo	0	100	5/23/2019	118.3	HEIGHT	20.8	16		0	0
2	377	60	Placebo	0	110	2/17/2020	121.2	HEIGHT	22.86363636	16.5		0	0
2	381	12	Placebo	1	9	6/16/2016	75.5	HEIGHT	9.55	15.5		1	0
2	381	24	Placebo	1	17	5/15/2017	86.1	HEIGHT	11.8	15.5		1	0
2	381	36	Placebo	1	34	6/9/2018	93.7	HEIGHT	14.25	16		1	0
2	381	48	Placebo	1	45	5/23/2019	101.6	HEIGHT	16.45	16		1	0
2	382	12	Placebo	1	56	6/16/2016	106.2	HEIGHT	15.55	14.5		1	0
2	382	36	Placebo	1	82	6/9/2018	116.5	HEIGHT	18.35	14.5		0	0
2	382	48	Placebo	1	93	5/23/2019	121.2	HEIGHT	19.75	15		0	0
2	382	60	Placebo	1	102	2/17/2020	124	HEIGHT	21.5	15.5		0	0
2	383	24	Placebo	0	38	5/15/2017	88	HEIGHT	13.1	15		1	0
2	383	36	Placebo	0	50	6/9/2018	97.2	HEIGHT	14.6	14.5		1	0
2	383	60	Placebo	0	70	2/17/2020	107.2	HEIGHT	17.63636364	14.8		0	0
2	385	0	Placebo	1	18	4/9/2015	76.9	HEIGHT	8.35	13		1	0
2	385	12	Placebo	1	32	6/16/2016	83.2	HEIGHT	9.65	13		1	0
2	385	24	Placebo	1	45	5/15/2017	89.5	HEIGHT	10.8	13		1	0
2	385	36	Placebo	1	58	6/9/2018	96.9	HEIGHT	12.1	14		1	0
2	385	48	Placebo	1	69	5/23/2019	102.8	HEIGHT	13.5	13		0	0
2	385	60	Placebo	1	78	2/17/2020	107.2	HEIGHT	15	13.1		0	0
2	387	12	Placebo	1	38	6/16/2016	89.5	HEIGHT	11.75	14.5		1	0
2	387	24	Placebo	1	51	5/15/2017	95.2	HEIGHT	13.05	14		1	0
2	387	36	Placebo	1	64	6/9/2018	102.4	HEIGHT	14.5	14		0	0
2	387	48	Placebo	1	75	5/23/2019	108.4	HEIGHT	16.15	14		0	0
2	387	60	Placebo	1	84	2/17/2020	113.3	HEIGHT	17.40909091	14.4		0	0
2	389	24	Placebo	0	12	5/15/2017	74.7	LENGTH	8.7	13		1	0
2	389	36	Placebo	0	27	6/9/2018	83.9	HEIGHT	11.1	14		1	0
2	389	48	Placebo	0	37	5/23/2019	92.3	HEIGHT	13.6	14		1	0

2	389	60	Placebo	0	47	2/17/2020	99.6	HEIGHT	14.63636364	14.5		1	0
2	394	0	Placebo	0	9	4/9/2015	69.2	LENGTH	7.55	13.5		1	0
2	394	24	Placebo	0	35	5/15/2017	81.9	HEIGHT	11.15	14		1	0
2	394	36	Placebo	0	47	6/9/2018	86.3	HEIGHT	11.15	13.5		1	0
2	394	48	Placebo	0	58	5/23/2019	92.9	LENGTH	12.9	14		1	0
2	399	0	Placebo	0	36	4/9/2015	89.7	HEIGHT	13.1	14		1	0
2	399	12	Placebo	0	54	6/16/2016	97.5	HEIGHT	15.25	15		1	0
2	399	36	Placebo	0	80	6/9/2018	112.7	HEIGHT	18.25	15.5		0	0
2	399	48	Placebo	0	91	5/23/2019	120.2	HEIGHT	19.75	14.5		0	0
2	400	12	Placebo	0	11	6/16/2016	71.8	HEIGHT	7.8	13		1	0
2	400	24	Placebo	0	23	5/15/2017	79.4	HEIGHT	9.75	13.5		1	0
2	400	36	Placebo	0	35	6/9/2018	86.4	HEIGHT	11.3	13.5		1	0
2	400	48	Placebo	0	46	5/23/2019	94	HEIGHT	11.95	13		1	0
2	400	60	Placebo	0	56	2/17/2020	98.5	HEIGHT	13	13.4		1	0
2	402	0	Placebo	1	18	4/9/2015	80.5	HEIGHT	9.5	14.5		1	0
2	402	12	Placebo	1	30	6/16/2016	88.4	HEIGHT	11.95	15		1	0
2	402	24	Placebo	1	49	5/15/2017	95.8	HEIGHT	13.65	15.5		1	0
2	403	0	Placebo	1	36	4/9/2015	88.7	HEIGHT	11.6	14.5		1	0
2	403	12	Placebo	1	50	6/16/2016	97.3	HEIGHT	12.9	15		1	0
2	403	24	Placebo	1	54	5/15/2017	103.6	HEIGHT	14.85	15		1	0
2	403	36	Placebo	1	67	6/9/2018	108.7	HEIGHT	16.2	15		0	0
2	403	48	Placebo	1	78	5/23/2019	116.8	HEIGHT	18.55	15.5		0	0
2	403	60	Placebo	1	87	2/17/2020	121	HEIGHT	20.86363636	15.5		0	0
2	406	0	Placebo	0	36	4/9/2015	95.9	HEIGHT	14.75	15		1	0
2	406	12	Placebo	0	42	6/16/2016	102.2	HEIGHT	17.15	16		1	0
2	406	24	Placebo	0	56	5/15/2017	109.3	HEIGHT	18.25	16		1	0
2	410	0	Placebo	0	12	4/9/2015	76.8	LENGTH	9.3	12.5		1	0
2	410	24	Placebo	0	39	5/15/2017	90	HEIGHT	12.65	13.5		1	0
2	410	36	Placebo	0	51	6/9/2018	99.2	HEIGHT	14.9	14.5		1	0
2	410	48	Placebo	0	62	5/23/2019	107.6	HEIGHT	17.25	14		0	0
2	411	24	Placebo	1	56	5/15/2017	91.7	HEIGHT	12.3	14.5		1	0
2	413	12	Placebo	1	56	6/16/2016	92.2	HEIGHT	11.65	13.5		1	0
2	413	36	Placebo	1	82	6/9/2018	108.4	HEIGHT	14.85	14		0	0
2	413	48	Placebo	1	93	5/23/2019	114.3	HEIGHT	16.25	14.5		0	0
2	413	60	Placebo	1	102	2/17/2020	118.7	HEIGHT	17.86363636	15		0	0
2	414	12	Placebo	1	54	6/16/2016	119.5	HEIGHT	20.65	15.5	30	1	0
2	416	0	Placebo	1	48	4/9/2015	91.5	HEIGHT	11	13.5		1	0
2	416	12	Placebo	1	62	6/16/2016	98	HEIGHT	12.95	14		0	0
2	416	36	Placebo	1	88	6/9/2018	110.4	HEIGHT	16.5	14.5		0	0
2	416	48	Placebo	1	99	5/23/2019	114.9	HEIGHT	18.3	14.5		0	0

2	416	60	Placebo	1	108	2/17/2020	117.9	HEIGHT	20.22727273	15.5		0	0
2	418	0	Placebo	1	48	4/9/2015	105	HEIGHT	13.5	13.5		1	0
2	418	12	Placebo	0	54	6/16/2016	109.5	HEIGHT	15.15	14		1	0
2	418	36	Placebo	0	80	6/9/2018	120	HEIGHT	17.85	15		0	0
2	418	48	Placebo	0	91	5/23/2019	122.7	HEIGHT	19.8	15		0	0
2	421	24	Placebo	0	9	5/15/2017	69.3	LENGTH	6.9	12.5	30	1	0
2	422	24	Placebo	1	5	5/15/2017	58.4	LENGTH	5.1	12.5		1	0
2	422	36	Placebo	1	15	6/9/2018	88.3	LENGTH	6.55	13		1	0
2	422	48	Placebo	1	26	5/23/2019	78.5	LENGTH	8.5	12.5		1	0
2	423	0	Placebo	1	48	4/9/2015	107.1	HEIGHT	16.3	15		1	0
2	423	36	Placebo	1	92	6/9/2018	125.6	HEIGHT	23.1	16.5		0	0
2	423	48	Placebo	1	103	5/23/2019	131.2	HEIGHT	25.35	16.5		0	0
2	423	60	Placebo	1	112	2/17/2020	137.4	HEIGHT	28.68181818	18		0	0
2	425	24	Placebo	0	24	5/15/2017	77.7	HEIGHT	9.6	13		1	0
2	425	48	Placebo	0	47	5/23/2019	92.5	HEIGHT	13.95	15		1	0
2	425	60	Placebo	0	57	2/17/2020	100.4	HEIGHT	15.36363636	14.8		1	0
2	426	0	Placebo	0	36	4/9/2015	97.1	HEIGHT	14.3	14	60	1	0
2	426	12	Placebo	0	54	6/16/2016	104.6	HEIGHT	16.65	15.5	60	1	0
2	429	12	Placebo	0	10	6/16/2016	75.2	LENGTH	9.4	14		1	0
2	429	24	Placebo	0	23	5/15/2017	85.3	HEIGHT	11.85	15		1	0
2	429	36	Placebo	0	35	6/9/2018	94	HEIGHT	14.6	15.5		1	0
2	429	48	Placebo	0	45	5/23/2019	102.3	HEIGHT	15.85	15		1	0
2	429	60	Placebo	0	55	2/17/2020	107.2	HEIGHT	18.18181818	15.5		1	0
2	431	0	Placebo	1	24	4/9/2015	81.2	HEIGHT	9.75	13.5		1	0
2	431	12	Placebo	1	39	6/16/2016	89	HEIGHT	12.05	15		1	0
2	431	24	Placebo	1	53	5/15/2017	95.6	HEIGHT	13.05	14.5		1	0
2	433	0	Placebo	1	24	4/9/2015	82.5	HEIGHT	10.25	13.5		1	0
2	433	36	Placebo	1	64	6/9/2018	108.3	HEIGHT	16.9	15		0	0
2	433	48	Placebo	1	75	5/23/2019	115.3	HEIGHT	19.1	15		0	0
2	433	60	Placebo	1	84	2/17/2020	121.2	HEIGHT	22.09090909	16		0	0
2	434	24	Placebo	0	12	5/15/2017	72.1	LENGTH	7.3	12	54	1	0
2	434	36	Placebo	0	24	6/9/2018	82.4	HEIGHT	10.05	13.5	54	1	0
2	434	48	Placebo	0	35	5/23/2019	89.7	HEIGHT	11.75	13.5	54	1	0
2	436	12	Placebo	0	2	6/16/2016	63.3	LENGTH	7.5	14		1	0
2	436	24	Placebo	0	16	5/15/2017	75.5	HEIGHT	9.1	14		1	0
2	436	36	Placebo	0	29	6/9/2018	82.8	HEIGHT	11.8	15.5		1	0
2	436	48	Placebo	0	39	5/23/2019	90.6	HEIGHT	12.8	14.5		1	0
2	436	60	Placebo	0	49	2/17/2020	96.7	HEIGHT	13.81818182	14		1	0
2	437	0	Placebo	1	24	4/9/2015	84	HEIGHT	11.8	15		1	0
2	437	12	Placebo	1	30	6/16/2016	95.5	HEIGHT	13.8	15		1	0

2	437	24	Placebo	1	52	5/15/2017	98.5	HEIGHT	14.05	14		1	0
2	437	36	Placebo	1	65	6/9/2018	105.3	HEIGHT	16.45	15.5		0	0
2	437	48	Placebo	1	76	5/23/2019	111.7	HEIGHT	16.8	14.5		0	0
2	438	0	Placebo	0	42	4/9/2015	99.1	HEIGHT	15.15	15.5		1	0
2	438	12	Placebo	0	66	6/16/2016	106.5	HEIGHT	15.95	15		0	0
2	438	36	Placebo	0	92	6/9/2018	118.5	HEIGHT	18.65	15		0	0
2	438	48	Placebo	0	102	5/23/2019	122.8	HEIGHT	20.6	15.5		0	0
2	439	12	Placebo	1	12	6/16/2016	71.1	LENGTH	8.2	14		1	0
2	439	24	Placebo	1	25	5/15/2017	78.5	HEIGHT	9.15	14		1	0
2	439	36	Placebo	1	39	6/9/2018	85.8	HEIGHT	11.45	14.5		1	0
2	439	48	Placebo	1	50	5/23/2019	93.1	HEIGHT	13	15		1	0
2	439	60	Placebo	1	59	2/17/2020	98.5	HEIGHT	14.04545455	14.9		1	0
2	440	0	Placebo	0	54	4/9/2015	120.8	HEIGHT	18.45	15		1	0
2	440	36	Placebo	0	115	6/9/2018	126.5	HEIGHT	25.85	18.5		0	0
2	440	48	Placebo	0	125	5/23/2019	141.1	HEIGHT	27.8	17		0	0
2	443	24	Placebo	1	8	5/15/2017	66.5	LENGTH	7.1	13		1	0
2	443	36	Placebo	1	15	6/9/2018	80.6	LENGTH	10.1	15		1	0
2	443	48	Placebo	1	26	5/23/2019	88.3	HEIGHT	13.1	15.5		1	0
2	443	60	Placebo	1	35	2/17/2020	94.2	HEIGHT	14.40909091	15.5		1	0
2	444	24	Placebo	0	13	5/15/2017	75.1	HEIGHT	8.6	14		1	0
2	448	0	Placebo	1	36	4/9/2015	102.6	HEIGHT	14.95	14.5		1	0
2	448	12	Placebo	1	51	6/16/2016	109	HEIGHT	16.3	15		1	0
2	448	36	Placebo	1	75	6/9/2018	124.2	HEIGHT	21.75	15.5		0	0
2	448	48	Placebo	1	86	5/23/2019	129.4	HEIGHT	23.5	16		0	0
2	448	60	Placebo	1	95	2/17/2020	134.8	HEIGHT	27.13636364	17		0	0
2	454	24	Placebo	1	1	5/15/2017	51.5	LENGTH	3.8	11.5		1	0
2	455	0	Placebo	0	12	4/9/2015	70	LENGTH	6.6	12.5		1	0
2	455	12	Placebo	0	30	6/16/2016	80.1	HEIGHT	8.65	12.5		1	0
2	455	24	Placebo	0	44	5/15/2017	87.5	HEIGHT	10.4	12.5		1	0
2	455	36	Placebo	0	56	6/9/2018	96.7	HEIGHT	11.65	13		1	0
2	455	48	Placebo	0	67	5/23/2019	102.7	HEIGHT	13.2	13		0	0
2	455	60	Placebo	0	76	2/17/2020	106.7	HEIGHT	14.86363636	13.5		0	0
2	456	12	Placebo	1	10	6/16/2016	67.3	LENGTH	5.7	11		1	0
2	456	24	Placebo	1	25	5/15/2017	74.1	LENGTH	7.65	12		1	0
2	456	36	Placebo	1	37	6/9/2018	80.9	LENGTH	10.15	14		1	0
2	456	48	Placebo	1	48	5/23/2019	87.2	HEIGHT	11.85	14.5		1	0
2	456	60	Placebo	1	57	2/17/2020	94.9	HEIGHT	13.27272727	14		1	0
2	458	0	Placebo	1	7	4/9/2015	72.1	LENGTH	7.9	13.5		1	0
2	458	12	Placebo	1	30	6/16/2016	83.5	LENGTH	10.25	14		1	0
2	458	24	Placebo	1	36	5/15/2017	89.8	HEIGHT	12.15	14		1	0

2	458	36	Placebo	1	48	6/9/2018	97.9	HEIGHT	14.35	15	1	0
2	458	48	Placebo	1	59	5/23/2019	106.3	HEIGHT	16.6	15	1	0
2	459	0	Placebo	0	24	4/9/2015	89.8	HEIGHT	13.2	15.5	1	0
2	459	12	Placebo	0	42	6/16/2016	100.7	HEIGHT	15.65	16	1	0
2	459	24	Placebo	0	55	5/15/2017	108.4	HEIGHT	17.9	16.5	1	0
2	459	36	Placebo	0	64	6/9/2018	115.5	HEIGHT	18.8	16	0	0
2	459	48	Placebo	0	75	5/23/2019	122	HEIGHT	20.8	16.5	0	0
2	465	12	Placebo	1	4	6/16/2016	63.7	LENGTH	7.25	15	1	0
2	465	24	Placebo	1	17	5/15/2017	74.4	HEIGHT	8.75	15	1	0
2	465	36	Placebo	1	29	6/9/2018	79.5	HEIGHT	10.45	14.5	1	0
2	465	48	Placebo	1	40	5/23/2019	82.6	HEIGHT	11.05	14.5	1	0
2	465	60	Placebo	1	50	2/17/2020	88.2	HEIGHT	12.31818182	14	1	0
2	468	12	Placebo	1	54	6/16/2016	103.2	HEIGHT	17.5	17	1	0
2	468	36	Placebo	1	80	6/9/2018	114.8	HEIGHT	20.15	17	0	0
2	468	48	Placebo	1	91	5/23/2019	120.1	HEIGHT	20.95	16.5	0	0
2	468	60	Placebo	1	100	2/17/2020	123.6	HEIGHT	24.04545455	17	0	0
2	472	12	Placebo	0	12	6/16/2016	77.8	HEIGHT	9.25	14.5	1	0
2	472	24	Placebo	0	26	5/15/2017	88.2	LENGTH	11.3	14.5	1	0
2	472	36	Placebo	0	39	6/9/2018	96.4	HEIGHT	13.6	15.5	1	0
2	472	48	Placebo	0	50	5/23/2019	102.2	HEIGHT	15.15	15	1	0
2	8058	48	Placebo	0	13	5/23/2019	75.8	LENGTH	9.05	14.5	1	0
2	8058	60	Placebo	0	23	2/17/2020	74.4	HEIGHT	9.909090909	14	1	0
2	8064	48	Placebo	0	2	5/23/2019	66.3	LENGTH	7.8	13.5	1	0
2	8153	48	Placebo	0	9	5/23/2019	68.3	LENGTH	7.15	12.5	1	0
2	8153	60	Placebo	0	18	2/17/2020	75.6	HEIGHT	8.863636364	16	1	0
2	8234	36	Placebo	1	5	6/9/2018	59.5	LENGTH	5.5	12	1	0
2	8234	48	Placebo	1	14	5/23/2019	67.4	LENGTH	7.4	12.5	1	0
2	8234	60	Placebo	1	24	2/17/2020	78.2	HEIGHT	9.772727273	13.6	1	0
2	8244	48	Placebo	0	15	5/23/2019	75.6	LENGTH	9.35	13	1	0
2	8250	60	Placebo	0	2	2/17/2020	60.9	LENGTH	5.954545455	12	1	0
2	8584	36	Placebo	1	8	6/9/2018	68.6	LENGTH	7.9	14	1	0
2	8584	60	Placebo	1	28	2/17/2020	85.2	HEIGHT	11.77272727	14.5	1	0
2	8623	48	Placebo	0	12	5/23/2019	82.5	LENGTH	11.4	16	1	0
2	8653	36	Placebo	0	5	6/9/2018	64.2	LENGTH	7.25	14.5	1	0
2	8653	48	Placebo	0	12	5/23/2019	75.9	LENGTH	9.4	14	1	0
2	8653	60	Placebo	0	21	2/17/2020	80.7	HEIGHT	11.68181818	14.5	1	0
2	8965	48	Placebo	0	5	5/23/2019	68	LENGTH	7.15	12.5	1	0
2	8965	60	Placebo	0	12	2/17/2020	79.5	HEIGHT	9.681818182	13.5	1	0
2	8999	36	Placebo	0	5	6/9/2018	62.8	LENGTH	6.6	13	1	0
2	8999	48	Placebo	0	15	5/23/2019	73.1	LENGTH	8.45	13	1	0

2	8999	60	Placebo	0	25	2/17/2020	79.2	LENGTH	10.27272727	14.2		1	0
2	9248	60	Placebo	1	7	2/17/2020	64.6	LENGTH	7.090909091	13.5		1	0
2	9286	36	Placebo	0	8	6/9/2018	65.9	LENGTH	6.45	11.5		1	0
2	9286	48	Placebo	0	15	5/23/2019	77.2	HEIGHT	8.65	12		1	0
2	9286	60	Placebo	0	25	2/17/2020	80.9	HEIGHT	10	12.5		1	0
2	9300	36	Placebo	1	13	6/9/2018	71.1	LENGTH	7.5	14		1	0
2	9300	48	Placebo	1	23	5/23/2019	82	LENGTH	10.6	15.5		1	0
2	9300	60	Placebo	1	33	2/17/2020	90.1	HEIGHT	12.90909091	15		1	0
2	9361	60	Placebo	1	9	2/17/2020	72.6	HEIGHT	9.954545455	14.6		1	0
2	9459	36	Placebo	0	12	6/9/2018	71.2	LENGTH	7.25	12		1	0
2	9459	48	Placebo	0	22	5/23/2019	78.3	LENGTH	9	12.5		1	0
2	9542	36	Placebo	1	13	6/9/2018	73.1	LENGTH	8.85	15.5		1	0
2	9542	48	Placebo	1	24	5/23/2019	80.6	LENGTH	10.6	15		1	0
2	9542	60	Placebo	1	33	2/17/2020	84.4	HEIGHT	13.09090909	15.5		1	0
3	483	0	Placebo	0	48	4/6/2015	105.2	HEIGHT	13.2	13		0	1
3	483	36	Placebo	1	88	6/16/2018	120.6	HEIGHT	17.59090909	12.5		0	1
3	483	48	Placebo	0	99	6/7/2019	124.1	HEIGHT	18.86363636	14		0	1
3	484	24	Placebo	1	24	5/2/2017	74.9	HEIGHT	6.590909091	10.5		1	1
3	486	12	Placebo	0	2	7/25/2016	63.2	HEIGHT		12.5		0	1
3	486	24	Placebo	0	12	5/3/2017	71.8	LENGTH	8.318181818	13		0	1
3	486	36	Placebo	0	27	6/16/2018	81.5	LENGTH	10.72727273	13.5		1	1
3	486	60	Placebo	0	47	2/27/2020	95.1	HEIGHT	14.18181818	14		0	1
3	487	12	Placebo	1	4	7/25/2016	64.6	LENGTH		11.5		0	1
3	487	24	Placebo	1	22	5/2/2017	73.7	HEIGHT	7.409090909	11		0	1
3	488	0	Placebo	1	48	6/15/2015	81.3	HEIGHT	8.5	12		1	1
3	490	24	Placebo	1	55	5/3/2017	102.8	HEIGHT	13.40909091	13.5		1	1
3	493	24	Placebo	1	49	5/3/2017	99.4	HEIGHT	12.81818182	13	12	1	1
3	495	24	Placebo	0	8	5/19/2017	67.5	LENGTH	7.15	12.5		1	1
3	496	0	Placebo	0	24	5/11/2015	83.3	HEIGHT	11.1	15		0	1
3	498	0	Placebo	0	18	5/11/2015	80.4	HEIGHT	9.2	13	18	1	1
3	499	24	Placebo	1	39	5/19/2017	87.6	HEIGHT	10.65	12		1	1
3	500	0	Placebo	0	48	4/6/2015	88.6	HEIGHT	10.7	12.5		1	1
3	500	24	Placebo	0	75	5/19/2017	102.5	HEIGHT	14.05	14		0	1
3	500	36	Placebo	0	88	6/16/2018	108.5	HEIGHT	15.36363636	12.5		0	1
3	500	48	Placebo	0	99	6/7/2019	112.6	HEIGHT	16.68181818	13.4		0	1
3	500	60	Placebo	0	108	3/11/2020	116.5	HEIGHT	18	14		0	1
3	503	24	Placebo	0	1	5/2/2017	53.7	LENGTH	3.727272727	10		1	1
3	504	0	Placebo	1	12	4/6/2015	69.1	LENGTH	6.6	11.5		0	1
3	504	12	Placebo	1	30	7/25/2016	78.3	HEIGHT		13		0	1
3	504	24	Placebo	1	39	5/2/2017	83.4	HEIGHT	9.727272727	13.5		0	1

3	504	36	Placebo	1	52	6/16/2018	89.6	HEIGHT	11.18181818	13.5		0	1
3	504	48	Placebo	1	63	5/21/2019	96.4	HEIGHT	13	14		0	1
3	504	60	Placebo	1	72	2/27/2020	101.7	HEIGHT	13.63636364	14		0	1
3	511	0	Placebo	1	36	5/11/2015	82.2	HEIGHT	8.65	11		0	1
3	511	12	Placebo	1	41	7/26/2016	90.6	HEIGHT		12		0	1
3	511	24	Placebo	1	51	5/2/2017	96	HEIGHT	11.22727273	12		0	1
3	511	36	Placebo	1	64	6/17/2018	103.7	HEIGHT	13.04545455	12.5		0	1
3	511	48	Placebo	1	75	6/7/2019	109.5	HEIGHT	14.27272727	13		0	1
3	513	0	Placebo	1	48	6/15/2015	98.1	HEIGHT	14.25	15.5		1	1
3	513	24	Placebo	1	75	5/2/2017	109.9	HEIGHT	16.86363636	15		0	1
3	513	36	Placebo	1	88	6/17/2018	116.5	HEIGHT	19.09090909	15		0	1
3	513	48	Placebo	1	99	5/21/2019	121.6	HEIGHT	22.15	16		0	1
3	513	60	Placebo	1	108	2/27/2020	124.4	HEIGHT	23.95454545	16.2		0	1
3	516	0	Placebo	0	47	4/6/2015	96.5	HEIGHT	12.55	14.5		1	1
3	519	0	Placebo	0	48	4/6/2015	101.3	HEIGHT	14.2	13.5		0	1
3	519	12	Placebo	0	65	7/26/2016	109.8	HEIGHT		14		0	1
3	519	24	Placebo	0	75	5/2/2017	113.7	HEIGHT	17.59090909	14		0	1
3	520	12	Placebo	1	22	7/25/2016	77.8	HEIGHT		13.5		1	1
3	521	12	Placebo	0	50	7/25/2016	106.8	HEIGHT		14		1	1
3	527	12	Placebo	0	26	7/28/2016	74.2	HEIGHT		11.5	30	1	1
3	527	24	Placebo	0	39	5/19/2017	75.6	HEIGHT	7.15	10.5	30	1	1
3	528	24	Placebo	1	15	5/2/2017	78.1	HEIGHT	9.590909091	13		1	1
3	528	60	Placebo	1	48	3/15/2020	102.9	HEIGHT	17.8	16.4		1	1
3	530	12	Placebo	1	26	7/25/2016	79.1	HEIGHT		12		1	1
3	530	36	Placebo	1	52	6/17/2018	89.8	HEIGHT	10.81818182	12		1	1
3	532	12	Placebo	0	44	7/25/2016	95.9	HEIGHT		15		0	1
3	532	36	Placebo	0	70	6/16/2018	129.9	HEIGHT	26.45454545	16		0	1
3	532	48	Placebo	0	81	5/21/2019	113.2	HEIGHT	17.22727273	14		0	1
3	533	0	Placebo	1	36	5/11/2015	81.9	HEIGHT	8.95	12		1	1
3	533	12	Placebo	1	55	7/25/2016	89.4	HEIGHT		12.5		0	1
3	534	0	Placebo	0	6	6/15/2015	70.3	LENGTH	7.2	13		1	1
3	534	12	Placebo	0	24	7/26/2016	81.9	HEIGHT		13.5		0	1
3	534	24	Placebo	0	33	5/19/2017	88.3	HEIGHT	10.85	13.5		0	1
3	535	0	Placebo	1	7	4/6/2015	70.5	LENGTH	7.1	12	12	0	1
3	536	24	Placebo	0	7	5/3/2017	66.6	LENGTH	7.227272727	13.5		1	1
3	536	36	Placebo	0	15	6/16/2018	77.8	LENGTH	8.727272727	12		0	1
3	536	48	Placebo	0	26	5/21/2019	85.6	LENGTH	11	13		1	1
3	536	60	Placebo	0	35	3/11/2020	93.4	HEIGHT	12.65	14.6		1	1
3	537	0	Placebo	0	36	5/11/2015	97.3	HEIGHT	13.45	14		1	1
3	537	12	Placebo	0	54	7/25/2016	107.4	HEIGHT		13.5		1	1

3	537	36	Placebo	0	80	6/17/2018	115.2	HEIGHT	17.81818182	13.5		0	1
3	537	48	Placebo	0	91	6/7/2019	121.3	LENGTH	18.95454545	15.3		0	1
3	538	0	Placebo	1	12	6/15/2015	73.5	LENGTH	8.4	13.5		0	1
3	538	12	Placebo	1	26	7/25/2016	81.7	HEIGHT		13		0	1
3	538	24	Placebo	1	39	5/2/2017	86.7	HEIGHT	10.63636364	13.5		0	1
3	538	36	Placebo	1	52	6/16/2018	93.2	HEIGHT	13	14		0	1
3	538	48	Placebo	1	63	5/21/2019	99.7	HEIGHT	14.25	14		0	1
3	538	60	Placebo	1	72	2/27/2020	105.6	HEIGHT	14.90909091	13		0	1
3	539	0	Placebo	0	10	5/11/2015	72.3	LENGTH	7.95	13		1	1
3	539	12	Placebo	0	20	7/25/2016	83.2	HEIGHT		14.5		0	1
3	539	24	Placebo	0	33	5/3/2017	90.2	HEIGHT	12.18181818	14.5		0	1
3	540	12	Placebo	0	8	7/25/2016	70.1	LENGTH		13		1	1
3	540	24	Placebo	0	16	5/2/2017	81.1	HEIGHT	8.636363636	13		0	1
3	540	36	Placebo	0	29	6/16/2018	86.3	HEIGHT	10.27272727	13		1	1
3	540	60	Placebo	0	49	2/27/2020	98.7	HEIGHT	12.86363636	13		0	1
3	542	0	Placebo	1	6	6/15/2015	64.6	LENGTH	6	12.5		1	1
3	543	24	Placebo	1	8	5/2/2017	58.3	LENGTH	3.636363636	9.5		1	1
3	544	12	Placebo	0	23	7/26/2016	77.3	HEIGHT		12.5		1	1
3	546	0	Placebo	1	2	5/11/2015	65.5	LENGTH	6.15	12		0	1
3	546	12	Placebo	1	18	7/25/2016	77.8	HEIGHT		13.5		0	1
3	546	24	Placebo	1	27	5/2/2017	85.2	HEIGHT	10.5	14		0	1
3	546	36	Placebo	1	40	6/17/2018	94.2	HEIGHT	11.81818182	13.5		0	1
3	546	48	Placebo	1	51	6/7/2019	99.3	HEIGHT	14.09090909	14.5		1	1
3	546	60	Placebo	1	60	2/27/2020	105.1	HEIGHT	15.59090909	14.2		0	1
3	547	12	Placebo	1	30	7/25/2016	81.1	HEIGHT		14		1	1
3	550	0	Placebo	1	58	6/15/2015	90.7	HEIGHT	12	13.5		1	1
3	550	24	Placebo	1	87	5/2/2017	105.2	HEIGHT	14.54545455	14		0	1
3	550	36	Placebo	1	100	6/16/2018	114	HEIGHT	17.27272727	14		0	1
3	550	48	Placebo	1	111	5/21/2019	120.3	HEIGHT	19.75	15		0	1
3	550	60	Placebo	1	120	2/27/2020	124.5	HEIGHT	22	15.5		0	1
3	552	0	Placebo	0	4	4/6/2015	69.1	LENGTH	8.05	13.5	36	0	1
3	552	24	Placebo	0	31	5/2/2017	87.4	HEIGHT	11.90909091	15.5	36	0	1
3	553	24	Placebo	0	7	5/19/2017	67	LENGTH	7.2	13.5		1	1
3	553	36	Placebo	0	15	6/17/2018	77	LENGTH	8.954545455	13.5		0	1
3	553	48	Placebo	0	26	5/21/2019	82.9	LENGTH	10.5	13.5		0	1
3	555	24	Placebo	0	8	5/2/2017	57	LENGTH	3.954545455	10		1	1
3	556	0	Placebo	1	24	5/11/2015	84.2	HEIGHT	10	13		0	1
3	556	24	Placebo	1	51	5/2/2017	98.6	HEIGHT	14.09090909	14		0	1
3	556	48	Placebo	1	75	6/7/2019	112.2	HEIGHT	16.95454545	14.4		0	1
3	559	0	Placebo	1	12	4/6/2015	70.3	LENGTH	7	13		0	1

3	560	24	Placebo	0	5	5/19/2017	66.8	LENGTH	7.9	13	1	1
3	560	36	Placebo	0	18	6/16/2018	77.7	LENGTH	8.090909091	13	0	1
3	560	48	Placebo	0	28	5/21/2019	85.5	LENGTH	10.25	13	0	1
3	560	60	Placebo	0	38	3/11/2020	91.3	LENGTH	12.85	14.7	1	1
3	562	0	Placebo	1	48	5/11/2015	103.5	HEIGHT	14.85	14.5	0	1
3	562	24	Placebo	1	76	5/19/2017	116.3	HEIGHT	17.5	15	0	1
3	562	48	Placebo	1	100	6/7/2019	123.5	HEIGHT	20.27272727	15.9	0	1
3	562	60	Placebo	1	109	2/27/2020	126.9	HEIGHT	21.86363636	15.7	0	1
3	563	0	Placebo	0	59	6/15/2015	125.6	HEIGHT	19.9	13	1	1
3	567	12	Placebo	1	30	7/25/2016	93.3	HEIGHT		13.5	1	1
3	567	24	Placebo	1	43	5/2/2017	99	HEIGHT	13	13.5	1	1
3	569	0	Placebo	0	36	4/6/2015	87.1	HEIGHT	10.6	13	0	1
3	569	12	Placebo	0	55	7/25/2016	97.5	HEIGHT		15	1	1
3	570	24	Placebo	1	6	5/2/2017	59.8	LENGTH	5.863636364	13	1	1
3	570	36	Placebo	1	19	6/16/2018	69.7	LENGTH	8	13	1	1
3	570	48	Placebo	1	30	5/21/2019	76.4	LENGTH	9.35	13	0	1
3	570	60	Placebo	1	39	2/27/2020	84.2	HEIGHT	11.72727273	14.5	1	1
3	571	0	Placebo	0	48	4/6/2015	100	HEIGHT	12.9	13.5	0	1
3	571	24	Placebo	0	76	5/2/2017	114.1	HEIGHT	16.86363636	14.5	0	1
3	579	0	Placebo	1	54	5/11/2015	102.9	HEIGHT	11.3	12	0	1
3	579	48	Placebo	1	91	6/7/2019	128.2	HEIGHT	18.81818182	13.2	0	1
3	580	24	Placebo	1	22	5/19/2017	77.6	HEIGHT	8.45	13	1	1
3	582	12	Placebo	0	45	7/25/2016	91.5	HEIGHT		13.5	1	1
3	582	24	Placebo	0	55	5/3/2017	96.8	HEIGHT	12.27272727	13	1	1
3	583	12	Placebo	0	54	7/28/2016	87.8	HEIGHT		14	1	1
3	583	60	Placebo	0	100	2/27/2020	122.7	HEIGHT	22.04545455	15.4	0	1
3	585	0	Placebo	1	12	4/6/2015	63.3	LENGTH	5.5	12	0	1
3	585	24	Placebo	1	39	5/19/2017	79.7	HEIGHT	8.6	11.5	0	1
3	585	36	Placebo	1	52	6/16/2018	88.2	HEIGHT	11.45454545	13.5	0	1
3	586	0	Placebo	0	30	5/11/2015	87.2	HEIGHT	9.95	12.5	0	1
3	586	12	Placebo	0	49	7/25/2016	96.6	HEIGHT		14	0	1
3	586	24	Placebo	0	58	5/19/2017	102.4	HEIGHT	12.95	13	1	1
3	586	36	Placebo	0	71	6/17/2018	107.8	HEIGHT	14.13636364	13	0	1
3	587	0	Placebo	0	5	6/15/2015	68.1	LENGTH	6.55	12.5	1	1
3	587	12	Placebo	0	23	7/25/2016	78.3	HEIGHT		13.5	0	1
3	587	24	Placebo	0	33	5/3/2017	85.6	HEIGHT	9.909090909	13	1	1
3	587	36	Placebo	0	46	6/16/2018	93.9	HEIGHT	11.95454545	13	1	1
3	587	48	Placebo	0	57	5/21/2019	99.8	HEIGHT	12.75	13	0	1
3	587	60	Placebo	0	66	2/27/2020	105.3	HEIGHT	14.5	12.9	0	1
3	588	0	Placebo	0	59	4/6/2015	124.3	HEIGHT	21.05	16	1	1

3	589	12	Placebo	0	44	7/28/2016	85.7	HEIGHT		14		0	1
3	589	36	Placebo	0	57	6/16/2018	100.3	HEIGHT	13.81818182	13		0	1
3	589	48	Placebo	0	68	5/21/2019	105.9	HEIGHT	14.54545455	13		0	1
3	591	12	Placebo	1	29	7/25/2016	78.4	HEIGHT		14		1	1
3	591	36	Placebo	1	52	6/17/2018	93.5	HEIGHT	14.54545455	14.5		1	1
3	593	24	Placebo	1	9	5/2/2017	68.5	LENGTH	6.681818182	13	48	1	1
3	593	36	Placebo	1	21	6/16/2018	80.1	LENGTH	9.363636364	14	48	1	1
3	595	24	Placebo	0	43	5/2/2017	90	HEIGHT	11.72727273	14.5	30	1	1
3	596	0	Placebo	1	54	6/15/2015	103.3	HEIGHT	15.1	12.3		1	1
3	596	24	Placebo	1	81	5/19/2017	116	HEIGHT	18.5	15.5		0	1
3	596	36	Placebo	1	94	6/16/2018	120.4	HEIGHT	20.86363636	15.5		0	1
3	596	48	Placebo	1	105	6/7/2019	124.6	HEIGHT	24.81818182	18		0	1
3	600	24	Placebo	0	25	5/19/2017	94.2	HEIGHT	13.4	15.5		1	1
3	600	36	Placebo	0	37	6/17/2018	101.5	HEIGHT	14.63636364	14.5		1	1
3	600	48	Placebo	0	48	6/7/2019	150.4	HEIGHT	37	16		1	1
3	600	60	Placebo	0	58	3/18/2020	111.9	HEIGHT	18.7	15.1		1	1
3	603	0	Placebo	0	48	4/6/2015	95.2	HEIGHT	13.05	14.5		1	1
3	603	24	Placebo	0	75	5/3/2017	107.7	HEIGHT	17.22727273	15		0	1
3	604	12	Placebo	1	26	7/25/2016	81.8	HEIGHT		14.5		1	1
3	606	24	Placebo	1	51	5/19/2017	104.4	HEIGHT	15.3	15.5		1	1
3	607	24	Placebo	0	6	5/19/2017	62.1	LENGTH	4.65	10.5		1	1
3	607	36	Placebo	0	19	6/16/2018	74.6	LENGTH	7.318181818	12		1	1
3	607	60	Placebo	0	39	3/15/2020	85.1	HEIGHT	10.05	14		1	1
3	609	0	Placebo	1	24	4/6/2015	82.6	HEIGHT	12.35	16		1	1
3	609	12	Placebo	1	42	7/25/2016	93.3	HEIGHT		16.5		0	1
3	609	24	Placebo	1	51	5/2/2017	98.9	HEIGHT	16.09090909	16		1	1
3	609	36	Placebo	1	64	6/16/2018	106.5	HEIGHT	19.04545455	16.5		0	1
3	609	48	Placebo	1	75	5/21/2019	112.3	HEIGHT	19.7	16		0	1
3	609	60	Placebo	1	84	2/27/2020	117	HEIGHT	21.27272727	15.7		0	1
3	612	0	Placebo	0	36	5/11/2015	86.2	HEIGHT	9.15	12		0	1
3	614	12	Placebo	1	2	7/28/2016	58.8	LENGTH		10.5		0	1
3	614	36	Placebo	1	25	6/16/2018	78.6	LENGTH	9.227272727	11.5		1	1
3	614	48	Placebo	1	35	5/21/2019	85.2	HEIGHT	10.59090909	12		1	1
3	616	0	Placebo	1	24	4/6/2015	88.2	HEIGHT	10.75	14		0	1
3	616	12	Placebo	1	38	7/26/2016	97.2	HEIGHT		15.5		0	1
3	616	24	Placebo	1	51	5/3/2017	102.8	HEIGHT	15.31818182	15		0	1
3	616	36	Placebo	1	64	6/17/2018	109.6	HEIGHT	16.54545455	15.5		0	1
3	616	60	Placebo	1	84	2/27/2020	119.4	HEIGHT	19.59090909	15		0	1
3	619	24	Placebo	0	15	5/2/2017	67.4	LENGTH	5.954545455	10.5		1	1
3	620	0	Placebo	0	2	6/15/2015	70.7	LENGTH	8.35	15		1	1

3	620	12	Placebo	1	12	7/25/2016	81.7	HEIGHT		14		0	1
3	622	0	Placebo	1	48	4/6/2015	93.3	HEIGHT	11.8	14.5		0	1
3	622	12	Placebo	1	54	7/25/2016	101.7	HEIGHT		14		1	1
3	625	0	Placebo	1	36	4/6/2015	89.3	HEIGHT	11.7	14.5		1	1
3	625	12	Placebo	1	50	7/25/2016	95.9	HEIGHT		12.5		0	1
3	625	24	Placebo	1	63	5/2/2017	102.7	HEIGHT	13.40909091	14		0	1
3	625	36	Placebo	1	76	6/16/2018	106.3	HEIGHT	12.68181818	12.5		0	1
3	625	48	Placebo	1	87	5/21/2019	110.2	HEIGHT	15.85	13.5		0	1
3	625	60	Placebo	1	96	2/27/2020	117	HEIGHT	18.54545455	14.5		0	1
3	626	0	Placebo	0	48	5/11/2015	98	HEIGHT	14.7	14		0	1
3	626	36	Placebo	0	88	6/17/2018	116.7	HEIGHT	18.59090909	14		0	1
3	627	12	Placebo	0	56	7/25/2016	100.3	HEIGHT		13		1	1
3	628	12	Placebo	0	52	7/25/2016	111.7	HEIGHT		16.5		1	1
3	629	12	Placebo	1	11	7/25/2016	72.5	HEIGHT		13.5		0	1
3	629	24	Placebo	1	22	5/2/2017	79.4	HEIGHT	9.090909091	13		0	1
3	629	36	Placebo	1	35	6/17/2018	86	HEIGHT	11.18181818	13		0	1
3	629	48	Placebo	1	46	6/7/2019	95.1	HEIGHT	12.63636364	13.3		1	1
3	629	60	Placebo	1	55	2/27/2020	100.8	HEIGHT	14.18181818	13.7		0	1
3	631	0	Placebo	1	30	5/11/2015	71.3	LENGTH	7	13		0	1
3	631	12	Placebo	1	44	7/25/2016	81.2	HEIGHT		12.5		0	1
3	633	0	Placebo	0	48	4/6/2015	108.3	HEIGHT	16.05	15		0	1
3	633	12	Placebo	0	54	7/25/2016	115.5	HEIGHT		15		1	1
3	633	36	Placebo	0	80	6/16/2018	124.9	HEIGHT	22.13636364	14.5		0	1
3	633	48	Placebo	0	91	5/21/2019	129.4	HEIGHT	25.2	15.5		0	1
3	634	0	Placebo	0	24	4/6/2015	76.8	HEIGHT	8.6	12.5		0	1
3	634	24	Placebo	0	52	5/2/2017	91.1	HEIGHT	11.68181818	13		0	1
3	636	0	Placebo	0	36	4/6/2015	88.7	HEIGHT	11.45	13.5		1	1
3	638	12	Placebo	0	54	7/28/2016	97.8	HEIGHT		13.5		1	1
3	641	0	Placebo	0	36	6/15/2015	100.4	HEIGHT	13.9	13		1	1
3	641	12	Placebo	0	50	7/25/2016	108.7	HEIGHT		15		0	1
3	643	12	Placebo	1	50	7/28/2016	99.2	HEIGHT		15		1	1
3	644	24	Placebo	1	39	5/3/2017	93.3	HEIGHT	10.63636364	13		1	1
3	644	36	Placebo	1	52	6/17/2018	100.3	HEIGHT	12.09090909	13		1	1
3	645	0	Placebo	0	24	4/6/2015	71.4	LENGTH	6.5	13	18	1	1
3	648	0	Placebo	0	24	4/6/2015	79.9	LENGTH	8.8	13		0	1
3	648	24	Placebo	0	51	5/3/2017	93.7	HEIGHT	12.27272727	14		0	1
3	651	12	Placebo	1	88	7/25/2016	121.3	HEIGHT		14		0	1
3	652	12	Placebo	1	59	7/26/2016	97.4	HEIGHT		12		1	1
3	653	0	Placebo	1	59	4/6/2015	110.7	HEIGHT	15.95	14.5		0	1
3	653	48	Placebo	1	127	6/7/2019	135	HEIGHT	26.40909091	17		0	1

3	653	60	Placebo	1	136	2/27/2020	138.2	HEIGHT	29.54545455	16.7	0	1
3	654	0	Placebo	1	18	6/15/2015	112	HEIGHT	16.2	14.5	1	1
3	656	0	Placebo	0	48	5/11/2015	85.2	HEIGHT	10.15	13.5	0	1
3	656	12	Placebo	0	42	7/25/2016	92.8	HEIGHT		14.5	0	1
3	656	24	Placebo	0	56	5/2/2017	98	HEIGHT	13.36363636	13.5	1	1
3	656	48	Placebo	0	80	6/7/2019	105.8	LENGTH	15.18181818	15.2	0	1
3	659	0	Placebo	0	36	6/15/2015	89.9	HEIGHT	11.45	14	1	1
3	659	36	Placebo	0	80	6/16/2018	109.5	HEIGHT	16.09090909	14	0	1
3	659	48	Placebo	0	91	6/7/2019	114	HEIGHT	17	14.9	0	1
3	660	24	Placebo	1	55	5/19/2017	101.1	HEIGHT	12.05	12.5	1	1
3	663	12	Placebo	1	42	7/25/2016	85.9	HEIGHT		14	0	1
3	663	24	Placebo	0	52	5/2/2017	90	HEIGHT	11.63636364	12	0	1
3	664	0	Placebo	0	36	5/11/2015	90.7	HEIGHT	11.8	14.5	1	1
3	664	12	Placebo	0	50	7/25/2016	100.2	HEIGHT		15	0	1
3	664	24	Placebo	0	63	5/3/2017	105.7	HEIGHT	14.36363636	12.5	0	1
3	664	36	Placebo	0	76	6/17/2018	111.2	HEIGHT	15.90909091	13.5	0	1
3	664	48	Placebo	0	87	6/7/2019	117.5	HEIGHT	18.40909091	14.8	0	1
3	665	24	Placebo	1	21	5/19/2017	79	HEIGHT	9.5	13.5	1	1
3	673	24	Placebo	0	56	5/19/2017	103.5	HEIGHT	13.3	13	1	1
3	676	0	Placebo	1	48	6/15/2015	97.5	HEIGHT	12.75	13.5	1	1
3	676	24	Placebo	1	77	5/19/2017	110.5	HEIGHT	15.35	13.5	0	1
3	676	36	Placebo	1	89	6/17/2018	117.7	HEIGHT	16.90909091	13.5	0	1
3	677	12	Placebo	0	53	7/25/2016	106.2	HEIGHT		14.5	1	1
3	678	0	Placebo	1	59	4/6/2015	101.7	HEIGHT	12.3	13	0	1
3	679	0	Placebo	1	12	6/15/2015	87.6	HEIGHT	10.75	15	1	1
3	680	0	Placebo	0	36	5/11/2015	87.2	HEIGHT	10.85	13	0	1
3	680	12	Placebo	0	50	7/25/2016	99.2	HEIGHT		14	0	1
3	680	24	Placebo	0	63	5/3/2017	104.7	HEIGHT	15	14	0	1
3	684	0	Placebo	0	48	4/6/2015	87.2	HEIGHT	14.3	15	1	1
3	684	12	Placebo	0	63	7/25/2016	93.3	HEIGHT		15	0	1
3	684	24	Placebo	0	76	5/2/2017	98.7	HEIGHT	17.45454545	15.5	0	1
3	684	36	Placebo	0	89	6/17/2018	105.7	HEIGHT	18.95454545	15	0	1
3	684	60	Placebo	0	105	3/11/2020	113.6	HEIGHT	22.5	18	0	1
3	685	0	Placebo	0	23	5/11/2015	82.3	HEIGHT	11.85	16.5	0	1
3	686	12	Placebo	1	36	7/25/2016	75.2	LENGTH		13.5	1	1
3	688	0	Placebo	1	59	4/6/2015	119.4	HEIGHT	18.35	14	1	1
3	688	12	Placebo	1	92	7/26/2016	125.8	HEIGHT		15	0	1
3	688	36	Placebo	1	118	6/16/2018	133.2	HEIGHT	24.22727273	15	0	1
3	688	48	Placebo	1	129	6/7/2019	136.3	HEIGHT	25.63636364	16	0	1
3	688	60	Placebo	1	138	3/15/2020	140.4	HEIGHT	29.15	17	0	1

3	689	0	Placebo	0	36	6/15/2015	80.8	LENGTH	9.1	13		1	1
3	690	0	Placebo	1	24	5/11/2015	79.5	HEIGHT	8.5	13		0	1
3	690	60	Placebo	1	83	3/15/2020	112.9	HEIGHT	15.25	13.5		0	1
3	692	12	Placebo	0	7	7/25/2016	67.2	LENGTH		12		0	1
3	692	36	Placebo	0	28	6/16/2018	81.3	LENGTH	9.818181818	12		1	1
3	692	60	Placebo	0	48	3/11/2020	96.8	HEIGHT	12.8	14		0	1
3	693	0	Placebo	1	4	4/6/2015	65.7	LENGTH	7.05	13		1	1
3	693	12	Placebo	1	18	7/25/2016	79.7	HEIGHT		13		0	1
3	693	24	Placebo	1	31	5/19/2017	87.2	HEIGHT	12.45	13		1	1
3	693	36	Placebo	1	44	6/17/2018	97.3	HEIGHT	15.22727273	14		1	1
3	693	48	Placebo	0	55	6/7/2019	104.1	HEIGHT	16.36363636	14		1	1
3	694	12	Placebo	1	2	7/28/2016	52.5	LENGTH		9	60	1	1
3	694	36	Placebo	1	25	6/16/2018	71	LENGTH	6.454545455	9.5	60	1	1
3	694	48	Placebo	1	35	5/21/2019	78.3	HEIGHT	8.136363636	11	60	0	1
3	695	0	Placebo	0	12	4/6/2015	68.8	LENGTH	6.7	12		1	1
3	695	12	Placebo	0	26	7/25/2016	81.2	HEIGHT		13.5		0	1
3	695	24	Placebo	0	39	5/2/2017	88.8	HEIGHT	11.13636364	14.5		0	1
3	695	36	Placebo	0	52	6/16/2018	96.5	HEIGHT	13.09090909	14		1	1
3	695	48	Placebo	0	63	5/21/2019	103.6	HEIGHT	14.85	14		0	1
3	695	60	Placebo	0	72	2/27/2020	108.7	HEIGHT	15.36363636	13.2		0	1
3	697	0	Placebo	1	36	4/6/2015	98.7	HEIGHT	14.65	15.5		0	1
3	698	0	Placebo	0	36	5/11/2015	93.3	HEIGHT	11.15	12.5		0	1
3	698	12	Placebo	0	50	7/25/2016	100	HEIGHT		13		0	1
3	698	36	Placebo	0	76	6/17/2018	110.7	HEIGHT	15.18181818	13		0	1
3	698	48	Placebo	0	87	6/7/2019	114.2	HEIGHT	16.86363636	13.7		0	1
3	699	24	Placebo	0	48	5/2/2017	100.2	HEIGHT	14.18181818	14.5		1	1
3	700	0	Placebo	1	36	6/15/2015	94.5	HEIGHT	11.75	13		1	1
3	700	24	Placebo	1	63	5/3/2017	109.2	HEIGHT	15.18181818	14		0	1
3	700	48	Placebo	1	87	5/21/2019	120.5	HEIGHT	18.77272727	14		0	1
3	701	12	Placebo	0	50	7/25/2016	95	HEIGHT		15		1	1
3	702	0	Placebo	1	24	4/6/2015	78.8	HEIGHT	8.45	12		1	1
3	702	12	Placebo	1	41	7/26/2016	90	HEIGHT		13		0	1
3	702	24	Placebo	1	51	5/2/2017	95.7	HEIGHT	12.63636364	13.5		1	1
3	702	36	Placebo	1	64	6/17/2018	104.7	HEIGHT	13.90909091	13		0	1
3	702	48	Placebo	1	75	6/7/2019	111	HEIGHT	16.59090909	15		0	1
3	705	0	Placebo	0	4	4/6/2015	65.9	LENGTH	5.85	11.5		0	1
3	705	24	Placebo	0	27	5/3/2017	83.3	HEIGHT	9.181818182	12		0	1
3	705	36	Placebo	0	40	6/16/2018	89.5	HEIGHT	10.95454545	12.5		1	1
3	705	48	Placebo	0	51	6/7/2019	97.1	HEIGHT	13	14		1	1
3	707	0	Placebo	0	12	4/6/2015	68.4	LENGTH	7.1	12.5	30	0	1

3	708	24	Placebo	0	22	5/19/2017	82.3	HEIGHT	9.8	13.5	1	1
3	708	60	Placebo	0	55	3/18/2020	101.2	HEIGHT	14.7	13.7	1	1
3	710	0	Placebo	0	38	4/6/2015	93.5	HEIGHT	12.15	14.5	1	1
3	710	12	Placebo	0	42	7/25/2016	100.7	HEIGHT		14.5	0	1
3	710	60	Placebo	0	88	2/27/2020	121.7	HEIGHT	20.59090909	15	0	1
3	712	0	Placebo	0	1	4/6/2015	62.3	HEIGHT	5.55	13	1	1
3	712	12	Placebo	0	18	7/25/2016	76.3	HEIGHT		12	0	1
3	712	24	Placebo	0	27	5/19/2017	81.7	HEIGHT	9.65	13.5	0	1
3	712	36	Placebo	0	41	6/17/2018	89.6	HEIGHT	11.90909091	13.5	0	1
3	712	60	Placebo	0	61	2/27/2020	111.5	HEIGHT	13.72727273	13.2	0	1
3	713	12	Placebo	0	10	7/25/2016	68.1	LENGTH		13.5	1	1
3	713	24	Placebo	0	22	5/2/2017	77.6	LENGTH	8.272727273	14	1	1
3	713	36	Placebo	0	35	6/16/2018	87.1	LENGTH	10.5	13.5	0	1
3	713	48	Placebo	0	46	6/7/2019	95.5	HEIGHT	12.5	14.5	1	1
3	713	60	Placebo	0	55	2/27/2020	100.2	HEIGHT	13.45454545	14	0	1
3	714	24	Placebo	1	31	5/19/2017	86.8	LENGTH	9.9	13	1	1
3	714	48	Placebo	1	55	6/7/2019	100.1	HEIGHT	13.09090909	13.5	1	1
3	715	0	Placebo	0	48	4/6/2015	94.6	HEIGHT	11.7	13	0	1
3	716	0	Placebo	0	24	4/6/2015	86.1	HEIGHT	11.2	14.5	0	1
3	716	12	Placebo	0	38	7/25/2016	93.7	HEIGHT		15	0	1
3	716	24	Placebo	0	51	5/2/2017	97.2	HEIGHT	14.04545455	14.5	1	1
3	717	0	Placebo	1	59	4/6/2015	105.7	HEIGHT	15.25	14.5	0	1
3	719	24	Placebo	1	56	5/2/2017	95.4	HEIGHT	12.90909091	15	1	1
3	721	0	Placebo	0	24	5/11/2015	82.8	HEIGHT	9.7	13	0	1
3	723	0	Placebo	0	48	4/6/2015	100.2	HEIGHT	14	16	0	1
3	723	24	Placebo	0	67	5/2/2017	111.3	HEIGHT	16.22727273	15	0	1
3	723	60	Placebo	0	100	2/27/2020	126.4	HEIGHT	21.27272727	14.6	0	1
3	727	24	Placebo	1	55	5/19/2017	98.3	HEIGHT	12.4	13.5	1	1
3	728	24	Placebo	0	31	5/2/2017	91.9	LENGTH	10.90909091	13.5	1	1
3	730	0	Placebo	1	6	5/11/2015	63.8	LENGTH	6.35	13.5	0	1
3	730	12	Placebo	1	18	7/25/2016	78.7	HEIGHT		13.5	0	1
3	730	24	Placebo	1	31	5/2/2017	84	HEIGHT	9.818181818	13	0	1
3	730	48	Placebo	1	54	6/7/2019	94.1	HEIGHT	11.81818182	13	1	1
3	731	0	Placebo	1	4	6/15/2015	67.5	LENGTH	7.75	14.5	1	1
3	731	24	Placebo	1	31	5/2/2017	85.9	HEIGHT	11.04545455	15	1	1
3	731	36	Placebo	1	44	6/16/2018	95.4	HEIGHT	13.31818182	14.5	0	1
3	731	48	Placebo	1	55	6/7/2019	101	HEIGHT	15.45454545	15.5	0	1
3	731	60	Placebo	1	56	2/27/2020	106.9	HEIGHT	16.09090909	14.3	0	1
3	733	0	Placebo	0	36	4/6/2015	81.2	HEIGHT	10.25	15	0	1
3	733	12	Placebo	0	51	7/28/2016	89.2	HEIGHT		14.5	1	1

3	733	24	Placebo	0	64	5/3/2017	95.1	HEIGHT	12.95454545	15		0	1
3	735	12	Placebo	0	12	7/25/2016	73.3	HEIGHT		12		1	1
3	735	36	Placebo	0	37	6/16/2018	88.2	HEIGHT	11.36363636	13		0	1
3	735	48	Placebo	0	48	6/7/2019	93.2	HEIGHT	11.68181818	13.2		0	1
3	737	0	Placebo	0	59	4/6/2015	116.5	HEIGHT	18.4	14.5		1	1
3	740	0	Placebo	1	1	5/11/2015	59.9	LENGTH	5.1	11.5		0	1
3	741	12	Placebo	1	6	7/25/2016	61.2	LENGTH		11		0	1
3	741	36	Placebo	1	29	6/16/2018	80.2	LENGTH	10.5	14.5		0	1
3	741	60	Placebo	1	49	3/11/2020	95.3	HEIGHT	13.45	15.5		0	1
3	8039	36	Placebo	0	11	6/16/2018	66.8	LENGTH	5.863636364	11	54	1	1
3	8039	48	Placebo	0	22	5/21/2019	73.6	HEIGHT	7.95	11.5	54	0	1
3	8123	60	Placebo	1	31	2/27/2020	74.2	HEIGHT	6.818181818	10.5		1	1
3	8129	36	Placebo	0	34	6/16/2018	86.5	HEIGHT	12	14		1	1
3	8129	48	Placebo	0	45	5/21/2019	93.3	HEIGHT	13	14.5		1	1
3	8174	36	Placebo	0	52	6/17/2018	107.9	HEIGHT	16	14		1	1
3	8188	60	Placebo	0	10	3/18/2020	70	HEIGHT	8.5	14.2		1	1
3	8200	60	Placebo	1	49	3/15/2020	98.4	HEIGHT	13.05	13		1	1
3	8228	36	Placebo	0	50	6/16/2018	91.9	HEIGHT	11.95454545	12.5		1	1
3	8249	36	Placebo	0	23	6/17/2018	79.6	LENGTH	8.954545455	13		1	1
3	8256	36	Placebo	0	28	6/17/2018	87.8	LENGTH	11.31818182	14.5		1	1
3	8260	60	Placebo	0	13	3/15/2020	75.8	LENGTH	8.55	15		1	1
3	8267	48	Placebo	0	67	5/21/2019	103.3	HEIGHT	14.72727273	14		0	1
3	8267	60	Placebo	0	76	2/27/2020	107.8	HEIGHT	15.36363636	13.5		0	1
3	8277	36	Placebo	1	11	6/16/2018	66.1	LENGTH	6.363636364	12		1	1
3	8277	48	Placebo	1	20	5/21/2019	77.3	HEIGHT	8.75	13		0	1
3	8338	60	Placebo	0	6	3/18/2020	64	LENGTH	5.9	11.7		1	1
3	8386	36	Placebo	1	1	6/17/2018	58.4	LENGTH	4.136363636	8	48	1	1
3	8423	36	Placebo	1	44	7/1/2018	88.9	HEIGHT	11.15	12		1	1
3	8423	60	Placebo	1	55	2/27/2020	99.9	HEIGHT	14.68181818	13.7		1	1
3	8433	36	Placebo	1	21	6/17/2018	78.2	LENGTH	9.363636364	13.5		1	1
3	8436	48	Placebo	0	12	5/21/2019	70.2	LENGTH	7.25	12.5		1	1
3	8436	60	Placebo	0	21	2/27/2020	77	LENGTH	10.36363636	13		1	1
3	8469	36	Placebo	0	12	7/1/2018	75.6	LENGTH	9.4	13.5		1	1
3	8469	48	Placebo	0	22	6/7/2019	83	HEIGHT	11.18181818	14.5		1	1
3	8469	60	Placebo	0	32	2/27/2020	90.1	HEIGHT	12.68181818	13.9		0	1
3	8491	48	Placebo	1	12	6/7/2019	67.8	LENGTH	6.227272727	12		1	1
3	8491	60	Placebo	1	21	3/11/2020	74.3	HEIGHT	7.5	12.5		0	1
3	8495	48	Placebo	1	22	5/21/2019	80.8	HEIGHT	10.7	15		1	1
3	8499	48	Placebo	0	51	6/7/2019	103.3	HEIGHT	15.5	15.4		1	1
3	8521	36	Placebo	0	37	7/1/2018	90.6	HEIGHT	12.95	14.5		1	1

3	8521	60	Placebo	0	58	3/15/2020	103.2	HEIGHT	15.35	14.7	1	1
3	8545	36	Placebo	0	37	7/1/2018	86.6	LENGTH	12.3	15	1	1
3	8545	60	Placebo	0	58	3/18/2020	96.3	HEIGHT	14.5	14.5	1	1
3	8588	36	Placebo	0	19	6/16/2018	75.5	LENGTH	7.636363636	12	1	1
3	8588	60	Placebo	0	39	3/18/2020	84.4	HEIGHT	9.15	12.5	1	1
3	8619	60	Placebo	1	20	3/11/2020	80	LENGTH	10.1	15	1	1
3	8621	48	Placebo	1	15	6/7/2019	68.8	LENGTH	5.636363636	10	1	1
3	8621	60	Placebo	1	25	3/15/2020	75.2	HEIGHT	8.85	14.8	1	1
3	8642	60	Placebo	1	7	2/27/2020	65.1	LENGTH	6	11.3	1	1
3	8643	36	Placebo	0	6	6/17/2018	68.6	LENGTH	7	12.5	1	1
3	8643	48	Placebo	0	14	5/21/2019	77.1	LENGTH	8.5	13	0	1
3	8659	60	Placebo	1	56	2/27/2020	106.6	HEIGHT	15.54545455	14.2	1	1
3	8670	36	Placebo	0	24	6/16/2018	77.2	HEIGHT	8	13	1	1
3	8729	60	Placebo	1	8	3/11/2020	65.5	LENGTH	6.15	13	1	1
3	8745	60	Placebo	1	22	3/15/2020	87.3	HEIGHT	11.7	15	1	1
3	8757	36	Placebo	0	47	6/16/2018	79.1	LENGTH	8.409090909	11	1	1
3	8762	48	Placebo	0	35	5/21/2019	89.1	HEIGHT	11.18181818	13	1	1
3	8762	60	Placebo	1	45	3/15/2020	93.7	HEIGHT	12.4	14.3	1	1
3	8787	48	Placebo	0	39	6/7/2019	81.3	HEIGHT	10.31818182	13.3	1	1
3	8800	48	Placebo	0	26	5/21/2019	81.2	HEIGHT	10.3	14	1	1
3	8836	60	Placebo	0	32	3/18/2020	92.1	LENGTH	12.8	15.2	1	1
3	8851	48	Placebo	0	57	5/21/2019	113.2	HEIGHT	18.22727273	15	1	1
3	8893	48	Placebo	0	33	6/7/2019	70.9	HEIGHT	7.181818182	13.3	1	1
3	8916	60	Placebo	0	28	3/18/2020	86.9	HEIGHT	12.3	14	1	1
3	8951	36	Placebo	1	10	7/1/2018	69.4	LENGTH	7.2	12	1	1
3	8951	60	Placebo	1	25	3/11/2020	85.9	HEIGHT	10.9	14.3	0	1
3	8989	60	Placebo	1	32	3/15/2020	89.9	HEIGHT	13.95	16	1	1
3	9027	36	Placebo	0	25	7/1/2018	77.6	HEIGHT	8.15	12	1	1
3	9027	60	Placebo	0	45	2/27/2020	88.1	HEIGHT	10.72727273	13.2	1	1
3	9059	60	Placebo	1	32	3/15/2020	88.4	HEIGHT	13.25	17	1	1
3	9073	60	Placebo	1	14	2/27/2020	76	HEIGHT	8.136363636	11.9	1	1
3	9121	48	Placebo	0	55	5/21/2019	97.1	HEIGHT	13.75	13.5	1	1
3	9182	60	Placebo	1	38	3/18/2020	92.5	HEIGHT	14.55	14.9	1	1
3	9197	36	Placebo	1	11	6/16/2018	69.6	LENGTH	7.454545455	13	1	1
3	9197	48	Placebo	1	22	5/21/2019	76.5	LENGTH	8.7	12.5	1	1
3	9197	60	Placebo	1	32	2/27/2020	84.7	HEIGHT	10.27272727	14.2	0	1
3	9201	48	Placebo	1	32	5/21/2019	86.5	HEIGHT	10.45454545	14	1	1
3	9225	60	Placebo	1	12	3/18/2020	72.4	HEIGHT	7.8	12.5	1	1
3	9277	36	Placebo	1	40	6/16/2018	87.6	HEIGHT	9.5	12	1	1
3	9282	48	Placebo	0	33	6/7/2019	88.6	HEIGHT	12.45454545	16	1	1

3	9282	60	Placebo	0	43	3/18/2020	97.1	HEIGHT	15.05	16		1	1
3	9311	60	Placebo	1	58	3/11/2020	115.5	HEIGHT	18.3	15		1	1
3	9353	48	Placebo	1	58	6/7/2019	105.9	HEIGHT	17.68181818	15.5		1	1
3	9388	60	Placebo	1	43	3/18/2020	88.3	HEIGHT	10.75	12.7		1	1
3	9391	60	Placebo	0	4	3/11/2020	56.5	LENGTH	3.7	9		1	1
3	9427	60	Placebo	1	30	3/18/2020	85.7	LENGTH	11.5	14		1	1
3	9444	60	Placebo	1	45	3/11/2020	89.6	HEIGHT	13.1	15.8		1	1
3	9460	36	Placebo	0	11	6/16/2018	76.2	LENGTH	8.5	13.5		1	1
3	9460	48	Placebo	0	20	5/21/2019	83.5	HEIGHT	10.2	13		1	1
3	9460	60	Placebo	0	30	2/27/2020	88.3	HEIGHT	11.77272727	13.7		0	1
3	9470	48	Placebo	0	15	6/7/2019	71.8	LENGTH	7.5	12.3		1	1
3	9470	60	Placebo	0	25	3/15/2020	77.9	HEIGHT	8.25	11		1	1
3	9486	48	Placebo	0	55	5/21/2019	100.6	HEIGHT	14.5	14		1	1
3	9538	36	Placebo	0	15	6/16/2018	73.2	LENGTH	8	13.5		1	1
3	9538	60	Placebo	0	35	2/27/2020	82.6	HEIGHT	11.13636364	13		1	1
3	9555	36	Placebo	1	46	7/1/2018	94.3	HEIGHT	12.5	12	42	1	1
4	769	0	Placebo	1	30	4/5/2015	87.5	HEIGHT	10.1	14.5		1	1
4	769	24	Placebo	1	57	4/30/2017	101	HEIGHT	12.81818182	14.5		0	1
4	769	36	Placebo	1	70	6/13/2018	117.9	HEIGHT	14.13636364	13.5		0	1
4	771	12	Placebo	1	42	7/17/2016	91.3	HEIGHT	12.3	14		1	1
4	774	12	Placebo	0	4	7/17/2016	66.6	LENGTH	6.3	12		0	1
4	774	24	Placebo	0	15	4/30/2017	75	HEIGHT	8.909090909	13.5		1	1
4	778	0	Placebo	0	48	4/5/2015	121.2	HEIGHT	21.45	17		0	1
4	778	12	Placebo	0	78	7/17/2016	128.2	HEIGHT	24.2	16.5		0	1
4	778	48	Placebo	0	115	4/27/2019	144	HEIGHT	34	18.5		0	1
4	779	0	Placebo	0	48	4/5/2015	100.6	HEIGHT	17.3	17.5		1	1
4	779	12	Placebo	0	60	7/17/2016	109	HEIGHT	19.8	17.5		0	1
4	779	24	Placebo	0	73	4/30/2017	114.2	HEIGHT	20.68181818	17		0	1
4	779	36	Placebo	0	86	6/13/2018	119.4	HEIGHT	23.63636364	17.5		0	1
4	779	48	Placebo	0	97	4/27/2019	123.7	HEIGHT	24.95	17.5		0	1
4	779	60	Placebo	0	106	2/19/2020	127.1	HEIGHT	26.95454545	18		0	1
4	781	0	Placebo	0	54	4/5/2015	118.9	HEIGHT	21.8	16.5		0	1
4	781	12	Placebo	0	90	7/17/2016	125.1	HEIGHT	23.85	17		0	1
4	781	48	Placebo	0	127	4/27/2019	137.6	HEIGHT	31.25	19		0	1
4	784	24	Placebo	1	14	4/30/2017	71	HEIGHT	7.272727273	14.5		1	1
4	784	36	Placebo	1	27	6/13/2018	80.6	HEIGHT	9.318181818	16		1	1
4	784	60	Placebo	1	47	2/19/2020	93.8	HEIGHT	13	15.3		1	1
4	786	12	Placebo	0	54	7/17/2016	102.5	HEIGHT	14.6	14		1	1
4	787	0	Placebo	0	36	4/5/2015	83.8	HEIGHT	10.5	13.5		0	1
4	787	12	Placebo	0	46	7/17/2016	90.7	HEIGHT	12.3	14.5		0	1

4	787	24	Placebo	0	55	4/30/2017	96.2	HEIGHT	13.31818182	13.5		0	1
4	787	48	Placebo	0	79	4/27/2019	107.4	HEIGHT	17	15.5		0	1
4	787	60	Placebo	0	88	2/19/2020	111.5	HEIGHT	18.18181818	14.9		0	1
4	791	0	Placebo	1	48	4/5/2015	94.7	HEIGHT	13.05	16.5		0	1
4	791	12	Placebo	1	66	7/17/2016	101.3	HEIGHT	15.05	16		0	1
4	791	24	Placebo	1	62	4/30/2017	107.3	HEIGHT	15.81818182	15.5		0	1
4	791	48	Placebo	1	86	4/27/2019	120	HEIGHT	19.3	16		0	1
4	791	60	Placebo	1	96	2/19/2020	124.6	HEIGHT	22	16.4		0	1
4	793	12	Placebo	0	7	7/17/2016	71.3	LENGTH	7.7	14	42	0	1
4	793	24	Placebo	0	15	4/30/2017	77.5	HEIGHT	9.545454545	14	42	0	1
4	793	36	Placebo	0	33	6/13/2018	85	HEIGHT	10.36363636	15.5	42	0	1
4	797	24	Placebo	1	45	4/30/2017	96.5	HEIGHT	15.36363636	15		1	1
4	798	12	Placebo	0	3	7/17/2016	67.8	LENGTH	8.5	14.5		0	1
4	798	48	Placebo	0	39	4/27/2019	91.8	HEIGHT	14.25	15.5		1	1
4	798	60	Placebo	0	48	2/19/2020	99.1	HEIGHT	16.13636364	15		1	1
4	799	24	Placebo	1	43	4/30/2017	92.8	HEIGHT	12.09090909	14		1	1
4	801	0	Placebo	1	30	4/5/2015	81.6	HEIGHT	10.3	15		1	1
4	801	12	Placebo	1	45	7/17/2016	89.8	HEIGHT	12.15	15		1	1
4	801	24	Placebo	1	48	4/30/2017	94.5	HEIGHT	12.86363636	14.5		0	1
4	802	0	Placebo	1	54	4/5/2015	94.9	HEIGHT	13.35	15		1	1
4	802	12	Placebo	1	42	7/17/2016	102.9	HEIGHT	14.7	14		0	1
4	802	24	Placebo	1	55	4/30/2017	109.7	HEIGHT	15.72727273	14.5		0	1
4	802	36	Placebo	1	68	6/13/2018	119	HEIGHT	19.45454545	15.5		0	1
4	802	48	Placebo	1	79	4/27/2019	123.8	HEIGHT	21.95	15.5		0	1
4	802	60	Placebo	1	88	2/19/2020	127.7	HEIGHT	24.36363636	16.1		0	1
4	803	24	Placebo	1	35	4/30/2017	85.6	HEIGHT	12.04545455	16	42	1	1
4	804	0	Placebo	0	7	4/5/2015	73.1	LENGTH	8.25	14.5		1	1
4	804	12	Placebo	0	20	7/17/2016	85.7	HEIGHT	10.9	13.5		0	1
4	804	24	Placebo	0	33	4/30/2017	92.5	HEIGHT	12.59090909	14		0	1
4	804	48	Placebo	0	57	4/27/2019	106.5	HEIGHT	15.4	14		1	1
4	804	60	Placebo	0	66	2/19/2020	111.2	HEIGHT	17.27272727	13.8		0	1
4	806	12	Placebo	0	54	7/17/2016	118	HEIGHT	18.35	15		1	1
4	808	0	Placebo	0	36	4/5/2015	93.9	HEIGHT	15.4	16		1	1
4	808	24	Placebo	0	64	4/30/2017	106.7	HEIGHT	17.27272727	14.5		0	1
4	811	0	Placebo	1	36	4/5/2015	94.8	HEIGHT	14.75	15		0	1
4	811	12	Placebo	1	42	7/17/2016	104.2	HEIGHT	17.65	16.5		0	1
4	811	36	Placebo	1	68	6/13/2018	116.1	HEIGHT	20.95454545	16.5		0	1
4	811	60	Placebo	1	88	2/19/2020	125.4	HEIGHT	25.45454545	16.9		0	1
4	813	0	Placebo	0	36	4/5/2015	99.5	HEIGHT	13.95	14.5		1	1
4	813	12	Placebo	0	50	7/17/2016	106.5	HEIGHT	15.35	14.5		0	1

4	813	24	Placebo	0	67	4/30/2017	111.9	HEIGHT	16.36363636	14	0	1
4	813	60	Placebo	0	100	2/19/2020	124.9	HEIGHT	23	16.5	0	1
4	814	12	Placebo	0	6	7/17/2016	69	LENGTH	7.45	14.5	0	1
4	814	24	Placebo	0	20	4/30/2017	77.3	HEIGHT	8.727272727	13	0	1
4	814	36	Placebo	0	34	6/13/2018	84.2	HEIGHT	11.45454545	15	1	1
4	814	48	Placebo	0	44	4/27/2019	89.2	HEIGHT	12.05	14.5	1	1
4	815	12	Placebo	1	12	7/17/2016	71.3	HEIGHT	7.55	12.5	1	1
4	815	36	Placebo	1	40	6/13/2018	85.8	HEIGHT	11.5	16	0	1
4	815	48	Placebo	1	51	4/27/2019	90.8	HEIGHT	12.55	14.5	1	1
4	815	60	Placebo	1	60	2/19/2020	97.3	HEIGHT	14.5	15	0	1
4	816	12	Placebo	0	24	7/17/2016	57.1	LENGTH	4.1	9.5	1	1
4	816	36	Placebo	0	25	6/13/2018	73.4	LENGTH	7.136363636	13	1	1
4	817	0	Placebo	1	48	4/5/2015	97.6	HEIGHT	14.45	15.5	0	1
4	817	12	Placebo	1	62	7/17/2016	105.5	HEIGHT	16.25	15.5	0	1
4	817	24	Placebo	1	75	4/30/2017	109.5	HEIGHT	17.13636364	15.5	0	1
4	817	48	Placebo	1	99	4/27/2019	120	HEIGHT	21.95	17	0	1
4	818	12	Placebo	0	50	7/17/2016	100.9	HEIGHT	14.4	15	1	1
4	819	12	Placebo	1	8	7/17/2016	73.8	LENGTH	9.45	14	1	1
4	819	60	Placebo	1	49	2/19/2020	106.2	HEIGHT	17.22727273	13.2	0	1
4	820	12	Placebo	0	42	7/17/2016	91.7	HEIGHT	14.1	17	1	1
4	820	24	Placebo	0	55	4/30/2017	96.2	HEIGHT	14.54545455	14.5	1	1
4	821	0	Placebo	0	48	4/5/2015	97.4	HEIGHT	11.95	15	0	1
4	821	12	Placebo	0	42	7/17/2016	98.9	HEIGHT	14.45	15	0	1
4	821	24	Placebo	0	62	4/30/2017	103.2	HEIGHT	15.18181818	14.5	0	1
4	821	48	Placebo	0	86	4/27/2019	114	HEIGHT	17.45	14	0	1
4	821	60	Placebo	0	96	2/19/2020	119	HEIGHT	20.77272727	15.4	0	1
4	822	0	Placebo	0	24	4/5/2015	76.4	LENGTH	8.9	13.5	1	1
4	822	12	Placebo	0	38	7/17/2016	85.6	HEIGHT	11.35	15	1	1
4	822	48	Placebo	0	51	4/27/2019	104.6	HEIGHT	15.25	13.5	0	1
4	822	60	Placebo	0	60	2/19/2020	109.1	HEIGHT	16.36363636	13.7	0	1
4	823	12	Placebo	1	2	7/17/2016	54.6	LENGTH	5.05	14	0	1
4	823	36	Placebo	1	23	6/13/2018	76	LENGTH	7.818181818	14	1	1
4	823	60	Placebo	1	44	2/19/2020	90.5	HEIGHT	12.18181818	14.6	1	1
4	824	0	Placebo	0	24	4/5/2015	84.6	HEIGHT	10.55	14	0	1
4	824	24	Placebo	0	51	4/30/2017	96.1	HEIGHT	12.81818182	14.5	0	1
4	824	48	Placebo	0	75	4/27/2019	111.7	HEIGHT	16.8	15	0	1
4	824	60	Placebo	0	84	2/19/2020	118	HEIGHT	18.09090909	14.5	0	1
4	825	0	Placebo	1	24	4/5/2015	81.7	HEIGHT	10.55	15.5	1	1
4	825	12	Placebo	1	30	7/17/2016	94.3	HEIGHT	12.7	15	1	1
4	825	36	Placebo	1	56	6/13/2018	109.2	HEIGHT	16.40909091	15.5	0	1

4	825	48	Placebo	1	67	4/27/2019	115.1	HEIGHT	17.5	15		0	1
4	825	60	Placebo	1	76	2/19/2020	119.7	HEIGHT	18.90909091	15		0	1
4	828	0	Placebo	1	48	4/5/2015	92.9	HEIGHT	13.05	15.5		0	1
4	828	12	Placebo	1	60	7/17/2016	101	HEIGHT	14.9	16		0	1
4	828	36	Placebo	1	75	6/13/2018	103.7	HEIGHT	17.40909091	15.5		0	1
4	828	48	Placebo	1	86	4/27/2019	118.2	HEIGHT	19.35	15.5		0	1
4	831	24	Placebo	1	52	4/30/2017	93.7	HEIGHT	12.31818182	13.5		1	1
4	832	0	Placebo	1	12	4/5/2015	77.2	HEIGHT	8.9	13		0	1
4	832	12	Placebo	1	30	7/17/2016	89.1	HEIGHT	11.45	14		0	1
4	832	24	Placebo	1	40	4/30/2017	95.5	HEIGHT	13.31818182	13		1	1
4	832	36	Placebo	1	53	6/13/2018	105	HEIGHT	15.54545455	14		0	1
4	832	48	Placebo	1	64	4/27/2019	111.2	HEIGHT	16.6	14		0	1
4	833	0	Placebo	0	24	4/5/2015	88	HEIGHT	12.95	16		1	1
4	833	12	Placebo	0	38	7/17/2016	96	HEIGHT	15.5	16		0	1
4	833	24	Placebo	0	51	4/30/2017	101.2	HEIGHT	16	15		0	1
4	833	36	Placebo	0	64	6/13/2018	107.7	HEIGHT	18.40909091	15.5		0	1
4	833	48	Placebo	0	75	4/27/2019	112.3	HEIGHT	19.45	15		0	1
4	833	60	Placebo	0	84	2/19/2020	117.1	HEIGHT	21.77272727	16		0	1
4	837	0	Placebo	0	24	4/5/2015	81.2	HEIGHT	9.45	12.5		1	1
4	837	12	Placebo	0	39	7/17/2016	93	HEIGHT	12.45	14		0	1
4	837	60	Placebo	0	86	2/19/2020	117.7	HEIGHT	18.63636364	14.1		0	1
4	838	12	Placebo	0	8	7/17/2016	70.9	LENGTH	8.25	14.5		0	1
4	838	24	Placebo	0	15	4/30/2017	79.3	HEIGHT	9.863636364	14		0	1
4	840	0	Placebo	1	12	4/5/2015	71.7	HEIGHT	8.15	13.5	18	0	1
4	840	12	Placebo	1	26	7/17/2016	79.2	HEIGHT	10.2	15	18	1	1
4	842	24	Placebo	0	43	4/30/2017	89.6	HEIGHT	13.04545455	15.5		1	1
4	843	24	Placebo	1	39	4/30/2017	93.8	HEIGHT	12.86363636	14		1	1
4	843	36	Placebo	1	52	6/13/2018	100.3	HEIGHT	14.77272727	15		1	1
4	844	0	Placebo	1	36	4/5/2015	93.4	HEIGHT	13.55	15.5		1	1
4	844	12	Placebo	1	54	7/17/2016	102.4	HEIGHT	15.65	16		0	1
4	844	24	Placebo	1	67	4/30/2017	107.3	HEIGHT	17.27272727	16		0	1
4	844	36	Placebo	1	80	6/13/2018	114.6	HEIGHT	18.40909091	15		0	1
4	844	48	Placebo	1	91	4/27/2019	118.8	HEIGHT	20.35	16		0	1
4	844	60	Placebo	1	100	2/19/2020	122.8	HEIGHT	20.95454545	15.5		0	1
4	849	12	Placebo	0	8	7/17/2016	74.7	LENGTH	9.4	14		1	1
4	849	36	Placebo	0	35	6/13/2018	89.6	HEIGHT	14.59090909	16.5		0	1
4	849	48	Placebo	0	45	4/27/2019	96.7	HEIGHT	16.25	16		0	1
4	849	60	Placebo	0	55	2/19/2020	103.2	HEIGHT	18.18181818	15.3		1	1
4	854	24	Placebo	1	24	4/30/2017	81.2	HEIGHT	10.40909091	14.5		1	1
4	854	48	Placebo	1	51	4/27/2019	100.4	HEIGHT	14.9	15		1	1

4	855	12	Placebo	1	42	7/17/2016	90.8	HEIGHT	12	15	1	1
4	855	24	Placebo	1	51	4/30/2017	96.6	HEIGHT	13.13636364	14.5	1	1
4	856	0	Placebo	1	54	4/5/2015	106.6	HEIGHT	14.65	14.5	1	1
4	856	12	Placebo	1	60	7/17/2016	113.7	HEIGHT	17	14.5	0	1
4	856	24	Placebo	1	81	4/30/2017	117.8	HEIGHT	18.63636364	15	0	1
4	856	36	Placebo	1	95	6/13/2018	123.2	HEIGHT	20.04545455	15.5	0	1
4	856	60	Placebo	1	115	2/19/2020	130.7	HEIGHT	24.09090909	15.9	0	1
4	857	0	Placebo	0	36	4/5/2015	83.2	HEIGHT	11.95	16	1	1
4	857	12	Placebo	0	42	7/17/2016	93.5	HEIGHT	13.3	16	0	1
4	857	48	Placebo	0	79	4/27/2019	111.8	HEIGHT	17.35	15.5	0	1
4	857	60	Placebo	0	88	2/19/2020	116.4	HEIGHT	19.81818182	15.5	0	1
4	858	12	Placebo	0	-1	7/17/2016	60.4	LENGTH	5.65	12.5	0	1
4	858	24	Placebo	0	12	4/30/2017	70.9	HEIGHT	7.818181818	13	0	1
4	858	60	Placebo	0	45	2/19/2020	96.6	HEIGHT	13.13636364	13.9	1	1
4	859	0	Placebo	1	54	4/5/2015	109	HEIGHT	17.25	16	0	1
4	861	0	Placebo	1	48	4/5/2015	116	HEIGHT	22	17.5	1	1
4	862	0	Placebo	0	48	4/5/2015	101.6	HEIGHT	14.55	14	0	1
4	862	48	Placebo	0	99	4/27/2019	121.3	HEIGHT	20.15	14.5	0	1
4	862	60	Placebo	0	108	2/19/2020	125.1	HEIGHT	22.86363636	15	0	1
4	864	0	Placebo	0	30	4/5/2015	86	HEIGHT	12.2	15	0	1
4	864	12	Placebo	0	42	7/17/2016	94.9	HEIGHT	13.45	15	1	1
4	864	24	Placebo	0	55	4/30/2017	98.9	HEIGHT	14.59090909	15	0	1
4	864	36	Placebo	0	68	6/13/2018	105.3	HEIGHT	16.54545455	16	0	1
4	864	48	Placebo	0	79	4/27/2019	110.1	HEIGHT	17.7	16	0	1
4	864	60	Placebo	0	88	2/19/2020	114.8	HEIGHT	19.5	16	0	1
4	865	24	Placebo	1	39	4/30/2017	86.7	HEIGHT	11.40909091	14	1	1
4	869	0	Placebo	1	10	4/5/2015	75.4	HEIGHT	8.5	14.5	0	1
4	869	12	Placebo	1	30	7/17/2016	85	HEIGHT	10.95	15	0	1
4	869	48	Placebo	1	67	4/27/2019	103.7	HEIGHT	16.5	15.5	0	1
4	869	60	Placebo	1	76	2/19/2020	108.1	HEIGHT	18.27272727	14.8	0	1
4	870	0	Placebo	1	59	4/5/2015	117.3	HEIGHT	19.7	17.5	0	1
4	870	12	Placebo	1	90	7/17/2016	123.7	HEIGHT	21.9	17.5	0	1
4	870	48	Placebo	1	127	4/27/2019	136.4	HEIGHT	28.2	19	0	1
4	870	60	Placebo	1	136	2/19/2020	140.8	HEIGHT	30.45454545	19.5	0	1
4	872	0	Placebo	0	1	4/5/2015	65	LENGTH	6.75	13	1	1
4	872	24	Placebo	0	27	4/30/2017	83	HEIGHT	11.59090909	15	1	1
4	872	36	Placebo	0	40	6/13/2018	93.7	HEIGHT	15.18181818	17	0	1
4	873	12	Placebo	1	10	7/17/2016	76.6	HEIGHT	8.5	14	0	1
4	873	24	Placebo	1	20	4/30/2017	82.8	HEIGHT	10.04545455	14	0	1
4	873	36	Placebo	1	33	6/13/2018	93.6	HEIGHT	12.04545455	14.5	0	1

4	874	24	Placebo	0	31	4/30/2017	77.2	HEIGHT	9.136363636	13.5		1	1
4	874	48	Placebo	0	55	4/27/2019	88.8	HEIGHT	11.55	14		1	1
4	875	12	Placebo	1	-2	7/17/2016	56.6	LENGTH	5.25	13.5		0	1
4	875	24	Placebo	1	11	4/30/2017	69.6	LENGTH	7.454545455	14		0	1
4	877	12	Placebo	1	4	7/17/2016	64.9	LENGTH	6.85	13.5		0	1
4	877	36	Placebo	1	29	6/13/2018	80.3	HEIGHT	10.63636364	15.5		0	1
4	877	60	Placebo	1	49	2/19/2020	92.4	HEIGHT	13	14.4		0	1
4	880	0	Placebo	1	36	4/5/2015	99.1	HEIGHT	13.4	14		1	1
4	880	12	Placebo	1	66	7/17/2016	105.3	HEIGHT	15.05	14.5		0	1
4	880	24	Placebo	1	79	4/30/2017	109.6	HEIGHT	15.45454545	13.5		0	1
4	880	36	Placebo	1	92	6/13/2018	116.2	HEIGHT	17.5	14.5		0	1
4	880	48	Placebo	1	103	4/27/2019	121.2	HEIGHT	19.05	14.5		0	1
4	880	60	Placebo	1	112	2/19/2020	125.5	HEIGHT	21.36363636	15.3		0	1
4	882	12	Placebo	0	4	7/17/2016	68.9	LENGTH	8.35	13.5		0	1
4	882	36	Placebo	0	31	6/13/2018	84.2	HEIGHT	13.09090909	16		0	1
4	883	12	Placebo	0	2	7/17/2016	66	LENGTH	6.8	13		0	1
4	883	24	Placebo	0	12	4/30/2017	73	HEIGHT	8.318181818	13		0	1
4	883	36	Placebo	0	23	6/13/2018	85	LENGTH	11.09090909	15		0	1
4	884	24	Placebo	1	55	4/30/2017	87.3	HEIGHT	9.727272727	12		1	1
4	885	0	Placebo	1	12	4/5/2015	72.9	LENGTH	7.85	13.5		0	1
4	887	12	Placebo	1	9	7/17/2016	75.8	HEIGHT	8.5	14		0	1
4	887	24	Placebo	1	22	4/30/2017	83.5	HEIGHT	9.818181818	13		0	1
4	887	36	Placebo	1	35	6/13/2018	92.4	HEIGHT	12.90909091	16		0	1
4	887	48	Placebo	1	45	4/27/2019	98.7	HEIGHT	15.15	15.5		1	1
4	887	60	Placebo	1	55	2/19/2020	106.5	HEIGHT	16.68181818	14.6		0	1
4	888	12	Placebo	1	10	7/17/2016	73.6	LENGTH	8.5	13.5	30	0	1
4	888	24	Placebo	1	22	4/30/2017	80	HEIGHT	10.22727273	15	30	0	1
4	889	0	Placebo	1	48	4/5/2015	106.5	HEIGHT	13.6	13.5		1	1
4	889	12	Placebo	1	62	7/17/2016	114.8	HEIGHT	16.4	14.5		0	1
4	889	24	Placebo	1	89	4/30/2017	118.1	HEIGHT	17.90909091	14.5		0	1
4	889	36	Placebo	1	103	6/13/2018	123.5	HEIGHT	19.59090909	15		0	1
4	889	48	Placebo	1	113	4/27/2019	126	HEIGHT	21.1	15.5		0	1
4	889	60	Placebo	1	123	2/19/2020	129.8	HEIGHT	23.13636364	15.5		0	1
4	892	24	Placebo	0	10	4/30/2017	72.3	LENGTH	8.545454545	14		1	1
4	892	48	Placebo	0	34	4/27/2019	85.2	HEIGHT	12.2	14		0	1
4	892	60	Placebo	0	43	2/19/2020	94.8	HEIGHT	14	14.5		0	1
4	893	0	Placebo	1	36	4/5/2015	87.7	HEIGHT	10.25	13		1	1
4	893	12	Placebo	1	42	7/17/2016	98.5	HEIGHT	12.5	13.5		0	1
4	893	24	Placebo	1	69	4/30/2017	104.1	HEIGHT	14.40909091	13.5		0	1
4	894	12	Placebo	0	9	7/17/2016	72.2	LENGTH	8.05	14.5		1	1

4	894	24	Placebo	0	20	4/30/2017	80	HEIGHT	9.636363636	16			0	1
4	894	36	Placebo	0	33	6/13/2018	87.3	HEIGHT	12.22727273	16			0	1
4	894	48	Placebo	0	44	4/27/2019	93.5	HEIGHT	13.7	16			0	1
4	894	60	Placebo	0	54	2/19/2020	101.2	HEIGHT	15.27272727	15.9			0	1
4	896	0	Placebo	0	48	4/5/2015	109	HEIGHT	16.9	15.5			0	1
4	896	12	Placebo	0	78	7/17/2016	117.5	HEIGHT	18.75	15			0	1
4	896	48	Placebo	0	115	4/27/2019	131.5	HEIGHT	23.7	16.5			0	1
4	896	60	Placebo	0	124	2/19/2020	135.2	HEIGHT	26.68181818	17			0	1
4	897	0	Placebo	1	12	4/5/2015	71.1	LENGTH	6.3	12			0	1
4	897	24	Placebo	1	43	4/30/2017	87.9	HEIGHT	10.68181818	13.5			1	1
4	897	60	Placebo	1	72	2/19/2020	107.1	HEIGHT	15.22727273	13.8			0	1
4	898	24	Placebo	0	55	4/30/2017	102.1	HEIGHT	16.45454545	15.5			1	1
4	899	0	Placebo	1	54	4/5/2015	115.5	HEIGHT	18	15			0	1
4	899	12	Placebo	1	66	7/17/2016	123.1	HEIGHT	20.6	15			0	1
4	899	24	Placebo	1	79	4/30/2017	127.9	HEIGHT	22.81818182	16			0	1
4	899	36	Placebo	1	92	6/13/2018	134.6	HEIGHT	25.18181818	16			0	1
4	899	48	Placebo	1	103	4/27/2019	138.4	HEIGHT	27.2	16			0	1
4	899	60	Placebo	1	112	2/19/2020	143	HEIGHT	30.36363636	17.5			0	1
4	900	12	Placebo	0	7	7/17/2016	70	LENGTH	7.25	13.5			1	1
4	900	24	Placebo	0	15	4/30/2017	76.9	HEIGHT	8.818181818	14			0	1
4	900	36	Placebo	0	29	6/13/2018	87.3	HEIGHT	11.09090909	14			0	1
4	900	48	Placebo	0	39	4/27/2019	94.5	HEIGHT	12.9	14.5			0	1
4	900	60	Placebo	0	49	2/19/2020	100.1	HEIGHT	14.68181818	14.1			0	1
4	901	0	Placebo	1	2	4/5/2015	65.3	LENGTH	6.9	13			0	1
4	901	12	Placebo	1	18	7/17/2016	73.6	LENGTH	7.45	13			0	1
4	901	24	Placebo	1	31	4/30/2017	77.9	HEIGHT	10.36363636	13.5			0	1
4	901	36	Placebo	1	44	6/13/2018	87.2	HEIGHT	13.40909091	15			0	1
4	901	48	Placebo	1	55	4/27/2019	97.3	HEIGHT	15.6	15.5			0	1
4	902	0	Placebo	0	24	4/5/2015	89.7	HEIGHT	11.25	15			1	1
4	902	12	Placebo	0	42	7/17/2016	100.6	HEIGHT	13.9	15.5			1	1
4	902	24	Placebo	0	55	4/30/2017	106.3	HEIGHT	15.04545455	14.5			0	1
4	902	36	Placebo	0	68	6/13/2018	114.6	HEIGHT	17.27272727	15			0	1
4	902	48	Placebo	0	79	4/27/2019	109.6	HEIGHT	19.5	16			0	1
4	903	0	Placebo	0	30	4/5/2015	85.2	HEIGHT	10.55	14.5			1	1
4	904	0	Placebo	0	5	4/5/2015	68.1	LENGTH	6.7	12			0	1
4	904	12	Placebo	0	20	7/17/2016	81.6	HEIGHT	9.95	14.5			0	1
4	904	24	Placebo	0	33	4/30/2017	85.6	HEIGHT	11.59090909	16			0	1
4	904	36	Placebo	0	46	6/13/2018	91.6	HEIGHT	13.13636364	16			0	1
4	904	48	Placebo	0	57	4/27/2019	98.6	HEIGHT	15.45	17			1	1
4	905	12	Placebo	0	6	7/17/2016	66.2	LENGTH	7.7	15			1	1

4	905	36	Placebo	0	29	6/13/2018	82.1	HEIGHT	10.95454545	16		0	1
4	905	48	Placebo	0	39	4/27/2019	88.4	HEIGHT	12.3	14		1	1
4	910	24	Placebo	1	10	4/30/2017	69.8	LENGTH	6.727272727	12		1	1
4	910	48	Placebo	1	34	4/27/2019	90	HEIGHT	14.05	16.5		1	1
4	912	24	Placebo	0	24	4/30/2017	79.6	HEIGHT	9	12		1	1
4	914	0	Placebo	0	12	4/5/2015	71	LENGTH	6.9	11.5		0	1
4	915	0	Placebo	0	54	4/5/2015	103.4	HEIGHT	15.45	15		0	1
4	915	12	Placebo	0	60	7/17/2016	109.9	HEIGHT	17.65	15.5		0	1
4	915	36	Placebo	0	86	6/13/2018	119.2	HEIGHT	20.5	16		0	1
4	916	0	Placebo	0	48	4/5/2015	107.7	HEIGHT	16.7	16		0	1
4	916	12	Placebo	0	60	7/17/2016	114.8	HEIGHT	19.2	17		0	1
4	916	24	Placebo	0	73	4/30/2017	117.8	HEIGHT	20.5	17		0	1
4	916	48	Placebo	0	97	4/27/2019	126.2	HEIGHT	23.75	18		0	1
4	917	0	Placebo	0	59	4/5/2015	110.5	HEIGHT	16.7	15		1	1
4	917	12	Placebo	0	90	7/17/2016	116.9	HEIGHT	18.4	15		0	1
4	917	48	Placebo	0	127	4/27/2019	130.7	HEIGHT	24.4	16		0	1
4	917	60	Placebo	0	136	2/19/2020	133.5	HEIGHT	26.04545455	17		0	1
4	918	12	Placebo	1	9	7/17/2016	73.5	LENGTH	8	13.5		0	1
4	918	60	Placebo	1	55	2/19/2020	106.3	HEIGHT	16.27272727	14.5		0	1
4	919	12	Placebo	1	10	7/17/2016	75.9	LENGTH	8.35	13.5	54	0	1
4	919	36	Placebo	1	37	6/13/2018	90.3	HEIGHT	12.77272727	15	54	1	1
4	920	24	Placebo	1	37	4/30/2017	96.4	HEIGHT	13.13636364	14		1	1
4	921	0	Placebo	1	36	4/5/2015	90.9	HEIGHT	12.2	14		0	1
4	921	12	Placebo	1	50	7/17/2016	99	HEIGHT	13.6	13.5		1	1
4	921	24	Placebo	1	63	4/30/2017	104.1	HEIGHT	14.54545455	13.5		0	1
4	922	0	Placebo	0	54	4/5/2015	109.1	HEIGHT	15.75	14.5		0	1
4	922	12	Placebo	0	78	7/17/2016	115.7	HEIGHT	18.1	15		0	1
4	922	36	Placebo	0	104	6/13/2018	125.8	HEIGHT	21.59090909	16		0	1
4	922	60	Placebo	0	124	2/19/2020	132.9	HEIGHT	26.68181818	17		0	1
4	924	0	Placebo	1	24	4/5/2015	79.2	HEIGHT	10.4	15		1	1
4	924	24	Placebo	1	50	4/30/2017	95.5	HEIGHT	14.31818182	15		1	1
4	924	36	Placebo	1	64	6/13/2018	103.5	HEIGHT	15.27272727	15.5		0	1
4	925	0	Placebo	0	54	4/5/2015	110.8	HEIGHT	17.6	15.5		1	1
4	925	36	Placebo	0	93	6/13/2018	123.6	HEIGHT	22.86363636	17		0	1
4	926	0	Placebo	1	3	4/5/2015	64.6	LENGTH	6.95	13.5		1	1
4	926	12	Placebo	1	18	7/17/2016	80.3	LENGTH	10	14.5		0	1
4	926	36	Placebo	1	44	6/13/2018	96.5	HEIGHT	14.04545455	14.5		1	1
4	926	48	Placebo	1	55	4/27/2019	103.3	HEIGHT	15.5	14.5		1	1
4	927	0	Placebo	1	36	4/5/2015	89.2	HEIGHT	13.25	16		0	1
4	927	12	Placebo	1	55	7/17/2016	94.6	HEIGHT	13	14.5		0	1

4	927	24	Placebo	1	64	4/30/2017	100.3	HEIGHT	14	14	0	1
4	927	36	Placebo	1	77	6/13/2018	107.8	HEIGHT	16.36363636	15	0	1
4	927	48	Placebo	1	88	4/27/2019	113.8	HEIGHT	17.7	15	0	1
4	927	60	Placebo	1	98	2/19/2020	119.6	HEIGHT	20.54545455	15.5	0	1
4	929	0	Placebo	1	59	4/5/2015	126.2	HEIGHT	24.1	16.5	0	1
4	929	12	Placebo	1	75	7/17/2016	131.4	HEIGHT	27.2	17.5	0	1
4	930	24	Placebo	0	55	4/30/2017	103.1	HEIGHT	15.27272727	14	1	1
4	932	12	Placebo	1	54	7/17/2016	102.1	HEIGHT	14.3	13.5	1	1
4	933	0	Placebo	1	36	4/5/2015	98.8	HEIGHT	16.6	17	1	1
4	933	12	Placebo	1	50	7/17/2016	107	HEIGHT	17.7	17.5	0	1
4	933	36	Placebo	1	84	6/13/2018	117.5	HEIGHT	20.81818182	18	0	1
4	933	48	Placebo	1	95	4/27/2019	122	HEIGHT	22.45	17.5	0	1
4	933	60	Placebo	1	104	2/19/2020	125.9	HEIGHT	24.90909091	17	0	1
4	934	0	Placebo	1	8	4/5/2015	73.5	LENGTH	8.95	15.5	0	1
4	934	12	Placebo	1	20	7/17/2016	85.3	HEIGHT	12.8	16	0	1
4	935	12	Placebo	0	7	7/17/2016	62.6	LENGTH	6.15	12	0	1
4	936	24	Placebo	0	55	4/30/2017	99.5	HEIGHT	14.95454545	14.5	1	1
4	937	12	Placebo	1	9	7/17/2016	66.8	LENGTH	7	14.5	0	1
4	937	36	Placebo	1	29	6/13/2018	79.3	HEIGHT	9.227272727	14	0	1
4	937	48	Placebo	1	39	4/27/2019	85.3	HEIGHT	10.75	14	1	1
4	937	60	Placebo	1	49	2/19/2020	92.8	HEIGHT	12.5	13.6	1	1
4	940	12	Placebo	0	50	7/17/2016	111	HEIGHT	17.85	15.5	1	1
4	944	0	Placebo	0	54	4/5/2015	106.2	HEIGHT	15.8	15.5	1	1
4	944	12	Placebo	0	68	7/17/2016	112.2	HEIGHT	18.1	15.5	0	1
4	944	24	Placebo	0	81	4/30/2017	116.6	HEIGHT	19.31818182	15.5	0	1
4	944	36	Placebo	0	94	6/13/2018	121.6	HEIGHT	21.72727273	17	0	1
4	944	48	Placebo	0	105	4/27/2019	125.6	HEIGHT	23.5	17	0	1
4	944	60	Placebo	0	114	2/19/2020	129.4	HEIGHT	25.72727273	17	0	1
4	947	12	Placebo	0	56	7/17/2016	99.1	HEIGHT	14.95	16.5	1	1
4	949	0	Placebo	1	59	4/5/2015	114.9	HEIGHT	18.5	16	1	1
4	954	0	Placebo	1	48	4/5/2015	100.3	HEIGHT	13.3	14.5	0	1
4	957	0	Placebo	1	24	4/5/2015	71.6	LENGTH	7.75	14	0	1
4	957	12	Placebo	1	34	7/17/2016	83.4	HEIGHT	10.3	14.5	1	1
4	957	24	Placebo	1	33	4/30/2017	89.4	HEIGHT	11.77272727	14.5	0	1
4	957	36	Placebo	1	46	6/13/2018	97.2	HEIGHT	13.77272727	15	1	1
4	957	48	Placebo	1	57	4/27/2019	101.7	HEIGHT	14.75	14.5	1	1
4	957	60	Placebo	1	66	2/19/2020	106.7	HEIGHT	15.45454545	13.7	0	1
4	958	0	Placebo	0	30	4/5/2015	86.2	HEIGHT	11.85	15.5	1	1
4	958	12	Placebo	0	42	7/17/2016	94.9	HEIGHT	13.45	14.5	0	1
4	958	24	Placebo	0	60	4/30/2017	100.1	HEIGHT	14.81818182	14.5	0	1

4	958	36	Placebo	0	73	6/13/2018	105.6	HEIGHT	16.04545455	14.5		0	1
4	958	48	Placebo	0	84	4/27/2019	112.6	HEIGHT	16.75	14		0	1
4	958	60	Placebo	0	94	2/19/2020	118.3	HEIGHT	18.36363636	13.5		0	1
4	959	0	Placebo	1	12	4/5/2015	73.2	HEIGHT	7.85	14.5		0	1
4	959	12	Placebo	1	26	7/17/2016	81.1	HEIGHT	9.4	13		1	1
4	959	24	Placebo	1	39	4/30/2017	87.1	HEIGHT	10.90909091	14.5		1	1
4	959	36	Placebo	1	52	6/13/2018	95.6	HEIGHT	12.13636364	14		0	1
4	959	48	Placebo	1	63	4/27/2019	100.1	HEIGHT	14.15	14		0	1
4	959	60	Placebo	1	72	2/19/2020	107.4	HEIGHT	15.90909091	14		0	1
4	960	24	Placebo	1	10	4/30/2017	65.5	LENGTH	7.909090909	15		1	1
4	960	36	Placebo	1	21	6/13/2018	77.7	LENGTH	10.5	16		0	1
4	960	48	Placebo	1	31	4/27/2019	83	HEIGHT	12.05	15.5		1	1
4	960	60	Placebo	1	41	2/19/2020	90.6	HEIGHT	13.27272727	15.4		0	1
4	961	0	Placebo	1	59	4/5/2015	113.1	HEIGHT	16.55	14.5		0	1
4	961	12	Placebo	1	75	7/17/2016	119	HEIGHT	18.05	14.5		0	1
4	961	36	Placebo	1	101	6/13/2018	127.4	HEIGHT	22.27272727	15.5		0	1
4	961	48	Placebo	1	112	4/27/2019	130.7	HEIGHT	23.55	16		0	1
4	961	60	Placebo	1	122	2/19/2020	135.2	HEIGHT	26.59090909	16.5		0	1
4	963	24	Placebo	0	6	4/30/2017	72.4	LENGTH	9.181818182	15.5		1	1
4	964	0	Placebo	1	10	4/5/2015	70.3	LENGTH	9.1	15.5		0	1
4	964	12	Placebo	1	20	7/17/2016	81.8	HEIGHT	10.95	14.5		0	1
4	964	24	Placebo	1	33	4/30/2017	87.2	HEIGHT	12.63636364	14.5		0	1
4	964	48	Placebo	1	57	4/27/2019	101.6	HEIGHT	16.85	16.5		1	1
4	964	60	Placebo	1	66	2/19/2020	107.2	HEIGHT	16.63636364	15		0	1
4	965	0	Placebo	1	54	4/5/2015	109.9	HEIGHT	15.4	14		1	1
4	965	12	Placebo	1	68	7/17/2016	114.5	HEIGHT	17.15	14.5		0	1
4	965	36	Placebo	1	113	6/13/2018	123.5	HEIGHT	20.13636364	15		0	1
4	965	48	Placebo	1	123	4/27/2019	127	HEIGHT	21.45	15.5		0	1
4	966	0	Placebo	1	36	4/5/2015	93.7	HEIGHT	11.3	13		0	1
4	966	12	Placebo	1	36	7/17/2016	101.5	HEIGHT	12.85	13.5		0	1
4	966	24	Placebo	1	49	4/30/2017	108.1	HEIGHT	14.90909091	14		0	1
4	966	48	Placebo	1	73	4/27/2019	119.9	HEIGHT	18.7	14		0	1
4	966	60	Placebo	1	82	2/19/2020	125.8	HEIGHT	22.63636364	15.8		0	1
4	967	24	Placebo	1	12	4/30/2017	77.8	LENGTH	10.22727273	15		1	1
4	967	48	Placebo	1	41	4/27/2019	94.1	HEIGHT	15.6	16		1	1
4	968	0	Placebo	1	36	4/5/2015	90.9	HEIGHT	12.25	15		0	1
4	970	12	Placebo	1	2	7/17/2016	53.4	LENGTH	4.85	13		0	1
4	970	36	Placebo	1	23	6/13/2018	78.5	HEIGHT	9	14		0	1
4	970	60	Placebo	1	44	2/19/2020	92.6	HEIGHT	12.86363636	15		0	1
4	971	0	Placebo	0	7	4/5/2015	77	HEIGHT	8.75	14.5	42	0	1

4	971	12	Placebo	0	24	7/17/2016	82.3	HEIGHT	9.85	13.5	42	0	1
4	973	0	Placebo	0	54	4/5/2015	117.3	HEIGHT	16.9	14		0	1
4	973	12	Placebo	0	60	7/17/2016	123.6	HEIGHT	18.85	14.5		0	1
4	973	24	Placebo	0	73	4/30/2017	128.1	HEIGHT	21.04545455	15.5		0	1
4	973	60	Placebo	0	106	2/19/2020	139.8	HEIGHT	28.81818182	17		0	1
4	974	0	Placebo	1	48	4/5/2015	94.8	HEIGHT	13.9	16		1	1
4	974	12	Placebo	1	66	7/17/2016	102.4	HEIGHT	16.05	16.5		0	1
4	974	36	Placebo	1	88	6/13/2018	114.5	HEIGHT	19.72727273	16.5		0	1
4	974	48	Placebo	1	99	4/27/2019	119.9	HEIGHT	20.8	17		0	1
4	974	60	Placebo	1	108	2/19/2020	122.5	HEIGHT	23.59090909	16.5		0	1
4	975	0	Placebo	1	36	4/5/2015	98.7	HEIGHT	14	15		0	1
4	975	12	Placebo	1	50	7/17/2016	107.2	HEIGHT	16.05	14.5		0	1
4	975	60	Placebo	1	93	2/19/2020	128.4	HEIGHT	25.36363636	16.5		0	1
4	977	0	Placebo	0	36	4/5/2015	111.6	HEIGHT	16.85	15.5		0	1
4	977	12	Placebo	0	50	7/17/2016	117.8	HEIGHT	18.4	15		0	1
4	977	24	Placebo	0	63	4/30/2017	120.5	HEIGHT	19.77272727	15		0	1
4	978	0	Placebo	1	30	4/5/2015	82	HEIGHT	12.05	16		0	1
4	978	12	Placebo	1	42	7/17/2016	92.5	HEIGHT	13.55	15.5		0	1
4	978	24	Placebo	1	55	4/30/2017	99.7	HEIGHT	14.68181818	15		0	1
4	978	36	Placebo	1	68	6/13/2018	110.6	HEIGHT	15.77272727	15		0	1
4	978	48	Placebo	1	79	4/27/2019	113.5	HEIGHT	18.65	15.5		0	1
4	978	60	Placebo	1	88	2/19/2020	119.2	HEIGHT	21.22727273	16.5		0	1
4	979	0	Placebo	0	9	4/5/2015	76.2	HEIGHT	8.25	13.5		1	1
4	979	24	Placebo	0	33	4/30/2017	93.8	HEIGHT	13.09090909	14		1	1
4	979	36	Placebo	0	46	6/13/2018	101.4	HEIGHT	15.13636364	15.5		1	1
4	979	48	Placebo	0	57	4/27/2019	108.2	HEIGHT	16.25	15		0	1
4	979	60	Placebo	0	56	2/19/2020	113.7	HEIGHT	18.18181818	14.4		0	1
4	981	0	Placebo	0	54	4/5/2015	99.1	HEIGHT	14.85	16		0	1
4	981	12	Placebo	0	54	7/17/2016	106.7	HEIGHT	17.2	16		0	1
4	981	24	Placebo	0	67	4/30/2017	110.6	HEIGHT	17.81818182	15		0	1
4	981	36	Placebo	0	80	6/13/2018	116.4	HEIGHT	20.36363636	17		0	1
4	981	48	Placebo	0	91	4/27/2019	120.3	HEIGHT	22.2	17		0	1
4	981	60	Placebo	0	100	2/19/2020	125	HEIGHT	24.18181818	17		0	1
4	982	24	Placebo	0	15	4/30/2017	76.9	LENGTH	8.409090909	13		1	1
4	987	12	Placebo	0	10	7/17/2016	72.5	LENGTH	8.15	15		0	1
4	987	24	Placebo	0	22	4/30/2017	81.2	HEIGHT	10.59090909	14		0	1
4	987	36	Placebo	0	35	6/13/2018	89.8	HEIGHT	13.59090909	17		1	1
4	987	48	Placebo	0	45	4/27/2019	95.1	HEIGHT	15.55	16		1	1
4	987	60	Placebo	0	55	2/19/2020	102.4	HEIGHT	17.5	15		0	1
4	990	12	Placebo	0	54	7/17/2016	103.2	HEIGHT	17	15.5		1	1

4	993	12	Placebo	1	42	7/17/2016	100.7	HEIGHT	16.4	17		1	1
4	995	24	Placebo	0	55	4/30/2017	100.2	HEIGHT	15.13636364	16.5		1	1
4	996	24	Placebo	0	2	4/30/2017	60.9	LENGTH	6.409090909	14		1	1
4	998	0	Placebo	1	54	4/5/2015	107.6	HEIGHT	14.55	14.5	24	1	1
4	999	12	Placebo	1	12	7/17/2016	74.1	LENGTH	8.2	13		1	1
4	999	24	Placebo	1	26	4/30/2017	80.5	HEIGHT	10.13636364	13.5		0	1
4	999	36	Placebo	1	39	6/13/2018	89.5	HEIGHT	12.68181818	16		0	1
4	999	48	Placebo	1	50	4/27/2019	96.9	HEIGHT	14.1	15		0	1
4	999	60	Placebo	1	60	2/19/2020	103.3	HEIGHT	16.04545455	15		0	1
4	1000	0	Placebo	1	36	4/5/2015	88.2	HEIGHT	12.5	15.5		1	1
4	1000	12	Placebo	1	42	7/17/2016	97.5	HEIGHT	13.9	14.5		0	1
4	1003	12	Placebo	1	26	7/17/2016	87.9	HEIGHT	13.45	17		1	1
4	1008	12	Placebo	0	1	7/17/2016	66.6	LENGTH	7.2	14		0	1
4	1008	36	Placebo	0	26	6/13/2018	82.7	HEIGHT	10.81818182	15		0	1
4	1011	12	Placebo	0	26	7/17/2016	86.4	HEIGHT	11.6	14	30	1	1
4	1012	0	Placebo	1	36	4/5/2015	86.3	HEIGHT	12.15	15.5		0	1
4	1012	24	Placebo	1	63	4/30/2017	101.6	HEIGHT	15.59090909	15		0	1
4	1012	36	Placebo	1	76	6/13/2018	107.3	HEIGHT	16.5	15.5		0	1
4	1012	48	Placebo	1	87	4/27/2019	112.7	HEIGHT	18.1	14.5		0	1
4	1012	60	Placebo	1	96	2/19/2020	117.4	HEIGHT	21.27272727	15		0	1
4	1014	12	Placebo	0	42	7/17/2016	94.8	HEIGHT	12.45	13.5		1	1
4	1015	12	Placebo	1	9	7/17/2016	73.7	HEIGHT	8.65	14.5		0	1
4	1015	36	Placebo	1	37	6/13/2018	88.6	HEIGHT	12.22727273	15.5		1	1
4	1015	48	Placebo	1	47	4/27/2019	94.3	HEIGHT	14.35	16		1	1
4	1017	0	Placebo	0	36	4/5/2015	84.4	HEIGHT	11.95	16		0	1
4	1018	12	Placebo	0	30	7/17/2016	83.2	HEIGHT	11.05	15.5		1	1
4	1019	0	Placebo	0	24	4/5/2015	86	HEIGHT	10.35	14		1	1
4	1022	12	Placebo	0	42	7/17/2016	95.5	HEIGHT	13.25	14.5		1	1
4	1022	24	Placebo	0	51	4/30/2017	100.3	HEIGHT	14.13636364	14		1	1
4	1024	24	Placebo	0	9	4/30/2017	67.6	LENGTH	6	12	30	1	1
4	1025	24	Placebo	0	51	4/30/2017	103.7	HEIGHT	16.5	17		1	1
4	1026	0	Placebo	1	24	4/5/2015	82.9	HEIGHT	10.6	13.5		0	1
4	1026	60	Placebo	1	100	2/19/2020	117	HEIGHT	18.63636364	14.2		0	1
4	1028	24	Placebo	1	25	4/30/2017	84.7	HEIGHT	13.27272727	17		1	1
4	1028	36	Placebo	1	39	6/13/2018	96.5	HEIGHT	15.77272727	17		1	1
4	1029	0	Placebo	1	24	4/5/2015	76.9	HEIGHT	8.05	13		0	1
4	1029	12	Placebo	1	38	7/17/2016	84.5	HEIGHT	10.75	13		0	1
4	1029	24	Placebo	1	47	4/30/2017	93	HEIGHT	12.81818182	15		0	1
4	1029	36	Placebo	1	60	6/13/2018	99.3	HEIGHT	13.77272727	14		0	1
4	1029	48	Placebo	1	69	4/27/2019	104.7	HEIGHT	15.1	14		0	1

4	1037	12	Placebo	0	-2	7/17/2016	56.1	LENGTH	5.9	14		0	1
4	1037	24	Placebo	0	11	4/30/2017	73.6	LENGTH	10.81818182	16.5		1	1
4	1037	36	Placebo	0	23	6/13/2018	82.4	LENGTH	11.36363636	14		0	1
4	1037	48	Placebo	0	34	4/27/2019	86.2	HEIGHT	13.5	17		0	1
4	1037	60	Placebo	0	43	2/19/2020	91.9	HEIGHT	15.09090909	15.5		0	1
4	1040	0	Placebo	1	54	4/5/2015	114.9	HEIGHT	20.15	16.5		0	1
4	1040	12	Placebo	1	78	7/17/2016	121.9	HEIGHT	22.2	17.5		0	1
4	1040	36	Placebo	1	104	6/13/2018	129.6	HEIGHT	26.22727273	18		0	1
4	1040	48	Placebo	1	115	4/27/2019	133.7	HEIGHT	27.4	18.5		0	1
4	1040	60	Placebo	1	124	2/19/2020	135.3	HEIGHT	29.63636364	18.8		0	1
4	1041	24	Placebo	1	55	4/30/2017	110.3	HEIGHT	17.18181818	15		1	1
4	1042	0	Placebo	0	36	4/5/2015	115.7	HEIGHT	17.6	14.5		0	1
4	1042	12	Placebo	0	54	7/17/2016	122.3	HEIGHT	19.55	15		0	1
4	1042	24	Placebo	0	68	4/30/2017	126.3	HEIGHT	21.04545455	15		0	1
4	1042	48	Placebo	0	92	4/27/2019	134	HEIGHT	25	17.5		0	1
4	1042	60	Placebo	0	102	2/19/2020	137.6	HEIGHT	26.72727273	17.3		0	1
4	1044	0	Placebo	1	24	4/5/2015	89	HEIGHT	12.15	15		1	1
4	1044	12	Placebo	1	42	7/17/2016	97.4	HEIGHT	13.9	14.5		0	1
4	1044	36	Placebo	1	68	6/13/2018	112.5	HEIGHT	17.86363636	15		0	1
4	1044	48	Placebo	1	79	4/27/2019	119	HEIGHT	20.5	16		0	1
4	1044	60	Placebo	1	88	2/19/2020	125	HEIGHT	22.45454545	16		0	1
4	1045	0	Placebo	1	48	4/5/2015	111.9	HEIGHT	17.65	16		0	1
4	1045	12	Placebo	1	48	7/17/2016	117.2	HEIGHT	19.25	16.5		1	1
4	1045	24	Placebo	1	61	4/30/2017	119.9	HEIGHT	20.09090909	16.5		0	1
4	1045	48	Placebo	1	85	4/27/2019	128.9	HEIGHT	24.5	18		0	1
4	1048	24	Placebo	0	19	4/30/2017	71.6	LENGTH	7.045454545	11.5		1	1
4	1048	36	Placebo	0	32	6/13/2018	80.5	HEIGHT	9.363636364	14		1	1
4	1050	0	Placebo	0	48	4/5/2015	104.4	HEIGHT	15.75	14.5		0	1
4	1050	12	Placebo	0	78	7/17/2016	112.4	HEIGHT	18.25	15.5		0	1
4	1051	12	Placebo	0	42	7/17/2016	99.9	HEIGHT	13.3	13.5		1	1
4	1052	0	Placebo	0	48	4/5/2015	106.5	HEIGHT	15.55	14		1	1
4	1053	12	Placebo	0	8	7/17/2016	75	HEIGHT	8.3	13.5	18	0	1
4	1055	0	Placebo	0	54	4/5/2015	112.2	HEIGHT	18.1	15.5		1	1
4	1055	12	Placebo	0	71	7/17/2016	120	HEIGHT	21.7	16		0	1
4	1058	12	Placebo	0	3	7/17/2016	70.5	LENGTH	7.7	13.5		0	1
4	1058	36	Placebo	0	28	6/13/2018	88.3	LENGTH	11.45454545	15		1	1
4	1058	60	Placebo	0	48	2/19/2020	100.1	HEIGHT	15.31818182	15.2		0	1
4	1060	24	Placebo	0	15	4/30/2017	76.9	HEIGHT	8.136363636	11.5		0	1
4	1060	36	Placebo	0	29	6/13/2018	85.9	HEIGHT	10.68181818	14.5		0	1
4	1060	60	Placebo	0	49	2/19/2020	98.8	HEIGHT	13.5	13.5		0	1

4	1062	0	Placebo	1	12	4/5/2015	77.8	HEIGHT	8.65	13.5	0	1
4	1062	24	Placebo	1	39	4/30/2017	96.6	HEIGHT	13.5	14.5	0	1
4	1062	36	Placebo	1	52	6/13/2018	105.1	HEIGHT	15.40909091	15.5	0	1
4	1063	0	Placebo	1	18	4/5/2015	75.2	HEIGHT	8.35	13.5	1	1
4	1063	12	Placebo	1	30	7/17/2016	82.1	HEIGHT	10.15	13	1	1
4	1063	24	Placebo	1	43	4/30/2017	86.6	HEIGHT	10.86363636	13.5	0	1
4	1063	36	Placebo	1	56	6/13/2018	94.2	HEIGHT	12.63636364	13.5	0	1
4	1063	48	Placebo	1	67	4/27/2019	99	HEIGHT	13.5	13.5	0	1
4	1063	60	Placebo	1	76	2/19/2020	103.8	HEIGHT	15.27272727	13.5	0	1
4	1064	0	Placebo	0	24	4/5/2015	79	LENGTH	10.5	15.5	1	1
4	1064	12	Placebo	0	30	7/17/2016	87.3	HEIGHT	12.5	15.5	1	1
4	1064	36	Placebo	0	56	6/13/2018	99.9	HEIGHT	15.27272727	16.5	0	1
4	1064	48	Placebo	0	67	4/27/2019	103.9	HEIGHT	16.55	16	0	1
4	1064	60	Placebo	0	76	2/19/2020	108.9	HEIGHT	17.22727273	15.8	0	1
4	1065	0	Placebo	0	24	4/5/2015	86.5	HEIGHT	12.15	16	0	1
4	1065	12	Placebo	0	36	7/17/2016	97.9	HEIGHT	15.2	16	0	1
4	1065	24	Placebo	0	49	4/30/2017	103.8	HEIGHT	16.36363636	15.5	0	1
4	1065	48	Placebo	0	73	4/27/2019	113.2	HEIGHT	18.95	15.5	0	1
4	1066	0	Placebo	1	18	4/5/2015	85.8	HEIGHT	12.3	16	0	1
4	1066	24	Placebo	1	55	4/30/2017	101.5	HEIGHT	15.63636364	15	0	1
4	1066	36	Placebo	1	68	6/13/2018	109.3	HEIGHT	17.63636364	15	0	1
4	1066	48	Placebo	1	79	4/27/2019	115.4	HEIGHT	19.4	15.5	0	1
4	1066	60	Placebo	1	88	2/19/2020	121.8	HEIGHT	21.77272727	15.3	0	1
4	1068	0	Placebo	1	48	4/5/2015	111.2	HEIGHT	18.3	16	0	1
4	1068	48	Placebo	1	115	4/27/2019	133.1	HEIGHT	26.65	17	0	1
4	1069	0	Placebo	0	54	4/5/2015	105.3	HEIGHT	15.9	15	0	1
4	1069	12	Placebo	0	54	7/17/2016	111.4	HEIGHT	17.25	15	1	1
4	1069	24	Placebo	0	67	4/30/2017	116.2	HEIGHT	18.81818182	15	0	1
4	1069	36	Placebo	0	80	6/13/2018	121.6	HEIGHT	21.18181818	16.5	0	1
4	1069	48	Placebo	0	91	4/27/2019	125.5	HEIGHT	22.45	16.5	0	1
4	1071	12	Placebo	1	54	7/17/2016	91.1	HEIGHT	14.1	16.5	1	1
4	1072	0	Placebo	0	36	4/5/2015	96.3	HEIGHT	14.95	15	1	1
4	1072	36	Placebo	0	76	6/13/2018	117.9	HEIGHT	21.81818182	16	0	1
4	1072	48	Placebo	0	87	4/27/2019	122	HEIGHT	23.1	16	0	1
4	1072	60	Placebo	0	96	2/19/2020	126.6	HEIGHT	26.86363636	17	0	1
4	1080	12	Placebo	1	12	7/17/2016	73.4	LENGTH	8.2	14	1	1
4	1080	36	Placebo	1	36	6/13/2018	85.5	HEIGHT	13.09090909	15.5	1	1
4	1080	48	Placebo	1	47	4/27/2019	96	HEIGHT	14.25	15.5	1	1
4	1080	60	Placebo	1	56	2/19/2020	102.5	HEIGHT	16.5	15.5	1	1
4	1083	12	Placebo	0	9	7/17/2016	72.2	LENGTH	8.7	13.5	0	1

4	1083	24	Placebo	0	21	4/30/2017	80.3	HEIGHT	9.909090909	13.5		0	1
4	1083	36	Placebo	0	35	6/13/2018	80.3	HEIGHT	11.90909091	15		1	1
4	1083	48	Placebo	0	45	4/27/2019	92.9	HEIGHT	13.75	13.5		1	1
4	1083	60	Placebo	0	55	2/19/2020	98.8	HEIGHT	15.13636364	13.5		0	1
4	1085	0	Placebo	1	36	4/5/2015	84.2	HEIGHT	11.5	15.5	30	0	1
4	1085	12	Placebo	1	54	7/17/2016	93	HEIGHT	13.4	15.5	30	0	1
4	1085	24	Placebo	1	63	4/30/2017	99.1	HEIGHT	14.22727273	15	30	0	1
4	1089	0	Placebo	1	18	4/5/2015	69.5	HEIGHT	8.65	12.5		0	1
4	1089	12	Placebo	1	30	7/17/2016	88.5	HEIGHT	10.9	13		0	1
4	1089	24	Placebo	1	46	4/30/2017	95.2	HEIGHT	11.31818182	12		1	1
4	1089	36	Placebo	1	59	6/13/2018	102.9	HEIGHT	13.5	13		0	1
4	1089	48	Placebo	1	70	4/27/2019	106.7	HEIGHT	14.9	13		0	1
4	1089	60	Placebo	1	79	2/19/2020	111.1	HEIGHT	16.40909091	13		0	1
4	1090	12	Placebo	1	7	7/17/2016	71.2	LENGTH	8.85	15		0	1
4	1090	24	Placebo	1	15	4/30/2017	80.5	HEIGHT	10.77272727	16		0	1
4	1090	36	Placebo	1	29	6/13/2018	89.8	HEIGHT	12.86363636	16		0	1
4	1090	60	Placebo	1	49	2/19/2020	99.8	HEIGHT	14.63636364	15.2		0	1
4	1095	0	Placebo	0	54	4/5/2015	116.6	HEIGHT	19.75	16.5		0	1
4	1095	12	Placebo	0	68	7/17/2016	123.7	HEIGHT	22.25	17		0	1
4	1095	24	Placebo	0	81	4/30/2017	127.5	HEIGHT	24.72727273	17		0	1
4	1095	36	Placebo	0	94	6/13/2018	133.1	HEIGHT	26.81818182	18		0	1
4	1101	0	Placebo	0	36	4/5/2015	89.4	HEIGHT	11.4	15		1	1
4	1101	24	Placebo	0	80	4/30/2017	106.4	HEIGHT	14.95454545	14		0	1
4	1101	36	Placebo	0	93	6/13/2018	114	HEIGHT	17.54545455	14.5		0	1
4	1101	48	Placebo	0	104	4/27/2019	118.8	HEIGHT	18.2	14.5		0	1
4	1101	60	Placebo	0	114	2/19/2020	124.5	HEIGHT	19.77272727	14.3		0	1
4	1102	0	Placebo	0	36	4/5/2015	92.1	HEIGHT	11.5	14		0	1
4	1103	12	Placebo	0	2	7/17/2016	67	LENGTH	7.7	14		0	1
4	8018	36	Placebo	0	58	6/13/2018	107	HEIGHT	16.40909091	15		1	1
4	8025	60	Placebo	0	48	2/19/2020	97.5	HEIGHT	14.63636364	13.9		1	1
4	8031	60	Placebo	0	41	2/20/2020	93.7	HEIGHT	13.54545455	14.2		1	1
4	8074	60	Placebo	0	31	2/20/2020	83.3	HEIGHT	11.72727273	14.5		1	1
4	8083	48	Placebo	1	39	4/27/2019	89.6	HEIGHT	11.75	14.5		1	1
4	8097	36	Placebo	1	25	6/13/2018	85.3	HEIGHT	13.36363636	17		1	1
4	8107	36	Placebo	1	56	6/13/2018	111.3	HEIGHT	18.36363636	15.5		1	1
4	8112	36	Placebo	1	12	6/13/2018	72.8	LENGTH	8.5	14		1	1
4	8112	48	Placebo	1	22	4/27/2019	81.2	LENGTH	8.65	12		0	1
4	8112	60	Placebo	1	32	2/19/2020	83.5	HEIGHT	12	14.3		1	1
4	8162	36	Placebo	0	38	6/13/2018	90.2	HEIGHT	13.40909091	16.5		1	1
4	8162	48	Placebo	0	48	4/27/2019	95.7	HEIGHT	15.35	16.5		1	1

4	8165	36	Placebo	1	1	6/13/2018	60.8	LENGTH	5.318181818	13		1	1
4	8165	48	Placebo	1	12	4/27/2019	70.9	LENGTH	8.25	14		0	1
4	8165	60	Placebo	1	21	2/19/2020	80	LENGTH	10.13636364	13.5		0	1
4	8178	36	Placebo	0	10	6/13/2018	70.8	LENGTH	8.136363636	14.5		1	1
4	8178	48	Placebo	0	20	4/27/2019	80.1	HEIGHT	11.3	16		0	1
4	8185	60	Placebo	0	32	2/19/2020	89.6	HEIGHT	12.95454545	15		1	1
4	8247	60	Placebo	1	35	2/19/2020	85.6	HEIGHT	12.90909091	15.5		1	1
4	8253	60	Placebo	0	32	2/19/2020	90.8	HEIGHT	12.36363636	12.7		1	1
4	8264	36	Placebo	1	27	6/13/2018	78.8	HEIGHT	10.13636364	16.5	48	1	1
4	8333	48	Placebo	0	7	4/27/2019	73.5	LENGTH	9.7	15.5		1	1
4	8340	48	Placebo	0	22	4/27/2019	79.4	LENGTH	9.55	13.5		1	1
4	8344	36	Placebo	1	34	6/13/2018	82.8	HEIGHT	10.36363636	14		1	1
4	8345	60	Placebo	1	14	2/19/2020	73.4	LENGTH	9.318181818	14.7		1	1
4	8372	36	Placebo	1	46	6/13/2018	91.5	HEIGHT	12.63636364	15	48	1	1
4	8378	36	Placebo	0	38	6/13/2018	93.2	HEIGHT	13.72727273	16.5		1	1
4	8384	60	Placebo	1	32	2/19/2020	87.3	HEIGHT	12.68181818	14.5		1	1
4	8416	36	Placebo	1	15	6/13/2018	76.9	HEIGHT	8.545454545	15		1	1
4	8416	48	Placebo	1	26	4/27/2019	84.2	HEIGHT	10.85	13.5		1	1
4	8437	36	Placebo	1	40	6/13/2018	100.4	HEIGHT	16.36363636	16		1	1
4	8475	60	Placebo	0	23	2/20/2020	82.9	LENGTH	10.40909091	14.3		1	1
4	8490	60	Placebo	1	32	2/19/2020	83.6	HEIGHT	11.63636364	14.5		1	1
4	8581	60	Placebo	1	9	2/19/2020	67	HEIGHT	6.454545455	12		1	1
4	8591	48	Placebo	0	2	4/27/2019	59.2	LENGTH	5.1	11.5		1	1
4	8591	60	Placebo	0	12	2/19/2020	70.6	HEIGHT	7.909090909	13		1	1
4	8614	48	Placebo	0	45	4/27/2019	99.3	HEIGHT	15.7	15.5		1	1
4	8614	60	Placebo	0	55	2/19/2020	105.9	HEIGHT	17.27272727	15		1	1
4	8615	48	Placebo	1	55	4/27/2019	104.1	HEIGHT	15.6	15.5		1	1
4	8646	60	Placebo	0	12	2/19/2020	73.6	HEIGHT	8.181818182	11.5		1	1
4	8683	48	Placebo	1	24	4/27/2019	79.8	LENGTH	8.4	12.5		1	1
4	8731	60	Placebo	1	11	2/19/2020	77.6	LENGTH	9.863636364	15		1	1
4	8768	60	Placebo	1	41	2/19/2020	91.8	HEIGHT	12.31818182	13.5		1	1
4	8821	60	Placebo	0	58	2/20/2020	100.8	HEIGHT	15.68181818	14.5		1	1
4	8848	48	Placebo	1	24	4/27/2019	74.4	LENGTH	7.95	11.5		1	1
4	8852	36	Placebo	1	12	6/13/2018	69.5	LENGTH	7.5	14		1	1
4	8852	48	Placebo	1	23	4/27/2019	80.4	LENGTH	9.55	14		0	1
4	8852	60	Placebo	1	32	2/19/2020	87.2	HEIGHT	12.13636364	14.5		1	1
4	8864	36	Placebo	1	15	6/13/2018	74.5	LENGTH	9.454545455	15		1	1
4	8982	60	Placebo	1	25	2/19/2020	83.2	HEIGHT	10.09090909	13.5		1	1
4	9021	36	Placebo	1	21	6/13/2018	73.4	LENGTH	8.681818182	13.5		1	1
4	9023	36	Placebo	1	17	6/13/2018	74	LENGTH	8.409090909	13		1	1

4	9074	60	Placebo	1	24	2/19/2020	81.7	HEIGHT	10.72727273	14.2	1	1
4	9080	60	Placebo	1	9	2/20/2020	67.2	LENGTH	7.181818182	13	1	1
4	9103	36	Placebo	1	26	6/13/2018	101.4	HEIGHT	13.59090909	13.5	1	1
4	9126	48	Placebo	1	10	4/27/2019	70.4	LENGTH	6.5	10.5	1	1
4	9126	60	Placebo	1	14	2/19/2020	78.9	HEIGHT	9.636363636	13	1	1
4	9127	36	Placebo	1	12	6/13/2018	69	LENGTH	6.863636364	13	1	1
4	9127	48	Placebo	1	22	4/27/2019	80	LENGTH	9.95	14.5	0	1
4	9127	60	Placebo	1	32	2/19/2020	89.9	HEIGHT	12.09090909	14	0	1
4	9155	60	Placebo	0	21	2/19/2020	87.5	HEIGHT	11.86363636	14	1	1
4	9202	48	Placebo	0	11	4/27/2019	71.4	LENGTH	9.3	15	1	1
4	9208	36	Placebo	1	15	6/13/2018	78.7	LENGTH	8.909090909	13	1	1
4	9208	48	Placebo	1	26	4/27/2019	84	HEIGHT	10.95	14	1	1
4	9229	36	Placebo	1	23	6/13/2018	78.8	HEIGHT	9.454545455	13.5	1	1
4	9229	60	Placebo	1	44	2/19/2020	88.5	HEIGHT	13.59090909	14.2	1	1
4	9231	36	Placebo	0	12	6/13/2018	78.9	LENGTH	10	14.5	1	1
4	9231	48	Placebo	0	22	4/27/2019	89.4	HEIGHT	11.25	14	0	1
4	9236	60	Placebo	1	44	2/19/2020	91.2	HEIGHT	12.68181818	14	1	1
4	9237	48	Placebo	0	12	4/27/2019	72.4	LENGTH	8.3	13.5	1	1
4	9237	60	Placebo	0	21	2/19/2020	80.7	LENGTH	10.09090909	14	1	1
4	9257	36	Placebo	1	21	6/13/2018	80.5	LENGTH	10.40909091	15	1	1
4	9332	48	Placebo	0	9	4/27/2019	70	LENGTH	7.4	13	1	1
4	9347	60	Placebo	0	3	2/20/2020	67.9	LENGTH	9.136363636	16	1	1
4	9374	60	Placebo	0	31	2/19/2020	87	HEIGHT	11.72727273	14.5	1	1
4	9377	48	Placebo	1	50	4/27/2019	81.2	HEIGHT	11.25	14.5	1	1
4	9398	48	Placebo	1	28	4/27/2019	77.3	HEIGHT	9.55	13.5	1	1
4	9409	36	Placebo	0	27	6/13/2018	78.6	HEIGHT	9.681818182	15.5	1	1
4	9413	48	Placebo	1	1	4/27/2019	55.5	LENGTH	4.85	12	1	1
4	9413	60	Placebo	1	10	2/19/2020	67.3	LENGTH	7.045454545	12.5	0	1
4	9463	36	Placebo	0	56	6/13/2018	107.6	HEIGHT	18.77272727	17	1	1
4	9465	60	Placebo	1	18	2/19/2020	76.2	HEIGHT	10.40909091	14.2	1	1
4	9483	48	Placebo	1	57	4/27/2019	106.1	HEIGHT	15.75	14	1	1
4	9492	36	Placebo	1	2	6/13/2018	59.7	LENGTH	5.636363636	14	1	1
4	9492	48	Placebo	1	13	4/27/2019	69.8	LENGTH	7.25	13	1	1
4	9531	48	Placebo	0	18	4/27/2019	78.3	LENGTH	9.8	14	1	1
4	9531	60	Placebo	0	27	2/20/2020	81.5	HEIGHT	12.40909091	14.3	1	1
4	9532	60	Placebo	0	9	2/20/2020	69.1	LENGTH	6.727272727	12.5	1	1
4	9582	60	Placebo	1	54	2/20/2020	100.4	HEIGHT	13.40909091	13	1	1
5	1118	0	Placebo	0	54	4/3/2015	123.3	HEIGHT	22.5	17	0	1
5	1118	12	Placebo	0	94	7/21/2016	128.8	HEIGHT		18.9	0	1
5	1118	24	Placebo	0	102	4/5/2017	131.7	HEIGHT	27.35	17.5	0	1

5	1119	24	Placebo	1	8	4/5/2017	68.4	LENGTH	7.1	13.5		1	1
5	1119	36	Placebo	1	22	5/22/2018	79.9	HEIGHT	9.3	13.5		0	1
5	1124	0	Placebo	1	54	4/3/2015	95	HEIGHT	13.9	15		0	1
5	1124	12	Placebo	1	62	7/21/2016	101.5	HEIGHT		15.4		0	1
5	1124	24	Placebo	1	74	4/5/2017	104.7	HEIGHT	16.4	15		0	1
5	1124	36	Placebo	1	88	5/22/2018	110.9	HEIGHT	18.35	15		0	1
5	1124	48	Placebo	1	99	4/26/2019	115.3	HEIGHT	19.35	15.5		0	1
5	1124	60	Placebo	1	108	2/16/2020	119.1	HEIGHT	21.65	15.5		0	1
5	1127	12	Placebo	0	42	7/21/2016	108.5	HEIGHT		14.8		1	1
5	1129	0	Placebo	1	30	4/3/2015	84.2	HEIGHT	9.95	13.5		0	1
5	1129	12	Placebo	1	42	7/21/2016	93.6	HEIGHT		15.8		0	1
5	1129	24	Placebo	1	56	4/5/2017	98	HEIGHT	13.65	15		0	1
5	1129	36	Placebo	1	69	5/22/2018	104.3	HEIGHT	15.75	15		0	1
5	1129	48	Placebo	1	80	4/26/2019	109.1	HEIGHT	16.35	15		0	1
5	1129	60	Placebo	1	90	2/16/2020	113.4	HEIGHT	17.5	15.5		0	1
5	1130	12	Placebo	1	7	7/21/2016	68.9	LENGTH		12.6		0	1
5	1130	36	Placebo	1	28	5/22/2018	83.9	HEIGHT	10.55	12.5		1	1
5	1131	0	Placebo	1	24	4/3/2015	81.6	HEIGHT	9.9	13		0	1
5	1131	12	Placebo	1	32	7/21/2016	88	HEIGHT		15.3		0	1
5	1131	36	Placebo	1	58	5/22/2018	99.9	HEIGHT	14.65	14		0	1
5	1131	48	Placebo	1	69	4/26/2019	106.1	HEIGHT	16.05	15		0	1
5	1132	0	Placebo	0	24	4/3/2015	83.1	HEIGHT	9.65	13		0	1
5	1132	12	Placebo	0	46	7/21/2016	93	HEIGHT		15.8		0	1
5	1132	24	Placebo	0	48	4/5/2017	99	HEIGHT	15.25	16		0	1
5	1132	36	Placebo	0	61	5/22/2018	106.5	HEIGHT	17.15	15.5		0	1
5	1132	48	Placebo	0	72	4/26/2019	112.8	HEIGHT	18.4	15.5		0	1
5	1132	60	Placebo	0	82	2/16/2020	116.9	HEIGHT	20.5	15.6		0	1
5	1133	12	Placebo	0	3	7/21/2016	67.5	LENGTH		14.8		0	1
5	1133	24	Placebo	0	15	4/5/2017	76.4	HEIGHT	8.75	14		1	1
5	1133	36	Placebo	0	29	5/22/2018	86.4	HEIGHT	12.1	14.5		0	1
5	1133	48	Placebo	0	40	4/26/2019	92.5	HEIGHT	12.7	14.5		1	1
5	1133	60	Placebo	0	50	2/16/2020	98.8	HEIGHT	15	14.5		0	1
5	1134	12	Placebo	0	8	7/21/2016	75.3	LENGTH		14.4	24	0	1
5	1135	12	Placebo	0	-3	7/21/2016	57.3	LENGTH		13.1		1	1
5	1135	24	Placebo	0	9	4/5/2017	69	HEIGHT	8.2	15		0	1
5	1135	48	Placebo	0	34	4/26/2019	88.9	HEIGHT	12.05	14		0	1
5	1135	60	Placebo	0	43	2/16/2020	93.9	HEIGHT	13.5	14		0	1
5	1136	0	Placebo	0	12	4/3/2015	75.6	LENGTH	8.95	15		1	1
5	1143	12	Placebo	1	12	7/21/2016	74	HEIGHT		12		1	1
5	1143	24	Placebo	1	24	4/5/2017	79.6	HEIGHT	9.5	13.5		0	1

5	1143	36	Placebo	1	39	5/22/2018	90.2	HEIGHT	11.4	13.5		0	1
5	1143	48	Placebo	1	50	4/26/2019	95.9	HEIGHT	12.45	14		0	1
5	1143	60	Placebo	1	59	2/16/2020	99.8	HEIGHT	13.75	14		0	1
5	1144	12	Placebo	0	48	7/21/2016	93.9	HEIGHT		13.9		1	1
5	1145	0	Placebo	0	6	4/3/2015	65.3	LENGTH	6.8	13		0	1
5	1145	12	Placebo	0	18	7/21/2016	81.1	HEIGHT		14.3		0	1
5	1145	24	Placebo	0	26	4/5/2017	84.6	HEIGHT	11.55	15		1	1
5	1145	36	Placebo	0	40	5/22/2018	94.3	HEIGHT	13.85	15.5		0	1
5	1145	48	Placebo	0	51	4/26/2019	101.6	HEIGHT	15.35	15.5		0	1
5	1145	60	Placebo	0	54	2/16/2020	108.5	HEIGHT	17.3	15.2		0	1
5	1147	0	Placebo	0	2	4/3/2015	57.3	LENGTH	4.95	12		1	1
5	1149	12	Placebo	0	44	7/21/2016	96.5	HEIGHT		15.6		1	1
5	1150	0	Placebo	1	36	4/3/2015	81.4	HEIGHT	8.45	12		0	1
5	1152	0	Placebo	1	48	4/3/2015	94.1	HEIGHT	13.9	15		1	1
5	1152	12	Placebo	1	56	7/21/2016	102.5	HEIGHT		15.2		0	1
5	1152	24	Placebo	1	68	4/5/2017	105.9	HEIGHT	16.2	15		0	1
5	1152	48	Placebo	1	93	4/26/2019	115	HEIGHT	18.85	15		0	1
5	1153	12	Placebo	0	7	7/21/2016	77	HEIGHT		15.2		0	1
5	1153	24	Placebo	0	20	4/5/2017	83.9	HEIGHT	11.2	15		0	1
5	1154	0	Placebo	1	10	4/3/2015	64.6	LENGTH	5.95	12		0	1
5	1154	12	Placebo	1	28	7/21/2016	78.1	HEIGHT		14.5		0	1
5	1154	24	Placebo	1	37	4/5/2017	84.2	HEIGHT	11.8	15		0	1
5	1154	48	Placebo	1	60	4/26/2019	100.9	HEIGHT	15.15	15		0	1
5	1154	60	Placebo	1	70	2/16/2020	107.2	HEIGHT	17.2	15.2		0	1
5	1155	24	Placebo	0	50	4/5/2017	95	HEIGHT	16.35	16		1	1
5	1156	24	Placebo	0	24	4/5/2017	85.7	HEIGHT	10.55	14.5		1	1
5	1157	0	Placebo	0	36	4/3/2015	89.8	HEIGHT	11.5	13.5		1	1
5	1157	12	Placebo	0	44	7/21/2016	98.1	HEIGHT		13.3		0	1
5	1157	24	Placebo	0	56	4/5/2017	102.9	HEIGHT	13.95	13		1	1
5	1157	36	Placebo	0	79	5/22/2018	109.9	HEIGHT	16.8	13.5		0	1
5	1157	48	Placebo	0	90	4/26/2019	113.3	HEIGHT	17.25	14		0	1
5	1159	12	Placebo	0	8	7/21/2016	67.6	LENGTH		12.5	18	0	1
5	1160	12	Placebo	0	9	7/21/2016	69.7	LENGTH		14.2	30	0	1
5	1160	24	Placebo	0	23	4/5/2017	78.4	HEIGHT	9.3	15	30	0	1
5	1161	24	Placebo	0	37	4/5/2017	88	HEIGHT	12.55	15		1	1
5	1161	36	Placebo	0	51	5/22/2018	93.9	HEIGHT	13.95	14.5		1	1
5	1163	0	Placebo	0	24	4/3/2015	81.9	HEIGHT	10.25	14		0	1
5	1163	12	Placebo	0	32	7/21/2016	88.9	HEIGHT		14.3		0	1
5	1163	24	Placebo	0	44	4/5/2017	94.7	HEIGHT	13.2	15		1	1
5	1163	48	Placebo	0	69	4/26/2019	109.3	HEIGHT	16.9	14.5		0	1

5	1163	60	Placebo	0	78	2/16/2020	113.1	HEIGHT	18.3	14.8		0	1
5	1164	0	Placebo	0	36	4/3/2015	96.8	HEIGHT	14.3	15		0	1
5	1164	12	Placebo	0	49	7/21/2016	107	HEIGHT		15		0	1
5	1165	0	Placebo	1	54	4/3/2015	100.8	HEIGHT	14.55	14		0	1
5	1165	48	Placebo	1	103	4/26/2019	123.8	HEIGHT	22.95	16		0	1
5	1168	0	Placebo	1	36	4/3/2015	90.3	HEIGHT	12.3	15		0	1
5	1168	12	Placebo	1	46	7/21/2016	98	HEIGHT		16.4		1	1
5	1168	24	Placebo	1	54	4/5/2017	104.4	HEIGHT	16.65	15.5		1	1
5	1168	36	Placebo	1	68	5/22/2018	112.7	HEIGHT	18.45	15		0	1
5	1168	48	Placebo	1	79	4/26/2019	117.3	HEIGHT	19.8	15.5		0	1
5	1172	0	Placebo	1	36	4/3/2015	104.7	HEIGHT	15.85	16		1	1
5	1172	24	Placebo	1	82	4/5/2017	117.3	HEIGHT	18.6	15		0	1
5	1172	36	Placebo	1	96	5/22/2018	123.5	HEIGHT	21.9	16		0	1
5	1172	48	Placebo	1	107	4/26/2019	128.2	HEIGHT	22.9	16		0	1
5	1172	60	Placebo	1	116	2/16/2020	131.8	HEIGHT	25.4	17		0	1
5	1174	0	Placebo	0	24	4/3/2015	115.7	HEIGHT	19.7	15.5		1	1
5	1174	12	Placebo	0	90	7/21/2016	121.8	HEIGHT		16.1		0	1
5	1174	24	Placebo	0	102	4/5/2017	126.4	HEIGHT	23.65	16.5		0	1
5	1174	36	Placebo	0	116	5/22/2018	131	HEIGHT	25.1	16.5		0	1
5	1174	48	Placebo	0	127	4/26/2019	133.7	HEIGHT	27.85	17		0	1
5	1174	60	Placebo	0	137	2/16/2020	136.5	HEIGHT	29.75	17.5		0	1
5	1176	24	Placebo	0	44	4/5/2017	105	HEIGHT	15.85	14.5		1	1
5	1177	12	Placebo	0	58	7/21/2016	101.3	HEIGHT		15.3		1	1
5	1180	0	Placebo	0	36	4/3/2015	94.5	HEIGHT	15.15	16		0	1
5	1180	12	Placebo	0	52	7/21/2016	103.5	HEIGHT		15.9		0	1
5	1180	24	Placebo	0	61	4/5/2017	106.1	HEIGHT	17.2	15		0	1
5	1180	48	Placebo	0	91	4/26/2019	115.8	HEIGHT	20.4	15		0	1
5	1180	60	Placebo	0	100	2/16/2020	120.5	HEIGHT	21.8	15		0	1
5	1183	0	Placebo	1	6	4/3/2015	61.7	LENGTH	5.6	13	42	0	1
5	1183	12	Placebo	1	18	7/21/2016	74.9	LENGTH		12.5	42	1	1
5	1183	24	Placebo	1	31	4/5/2017	78.4	HEIGHT	8.2	13	42	0	1
5	1183	36	Placebo	1	44	5/22/2018	88.7	HEIGHT	10.35	13.5	42	0	1
5	1185	12	Placebo	0	8	7/21/2016	76	HEIGHT		17.5		0	1
5	1185	24	Placebo	0	15	4/5/2017	83.7	HEIGHT	11.05	16		0	1
5	1185	60	Placebo	0	49	2/16/2020	103.9	HEIGHT	16.4	15.4		0	1
5	1186	12	Placebo	1	8	7/21/2016	66.4	LENGTH		11		0	1
5	1186	24	Placebo	1	20	4/5/2017	74.7	HEIGHT	7.85	12.5		0	1
5	1186	36	Placebo	1	34	5/22/2018	81.6	LENGTH	11.15	14.5		1	1
5	1187	0	Placebo	0	42	4/3/2015	90.3	HEIGHT	11.7	14		0	1
5	1189	0	Placebo	0	18	4/3/2015	84.3	HEIGHT	12.1	13	12	0	1

5	1191	0	Placebo	0	48	4/3/2015	106.4	HEIGHT	17.1	14.5	0	1
5	1191	12	Placebo	0	56	7/21/2016	111.8	HEIGHT		14.6	0	1
5	1191	36	Placebo	0	82	5/22/2018	121.1	HEIGHT	22.1	15	0	1
5	1191	48	Placebo	0	93	4/26/2019	124	HEIGHT	22.95	15.5	0	1
5	1192	12	Placebo	1	54	7/21/2016	116.6	HEIGHT		14.6	1	1
5	1194	0	Placebo	1	24	4/3/2015	69.5	LENGTH	7.65	12.5	0	1
5	1194	12	Placebo	1	41	7/21/2016	81	LENGTH		13.2	0	1
5	1197	12	Placebo	0	6	7/21/2016	68.9	LENGTH		14.2	0	1
5	1197	24	Placebo	0	15	4/5/2017	73.9	HEIGHT	8.65	14	0	1
5	1197	36	Placebo	0	28	5/22/2018	81.2	HEIGHT	10.95	14.5	0	1
5	1197	48	Placebo	0	39	4/26/2019	85.3	HEIGHT	12.1	14.5	1	1
5	1197	60	Placebo	0	49	2/16/2020	91.2	HEIGHT	15.05	17	0	1
5	1200	12	Placebo	1	4	7/21/2016	56	LENGTH		9.2	0	1
5	1200	36	Placebo	1	29	5/22/2018	72.3	HEIGHT	5.8	10	0	1
5	1201	0	Placebo	1	5	4/3/2015	65.8	LENGTH	6.05	12.5	1	1
5	1201	12	Placebo	1	14	7/21/2016	78.5	HEIGHT		12.5	0	1
5	1201	36	Placebo	1	43	5/22/2018	91.2	HEIGHT	10.9	12.5	0	1
5	1201	48	Placebo	1	54	4/26/2019	97.6	HEIGHT	11.9	13	1	1
5	1204	0	Placebo	1	4	4/3/2015	64	LENGTH	6.3	12.5	0	1
5	1205	12	Placebo	0	8	7/21/2016	73	HEIGHT		15.3	0	1
5	1205	24	Placebo	0	15	4/5/2017	81.6	HEIGHT	10.8	14	0	1
5	1205	60	Placebo	0	49	2/16/2020	107.1	HEIGHT	18.7	14.3	0	1
5	1206	0	Placebo	1	10	4/3/2015	66.4	LENGTH	6.4	12	0	1
5	1206	12	Placebo	1	18	7/21/2016	78.6	HEIGHT		14.1	0	1
5	1206	24	Placebo	1	30	4/5/2017	81.7	HEIGHT	10	13.5	0	1
5	1206	48	Placebo	1	60	4/26/2019	94.7	HEIGHT	12.45	14	0	1
5	1206	60	Placebo	1	70	2/16/2020	100.4	HEIGHT	14.35	14	0	1
5	1208	0	Placebo	1	48	4/3/2015	106.2	HEIGHT	15.35	14.5	0	1
5	1208	12	Placebo	1	56	7/21/2016	113.4	HEIGHT		15.8	0	1
5	1208	60	Placebo	1	102	2/16/2020	127.7	HEIGHT	24	16.5	0	1
5	1209	0	Placebo	1	18	4/3/2015	74.3	LENGTH	8.55	13	0	1
5	1209	24	Placebo	1	38	4/5/2017	90.3	HEIGHT	12.05	14	0	1
5	1209	36	Placebo	1	52	5/22/2018	97.5	HEIGHT	13.2	14	0	1
5	1209	48	Placebo	1	63	4/26/2019	102.4	HEIGHT	15.2	14.5	0	1
5	1210	0	Placebo	0	48	4/3/2015	96.8	HEIGHT	14.7	16	1	1
5	1210	36	Placebo	0	82	5/22/2018	115.7	HEIGHT	18.85	16	0	1
5	1210	48	Placebo	0	93	4/26/2019	121.2	HEIGHT	21.1	16	0	1
5	1212	0	Placebo	1	36	4/3/2015	89.7	HEIGHT	12.75	15.5	0	1
5	1213	0	Placebo	0	36	4/3/2015	80.8	HEIGHT	8.95	12.5	1	1
5	1213	12	Placebo	0	34	7/21/2016	91.5	HEIGHT		13.6	0	1

5	1213	24	Placebo	0	42	4/5/2017	97.6	HEIGHT	13	13.5	0	1
5	1213	48	Placebo	0	67	4/26/2019	110.5	HEIGHT	16.45	13	0	1
5	1213	60	Placebo	0	77	2/16/2020	115.5	HEIGHT	19	13.9	0	1
5	1214	0	Placebo	0	48	4/3/2015	116.6	HEIGHT	20.25	15.5	0	1
5	1214	12	Placebo	0	56	7/21/2016	121.1	HEIGHT		16.1	0	1
5	1214	24	Placebo	0	68	4/5/2017	125	HEIGHT	23.4	16.5	0	1
5	1214	36	Placebo	0	82	5/22/2018	130.9	HEIGHT	26.45	17	0	1
5	1214	48	Placebo	0	93	4/26/2019	134.6	HEIGHT	27.3	17	0	1
5	1215	0	Placebo	1	48	4/3/2015	112.7	HEIGHT	16.9	14	1	1
5	1215	60	Placebo	1	120	2/16/2020	139.8	HEIGHT	26.95	16	0	1
5	1217	12	Placebo	0	10	7/21/2016	68.3	LENGTH		14.5	0	1
5	1218	24	Placebo	1	31	4/5/2017	86.9	HEIGHT	11.35	14	1	1
5	1219	0	Placebo	1	12	4/3/2015	74.1	HEIGHT	7.95	13.5	1	1
5	1219	12	Placebo	1	25	7/21/2016	81.6	HEIGHT		13.4	1	1
5	1219	48	Placebo	1	69	4/26/2019	99.9	HEIGHT	13.45	13.5	0	1
5	1219	60	Placebo	1	79	2/16/2020	104.6	HEIGHT	15.85	14.7	0	1
5	1224	24	Placebo	0	2	4/5/2017	54.2	LENGTH	5.4	13	1	1
5	1224	36	Placebo	0	16	5/22/2018	72.9	HEIGHT	8.9	14	0	1
5	1224	48	Placebo	0	26	4/26/2019	81	HEIGHT	10.2	14	0	1
5	1225	0	Placebo	0	10	4/3/2015	72.3	LENGTH	8.85	14.5	0	1
5	1225	12	Placebo	0	20	7/21/2016	85	HEIGHT		15.4	1	1
5	1225	24	Placebo	0	32	4/5/2017	89.7	HEIGHT	13.1	16.5	0	1
5	1225	36	Placebo	0	42	5/22/2018	97.9	HEIGHT	15.15	15.5	1	1
5	1225	48	Placebo	0	59	4/26/2019	105.8	HEIGHT	16.8	16	1	1
5	1226	24	Placebo	1	56	4/5/2017	100	HEIGHT	17.1	16	1	1
5	1230	24	Placebo	0	10	4/5/2017	69.5	HEIGHT	7.4	13.5	1	1
5	1230	36	Placebo	0	21	5/22/2018	79.2	HEIGHT	9.1	12.5	0	1
5	1230	48	Placebo	0	33	4/26/2019	86	HEIGHT	10.8	13.5	0	1
5	1230	60	Placebo	0	43	2/16/2020	92.8	HEIGHT	12.4	13.5	0	1
5	1231	24	Placebo	1	38	4/5/2017	95.7	HEIGHT	14.65	16	1	1
5	1231	36	Placebo	1	52	5/22/2018	103.5	HEIGHT	16.75	16	1	1
5	1233	12	Placebo	0	6	7/21/2016	72.3	LENGTH		14.5	1	1
5	1233	24	Placebo	0	15	4/5/2017	78.3	HEIGHT	9.1	13.5	0	1
5	1233	36	Placebo	0	28	5/22/2018	87.2	HEIGHT	12.35	14.5	0	1
5	1233	48	Placebo	0	39	4/26/2019	93.7	HEIGHT	12.95	14.5	0	1
5	1233	60	Placebo	0	49	2/16/2020	100.5	HEIGHT	14.6	14.2	1	1
5	1234	0	Placebo	1	36	4/3/2015	85.7	HEIGHT	10.25	14	0	1
5	1236	24	Placebo	0	48	4/5/2017	106.6	HEIGHT	18.1	16	1	1
5	1238	0	Placebo	0	12	4/3/2015	79.5	HEIGHT	11.55	17	0	1
5	1238	12	Placebo	0	30	7/21/2016	89.8	HEIGHT		16.5	0	1

5	1238	24	Placebo	0	42	4/5/2017	95.5	HEIGHT	14.7	17	1	1
5	1238	36	Placebo	0	50	5/22/2018	103.8	HEIGHT	16.35	16	0	1
5	1238	48	Placebo	0	61	4/26/2019	111.6	HEIGHT	17.8	16	0	1
5	1240	12	Placebo	0	42	7/21/2016	103.5	HEIGHT		16.2	1	1
5	1242	24	Placebo	0	24	4/5/2017	82.6	HEIGHT	10.15	14	1	1
5	1243	0	Placebo	1	36	4/3/2015	99.6	HEIGHT	13.3	14.5	1	1
5	1243	12	Placebo	1	44	7/21/2016	107.5	HEIGHT		14.7	1	1
5	1243	24	Placebo	1	56	4/5/2017	109.7	HEIGHT	15.75	14.5	0	1
5	1243	48	Placebo	1	81	4/26/2019	118	HEIGHT	17.95	14.5	0	1
5	1245	24	Placebo	0	30	4/5/2017	94	HEIGHT	14.2	16	1	1
5	1249	12	Placebo	1	6	7/21/2016	68.3	LENGTH		14	0	1
5	1249	24	Placebo	1	15	4/5/2017	72.8	HEIGHT	9.1	15	0	1
5	1249	36	Placebo	1	28	5/22/2018	83.3	HEIGHT	12	15	0	1
5	1249	60	Placebo	1	49	2/16/2020	97.1	HEIGHT	15.1	16	0	1
5	1250	12	Placebo	1	54	7/21/2016	115.5	HEIGHT		15.8	1	1
5	1251	0	Placebo	0	36	4/3/2015	83.3	HEIGHT	11.15	14.5	1	1
5	1251	12	Placebo	0	42	7/21/2016	90	HEIGHT		14.8	0	1
5	1251	48	Placebo	0	79	4/26/2019	106.4	HEIGHT	16.85	14.5	0	1
5	1254	0	Placebo	0	48	4/3/2015	93.6	HEIGHT	15.3	16	0	1
5	1257	12	Placebo	1	12	7/21/2016	76.6	LENGTH		15	1	1
5	1258	24	Placebo	0	24	4/5/2017	78.7	HEIGHT	9.9	14.5	1	1
5	1258	60	Placebo	0	54	2/16/2020	95.1	HEIGHT	13.2	13.5	1	1
5	1259	0	Placebo	1	24	4/3/2015	71.5	LENGTH	7.3	12	1	1
5	1259	12	Placebo	1	30	7/21/2016	77	HEIGHT		15.1	0	1
5	1259	24	Placebo	1	42	4/5/2017	81	HEIGHT	11.1	15.5	0	1
5	1259	36	Placebo	1	56	5/22/2018	89.4	HEIGHT	12.95	14.5	0	1
5	1260	12	Placebo	1	5	7/21/2016	62.6	LENGTH		14	0	1
5	1260	24	Placebo	1	15	4/5/2017	70.8	HEIGHT	8.05	13	0	1
5	1260	36	Placebo	1	36	5/22/2018	80	HEIGHT	9.8	12.5	1	1
5	1260	60	Placebo	1	57	2/16/2020	90.2	HEIGHT	12.5	13.7	1	1
5	1261	12	Placebo	1	9	7/21/2016	72.2	LENGTH		17.5	0	1
5	1261	36	Placebo	1	31	5/22/2018	85.2	HEIGHT	12.3	15.5	0	1
5	1263	12	Placebo	0	-2	7/21/2016	56.7	LENGTH		14.3	0	1
5	1263	24	Placebo	0	10	4/5/2017	70.2	HEIGHT	8.05	14.5	0	1
5	1263	60	Placebo	0	41	2/16/2020	96.6	HEIGHT	13.95	15	0	1
5	1264	0	Placebo	1	24	4/3/2015	82.8	HEIGHT	10	14	0	1
5	1265	0	Placebo	1	48	4/3/2015	106.9	HEIGHT	14.2	13.5	1	1
5	1265	24	Placebo	1	68	4/5/2017	119.6	HEIGHT	18.3	14.5	0	1
5	1265	36	Placebo	1	82	5/22/2018	125.9	HEIGHT	19.85	15	0	1
5	1265	48	Placebo	1	93	4/26/2019	130.3	HEIGHT	22.3	15.5	0	1

5	1265	60	Placebo	1	102	2/16/2020	133.8	HEIGHT	25.15	16.5		0	1
5	1270	24	Placebo	1	50	4/5/2017	104.2	HEIGHT	15.4	16		1	1
5	1271	0	Placebo	0	48	4/3/2015	108.3	HEIGHT	15.85	14		0	1
5	1271	48	Placebo	0	93	4/26/2019	126.9	HEIGHT	22.35	16		0	1
5	1273	12	Placebo	0	34	7/21/2016	86	HEIGHT		15.2		0	1
5	1275	0	Placebo	0	36	4/3/2015	84	HEIGHT	10.8	13.5		0	1
5	1275	12	Placebo	0	42	7/21/2016	92.5	HEIGHT		14.9		0	1
5	1275	24	Placebo	0	54	4/5/2017	98.5	HEIGHT	14.35	15.5		0	1
5	1275	36	Placebo	0	57	5/22/2018	106.5	HEIGHT	16.7	15		0	1
5	1275	48	Placebo	0	68	4/26/2019	110.1	HEIGHT	18.1	15		0	1
5	1275	60	Placebo	0	77	2/16/2020	114.9	HEIGHT	19.2	14.7		0	1
5	1278	0	Placebo	0	12	4/3/2015	66.3	LENGTH	6.4	12	18	0	1
5	1281	0	Placebo	1	7	4/3/2015	64.5	LENGTH	7.45	14.5		0	1
5	1281	12	Placebo	1	18	7/21/2016	80.2	HEIGHT		14.9		0	1
5	1281	36	Placebo	1	44	5/22/2018	92.8	HEIGHT	12.35	14		0	1
5	1281	48	Placebo	1	55	4/26/2019	98.3	HEIGHT	13.5	13.5		0	1
5	1281	60	Placebo	1	65	2/16/2020	103.1	HEIGHT	15.05	14.5		0	1
5	1283	0	Placebo	0	48	6/13/2015	111.1	HEIGHT	17.8	15.5		1	1
5	1284	0	Placebo	0	12	4/3/2015	75.3	LENGTH	9.35	14.5		0	1
5	1285	12	Placebo	0	2	7/21/2016	68.6	LENGTH		15.5		0	1
5	1285	24	Placebo	0	14	4/5/2017	76.6	HEIGHT	9.3	14.5		0	1
5	1285	36	Placebo	0	28	5/22/2018	85.7	HEIGHT	12.1	15.5		1	1
5	1285	60	Placebo	0	48	2/16/2020	100.5	HEIGHT	15.3	15.7		0	1
5	1288	0	Placebo	1	24	4/3/2015	92.4	HEIGHT	13.2	14.5		1	1
5	1289	0	Placebo	1	42	4/3/2015	94.9	HEIGHT	13.75	15.5		0	1
5	1289	12	Placebo	1	54	7/21/2016	105	HEIGHT		14.5		0	1
5	1289	24	Placebo	1	66	4/5/2017	108.2	HEIGHT	17.05	15		0	1
5	1289	36	Placebo	1	80	5/22/2018	114.7	HEIGHT	18.75	15.5		0	1
5	1289	48	Placebo	1	91	4/26/2019	118.9	HEIGHT	20.55	16		0	1
5	1289	60	Placebo	1	101	2/16/2020	122.5	HEIGHT	22.55	16		0	1
5	1296	24	Placebo	1	5	4/5/2017	62.6	LENGTH	6.5	13		1	1
5	1296	36	Placebo	1	20	5/22/2018	75.8	LENGTH	9	14		0	1
5	1296	48	Placebo	1	31	4/26/2019	84	HEIGHT	11.25	14.5		1	1
5	1296	60	Placebo	1	41	2/16/2020	91.1	HEIGHT	13.5	15.7		0	1
5	1297	0	Placebo	1	48	4/3/2015	103.1	HEIGHT	16.4	16.5		1	1
5	1297	24	Placebo	1	80	4/5/2017	111.7	HEIGHT	17.75	16.5		0	1
5	1297	48	Placebo	1	105	4/26/2019	121.4	HEIGHT	20.9	17		0	1
5	1302	0	Placebo	0	36	4/3/2015	95.5	HEIGHT	13.45	14.5		0	1
5	1302	12	Placebo	0	42	7/21/2016	103	HEIGHT		16		1	1
5	1302	24	Placebo	0	54	4/5/2017	108.2	HEIGHT	17.3	15.5		0	1

5	1302	48	Placebo	0	79	4/26/2019	118	HEIGHT	19.75	16		0	1
5	1303	0	Placebo	0	48	6/13/2015	104.6	HEIGHT	16.15	15.5		1	1
5	1303	48	Placebo	0	91	4/26/2019	123.7	HEIGHT	22.75	16.5		0	1
5	1303	60	Placebo	0	101	2/16/2020	126.3	HEIGHT	23.4	16.5		0	1
5	1306	0	Placebo	0	24	4/3/2015	75.2	LENGTH	8.9	14.5		0	1
5	1306	12	Placebo	0	32	7/21/2016	86.5	HEIGHT		16.5		1	1
5	1306	24	Placebo	0	44	4/5/2017	91.8	HEIGHT	13.9	15.5		0	1
5	1306	36	Placebo	0	58	5/22/2018	100.9	HEIGHT	16	16		1	1
5	1306	48	Placebo	0	69	4/26/2019	105.4	HEIGHT	15.95	15		0	1
5	1306	60	Placebo	0	78	2/16/2020	110.9	HEIGHT	18.55	16		0	1
5	1308	24	Placebo	1	54	4/5/2017	90.2	HEIGHT	10.55	13.5		1	1
5	1309	0	Placebo	1	12	6/13/2015	120.3	HEIGHT	21.35	15.5		1	1
5	1309	12	Placebo	1	86	7/21/2016	126.8	HEIGHT		16		0	1
5	1311	0	Placebo	1	48	4/3/2015	89.2	HEIGHT	14.05	16.5		0	1
5	1311	12	Placebo	1	42	7/21/2016	98.3	HEIGHT		17.7		0	1
5	1312	12	Placebo	1	42	7/21/2016	86.7	HEIGHT		15.1		1	1
5	1313	24	Placebo	0	30	4/5/2017	85.9	HEIGHT	11.05	15		1	1
5	1315	0	Placebo	0	12	4/3/2015	79.6	LENGTH	9.45	14		0	1
5	1315	12	Placebo	0	24	7/21/2016	90.6	HEIGHT		14.5		0	1
5	1315	36	Placebo	0	46	5/22/2018	103.2	HEIGHT	14.9	15		0	1
5	1315	48	Placebo	0	57	4/26/2019	108.5	HEIGHT	15.2	14		0	1
5	1315	60	Placebo	0	56	2/16/2020	113.4	HEIGHT	17.15	15		1	1
5	1316	0	Placebo	0	12	4/3/2015	69.8	LENGTH	5.9	10		0	1
5	1316	12	Placebo	0	28	7/21/2016	79.8	LENGTH		13.9		0	1
5	1316	24	Placebo	0	37	4/5/2017	83.7	HEIGHT	11.05	14		1	1
5	1317	0	Placebo	0	54	4/3/2015	108.7	HEIGHT	17.4	14.5		0	1
5	1317	12	Placebo	0	90	7/21/2016	114.1	HEIGHT		15.5		0	1
5	1317	36	Placebo	0	116	5/22/2018	121.2	HEIGHT	22.05	15		0	1
5	1318	24	Placebo	0	54	4/5/2017	99.2	HEIGHT	14.15	15		1	1
5	1319	12	Placebo	1	3	7/21/2016	57.8	LENGTH		10		0	1
5	1319	36	Placebo	1	29	5/22/2018	73.3	LENGTH	6.85	11.5		0	1
5	1320	12	Placebo	0	-2	7/21/2016	60.8	LENGTH		14		0	1
5	1320	60	Placebo	0	43	2/16/2020	97.4	HEIGHT	15.65	15.5		1	1
5	1321	12	Placebo	0	2	7/21/2016	71.6	LENGTH		16.3	60	0	1
5	1321	24	Placebo	0	14	4/5/2017	74.8	HEIGHT	8.95	15	60	1	1
5	1321	36	Placebo	0	28	5/22/2018	83.7	HEIGHT	13.7	17	60	0	1
5	1324	0	Placebo	1	36	4/3/2015	82.9	HEIGHT	10.45	14		0	1
5	1324	12	Placebo	1	48	7/21/2016	92.5	HEIGHT		17.9		1	1
5	1324	24	Placebo	1	56	4/5/2017	98.4	HEIGHT	15.65	16		0	1
5	1325	24	Placebo	1	42	4/5/2017	83.6	HEIGHT	14.75	17		1	1

5	1326	0	Placebo	0	24	4/3/2015	83.2	HEIGHT	11.35	15		1	1
5	1329	12	Placebo	0	58	7/21/2016	112.2	HEIGHT		16.1		1	1
5	1331	0	Placebo	1	36	6/13/2015	80.7	HEIGHT	10.95	13.5		1	1
5	1331	24	Placebo	1	42	4/5/2017	100.1	HEIGHT	16.3	15.5		0	1
5	1331	48	Placebo	1	67	4/26/2019	113	HEIGHT	18.5	15		0	1
5	1332	0	Placebo	1	54	4/3/2015	133.2	HEIGHT	28.3	19		0	1
5	1333	0	Placebo	1	6	4/3/2015	62.3	LENGTH	6.25	12.5		0	1
5	1333	12	Placebo	1	12	7/21/2016	75.4	HEIGHT		13.9		0	1
5	1333	48	Placebo	1	54	4/26/2019	91.3	HEIGHT	13.15	15		0	1
5	1333	60	Placebo	1	63	2/16/2020	98.1	HEIGHT	15.05	15		0	1
5	1338	0	Placebo	0	18	4/3/2015	83.1	HEIGHT	10.7	15		0	1
5	1338	24	Placebo	0	49	4/5/2017	97.9	HEIGHT	14	14.5		0	1
5	1338	36	Placebo	0	62	5/22/2018	104.9	HEIGHT	15.4	14.5		0	1
5	1338	48	Placebo	0	73	4/26/2019	110.3	HEIGHT	17.5	15.5		0	1
5	1339	0	Placebo	0	48	4/3/2015	97.4	HEIGHT	14.1	14.5		0	1
5	1339	24	Placebo	0	66	4/5/2017	108.5	HEIGHT	17.6	15		0	1
5	1339	36	Placebo	0	80	5/22/2018	113.5	HEIGHT	19.4	16		0	1
5	1339	48	Placebo	0	91	4/26/2019	118.1	HEIGHT	20.65	15.5		0	1
5	1342	12	Placebo	0	44	7/21/2016	103.5	HEIGHT		16.4		1	1
5	1345	0	Placebo	0	36	4/3/2015	102.9	HEIGHT	16	15		1	1
5	1346	12	Placebo	0	12	7/22/2016	68.9	LENGTH		12.5	24	1	1
5	1350	0	Placebo	0	1	4/3/2015	57.1	LENGTH	5.55	13.5		1	1
5	1350	12	Placebo	0	12	7/21/2016	71.3	LENGTH		14.2		0	1
5	1353	0	Placebo	0	36	4/3/2015	92.9	HEIGHT	14.1	15.5		0	1
5	1355	24	Placebo	0	8	4/5/2017	80	HEIGHT	11.95	15.5		1	1
5	1355	36	Placebo	0	34	5/22/2018	92.4	HEIGHT	15.8	16.5		1	1
5	1355	48	Placebo	0	45	4/26/2019	98.2	HEIGHT	16.25	16		0	1
5	1356	24	Placebo	0	8	4/5/2017	66.3	LENGTH	5.2	10.5	42	1	1
5	1357	0	Placebo	1	36	4/3/2015	88.4	HEIGHT	10.5	13		0	1
5	1357	12	Placebo	1	44	7/21/2016	99.4	HEIGHT		13.5		0	1
5	1357	24	Placebo	1	56	4/5/2017	103.6	HEIGHT	14.1	13.5		0	1
5	1357	48	Placebo	1	81	4/26/2019	115.4	HEIGHT	16.5	13.5		0	1
5	1358	0	Placebo	0	48	6/13/2015	107.3	HEIGHT	17.45	16		0	1
5	1358	12	Placebo	0	56	7/21/2016	114.5	HEIGHT		16.9		0	1
5	1358	24	Placebo	0	68	4/5/2017	119	HEIGHT	21.5	17.5		0	1
5	1358	36	Placebo	0	82	5/22/2018	124.5	HEIGHT	23.3	17		0	1
5	1359	0	Placebo	1	36	4/3/2015	91.4	HEIGHT	13.15	13.5		0	1
5	1359	24	Placebo	1	54	4/5/2017	103.4	HEIGHT	16.35	14.5		0	1
5	1359	48	Placebo	1	79	4/26/2019	113.4	HEIGHT	18.3	14		0	1
5	1361	24	Placebo	0	32	4/5/2017	87.2	HEIGHT	12.2	15		1	1

5	1361	36	Placebo	0	46	5/22/2018	94.2	HEIGHT	13.65	14	1	1
5	1362	12	Placebo	0	24	7/21/2016	79.7	HEIGHT		14.2	1	1
5	1364	24	Placebo	0	14	4/5/2017	74.2	HEIGHT	9.75	15.5	1	1
5	1365	12	Placebo	1	9	7/21/2016	74.4	HEIGHT		14.8	0	1
5	1366	12	Placebo	1	42	7/21/2016	89.7	HEIGHT		14.9	1	1
5	1369	0	Placebo	1	42	4/3/2015	93.7	HEIGHT	13.4	15	1	1
5	1369	12	Placebo	1	54	7/21/2016	103.8	HEIGHT		15.5	1	1
5	1369	24	Placebo	1	62	4/5/2017	107.7	HEIGHT	15.9	14.5	0	1
5	1369	48	Placebo	1	87	4/26/2019	119.2	HEIGHT	19.6	15	0	1
5	1370	0	Placebo	0	48	4/3/2015	104.4	HEIGHT	15.25	15	0	1
5	1370	12	Placebo	0	56	7/21/2016	112.4	HEIGHT		16	0	1
5	1370	24	Placebo	0	79	4/5/2017	116.5	HEIGHT	19.2	15.5	0	1
5	1370	36	Placebo	0	92	5/22/2018	122.6	HEIGHT	21.55	16	0	1
5	1370	48	Placebo	0	103	4/26/2019	126.4	HEIGHT	22.5	15.5	0	1
5	1371	0	Placebo	1	30	6/13/2015	85.9	HEIGHT	9.75	13.5	1	1
5	1371	24	Placebo	1	50	4/5/2017	102.2	HEIGHT	15	15.5	0	1
5	1371	36	Placebo	1	64	5/22/2018	110.7	HEIGHT	16.9	15	0	1
5	1371	48	Placebo	1	75	4/26/2019	116.7	HEIGHT	18.85	15	0	1
5	1371	60	Placebo	1	84	2/16/2020	121	HEIGHT	22.05	16.7	0	1
5	1372	0	Placebo	1	12	6/13/2015	71.1	LENGTH	6.55	12	1	1
5	1372	12	Placebo	1	28	7/21/2016	81	HEIGHT		14.7	1	1
5	1372	24	Placebo	1	37	4/5/2017	87.9	HEIGHT	11.4	14.5	0	1
5	1372	36	Placebo	1	50	5/22/2018	93.6	HEIGHT	13.35	15	0	1
5	1374	0	Placebo	0	36	4/3/2015	84.3	HEIGHT	12.25	15.5	1	1
5	1374	12	Placebo	0	44	7/21/2016	96.6	HEIGHT		15.7	0	1
5	1374	24	Placebo	0	56	4/5/2017	100.6	HEIGHT	15.5	15	1	1
5	1374	36	Placebo	0	70	5/22/2018	106.8	HEIGHT	17.9	15	0	1
5	1374	48	Placebo	0	81	4/26/2019	111.6	HEIGHT	18.15	15	0	1
5	1374	60	Placebo	0	90	2/16/2020	116.3	HEIGHT	20.7	15.7	0	1
5	1377	0	Placebo	0	48	4/3/2015	102.4	HEIGHT	14.7	15.5	0	1
5	1377	48	Placebo	0	93	4/26/2019	112.7	HEIGHT	21.2	17.5	0	1
5	1378	0	Placebo	1	24	4/3/2015	83.8	HEIGHT	9.25	13	0	1
5	1378	12	Placebo	1	42	7/21/2016	92.9	HEIGHT		14.4	0	1
5	1378	24	Placebo	1	54	4/5/2017	100.5	HEIGHT	12.45	13.5	0	1
5	1378	36	Placebo	1	57	5/22/2018	106.5	HEIGHT	14.82	14	0	1
5	1378	48	Placebo	1	68	4/26/2019	114.1	HEIGHT	16.3	14	0	1
5	1378	60	Placebo	1	77	2/16/2020	119.4	HEIGHT	17.65	14.5	0	1
5	1381	0	Placebo	1	54	4/3/2015	105.2	HEIGHT	16.1	15.5	1	1
5	1381	12	Placebo	1	60	7/21/2016	110.3	HEIGHT		15.3	0	1
5	1381	24	Placebo	1	73	4/5/2017	113.4	HEIGHT	18.85	15.5	0	1

5	1381	36	Placebo	1	86	5/22/2018	118.5	HEIGHT	21.15	16		0	1
5	1381	48	Placebo	1	97	4/26/2019	121.7	HEIGHT	24.25	17.5		0	1
5	1381	60	Placebo	1	107	2/16/2020	126.6	HEIGHT	26.65	17.8		0	1
5	1383	12	Placebo	0	14	7/21/2016	79	HEIGHT		15.3		1	1
5	1383	48	Placebo	0	54	4/26/2019	99.6	HEIGHT	16.4	15.5		1	1
5	1384	0	Placebo	0	30	4/3/2015	83.9	HEIGHT	10.15	13.5		0	1
5	1384	24	Placebo	0	42	4/5/2017	99.6	HEIGHT	14.5	14.5		0	1
5	1384	36	Placebo	0	56	5/22/2018	107.3	HEIGHT	16.6	14.5		0	1
5	1384	48	Placebo	0	67	4/26/2019	111.7	HEIGHT	17.85	14		0	1
5	1384	60	Placebo	0	77	2/16/2020	114.9	HEIGHT	19.85	14.8		0	1
5	1387	0	Placebo	1	8	4/3/2015	67.9	HEIGHT	6.7	13	48	0	1
5	1387	24	Placebo	1	30	4/5/2017	83.1	HEIGHT	9.35	13	48	0	1
5	1389	0	Placebo	1	48	4/3/2015	93.8	HEIGHT	12.2	13		0	1
5	1389	12	Placebo	1	48	7/21/2016	100.8	HEIGHT		14.3		0	1
5	1389	24	Placebo	1	61	4/5/2017	104.1	HEIGHT	14.6	13.5		0	1
5	1389	48	Placebo	1	100	4/26/2019	115.9	HEIGHT	18	15		0	1
5	1389	60	Placebo	1	110	2/16/2020	120	HEIGHT	21.2	16		0	1
5	1391	12	Placebo	1	12	7/21/2016	76.1	HEIGHT		14		1	1
5	1391	24	Placebo	1	24	4/5/2017	83.3	HEIGHT	10.95	14		1	1
5	1391	48	Placebo	1	48	4/26/2019	100.7	HEIGHT	15.9	16		1	1
5	1392	24	Placebo	1	56	4/5/2017	102.6	HEIGHT	14.55	14.5		1	1
5	1393	12	Placebo	1	11	7/21/2016	72.8	HEIGHT		13.5		0	1
5	1393	36	Placebo	1	36	5/22/2018	87.9	HEIGHT	10.95	14		0	1
5	1393	48	Placebo	1	47	4/26/2019	94.7	HEIGHT	12.05	14		1	1
5	1393	60	Placebo	1	56	2/16/2020	99.9	HEIGHT	13	14		0	1
5	1398	0	Placebo	1	12	6/13/2015	77.5	LENGTH	8.3	13.5		0	1
5	1398	12	Placebo	1	20	7/21/2016	87.3	HEIGHT		16		0	1
5	1398	24	Placebo	1	32	4/5/2017	93.7	HEIGHT	12.3	15		0	1
5	1398	36	Placebo	1	56	5/22/2018	103	HEIGHT	14.9	15.5		0	1
5	1398	48	Placebo	1	67	4/26/2019	109.6	HEIGHT	16	15		0	1
5	1399	0	Placebo	1	30	6/13/2015	78.3	HEIGHT	9.5	14.5		1	1
5	1399	12	Placebo	1	34	7/21/2016	85	HEIGHT		15.4		1	1
5	1399	24	Placebo	1	42	4/5/2017	89.1	HEIGHT	13.9	16.5		0	1
5	1399	36	Placebo	1	56	5/22/2018	95.1	HEIGHT	14.65	15.5		0	1
5	1399	48	Placebo	1	66	4/26/2019	98.3	HEIGHT	15	16		0	1
5	1405	12	Placebo	1	44	7/21/2016	90	HEIGHT		14.6		1	1
5	1407	24	Placebo	0	42	4/5/2017	98.8	HEIGHT	13.95	15		1	1
5	1409	12	Placebo	0	7	7/21/2016	62.4	LENGTH		12.1		0	1
5	1409	36	Placebo	0	26	5/22/2018	79.9	HEIGHT	9.55	13		1	1
5	1409	48	Placebo	0	37	4/26/2019	87.6	HEIGHT	11.25	13		1	1

5	1410	12	Placebo	0	20	7/21/2016	79	HEIGHT		14.4		1	1
5	1410	36	Placebo	0	40	5/22/2018	90.9	HEIGHT	10.75	12.5		1	1
5	1411	12	Placebo	0	54	7/21/2016	108.7	HEIGHT		14.2		1	1
5	1412	12	Placebo	0	32	7/21/2016	86.6	HEIGHT		14.8		1	1
5	1413	12	Placebo	0	8	7/21/2016	68.3	LENGTH		13.8		0	1
5	1413	24	Placebo	0	15	4/5/2017	72.8	HEIGHT	8.65	14.5		0	1
5	1413	36	Placebo	0	28	5/22/2018	81.3	HEIGHT	11.5	15.5		0	1
5	1415	24	Placebo	1	26	4/5/2017	81.5	HEIGHT	10.35	14.5		1	1
5	1415	36	Placebo	1	41	5/22/2018	90.9	HEIGHT	12.4	14.5		1	1
5	1420	24	Placebo	1	3	4/5/2017	69.9	LENGTH	7.85	14.5		1	1
5	1420	36	Placebo	1	17	5/22/2018	83	HEIGHT	10.05	14		1	1
5	1421	0	Placebo	1	4	4/3/2015	63.8	LENGTH	6.15	13	60	1	1
5	1421	24	Placebo	1	30	4/5/2017	83.1	HEIGHT	10.45	14.5	60	1	1
5	1421	36	Placebo	1	44	5/22/2018	92.8	HEIGHT	12.85	14.5	60	0	1
5	1421	48	Placebo	1	55	4/26/2019	99.3	HEIGHT	13.85	14.5	60	0	1
5	1423	12	Placebo	1	9	7/21/2016	70.6	LENGTH		13.8		0	1
5	1423	24	Placebo	1	19	4/5/2017	77.4	LENGTH	8.9	14.5		0	1
5	1423	60	Placebo	1	53	2/16/2020	97.4	HEIGHT	14.25	14.2		1	1
5	1424	0	Placebo	0	24	4/3/2015	91.8	HEIGHT	12.75	15		1	1
5	1424	12	Placebo	0	42	7/21/2016	97.7	HEIGHT		14.4		0	1
5	1424	24	Placebo	0	54	4/5/2017	102.3	HEIGHT	14.9	14.5		0	1
5	1424	36	Placebo	0	68	5/22/2018	106.7	HEIGHT	16.25	14.5		0	1
5	1424	48	Placebo	0	79	4/26/2019	111.1	HEIGHT	17.85	14.5		0	1
5	1424	60	Placebo	0	89	2/16/2020	114.4	HEIGHT	19.2	14.5		0	1
5	1425	0	Placebo	0	12	4/3/2015	70.1	LENGTH	7.5	14		1	1
5	1426	12	Placebo	0	3	7/21/2016	68.9	LENGTH		13.3		0	1
5	1426	24	Placebo	0	15	4/5/2017	77.8	HEIGHT	9.95	14.5		0	1
5	1426	60	Placebo	0	49	2/16/2020	101.9	HEIGHT	16.85	16.3		0	1
5	1431	0	Placebo	0	36	4/3/2015	92.5	HEIGHT	14	15.5		1	1
5	1431	24	Placebo	0	56	4/5/2017	105	HEIGHT	16.9	16		1	1
5	1431	36	Placebo	0	70	5/22/2018	111.3	HEIGHT	18.05	15.5		0	1
5	1431	48	Placebo	0	81	4/26/2019	115.1	HEIGHT	19.9	16		0	1
5	1431	60	Placebo	0	90	2/16/2020	119.6	HEIGHT	22.65	16.8		0	1
5	1432	0	Placebo	1	24	4/3/2015	82.9	HEIGHT	9.8	13.5		0	1
5	1432	24	Placebo	1	44	4/5/2017	97.2	HEIGHT	15.5	17		1	1
5	1432	36	Placebo	1	58	5/22/2018	104.1	HEIGHT	16.15	16		0	1
5	1432	48	Placebo	1	69	4/26/2019	111.4	HEIGHT	17.75	16		0	1
5	1433	0	Placebo	1	42	4/3/2015	90.2	HEIGHT	13.55	16		0	1
5	1433	12	Placebo	1	50	7/21/2016	100.5	HEIGHT		17		0	1
5	1433	24	Placebo	1	62	4/5/2017	105.7	HEIGHT	17.35	16		0	1

5	1433	36	Placebo	1	76	5/22/2018	112.7	HEIGHT	18.85	16.5		0	1
5	1433	48	Placebo	1	87	4/26/2019	118.5	HEIGHT	21	17		0	1
5	1433	60	Placebo	1	96	2/16/2020	123.2	HEIGHT	24.1	17.2		0	1
5	1436	0	Placebo	0	24	4/3/2015	81.4	HEIGHT	10.55	14		0	1
5	1436	12	Placebo	0	32	7/21/2016	89.7	HEIGHT		16.1		0	1
5	1436	36	Placebo	0	58	5/22/2018	100.4	HEIGHT	15.5	15.5		0	1
5	1436	48	Placebo	0	69	4/26/2019	105.5	HEIGHT	16.05	14		0	1
5	1437	0	Placebo	0	6	4/3/2015	66.1	LENGTH	7.15	14		0	1
5	1437	12	Placebo	0	14	7/21/2016	79.5	HEIGHT		15.5		1	1
5	1437	24	Placebo	0	26	4/5/2017	85.6	HEIGHT	11.4	15.5		0	1
5	1437	36	Placebo	0	40	5/22/2018	96.9	HEIGHT	14.6	15.5		0	1
5	1437	48	Placebo	0	51	4/26/2019	103.1	HEIGHT	15.8	14		0	1
5	1439	12	Placebo	0	7	7/21/2016	74.3	LENGTH		13.3		1	1
5	1439	36	Placebo	0	28	5/22/2018	86.2	HEIGHT	11.7	13.5		1	1
5	1439	48	Placebo	0	39	4/26/2019	91.1	HEIGHT	11.65	13		0	1
5	1439	60	Placebo	0	49	2/16/2020	94.1	HEIGHT	12.5	13.3		0	1
5	1440	0	Placebo	0	36	4/3/2015	84.2	HEIGHT	11.65	14.5		1	1
5	1443	12	Placebo	1	18	7/21/2016	72.8	HEIGHT		14.2		1	1
5	1443	24	Placebo	1	27	4/5/2017	79.2	HEIGHT	10.3	14.5		0	1
5	1443	36	Placebo	1	41	5/22/2018	89.3	HEIGHT	12.15	15		0	1
5	1443	60	Placebo	1	61	2/16/2020	101.9	HEIGHT	15.4	15.1		0	1
5	1444	12	Placebo	1	10	7/21/2016	74.3	LENGTH		13		0	1
5	1444	36	Placebo	1	34	5/22/2018	88.2	HEIGHT	11.4	14.5		0	1
5	1444	60	Placebo	1	55	2/16/2020	100.5	HEIGHT	14.9	15		1	1
5	1445	0	Placebo	0	36	4/3/2015	80.2	HEIGHT	10.05	14.5		1	1
5	1445	12	Placebo	0	30	7/21/2016	91.5	HEIGHT		14.6		0	1
5	1445	24	Placebo	0	42	4/5/2017	96.1	HEIGHT	12.4	14.5		0	1
5	1445	36	Placebo	0	56	5/22/2018	103.4	HEIGHT	14.6	14.5		0	1
5	1445	48	Placebo	0	67	4/26/2019	107.5	HEIGHT	15.05	14.5		0	1
5	1445	60	Placebo	0	77	2/16/2020	112.5	HEIGHT	16.9	14.8		0	1
5	1446	0	Placebo	1	48	4/3/2015	102.3	HEIGHT	14.9	16		1	1
5	1446	12	Placebo	1	64	7/21/2016	109.6	HEIGHT		15.3		0	1
5	1446	36	Placebo	1	87	5/22/2018	119.4	HEIGHT	19.35	15.5		0	1
5	1446	48	Placebo	1	97	4/26/2019	123.8	HEIGHT	20.55	15.5		0	1
5	1446	60	Placebo	1	118	2/16/2020	127.5	HEIGHT	23.9	16		0	1
5	1449	12	Placebo	0	5	7/21/2016	69.4	LENGTH		13.2	30	0	1
5	1449	24	Placebo	0	15	4/5/2017	76.2	HEIGHT	8.95	13	30	0	1
5	1452	0	Placebo	0	48	4/3/2015	114.9	HEIGHT	20.7	16		0	1
5	1452	48	Placebo	0	91	4/26/2019	132.7	HEIGHT	29.55	18		0	1
5	1454	12	Placebo	1	10	7/21/2016	72.7	LENGTH		13.6		0	1

5	1454	24	Placebo	1	20	4/5/2017	75.7	HEIGHT	9.8	14		0	1
5	1454	60	Placebo	1	57	2/16/2020	97.3	HEIGHT	15.05	15.2		0	1
5	8014	36	Placebo	1	16	5/22/2018	75.6	LENGTH	8.6	13.5		1	1
5	8014	48	Placebo	1	26	4/26/2019	83.8	HEIGHT	10.95	14		1	1
5	8015	36	Placebo	0	58	5/22/2018	108.9	HEIGHT	18.05	15.5		1	1
5	8030	36	Placebo	0	18	5/22/2018	75.8	HEIGHT	8.05	12.5		1	1
5	8030	48	Placebo	0	29	4/26/2019	81.2	HEIGHT	9.65	13.5		1	1
5	8030	60	Placebo	0	39	2/16/2020	85.9	HEIGHT	11.2	14.2		1	1
5	8049	36	Placebo	1	15	5/22/2018	74	LENGTH	8.25	13		1	1
5	8053	48	Placebo	1	12	4/26/2019	74.5	LENGTH	8.6	13.5		1	1
5	8059	36	Placebo	1	39	5/22/2018	91.4	HEIGHT	12.45	15		1	1
5	8075	60	Placebo	1	8	2/20/2020	63.5	HEIGHT	5.9	12.3		1	1
5	8102	36	Placebo	1	9	5/22/2018	70.8	LENGTH	8.5	15.5		1	1
5	8102	48	Placebo	1	20	4/26/2019	80.5	LENGTH	10.35	14		0	1
5	8111	48	Placebo	1	51	4/26/2019	95.9	HEIGHT	12.7	13		1	1
5	8151	60	Placebo	1	4	2/16/2020	65.5	HEIGHT	6.9	13.3		1	1
5	8157	60	Placebo	1	54	2/16/2020	101.1	HEIGHT	14.5	15.5		1	1
5	8168	48	Placebo	0	12	4/26/2019	70.9	LENGTH	8.7	14.5		1	1
5	8168	60	Placebo	0	21	2/16/2020	80.4	HEIGHT	9.5	13.7		0	1
5	8181	48	Placebo	0	29	4/26/2019	84.3	LENGTH	12.05	16.5		1	1
5	8181	60	Placebo	0	38	2/16/2020	90	HEIGHT	13.9	17.5		1	1
5	8190	48	Placebo	0	35	4/26/2019	89	HEIGHT	11.15	14		1	1
5	8204	48	Placebo	1	57	4/26/2019	92.6	HEIGHT	13	13		1	1
5	8204	60	Placebo	1	56	2/20/2020	99.1	HEIGHT	15.15	13.5		1	1
5	8227	48	Placebo	0	39	4/26/2019	92.1	HEIGHT	12.95	15.5		1	1
5	8240	48	Placebo	1	39	4/26/2019	93.8	HEIGHT	11.95	13		1	1
5	8290	36	Placebo	0	12	5/22/2018	72.5	LENGTH	7.9	13.5		1	1
5	8290	48	Placebo	0	23	4/26/2019	83.8	LENGTH	10.45	14.5		0	1
5	8290	60	Placebo	0	33	2/16/2020	89.6	HEIGHT	13.9	16.2		0	1
5	8309	36	Placebo	0	34	5/22/2018	94.1	HEIGHT	15.3	16.5		1	1
5	8309	60	Placebo	1	54	2/16/2020	106.8	HEIGHT	17.8	15.5		1	1
5	8315	36	Placebo	0	6	5/22/2018	64.6	LENGTH	5.65	11		1	1
5	8315	48	Placebo	0	15	4/26/2019	74.9	LENGTH	8.9	14		0	1
5	8315	60	Placebo	0	36	2/16/2020	82.6	HEIGHT	10.95	14.6		0	1
5	8317	36	Placebo	1	50	5/22/2018	96.3	HEIGHT	13.25	14		1	1
5	8369	60	Placebo	0	11	2/16/2020	72.5	LENGTH	7.65	13		1	1
5	8395	36	Placebo	1	2	5/22/2018	57.2	LENGTH	5.8	12.5	42	1	1
5	8420	60	Placebo	0	38	2/16/2020	78.5	HEIGHT	10.95	14		1	1
5	8453	36	Placebo	1	13	5/22/2018	69	LENGTH	6.3	11.5		1	1
5	8467	60	Placebo	1	28	2/16/2020	79.6	HEIGHT	9.7	12.8		1	1

5	8505	60	Placebo	0	21	2/16/2020	81.5	HEIGHT	10.05	13.6		1	1
5	8513	48	Placebo	1	2	4/26/2019	51.8	LENGTH	4.1	12		1	1
5	8513	60	Placebo	1	11	2/16/2020	67.5	LENGTH	7.65	13.5		0	1
5	8524	36	Placebo	1	14	5/22/2018	73.2	LENGTH	8.4	13		1	1
5	8524	60	Placebo	1	34	2/20/2020	88.8	HEIGHT	12.1	14		1	1
5	8546	60	Placebo	1	3	2/16/2020	64.2	LENGTH	7.55	14		1	1
5	8560	60	Placebo	1	31	2/16/2020	83.9	HEIGHT	9.65	12.9		1	1
5	8565	60	Placebo	0	35	2/16/2020	91.6	HEIGHT	13.75	14.5		1	1
5	8568	48	Placebo	1	11	4/26/2019	66.6	LENGTH	6.95	13		1	1
5	8630	48	Placebo	0	52	4/26/2019	107.5	HEIGHT	15.95	15		1	1
5	8632	48	Placebo	1	8	4/26/2019	64.2	LENGTH	6.1	12		1	1
5	8632	60	Placebo	1	15	2/16/2020	73.1	LENGTH	8.4	13		0	1
5	8650	48	Placebo	0	1	4/26/2019	59.5	LENGTH	6.3	14		1	1
5	8655	48	Placebo	1	4	4/26/2019	63.8	LENGTH	7.5	14		1	1
5	8655	60	Placebo	1	12	2/16/2020	75.4	HEIGHT	9.95	14.3		0	1
5	8672	36	Placebo	0	17	5/22/2018	73.5	LENGTH	8.65	13.5		1	1
5	8684	60	Placebo	0	27	2/16/2020	82.1	HEIGHT	11.25	14		1	1
5	8715	48	Placebo	1	55	4/26/2019	97.6	HEIGHT	14.65	15.5		1	1
5	8720	60	Placebo	0	55	2/16/2020	103.5	HEIGHT	17.55	15.6		1	1
5	8730	36	Placebo	1	3	5/22/2018	70.3	LENGTH	6.7	12		1	1
5	8730	48	Placebo	1	21	4/26/2019	79.7	LENGTH	8.15	12		0	1
5	8730	60	Placebo	1	31	2/16/2020	85.1	HEIGHT	10.1	12.2		0	1
5	8737	48	Placebo	1	16	4/26/2019	69.9	LENGTH	7.35	12.5		1	1
5	8758	36	Placebo	0	15	5/22/2018	75	LENGTH	9.3	13.5	48	1	1
5	8784	60	Placebo	1	6	2/16/2020	63.2	LENGTH	6.45	12.7		1	1
5	8813	36	Placebo	0	10	5/22/2018	69	LENGTH	6.9	12		1	1
5	8813	48	Placebo	0	21	4/26/2019	76.7	LENGTH	9.35	14		1	1
5	8814	48	Placebo	0	3	4/26/2019	64.9	LENGTH	7.55	14		1	1
5	8842	60	Placebo	1	42	2/16/2020	99.4	HEIGHT	16.25	14.7		1	1
5	8847	36	Placebo	0	15	5/22/2018	74	HEIGHT	9.3	14		1	1
5	8859	60	Placebo	0	24	2/16/2020	78.8	LENGTH	9.65	13.6		1	1
5	9028	36	Placebo	1	28	5/22/2018	88.1	HEIGHT	10.65	12.5		1	1
5	9037	48	Placebo	1	12	4/26/2019	74.4	LENGTH	7.95	12		1	1
5	9058	60	Placebo	1	16	2/16/2020	73.4	LENGTH	8.9	13.5		1	1
5	9084	48	Placebo	0	48	4/26/2019	97.6	HEIGHT	13.2	13		1	1
5	9099	36	Placebo	1	6	5/22/2018	63	LENGTH	5.3	11.5		1	1
5	9099	48	Placebo	1	16	4/26/2019	75.4	LENGTH	8.3	13.5		0	1
5	9099	60	Placebo	1	25	2/16/2020	85.1	HEIGHT	9.45	12.6		0	1
5	9100	60	Placebo	0	40	2/16/2020	90.6	HEIGHT	13.45	15		1	1
5	9116	36	Placebo	1	8	5/22/2018	64.3	LENGTH	6.25	12.5		1	1

5	9130	36	Placebo	1	56	5/22/2018	97.7	HEIGHT	14.05	14.5		1	1
5	9138	48	Placebo	0	51	4/26/2019	95.4	HEIGHT	15.45	16		1	1
5	9245	48	Placebo	1	58	4/26/2019	100.4	HEIGHT	14.1	13		1	1
5	9265	36	Placebo	1	40	5/22/2018	86.6	HEIGHT	10.8	14.5		1	1
5	9266	60	Placebo	0	10	2/16/2020	69.8	LENGTH	8.25	14		1	1
5	9313	48	Placebo	0	16	4/26/2019	80.3	LENGTH	11.5	16		1	1
5	9313	60	Placebo	0	26	2/16/2020	87.6	HEIGHT	14	15.5		1	1
5	9316	60	Placebo	0	44	2/16/2020	96.2	HEIGHT	16.15	15.2		1	1
5	9318	60	Placebo	1	44	2/20/2020	90.2	HEIGHT	12.35	14.2		1	1
5	9340	36	Placebo	0	15	5/22/2018	73.9	LENGTH	9.3	16	54	1	1
5	9362	60	Placebo	1	41	2/20/2020	105.5	HEIGHT	20.35	16.7		1	1
5	9368	60	Placebo	1	18	2/20/2020	85.6	HEIGHT	9.65	14.4		1	1
5	9405	48	Placebo	1	10	4/26/2019	67.5	LENGTH	7.5	13.5		1	1
5	9405	60	Placebo	1	20	2/16/2020	76.2	HEIGHT	9.7	14.3		0	1
5	9435	48	Placebo	0	49	4/26/2019	95.4	HEIGHT	13.2	14.5		1	1
5	9435	60	Placebo	0	58	2/16/2020	102	HEIGHT	15	14.5		1	1
5	9453	36	Placebo	1	51	5/22/2018	86.8	HEIGHT	13.75	16.5		1	1
5	9475	48	Placebo	1	58	4/26/2019	102.2	HEIGHT	14.6	14.5		1	1
5	9494	48	Placebo	1	5	4/26/2019	62	LENGTH	6.35	12.5		1	1
5	9494	60	Placebo	1	15	2/16/2020	72.6	HEIGHT	7.85	13		0	1
5	9497	60	Placebo	0	54	2/20/2020	103.1	HEIGHT	16.5	15		1	1
5	9507	48	Placebo	1	51	4/26/2019	101.2	HEIGHT	13.5	14		1	1
5	9520	60	Placebo	0	7	2/16/2020	69.9	LENGTH	7.1	12.4		1	1
5	9523	48	Placebo	0	22	4/26/2019	79.4	LENGTH	10.4	14.5		1	1
5	9528	60	Placebo	1	1	2/16/2020	58.3	LENGTH	5.65	12.1		1	1
5	9543	36	Placebo	1	9	5/22/2018	65.8	LENGTH	6.3	11.5		1	1
5	9543	60	Placebo	1	29	2/16/2020	84.5	HEIGHT	10.2	12.8		0	1
5	9548	36	Placebo	1	9	5/22/2018	67	LENGTH	7.75	14		1	1
5	9548	48	Placebo	1	20	4/26/2019	79.7	HEIGHT	10.3	15		0	1
5	9548	60	Placebo	1	30	2/16/2020	83.2	HEIGHT	11.45	15		1	1
5	9556	48	Placebo	1	15	4/26/2019	80.8	LENGTH	10.25	12.5		1	1
5	9567	36	Placebo	0	125	5/22/2018	136.7	HEIGHT	31.55	18.5		0	1
5	9567	48	Placebo	0	136	4/26/2019	140.2	HEIGHT	34.95	18		0	1
5	9568	48	Placebo	1	37	4/26/2019	87.6	HEIGHT	12.1	14		1	1
6	1469	0	Placebo	1	24	3/27/2015	75.3	HEIGHT	7.8	12		1	1
6	1469	12	Placebo	1	42	7/9/2016	84.6	HEIGHT	9.7	13.5		0	1
6	1469	24	Placebo	1	53	3/23/2017	89	HEIGHT	10.8	13		0	1
6	1469	36	Placebo	1	68	5/21/2018	96	HEIGHT	12.2	13.5		0	1
6	1469	48	Placebo	1	79	5/25/2019	101.7	HEIGHT	13.27272727	13.5		0	1
6	1469	60	Placebo	1	88	2/17/2020	106.9	HEIGHT	15.1	14		0	1

6	1470	0	Placebo	0	24	3/27/2015	84.4	HEIGHT	11.1	14	18	0	1
6	1471	0	Placebo	0	12	3/27/2015	71.5	LENGTH	8.25	14.5		1	1
6	1471	48	Placebo	0	55	5/25/2019	101.6	HEIGHT	15	14.5		0	1
6	1472	0	Placebo	1	36	3/27/2015	87.3	HEIGHT	12.35	15.5		0	1
6	1472	24	Placebo	1	60	3/23/2017	103.6	HEIGHT	16.35	15		0	1
6	1472	48	Placebo	1	85	5/25/2019	115.2	HEIGHT	20.77272727	16		0	1
6	1472	60	Placebo	1	95	2/17/2020	119.1	HEIGHT	20.9	16.8		0	1
6	1477	24	Placebo	1	47	3/26/2017	98.6	HEIGHT	14.5	15.5		1	1
6	1478	12	Placebo	1	54	7/15/2016	109.5	HEIGHT	15.8	14		1	1
6	1479	0	Placebo	1	48	3/27/2015	112.9	HEIGHT	16.3	14.5		0	1
6	1479	24	Placebo	1	101	3/23/2017	124	HEIGHT	19.75	15		0	1
6	1479	48	Placebo	1	127	5/25/2019	135.9	HEIGHT	25.31818182	17		0	1
6	1480	12	Placebo	1	7	7/9/2016	66.8	LENGTH	6.05	11.5		0	1
6	1480	24	Placebo	1	14	3/23/2017	74.3	HEIGHT	7.5	11		1	1
6	1480	60	Placebo	1	53	2/17/2020	98.4	HEIGHT	12.15	13		1	1
6	1481	0	Placebo	0	36	3/27/2015	87.1	HEIGHT	12.2	14		1	1
6	1481	12	Placebo	0	66	7/9/2016	97.2	HEIGHT	13.35	14		0	1
6	1481	24	Placebo	0	77	3/23/2017	101	HEIGHT	15	14		0	1
6	1481	36	Placebo	0	66	5/21/2018	107.6	HEIGHT	15.85	14		0	1
6	1481	48	Placebo	0	77	5/25/2019	112.5	HEIGHT	18.36363636	15		0	1
6	1481	60	Placebo	0	87	2/17/2020	115.5	HEIGHT	19.35	15		0	1
6	1482	0	Placebo	0	54	3/27/2015	123.9	HEIGHT	21.3	15		0	1
6	1482	12	Placebo	0	126	7/15/2016	127	HEIGHT	23.25	16		0	1
6	1483	12	Placebo	1	50	7/9/2016	111.3	HEIGHT	17.6	16		1	1
6	1485	0	Placebo	0	18	3/27/2015	80.8	HEIGHT	10.4	15		1	1
6	1485	36	Placebo	0	56	5/21/2018	103.3	HEIGHT	16.6	15.5		0	1
6	1485	48	Placebo	0	67	5/25/2019	108.2	HEIGHT	18.27272727	15		0	1
6	1491	0	Placebo	0	24	3/27/2015	77.8	LENGTH	9.25	13		1	1
6	1491	12	Placebo	1	30	7/9/2016	86.8	HEIGHT	12.4	14		0	1
6	1491	24	Placebo	1	41	3/23/2017	94.2	HEIGHT	13.4	13.5		1	1
6	1494	0	Placebo	0	48	3/27/2015	101.5	HEIGHT	14.55	15.5		1	1
6	1494	24	Placebo	0	77	5/16/2017	114.2	HEIGHT	18.5	16.5		0	1
6	1494	48	Placebo	0	80	5/25/2019	124.3	HEIGHT	22.86363636	17		0	1
6	1494	60	Placebo	0	89	2/17/2020	128	HEIGHT	23.25	17		0	1
6	1496	0	Placebo	0	36	3/27/2015	108.3	HEIGHT	16.05	14		0	1
6	1496	12	Placebo	0	52	7/10/2016	116	HEIGHT	19.05	14		0	1
6	1496	24	Placebo	0	60	3/23/2017	120.2	HEIGHT	21.1	15		0	1
6	1496	48	Placebo	0	85	5/25/2019	131.3	HEIGHT	24.36363636	15		0	1
6	1496	60	Placebo	0	95	2/17/2020	133.7	HEIGHT	26.65	15.1		0	1
6	1497	0	Placebo	0	36	3/27/2015	94.1	HEIGHT	12.85	13.5		0	1

6	1498	24	Placebo	1	10	5/16/2017	66.3	LENGTH	5.15	10		1	1
6	1498	48	Placebo	1	36	5/25/2019	80.6	HEIGHT	7.636363636	11		1	1
6	1499	0	Placebo	0	12	3/27/2015	77.6	LENGTH	9.8	14		1	1
6	1499	12	Placebo	0	30	7/9/2016	89.4	HEIGHT	13.6	15.5		1	1
6	1501	12	Placebo	1	3	7/9/2016	63.1	LENGTH	5.95	12.5		0	1
6	1501	24	Placebo	1	12	3/23/2017	70.2	LENGTH	7.35	12.5		0	1
6	1501	36	Placebo	1	26	5/21/2018	82	HEIGHT	9.55	13.5		0	1
6	1501	60	Placebo	1	48	2/17/2020	94.5	HEIGHT	13.65	14		1	1
6	1502	0	Placebo	1	48	3/27/2015	107.2	HEIGHT	17.8	16		1	1
6	1505	0	Placebo	0	30	3/27/2015	82.2	HEIGHT	10.2	13.5		0	1
6	1505	24	Placebo	0	49	5/16/2017	96.5	HEIGHT	12.9	14.5		0	1
6	1505	36	Placebo	0	64	5/21/2018	103.4	HEIGHT	14.2	14		0	1
6	1505	48	Placebo	0	75	5/25/2019	108	HEIGHT	17.27272727	14.5		0	1
6	1506	0	Placebo	0	3	3/27/2015	63.8	LENGTH	7.75	15		0	1
6	1506	12	Placebo	0	15	7/10/2016	76	LENGTH	7.4	11.5		1	1
6	1506	24	Placebo	0	24	5/16/2017	82.4	HEIGHT	10.6	13		1	1
6	1506	36	Placebo	0	38	5/21/2018	93	HEIGHT	14.55	15		1	1
6	1506	60	Placebo	0	59	2/17/2020	107.9	HEIGHT	18.65	15		0	1
6	1507	0	Placebo	1	48	3/27/2015	98.6	HEIGHT	15.25	15		0	1
6	1507	12	Placebo	1	59	7/9/2016	108.7	HEIGHT	17.05	15		0	1
6	1507	24	Placebo	1	68	3/23/2017	115.7	HEIGHT	19.4	15		0	1
6	1507	36	Placebo	1	83	5/21/2018	122	HEIGHT	21.9	15.5		0	1
6	1507	48	Placebo	1	94	5/25/2019	126.9	HEIGHT	23.54545455	15		0	1
6	1507	60	Placebo	1	103	2/17/2020	130.5	HEIGHT	25.6	16.5		0	1
6	1508	0	Placebo	1	36	3/27/2015	90.5	HEIGHT	13.5	15	6	1	1
6	1509	12	Placebo	0	28	7/15/2016	91.1	HEIGHT	14.05	16		1	1
6	1510	12	Placebo	1	10	7/9/2016	69.3	LENGTH	7	13		0	1
6	1510	24	Placebo	1	21	5/16/2017	76.9	HEIGHT	8.35	13		0	1
6	1510	36	Placebo	1	36	5/21/2018	85.8	HEIGHT	10.05	14		0	1
6	1510	60	Placebo	1	56	2/17/2020	98.2	HEIGHT	12.75	14		0	1
6	1511	12	Placebo	1	50	7/9/2016	101.1	HEIGHT	14.85	15		1	1
6	1512	12	Placebo	1	12	7/15/2016	70.6	LENGTH	6.55	12		1	1
6	1512	48	Placebo	1	48	5/25/2019	92.5	HEIGHT	12	14		0	1
6	1516	0	Placebo	0	36	3/27/2015	102.5	HEIGHT	17.6	15.5	48	0	1
6	1516	12	Placebo	0	42	7/15/2016	115.5	HEIGHT	21.7	16.5	48	0	1
6	1516	24	Placebo	0	53	3/26/2017	121.2	HEIGHT	22.85	16	48	1	1
6	1516	36	Placebo	0	84	5/21/2018	128.1	HEIGHT	26.15	16.5	48	0	1
6	1517	12	Placebo	1	7	7/10/2016	69.9	LENGTH	6.65	12		1	1
6	1517	24	Placebo	1	14	5/16/2017	78.7	HEIGHT	8.85	12.5		0	1
6	1517	36	Placebo	1	32	5/21/2018	85.5	HEIGHT	10.75	13		0	1

6	1517	48	Placebo	1	43	5/25/2019	96.1	HEIGHT	12	13	0	1
6	1518	12	Placebo	0	3	7/9/2016	63.9	LENGTH	6.3	13.5	0	1
6	1518	24	Placebo	0	12	3/23/2017	74.4	LENGTH	8.55	14	1	1
6	1518	36	Placebo	0	28	5/21/2018	83.7	HEIGHT	11.4	15.5	1	1
6	1518	48	Placebo	0	38	5/25/2019	92.8	HEIGHT	14.40909091	15	0	1
6	1518	60	Placebo	0	48	2/17/2020	99.4	HEIGHT	15.8	15.5	0	1
6	1520	0	Placebo	1	48	3/27/2015	107.5	HEIGHT	16.15	14.5	0	1
6	1520	12	Placebo	1	61	7/10/2016	114.6	HEIGHT	17.9	15	0	1
6	1520	36	Placebo	1	87	5/21/2018	124.7	HEIGHT	21.5	16.5	0	1
6	1520	48	Placebo	1	97	5/25/2019	129.1	HEIGHT	24.31818182	17	0	1
6	1520	60	Placebo	1	107	2/17/2020	133.1	HEIGHT	24.95	16.7	0	1
6	1521	0	Placebo	1	48	3/27/2015	98.2	HEIGHT	15.25	15	1	1
6	1521	12	Placebo	1	68	7/10/2016	107.3	HEIGHT	17.4	15.5	0	1
6	1521	60	Placebo	1	115	2/17/2020	124.9	HEIGHT	25.65	16.8	0	1
6	1523	12	Placebo	0	10	7/9/2016	75.1	HEIGHT	8.85	14.5	0	1
6	1523	24	Placebo	0	18	3/23/2017	80.7	HEIGHT	10.35	14	0	1
6	1523	36	Placebo	0	33	5/21/2018	90.1	HEIGHT	14.4	17.5	0	1
6	1523	48	Placebo	0	44	5/25/2019	98.9	HEIGHT	16.77272727	16	1	1
6	1523	60	Placebo	0	53	2/17/2020	103.8	HEIGHT	18.45	16.3	1	1
6	1526	0	Placebo	0	8	3/27/2015	66.1	LENGTH	7.4	14	1	1
6	1526	24	Placebo	0	26	3/26/2017	84.3	HEIGHT	11.2	14	1	1
6	1526	48	Placebo	0	52	5/25/2019	97.3	HEIGHT	14.5	14	1	1
6	1527	0	Placebo	1	36	3/27/2015	95.3	HEIGHT	13.55	14	0	1
6	1527	12	Placebo	1	54	7/9/2016	105.3	HEIGHT	15.9	14.5	1	1
6	1527	24	Placebo	1	65	3/23/2017	109.5	HEIGHT	18.15	15	0	1
6	1527	36	Placebo	1	80	5/21/2018	116.6	HEIGHT	19.75	14.5	0	1
6	1527	48	Placebo	1	91	5/25/2019	121.3	HEIGHT	21.68181818	15.5	0	1
6	1527	60	Placebo	1	100	2/17/2020	125.1	HEIGHT	23.35	15.4	0	1
6	1530	12	Placebo	1	8	7/9/2016	70.3	HEIGHT	7.7	14	0	1
6	1530	48	Placebo	1	45	6/6/2019	94	HEIGHT	14.27272727	15	1	1
6	1531	0	Placebo	1	54	3/27/2015	93.7	HEIGHT	13.15	14.5	1	1
6	1531	24	Placebo	1	62	3/23/2017	105.3	HEIGHT	16.15	14.5	0	1
6	1532	12	Placebo	1	34	7/15/2016	90.6	HEIGHT	11.45	13.5	1	1
6	1533	0	Placebo	0	24	3/27/2015	100.5	HEIGHT	16.05	14.5	1	1
6	1534	12	Placebo	0	8	7/10/2016	72.2	LENGTH	7.6	14	1	1
6	1534	24	Placebo	0	20	3/23/2017	81.8	LENGTH	9.65	14.5	1	1
6	1534	48	Placebo	0	44	5/25/2019	93.5	HEIGHT	12	13.5	1	1
6	1535	0	Placebo	0	2	3/27/2015	63.6	LENGTH	6.15	13	1	1
6	1538	12	Placebo	0	42	7/15/2016	101.4	HEIGHT	15.85	16	0	1
6	1538	24	Placebo	0	53	3/23/2017	103.9	HEIGHT	16.9	16	1	1

6	1543	12	Placebo	1	13	7/9/2016	74.8	LENGTH	9.65	15.5		0	1
6	1543	24	Placebo	1	20	3/23/2017	83.2	LENGTH	11.2	15		1	1
6	1543	60	Placebo	1	55	2/17/2020	105.2	HEIGHT	16	14.7		0	1
6	1544	0	Placebo	1	36	3/27/2015	92.7	HEIGHT	11.45	14.5		1	1
6	1544	24	Placebo	1	56	3/23/2017	104.3	HEIGHT	14.22	15		0	1
6	1544	36	Placebo	1	71	5/21/2018	111.1	HEIGHT	16	15		0	1
6	1544	48	Placebo	1	82	5/25/2019	115.8	HEIGHT	16.90909091	14		0	1
6	1544	60	Placebo	1	91	2/17/2020	118.5	HEIGHT	18.6	14.8		0	1
6	1545	0	Placebo	1	54	3/27/2015	114	HEIGHT	17.9	13.5		1	1
6	1545	12	Placebo	1	90	7/9/2016	120.3	HEIGHT	20.15	14.5		0	1
6	1545	24	Placebo	1	101	3/23/2017	124.2	HEIGHT	22.35	15		0	1
6	1545	36	Placebo	1	116	5/21/2018	130.5	HEIGHT	24.55	15		0	1
6	1545	48	Placebo	1	127	5/25/2019	135.7	HEIGHT	27.09090909	16		0	1
6	1545	60	Placebo	1	136	2/17/2020	138.3	HEIGHT	28.35	16.7		0	1
6	1546	0	Placebo	1	24	3/27/2015	85.3	HEIGHT	10.75	13		0	1
6	1546	12	Placebo	1	36	7/15/2016	97.3	HEIGHT	13.9	14		0	1
6	1546	24	Placebo	1	47	3/23/2017	101.7	HEIGHT	14.8	14		0	1
6	1546	36	Placebo	1	62	5/21/2018	108.7	HEIGHT	15.8	14.5		0	1
6	1546	48	Placebo	1	73	5/25/2019	113.4	HEIGHT	17.40909091	14		0	1
6	1546	60	Placebo	1	82	2/17/2020	117.3	HEIGHT	18.15	14.9		0	1
6	1548	0	Placebo	1	11	3/27/2015	68.3	LENGTH	7.05	12.5		1	1
6	1548	36	Placebo	1	50	5/21/2018	94.2	HEIGHT	12.9	14		0	1
6	1548	48	Placebo	1	61	5/25/2019	101.4	HEIGHT	14.27272727	14		0	1
6	1548	60	Placebo	1	70	2/17/2020	106.4	HEIGHT	15.9	14.8		0	1
6	1553	12	Placebo	0	2	7/10/2016	68.3	LENGTH	8.5	14.5		0	1
6	1553	24	Placebo	0	12	3/23/2017	76.6	LENGTH	9.95	14		0	1
6	1553	36	Placebo	0	26	5/21/2018	84.6	HEIGHT	11.5	14		0	1
6	1553	48	Placebo	0	37	5/25/2019	90.6	HEIGHT	14.04545455	14.5		1	1
6	1553	60	Placebo	0	47	2/17/2020	95.1	HEIGHT	15.3	15		1	1
6	1554	0	Placebo	0	24	3/27/2015	83.8	HEIGHT	10.6	14		0	1
6	1554	12	Placebo	0	33	7/9/2016	89.3	HEIGHT	13.65	15.5		0	1
6	1554	24	Placebo	0	44	3/23/2017	94.1	HEIGHT	13.9	14.5		0	1
6	1554	36	Placebo	0	59	5/21/2018	101.5	HEIGHT	16.45	15.5		1	1
6	1555	0	Placebo	1	18	3/27/2015	76.8	LENGTH	9.25	13.5		0	1
6	1555	24	Placebo	0	41	3/26/2017	96.8	HEIGHT	13.35	14		0	1
6	1557	24	Placebo	1	6	3/23/2017	66.6	LENGTH	7.65	14		1	1
6	1557	48	Placebo	1	31	5/25/2019	84.8	LENGTH	11.5	14		0	1
6	1558	0	Placebo	0	36	3/27/2015	83.9	HEIGHT	11.15	13.5	24	1	1
6	1558	12	Placebo	0	47	7/9/2016	93.4	HEIGHT	14.3	15	24	0	1
6	1559	24	Placebo	0	54	3/23/2017	102.2	HEIGHT	18	14.5		1	1

6	1561	24	Placebo	1	48	5/16/2017	103.1	HEIGHT	15.3	15.5	0	1
6	1561	36	Placebo	1	91	5/21/2018	107.5	HEIGHT	16.6	15	0	1
6	1563	12	Placebo	0	45	7/9/2016	99.3	HEIGHT	15.5	16.5	1	1
6	1563	24	Placebo	0	56	5/16/2017	106.3	HEIGHT	17.35	17	1	1
6	1564	0	Placebo	0	36	3/27/2015	89.8	HEIGHT	14.15	17	1	1
6	1564	12	Placebo	0	45	7/9/2016	98.4	HEIGHT	16.8	16.5	1	1
6	1567	0	Placebo	0	24	3/27/2015	81.4	HEIGHT	9.8	13	1	1
6	1567	12	Placebo	0	30	7/15/2016	93	HEIGHT	11.75	14.5	1	1
6	1567	24	Placebo	0	41	3/23/2017	98.9	HEIGHT	13.85	15	0	1
6	1567	48	Placebo	0	67	5/25/2019	111.2	HEIGHT	16.95454545	14	0	1
6	1567	60	Placebo	0	76	2/17/2020	116	HEIGHT	18.35	15	0	1
6	1568	24	Placebo	1	72	5/16/2017	116.4	HEIGHT	20.55	16	0	1
6	1568	36	Placebo	1	87	5/21/2018	121.2	HEIGHT	22.25	16.5	0	1
6	1569	0	Placebo	1	11	3/27/2015	65.8	LENGTH	6	12.5	0	1
6	1569	24	Placebo	1	32	3/26/2017	81.5	HEIGHT	9.85	13.5	0	1
6	1569	48	Placebo	1	58	5/25/2019	96.7	HEIGHT	12.90909091	14	0	1
6	1569	60	Placebo	1	67	2/17/2020	100.2	HEIGHT	14.4	14.2	0	1
6	1573	12	Placebo	0	11	7/10/2016	76.9	HEIGHT	9.45	14	1	1
6	1575	0	Placebo	0	54	3/27/2015	119.5	HEIGHT	20.4	15.5	0	1
6	1575	24	Placebo	0	78	3/23/2017	128.6	HEIGHT	25.1	17	0	1
6	1575	60	Placebo	0	113	2/17/2020	141	HEIGHT	30.05	18.5	0	1
6	1576	0	Placebo	0	24	3/27/2015	85.2	HEIGHT	12.4	16.5	1	1
6	1576	12	Placebo	0	33	7/9/2016	97.3	HEIGHT	17.05	18	0	1
6	1576	24	Placebo	0	44	3/23/2017	103.3	HEIGHT	18.05	17	1	1
6	1576	36	Placebo	0	59	5/21/2018	110.4	HEIGHT	19.1	16.5	0	1
6	1576	48	Placebo	0	70	5/25/2019	116.5	HEIGHT	21.36363636	16.5	0	1
6	1576	60	Placebo	0	79	2/17/2020	120.1	HEIGHT	22.65	16.6	0	1
6	1577	0	Placebo	1	48	3/27/2015	97.8	HEIGHT	13.45	14.5	0	1
6	1577	12	Placebo	1	54	7/10/2016	104.9	HEIGHT	15.7	14.5	0	1
6	1577	24	Placebo	0	65	3/23/2017	108.9	HEIGHT	15.95	13.5	0	1
6	1577	36	Placebo	0	80	5/21/2018	112.7	HEIGHT	18.45	14.5	0	1
6	1577	48	Placebo	0	91	5/25/2019	118	HEIGHT	19.86363636	15	0	1
6	1577	60	Placebo	0	100	2/17/2020	121.1	HEIGHT	20.45	15.1	0	1
6	1579	0	Placebo	1	24	3/27/2015	76.7	HEIGHT	8.65	13.5	1	1
6	1579	48	Placebo	1	69	5/25/2019	106	HEIGHT	14.59090909	14	0	1
6	1579	60	Placebo	1	79	2/17/2020	109.6	HEIGHT	16.05	14.5	0	1
6	1582	0	Placebo	1	24	3/27/2015	90.2	HEIGHT	12.55	14.5	0	1
6	1582	12	Placebo	1	48	7/15/2016	101.6	HEIGHT	14.15	14.5	0	1
6	1582	24	Placebo	1	59	3/26/2017	104.9	HEIGHT	15.2	14	0	1
6	1582	36	Placebo	1	74	5/21/2018	111.9	HEIGHT	16.55	14.5	0	1

6	1582	48	Placebo	1	85	5/25/2019	116.8	HEIGHT	18.59090909	14		0	1
6	1584	12	Placebo	1	3	7/9/2016	57.1	LENGTH	4.15	10.5		1	1
6	1584	24	Placebo	1	14	3/23/2017	64.8	LENGTH	6.6	12.5		1	1
6	1584	36	Placebo	1	28	5/21/2018	76.5	LENGTH	8.5	13		1	1
6	1584	48	Placebo	1	39	5/25/2019	83.5	HEIGHT	10.95454545	14.5		1	1
6	1584	60	Placebo	1	49	2/17/2020	91.1	HEIGHT	12.3	14.7		0	1
6	1586	0	Placebo	0	24	3/27/2015	75.8	LENGTH	7.9	13		0	1
6	1586	24	Placebo	0	41	3/23/2017	92.4	HEIGHT	12	13.5		1	1
6	1586	48	Placebo	0	67	5/25/2019	107	HEIGHT	15.90909091	15		0	1
6	1586	60	Placebo	0	76	2/17/2020	110.3	HEIGHT	16.65	14.7		0	1
6	1588	12	Placebo	1	1	7/10/2016	60.3	LENGTH	6.7	13.5	24	1	1
6	1590	12	Placebo	0	11	7/9/2016	75	HEIGHT	9	13		0	1
6	1590	24	Placebo	0	20	3/26/2017	80	HEIGHT	10.1	12		0	1
6	1590	36	Placebo	0	34	5/21/2018	91.7	HEIGHT	14	14		0	1
6	1590	48	Placebo	0	45	5/25/2019	100.1	HEIGHT	16	14		1	1
6	1590	60	Placebo	0	55	2/17/2020	105.7	HEIGHT	16.75	13.8		0	1
6	1591	0	Placebo	0	36	3/27/2015	102.1	HEIGHT	16.35	16		0	1
6	1591	12	Placebo	0	47	7/9/2016	111.3	HEIGHT	18.2	15		0	1
6	1591	24	Placebo	0	56	3/23/2017	116.7	HEIGHT	20.8	16		1	1
6	1591	36	Placebo	0	71	5/21/2018	124.5	HEIGHT	23.05	17		0	1
6	1591	48	Placebo	0	82	5/25/2019	130.5	HEIGHT	26.36363636	17		0	1
6	1591	60	Placebo	0	91	2/17/2020	133.6	HEIGHT	28.4	17		0	1
6	1594	0	Placebo	0	48	3/27/2015	93.3	HEIGHT	13.05	14		0	1
6	1594	48	Placebo	0	97	5/25/2019	121	HEIGHT	21	15		0	1
6	1594	60	Placebo	0	107	2/17/2020	124.1	HEIGHT	21.95	15.6		0	1
6	1596	0	Placebo	1	12	3/27/2015	70.8	LENGTH	8.75	15		1	1
6	1596	12	Placebo	1	21	7/9/2016	83.6	HEIGHT	12.1	15.5		0	1
6	1596	24	Placebo	1	35	3/23/2017	89.9	HEIGHT	13.85	15.5		1	1
6	1596	36	Placebo	1	50	5/21/2018	99.3	HEIGHT	15.25	15.5		0	1
6	1596	48	Placebo	1	61	5/25/2019	105.3	HEIGHT	18.22727273	15.5		0	1
6	1596	60	Placebo	1	70	2/17/2020	109.7	HEIGHT	19.35	16.2		0	1
6	1597	0	Placebo	0	36	3/27/2015	85.4	HEIGHT	10.05	13		0	1
6	1597	12	Placebo	0	42	7/9/2016	95.5	HEIGHT	13	15.5		0	1
6	1597	36	Placebo	0	68	5/21/2018	107.9	HEIGHT	17.15	16		0	1
6	1597	60	Placebo	0	88	2/17/2020	116.9	HEIGHT	20	15.8		0	1
6	1598	0	Placebo	1	36	3/27/2015	89.5	HEIGHT	10.75	13.5		0	1
6	1598	24	Placebo	1	59	3/23/2017	101.7	HEIGHT	13.4	13.5		1	1
6	1598	48	Placebo	1	85	5/25/2019	109.5	HEIGHT	16.54545455	14		0	1
6	1599	0	Placebo	0	24	3/27/2015	81.7	HEIGHT	10.3	14.5		0	1
6	1599	24	Placebo	0	48	5/16/2017	96.1	HEIGHT	14	16		0	1

6	1599	36	Placebo	0	63	5/21/2018	103	HEIGHT	15.95	16		0	1
6	1599	48	Placebo	0	73	5/25/2019	107.7	HEIGHT	17.5	15.5		0	1
6	1599	60	Placebo	0	83	3/7/2020	112.2	HEIGHT	18.75	16		0	1
6	1600	0	Placebo	0	24	3/27/2015	84.2	HEIGHT	9.9	12.5		0	1
6	1600	48	Placebo	0	79	5/25/2019	109.2	HEIGHT	16.31818182	13.5		0	1
6	1600	60	Placebo	0	88	2/17/2020	112.5	HEIGHT	16.8	13.8		0	1
6	1602	12	Placebo	1	14	7/9/2016	68.9	LENGTH	7.35	12.5		1	1
6	1602	24	Placebo	1	18	5/16/2017	77.8	LENGTH	9.1	13.5		1	1
6	1602	36	Placebo	1	33	5/21/2018	87.4	HEIGHT	11.35	14		1	1
6	1602	48	Placebo	1	44	5/25/2019	94.3	HEIGHT	13.36363636	14		1	1
6	1603	24	Placebo	1	23	3/23/2017	84.9	HEIGHT	12	14.5		1	1
6	1603	48	Placebo	1	48	5/25/2019	102.7	HEIGHT	15.22727273	15		1	1
6	1603	60	Placebo	1	58	2/17/2020	107	HEIGHT	16.55	15		1	1
6	1604	24	Placebo	1	10	5/16/2017	68.9	LENGTH	6.5	11.5	54	1	1
6	1604	48	Placebo	1	36	5/25/2019	79.6	HEIGHT	8.818181818	12	54	1	1
6	1605	0	Placebo	0	54	3/27/2015	110.2	HEIGHT	14.15	13		0	1
6	1605	48	Placebo	0	103	5/25/2019	127	HEIGHT	22.68181818	16		0	1
6	1605	60	Placebo	0	113	2/17/2020	129.6	HEIGHT	23.6	16		0	1
6	1606	0	Placebo	1	36	3/27/2015	87.7	HEIGHT	11.95	15		0	1
6	1606	24	Placebo	1	53	5/16/2017	103.9	HEIGHT	15.55	14		0	1
6	1606	36	Placebo	1	68	5/21/2018	111	HEIGHT	17.65	15.5		0	1
6	1608	24	Placebo	0	53	5/16/2017	112.3	HEIGHT	16.6	14		1	1
6	1610	0	Placebo	1	36	3/27/2015	84.8	HEIGHT	10.9	13		0	1
6	1611	0	Placebo	0	54	3/27/2015	110.9	HEIGHT	16.65	14.5		1	1
6	1611	12	Placebo	0	90	7/15/2016	118	HEIGHT	17.8	14.5		0	1
6	1611	48	Placebo	0	127	5/25/2019	130.2	HEIGHT	23.77272727	15		0	1
6	1611	60	Placebo	0	136	2/17/2020	134.1	HEIGHT	25.8	15.6		0	1
6	1612	0	Placebo	0	30	3/27/2015	85.4	HEIGHT	11.5	13.5		0	1
6	1612	12	Placebo	0	39	7/9/2016	93	HEIGHT	14.4	14.5		0	1
6	1612	24	Placebo	0	50	3/23/2017	100.1	HEIGHT	16.5	14		1	1
6	1612	36	Placebo	0	65	5/21/2018	108.5	HEIGHT	19	15		0	1
6	1612	48	Placebo	0	76	5/25/2019	116.5	HEIGHT	21.27272727	15		0	1
6	1612	60	Placebo	0	85	2/17/2020	120.4	HEIGHT	22.1	14.3		0	1
6	1613	0	Placebo	1	36	3/27/2015	90	HEIGHT	12.25	14		0	1
6	1613	12	Placebo	1	42	7/10/2016	99.8	HEIGHT	14.75	15.5		0	1
6	1613	24	Placebo	1	53	3/23/2017	104.8	HEIGHT	15.65	15		1	1
6	1613	48	Placebo	1	79	5/25/2019	118.3	HEIGHT	19.90909091	15.5		0	1
6	1614	0	Placebo	0	12	3/27/2015	79.3	LENGTH	9.1	13.5	18	0	1
6	1614	12	Placebo	0	23	7/9/2016	91.8	HEIGHT	13.8	15.5	18	1	1
6	1616	0	Placebo	0	36	3/27/2015	83.6	HEIGHT	10.15	13		1	1

6	1616	12	Placebo	0	44	7/9/2016	92.2	HEIGHT	12.25	13.5	1	1
6	1616	24	Placebo	0	55	5/16/2017	97.1	HEIGHT	13.7	14.5	0	1
6	1616	36	Placebo	0	46	5/21/2018	102.7	HEIGHT	15.25	14.5	0	1
6	1616	48	Placebo	0	57	5/25/2019	107.7	HEIGHT	17.45454545	15	1	1
6	1616	60	Placebo	0	67	2/17/2020	111.4	HEIGHT	18.7	15.6	0	1
6	1617	24	Placebo	1	12	4/2/2017	76.3	HEIGHT	8.75	13.5	1	1
6	1617	36	Placebo	1	24	5/21/2018	85.5	HEIGHT	11.4	15.5	0	1
6	1617	48	Placebo	1	34	5/25/2019	92.3	HEIGHT	13.45454545	15	1	1
6	1617	60	Placebo	1	44	2/17/2020	96.3	HEIGHT	15.05	16.1	0	1
6	1623	12	Placebo	0	12	7/10/2016	77.8	LENGTH	9.95	14.5	1	1
6	1623	24	Placebo	0	23	3/26/2017	83.6	HEIGHT	11.55	15	0	1
6	1624	0	Placebo	0	24	3/27/2015	96.3	HEIGHT	13.7	14	0	1
6	1624	24	Placebo	0	44	3/26/2017	111.2	HEIGHT	16	14.5	1	1
6	1624	48	Placebo	0	70	5/25/2019	119.8	HEIGHT	19.77272727	15.5	0	1
6	1625	0	Placebo	1	36	3/27/2015	90.5	HEIGHT	13.25	15.5	1	1
6	1625	48	Placebo	1	92	5/25/2019	117.5	HEIGHT	19.27272727	15.5	0	1
6	1627	12	Placebo	0	9	7/15/2016	76.4	LENGTH	9.3	14	0	1
6	1627	24	Placebo	0	14	5/16/2017	86.7	HEIGHT	11.7	15	0	1
6	1629	24	Placebo	1	13	3/26/2017	76.7	HEIGHT	8.6	13.5	1	1
6	1631	0	Placebo	0	24	3/27/2015	78.5	LENGTH	9.3	13.5	0	1
6	1631	12	Placebo	0	17	7/9/2016	88.6	HEIGHT	12	14.5	1	1
6	1631	48	Placebo	0	52	5/25/2019	109.2	HEIGHT	16.77272727	14	0	1
6	1632	0	Placebo	1	36	3/27/2015	88.3	HEIGHT	12.9	15.5	0	1
6	1632	24	Placebo	1	56	3/26/2017	100.7	HEIGHT	15.7	16	0	1
6	1632	48	Placebo	1	82	5/25/2019	111.2	HEIGHT	18	16	0	1
6	1632	60	Placebo	1	91	2/17/2020	114.7	HEIGHT	19.8	17	0	1
6	1636	0	Placebo	0	36	3/27/2015	90	HEIGHT	11.3	13	1	1
6	1636	48	Placebo	0	85	5/25/2019	113.4	HEIGHT	17.72727273	13.5	0	1
6	1636	60	Placebo	0	95	2/17/2020	116.5	HEIGHT	18.2	13.4	0	1
6	1638	0	Placebo	0	24	3/27/2015	85.7	HEIGHT	11.25	14	1	1
6	1638	24	Placebo	0	44	3/26/2017	101.1	HEIGHT	15.5	15.5	0	1
6	1639	0	Placebo	1	54	3/27/2015	105.8	HEIGHT	14.95	14.5	1	1
6	1639	24	Placebo	1	11	3/23/2017	114.8	HEIGHT	17.9	15	0	1
6	1639	60	Placebo	1	102	2/17/2020	125	HEIGHT	23.95	16.2	0	1
6	1641	24	Placebo	0	16	3/23/2017	78.5	HEIGHT	9.7	13	1	1
6	1641	36	Placebo	0	31	5/21/2018	90.3	HEIGHT	12.6	14.5	1	1
6	1641	60	Placebo	0	51	2/17/2020	103.2	HEIGHT	14.95	14.1	1	1
6	1645	0	Placebo	0	48	3/27/2015	102	HEIGHT	16.2	15.5	1	1
6	1645	12	Placebo	0	57	7/10/2016	108.8	HEIGHT	18.05	15.5	0	1
6	1645	24	Placebo	0	68	5/16/2017	114.8	HEIGHT	20.15	16.5	0	1

6	1645	36	Placebo	0	83	5/21/2018	119	HEIGHT	20.7	16		0	1
6	1645	48	Placebo	0	94	5/25/2019	121.5	HEIGHT	22.13636364	16		0	1
6	1646	0	Placebo	0	11	3/27/2015	71.4	LENGTH	7.6	13.5		0	1
6	1646	48	Placebo	0	59	5/25/2019	98.7	HEIGHT	14.27272727	15		0	1
6	1647	0	Placebo	1	18	3/27/2015	86.3	LENGTH	12.9	17.5		0	1
6	1647	12	Placebo	0	27	7/9/2016	93.9	HEIGHT	15.05	17.5		0	1
6	1647	24	Placebo	0	38	3/23/2017	98.8	HEIGHT	15.9	16		0	1
6	1647	36	Placebo	0	53	5/21/2018	106.6	HEIGHT	17.9	16.5		1	1
6	1647	60	Placebo	0	73	2/17/2020	117.4	HEIGHT	21.2	17		0	1
6	1648	0	Placebo	1	54	3/27/2015	104.4	HEIGHT	14.65	15		0	1
6	1649	0	Placebo	0	36	3/27/2015	94.4	HEIGHT	12.3	13.5		1	1
6	1649	12	Placebo	0	45	7/10/2016	102	HEIGHT	13.9	13.5		1	1
6	1649	24	Placebo	0	56	3/23/2017	105	HEIGHT	14.9	13.5		0	1
6	1649	36	Placebo	0	71	5/21/2018	110.4	HEIGHT	16.65	14		0	1
6	1649	48	Placebo	0	82	5/25/2019	115.1	HEIGHT	17.68181818	13.5		0	1
6	1649	60	Placebo	0	91	2/17/2020	118.9	HEIGHT	19.15	14.5		0	1
6	1651	24	Placebo	1	32	3/23/2017	85.4	HEIGHT	13.1	15		1	1
6	1651	36	Placebo	1	47	5/21/2018	94.5	HEIGHT	15.4	16.5		1	1
6	1652	0	Placebo	1	36	3/27/2015	86.2	HEIGHT	11.9	15.5		0	1
6	1652	12	Placebo	1	44	7/9/2016	93.3	HEIGHT	14.1	16		0	1
6	1653	0	Placebo	0	36	3/27/2015	92	HEIGHT	13.05	15		0	1
6	1653	12	Placebo	0	45	7/9/2016	100	HEIGHT	14.65	15		0	1
6	1653	36	Placebo	0	74	5/21/2018	112.4	HEIGHT	18	15.5		0	1
6	1653	48	Placebo	0	85	5/25/2019	115.5	HEIGHT	20.09090909	15		0	1
6	1653	60	Placebo	0	94	2/17/2020	119.3	HEIGHT	21.25	16.1		0	1
6	1656	12	Placebo	0	18	7/15/2016	76.5	LENGTH	9	14.5	30	0	1
6	1659	0	Placebo	0	12	3/27/2015	71.1	LENGTH	6.75	12		1	1
6	1659	24	Placebo	0	36	5/16/2017	85.8	HEIGHT	9.75	12		1	1
6	1659	48	Placebo	0	61	5/25/2019	98	HEIGHT	13.77272727	12.5		0	1
6	1659	60	Placebo	0	61	2/17/2020	103.8	HEIGHT	13.85	12.3		0	1
6	1661	12	Placebo	1	30	7/15/2016	89.2	HEIGHT	12.7	14.5	30	1	1
6	1661	24	Placebo	1	41	5/16/2017	94	HEIGHT	13.65	15	30	1	1
6	1662	0	Placebo	0	24	3/27/2015	92.9	HEIGHT	13.55	14		0	1
6	1662	12	Placebo	0	42	7/10/2016	102.3	HEIGHT	15.25	14.5		0	1
6	1662	24	Placebo	0	53	3/23/2017	105.9	HEIGHT	16.15	14		1	1
6	1664	0	Placebo	0	8	3/27/2015	65.6	LENGTH	6.15	12		0	1
6	1664	12	Placebo	0	21	7/9/2016	83	HEIGHT	10.55	14		0	1
6	1664	36	Placebo	0	47	5/21/2018	98.9	HEIGHT	14.7	14		1	1
6	1664	48	Placebo	0	58	5/25/2019	105.1	HEIGHT	16.09090909	15		1	1
6	1664	60	Placebo	0	67	2/17/2020	108.4	HEIGHT	16.35	14		0	1

6	1666	24	Placebo	1	8	3/23/2017	67.5	LENGTH	7.1	12.5		1	1
6	1666	36	Placebo	1	21	5/21/2018	80.5	LENGTH	10.6	15.5		0	1
6	1667	0	Placebo	0	54	3/27/2015	94.3	HEIGHT	12.55	14		0	1
6	1667	12	Placebo	0	67	7/15/2016	102.3	HEIGHT	15.25	14.5		0	1
6	1667	24	Placebo	0	78	3/23/2017	107.4	HEIGHT	16.25	14.5		0	1
6	1667	36	Placebo	0	93	5/21/2018	112.9	HEIGHT	19.1	15.5		0	1
6	1667	48	Placebo	0	103	5/25/2019	117.7	HEIGHT	20.54545455	15		0	1
6	1667	60	Placebo	0	113	2/17/2020	121.4	HEIGHT	21.9	16		0	1
6	1668	0	Placebo	0	48	3/27/2015	105.4	HEIGHT	15.8	14		0	1
6	1668	12	Placebo	0	61	7/10/2016	110.4	HEIGHT	17.5	15		0	1
6	1668	24	Placebo	0	72	5/16/2017	113.8	HEIGHT	18.5	14.5		0	1
6	1668	36	Placebo	0	87	5/21/2018	116.7	HEIGHT	20	15		0	1
6	1668	48	Placebo	0	97	5/25/2019	120.6	HEIGHT	21.77272727	15.5		0	1
6	1672	0	Placebo	1	24	3/27/2015	88.4	HEIGHT	12.6	15		0	1
6	1672	12	Placebo	1	42	7/15/2016	100.2	HEIGHT	14.75	14.5		0	1
6	1672	24	Placebo	1	53	3/23/2017	105.5	HEIGHT	16.5	15.5		0	1
6	1672	60	Placebo	1	96	2/17/2020	122.3	HEIGHT	22.15	16		0	1
6	1673	0	Placebo	0	12	3/27/2015	69.1	LENGTH	7.15	13.5	6	0	1
6	1675	0	Placebo	1	48	3/27/2015	104.5	HEIGHT	15.9	15.5		0	1
6	1675	12	Placebo	1	60	7/15/2016	113.7	HEIGHT	17.3	15		1	1
6	1675	24	Placebo	1	71	3/26/2017	116.3	HEIGHT	19.35	16		0	1
6	1675	36	Placebo	1	86	5/21/2018	122.2	HEIGHT	21.05	16.5		0	1
6	1675	48	Placebo	1	97	5/25/2019	126	HEIGHT	23.45454545	13.5		0	1
6	1678	0	Placebo	0	48	3/27/2015	98	HEIGHT	14.05	14		1	1
6	1678	12	Placebo	1	66	7/9/2016	105.2	HEIGHT	14.5	13.5		0	1
6	1678	24	Placebo	1	77	3/23/2017	109.2	HEIGHT	16.15	13.5		0	1
6	1678	48	Placebo	1	103	5/25/2019	119.6	HEIGHT	18.59090909	14		0	1
6	1679	12	Placebo	1	54	7/15/2016	98.1	HEIGHT	12.5	14.5		1	1
6	1682	0	Placebo	0	12	3/27/2015	71.2	LENGTH	7.65	13		0	1
6	1682	12	Placebo	0	18	7/9/2016	82.5	HEIGHT	9.9	13.5		1	1
6	1682	24	Placebo	0	29	3/23/2017	88.9	HEIGHT	11.7	12.5		0	1
6	1682	36	Placebo	0	44	5/21/2018	99.2	HEIGHT	14.45	14		0	1
6	1682	48	Placebo	0	55	5/25/2019	106.1	HEIGHT	16	13.5		0	1
6	1682	60	Placebo	0	64	2/17/2020	111.1	HEIGHT	16.95	13.8		0	1
6	1683	24	Placebo	0	12	3/23/2017	71.9	LENGTH	9.3	15.5		1	1
6	1683	36	Placebo	0	26	5/21/2018	80.7	HEIGHT	10.4	15		0	1
6	1683	48	Placebo	0	37	5/25/2019	86.7	HEIGHT	12.31818182	15.5		0	1
6	1684	0	Placebo	1	18	3/27/2015	96.9	HEIGHT	14.5	15.5	24	1	1
6	1688	0	Placebo	1	54	3/27/2015	110.8	HEIGHT	16.85	15		0	1
6	1688	48	Placebo	1	121	5/25/2019	130.4	HEIGHT	26.36363636	17.5		0	1

6	1690	0	Placebo	1	48	3/27/2015	85.9	HEIGHT	11.35	14.5		0	1
6	1690	24	Placebo	1	53	3/23/2017	101.2	HEIGHT	15.1	15.5		1	1
6	1690	48	Placebo	1	79	5/25/2019	112.6	HEIGHT	18.09090909	15		0	1
6	1690	60	Placebo	1	88	2/17/2020	116.5	HEIGHT	19.65	15.6		0	1
6	1691	12	Placebo	1	42	7/15/2016	115.6	HEIGHT	18	15		1	1
6	1693	12	Placebo	1	49	7/10/2016	106.1	HEIGHT	16.95	15		1	1
6	1694	0	Placebo	1	36	3/27/2015	95.3	HEIGHT	15.45	16.5		0	1
6	1694	12	Placebo	1	45	7/9/2016	102.2	HEIGHT	16.85	17		0	1
6	1694	24	Placebo	1	56	5/16/2017	108.6	HEIGHT	18.3	17		0	1
6	1695	0	Placebo	1	48	3/27/2015	103.9	HEIGHT	14.5	13		0	1
6	1695	12	Placebo	1	78	7/10/2016	111.6	HEIGHT	15.9	13		0	1
6	1695	24	Placebo	1	89	3/23/2017	114.9	HEIGHT	17.15	13.5		0	1
6	1698	12	Placebo	0	7	7/10/2016	69.1	HEIGHT	7.15	12		0	1
6	1698	24	Placebo	0	14	5/16/2017	80	HEIGHT	9.2	13		0	1
6	1698	36	Placebo	0	29	5/21/2018	88.6	HEIGHT	11.1	13.5		1	1
6	1698	60	Placebo	0	53	3/7/2020	102.2	HEIGHT	14.35	13.5		0	1
6	1699	24	Placebo	0	42	5/16/2017	104.7	HEIGHT	17.15	17		1	1
6	1699	36	Placebo	0	57	5/21/2018	110.4	HEIGHT	18	16		1	1
6	1700	0	Placebo	0	48	3/27/2015	95	HEIGHT	13.55	13.5		1	1
6	1700	48	Placebo	0	94	5/25/2019	120.6	HEIGHT	20.27272727	15		0	1
6	1700	60	Placebo	0	103	2/17/2020	124.6	HEIGHT	22.55	15.7		0	1
6	1701	12	Placebo	0	11	7/10/2016	70.8	LENGTH	7.2	12.5		0	1
6	1701	24	Placebo	0	20	3/23/2017	78.1	HEIGHT	8.7	13.5		1	1
6	1702	12	Placebo	1	3	7/9/2016	57.3	LENGTH	5	12.5		0	1
6	1702	24	Placebo	1	14	3/23/2017	65.2	LENGTH	7.25	14.5		0	1
6	1702	36	Placebo	1	28	5/21/2018	75.7	HEIGHT	9.7	15.5		1	1
6	1702	60	Placebo	1	49	2/17/2020	87.6	LENGTH	13.65	16.4		1	1
6	1703	12	Placebo	0	12	7/15/2016	76.9	LENGTH	9.2	14		1	1
6	1703	36	Placebo	0	40	5/21/2018	92.3	HEIGHT	12.85	15		0	1
6	1703	48	Placebo	0	45	5/25/2019	98.1	HEIGHT	14.22727273	14.5		1	1
6	1705	0	Placebo	1	5	3/27/2015	61.8	LENGTH	5.75	13	6	0	1
6	1706	0	Placebo	1	36	3/27/2015	103	HEIGHT	15.15	14.5		0	1
6	1707	0	Placebo	1	36	3/27/2015	98.2	HEIGHT	14	13.5		0	1
6	1708	12	Placebo	1	66	7/15/2016	121.6	HEIGHT	21.55	16.5		0	1
6	1710	24	Placebo	0	32	3/23/2017	93.6	HEIGHT	13.2	14		1	1
6	1710	36	Placebo	0	46	5/21/2018	102.3	HEIGHT	16.75	16		1	1
6	1712	0	Placebo	0	10	3/27/2015	77.3	LENGTH	9.1	12		0	1
6	1715	0	Placebo	1	48	3/27/2015	112.4	HEIGHT	16.05	15		1	1
6	1715	12	Placebo	1	59	7/9/2016	117.3	HEIGHT	18.85	16		1	1
6	1715	24	Placebo	1	68	3/23/2017	121	HEIGHT	19.15	15.5		0	1

6	1715	36	Placebo	1	83	5/21/2018	126.2	HEIGHT	21.3	16.5		0	1
6	1715	48	Placebo	1	94	5/25/2019	130	HEIGHT	23.40909091	16.5		0	1
6	1715	60	Placebo	1	103	2/17/2020	133.6	HEIGHT	23.92	16.4		0	1
6	1716	0	Placebo	1	5	3/27/2015	66.5	LENGTH	6.85	13		0	1
6	1716	12	Placebo	1	18	7/15/2016	81.9	LENGTH	9.7	15		1	1
6	1716	24	Placebo	1	29	3/26/2017	88.6	HEIGHT	12.15	15.5		1	1
6	1716	36	Placebo	1	44	5/21/2018	98.3	HEIGHT	13.95	16		1	1
6	1716	48	Placebo	1	55	5/25/2019	104.8	HEIGHT	15.40909091	15		0	1
6	1718	12	Placebo	0	42	7/10/2016	93.3	HEIGHT	13.6	15		1	1
6	1719	0	Placebo	0	7	3/27/2015	65.3	LENGTH	6	11.5		0	1
6	1719	12	Placebo	0	30	7/10/2016	80.4	HEIGHT	9.75	14		1	1
6	1719	24	Placebo	0	41	3/23/2017	86.9	HEIGHT	12.4	15.5		0	1
6	1719	36	Placebo	0	56	5/21/2018	97.3	HEIGHT	14.8	15.5		1	1
6	1719	48	Placebo	0	56	5/25/2019	106.1	HEIGHT	16.68181818	15		1	1
6	1722	0	Placebo	0	9	3/27/2015	64.8	LENGTH	6.45	13	18	1	1
6	1722	12	Placebo	0	18	7/9/2016	74.3	LENGTH	9.6	15	18	1	1
6	1723	24	Placebo	1	5	3/26/2017	64.5	LENGTH	6.9	14		1	1
6	1723	48	Placebo	1	31	5/25/2019	87.8	HEIGHT	12.68181818	14.5		1	1
6	1726	0	Placebo	0	36	3/27/2015	105.8	HEIGHT	17.4	16		0	1
6	1726	12	Placebo	0	57	7/9/2016	112.9	HEIGHT	18.9	15		0	1
6	1726	24	Placebo	0	68	3/26/2017	117.2	HEIGHT	20.45	16.5		0	1
6	1726	36	Placebo	0	83	5/21/2018	122.6	HEIGHT	23.5	17.5		0	1
6	1726	48	Placebo	0	94	5/25/2019	127.6	HEIGHT	25.72727273	17		0	1
6	1726	60	Placebo	0	103	2/17/2020	129.7	HEIGHT	27.8	18		0	1
6	1727	0	Placebo	0	36	3/27/2015	89	HEIGHT	11.3	14.5		1	1
6	1727	12	Placebo	0	45	7/10/2016	95.8	HEIGHT	13.75	14.5		1	1
6	1727	24	Placebo	0	56	5/16/2017	104.5	HEIGHT	16.15	15		0	1
6	1727	36	Placebo	0	71	5/21/2018	110.2	HEIGHT	17.3	15		0	1
6	1727	48	Placebo	0	82	5/25/2019	115	HEIGHT	18.27272727	15		0	1
6	8004	36	Placebo	1	37	5/21/2018	89.2	HEIGHT	11.6	13.5		1	1
6	8004	60	Placebo	1	58	2/17/2020	102.3	HEIGHT	14.4	13.5		1	1
6	8008	60	Placebo	1	21	2/17/2020	84.1	HEIGHT	11.4	14.7		1	1
6	8010	36	Placebo	1	15	6/12/2018	72.2	LENGTH	7.590909091	13	48	1	1
6	8022	36	Placebo	1	32	6/12/2018	90.6	HEIGHT	12.27272727	14		1	1
6	8022	48	Placebo	1	42	5/25/2019	99.3	HEIGHT	14.86363636	14.5		1	1
6	8027	60	Placebo	0	38	3/7/2020	86.9	HEIGHT	11.95	13.5		1	1
6	8036	48	Placebo	1	8	5/25/2019	65.5	HEIGHT	6.772727273	13		1	1
6	8040	48	Placebo	1	2	5/25/2019	57.6	LENGTH	5.227272727	12		1	1
6	8040	60	Placebo	1	12	2/17/2020	75.7	LENGTH	7.15	12.1		0	1
6	8060	60	Placebo	0	24	3/7/2020	77.4	LENGTH	9.15	12.5		1	1

6	8080	60	Placebo	1	7	3/7/2020	68.9	LENGTH	8.3	14.2		1	1
6	8089	36	Placebo	0	8	6/12/2018	71.8	LENGTH	8.681818182	14		1	1
6	8092	60	Placebo	1	12	3/7/2020	81	HEIGHT	10	12.7		1	1
6	8164	36	Placebo	0	11	6/12/2018	72.8	LENGTH	8.5	14		1	1
6	8164	48	Placebo	0	22	5/25/2019	81.8	LENGTH	10.22727273	14		0	1
6	8198	60	Placebo	1	29	2/17/2020	83.1	HEIGHT	9.9	12.2		1	1
6	8202	48	Placebo	0	5	6/6/2019	64.8	LENGTH	6.909090909	13.5		1	1
6	8202	60	Placebo	0	15	2/17/2020	74.5	HEIGHT	8.5	13.2		1	1
6	8252	60	Placebo	1	1	2/17/2020	60.2	HEIGHT	5.65	12.5		1	1
6	8278	48	Placebo	0	2	5/25/2019	61.1	LENGTH	6.681818182	13.5		1	1
6	8296	36	Placebo	0	93	5/21/2018	144.8	HEIGHT	31.35	18.5		0	1
6	8296	60	Placebo	0	113	2/17/2020	156.8	HEIGHT	39.75	20		0	1
6	8335	36	Placebo	0	13	5/21/2018	76.9	LENGTH	10.3	14		1	1
6	8335	48	Placebo	0	24	5/25/2019	86.1	HEIGHT	12.95454545	14.5		1	1
6	8335	60	Placebo	0	34	2/17/2020	93.2	HEIGHT	14.8	15.4		0	1
6	8352	36	Placebo	1	57	5/21/2018	111.1	HEIGHT	17.3	16		1	1
6	8390	60	Placebo	0	14	3/7/2020	78.5	LENGTH	9.65	13.5		1	1
6	8407	36	Placebo	1	21	5/21/2018	83.8	LENGTH	10.3	14		1	1
6	8407	48	Placebo	1	34	5/25/2019	91.8	HEIGHT	12.86363636	15		1	1
6	8407	60	Placebo	1	43	2/17/2020	98.1	HEIGHT	15.35	16		1	1
6	8422	48	Placebo	0	19	5/25/2019	75.8	LENGTH	8.636363636	14		1	1
6	8422	60	Placebo	0	28	2/17/2020	83.9	LENGTH	10.6	15		1	1
6	8451	48	Placebo	1	3	6/6/2019	64.6	LENGTH	8.045454545	15.4		1	1
6	8451	60	Placebo	1	13	2/17/2020	71.5	HEIGHT	9.7	15		0	1
6	8466	36	Placebo	0	29	6/12/2018	85	HEIGHT	9.954545455	12.5		1	1
6	8466	60	Placebo	0	50	3/7/2020	96	HEIGHT	13.4	12.9		1	1
6	8486	60	Placebo	1	6	2/17/2020	67.9	LENGTH	7.55	13.5		1	1
6	8532	60	Placebo	1	21	2/17/2020	78.2	HEIGHT	9.2	12.3		1	1
6	8537	36	Placebo	0	12	5/21/2018	72.8	LENGTH	9.45	15		1	1
6	8537	48	Placebo	0	23	5/25/2019	85.9	HEIGHT	12.5	14.5		1	1
6	8537	60	Placebo	0	32	2/17/2020	93.4	HEIGHT	15.2	17		0	1
6	8555	36	Placebo	0	23	6/12/2018	81.1	HEIGHT	9.5	13		1	1
6	8555	48	Placebo	0	34	5/25/2019	91.3	HEIGHT	11.86363636	13.5		1	1
6	8555	60	Placebo	0	44	2/17/2020	97.4	HEIGHT	13.55	14.4		1	1
6	8575	60	Placebo	1	22	3/7/2020	78.5	LENGTH	8.75	12		1	1
6	8725	36	Placebo	0	21	5/21/2018	84.6	HEIGHT	12.25	15		1	1
6	8725	48	Placebo	0	31	6/6/2019	92.1	HEIGHT	15.13636364	15.5		1	1
6	8725	60	Placebo	0	41	3/7/2020	97.4	HEIGHT	16.7	15		1	1
6	8736	60	Placebo	0	8	2/17/2020	72.5	LENGTH	9.95	15		1	1
6	8740	48	Placebo	0	18	5/25/2019	75	LENGTH	8.545454545	13		1	1

6	8744	60	Placebo	1	21	3/7/2020	82.4	HEIGHT	10.25	13.5	1	1
6	8799	36	Placebo	1	26	6/12/2018	80	HEIGHT	9.363636364	12.5	1	1
6	8799	60	Placebo	1	47	2/17/2020	94.5	HEIGHT	13.85	14.7	1	1
6	8833	60	Placebo	0	10	3/7/2020	68.8	LENGTH	8.2	14	1	1
6	8873	36	Placebo	0	24	5/21/2018	80.3	HEIGHT	9.7	13	1	1
6	8873	60	Placebo	0	45	2/17/2020	95	HEIGHT	14.4	15.5	1	1
6	8922	60	Placebo	1	19	3/7/2020	82.9	LENGTH	10.3	13.2	1	1
6	8942	36	Placebo	0	9	5/21/2018	66.8	LENGTH	7.6	13.5	1	1
6	8942	48	Placebo	0	20	5/25/2019	80.1	HEIGHT	10.5	14	1	1
6	8942	60	Placebo	0	29	2/17/2020	86.4	LENGTH	11.7	14	0	1
6	8943	60	Placebo	0	10	3/7/2020	71.5	LENGTH	7.65	13	1	1
6	8945	36	Placebo	1	22	6/12/2018	81.3	HEIGHT	10.54545455	15	1	1
6	8974	36	Placebo	0	9	6/12/2018	67.4	LENGTH	7.136363636	12	1	1
6	8974	48	Placebo	0	19	5/25/2019	79.5	LENGTH	9.727272727	14	0	1
6	8974	60	Placebo	0	29	2/17/2020	87.7	HEIGHT	11.95	14.5	0	1
6	8980	36	Placebo	1	29	5/21/2018	86.3	HEIGHT	10.95	14	1	1
6	9025	48	Placebo	0	47	5/25/2019	98.2	HEIGHT	15.09090909	15	1	1
6	9081	36	Placebo	0	17	5/21/2018	78.5	LENGTH	10	14.5	1	1
6	9098	36	Placebo	0	11	5/21/2018	76.8	LENGTH	8.85	14	1	1
6	9098	48	Placebo	0	22	5/25/2019	85.4	HEIGHT	11.54545455	15.5	1	1
6	9098	60	Placebo	0	32	2/17/2020	93.5	HEIGHT	14.6	16.5	0	1
6	9119	36	Placebo	1	34	5/21/2018	96.3	HEIGHT	14.45	16	1	1
6	9136	60	Placebo	1	25	2/17/2020	77.3	HEIGHT	9.6	13.5	1	1
6	9166	60	Placebo	0	41	2/17/2020	80.8	HEIGHT	10.8	13.5	1	1
6	9184	48	Placebo	1	48	5/25/2019	96.3	HEIGHT	12.40909091	13	1	1
6	9184	60	Placebo	1	58	3/7/2020	102.6	HEIGHT	13.55	13	1	1
6	9194	36	Placebo	0	33	6/12/2018	83.8	HEIGHT	9.636363636	12	1	1
6	9194	60	Placebo	0	53	3/7/2020	94.9	HEIGHT	12.35	12	1	1
6	9240	36	Placebo	0	51	5/21/2018	94.8	HEIGHT	13.55	14	1	1
6	9271	36	Placebo	1	56	6/12/2018	103.7	HEIGHT	15	14	1	1
6	9307	60	Placebo	0	3	2/17/2020	64.1	HEIGHT	7.1	13.5	1	1
6	9342	36	Placebo	0	12	6/12/2018	69.4	HEIGHT	6.909090909	12	1	1
6	9343	48	Placebo	0	44	6/6/2019	97.6	HEIGHT	14.18181818	15	1	1
6	9415	60	Placebo	1	25	2/17/2020	89.8	HEIGHT	14.15	16.4	1	1
6	9464	48	Placebo	0	29	5/25/2019	81.1	HEIGHT	8.727272727	12	1	1
6	9480	36	Placebo	0	20	6/12/2018	78.6	HEIGHT	9	12.5	1	1
6	9513	60	Placebo	1	12	2/17/2020	68.7	LENGTH	7.15	12	1	1
6	9514	48	Placebo	0	10	5/25/2019	72.8	LENGTH	8.318181818	13.5	1	1
6	9517	48	Placebo	1	22	5/25/2019	80.5	LENGTH	8.954545455	13	1	1
6	9518	48	Placebo	1	26	5/25/2019	74.9	HEIGHT	7.863636364	12.5	1	1

6	9537	60	Placebo	1	43	2/17/2020	96.7	HEIGHT	14.8	15.8			1	1
6	9540	48	Placebo	0	5	5/25/2019	67.5	LENGTH	7.181818182	13			1	1
6	9540	60	Placebo	0	15	2/17/2020	74.3	LENGTH	8.45	12.8			0	1
7	1742	0	Placebo	1	42	3/21/2015	102.4	HEIGHT	15	14.5			1	0
7	1742	12	Placebo	1	66	6/12/2016	107.5	HEIGHT	16.7	14.5			0	0
7	1742	24	Placebo	1	77	3/21/2017	112.8	HEIGHT	17.65	14.5			0	0
7	1742	36	Placebo	1	92	6/11/2018	119.1	HEIGHT	19.2	14.5			0	0
7	1742	48	Placebo	1	103	4/17/2019	122.6	HEIGHT	20.6	15.5			0	0
7	1742	60	Placebo	1	112	2/9/2020	126.5	HEIGHT	23.3	16.5			0	0
7	1745	0	Placebo	1	36	3/21/2015	90.5	HEIGHT	12.6	14.5			1	0
7	1745	12	Placebo	1	42	6/12/2016	98.7	HEIGHT	13.95	15.5			1	0
7	1745	24	Placebo	1	53	3/21/2017	105.5	HEIGHT	14.8	14.5			1	0
7	1745	36	Placebo	1	66	6/11/2018	113.4	HEIGHT	16.9	14.5			0	0
7	1745	48	Placebo	1	77	4/17/2019	118.8	HEIGHT	18.8	15			0	0
7	1745	60	Placebo	1	86	2/9/2020	123.3	HEIGHT	21.65	17			0	0
7	1750	0	Placebo	0	54	3/21/2015	104.8	HEIGHT	15.15	15.5			1	0
7	1750	12	Placebo	0	65	6/12/2016	111.6	HEIGHT	16.9	15.5			0	0
7	1750	24	Placebo	0	74	3/21/2017	117.8	HEIGHT	17.7	15			0	0
7	1750	36	Placebo	0	89	6/11/2018	124.2	HEIGHT	20.75	15.5			0	0
7	1750	48	Placebo	0	100	4/17/2019	128.5	HEIGHT	22.9	16			0	0
7	1751	24	Placebo	1	6	3/21/2017	66.3	LENGTH	6.75	13			1	0
7	1751	36	Placebo	1	21	6/11/2018	77.3	LENGTH	8.9	12.5			1	0
7	1751	48	Placebo	1	32	4/17/2019	83.4	HEIGHT	10.2	13.5			1	0
7	1751	60	Placebo	1	41	2/9/2020	92.4	HEIGHT	12.2	14			1	0
7	1752	0	Placebo	0	36	3/21/2015	84	HEIGHT	10.7	13			1	0
7	1752	12	Placebo	0	45	6/12/2016	93.2	HEIGHT	12.5	14.5			1	0
7	1752	24	Placebo	0	56	3/21/2017	97.6	HEIGHT	13.1	13.5			1	0
7	1752	36	Placebo	0	71	6/11/2018	104.7	HEIGHT	14.9	14			0	0
7	1752	48	Placebo	0	82	4/17/2019	109	HEIGHT	16.95	14.5			0	0
7	1752	60	Placebo	0	91	2/9/2020	113.5	HEIGHT	18.4	15.2			0	0
7	1753	0	Placebo	0	48	3/21/2015	88.7	HEIGHT	10	13.5			1	0
7	1753	12	Placebo	0	44	6/12/2016	99.8	HEIGHT	12.85	14.5			1	0
7	1753	24	Placebo	0	53	3/21/2017	102.6	HEIGHT	13.1	13.5			1	0
7	1753	36	Placebo	0	68	6/11/2018	109.2	HEIGHT	14.5	13.5			0	0
7	1753	48	Placebo	0	79	4/17/2019	112.2	HEIGHT	15.85	14			0	0
7	1753	60	Placebo	0	88	2/9/2020	116.3	HEIGHT	16.75	14			0	0
7	1754	12	Placebo	1	69	6/27/2016	114.5	HEIGHT	17.45	14.5			0	0
7	1754	36	Placebo	1	95	6/11/2018	123.6	HEIGHT	21.1	16			0	0
7	1754	60	Placebo	1	115	2/9/2020	131.6	HEIGHT	25.4	18			0	0
7	1755	12	Placebo	0	54	6/12/2016	112.2	HEIGHT	17	15.5			1	0

7	1755	36	Placebo	0	80	6/11/2018	122.4	HEIGHT	20.2	15.5		0	0
7	1755	60	Placebo	0	100	2/9/2020	130.5	HEIGHT	23.8	16		0	0
7	1756	24	Placebo	0	14	3/21/2017	70.1	HEIGHT	7.05	12	30	1	0
7	1759	0	Placebo	1	1	3/21/2015	94.2	HEIGHT	14.5	16		1	0
7	1759	12	Placebo	0	12	6/12/2016	103.9	HEIGHT	17.65	17		1	0
7	1759	24	Placebo	0	69	3/21/2017	110.2	HEIGHT	19.2	16.5		0	0
7	1759	36	Placebo	0	120	6/11/2018	116.8	HEIGHT	21.5	17		0	0
7	1759	48	Placebo	0	130	4/17/2019	121.4	HEIGHT	24.85	18.5		0	0
7	1759	60	Placebo	0	140	2/9/2020	125.2	HEIGHT	26.45	19		0	0
7	1763	0	Placebo	1	28	3/21/2015	82.9	HEIGHT	11.2	14.5		1	0
7	1764	24	Placebo	1	3	3/21/2017	60	LENGTH	5.35	12		1	0
7	1764	36	Placebo	1	15	6/11/2018	74.4	LENGTH	8.05	13		1	0
7	1764	48	Placebo	1	26	4/17/2019	81.4	HEIGHT	10.3	14.5		1	0
7	1764	60	Placebo	1	35	2/9/2020	89.4	HEIGHT	12.35	14.5		1	0
7	1766	0	Placebo	0	24	3/21/2015	82.4	HEIGHT	9.65	14		1	0
7	1766	12	Placebo	0	35	6/12/2016	92.1	HEIGHT	12.8	15		1	0
7	1766	24	Placebo	0	44	3/21/2017	97.3	HEIGHT	13.95	14		1	0
7	1766	36	Placebo	0	59	6/11/2018	103.9	HEIGHT	15.95	14.5		1	0
7	1766	48	Placebo	0	70	4/17/2019	109.8	HEIGHT	17.3	15		0	0
7	1766	60	Placebo	0	79	2/9/2020	116	HEIGHT	18.85	15		0	0
7	1769	24	Placebo	1	2	3/21/2017	59.1	LENGTH	5.9	12.5		1	0
7	1769	36	Placebo	1	17	6/11/2018	74	LENGTH	9.05	13		1	0
7	1769	48	Placebo	1	28	4/17/2019	81.6	HEIGHT	10.95	14		1	0
7	1769	60	Placebo	1	37	2/9/2020	88.6	HEIGHT	12.4	14		1	0
7	1770	24	Placebo	1	8	3/21/2017	71.4	HEIGHT	8.2	14.5		1	0
7	1770	36	Placebo	1	21	6/11/2018	81.1	HEIGHT	10.55	14		1	0
7	1771	0	Placebo	1	7	3/21/2015	69	LENGTH	7.2	13.5		1	0
7	1771	24	Placebo	1	26	3/21/2017	90	HEIGHT	10.85	13		1	0
7	1771	36	Placebo	1	41	6/11/2018	97.7	HEIGHT	13.15	13.5		1	0
7	1771	48	Placebo	1	52	4/17/2019	102.7	HEIGHT	14.6	14.5		1	0
7	1773	0	Placebo	0	18	3/21/2015	72.7	LENGTH	8.3	14		1	0
7	1773	12	Placebo	0	20	6/12/2016	83.2	HEIGHT	11	14.5		1	0
7	1773	24	Placebo	0	29	3/21/2017	90.1	HEIGHT	13.25	14.5		1	0
7	1773	36	Placebo	0	44	6/11/2018	98.5	HEIGHT	15.4	15		1	0
7	1773	48	Placebo	0	55	4/17/2019	104.4	HEIGHT	16.85	15.5		1	0
7	1773	60	Placebo	0	56	2/9/2020	109.6	HEIGHT	19.1	15.5		1	0
7	1774	0	Placebo	0	36	3/21/2015	91.5	HEIGHT	13.3	15.5		1	0
7	1774	24	Placebo	0	56	3/21/2017	109.4	HEIGHT	17.4	15.5		1	0
7	1774	36	Placebo	0	71	6/11/2018	116.8	HEIGHT	19.4	15		0	0
7	1774	48	Placebo	0	82	4/17/2019	122.5	HEIGHT	20.75	15		0	0

7	1774	60	Placebo	0	91	2/9/2020	127.1	HEIGHT	22.75	15.5	0	0
7	1775	0	Placebo	0	36	3/21/2015	81.2	HEIGHT	9.8	14	1	0
7	1775	24	Placebo	0	56	3/21/2017	94.3	HEIGHT	12.9	14	1	0
7	1775	36	Placebo	0	71	6/11/2018	99.9	HEIGHT	13.4	14	0	0
7	1775	48	Placebo	0	82	4/17/2019	105.1	HEIGHT	15.45	14.5	0	0
7	1775	60	Placebo	0	91	2/9/2020	109.3	HEIGHT	16.4	14.5	0	0
7	1787	12	Placebo	1	35	6/12/2016	84.6	HEIGHT	10.4	13.5	1	0
7	1787	24	Placebo	0	44	3/21/2017	89.7	HEIGHT	12	12.5	1	0
7	1787	36	Placebo	0	59	6/11/2018	98.6	HEIGHT	14.6	13.5	1	0
7	1787	60	Placebo	0	79	2/9/2020	113.4	HEIGHT	18.3	14	0	0
7	1788	12	Placebo	1	12	6/12/2016	74.6	HEIGHT	7.95	13	1	0
7	1794	0	Placebo	0	24	3/21/2015	86.2	HEIGHT	11.5	13.5	1	0
7	1794	12	Placebo	0	35	6/12/2016	94.2	HEIGHT	14	15	1	0
7	1794	24	Placebo	0	50	3/21/2017	101.5	HEIGHT	16.25	14.5	1	0
7	1794	36	Placebo	0	65	6/11/2018	109.4	HEIGHT	18	14	0	0
7	1794	48	Placebo	0	76	4/17/2019	114.4	HEIGHT	20.7	15	0	0
7	1794	60	Placebo	0	85	2/9/2020	120.2	HEIGHT	21.95	15.5	0	0
7	1795	0	Placebo	1	24	3/21/2015	86.3	HEIGHT	11.15	15	1	0
7	1795	12	Placebo	1	33	6/12/2016	94.2	HEIGHT	12.8	14.5	1	0
7	1795	24	Placebo	1	50	3/21/2017	99	HEIGHT	13.5	14.5	1	0
7	1795	36	Placebo	1	65	6/11/2018	106.8	HEIGHT	15.35	15	0	0
7	1795	48	Placebo	1	76	4/17/2019	112	HEIGHT	17.2	16	0	0
7	1795	60	Placebo	1	85	2/9/2020	116.1	HEIGHT	18.6	16	0	0
7	1797	0	Placebo	0	18	3/21/2015	82.7	HEIGHT	11.55	15	1	0
7	1797	12	Placebo	0	27	6/12/2016	93.5	HEIGHT	15.35	17	1	0
7	1797	24	Placebo	0	36	3/21/2017	99.8	HEIGHT	16.5	16	1	0
7	1797	36	Placebo	0	51	6/11/2018	109.5	HEIGHT	19.5	17	1	0
7	1797	48	Placebo	0	62	4/17/2019	114.2	HEIGHT	21.9	17	0	0
7	1797	60	Placebo	0	61	2/9/2020	120.2	HEIGHT	23.25	17.5	0	0
7	1798	0	Placebo	1	24	3/21/2015	77.6	HEIGHT	8.8	13.5	1	0
7	1798	12	Placebo	1	38	6/12/2016	87.3	HEIGHT	10.85	14.5	1	0
7	1798	24	Placebo	1	48	3/21/2017	94.9	HEIGHT	12.5	14	1	0
7	1798	36	Placebo	1	62	6/11/2018	103.8	HEIGHT	13.2	13	0	0
7	1798	48	Placebo	1	73	4/17/2019	107.9	HEIGHT	14.5	14	0	0
7	1800	0	Placebo	1	4	3/21/2015	55.5	LENGTH	5.2	13	1	0
7	1800	12	Placebo	1	15	6/12/2016	70.2	LENGTH	7	12	1	0
7	1800	24	Placebo	1	26	3/21/2017	78	HEIGHT	9.45	13	1	0
7	1800	36	Placebo	1	41	6/11/2018	86.6	HEIGHT	11.35	14.5	1	0
7	1800	48	Placebo	1	52	4/17/2019	93.3	HEIGHT	12.5	14.5	1	0
7	1800	60	Placebo	1	61	2/9/2020	97.9	HEIGHT	13.25	14	0	0

7	1802	0	Placebo	0	8	3/21/2015	63.5	LENGTH	6.9	13.5	1	0
7	1802	12	Placebo	0	15	6/12/2016	72.8	HEIGHT	8.7	14	1	0
7	1802	24	Placebo	0	26	3/21/2017	77.6	HEIGHT	10.1	14	1	0
7	1802	36	Placebo	0	41	6/11/2018	88.6	HEIGHT	12.1	14	1	0
7	1802	48	Placebo	0	52	4/17/2019	94.5	HEIGHT	13.9	15	1	0
7	1802	60	Placebo	0	61	2/9/2020	98.6	HEIGHT	14.8	15	0	0
7	1803	0	Placebo	0	12	3/21/2015	77.3	HEIGHT	9.85	15	1	0
7	1804	0	Placebo	1	10	3/21/2015	66.9	LENGTH	7.85	15.5	1	0
7	1804	12	Placebo	1	21	6/12/2016	77.8	HEIGHT	9.6	15.5	1	0
7	1804	24	Placebo	1	32	3/21/2017	83.5	HEIGHT	11.35	15	1	0
7	1804	36	Placebo	1	50	6/11/2018	91.1	HEIGHT	13.1	15	1	0
7	1804	48	Placebo	1	61	4/17/2019	96.4	HEIGHT	13.85	15.5	0	0
7	1804	60	Placebo	1	59	2/9/2020	100.9	HEIGHT	14.7	15.5	1	0
7	1806	0	Placebo	0	18	3/21/2015	78.6	HEIGHT	10.2	15	1	0
7	1806	12	Placebo	0	30	6/12/2016	86.4	HEIGHT	12.95	16.5	1	0
7	1806	24	Placebo	0	41	3/21/2017	91.6	HEIGHT	13.5	15.5	1	0
7	1806	36	Placebo	0	56	6/11/2018	98.8	HEIGHT	14.75	15	1	0
7	1806	48	Placebo	0	67	4/17/2019	103.2	HEIGHT	15.8	15	0	0
7	1806	60	Placebo	0	76	2/9/2020	108.6	HEIGHT	16.75	15.5	0	0
7	1808	12	Placebo	1	7	6/12/2016	65.5	HEIGHT	7.6	14.5	1	0
7	1808	24	Placebo	1	14	3/21/2017	76	HEIGHT	9.3	13.5	1	0
7	1808	36	Placebo	1	29	6/11/2018	84.8	HEIGHT	11.7	14	1	0
7	1808	48	Placebo	1	40	4/17/2019	91.9	HEIGHT	13.55	15.5	1	0
7	1808	60	Placebo	1	49	2/9/2020	98.4	HEIGHT	14.85	15	1	0
7	1809	12	Placebo	1	69	6/27/2016	111.2	HEIGHT	17.75	14	0	0
7	1809	36	Placebo	1	93	6/11/2018	123.2	HEIGHT	20.9	15	0	0
7	1809	60	Placebo	1	113	2/9/2020	132.3	HEIGHT	24.9	16	0	0
7	1811	12	Placebo	1	32	6/12/2016	92.4	HEIGHT	12.9	14.5	1	0
7	1811	24	Placebo	1	32	3/21/2017	97.7	HEIGHT	14.25	15	1	0
7	1811	36	Placebo	1	47	6/11/2018	105.4	HEIGHT	15.2	14	1	0
7	1811	48	Placebo	1	58	4/17/2019	110.9	HEIGHT	16.95	14.5	1	0
7	1811	60	Placebo	1	67	2/9/2020	117.3	HEIGHT	18.45	15	0	0
7	1813	0	Placebo	0	36	3/21/2015	110.5	HEIGHT	16	13	1	0
7	1813	12	Placebo	0	80	6/12/2016	118.6	HEIGHT	17.7	13	0	0
7	1813	48	Placebo	0	117	4/17/2019	132.2	HEIGHT	25.65	16	0	0
7	1815	24	Placebo	1	53	3/21/2017	93.7	HEIGHT	13.4	14.5	1	0
7	1815	36	Placebo	1	68	6/11/2018	99.1	HEIGHT	14	14	0	0
7	1815	60	Placebo	1	88	2/9/2020	109.2	HEIGHT	16.55	14.5	0	0
7	8013	36	Placebo	1	48	6/11/2018	93.2	HEIGHT	12.25	13.5	1	0
7	8013	48	Placebo	1	58	4/17/2019	97.9	HEIGHT	14.4	14.5	1	0

7	8050	48	Placebo	0	43	4/17/2019	98.1	HEIGHT	15.8	15.5		1	0
7	8050	60	Placebo	0	53	2/9/2020	105.6	HEIGHT	16.45	16		1	0
7	8052	60	Placebo	1	26	2/9/2020	82.3	HEIGHT	8.75	12.5		1	0
7	8263	36	Placebo	0	7	6/11/2018	70	LENGTH	7.6	13		1	0
7	8263	48	Placebo	0	18	4/17/2019	81.6	HEIGHT	9.75	13.5		1	0
7	8263	60	Placebo	0	27	3/8/2020	88.4	HEIGHT	11.8	13.2		1	0
7	8301	36	Placebo	1	24	6/11/2018	74.7	LENGTH	8.9	13		1	0
7	8301	48	Placebo	1	35	4/17/2019	81.1	HEIGHT	10.95	14		1	0
7	8301	60	Placebo	1	44	2/9/2020	87.7	HEIGHT	12.45	14		1	0
7	8322	36	Placebo	1	15	6/11/2018	74.8	HEIGHT	8.9	14		1	0
7	8322	48	Placebo	1	25	4/17/2019	81.6	HEIGHT	10.85	14.5		1	0
7	8322	60	Placebo	1	35	3/8/2020	88.7	HEIGHT	13.3	15.2		1	0
7	8347	48	Placebo	1	11	4/17/2019	69.5	LENGTH	8.2	15		1	0
7	8347	60	Placebo	1	20	2/9/2020	79.2	HEIGHT	10	14		1	0
7	8363	36	Placebo	0	8	6/11/2018	66.7	LENGTH	5.95	11		1	0
7	8363	48	Placebo	0	19	4/17/2019	77.7	LENGTH	9.45	13.5		1	0
7	8363	60	Placebo	0	28	2/9/2020	85.3	HEIGHT	12.3	15		1	0
7	8382	36	Placebo	0	15	6/11/2018	81.5	LENGTH	11.25	15		1	0
7	8382	48	Placebo	0	26	4/17/2019	88.4	HEIGHT	12.6	15.5		1	0
7	8382	60	Placebo	0	35	2/9/2020	95.3	HEIGHT	14.45	15.5		1	0
7	8542	36	Placebo	1	11	6/11/2018	70.9	LENGTH	8.35	13.5		1	0
7	8542	48	Placebo	1	15	4/17/2019	81.6	HEIGHT	10.1	13.5		1	0
7	8542	60	Placebo	1	25	2/9/2020	88.3	HEIGHT	12.7	15		1	0
7	8562	60	Placebo	1	50	2/9/2020	101.6	HEIGHT	15	15		1	0
7	8569	60	Placebo	0	50	2/9/2020	101.2	HEIGHT	13.75	12.7		1	0
7	8625	48	Placebo	0	12	4/17/2019	77.4	HEIGHT	9.2	13.5		1	0
7	8626	48	Placebo	0	32	4/17/2019	80.6	LENGTH	11.55	17		1	0
7	8626	60	Placebo	0	41	2/9/2020	87.2	HEIGHT	12.55	15.5		1	0
7	8638	48	Placebo	1	12	4/17/2019	73.7	LENGTH	8.8	15		1	0
7	8666	36	Placebo	1	10	6/11/2018	68.5	LENGTH	7.35	13	42	1	0
7	8728	60	Placebo	1	1	2/9/2020	58.5	LENGTH	5.3	12		1	0
7	8739	48	Placebo	1	12	4/17/2019	65.6	LENGTH	6.75	12.5		1	0
7	8739	60	Placebo	1	21	2/9/2020	73.4	HEIGHT	8.35	12.5		1	0
7	8755	36	Placebo	0	30	6/11/2018	81.2	LENGTH	10.6	14	48	1	0
7	8785	36	Placebo	0	12	6/11/2018	74.3	LENGTH	8.95	14		1	0
7	8785	48	Placebo	0	23	4/17/2019	84.5	LENGTH	11.8	16		1	0
7	8785	60	Placebo	0	33	2/9/2020	92.3	HEIGHT	14.35	16.5		1	0
7	8829	36	Placebo	1	14	6/11/2018	74.4	HEIGHT	8.75	13		1	0
7	8829	48	Placebo	1	25	4/17/2019	82.8	HEIGHT	10.5	13.5		1	0
7	8829	60	Placebo	1	35	2/9/2020	88.4	HEIGHT	12	14.5		1	0

7	8832	48	Placebo	0	14	4/17/2019	76.4	HEIGHT	10	14.5	1	0
7	8832	60	Placebo	0	23	3/8/2020	83.7	HEIGHT	12.75	15	1	0
7	8856	60	Placebo	0	11	2/9/2020	86.4	HEIGHT	9.5	12.5	1	0
7	8883	36	Placebo	0	7	6/11/2018	70.5	LENGTH	7.7	13	1	0
7	8883	48	Placebo	0	15	4/17/2019	81.7	HEIGHT	10	14.5	1	0
7	8883	60	Placebo	0	25	2/9/2020	90	HEIGHT	12	14	1	0
7	8884	36	Placebo	0	4	6/11/2018	61.4	LENGTH	5.7	12	1	0
7	8884	48	Placebo	0	12	4/17/2019	72.5	LENGTH	8.3	13.5	1	0
7	9060	48	Placebo	1	6	4/17/2019	65.5	LENGTH	7.1	14	1	0
7	9060	60	Placebo	1	14	2/9/2020	93.1	HEIGHT	8.5	13	1	0
7	9106	60	Placebo	1	10	3/8/2020	67.1	HEIGHT	6.55	12	1	0
7	9108	48	Placebo	0	42	4/17/2019	97.6	HEIGHT	13.15	15	1	0
7	9108	60	Placebo	0	51	2/9/2020	105.1	HEIGHT	14.7	14	1	0
7	9110	48	Placebo	1	12	4/17/2019	68.2	LENGTH	6.3	12	1	0
7	9110	60	Placebo	1	21	2/9/2020	76.9	LENGTH	8.3	12.5	1	0
7	9120	48	Placebo	0	4	4/17/2019	56.9	LENGTH	5.05	12	1	0
7	9120	60	Placebo	0	12	2/9/2020	59.9	LENGTH	7	12.3	1	0
7	9162	48	Placebo	1	1	4/17/2019	52.5	LENGTH	4.3	12	1	0
7	9162	60	Placebo	1	10	2/9/2020	67.1	LENGTH	7	12.8	1	0
7	9171	36	Placebo	1	24	6/11/2018	72.5	LENGTH	7.2	11	1	0
7	9171	48	Placebo	1	34	4/17/2019	79.2	LENGTH	9.6	12.5	1	0
7	9171	60	Placebo	1	44	2/9/2020	88.2	HEIGHT	11	12.3	1	0
7	9337	48	Placebo	1	24	4/17/2019	79.1	HEIGHT	9.15	13.5	1	0
7	9337	60	Placebo	1	33	2/9/2020	88	HEIGHT	11.2	15	1	0
7	9346	48	Placebo	1	1	4/17/2019	66	LENGTH	6.9	12.5	1	0
7	9346	60	Placebo	1	23	2/9/2020	97.8	HEIGHT	9.45	14	1	0
7	9363	60	Placebo	1	38	2/9/2020	86.8	HEIGHT	13.55	16	1	0
7	9474	36	Placebo	0	39	6/11/2018	99.2	HEIGHT	15.05	15.5	1	0
7	9474	60	Placebo	0	59	3/8/2020	110.8	HEIGHT	18.5	15	1	0
7	9510	36	Placebo	1	34	6/11/2018	85.3	HEIGHT	8.85	12	1	0
7	9510	48	Placebo	1	45	4/17/2019	91.8	HEIGHT	11.05	13.5	1	0
7	9510	60	Placebo	1	55	2/9/2020	100	HEIGHT	12.95	13.5	1	0
7	9519	60	Placebo	1	26	2/9/2020	82.4	HEIGHT	10.65	14.5	1	0
7	9563	60	Placebo	0	8	2/9/2020	73.2	LENGTH	8.2	13	1	0
7	9565	36	Placebo	0	7	6/11/2018	68.5	LENGTH	6.3	11.5	1	0
7	9565	60	Placebo	0	27	2/9/2020	86.6	HEIGHT	10.55	13	1	0
8	1829	0	Placebo	1	48	3/25/2015	99.2	HEIGHT	13.15	14.5	1	0
8	1829	12	Placebo	1	45	7/19/2016	108.9	HEIGHT	16.8	15	1	0
8	1832	0	Placebo	0	24	3/25/2015	90.3	HEIGHT	12.1	14.5	1	0
8	1833	0	Placebo	0	48	3/25/2015	89.3	HEIGHT	12.05	14.5	1	0

8	1833	12	Placebo	0	57	7/19/2016	97.6	HEIGHT	13.4	14	0	0
8	1833	24	Placebo	0	68	3/27/2017	100.4	HEIGHT	14.3	14	0	0
8	1833	36	Placebo	0	83	5/24/2018	107.2	HEIGHT	16.15	15	0	0
8	1833	48	Placebo	0	94	4/16/2019	110.9	HEIGHT	17.25	14.5	0	0
8	1833	60	Placebo	0	103	2/13/2020	127.9	HEIGHT	24.15	15.9	0	0
8	1836	0	Placebo	1	36	3/25/2015	89.8	HEIGHT	13	15	0	0
8	1836	12	Placebo	0	42	7/19/2016	101	HEIGHT	15.45	15	1	0
8	1836	24	Placebo	0	53	3/27/2017	106.3	HEIGHT	17	16.5	0	0
8	1836	36	Placebo	0	68	5/24/2018	113.5	HEIGHT	18.5	17	0	0
8	1836	48	Placebo	0	79	4/16/2019	118.4	HEIGHT	20.7	16.5	0	0
8	1836	60	Placebo	0	88	2/13/2020	121.5	HEIGHT	22.2	16.5	0	0
8	1837	12	Placebo	1	11	7/19/2016	72.1	LENGTH	7.5	13	1	0
8	1837	24	Placebo	0	18	3/27/2017	78	HEIGHT	8.7	13.5	0	0
8	1837	36	Placebo	0	37	5/24/2018	86	HEIGHT	10.75	14	0	0
8	1837	48	Placebo	0	48	4/16/2019	91.4	HEIGHT	11.45	14	0	0
8	1837	60	Placebo	0	57	2/13/2020	95.8	HEIGHT	12.3	13.5	0	0
8	1838	0	Placebo	0	36	3/25/2015	104.8	HEIGHT	15.5	14	1	0
8	1838	12	Placebo	0	45	7/19/2016	112.3	HEIGHT	18.1	14.5	0	0
8	1838	48	Placebo	0	82	4/16/2019	124.3	HEIGHT	23.1	16.5	0	0
8	1838	60	Placebo	0	91	2/13/2020	128.6	HEIGHT	24.35	16.5	0	0
8	1839	0	Placebo	0	24	6/11/2015	78.5	HEIGHT	9.8	15	0	0
8	1839	48	Placebo	0	57	4/16/2019	109	HEIGHT	18.35	16	0	0
8	1840	0	Placebo	0	54	3/25/2015	107.2	HEIGHT	17.15	15	0	0
8	1840	12	Placebo	0	54	7/19/2016	117.7	HEIGHT	20.35	14.5	1	0
8	1840	24	Placebo	0	65	3/27/2017	121.5	HEIGHT	21.5	15	0	0
8	1840	36	Placebo	0	80	5/24/2018	128.9	HEIGHT	24.5	16	0	0
8	1840	48	Placebo	0	91	4/16/2019	134	HEIGHT	27.6	17	0	0
8	1840	60	Placebo	0	100	2/13/2020	138.9	HEIGHT	31.6	17.5	0	0
8	1841	0	Placebo	1	48	3/25/2015	93.1	HEIGHT	14.3	15.5	1	0
8	1841	12	Placebo	1	42	7/19/2016	103.5	HEIGHT	17.05	15.5	0	0
8	1841	24	Placebo	1	53	3/27/2017	110.2	HEIGHT	19	15.5	0	0
8	1841	36	Placebo	1	68	5/24/2018	121.6	HEIGHT	24.15	19	0	0
8	1841	48	Placebo	1	79	4/16/2019	126.8	HEIGHT	26.5	19	0	0
8	1841	60	Placebo	1	88	2/13/2020	130	HEIGHT	29.6	19	0	0
8	1843	0	Placebo	1	48	6/11/2015	96.3	HEIGHT	16.3	15.5	0	0
8	1843	12	Placebo	1	57	7/19/2016	104.3	HEIGHT	17.4	15	0	0
8	1843	24	Placebo	1	60	3/27/2017	109.2	HEIGHT	19.55	15.5	1	0
8	1843	36	Placebo	1	75	5/24/2018	116.3	HEIGHT	20.15	15.5	0	0
8	1843	48	Placebo	1	86	4/16/2019	121.2	HEIGHT	23.75	17	0	0
8	1843	60	Placebo	1	96	2/13/2020	126.7	HEIGHT	26.7	17.5	0	0

8	1844	0	Placebo	0	48	3/25/2015	93.5	HEIGHT	12.55	14.5	1	0
8	1844	12	Placebo	0	57	7/19/2016	102.2	HEIGHT	13.75	14	1	0
8	1844	24	Placebo	0	54	3/27/2017	106.3	HEIGHT	14.85	14	1	0
8	1844	36	Placebo	0	69	5/24/2018	112.9	HEIGHT	17.6	16	0	0
8	1844	48	Placebo	0	104	4/16/2019	117.2	HEIGHT	18.95	15.5	0	0
8	1844	60	Placebo	0	114	2/13/2020	121.4	HEIGHT	20.3	15.5	0	0
8	1846	0	Placebo	0	48	3/25/2015	100.7	HEIGHT	13.85	14	1	0
8	1846	12	Placebo	0	57	7/19/2016	107.9	HEIGHT	15.9	14.5	0	0
8	1846	24	Placebo	0	68	3/27/2017	110.7	HEIGHT	16.8	14	0	0
8	1846	36	Placebo	0	83	5/24/2018	115.8	HEIGHT	18.36363636	16	0	0
8	1846	48	Placebo	0	94	4/16/2019	118.5	HEIGHT	19.65	15.5	0	0
8	1849	24	Placebo	0	9	3/27/2017	70.4	HEIGHT	7.45	13	1	0
8	1851	0	Placebo	0	36	3/25/2015	81.3	HEIGHT	9.7	14	0	0
8	1851	12	Placebo	0	49	7/19/2016	91.8	HEIGHT	12.5	14	0	0
8	1851	24	Placebo	0	60	3/27/2017	97.8	HEIGHT	13.9	14.5	0	0
8	1851	36	Placebo	0	75	5/24/2018	106.6	HEIGHT	15.81818182	15.5	0	0
8	1851	48	Placebo	0	85	4/16/2019	111.1	HEIGHT	17	14.5	0	0
8	1851	60	Placebo	0	95	2/13/2020	116.7	HEIGHT	20.5	16	0	0
8	1855	0	Placebo	0	10	3/25/2015	73.6	LENGTH	10.1	15.5	1	0
8	1855	12	Placebo	0	18	7/19/2016	84.6	HEIGHT	11.8	15	0	0
8	1855	24	Placebo	0	29	3/27/2017	89.9	HEIGHT	13.3	15.5	1	0
8	1855	36	Placebo	0	44	5/24/2018	97.6	HEIGHT	14.36363636	15	1	0
8	1855	48	Placebo	0	55	4/16/2019	104.2	HEIGHT	16.9	16	1	0
8	1855	60	Placebo	0	64	2/13/2020	108.9	HEIGHT	18.5	15.9	0	0
8	1859	0	Placebo	1	30	3/25/2015	109.7	HEIGHT	19.6	15.5	0	0
8	1859	48	Placebo	0	91	4/16/2019	120	HEIGHT	22.25	16.5	0	0
8	1859	60	Placebo	0	100	2/13/2020	124.1	HEIGHT	24.85	17	0	0
8	1860	12	Placebo	0	51	7/19/2016	107	HEIGHT	17.05	15	1	0
8	1864	0	Placebo	0	2	3/25/2015	59.8	LENGTH	7	14	1	0
8	1864	12	Placebo	0	12	7/19/2016	78.5	LENGTH	10.25	14	0	0
8	1864	24	Placebo	0	21	3/27/2017	84.6	HEIGHT	12.4	15.5	1	0
8	1864	36	Placebo	0	36	5/24/2018	96.3	HEIGHT	15.9	17	1	0
8	1864	48	Placebo	0	46	4/16/2019	102.8	HEIGHT	16.9	16	1	0
8	1864	60	Placebo	0	56	2/13/2020	109.6	HEIGHT	18.7	15	0	0
8	1866	0	Placebo	1	48	3/25/2015	125.3	HEIGHT	20.45	16	1	0
8	1866	24	Placebo	1	79	3/27/2017	135.5	HEIGHT	25.25	17	0	0
8	1869	0	Placebo	0	12	3/25/2015	70.5	LENGTH	7.7	12.5	0	0
8	1869	12	Placebo	0	30	7/19/2016	85	LENGTH	11.15	13.5	0	0
8	1869	24	Placebo	0	41	3/27/2017	87.6	HEIGHT	12.1	14	1	0
8	1869	48	Placebo	0	67	4/16/2019	98.9	HEIGHT	14.65	14	0	0

8	1869	60	Placebo	0	76	2/13/2020	103.6	HEIGHT	15.5	13.5	0	0
8	1870	0	Placebo	1	36	3/25/2015	95.6	HEIGHT	13.8	14	0	0
8	1872	12	Placebo	1	30	7/19/2016	89.7	HEIGHT	12.05	14	1	0
8	1872	36	Placebo	1	56	5/24/2018	104.8	HEIGHT	15.9	15	1	0
8	1874	24	Placebo	1	1	3/27/2017	54.5	LENGTH	5.2	12	1	0
8	1874	48	Placebo	1	26	4/16/2019	84.2	HEIGHT	10.95	13.5	0	0
8	1874	60	Placebo	1	36	2/13/2020	91.2	HEIGHT	12.15	13.6	1	0
8	1876	24	Placebo	1	53	3/27/2017	88.8	HEIGHT	11.35	13.5	1	0
8	1877	24	Placebo	0	53	3/27/2017	95.3	HEIGHT	14.05	14.5	1	0
8	1878	0	Placebo	1	36	3/25/2015	110.5	HEIGHT	18.55	16	0	0
8	1878	12	Placebo	1	45	7/19/2016	120.5	HEIGHT	21.95	16.5	0	0
8	1878	24	Placebo	1	56	3/27/2017	124.7	HEIGHT	23.6	16	0	0
8	1878	36	Placebo	1	71	5/24/2018	131.9	HEIGHT	25.5	17	0	0
8	1878	48	Placebo	1	82	4/16/2019	135.7	HEIGHT	28.05	18	0	0
8	1878	60	Placebo	1	91	2/13/2020	139.9	HEIGHT	30.7	18	0	0
8	1879	24	Placebo	0	20	3/27/2017	84	HEIGHT	10.45	13.5	1	0
8	1879	48	Placebo	0	46	4/16/2019	100.9	HEIGHT	15.4	15	1	0
8	1883	0	Placebo	1	30	3/25/2015	83.4	HEIGHT	10	14.5	0	0
8	1883	36	Placebo	1	69	5/24/2018	108.6	HEIGHT	18	17.5	0	0
8	1884	0	Placebo	1	54	6/11/2015	114	HEIGHT	19.55	16.5	0	0
8	1884	12	Placebo	1	68	7/19/2016	121.6	HEIGHT	20.6	16	0	0
8	1884	24	Placebo	1	77	3/27/2017	123.2	HEIGHT	22.3	17	0	0
8	1884	36	Placebo	1	92	5/24/2018	129.1	HEIGHT	23.36363636	17.5	0	0
8	1884	48	Placebo	1	103	4/16/2019	131.9	HEIGHT	25.1	18	0	0
8	1884	60	Placebo	1	112	2/13/2020	135.3	HEIGHT	27.55	17.8	0	0
8	1887	24	Placebo	0	53	3/27/2017	110.9	HEIGHT	19.3	16	1	0
8	1888	24	Placebo	1	6	3/27/2017	64.8	LENGTH	6.05	12	1	0
8	1888	36	Placebo	1	21	5/24/2018	76.2	LENGTH	8.181818182	12.5	0	0
8	1888	48	Placebo	1	32	4/16/2019	81.3	HEIGHT	10.1	13.5	1	0
8	1888	60	Placebo	1	41	2/13/2020	88.6	HEIGHT	11.6	14	1	0
8	1890	0	Placebo	1	54	3/25/2015	109.5	HEIGHT	18.3	16.5	0	0
8	1890	36	Placebo	1	88	5/24/2018	125.4	HEIGHT	25.45454545	18.5	0	0
8	1890	48	Placebo	1	99	4/16/2019	128.9	HEIGHT	27.3	18.5	0	0
8	1890	60	Placebo	1	108	2/13/2020	134.3	HEIGHT	32.4	20	0	0
8	1892	0	Placebo	1	12	3/25/2015	72.6	HEIGHT	8.5	13.5	1	0
8	1892	12	Placebo	1	24	7/19/2016	83	HEIGHT	10.45	13.5	0	0
8	1892	24	Placebo	1	35	3/27/2017	89.8	HEIGHT	11.65	13.5	0	0
8	1892	36	Placebo	1	50	5/24/2018	96.8	HEIGHT	14.27272727	15.5	1	0
8	1893	0	Placebo	1	24	3/25/2015	92.1	HEIGHT	12.9	16	0	0
8	1893	12	Placebo	1	56	7/19/2016	102.7	HEIGHT	15.2	15	0	0

8	1893	24	Placebo	1	65	3/27/2017	106.5	HEIGHT	15.95	15	0	0
8	1893	36	Placebo	1	80	5/24/2018	114.8	HEIGHT	18.5	16	0	0
8	1893	48	Placebo	1	91	4/16/2019	120.3	HEIGHT	21.15	17	0	0
8	1893	60	Placebo	1	100	2/13/2020	125.7	HEIGHT	23.05	16.5	0	0
8	1894	0	Placebo	1	6	3/25/2015	63.5	LENGTH	6	13	1	0
8	1894	12	Placebo	1	18	7/19/2016	77.1	HEIGHT	8.4	12	0	0
8	1894	24	Placebo	1	29	3/27/2017	80.4	HEIGHT	9.7	13	1	0
8	1894	36	Placebo	1	44	5/24/2018	88.8	HEIGHT	12.45454545	15	1	0
8	1894	48	Placebo	1	54	4/16/2019	94.7	HEIGHT	13.8	15	0	0
8	1894	60	Placebo	1	64	2/13/2020	99.9	HEIGHT	15.35	15.4	0	0
8	1895	0	Placebo	0	48	3/25/2015	92.8	HEIGHT	14.2	17	1	0
8	1895	12	Placebo	0	57	7/19/2016	101.6	HEIGHT	13.95	14	1	0
8	1895	24	Placebo	0	68	3/27/2017	104.1	HEIGHT	16.2	15	0	0
8	1895	36	Placebo	0	83	5/24/2018	110.7	HEIGHT	18.5	16.5	0	0
8	1895	48	Placebo	0	94	4/16/2019	115.1	HEIGHT	20.7	16.5	0	0
8	1895	60	Placebo	0	103	2/13/2020	118.5	HEIGHT	21.6	16.1	0	0
8	1897	0	Placebo	0	48	3/25/2015	103.3	HEIGHT	15.6	14	1	0
8	1897	12	Placebo	0	57	7/19/2016	111.2	HEIGHT	16.85	14	0	0
8	1897	24	Placebo	0	68	3/27/2017	115.6	HEIGHT	19.1	14.5	0	0
8	1897	36	Placebo	0	83	5/24/2018	121.2	HEIGHT	21.18181818	16	0	0
8	1897	48	Placebo	0	94	4/16/2019	125.3	HEIGHT	22.5	15.5	0	0
8	1897	60	Placebo	0	103	2/13/2020	128.1	HEIGHT	23.6	15	0	0
8	1898	12	Placebo	1	30	7/19/2016	74.2	HEIGHT	12.25	13	1	0
8	1898	36	Placebo	1	56	5/24/2018	107.7	HEIGHT	15.09090909	14	1	0
8	1900	0	Placebo	0	48	3/25/2015	124.4	HEIGHT	23.4	16.5	1	0
8	1900	36	Placebo	0	130	5/24/2018	145.5	HEIGHT	29.65	18	0	0
8	1900	48	Placebo	0	141	4/16/2019	144.5	HEIGHT	31.85	18	0	0
8	1902	0	Placebo	1	36	3/25/2015	95.5	HEIGHT	13.9	15	1	0
8	1902	12	Placebo	1	66	7/19/2016	105.9	HEIGHT	15.65	14	0	0
8	1902	24	Placebo	1	77	3/27/2017	109.2	HEIGHT	17.7	15	0	0
8	1902	36	Placebo	1	92	5/24/2018	116.7	HEIGHT	19.55	15	0	0
8	1902	48	Placebo	1	103	4/16/2019	120.7	HEIGHT	20.75	16	0	0
8	1902	60	Placebo	1	112	2/13/2020	124.4	HEIGHT	21.95	15.5	0	0
8	1904	12	Placebo	0	2	7/19/2016	65.4	LENGTH	6.55	13	1	0
8	1904	24	Placebo	0	12	3/27/2017	73.7	HEIGHT	7.45	13.5	0	0
8	1905	12	Placebo	0	12	7/19/2016	72.2	LENGTH	7.6	13	1	0
8	1905	24	Placebo	1	20	3/27/2017	79.1	HEIGHT	10.35	13.5	0	0
8	1907	0	Placebo	0	5	6/11/2015	62.2	LENGTH	5.7	12.5	0	0
8	1907	24	Placebo	0	29	3/27/2017	79.7	HEIGHT	9.95	13.5	0	0
8	1909	0	Placebo	0	54	3/25/2015	116.1	HEIGHT	19.3	16	0	0

8	1909	12	Placebo	0	92	7/19/2016	121.7	HEIGHT	21.35	16		0	0
8	1909	24	Placebo	0	101	3/27/2017	124	HEIGHT	22.75	17		0	0
8	1909	36	Placebo	0	116	5/24/2018	129	HEIGHT	25.04545455	18		0	0
8	1909	48	Placebo	0	127	4/16/2019	132.1	HEIGHT	27.05	18		0	0
8	1909	60	Placebo	0	136	2/13/2020	134.6	HEIGHT	28.95	18.2		0	0
8	1911	0	Placebo	0	12	3/25/2015	77.5	LENGTH	8.85	14.5	42	0	0
8	1911	12	Placebo	0	25	7/19/2016	85.4	HEIGHT	10.5	13	42	0	0
8	1911	24	Placebo	0	36	3/27/2017	91.2	HEIGHT	12.95	15	42	0	0
8	1913	0	Placebo	1	54	3/25/2015	101.4	HEIGHT	13.1	13.5		0	0
8	1913	12	Placebo	0	63	7/19/2016	112.1	HEIGHT	16.15	13.5		0	0
8	1913	36	Placebo	0	89	5/24/2018	123.5	HEIGHT	19.72727273	15.5		0	0
8	1914	0	Placebo	1	36	3/25/2015	90.8	HEIGHT	11.35	14.5		0	0
8	1914	12	Placebo	1	44	7/19/2016	100.7	HEIGHT	12.7	13		0	0
8	1914	24	Placebo	1	55	3/27/2017	105.9	HEIGHT	14.1	13.5		1	0
8	1914	36	Placebo	1	70	5/24/2018	113.2	HEIGHT	15.7	14.5		0	0
8	1914	48	Placebo	1	80	4/16/2019	118.1	HEIGHT	16.95	14		0	0
8	1915	0	Placebo	1	24	3/25/2015	82.3	HEIGHT	9.95	14		0	0
8	1915	36	Placebo	1	68	5/24/2018	106.9	HEIGHT	14.85	15		0	0
8	1916	24	Placebo	1	30	3/27/2017	86.2	HEIGHT	11.05	13.5		1	0
8	1918	12	Placebo	0	15	7/19/2016	82	HEIGHT	10.9	14		1	0
8	1918	24	Placebo	0	26	3/27/2017	87.2	HEIGHT	12	14.5		0	0
8	1918	36	Placebo	0	41	5/24/2018	95.3	HEIGHT	14.68181818	16		1	0
8	1918	60	Placebo	0	61	2/13/2020	107.9	HEIGHT	17.8	15.5		0	0
8	1921	0	Placebo	0	12	3/25/2015	67.2	LENGTH	7.4	14.5		0	0
8	1921	12	Placebo	0	24	7/19/2016	78.7	LENGTH	9.6	14		1	0
8	1921	36	Placebo	0	45	5/24/2018	88.9	HEIGHT	11.95454545	15		1	0
8	1921	48	Placebo	0	56	4/16/2019	93.9	HEIGHT	12.15	15		0	0
8	1921	60	Placebo	0	65	2/13/2020	98.8	HEIGHT	14.2	14.3		0	0
8	1922	12	Placebo	1	42	7/19/2016	123.8	HEIGHT	20.1	15		1	0
8	1923	0	Placebo	1	36	3/25/2015	80.5	LENGTH	9.9	13.5		1	0
8	1923	12	Placebo	1	42	7/19/2016	95	HEIGHT	13.05	14		0	0
8	1923	24	Placebo	1	53	3/27/2017	101.2	HEIGHT	15.55	15		1	0
8	1923	36	Placebo	1	68	5/24/2018	111.3	HEIGHT	16.85	15		0	0
8	1923	48	Placebo	1	79	4/16/2019	116.3	HEIGHT	18.4	15.5		0	0
8	1923	60	Placebo	1	88	2/13/2020	122.2	HEIGHT	21.6	16		0	0
8	1924	0	Placebo	1	48	3/25/2015	121.9	HEIGHT	22.4	17		0	0
8	1924	24	Placebo	1	79	3/27/2017	132	HEIGHT	27.95	19		0	0
8	1925	0	Placebo	1	24	3/25/2015	77.7	HEIGHT	10.4	14.5		0	0
8	1925	12	Placebo	1	33	7/19/2016	88.7	HEIGHT	14.55	16		0	0
8	1925	24	Placebo	1	44	3/27/2017	94.3	HEIGHT	15.6	16		1	0

8	1925	36	Placebo	1	59	5/24/2018	102.7	HEIGHT	17.22727273	16.5		0	0
8	1925	48	Placebo	1	69	4/16/2019	108.6	HEIGHT	18.35	16		0	0
8	1925	60	Placebo	1	79	2/13/2020	113.4	HEIGHT	20.2	16.5		0	0
8	1928	0	Placebo	1	12	3/25/2015	81	HEIGHT	10.2	15		1	0
8	1928	36	Placebo	1	68	5/24/2018	100.7	HEIGHT	15.18181818	15.5		0	0
8	1928	48	Placebo	1	79	4/16/2019	105.7	HEIGHT	16.9	15.5		0	0
8	1929	0	Placebo	0	36	3/25/2015	77.6	HEIGHT	8.55	12	18	0	0
8	1930	0	Placebo	0	24	3/25/2015	86.5	HEIGHT	10.95	14		0	0
8	1930	12	Placebo	0	33	7/19/2016	96	HEIGHT	13.1	15		1	0
8	1930	24	Placebo	0	44	3/27/2017	98.8	HEIGHT	13.75	14.5		0	0
8	1930	36	Placebo	0	59	5/24/2018	104.9	HEIGHT	15.77272727	15		0	0
8	1931	0	Placebo	1	24	6/11/2015	76.5	HEIGHT	8.1	13.5	18	0	0
8	1932	0	Placebo	0	36	3/25/2015	94.3	HEIGHT	14.1	14.5		0	0
8	1932	24	Placebo	0	56	3/27/2017	110.5	HEIGHT	18	16		0	0
8	1932	36	Placebo	0	71	5/24/2018	118.7	HEIGHT	19.3	16		0	0
8	1932	48	Placebo	0	82	4/16/2019	124.1	HEIGHT	22	16.5		0	0
8	1932	60	Placebo	0	91	2/13/2020	127.5	HEIGHT	24.5	16.1		0	0
8	1934	12	Placebo	0	30	7/19/2016	88.9	HEIGHT	11.65	13		1	0
8	1936	24	Placebo	0	10	3/27/2017	69.6	LENGTH	7.75	13.5		1	0
8	1936	36	Placebo	0	21	5/24/2018	79.5	LENGTH	10.05	16		1	0
8	1936	48	Placebo	0	32	4/16/2019	85.4	HEIGHT	12	15		0	0
8	1936	60	Placebo	0	45	2/13/2020	91.9	HEIGHT	13.45	15.5		1	0
8	1940	0	Placebo	0	48	3/25/2015	103.9	HEIGHT	15.75	15.5		0	0
8	1940	12	Placebo	0	56	7/19/2016	112.3	HEIGHT	16.95	15		1	0
8	1940	24	Placebo	0	65	3/27/2017	116.2	HEIGHT	18.7	15		0	0
8	1940	36	Placebo	0	80	5/24/2018	122.9	HEIGHT	20.77272727	16		0	0
8	1940	48	Placebo	0	91	4/16/2019	126.8	HEIGHT	23.05	16		0	0
8	1940	60	Placebo	0	100	2/13/2020	131.4	HEIGHT	25.4	16.7		0	0
8	1941	0	Placebo	1	24	3/25/2015	84.5	HEIGHT	12.85	15.5		0	0
8	1947	0	Placebo	1	36	3/25/2015	100.8	HEIGHT	14.1	14.5		1	0
8	1947	12	Placebo	1	45	7/19/2016	110.4	HEIGHT	16.4	14		0	0
8	1950	0	Placebo	1	12	6/11/2015	81.3	HEIGHT	10.65	15.5		0	0
8	1950	12	Placebo	1	21	7/19/2016	90.4	HEIGHT	14.2	16		0	0
8	1950	24	Placebo	0	41	3/27/2017	93.5	HEIGHT	15.2	16.5		1	0
8	1950	36	Placebo	0	56	5/24/2018	102.2	HEIGHT	16.95	16		1	0
8	1953	12	Placebo	0	10	7/19/2016	80.3	LENGTH	12.15	16.5		1	0
8	1953	24	Placebo	0	18	3/27/2017	87.5	HEIGHT	14.35	17		0	0
8	1953	36	Placebo	0	33	5/24/2018	99	HEIGHT	17.63636364	17		0	0
8	1953	48	Placebo	0	44	4/16/2019	104.3	HEIGHT	19.35	17.5		1	0
8	1957	0	Placebo	0	24	3/25/2015	69.8	LENGTH	7.7	14		1	0

8	1957	12	Placebo	0	27	7/19/2016	80.5	HEIGHT	10.25	13.5	0	0
8	1957	24	Placebo	0	38	3/27/2017	86	HEIGHT	11.45	14	0	0
8	1957	36	Placebo	0	49	5/24/2018	93.4	HEIGHT	13.45	14	0	0
8	1957	48	Placebo	0	60	4/16/2019	97.8	HEIGHT	13.65	14	0	0
8	1957	60	Placebo	0	70	2/13/2020	101.9	HEIGHT	14.95	14	0	0
8	1958	0	Placebo	0	48	6/11/2015	83.7	HEIGHT	10.15	13.5	0	0
8	1958	12	Placebo	0	61	7/19/2016	89.5	HEIGHT	12.1	15	0	0
8	1958	24	Placebo	0	50	3/27/2017	94.4	HEIGHT	12.65	14	0	0
8	1960	12	Placebo	1	8	7/19/2016	71.7	LENGTH	8.3	14.5	0	0
8	1960	24	Placebo	1	14	3/27/2017	78.5	HEIGHT	9.55	14	0	0
8	1960	36	Placebo	1	29	5/24/2018	86.8	HEIGHT	11.75	15.5	0	0
8	1960	48	Placebo	1	44	4/16/2019	94	HEIGHT	13.4	15.5	1	0
8	1960	60	Placebo	1	53	2/13/2020	101.7	HEIGHT	14.65	15	0	0
8	1961	0	Placebo	1	36	3/25/2015	114.4	HEIGHT	17.25	15	0	0
8	1961	36	Placebo	1	94	5/24/2018	131.3	HEIGHT	23.85	16.5	0	0
8	1964	0	Placebo	0	24	3/25/2015	66.3	LENGTH	7.2	14.5	0	0
8	1965	0	Placebo	1	48	3/25/2015	107.8	HEIGHT	18.3	17	0	0
8	1965	12	Placebo	1	78	7/19/2016	117.4	HEIGHT	19.75	16.5	0	0
8	1965	36	Placebo	1	72	5/24/2018	126.8	HEIGHT	26.15	19	0	0
8	1965	48	Placebo	1	82	4/16/2019	131	HEIGHT	28.65	19	0	0
8	1967	24	Placebo	1	29	3/27/2017	92.4	HEIGHT	12.9	15	1	0
8	1967	48	Placebo	1	55	4/16/2019	104.6	HEIGHT	16.3	15	1	0
8	1969	0	Placebo	0	48	3/25/2015	105.6	HEIGHT	17.75	14.5	0	0
8	1969	24	Placebo	0	74	3/27/2017	117	HEIGHT	22.2	15	0	0
8	1969	36	Placebo	0	89	5/24/2018	123.9	HEIGHT	24.95	16	0	0
8	1970	24	Placebo	1	5	3/27/2017	62.1	LENGTH	6.3	12	1	0
8	1970	36	Placebo	1	15	5/24/2018	75.5	LENGTH	9.1	13.5	1	0
8	1970	48	Placebo	1	26	4/16/2019	81.1	HEIGHT	9.95	14	0	0
8	1970	60	Placebo	1	35	2/13/2020	87.6	HEIGHT	11.95	14.5	0	0
8	1971	12	Placebo	1	30	7/19/2016	111.9	HEIGHT	16.5	14.5	1	0
8	1971	24	Placebo	1	41	3/27/2017	115.4	HEIGHT	19.35	14.5	1	0
8	1971	36	Placebo	1	56	5/24/2018	120.9	HEIGHT	21.1	15.5	1	0
8	1975	0	Placebo	0	48	3/25/2015	90.7	HEIGHT	13.6	15.5	1	0
8	1975	12	Placebo	0	59	7/19/2016	102	HEIGHT	15.8	15.5	1	0
8	1975	24	Placebo	0	68	3/27/2017	104.9	HEIGHT	16.85	15.5	0	0
8	1975	36	Placebo	0	83	5/24/2018	111.1	HEIGHT	18.55	15.5	0	0
8	1975	48	Placebo	0	94	4/16/2019	116.1	HEIGHT	19.6	14.5	0	0
8	1975	60	Placebo	0	103	2/13/2020	119.8	HEIGHT	21.5	15	0	0
8	1976	12	Placebo	0	12	7/19/2016	76.5	LENGTH	8.4	12.5	1	0
8	1977	0	Placebo	1	48	3/25/2015	127.2	HEIGHT	23.2	15.5	0	0

8	1977	48	Placebo	1	120	4/16/2019	138.6	HEIGHT	29.95	18		0	0
8	1977	60	Placebo	1	130	2/13/2020	144.5	HEIGHT	33.1	19		0	0
8	1979	0	Placebo	0	24	6/11/2015	87.3	HEIGHT	12.2	15		0	0
8	1979	12	Placebo	0	33	7/19/2016	96.2	HEIGHT	13.55	14		0	0
8	1979	24	Placebo	0	44	3/27/2017	101.4	HEIGHT	14.55	13.5		1	0
8	1979	36	Placebo	0	59	5/24/2018	108.7	HEIGHT	15.81818182	13.5		0	0
8	1979	48	Placebo	0	70	4/16/2019	114.5	HEIGHT	17.85	13.5		0	0
8	1979	60	Placebo	0	79	2/13/2020	119.4	HEIGHT	20.6	14.2		0	0
8	1980	24	Placebo	0	20	3/27/2017	88.5	HEIGHT	12.5	14.5	30	1	0
8	1981	0	Placebo	0	48	3/25/2015	108	HEIGHT	15.2	14.5		0	0
8	1981	36	Placebo	0	116	5/24/2018	124.4	HEIGHT	19.8	15		0	0
8	1982	0	Placebo	1	48	3/25/2015	106.4	HEIGHT	15.2	13.5		1	0
8	1982	12	Placebo	1	57	7/19/2016	112.8	HEIGHT	16.2	13		1	0
8	1982	36	Placebo	1	83	5/24/2018	122.2	HEIGHT	20.22727273	15.5		0	0
8	1982	48	Placebo	1	94	4/16/2019	124.3	HEIGHT	22.65	16		0	0
8	1982	60	Placebo	1	103	2/13/2020	128.4	HEIGHT	23.85	16		0	0
8	1983	24	Placebo	0	9	3/27/2017	66.6	LENGTH	7.05	12.5		1	0
8	1983	36	Placebo	0	24	5/24/2018	82.7	HEIGHT	10.31818182	15		1	0
8	1983	48	Placebo	0	35	4/16/2019	86.2	HEIGHT	11.95	15		1	0
8	1990	0	Placebo	1	48	3/25/2015	101.6	HEIGHT	15.35	14.5		0	0
8	1990	12	Placebo	1	63	7/19/2016	113.1	HEIGHT	18.35	14.5		0	0
8	1990	24	Placebo	0	74	3/27/2017	116.5	HEIGHT	19.7	15.5		0	0
8	1990	60	Placebo	0	109	2/13/2020	132.9	HEIGHT	26.85	17.5		0	0
8	1991	0	Placebo	0	48	3/25/2015	119.2	HEIGHT	17.95	14.5		1	0
8	1991	24	Placebo	1	77	3/27/2017	127.6	HEIGHT	21.5	15		0	0
8	1991	36	Placebo	1	92	5/24/2018	133.5	HEIGHT	24.27272727	16		0	0
8	1991	48	Placebo	1	103	4/16/2019	136.7	HEIGHT	25.5	16		0	0
8	1991	60	Placebo	1	112	2/13/2020	139.5	HEIGHT	27.3	16.5		0	0
8	1992	0	Placebo	1	12	3/25/2015	71.2	LENGTH	6.7	13		1	0
8	1992	12	Placebo	1	21	7/19/2016	83	HEIGHT	8.9	12		1	0
8	1992	24	Placebo	1	41	3/27/2017	87.8	HEIGHT	9.65	12.5		0	0
8	1992	48	Placebo	1	57	4/16/2019	101.3	HEIGHT	11.95	13		1	0
8	1992	60	Placebo	1	67	2/13/2020	107.6	HEIGHT	14.15	13.4		0	0
8	1993	12	Placebo	1	42	7/19/2016	113.5	HEIGHT	17.85	15.5		1	0
8	1994	0	Placebo	0	54	3/25/2015	94	HEIGHT	12.7	14.5		1	0
8	1994	12	Placebo	0	47	7/19/2016	102	HEIGHT	14.6	14		1	0
8	1994	24	Placebo	0	56	3/27/2017	106.5	HEIGHT	16.15	14.5		0	0
8	1994	36	Placebo	0	71	5/24/2018	113.4	HEIGHT	18.05	15		0	0
8	1994	48	Placebo	0	82	4/16/2019	116.7	HEIGHT	19.25	15.5		0	0
8	1996	12	Placebo	0	8	7/19/2016	73.8	LENGTH	7.5	13		0	0

8	1996	24	Placebo	0	14	3/27/2017	79	HEIGHT	8.65	12.5		1	0
8	1996	36	Placebo	0	29	5/24/2018	86.5	HEIGHT	10.85	13		0	0
8	1996	60	Placebo	0	51	2/13/2020	101.6	LENGTH	14.4	14		0	0
8	1998	0	Placebo	1	9	3/25/2015	72.1	LENGTH	9	13.5		1	0
8	1998	12	Placebo	1	18	7/19/2016	87.2	HEIGHT	10.7	13		1	0
8	1998	24	Placebo	1	38	3/27/2017	90.3	HEIGHT	12.2	14.5		0	0
8	1998	48	Placebo	1	64	4/16/2019	103.9	HEIGHT	15.35	14		0	0
8	1998	60	Placebo	1	74	2/13/2020	109.2	HEIGHT	16.6	13.2		0	0
8	2000	0	Placebo	1	12	3/25/2015	77.9	HEIGHT	9.35	14		1	0
8	2000	12	Placebo	1	23	7/19/2016	89.8	HEIGHT	11.9	14		1	0
8	2000	24	Placebo	1	32	3/27/2017	95.6	HEIGHT	13.15	14.5		1	0
8	2000	36	Placebo	1	47	5/24/2018	104.1	HEIGHT	15.27272727	15		0	0
8	2000	48	Placebo	1	58	4/16/2019	107.3	HEIGHT	17	15		0	0
8	2000	60	Placebo	1	67	2/13/2020	113	HEIGHT	18	14.7		0	0
8	2001	0	Placebo	1	7	3/25/2015	68.3	LENGTH	8	14.5		0	0
8	2001	12	Placebo	1	18	7/19/2016	81.2	HEIGHT	10.2	14		1	0
8	2001	24	Placebo	1	29	3/27/2017	86.8	HEIGHT	11.5	14		0	0
8	2001	36	Placebo	1	44	5/24/2018	95.6	HEIGHT	13.31818182	15		1	0
8	2001	48	Placebo	1	55	4/16/2019	101.2	HEIGHT	14.85	15		1	0
8	2002	0	Placebo	0	36	3/25/2015	89.4	HEIGHT	11.9	14		0	0
8	2002	12	Placebo	0	54	7/19/2016	100	HEIGHT	13.65	13.5		0	0
8	2002	24	Placebo	0	60	3/27/2017	103.2	HEIGHT	14.5	13.5		0	0
8	2002	36	Placebo	0	75	5/24/2018	109.9	HEIGHT	16.25	14.5		0	0
8	2002	48	Placebo	0	85	4/16/2019	112.7	HEIGHT	17.1	15		0	0
8	2002	60	Placebo	0	95	2/13/2020	117.4	HEIGHT	19.7	15.5		0	0
8	2003	0	Placebo	1	3	3/25/2015	60.6	LENGTH	5.65	12.5	12	0	0
8	2004	0	Placebo	1	48	3/25/2015	100	HEIGHT	13.6	15		1	0
8	2006	12	Placebo	1	15	7/19/2016	77.1	LENGTH	8.2	12.5		1	0
8	2007	0	Placebo	1	24	3/25/2015	76.7	HEIGHT	8.9	13.5		1	0
8	2007	12	Placebo	1	33	7/19/2016	88	HEIGHT	10.7	13.5		0	0
8	2007	24	Placebo	1	44	3/27/2017	93	HEIGHT	12.65	14.5		1	0
8	2007	36	Placebo	1	59	5/24/2018	100.9	HEIGHT	14.15	14.5		0	0
8	2007	48	Placebo	1	56	4/16/2019	106.4	HEIGHT	16	14.5		0	0
8	2007	60	Placebo	1	66	2/13/2020	112.3	HEIGHT	16.1	14		0	0
8	2008	12	Placebo	0	5	7/19/2016	62.4	LENGTH	5.55	12		0	0
8	2008	24	Placebo	1	25	3/27/2017	67.8	HEIGHT	6.6	12		1	0
8	2008	36	Placebo	1	40	5/24/2018	79.1	LENGTH	9.318181818	14		1	0
8	2009	12	Placebo	0	12	7/19/2016	74.6	LENGTH	7.05	11		1	0
8	2009	24	Placebo	0	18	3/27/2017	79.1	LENGTH	9	12		1	0
8	2009	36	Placebo	0	32	5/24/2018	90.2	HEIGHT	10.90909091	13		1	0

8	2009	48	Placebo	0	43	4/16/2019	94.6	HEIGHT	12.75	13		1	0
8	2009	60	Placebo	0	53	2/13/2020	100.3	HEIGHT	14.05	13		0	0
8	2011	0	Placebo	0	36	6/11/2015	95.4	HEIGHT	12.3	14.5		0	0
8	2011	12	Placebo	0	48	7/19/2016	104.1	HEIGHT	13.65	13.5		1	0
8	2011	24	Placebo	0	56	3/27/2017	108	HEIGHT	14.1	13.5		0	0
8	2011	36	Placebo	0	92	5/24/2018	115.5	HEIGHT	16.31818182	14		0	0
8	2011	48	Placebo	0	103	4/16/2019	119.8	HEIGHT	17.4	14		0	0
8	2011	60	Placebo	0	112	2/13/2020	124.3	HEIGHT	19.3	14.4		0	0
8	2012	0	Placebo	0	24	6/11/2015	89.1	HEIGHT	12.8	14.5		0	0
8	2014	0	Placebo	0	48	6/11/2015	129.8	HEIGHT	21.1	15		0	0
8	2014	12	Placebo	0	106	7/19/2016	130.8	HEIGHT	22.45	15		0	0
8	2014	60	Placebo	0	150	2/13/2020	149.3	HEIGHT	31.55	17.5		0	0
8	2015	12	Placebo	0	-1	7/19/2016	61.7	LENGTH	6.2	13.5	42	1	0
8	2018	0	Placebo	0	54	3/25/2015	114.4	HEIGHT	17.8	14		0	0
8	2018	24	Placebo	0	77	3/27/2017	122.4	HEIGHT	20.6	14.5		0	0
8	2018	36	Placebo	0	92	5/24/2018	127.6	HEIGHT	22.81818182	15		0	0
8	2018	48	Placebo	0	103	4/16/2019	130.8	HEIGHT	26	16		0	0
8	2019	12	Placebo	1	6	7/19/2016	70.9	LENGTH	7.85	13.5		0	0
8	2021	24	Placebo	1	10	3/27/2017	71.1	LENGTH	9.4	15	42	1	0
8	2021	36	Placebo	1	21	5/24/2018	82.7	HEIGHT	12.25	16	42	1	0
8	2022	12	Placebo	1	42	7/19/2016	103.6	HEIGHT	13.8	13.5		1	0
8	2024	0	Placebo	0	24	3/25/2015	81.5	HEIGHT	10.9	15		1	0
8	2024	24	Placebo	0	35	3/27/2017	98.8	HEIGHT	14.35	15		0	0
8	2024	36	Placebo	0	171	5/24/2018	108.5	HEIGHT	16.68181818	16		0	0
8	2024	48	Placebo	0	182	4/16/2019	114.5	HEIGHT	18.4	16		0	0
8	2024	60	Placebo	0	191	2/13/2020	119.1	HEIGHT	20.9	16		0	0
8	2025	0	Placebo	0	48	3/25/2015	98	HEIGHT	14.5	15.5		0	0
8	2027	24	Placebo	0	26	3/27/2017	83.5	HEIGHT	10.75	14.5		1	0
8	2028	0	Placebo	1	24	3/25/2015	86.4	HEIGHT	11	14		1	0
8	2028	12	Placebo	1	54	7/19/2016	96.1	HEIGHT	12.85	14		0	0
8	2028	24	Placebo	1	65	3/27/2017	99.5	HEIGHT	13.45	13.5		0	0
8	2028	36	Placebo	1	80	5/24/2018	106.7	HEIGHT	14.13636364	14		0	0
8	2028	48	Placebo	1	91	4/16/2019	111	HEIGHT	16.65	14.5		0	0
8	2028	60	Placebo	1	100	2/13/2020	115.5	HEIGHT	18.45	15		0	0
8	2029	0	Placebo	0	24	3/25/2015	82.6	HEIGHT	9.05	12.5		1	0
8	2029	12	Placebo	0	54	7/19/2016	93.2	HEIGHT	13.2	14		0	0
8	2029	24	Placebo	0	40	3/27/2017	97.9	HEIGHT	15.45	14		1	0
8	2029	36	Placebo	0	55	5/24/2018	107.8	HEIGHT	16.65	14.5		0	0
8	2029	48	Placebo	0	65	4/16/2019	112.6	HEIGHT	17.8	14		0	0
8	2030	0	Placebo	0	48	6/11/2015	115.6	HEIGHT	19.9	16		0	0

8	2031	0	Placebo	1	12	3/25/2015	71.4	LENGTH	8.67	15	0	0
8	2031	12	Placebo	0	18	7/19/2016	80.9	HEIGHT	10.9	14.5	1	0
8	2031	24	Placebo	1	36	3/27/2017	83.7	HEIGHT	13.05	17	0	0
8	2031	36	Placebo	1	51	5/24/2018	93.2	HEIGHT	15	16.5	1	0
8	2031	48	Placebo	1	61	4/16/2019	98	HEIGHT	15.75	16.5	0	0
8	2031	60	Placebo	1	71	2/13/2020	104.3	HEIGHT	17.85	16.3	0	0
8	2032	0	Placebo	0	12	3/25/2015	72.7	HEIGHT	8.6	15	1	0
8	2032	12	Placebo	0	18	7/19/2016	84.9	HEIGHT	12.3	15	0	0
8	2032	24	Placebo	0	29	3/27/2017	88.4	HEIGHT	13	15.5	0	0
8	2032	36	Placebo	0	44	5/24/2018	98.3	HEIGHT	14.95	16.5	0	0
8	2032	48	Placebo	0	55	4/16/2019	101.7	HEIGHT	15.45	15	0	0
8	2032	60	Placebo	0	64	2/13/2020	106.5	HEIGHT	17.2	15	0	0
8	2033	0	Placebo	0	48	3/25/2015	89.8	HEIGHT	11.35	14	1	0
8	2033	12	Placebo	0	42	7/19/2016	100	HEIGHT	13.75	14	0	0
8	2033	24	Placebo	0	72	3/27/2017	103.9	HEIGHT	14.55	14.5	0	0
8	2033	36	Placebo	0	87	5/24/2018	110.9	HEIGHT	16.13636364	14	0	0
8	2033	48	Placebo	0	97	4/16/2019	115	HEIGHT	16.75	13.5	0	0
8	2033	60	Placebo	0	107	2/13/2020	119.5	HEIGHT	19.05	14.2	0	0
8	2040	12	Placebo	1	9	7/19/2016	71.4	LENGTH	9	15	0	0
8	2040	36	Placebo	0	29	5/24/2018	88.8	HEIGHT	13.1	17	1	0
8	2040	48	Placebo	0	45	4/16/2019	94.7	HEIGHT	14.35	15	1	0
8	2042	0	Placebo	1	48	3/25/2015	91.5	HEIGHT	13.3	15.5	0	0
8	2042	12	Placebo	1	42	7/19/2016	108.2	HEIGHT	18.55	16.5	0	0
8	2042	24	Placebo	0	53	3/27/2017	113.3	HEIGHT	21.8	18	0	0
8	2042	36	Placebo	0	68	5/24/2018	119.5	HEIGHT	22.85	17	0	0
8	2042	48	Placebo	0	79	4/16/2019	124.4	HEIGHT	26.05	17	0	0
8	2042	60	Placebo	0	88	2/13/2020	130.2	HEIGHT	29.9	18.5	0	0
8	2045	12	Placebo	1	11	7/19/2016	73.2	LENGTH	8.65	13.5	0	0
8	2045	24	Placebo	1	14	3/27/2017	81.6	HEIGHT	10	14	0	0
8	2045	36	Placebo	1	29	5/24/2018	89.6	HEIGHT	12.55	15.5	0	0
8	2045	48	Placebo	1	40	4/16/2019	96.1	HEIGHT	13.75	14	1	0
8	2045	60	Placebo	1	50	2/13/2020	102.8	HEIGHT	14.65	13.8	0	0
8	2048	0	Placebo	0	12	3/25/2015	76.5	HEIGHT	7.95	13	1	0
8	2048	12	Placebo	0	21	7/19/2016	84.8	HEIGHT	9.85	13.5	0	0
8	2049	0	Placebo	0	54	3/25/2015	107.8	HEIGHT	17.9	16	0	0
8	2049	12	Placebo	0	54	7/19/2016	117.2	HEIGHT	19.25	15	0	0
8	2049	24	Placebo	0	65	3/27/2017	120.2	HEIGHT	21.55	15.5	0	0
8	2049	36	Placebo	0	80	5/24/2018	125.6	HEIGHT	24.45454545	17	0	0
8	2049	48	Placebo	0	91	4/16/2019	130.4	HEIGHT	25.65	17	0	0
8	2049	60	Placebo	0	100	2/13/2020	133.6	HEIGHT	28.4	17.5	0	0

8	2050	0	Placebo	0	2	3/25/2015	62.8	LENGTH	7.4	15.5	1	0
8	2052	0	Placebo	0	24	6/11/2015	93.6	HEIGHT	14.2	16	0	0
8	2052	12	Placebo	0	37	7/19/2016	102.6	HEIGHT	15.4	14	0	0
8	2052	24	Placebo	0	53	3/27/2017	107.7	HEIGHT	15.6	14	0	0
8	2052	36	Placebo	0	68	5/24/2018	114.2	HEIGHT	18.40909091	15.5	0	0
8	2052	48	Placebo	0	79	4/16/2019	118.5	HEIGHT	18.55	14.5	0	0
8	2053	0	Placebo	1	48	3/25/2015	96.6	HEIGHT	13.4	14	1	0
8	2055	12	Placebo	1	4	7/19/2016	65.6	LENGTH	7.4	13.5	1	0
8	2055	24	Placebo	1	14	3/27/2017	75.5	HEIGHT	9	15	0	0
8	2055	36	Placebo	1	29	5/24/2018	86.7	HEIGHT	11.59090909	15	1	0
8	2055	48	Placebo	1	40	4/16/2019	93.2	HEIGHT	13.25	15.5	1	0
8	2055	60	Placebo	1	50	2/13/2020	99.5	HEIGHT	14.75	15.5	0	0
8	2057	0	Placebo	0	24	3/25/2015	82.2	HEIGHT	10.25	13.5	0	0
8	2057	12	Placebo	0	33	7/19/2016	90.3	HEIGHT	13	15	1	0
8	2057	24	Placebo	0	44	3/27/2017	94.9	HEIGHT	13.65	14	0	0
8	2057	36	Placebo	0	59	5/24/2018	101.7	HEIGHT	14.36363636	14	1	0
8	2057	48	Placebo	0	70	4/16/2019	106.4	HEIGHT	15.75	14	0	0
8	2057	60	Placebo	0	79	2/13/2020	110.8	HEIGHT	17.4	13.8	0	0
8	2059	12	Placebo	0	4	7/19/2016	71.4	LENGTH	9.1	13.5	0	0
8	2059	24	Placebo	0	14	3/27/2017	77.5	HEIGHT	11.45	15	1	0
8	2059	36	Placebo	0	29	5/24/2018	87.9	HEIGHT	12.85	15.5	0	0
8	2059	48	Placebo	0	40	4/16/2019	92.3	HEIGHT	14.15	14.5	1	0
8	2059	60	Placebo	0	50	2/13/2020	97.9	HEIGHT	16.35	15	1	0
8	2061	24	Placebo	1	53	3/27/2017	101.5	HEIGHT	15.3	14	1	0
8	2062	12	Placebo	1	4	7/19/2016	65.6	LENGTH	8.05	14.5	1	0
8	2062	24	Placebo	1	14	3/27/2017	74.3	HEIGHT	9	14	1	0
8	2062	36	Placebo	1	27	5/24/2018	84	HEIGHT	12.35	15.5	0	0
8	2062	48	Placebo	1	38	4/16/2019	90.8	HEIGHT	13.65	15.5	1	0
8	2062	60	Placebo	1	47	2/13/2020	96.1	HEIGHT	14.6	16.5	0	0
8	2063	12	Placebo	1	53	7/19/2016	95.7	HEIGHT	12.15	12.5	1	0
8	2064	0	Placebo	1	24	3/25/2015	83.5	HEIGHT	10.8	13.5	0	0
8	2064	12	Placebo	1	42	7/19/2016	96.8	HEIGHT	14.3	13	0	0
8	2064	24	Placebo	1	53	3/27/2017	100.5	HEIGHT	15.1	14.5	1	0
8	2065	24	Placebo	1	19	3/27/2017	82.7	HEIGHT	8.65	12.5	1	0
8	2067	12	Placebo	1	11	7/19/2016	72.6	LENGTH	7.75	12.5	0	0
8	2067	24	Placebo	1	20	3/27/2017	81.5	HEIGHT	10.2	13.5	1	0
8	2067	48	Placebo	1	46	4/16/2019	99.5	HEIGHT	13.45	14	1	0
8	2069	0	Placebo	1	24	3/25/2015	79.5	HEIGHT	9.25	14	1	0
8	2069	12	Placebo	1	38	7/19/2016	89.3	HEIGHT	11.65	14	1	0
8	2069	24	Placebo	1	48	3/27/2017	93.9	HEIGHT	12.8	14	1	0

8	2069	48	Placebo	1	73	4/16/2019	104.1	HEIGHT	15.3	14.5	0	0
8	2074	0	Placebo	1	12	3/25/2015	71.5	LENGTH	8.35	15	1	0
8	2074	12	Placebo	1	21	7/19/2016	84.6	HEIGHT	10.25	13.5	0	0
8	2074	24	Placebo	1	32	3/27/2017	87.5	HEIGHT	11.45	13.5	0	0
8	2074	36	Placebo	1	47	5/24/2018	95.7	HEIGHT	13.5	14	1	0
8	2074	60	Placebo	1	67	2/13/2020	108.4	HEIGHT	16.15	13.6	0	0
8	2078	0	Placebo	1	48	3/25/2015	108.5	HEIGHT	17.2	14.5	1	0
8	2078	12	Placebo	1	61	7/19/2016	116.8	HEIGHT	19.65	14.5	0	0
8	2079	12	Placebo	0	12	7/19/2016	76.2	LENGTH	9.15	13.5	1	0
8	2079	24	Placebo	0	23	3/27/2017	83.7	HEIGHT	10.85	14.5	0	0
8	2079	36	Placebo	0	38	5/24/2018	92.8	HEIGHT	14.04545455	16	0	0
8	2079	48	Placebo	0	48	4/16/2019	99.9	HEIGHT	15.8	16.5	0	0
8	2079	60	Placebo	0	58	2/13/2020	105.7	HEIGHT	17.7	16	0	0
8	8024	48	Placebo	0	5	4/16/2019	65.3	LENGTH	6.9	13.5	1	0
8	8024	60	Placebo	0	14	2/13/2020	76.4	LENGTH	9.4	14.1	1	0
8	8105	36	Placebo	0	12	5/24/2018	76.7	LENGTH	8.954545455	14.5	1	0
8	8105	48	Placebo	0	22	4/16/2019	87	LENGTH	10.25	14	0	0
8	8105	60	Placebo	0	32	2/13/2020	92.5	LENGTH	13.3	14	1	0
8	8136	36	Placebo	1	29	5/24/2018	85.7	LENGTH	11.18181818	14.5	1	0
8	8136	60	Placebo	1	50	2/13/2020	97.5	HEIGHT	15.75	16	1	0
8	8166	36	Placebo	0	16	5/24/2018	76.8	LENGTH	9.5	15	1	0
8	8166	48	Placebo	0	27	4/16/2019	84.3	HEIGHT	11.35	14	1	0
8	8172	60	Placebo	1	20	2/13/2020	82.6	HEIGHT	11.9	15.2	1	0
8	8210	60	Placebo	0	6	2/13/2020	66.4	HEIGHT	7.7	13.6	1	0
8	8237	48	Placebo	1	24	4/16/2019	81.5	HEIGHT	9	13.5	1	0
8	8283	48	Placebo	1	12	4/16/2019	75.2	HEIGHT	10	17	1	0
8	8356	36	Placebo	0	8	5/24/2018	69.5	LENGTH	7.5	13	1	0
8	8356	48	Placebo	0	19	4/16/2019	81.2	HEIGHT	10.05	14	1	0
8	8356	60	Placebo	0	28	2/13/2020	90.4	HEIGHT	12.55	14.5	0	0
8	8358	48	Placebo	1	12	4/16/2019	69.6	LENGTH	7.25	13.5	1	0
8	8402	60	Placebo	1	32	2/13/2020	88.6	HEIGHT	12.3	14.9	1	0
8	8411	36	Placebo	1	21	5/24/2018	82.4	LENGTH	9.045454545	14	1	0
8	8428	48	Placebo	0	12	4/16/2019	76.7	LENGTH	9.4	13.5	1	0
8	8435	60	Placebo	0	47	2/13/2020	97.2	HEIGHT	14.1	13.7	1	0
8	8468	60	Placebo	0	12	2/13/2020	75.4	LENGTH	8.55	12.5	1	0
8	8472	60	Placebo	0	41	2/13/2020	97.1	HEIGHT	13.95	14.5	1	0
8	8523	48	Placebo	1	35	4/16/2019	89.5	HEIGHT	12.2	15	1	0
8	8523	60	Placebo	1	44	2/13/2020	95.7	HEIGHT	13.55	14.5	1	0
8	8599	60	Placebo	0	20	2/13/2020	79.2	HEIGHT	9.8	14	1	0
8	8600	36	Placebo	0	5	5/24/2018	67.3	LENGTH	8	14.5	1	0

8	8600	48	Placebo	0	12	4/16/2019	76.3	LENGTH	9.6	14.5		1	0
8	8600	60	Placebo	0	21	2/13/2020	84.4	LENGTH	11.5	15		1	0
8	8601	48	Placebo	1	8	4/16/2019	66.9	LENGTH	8.5	16.5		1	0
8	8601	60	Placebo	1	14	2/13/2020	76.3	LENGTH	10.2	15		0	0
8	8612	48	Placebo	1	15	4/16/2019	75.8	LENGTH	9.95	14	60	1	0
8	8667	48	Placebo	1	2	4/16/2019	58.9	LENGTH	5.8	13		1	0
8	8667	60	Placebo	1	11	2/13/2020	72.8	LENGTH	8.6	13.5		1	0
8	8668	60	Placebo	0	32	2/13/2020	88.7	HEIGHT	11.7	13.3		1	0
8	8689	36	Placebo	0	164	5/24/2018	121.5	HEIGHT	21.18181818	16.5		0	0
8	8695	60	Placebo	1	26	2/18/2020	81.4	HEIGHT	10.3	13		1	0
8	8702	60	Placebo	0	18	2/13/2020	77.7	HEIGHT	9.45	14.7		1	0
8	8727	60	Placebo	1	57	2/13/2020	88.5	HEIGHT	10.85	13.2		1	0
8	8735	36	Placebo	1	14	5/24/2018	73.1	LENGTH	8.2	13.5		1	0
8	8735	48	Placebo	1	25	4/16/2019	85.1	HEIGHT	9.7	13		1	0
8	8735	60	Placebo	1	35	2/13/2020	90.6	HEIGHT	11.15	13.4		1	0
8	8742	36	Placebo	0	17	5/24/2018	76.7	LENGTH	8.55	13.5		1	0
8	8743	36	Placebo	0	19	5/24/2018	79.1	LENGTH	8.954545455	12.5		1	0
8	8743	48	Placebo	0	30	4/16/2019	85.8	HEIGHT	10.1	12.5		1	0
8	8743	60	Placebo	0	39	2/13/2020	91.8	HEIGHT	12.35	12.5		1	0
8	8766	48	Placebo	1	9	4/16/2019	65.3	LENGTH	6.7	14		1	0
8	8766	60	Placebo	1	14	2/13/2020	75.1	LENGTH	8.95	15		1	0
8	8776	48	Placebo	0	12	4/16/2019	76.8	LENGTH	9.05	14.5		1	0
8	8781	48	Placebo	1	14	4/16/2019	71.2	LENGTH	9.25	15		1	0
8	8786	36	Placebo	0	17	5/24/2018	78.5	LENGTH	9.25	14		1	0
8	8786	48	Placebo	0	28	4/16/2019	85.7	LENGTH	10.8	14.5		1	0
8	8786	60	Placebo	0	37	2/13/2020	90.2	HEIGHT	11.65	13.5		1	0
8	8830	36	Placebo	1	2	5/24/2018	57.6	LENGTH	6.25	13.5		1	0
8	8830	48	Placebo	1	13	4/16/2019	72.4	LENGTH	9.65	15		1	0
8	8830	60	Placebo	1	22	2/13/2020	80.7	LENGTH	11.05	15		0	0
8	8871	60	Placebo	1	55	2/13/2020	114.5	HEIGHT	19.3	15.5		1	0
8	8880	60	Placebo	0	14	2/13/2020	76.9	HEIGHT	9.5	14		1	0
8	8886	36	Placebo	0	12	5/24/2018	75.6	LENGTH	8.818181818	14		1	0
8	8886	48	Placebo	0	22	4/16/2019	83.7	HEIGHT	10.45	14		0	0
8	8886	60	Placebo	0	32	2/13/2020	90	HEIGHT	12.55	14.5		1	0
8	8896	48	Placebo	0	5	4/16/2019	66.4	LENGTH	7.7	14		1	0
8	8896	60	Placebo	0	14	2/13/2020	77.5	LENGTH	9.6	13.7		1	0
8	8948	36	Placebo	0	7	5/24/2018	65.4	LENGTH	7.55	14		1	0
8	9029	36	Placebo	1	44	5/24/2018	91.2	HEIGHT	12.35	15		1	0
8	9057	60	Placebo	1	10	2/13/2020	71.5	LENGTH	7.5	13		1	0
8	9109	36	Placebo	0	24	5/24/2018	84.5	LENGTH	12.15	16.5		1	0

8	9114	60	Placebo	1	3	2/18/2020	63.5	LENGTH	6.05	12	1	0
8	9158	60	Placebo	0	45	2/13/2020	99.1	HEIGHT	14.7	15	1	0
8	9206	36	Placebo	0	7	5/24/2018	64.2	LENGTH	6.55	13	1	0
8	9206	48	Placebo	0	17	4/16/2019	75.3	LENGTH	8.7	13.5	0	0
8	9206	60	Placebo	0	27	2/13/2020	83.8	LENGTH	10.35	13.5	0	0
8	9255	60	Placebo	0	45	2/13/2020	84	LENGTH	11.55	15	1	0
8	9259	60	Placebo	0	58	2/13/2020	112.2	HEIGHT	20.7	17	1	0
8	9280	48	Placebo	1	11	4/16/2019	70.4	LENGTH	8.05	13	1	0
8	9280	60	Placebo	1	20	2/13/2020	78.7	LENGTH	9.4	13.5	1	0
8	9348	60	Placebo	1	10	2/18/2020	69.8	LENGTH	8.75	14	1	0
8	9369	48	Placebo	0	11	4/16/2019	72.5	LENGTH	8.45	14	1	0
8	9369	60	Placebo	0	20	2/13/2020	82.4	HEIGHT	10.35	13.5	1	0
8	9381	60	Placebo	1	19	2/13/2020	77.6	LENGTH	10.25	14.3	1	0
8	9390	36	Placebo	0	56	5/24/2018	105.4	HEIGHT	15.3	14	1	0
8	9390	48	Placebo	0	56	4/16/2019	110.7	HEIGHT	17.4	14.5	1	0
8	9424	36	Placebo	0	12	5/24/2018	73.2	LENGTH	8.3	13	1	0
8	9424	48	Placebo	0	22	4/16/2019	82.5	HEIGHT	9.95	14.5	0	0
8	9424	60	Placebo	0	32	2/13/2020	88.8	HEIGHT	11.75	13.8	1	0
8	9426	48	Placebo	0	15	4/16/2019	63.8	LENGTH	8.1	15.5	1	0
8	9434	36	Placebo	1	39	5/24/2018	88.9	HEIGHT	11.40909091	14	1	0
8	9434	48	Placebo	1	49	4/16/2019	94.5	HEIGHT	12.65	14.5	1	0
8	9434	60	Placebo	1	59	2/13/2020	100.6	HEIGHT	13.8	15.3	1	0
8	9447	36	Placebo	0	26	5/24/2018	86	LENGTH	12.18181818	15	1	0
9	2092	0	Placebo	0	30	3/14/2015	88	HEIGHT	12.15	15	1	1
9	2092	12	Placebo	0	39	6/11/2016	97.5	HEIGHT	14.05	14.5	0	1
9	2092	24	Placebo	0	51	4/20/2017	103	HEIGHT	15	14	0	1
9	2092	36	Placebo	0	65	5/12/2018	109.5	HEIGHT	17.2	14	0	1
9	2092	48	Placebo	0	76	4/10/2019	114.5	HEIGHT	18.2	14.5	0	1
9	2092	60	Placebo	0	86	2/5/2020	120.3	HEIGHT	20.2	15.3	0	1
9	2093	24	Placebo	0	18	4/20/2017	77	HEIGHT	9.181818182	14.5	1	1
9	2096	24	Placebo	0	47	4/20/2017	98	HEIGHT	14.04545455	14	1	1
9	2097	12	Placebo	1	18	6/11/2016	76.9	LENGTH	8.65	13.5	0	1
9	2097	24	Placebo	1	29	4/20/2017	80.3	LENGTH	9.454545455	12.5	0	1
9	2097	36	Placebo	1	43	5/12/2018	87	HEIGHT	10.85	13	0	1
9	2097	48	Placebo	1	54	4/10/2019	94	HEIGHT	12.7	13.5	0	1
9	2098	12	Placebo	0	5	6/11/2016	61.5	LENGTH	6.05	12.5	0	1
9	2098	36	Placebo	0	28	5/12/2018	77	HEIGHT	9.7	12	0	1
9	2103	0	Placebo	0	48	3/14/2015	98.2	HEIGHT	13.65	15.6	1	1
9	2103	12	Placebo	0	48	6/11/2016	105.5	HEIGHT	14.35	14	0	1
9	2103	24	Placebo	0	72	4/20/2017	111.5	HEIGHT	15.18181818	15	0	1

9	2103	36	Placebo	0	86	5/12/2018	116	HEIGHT	17.65	15.5		0	1
9	2103	48	Placebo	0	97	4/10/2019	119.9	HEIGHT	18.35	14.1		0	1
9	2103	60	Placebo	0	107	2/5/2020	124.4	HEIGHT	20.9	16		0	1
9	2104	0	Placebo	1	12	3/14/2015	75.3	LENGTH	8.8	14.6		1	1
9	2104	12	Placebo	1	25	6/12/2016	80.5	HEIGHT	10.5	14.5		0	1
9	2104	24	Placebo	1	36	4/20/2017	88	HEIGHT	12.54545455	16		1	1
9	2104	36	Placebo	1	50	5/12/2018	94.5	HEIGHT	14.05	14.5		0	1
9	2104	60	Placebo	1	71	2/5/2020	109.4	HEIGHT	17.1	15.7		0	1
9	2105	0	Placebo	1	42	3/14/2015	87.5	HEIGHT	10.95	14.2		0	1
9	2105	24	Placebo	1	60	4/20/2017	101	HEIGHT	14.31818182	15		0	1
9	2105	36	Placebo	1	73	5/12/2018	107.5	HEIGHT	15.7	15.3		0	1
9	2105	48	Placebo	1	84	4/10/2019	112	HEIGHT	17.05	14.7		0	1
9	2105	60	Placebo	1	94	2/5/2020	117.1	HEIGHT	18.5	18		0	1
9	2106	12	Placebo	1	50	6/11/2016	100.3	HEIGHT	14.35	14		1	1
9	2107	0	Placebo	1	31	6/3/2015	71.1	LENGTH	8.25	14.5		0	1
9	2107	12	Placebo	1	30	6/12/2016	75.8	LENGTH	10.3	15.5		0	1
9	2107	36	Placebo	1	56	5/12/2018	95.2	HEIGHT	16.4	16.5		0	1
9	2107	48	Placebo	1	67	4/10/2019	102.1	HEIGHT	18.55	17		0	1
9	2107	60	Placebo	1	76	2/5/2020	108.2	HEIGHT	20.5	17.5		0	1
9	2109	0	Placebo	0	54	3/14/2015	107.1	HEIGHT	15.2	14.6		0	1
9	2109	12	Placebo	0	67	6/12/2016	116	HEIGHT	17.85	14.5		0	1
9	2109	24	Placebo	0	86	4/20/2017	121	HEIGHT	19.72727273	14.5		0	1
9	2109	36	Placebo	0	101	5/12/2018	126.4	HEIGHT	21.9	14.5		0	1
9	2109	48	Placebo	0	112	4/10/2019	132	HEIGHT	23.75	15.5		0	1
9	2109	60	Placebo	0	121	2/5/2020	135.3	HEIGHT	25.9	17		0	1
9	2110	0	Placebo	0	11	3/14/2015	69.5	LENGTH	6.8	13	18	1	1
9	2110	12	Placebo	0	24	6/11/2016	74.7	LENGTH	6.4	11.5	18	0	1
9	2111	12	Placebo	1	7	6/12/2016	66.3	LENGTH	6.65	13		0	1
9	2111	24	Placebo	1	14	4/20/2017	76	HEIGHT	8.227272727	13		0	1
9	2111	36	Placebo	1	32	5/12/2018	85.5	HEIGHT	10.6	14		0	1
9	2111	60	Placebo	1	53	2/5/2020	99.2	HEIGHT	13.3	14		1	1
9	2114	24	Placebo	0	14	4/20/2017	72.4	LENGTH	8.454545455	13.2		1	1
9	2114	36	Placebo	0	29	5/12/2018	82.9	HEIGHT	11.75	15		1	1
9	2117	0	Placebo	1	12	3/14/2015	72.3	LENGTH	8.95	17		0	1
9	2117	12	Placebo	1	24	6/11/2016	81.2	HEIGHT	12.4	16.5		0	1
9	2117	24	Placebo	1	35	4/20/2017	89.5	HEIGHT	13.68181818	17		0	1
9	2117	36	Placebo	1	50	5/12/2018	99.9	HEIGHT	15.75	15.5		0	1
9	2117	48	Placebo	1	61	4/10/2019	106.4	HEIGHT	17.9	16		0	1
9	2117	60	Placebo	1	70	2/5/2020	112	HEIGHT	19	15.4		0	1
9	2118	0	Placebo	1	30	3/14/2015	84.3	HEIGHT	10.65	15		0	1

9	2118	12	Placebo	1	33	6/11/2016	91.2	HEIGHT	12.5	15		0	1
9	2118	24	Placebo	1	56	4/20/2017	98	HEIGHT	13.45454545	14.5		0	1
9	2118	36	Placebo	1	71	5/12/2018	105	HEIGHT	15.7	14.5		0	1
9	2118	60	Placebo	1	92	2/5/2020	114.5	HEIGHT	18.1	15.4		0	1
9	2119	12	Placebo	1	24	6/12/2016	81	LENGTH	10.65	14	36	1	1
9	2123	0	Placebo	0	54	3/14/2015	102.3	HEIGHT	15.2	14.4		0	1
9	2123	24	Placebo	0	75	4/20/2017	114	HEIGHT	16.95454545	14		0	1
9	2123	36	Placebo	0	89	5/12/2018	118	HEIGHT	18.5	17.5		0	1
9	2123	48	Placebo	0	100	4/10/2019	122.5	HEIGHT	20.4	14.5		0	1
9	2123	60	Placebo	0	110	2/5/2020	126.9	HEIGHT	23.9	19		0	1
9	2129	0	Placebo	1	36	3/14/2015	94.5	HEIGHT	13.95	15.4		1	1
9	2129	12	Placebo	1	45	6/11/2016	102	HEIGHT	15.1	13.5		0	1
9	2129	36	Placebo	1	86	5/12/2018	112.1	HEIGHT	18.1	15		0	1
9	2129	48	Placebo	1	97	4/10/2019	117.5	HEIGHT	21.45	15.2		0	1
9	2130	12	Placebo	1	4	6/12/2016	65.5	LENGTH	7.6	15		0	1
9	2131	12	Placebo	1	42	6/11/2016	88.5	HEIGHT	12.8	15		1	1
9	2136	0	Placebo	1	22	3/14/2015	81.2	LENGTH	11.5	15.5		0	1
9	2136	12	Placebo	1	35	6/12/2016	90.9	HEIGHT	14.9	15.5		0	1
9	2136	24	Placebo	1	46	4/20/2017	99.5	HEIGHT	17.4	16.5		0	1
9	2136	36	Placebo	1	61	5/12/2018	105.8	HEIGHT	18.65	15.5		0	1
9	2136	48	Placebo	1	71	4/10/2019	113	HEIGHT	20.45	16.5		0	1
9	2137	0	Placebo	0	43	3/14/2015	97	HEIGHT	14.25	15.9		1	1
9	2137	12	Placebo	0	54	6/11/2016	105	HEIGHT	15.25	14.5		1	1
9	2137	24	Placebo	0	65	4/20/2017	109	HEIGHT	16.90909091	15.5		0	1
9	2137	36	Placebo	0	84	5/12/2018	114	HEIGHT	18.45	14.9		0	1
9	2137	48	Placebo	0	95	4/10/2019	119	HEIGHT	19.75	14.8		0	1
9	2137	60	Placebo	0	105	2/5/2020	123.1	HEIGHT	21.8	17.4		0	1
9	2143	12	Placebo	1	42	6/11/2016	89.6	HEIGHT	12.4	15		1	1
9	2143	36	Placebo	1	56	5/12/2018	106	HEIGHT	14.5	14		1	1
9	2144	0	Placebo	1	48	3/14/2015	106.6	HEIGHT	16.5	17.1		1	1
9	2144	12	Placebo	1	57	6/11/2016	111.5	HEIGHT	17.1	15		0	1
9	2144	24	Placebo	1	69	4/20/2017	116	HEIGHT	18.09090909	15		0	1
9	2144	36	Placebo	1	83	5/12/2018	119	HEIGHT	20.6	15.7		0	1
9	2144	48	Placebo	1	94	4/10/2019	124.1	HEIGHT	21.05	16		0	1
9	2144	60	Placebo	1	104	2/5/2020	127.4	HEIGHT	22.8	17		0	1
9	2145	24	Placebo	1	33	4/20/2017	78.5	HEIGHT	9.090909091	13		1	1
9	2145	48	Placebo	1	58	4/10/2019	94	HEIGHT	12.25	13.5		1	1
9	2151	12	Placebo	0	45	6/12/2016	117.1	HEIGHT	20.05	15.5		1	1
9	2151	24	Placebo	0	67	4/20/2017	122.3	HEIGHT	21.72727273	16.5		0	1
9	2151	36	Placebo	0	82	5/12/2018	126	HEIGHT	23.85	18.5		0	1

9	2151	48	Placebo	0	93	4/10/2019	131.5	HEIGHT	26	17.7		0	1
9	2151	60	Placebo	0	102	2/5/2020	135.1	HEIGHT	28.1	19		0	1
9	2152	12	Placebo	0	42	6/11/2016	98	HEIGHT	13.9	14		1	1
9	2153	0	Placebo	0	36	3/14/2015	85.2	LENGTH	11.1	15.2	6	0	1
9	2154	0	Placebo	1	49	3/14/2015	103.8	HEIGHT	16.6	16.3		1	1
9	2154	12	Placebo	1	54	6/11/2016	109	HEIGHT	18.55	15		0	1
9	2154	24	Placebo	1	67	4/20/2017	114	HEIGHT	19.81818182	16		0	1
9	2154	36	Placebo	1	81	5/12/2018	118.5	HEIGHT	22.3	16		0	1
9	2154	48	Placebo	1	92	4/10/2019	123.4	HEIGHT	23.7	16.5		0	1
9	2156	0	Placebo	0	48	3/14/2015	96.1	HEIGHT	13.65	15.6		1	1
9	2156	12	Placebo	0	57	6/12/2016	103.2	HEIGHT	15.55	15.5		1	1
9	2156	24	Placebo	0	69	4/20/2017	111.5	HEIGHT	17.5	15		0	1
9	2156	36	Placebo	0	83	5/12/2018	117.4	HEIGHT	19.15	14.5		0	1
9	2156	48	Placebo	0	94	4/10/2019	122.3	HEIGHT	21.4	15.5		0	1
9	2157	24	Placebo	1	9	4/20/2017	70.4	LENGTH	8.363636364	15.5		1	1
9	2157	36	Placebo	1	23	5/12/2018	80.7	LENGTH	10	14.7		0	1
9	2157	48	Placebo	1	34	4/10/2019	86.1	HEIGHT	10.95	14		0	1
9	2157	60	Placebo	1	44	2/5/2020	93	HEIGHT	13.3	15.5		0	1
9	2159	0	Placebo	0	7	3/14/2015	63.5	LENGTH	6.2	12.6		1	1
9	2159	12	Placebo	0	26	6/11/2016	73.5	LENGTH	8.3	12		0	1
9	2159	36	Placebo	0	46	5/12/2018	87	HEIGHT	12.1	14.1		0	1
9	2161	12	Placebo	1	15	6/11/2016	82.3	HEIGHT	9.85	13.5		1	1
9	2161	48	Placebo	1	55	4/10/2019	103	HEIGHT	14.95	13.2		1	1
9	2162	0	Placebo	0	54	3/14/2015	117.9	HEIGHT	19.35	17.2		0	1
9	2162	24	Placebo	0	96	4/20/2017	128.5	HEIGHT	22.81818182	17		0	1
9	2162	36	Placebo	0	110	5/12/2018	135	HEIGHT	25.7	17.5		0	1
9	2162	48	Placebo	0	121	4/10/2019	138	HEIGHT	28	18		0	1
9	2162	60	Placebo	0	131	2/5/2020	140.1	HEIGHT	30	19		0	1
9	2165	24	Placebo	0	13	4/20/2017	74	HEIGHT	7.409090909	13.5		1	1
9	2166	0	Placebo	0	48	3/14/2015	100.3	HEIGHT	14.8	14.4		0	1
9	2166	12	Placebo	0	66	6/11/2016	105.1	HEIGHT	17.2	14.5		0	1
9	2166	24	Placebo	0	71	4/20/2017	110.1	HEIGHT	17.95454545	15		0	1
9	2166	36	Placebo	0	85	5/12/2018	117	HEIGHT	20.75	15.4		0	1
9	2166	48	Placebo	0	96	4/10/2019	122.3	HEIGHT	22.85	15.5		0	1
9	2166	60	Placebo	0	106	2/5/2020	124.8	HEIGHT	23.7	16.4		0	1
9	2168	0	Placebo	0	48	3/14/2015	97	HEIGHT	14.25	15.4		1	1
9	2169	0	Placebo	0	24	3/14/2015	78.3	HEIGHT	11.05	15.5		0	1
9	2169	12	Placebo	0	30	6/11/2016	86.9	HEIGHT	13.1	15		0	1
9	2169	24	Placebo	0	41	4/20/2017	94.5	HEIGHT	14.5	15.5		0	1
9	2169	36	Placebo	0	55	5/12/2018	102	HEIGHT	15.95	14		1	1

9	2169	48	Placebo	0	66	4/10/2019	109.4	HEIGHT	17.4	14.5	0	1
9	2169	60	Placebo	0	76	2/5/2020	114.8	HEIGHT	19.8	15	0	1
9	2172	12	Placebo	1	42	6/12/2016	104	HEIGHT	16.9	15.5	1	1
9	2173	24	Placebo	0	32	4/20/2017	85.8	HEIGHT	11.38636364	14.4	1	1
9	2174	0	Placebo	0	48	3/14/2015	104.6	HEIGHT	16.6	15	0	1
9	2174	12	Placebo	0	66	6/11/2016	112.3	HEIGHT	18.2	15	0	1
9	2174	24	Placebo	0	77	4/20/2017	115.8	LENGTH	18.59090909	15	0	1
9	2174	36	Placebo	0	92	5/12/2018	123	HEIGHT	20.8	15	0	1
9	2174	48	Placebo	0	103	4/10/2019	124.5	HEIGHT	22.85	15.5	0	1
9	2174	60	Placebo	0	112	2/5/2020	129.9	HEIGHT	26.2	17	0	1
9	2177	0	Placebo	1	10	3/14/2015	60.7	LENGTH	5.95	13.5	0	1
9	2177	12	Placebo	1	18	6/11/2016	75.8	LENGTH	8.65	14	1	1
9	2177	24	Placebo	1	29	4/20/2017	82.1	HEIGHT	10.54545455	13.5	0	1
9	2177	36	Placebo	1	43	5/12/2018	92.6	HEIGHT	12.65	13	0	1
9	2177	48	Placebo	1	54	4/10/2019	98.4	HEIGHT	14.3	13.5	1	1
9	2177	60	Placebo	1	64	2/5/2020	104.5	HEIGHT	15.2	13.8	0	1
9	2178	12	Placebo	1	43	6/11/2016	93	HEIGHT	12.55	14.5	1	1
9	2178	24	Placebo	1	54	4/20/2017	98.3	HEIGHT	14.72727273	14.5	1	1
9	2179	0	Placebo	1	4	3/14/2015	60.6	LENGTH	6.2	13.5	1	1
9	2179	12	Placebo	1	12	6/11/2016	72.3	LENGTH	7.9	12.5	0	1
9	2179	24	Placebo	1	23	4/20/2017	78.4	LENGTH	8.681818182	13	1	1
9	2179	36	Placebo	1	42	5/12/2018	85	HEIGHT	11.05	13.1	0	1
9	2179	48	Placebo	1	53	4/10/2019	93	HEIGHT	13.25	14	1	1
9	2179	60	Placebo	1	63	2/5/2020	102	HEIGHT	14.7	14.9	0	1
9	2180	0	Placebo	1	30	3/14/2015	94	HEIGHT	11.85	14.5	1	1
9	2180	12	Placebo	1	39	6/11/2016	102	HEIGHT	13.95	14	0	1
9	2180	24	Placebo	1	67	4/20/2017	109	HEIGHT	15.18181818	14	0	1
9	2180	36	Placebo	1	81	5/12/2018	115.4	HEIGHT	17.05	14	0	1
9	2180	48	Placebo	1	92	4/10/2019	120	HEIGHT	19.45	15	0	1
9	2180	60	Placebo	1	102	2/5/2020	124.6	HEIGHT	20.1	14.5	0	1
9	2181	12	Placebo	0	6	6/11/2016	61.3	LENGTH	4.85	11	0	1
9	2181	24	Placebo	0	14	4/20/2017	69	HEIGHT	7.090909091	13.5	0	1
9	2181	36	Placebo	0	28	5/12/2018	75	HEIGHT	9.35	14.5	0	1
9	2182	24	Placebo	1	9	4/20/2017	67.3	LENGTH	8.136363636	15	1	1
9	2182	36	Placebo	1	23	5/12/2018	76	HEIGHT	9.6	14	0	1
9	2182	48	Placebo	1	34	4/10/2019	82.7	HEIGHT	11.45	14.5	0	1
9	2183	0	Placebo	0	36	3/14/2015	81.3	HEIGHT	9.3	12.7	0	1
9	2183	12	Placebo	0	36	6/11/2016	88.4	HEIGHT	14.2	15.5	0	1
9	2183	24	Placebo	0	47	4/20/2017	94.5	HEIGHT	13.90909091	14.5	0	1
9	2183	36	Placebo	0	61	5/12/2018	98.9	HEIGHT	13.8	13.5	0	1

9	2183	48	Placebo	0	72	4/10/2019	101.8	HEIGHT	15.65	13.5		0	1
9	2184	12	Placebo	0	66	6/11/2016	106	HEIGHT	15.35	15		0	1
9	2184	24	Placebo	0	77	4/20/2017	110	HEIGHT	16	14.5		0	1
9	2184	36	Placebo	0	92	5/12/2018	114.5	HEIGHT	17.35	15		0	1
9	2184	60	Placebo	0	112	2/5/2020	122.7	HEIGHT	20.7	15.5		0	1
9	2187	0	Placebo	0	12	3/14/2015	79.2	LENGTH	9.4	16.2		0	1
9	2188	0	Placebo	1	36	3/14/2015	91.5	HEIGHT	14.5	17.1		1	1
9	2188	12	Placebo	1	41	6/11/2016	99.1	LENGTH	17.25	16.5		1	1
9	2188	24	Placebo	1	51	4/20/2017	106.4	HEIGHT	18	16.5		0	1
9	2188	36	Placebo	1	65	5/12/2018	112	HEIGHT	19.9	16		0	1
9	2188	48	Placebo	1	76	4/10/2019	117	HEIGHT	21.4	16.5		0	1
9	2188	60	Placebo	1	86	2/5/2020	122.3	HEIGHT	23.3	17.5		0	1
9	2190	12	Placebo	1	54	6/12/2016	99.2	HEIGHT	14.4	14		1	1
9	2192	0	Placebo	1	20	3/14/2015	75.1	LENGTH	8.55	14	18	0	1
9	2193	0	Placebo	1	36	3/14/2015	75.6	LENGTH	8.5	13.4		0	1
9	2193	24	Placebo	1	57	4/20/2017	94	HEIGHT	13.72727273	15.5		1	1
9	2193	36	Placebo	1	71	5/12/2018	100.1	HEIGHT	14.15	15		0	1
9	2193	48	Placebo	1	82	4/10/2019	105.9	HEIGHT	16.7	14.8		0	1
9	2194	0	Placebo	0	30	3/14/2015	73.7	LENGTH	8.05	12.6		1	1
9	2194	12	Placebo	0	36	6/11/2016	84.4	HEIGHT	10.7	13.5		0	1
9	2194	24	Placebo	1	47	4/20/2017	91	HEIGHT	12.22727273	14.5		0	1
9	2194	36	Placebo	0	62	5/12/2018	99	HEIGHT	13.6	14		0	1
9	2194	48	Placebo	0	72	4/10/2019	103	HEIGHT	14.6	13.9		0	1
9	2195	0	Placebo	0	42	3/14/2015	95.3	HEIGHT	14.2	14.9		0	1
9	2195	12	Placebo	0	51	6/12/2016	103	HEIGHT	16.15	14		1	1
9	2195	24	Placebo	0	63	4/20/2017	109	HEIGHT	17.86363636	15		0	1
9	2195	36	Placebo	0	77	5/12/2018	114.5	HEIGHT	19.95	14.5		0	1
9	2195	48	Placebo	0	88	4/10/2019	118.4	HEIGHT	20.65	15.2		0	1
9	2195	60	Placebo	0	98	2/5/2020	121.4	HEIGHT	23.3	15		0	1
9	2196	24	Placebo	0	50	4/20/2017	100.8	HEIGHT	13.31818182	13.5		1	1
9	2197	0	Placebo	1	23	3/14/2015	85.1	LENGTH	12.55	17.1		0	1
9	2197	12	Placebo	1	36	6/12/2016	93.3	HEIGHT	15.4	17		0	1
9	2197	24	Placebo	1	47	4/20/2017	101	HEIGHT	17.90909091	17.5		0	1
9	2197	36	Placebo	1	62	5/12/2018	107.8	HEIGHT	19.15	16		0	1
9	2197	48	Placebo	1	72	4/10/2019	113.5	HEIGHT	20.85	16		0	1
9	2197	60	Placebo	1	82	2/5/2020	118.6	HEIGHT	23.1	18		0	1
9	2199	24	Placebo	0	31	4/20/2017	87	HEIGHT	12.18181818	14		1	1
9	2199	48	Placebo	0	55	4/10/2019	102.5	HEIGHT	16.45	14.6		1	1
9	2201	12	Placebo	1	8	6/11/2016	63.5	LENGTH	5.65	11.5		0	1
9	2201	24	Placebo	1	15	4/20/2017	74	HEIGHT	8	14		0	1

9	2201	36	Placebo	1	30	5/12/2018	80.3	HEIGHT	9.85	13	0	1
9	2201	60	Placebo	1	50	2/5/2020	95	HEIGHT	12.5	13.9	0	1
9	2203	0	Placebo	1	23	3/14/2015	79.7	HEIGHT	8.9	13.9	0	1
9	2203	12	Placebo	1	42	6/11/2016	86.5	HEIGHT	10.75	13.5	0	1
9	2203	24	Placebo	1	167	4/20/2017	92.4	HEIGHT	12.04545455	14.5	0	1
9	2203	36	Placebo	1	61	5/12/2018	99	HEIGHT	13.75	14.5	0	1
9	2205	12	Placebo	1	56	6/11/2016	97	HEIGHT	13.05	13.5	1	1
9	2206	0	Placebo	1	24	3/14/2015	78.6	HEIGHT	9.75	14.3	1	1
9	2206	12	Placebo	1	30	6/11/2016	90.5	HEIGHT	13.4	15.5	0	1
9	2206	24	Placebo	1	41	4/20/2017	98	HEIGHT	15.77272727	16	1	1
9	2206	36	Placebo	1	62	5/12/2018	106.4	HEIGHT	18.45	16	0	1
9	2206	48	Placebo	1	73	4/10/2019	113	HEIGHT	19.9	16.5	0	1
9	2210	0	Placebo	1	34	3/14/2015	82.7	HEIGHT	11.05	14.9	1	1
9	2210	12	Placebo	1	47	6/11/2016	90	HEIGHT	12.2	15	0	1
9	2210	24	Placebo	1	58	4/20/2017	97	HEIGHT	13.59090909	14.5	0	1
9	2210	36	Placebo	1	73	5/12/2018	102.5	HEIGHT	14.8	14	0	1
9	2210	48	Placebo	1	84	4/10/2019	107.9	HEIGHT	16.05	14.1	0	1
9	2210	60	Placebo	1	94	2/5/2020	112.2	HEIGHT	18.2	15	0	1
9	2212	24	Placebo	0	54	4/20/2017	107.5	HEIGHT	16.18181818	14.5	1	1
9	2213	24	Placebo	0	2	4/20/2017	66.3	LENGTH	7.227272727	16.5	1	1
9	2213	36	Placebo	0	16	5/12/2018	78	HEIGHT	10.25	16	0	1
9	2213	48	Placebo	0	27	4/10/2019	85.1	HEIGHT	11.45	14	0	1
9	2213	60	Placebo	0	37	2/5/2020	88.8	HEIGHT	13.7	16.5	1	1
9	2214	0	Placebo	1	42	3/14/2015	85.5	LENGTH	11.15	15.5	1	1
9	2214	12	Placebo	1	42	6/12/2016	94.3	HEIGHT	12.65	14.5	1	1
9	2214	24	Placebo	1	53	4/20/2017	100	HEIGHT	13.77272727	14.5	0	1
9	2214	36	Placebo	1	68	5/12/2018	106.5	HEIGHT	15.1	14.5	0	1
9	2214	48	Placebo	1	79	4/10/2019	111	HEIGHT	16.45	14.8	0	1
9	2214	60	Placebo	1	88	2/5/2020	116.2	HEIGHT	17.9	15	0	1
9	2215	12	Placebo	0	48	6/11/2016	94	HEIGHT	14.8	15.5	1	1
9	2216	12	Placebo	1	31	6/12/2016	87.7	HEIGHT	12.95	15.5	1	1
9	2216	36	Placebo	0	56	5/12/2018	103	HEIGHT	16.35	15.2	1	1
9	2217	0	Placebo	1	24	3/14/2015	79.9	LENGTH	9.6	13.5	0	1
9	2217	12	Placebo	1	37	6/11/2016	88.5	HEIGHT	12.45	14.5	0	1
9	2217	24	Placebo	1	54	4/20/2017	95.6	HEIGHT	13.09090909	14	1	1
9	2217	36	Placebo	1	74	5/12/2018	102	HEIGHT	14.95	13.5	0	1
9	2217	48	Placebo	1	85	4/10/2019	108.2	HEIGHT	16.65	14	0	1
9	2217	60	Placebo	1	95	2/5/2020	102.2	HEIGHT	17.6	15	0	1
9	2223	0	Placebo	0	12	3/14/2015	65	LENGTH	7.5	15.2	0	1
9	2223	12	Placebo	0	12	6/11/2016	77.3	LENGTH	9.65	14.5	0	1

9	2223	24	Placebo	0	30	4/20/2017	84	HEIGHT	11.22727273	16.5	0	1
9	2223	36	Placebo	0	44	5/12/2018	91.5	HEIGHT	12.15	15	0	1
9	2223	48	Placebo	0	55	4/10/2019	97.5	HEIGHT	13.85	14.4	0	1
9	2223	60	Placebo	0	65	2/5/2020	102	HEIGHT	14.5	14.8	0	1
9	2225	12	Placebo	0	10	6/12/2016	73.4	LENGTH	9.2	14.5	1	1
9	2225	24	Placebo	0	21	4/20/2017	82	HEIGHT	10.54545455	15	0	1
9	2225	36	Placebo	0	36	5/12/2018	89.5	HEIGHT	13.65	15.7	0	1
9	2225	48	Placebo	0	47	4/10/2019	96.9	HEIGHT	15.25	16	0	1
9	2225	60	Placebo	0	56	2/5/2020	102.5	HEIGHT	16.9	16.7	0	1
9	2226	12	Placebo	1	3	6/11/2016	66	HEIGHT	6.35	12	0	1
9	2226	24	Placebo	1	15	4/20/2017	78.2	LENGTH	9.181818182	13.5	0	1
9	2226	36	Placebo	1	29	5/12/2018	86	HEIGHT	10.55	13.5	1	1
9	2226	48	Placebo	1	40	4/10/2019	93.3	HEIGHT	12	13	1	1
9	2226	60	Placebo	1	50	2/5/2020	99.9	HEIGHT	14.6	15	0	1
9	2229	0	Placebo	0	31	3/14/2015	88.2	HEIGHT	13.2	16.8	1	1
9	2229	12	Placebo	0	36	6/12/2016	99.4	HEIGHT	16.75	17	0	1
9	2229	24	Placebo	0	47	4/20/2017	106	HEIGHT	19.18181818	17.5	0	1
9	2229	36	Placebo	0	65	5/12/2018	113	HEIGHT	20.15	17	0	1
9	2229	48	Placebo	0	76	4/10/2019	119.5	HEIGHT	22.65	17.1	0	1
9	2229	60	Placebo	0	85	2/5/2020	124	HEIGHT	25.6	19	0	1
9	2231	0	Placebo	1	54	3/14/2015	101.3	HEIGHT	17.4	17.8	0	1
9	2231	12	Placebo	1	63	6/11/2016	109.9	HEIGHT	19.8	16	0	1
9	2231	24	Placebo	1	75	4/20/2017	115	HEIGHT	21.09090909	17.5	0	1
9	2231	36	Placebo	1	89	5/12/2018	119.5	HEIGHT	24	17	0	1
9	2231	48	Placebo	1	100	4/10/2019	123	HEIGHT	26.1	17.7	0	1
9	2231	60	Placebo	1	110	2/5/2020	127.3	HEIGHT	26.5	18	0	1
9	2237	0	Placebo	0	11	3/14/2015	69.1	LENGTH	8.2	14.2	0	1
9	2237	12	Placebo	0	18	6/12/2016	80.3	LENGTH	11.1	15	0	1
9	2237	24	Placebo	0	31	4/20/2017	88	HEIGHT	13.5	15	0	1
9	2237	36	Placebo	0	45	5/12/2018	97	HEIGHT	15.25	14.2	0	1
9	2237	48	Placebo	0	56	4/10/2019	104	HEIGHT	17.3	14.8	0	1
9	2237	60	Placebo	0	66	2/5/2020	109.2	HEIGHT	19.5	15	0	1
9	2238	0	Placebo	1	30	3/14/2015	87.8	HEIGHT	12.25	16	0	1
9	2238	12	Placebo	1	42	6/11/2016	93	HEIGHT	14.6	15.5	0	1
9	2238	36	Placebo	1	86	5/12/2018	107	HEIGHT	17.35	14.7	0	1
9	2238	48	Placebo	1	97	4/10/2019	112	HEIGHT	18.55	15	0	1
9	2239	12	Placebo	0	34	6/12/2016	85	HEIGHT	12.4	16	1	1
9	2240	24	Placebo	1	34	4/20/2017	87	HEIGHT	12.81818182	15.5	1	1
9	2241	0	Placebo	1	18	3/14/2015	67.7	LENGTH	8.95	17	1	1
9	2241	12	Placebo	1	23	6/11/2016	74.5	LENGTH	10.05	14	0	1

9	2241	24	Placebo	1	33	4/20/2017	83	HEIGHT	12.31818182	17.5	0	1
9	2241	36	Placebo	1	47	5/12/2018	90.5	HEIGHT	14.1	15.5	0	1
9	2241	48	Placebo	1	58	4/10/2019	96.9	HEIGHT	14.5	15.1	1	1
9	2241	60	Placebo	1	68	2/5/2020	103.3	HEIGHT	16.9	16.4	0	1
9	2242	0	Placebo	0	48	3/14/2015	102.4	HEIGHT	14.7	14.7	1	1
9	2242	12	Placebo	0	57	6/11/2016	109	HEIGHT	16.6	14.5	0	1
9	2242	24	Placebo	0	69	4/20/2017	113.5	HEIGHT	17.68181818	14.8	0	1
9	2242	36	Placebo	0	83	5/12/2018	118.5	HEIGHT	18.9	14.8	0	1
9	2242	48	Placebo	0	94	4/10/2019	122.2	HEIGHT	19.65	14.5	0	1
9	2245	0	Placebo	0	9	3/14/2015	72.6	LENGTH	8.75	15.4	1	1
9	2245	12	Placebo	0	21	6/11/2016	83	HEIGHT	10.55	14	0	1
9	2245	24	Placebo	0	33	4/20/2017	87	HEIGHT	12.09090909	15	0	1
9	2245	36	Placebo	0	46	5/12/2018	92.5	HEIGHT	13.95	14.5	0	1
9	2245	48	Placebo	0	57	4/10/2019	97.9	HEIGHT	15.75	15	0	1
9	2245	60	Placebo	0	67	2/5/2020	103.5	HEIGHT	17	16	0	1
9	2247	0	Placebo	0	48	3/14/2015	90.2	HEIGHT	10.7	13.2	1	1
9	2247	12	Placebo	0	63	6/11/2016	96	HEIGHT	12.7	13	0	1
9	2248	0	Placebo	0	26	3/14/2015	80.3	HEIGHT	10.35	14.3	0	1
9	2248	12	Placebo	0	39	6/11/2016	90.5	HEIGHT	12.9	15	0	1
9	2248	24	Placebo	0	50	4/20/2017	95.9	LENGTH	14.5	14	0	1
9	2248	36	Placebo	0	65	5/12/2018	105.5	HEIGHT	15.5	14.1	0	1
9	2248	48	Placebo	0	76	4/10/2019	111.3	HEIGHT	17.4	14.5	0	1
9	2248	60	Placebo	0	86	2/5/2020	116.8	HEIGHT	19.1	15	0	1
9	2249	12	Placebo	0	11	6/12/2016	72.3	LENGTH	8.05	13.5	0	1
9	2249	36	Placebo	0	35	5/12/2018	91	HEIGHT	12	12	0	1
9	2249	48	Placebo	0	46	4/10/2019	96.1	HEIGHT	13.85	14	1	1
9	2251	0	Placebo	1	36	3/14/2015	92.3	HEIGHT	12.45	14.9	0	1
9	2251	12	Placebo	1	45	6/11/2016	100.2	HEIGHT	15.15	14.3	0	1
9	2251	24	Placebo	1	57	4/20/2017	105.1	HEIGHT	16	14.5	0	1
9	2251	36	Placebo	1	86	5/12/2018	110.1	HEIGHT	17.2	14.5	0	1
9	2251	48	Placebo	1	97	4/10/2019	114.5	HEIGHT	18.66	15	0	1
9	2252	24	Placebo	0	53	4/20/2017	94	HEIGHT	13.81818182	15	1	1
9	2253	12	Placebo	0	54	6/11/2016	94.6	HEIGHT	14.35	14.5	1	1
9	2255	0	Placebo	1	34	3/14/2015	83.5	HEIGHT	10.1	13.7	0	1
9	2255	12	Placebo	1	47	6/11/2016	92.9	HEIGHT	12.45	15	0	1
9	2255	24	Placebo	1	58	4/20/2017	98.5	HEIGHT	13.45454545	14.5	0	1
9	2255	36	Placebo	1	73	5/12/2018	103	HEIGHT	14.35	13.5	0	1
9	2255	48	Placebo	1	84	4/10/2019	108.4	HEIGHT	16.05	14.3	0	1
9	2255	60	Placebo	1	94	2/5/2020	115.3	HEIGHT	17.7	14.2	0	1
9	2256	24	Placebo	0	2	4/20/2017	66.3	LENGTH	7.045454545	14	1	1

9	2256	36	Placebo	0	14	5/12/2018	80.5	HEIGHT	9.5	12.5		1	1
9	2256	48	Placebo	0	25	4/10/2019	90	HEIGHT	13.05	14		0	1
9	2256	60	Placebo	0	35	2/5/2020	95.6	HEIGHT	13.5	13.7		0	1
9	2257	24	Placebo	0	14	4/20/2017	82.1	HEIGHT	10	15		1	1
9	2257	48	Placebo	0	39	4/10/2019	95.9	HEIGHT	14.1	15.3		1	1
9	2258	12	Placebo	1	54	6/11/2016	104	HEIGHT	15.65	14.5		1	1
9	2262	24	Placebo	0	7	4/20/2017	70.8	LENGTH	8	14.5		1	1
9	2262	36	Placebo	0	21	5/12/2018	80.5	HEIGHT	10.9	13.6		0	1
9	2264	24	Placebo	0	7	4/20/2017	70.3	LENGTH	7.681818182	13.5		1	1
9	2264	36	Placebo	0	21	5/12/2018	76.1	HEIGHT	8.95	13.8		1	1
9	2264	48	Placebo	0	32	4/10/2019	82	HEIGHT	10.35	13		0	1
9	2264	60	Placebo	0	42	2/5/2020	86.2	HEIGHT	11.9	14		0	1
9	2267	24	Placebo	1	9	4/20/2017	71.5	LENGTH	8.363636364	14.5	30	1	1
9	2269	0	Placebo	1	21	3/14/2015	79.7	LENGTH	10.7	16		0	1
9	2269	12	Placebo	1	42	6/11/2016	87.3	HEIGHT	12.2	14.5		0	1
9	2269	24	Placebo	1	53	4/20/2017	94	HEIGHT	14.31818182	15		0	1
9	2272	24	Placebo	1	50	4/20/2017	100.5	HEIGHT	15.40909091	16		1	1
9	2274	0	Placebo	1	54	3/14/2015	101.5	HEIGHT	13.55	15.2		0	1
9	2274	12	Placebo	1	60	6/11/2016	107.5	HEIGHT	15.4	14		0	1
9	2274	24	Placebo	1	71	4/20/2017	113.6	HEIGHT	16.59090909	14.5		0	1
9	2274	36	Placebo	1	96	5/12/2018	117.5	HEIGHT	18.05	14.5		0	1
9	2274	48	Placebo	1	106	4/10/2019	121	HEIGHT	19.35	14.5		0	1
9	2274	60	Placebo	1	116	2/5/2020	124.4	HEIGHT	20.8	15.3		0	1
9	2275	12	Placebo	0	85	6/11/2016	116.2	HEIGHT	18.35	16		1	1
9	2278	12	Placebo	0	44	6/11/2016	105.4	HEIGHT	15.7	15		1	1
9	2278	24	Placebo	0	55	4/20/2017	106	HEIGHT	16.04545455	15		1	1
9	2279	0	Placebo	0	12	3/14/2015	69.3	LENGTH	7.9	13.5	6	0	1
9	2281	0	Placebo	1	23	3/14/2015	77.3	LENGTH	9.22	14.7		0	1
9	2281	12	Placebo	1	31	6/11/2016	85.1	HEIGHT	11.55	14.5		0	1
9	2281	24	Placebo	1	42	4/20/2017	93	HEIGHT	13.31818182	14.5		0	1
9	2281	36	Placebo	1	57	5/12/2018	100	HEIGHT	14.7	14.5		0	1
9	2281	48	Placebo	1	68	4/10/2019	105	HEIGHT	15.65	14.5		0	1
9	2281	60	Placebo	1	77	2/5/2020	110.1	HEIGHT	17.3	14.5		0	1
9	2284	0	Placebo	0	6	3/14/2015	66.5	LENGTH	7.65	14.4	30	1	1
9	2284	12	Placebo	0	13	6/11/2016	78.6	LENGTH	9.35	14.5	30	0	1
9	2284	24	Placebo	0	24	4/20/2017	83.5	LENGTH	11.43181818	16	30	1	1
9	2288	0	Placebo	0	24	3/14/2015	78.3	LENGTH	8.95	13.4		0	1
9	2288	12	Placebo	0	24	6/11/2016	89.2	HEIGHT	11.8	14		0	1
9	2288	24	Placebo	0	35	4/20/2017	98	HEIGHT	12.95454545	14		0	1
9	2288	36	Placebo	0	55	5/12/2018	105	HEIGHT	15	13.5		0	1

9	2288	60	Placebo	0	76	2/5/2020	117.8	HEIGHT	18.8	14		0	1
9	2291	24	Placebo	1	55	4/20/2017	99.7	HEIGHT	15.22727273	15		1	1
9	2292	0	Placebo	1	48	3/14/2015	100	HEIGHT	15.4	15.8		1	1
9	2292	12	Placebo	1	54	6/11/2016	106.4	HEIGHT	17.45	16		0	1
9	2292	24	Placebo	1	65	4/20/2017	112	HEIGHT	18.13636364	16		0	1
9	2292	36	Placebo	1	80	5/12/2018	116.5	HEIGHT	19.5	15.2		0	1
9	2292	48	Placebo	1	91	4/10/2019	120.5	HEIGHT	22.9	16.6		0	1
9	2292	60	Placebo	1	100	2/5/2020	126.1	HEIGHT	24	17		0	1
9	2293	0	Placebo	1	42	3/14/2015	91.7	HEIGHT	13	14.9		0	1
9	2293	36	Placebo	1	75	5/12/2018	113	HEIGHT	17.8	14.5		0	1
9	2293	48	Placebo	1	86	4/10/2019	119.1	HEIGHT	19.7	15		0	1
9	2294	0	Placebo	0	54	3/14/2015	99.2	HEIGHT	13.95	15.2		1	1
9	2294	12	Placebo	0	57	6/11/2016	105	HEIGHT	15.75	14.5		0	1
9	2294	24	Placebo	0	69	4/20/2017	109	HEIGHT	16.13636364	15		0	1
9	2294	36	Placebo	0	83	5/12/2018	114	HEIGHT	17.9	14.6		0	1
9	2294	48	Placebo	0	94	4/10/2019	117.4	HEIGHT	18.6	15.1		0	1
9	2295	0	Placebo	1	54	3/14/2015	103	HEIGHT	15.85	15.9		0	1
9	2295	12	Placebo	1	69	6/12/2016	108.6	HEIGHT	17.95	17		0	1
9	2295	24	Placebo	1	78	4/20/2017	112	HEIGHT	19.04545455	16		0	1
9	2295	36	Placebo	1	92	5/12/2018	118	HEIGHT	20.4	15.5		0	1
9	2296	0	Placebo	1	19	3/14/2015	74.3	LENGTH	8.65	14.4		0	1
9	2296	12	Placebo	1	30	6/11/2016	84	HEIGHT	11.2	14.5		0	1
9	2296	24	Placebo	1	41	4/20/2017	90	HEIGHT	13.95454545	16		0	1
9	2296	36	Placebo	1	56	5/12/2018	97	HEIGHT	15.4	15.5		0	1
9	2296	48	Placebo	1	67	4/10/2019	102	HEIGHT	16.7	16		0	1
9	2299	0	Placebo	1	7	3/14/2015	63.8	LENGTH	6.55	13.4		1	1
9	2299	12	Placebo	1	17	6/11/2016	77.5	HEIGHT	8.95	13		0	1
9	2299	24	Placebo	1	27	4/20/2017	82.5	HEIGHT	11.68181818	15		0	1
9	2299	48	Placebo	1	52	4/10/2019	99.4	HEIGHT	14.4	15.4		0	1
9	2301	0	Placebo	0	24	3/14/2015	77.3	LENGTH	9.1	12.9		0	1
9	2301	12	Placebo	0	36	6/12/2016	86.7	HEIGHT	10.75	13.5		0	1
9	2301	24	Placebo	0	48	4/20/2017	93	HEIGHT	11.63636364	13		0	1
9	2301	36	Placebo	0	63	5/12/2018	98.5	HEIGHT	12.95	12.6		0	1
9	2301	48	Placebo	0	73	4/10/2019	104	HEIGHT	14.55	13.2		0	1
9	2302	0	Placebo	1	18	3/14/2015	73.8	LENGTH	8.7	14.2	18	1	1
9	2302	12	Placebo	1	36	6/11/2016	81.8	LENGTH	10.6	14.5	18	1	1
9	2303	12	Placebo	1	1	6/11/2016	57.3	LENGTH	4.5	11.5		0	1
9	2303	24	Placebo	1	12	4/20/2017	69.3	LENGTH	6.863636364	13		0	1
9	2303	36	Placebo	1	26	5/12/2018	72.9	HEIGHT	8.15	12.2		0	1
9	2303	60	Placebo	1	47	2/5/2020	85.2	HEIGHT	11.5	14		1	1

9	2304	0	Placebo	1	36	3/14/2015	79.2	HEIGHT	11.1	15		1	1
9	2304	12	Placebo	1	45	6/12/2016	88	HEIGHT	12.9	14.5		0	1
9	2304	24	Placebo	1	57	4/20/2017	94	HEIGHT	14.13636364	14.5		0	1
9	2304	36	Placebo	1	71	5/12/2018	100	HEIGHT	14.35	14		0	1
9	2304	48	Placebo	1	82	4/10/2019	104.3	HEIGHT	15.1	14		0	1
9	2304	60	Placebo	1	92	2/5/2020	107.8	HEIGHT	16.9	14.6		0	1
9	2305	0	Placebo	0	48	3/14/2015	97	HEIGHT	15.2	16.7		0	1
9	2305	12	Placebo	0	61	6/11/2016	102.8	LENGTH	16.15	15		0	1
9	2305	24	Placebo	0	72	4/20/2017	109.4	HEIGHT	17.72727273	15.5		0	1
9	2305	48	Placebo	0	97	4/10/2019	118.4	HEIGHT	20.2	15.7		0	1
9	2305	60	Placebo	0	107	2/5/2020	122.1	HEIGHT	22.8	17		0	1
9	2307	0	Placebo	1	1	3/14/2015	59.1	LENGTH	5.95	13.9		0	1
9	2307	12	Placebo	1	14	6/11/2016	78.3	LENGTH	9.95	15		0	1
9	2307	24	Placebo	1	25	4/20/2017	88.9	LENGTH	13.63636364	15.5		0	1
9	2307	36	Placebo	1	40	5/12/2018	98	HEIGHT	15.45	15.8		0	1
9	2307	48	Placebo	1	51	4/10/2019	105.6	HEIGHT	16.6	15.1		1	1
9	2308	0	Placebo	0	36	3/14/2015	96.4	HEIGHT	13.75	15.5		1	1
9	2308	12	Placebo	0	49	6/11/2016	105	HEIGHT	15.05	13		0	1
9	2308	24	Placebo	0	60	4/20/2017	110	HEIGHT	16.18181818	15		0	1
9	2308	36	Placebo	0	74	5/12/2018	116.4	HEIGHT	17.9	14.5		0	1
9	2308	48	Placebo	0	85	4/10/2019	122	HEIGHT	18.6	14.6		0	1
9	2308	60	Placebo	0	95	2/5/2020	127.5	HEIGHT	21.6	16		0	1
9	2312	12	Placebo	0	54	6/11/2016	102.3	HEIGHT	14.75	14		1	1
9	2315	0	Placebo	0	24	3/14/2015	69.7	LENGTH	7.75	13.7	12	0	1
9	2316	12	Placebo	0	6	6/11/2016	67.4	LENGTH	7.4	14	18	1	1
9	2317	0	Placebo	0	9	3/14/2015	73.8	LENGTH	8.1	14		0	1
9	2317	24	Placebo	0	29	4/20/2017	97	HEIGHT	12.95454545	14		0	1
9	2317	36	Placebo	0	44	5/12/2018	106.1	HEIGHT	16	15		1	1
9	2317	48	Placebo	0	55	4/10/2019	112.5	HEIGHT	17.75	14.5		0	1
9	2317	60	Placebo	0	64	2/5/2020	118.6	HEIGHT	19	15		0	1
9	2318	12	Placebo	0	6	6/11/2016	64.2	LENGTH	5.85	12		0	1
9	2318	24	Placebo	0	5	4/20/2017	70	HEIGHT	7.227272727	11		0	1
9	2318	36	Placebo	0	19	5/12/2018	79.4	HEIGHT	10.2	13.5		0	1
9	2318	60	Placebo	0	40	2/5/2020	94.2	HEIGHT	13.9	14.3		0	1
9	2319	0	Placebo	1	34	6/3/2015	76.8	LENGTH	9.3	13.8		0	1
9	2320	0	Placebo	1	36	3/14/2015	81.1	LENGTH	10.7	15.4		1	1
9	2320	36	Placebo	1	58	5/12/2018	105.5	HEIGHT	16.3	15.4		1	1
9	2320	48	Placebo	1	69	4/10/2019	110.8	HEIGHT	18.1	15.2		0	1
9	2322	0	Placebo	1	8	3/14/2015	64.9	LENGTH	7.7	15.3		1	1
9	2322	12	Placebo	1	20	6/11/2016	77.4	HEIGHT	10.2	15		0	1

9	2322	24	Placebo	1	29	4/20/2017	84	HEIGHT	12.31818182	14.5	0	1
9	2322	36	Placebo	1	44	5/12/2018	91.1	HEIGHT	13.2	15.1	0	1
9	2323	0	Placebo	1	40	3/14/2015	88.4	HEIGHT	12.15	15.6	0	1
9	2323	12	Placebo	1	61	6/11/2016	97	HEIGHT	14.8	15.5	0	1
9	2323	24	Placebo	1	72	4/20/2017	101.6	HEIGHT	14.68181818	15	0	1
9	2323	36	Placebo	1	86	5/12/2018	106.6	HEIGHT	16.7	15.5	0	1
9	2323	48	Placebo	1	97	4/10/2019	109.9	HEIGHT	17.3	15.1	0	1
9	2323	60	Placebo	1	107	2/5/2020	113.5	HEIGHT	18.2	15.5	0	1
9	2324	0	Placebo	0	42	3/14/2015	91.6	HEIGHT	14.3	16.1	0	1
9	2324	12	Placebo	0	45	6/11/2016	98.9	HEIGHT	15.45	15	0	1
9	2324	48	Placebo	0	78	4/10/2019	114.5	HEIGHT	21.6	15.7	0	1
9	2330	24	Placebo	0	47	4/20/2017	99	HEIGHT	16.18181818	16.5	1	1
9	2331	0	Placebo	1	25	3/14/2015	80.9	HEIGHT	9.95	15	1	1
9	2331	12	Placebo	1	42	6/11/2016	89	HEIGHT	12.4	14.5	0	1
9	2331	24	Placebo	1	53	4/20/2017	96.5	HEIGHT	14.27272727	15.5	1	1
9	2331	36	Placebo	1	68	5/12/2018	103.4	HEIGHT	15.1	15	0	1
9	2331	48	Placebo	1	79	4/10/2019	109.9	HEIGHT	17	15	0	1
9	2331	60	Placebo	1	88	2/5/2020	115.3	HEIGHT	18.6	15.4	0	1
9	2334	24	Placebo	0	46	4/20/2017	92.6	HEIGHT	13	14.9	1	1
9	2335	24	Placebo	1	35	4/20/2017	87	HEIGHT	12.54545455	15	1	1
9	2336	0	Placebo	1	48	3/14/2015	95.2	HEIGHT	14.8	16.5	0	1
9	2336	12	Placebo	1	53	6/12/2016	103.3	HEIGHT	16.95	16.5	0	1
9	2336	24	Placebo	1	63	4/20/2017	110.5	HEIGHT	18.72727273	16.5	0	1
9	2336	48	Placebo	1	88	4/10/2019	122.5	HEIGHT	22.75	16.2	0	1
9	2336	60	Placebo	1	98	2/5/2020	126.4	HEIGHT	25.9	18.7	0	1
9	2337	12	Placebo	0	36	6/12/2016	84.5	HEIGHT	10	13	1	1
9	2338	0	Placebo	0	12	3/14/2015	67.5	LENGTH	6.6	13.1	1	1
9	2338	24	Placebo	0	35	4/20/2017	79	HEIGHT	8.954545455	12.5	1	1
9	2338	36	Placebo	0	49	5/12/2018	84.4	HEIGHT	9.6	13	0	1
9	2338	48	Placebo	0	60	4/10/2019	89	HEIGHT	10.4	12.5	0	1
9	2340	0	Placebo	0	52	3/14/2015	100.5	HEIGHT	15.4	15.3	0	1
9	2340	12	Placebo	0	60	6/11/2016	105.9	HEIGHT	17.5	15	0	1
9	2340	24	Placebo	0	72	4/20/2017	111	HEIGHT	18.40909091	15	0	1
9	2340	36	Placebo	0	99	5/12/2018	116.5	HEIGHT	20.25	15.8	0	1
9	2340	48	Placebo	0	110	4/10/2019	121	HEIGHT	22.15	17	0	1
9	2340	60	Placebo	0	120	2/5/2020	123.7	HEIGHT	23.3	17	0	1
9	2341	0	Placebo	0	3	3/14/2015	60.8	LENGTH	6.7	15.4	1	1
9	2344	0	Placebo	0	25	3/14/2015	80.2	LENGTH	9.5	13.7	0	1
9	2344	12	Placebo	0	38	6/11/2016	87	HEIGHT	12.04	14.5	0	1
9	2344	24	Placebo	0	50	4/20/2017	95	HEIGHT	12.90909091	13.5	0	1

9	2344	36	Placebo	0	64	5/12/2018	103	HEIGHT	14.35	13.5		0	1
9	2344	48	Placebo	0	75	4/10/2019	109	HEIGHT	15.85	14		0	1
9	2344	60	Placebo	0	85	2/5/2020	112.6	HEIGHT	18.2	14.3		0	1
9	2345	12	Placebo	0	39	6/12/2016	96	HEIGHT	14.05	15		1	1
9	2345	24	Placebo	0	51	4/20/2017	101.1	HEIGHT	15	15.5		1	1
9	2346	0	Placebo	1	5	3/14/2015	62.4	LENGTH	5.85	11.6		0	1
9	2346	12	Placebo	1	18	6/11/2016	75.3	LENGTH	8.5	14		0	1
9	2346	24	Placebo	1	29	4/20/2017	79.5	HEIGHT	9.909090909	13.5		0	1
9	2346	36	Placebo	1	44	5/12/2018	88	HEIGHT	11.1	12.7		0	1
9	2346	48	Placebo	1	55	4/10/2019	95.7	HEIGHT	11.65	12.5		0	1
9	2349	0	Placebo	0	4	3/14/2015	62.5	LENGTH	6.3	13.8		0	1
9	2349	12	Placebo	0	12	6/11/2016	74.1	LENGTH	8.55	13.5		0	1
9	2349	24	Placebo	0	30	4/20/2017	81	HEIGHT	10.81818182	14.5		0	1
9	2349	36	Placebo	0	44	5/12/2018	90	HEIGHT	12.65	14		0	1
9	2349	48	Placebo	0	4	4/10/2019	95.4	HEIGHT	13.7	14.5		0	1
9	2349	60	Placebo	0	56	2/5/2020	101.4	HEIGHT	15.4	14.4		0	1
9	2350	0	Placebo	1	36	3/14/2015	85.3	HEIGHT	11.3	15.5		0	1
9	2351	0	Placebo	0	54	3/14/2015	101.7	HEIGHT	14.45	14.1		1	1
9	2351	12	Placebo	0	65	6/12/2016	108.2	HEIGHT	15.65	14		0	1
9	2351	36	Placebo	0	102	5/12/2018	121.5	HEIGHT	19.25	13.5		0	1
9	2352	12	Placebo	0	12	6/11/2016	78.3	HEIGHT	11.65	15	18	1	1
9	2354	0	Placebo	0	18	3/14/2015	75.6	LENGTH	8.6	14.5		0	1
9	2354	12	Placebo	0	27	6/11/2016	85.2	HEIGHT	10.35	13.5		0	1
9	2354	36	Placebo	0	56	5/12/2018	100	HEIGHT	14.15	13.5		0	1
9	2355	12	Placebo	0	36	6/11/2016	79.2	HEIGHT	9.5	13.5		1	1
9	2362	24	Placebo	1	47	4/20/2017	109	HEIGHT	15.09090909	14		1	1
9	2364	12	Placebo	1	22	6/11/2016	80	HEIGHT	8.95	12.5		1	1
9	2364	36	Placebo	1	47	5/12/2018	93	HEIGHT	12	13.5		1	1
9	2368	0	Placebo	1	36	3/14/2015	86.5	HEIGHT	11.6	15.7		0	1
9	2368	12	Placebo	1	45	6/12/2016	93.9	HEIGHT	13.15	15		0	1
9	2368	24	Placebo	1	57	4/20/2017	102	HEIGHT	14.54545455	15		0	1
9	2368	36	Placebo	1	72	5/12/2018	110.5	HEIGHT	15.75	14.9		0	1
9	2368	48	Placebo	1	83	4/10/2019	115.6	HEIGHT	17.45	14.5		0	1
9	2368	60	Placebo	1	93	2/5/2020	119.1	HEIGHT	18.7	14.9		0	1
9	2369	0	Placebo	1	48	3/14/2015	100.2	HEIGHT	14.25	15.3		0	1
9	2369	12	Placebo	1	63	6/11/2016	106	HEIGHT	15.65	14.5		0	1
9	2369	36	Placebo	1	86	5/12/2018	118.5	HEIGHT	18.85	15		0	1
9	2369	48	Placebo	1	97	4/10/2019	124	HEIGHT	20.75	15.4		0	1
9	2369	60	Placebo	1	107	2/5/2020	131	HEIGHT	22.7	17		0	1
9	2372	0	Placebo	0	36	3/14/2015	98.5	HEIGHT	15.05	15.6		1	1

9	2372	12	Placebo	0	54	6/11/2016	106.3	HEIGHT	15.6	14		0	1
9	2372	24	Placebo	0	65	4/20/2017	109.5	HEIGHT	17.13636364	14.5		0	1
9	2372	36	Placebo	0	80	5/12/2018	115	HEIGHT	18.1	14.5		0	1
9	2372	48	Placebo	0	91	4/10/2019	120	HEIGHT	20.3	14.6		0	1
9	2373	24	Placebo	1	31	4/20/2017	88.3	LENGTH	11.13636364	15		1	1
9	2374	0	Placebo	1	42	6/3/2015	97.5	HEIGHT	14.05	17		0	1
9	2374	12	Placebo	1	45	6/12/2016	102	HEIGHT	15.4	15.8		0	1
9	2377	0	Placebo	1	35	3/14/2015	82.1	HEIGHT	9.5	13.7		0	1
9	2377	12	Placebo	1	48	6/11/2016	93.5	HEIGHT	12	13.5		1	1
9	2377	24	Placebo	1	60	4/20/2017	98	HEIGHT	13.09090909	13.5		0	1
9	2377	36	Placebo	1	74	5/12/2018	104.5	HEIGHT	14.85	13.7		0	1
9	2377	48	Placebo	1	85	4/10/2019	110.9	HEIGHT	16.45	13.5		0	1
9	2378	12	Placebo	1	48	6/11/2016	92	HEIGHT	13.05	14		1	1
9	2381	24	Placebo	1	20	4/20/2017	87.3	LENGTH	13.13636364	15	42	1	1
9	2383	0	Placebo	0	37	3/14/2015	83.5	HEIGHT	9.85	12.9		0	1
9	2383	12	Placebo	0	50	6/11/2016	93.5	HEIGHT	12	13		0	1
9	2383	24	Placebo	0	61	4/20/2017	99.5	HEIGHT	13.5	14.5		0	1
9	2383	36	Placebo	0	76	5/12/2018	106	HEIGHT	14.2	13.2		0	1
9	2383	60	Placebo	0	96	2/5/2020	115.7	HEIGHT	16.9	13.5		0	1
9	2385	12	Placebo	0	4	6/11/2016	70	HEIGHT	7.75	15		1	1
9	2385	24	Placebo	0	14	4/20/2017	78	HEIGHT	9.227272727	15		0	1
9	2385	36	Placebo	0	30	5/12/2018	84.5	HEIGHT	11.45	15.5		1	1
9	2385	48	Placebo	0	41	4/10/2019	90	HEIGHT	12	14.8		0	1
9	2385	60	Placebo	0	50	2/5/2020	96.5	HEIGHT	14.2	14.6		0	1
9	2387	24	Placebo	1	55	4/20/2017	106	HEIGHT	17.31818182	17		1	1
9	2393	0	Placebo	1	52	3/14/2015	96.7	HEIGHT	13.8	14.5		1	1
9	2393	12	Placebo	1	66	6/11/2016	102.5	HEIGHT	15.25	14.5		0	1
9	2393	24	Placebo	1	77	4/20/2017	109.1	HEIGHT	16.13636364	14.5		0	1
9	2395	0	Placebo	0	36	3/14/2015	84.5	HEIGHT	12.4	17		0	1
9	2395	12	Placebo	0	43	6/11/2016	95	HEIGHT	14.4	15		0	1
9	2395	24	Placebo	0	54	4/20/2017	96.3	LENGTH	15.27272727	15		0	1
9	2395	36	Placebo	0	69	5/12/2018	103.5	HEIGHT	16.5	15.5		0	1
9	2395	48	Placebo	0	80	4/10/2019	108.4	HEIGHT	18.1	16		0	1
9	2395	60	Placebo	0	90	2/5/2020	112.6	HEIGHT	20.1	16		0	1
9	2396	12	Placebo	1	33	6/12/2016	84	HEIGHT	12.55	16		0	1
9	2396	24	Placebo	1	45	4/20/2017	94	HEIGHT	14.59090909	16		0	1
9	2398	12	Placebo	1	15	6/11/2016	81.3	LENGTH	11.8	15.5		1	1
9	2398	36	Placebo	1	41	5/12/2018	98	HEIGHT	16.7	17		1	1
9	2398	48	Placebo	1	52	4/10/2019	104.7	HEIGHT	17.9	15.4		1	1
9	2400	12	Placebo	1	11	6/12/2016	70.8	LENGTH	7.6	13		0	1

9	2400	36	Placebo	1	35	5/12/2018	83	HEIGHT	12.5	15.5		1	1
9	2401	0	Placebo	1	30	3/14/2015	78.1	LENGTH	9.45	14.1	6	0	1
9	2402	24	Placebo	1	47	4/20/2017	101.5	HEIGHT	15.68181818	15.5		1	1
9	2403	0	Placebo	0	36	3/14/2015	78.3	LENGTH	10.65	15.9		1	1
9	2403	12	Placebo	0	30	6/11/2016	86	HEIGHT	13.15	16.5		1	1
9	2403	24	Placebo	0	41	4/20/2017	94.5	HEIGHT	15.54545455	16		1	1
9	2403	36	Placebo	0	56	5/12/2018	102	HEIGHT	16.35	15		0	1
9	2403	48	Placebo	0	67	4/10/2019	106.1	HEIGHT	17.65	14.7		0	1
9	2403	60	Placebo	0	76	2/5/2020	111.4	HEIGHT	19	14.6		0	1
9	2404	12	Placebo	0	12	6/11/2016	75.8	LENGTH	9	14		0	1
9	2404	24	Placebo	0	21	4/20/2017	83.8	LENGTH	12.40909091	15		0	1
9	2404	36	Placebo	0	35	5/12/2018	93	HEIGHT	14.5	14		0	1
9	2405	12	Placebo	1	8	6/11/2016	69.6	LENGTH	6.9	13.5		0	1
9	2405	24	Placebo	1	20	4/20/2017	79	HEIGHT	9.272727273	14		0	1
9	2405	36	Placebo	1	34	5/12/2018	88	HEIGHT	11.65	14.5		0	1
9	2405	48	Placebo	1	45	4/10/2019	95.1	HEIGHT	12.95	13.7		1	1
9	2405	60	Placebo	1	55	2/5/2020	101.1	HEIGHT	14.95	14.9		0	1
9	2408	12	Placebo	1	6	6/11/2016	65.5	LENGTH	6.95	14		0	1
9	2408	36	Placebo	1	32	5/12/2018	84.2	HEIGHT	10.85	14		0	1
9	2408	60	Placebo	1	53	2/5/2020	97.3	LENGTH	13.7	15		1	1
9	2409	24	Placebo	0	48	4/20/2017	96	HEIGHT	13.45454545	14.5		1	1
9	2411	0	Placebo	1	24	3/14/2015	77.7	LENGTH	9.7	14.1		1	1
9	2411	12	Placebo	1	33	6/12/2016	87.4	HEIGHT	11.4	14		0	1
9	2411	24	Placebo	1	45	4/20/2017	95.2	HEIGHT	11.86363636	14.3		0	1
9	2411	36	Placebo	1	59	5/12/2018	103.1	HEIGHT	14.4	14		0	1
9	2411	60	Placebo	1	80	2/5/2020	115.1	HEIGHT	17.1	15		0	1
9	2412	12	Placebo	0	26	6/12/2016	86.3	LENGTH	11.9	15.5		1	1
9	2413	0	Placebo	1	48	3/14/2015	101.5	HEIGHT	15.55	15.2		0	1
9	2413	12	Placebo	1	42	6/11/2016	108.4	HEIGHT	17.05	14		0	1
9	2413	24	Placebo	1	62	4/20/2017	115.5	HEIGHT	17.63636364	14		0	1
9	2413	36	Placebo	1	76	5/12/2018	120.4	HEIGHT	19.5	14.5		0	1
9	2413	48	Placebo	1	87	4/10/2019	125.1	HEIGHT	20.8	14.5		0	1
9	2413	60	Placebo	1	97	2/5/2020	127.5	HEIGHT	22.7	15		0	1
9	2414	0	Placebo	1	54	3/14/2015	106.3	HEIGHT	15.85	15.3		0	1
9	2414	12	Placebo	1	74	6/11/2016	112	HEIGHT	16.75	14.5		0	1
9	2414	24	Placebo	1	94	4/20/2017	115	HEIGHT	16.77272727	13.5		0	1
9	2414	36	Placebo	1	109	5/12/2018	119	HEIGHT	19.7	15		0	1
9	2414	48	Placebo	1	119	4/10/2019	122.9	HEIGHT	20.75	15.4		0	1
9	2417	24	Placebo	0	20	4/20/2017	72	HEIGHT	8.181818182	12		1	1
9	2419	12	Placebo	0	6	6/11/2016	66.7	LENGTH	6.95	13.5	36	0	1

9	2419	24	Placebo	0	17	4/20/2017	76	HEIGHT	9.045454545	14	36	0	1
9	2420	12	Placebo	1	24	6/12/2016	67.5	HEIGHT	9.85	14		1	1
9	8001	36	Placebo	1	44	5/12/2018	87.5	HEIGHT	12.6	15.5		1	1
9	8012	60	Placebo	0	56	2/5/2020	99.2	HEIGHT	14.2	14		1	1
9	8016	36	Placebo	0	21	5/12/2018	80.5	HEIGHT	10.1	14		1	1
9	8054	60	Placebo	0	41	2/5/2020	90.3	HEIGHT	12.8	14.5		1	1
9	8081	36	Placebo	0	15	5/12/2018	70.2	HEIGHT	7.35	12.5		1	1
9	8081	48	Placebo	0	26	4/10/2019	77.2	HEIGHT	8.9	13		0	1
9	8081	60	Placebo	0	36	2/5/2020	82.2	HEIGHT	11.1	14.5		0	1
9	8095	60	Placebo	1	5	2/5/2020	65.9	HEIGHT	6.8	14		1	1
9	8103	60	Placebo	0	29	2/5/2020	82.1	HEIGHT	9.5	14		1	1
9	8120	60	Placebo	0	14	2/5/2020	70.6	LENGTH	7.9	15		1	1
9	8128	36	Placebo	0	48	5/12/2018	91.2	HEIGHT	12.3	15		1	1
9	8138	36	Placebo	1	26	5/12/2018	77	HEIGHT	8.9	14		1	1
9	8138	60	Placebo	1	46	2/5/2020	89.1	HEIGHT	11.8	14.5		1	1
9	8205	36	Placebo	1	9	5/12/2018	68.5	LENGTH	6.2	11.5		1	1
9	8205	48	Placebo	1	15	4/10/2019	74.3	LENGTH	7.2	10.5		0	1
9	8205	60	Placebo	1	25	2/5/2020	81.4	HEIGHT	9.5	12.5		0	1
9	8215	60	Placebo	0	14	2/5/2020	79.9	HEIGHT	12	16		1	1
9	8219	48	Placebo	0	14	4/10/2019	73.4	LENGTH	9.5	14.5		1	1
9	8221	60	Placebo	1	28	2/5/2020	80.5	HEIGHT	9.1	12.2		1	1
9	8262	48	Placebo	1	30	4/10/2019	85.5	HEIGHT	11.75	14.5		1	1
9	8273	60	Placebo	1	21	2/5/2020	75.3	HEIGHT	8.7	13.5		1	1
9	8292	48	Placebo	1	26	4/10/2019	81.5	LENGTH	9.85	13.1	60	1	1
9	8298	36	Placebo	1	20	5/12/2018	71.7	LENGTH	8.05	14.2		1	1
9	8298	60	Placebo	1	41	2/5/2020	85.5	HEIGHT	11.9	15		1	1
9	8330	36	Placebo	0	56	5/12/2018	100.6	HEIGHT	15.6	15.5		1	1
9	8367	36	Placebo	1	8	5/12/2018	63.8	LENGTH	6.15	13.8		1	1
9	8367	48	Placebo	1	19	4/10/2019	72.8	LENGTH	7.75	12.5		0	1
9	8392	36	Placebo	1	39	5/12/2018	87.9	HEIGHT	11.45	13		1	1
9	8415	60	Placebo	1	52	2/5/2020	92.8	HEIGHT	12.7	14.3		1	1
9	8460	60	Placebo	0	57	2/5/2020	95.1	HEIGHT	13.6	14.2		1	1
9	8477	60	Placebo	0	48	2/5/2020	102.3	HEIGHT	17.5	16		1	1
9	8489	36	Placebo	1	3	5/12/2018	57.6	HEIGHT	5.65	13.5		1	1
9	8489	48	Placebo	1	14	4/10/2019	67.5	LENGTH	6.5	12.2		0	1
9	8544	36	Placebo	1	56	5/12/2018	96	HEIGHT	13.85	14.5		1	1
9	8547	36	Placebo	1	47	5/12/2018	95.1	HEIGHT	13.55	15.3		1	1
9	8573	36	Placebo	1	24	5/12/2018	80.8	LENGTH	9.6	13		1	1
9	8577	60	Placebo	1	44	2/5/2020	94.8	HEIGHT	13	15		1	1
9	8578	36	Placebo	0	15	5/12/2018	71.8	LENGTH	7.95	13.9		1	1

9	8583	36	Placebo	1	37	5/12/2018	79.9	HEIGHT	9.3	12.5		1	1
9	8583	60	Placebo	1	58	2/5/2020	93.4	HEIGHT	12.6	13		1	1
9	8620	48	Placebo	0	4	4/10/2019	63.3	LENGTH	6.45	13		1	1
9	8633	48	Placebo	0	1	4/10/2019	60.7	LENGTH	7.25	13.3		1	1
9	8633	60	Placebo	0	11	2/5/2020	68.2	LENGTH	6.5	13		1	1
9	8652	36	Placebo	1	53	5/12/2018	92.9	HEIGHT	12.55	13.5	48	1	1
9	8662	48	Placebo	1	23	4/10/2019	83.5	HEIGHT	12.05	14.2		1	1
9	8685	48	Placebo	1	34	4/10/2019	85.4	HEIGHT	10.6	13.2		1	1
9	8700	60	Placebo	0	19	2/5/2020	74.8	LENGTH	7.8	12		1	1
9	8726	48	Placebo	0	10	4/10/2019	69.3	LENGTH	6.75	12		1	1
9	8726	60	Placebo	0	20	2/5/2020	75.5	LENGTH	7.7	11.1		0	1
9	8759	60	Placebo	0	31	2/5/2020	85.8	HEIGHT	12.6	14.4		1	1
9	8761	48	Placebo	1	12	4/10/2019	64.8	LENGTH	6.4	13		1	1
9	8764	48	Placebo	1	33	4/10/2019	85.5	HEIGHT	10.55	13.5		1	1
9	8769	48	Placebo	0	6	4/10/2019	65.4	LENGTH	8.2	14		1	1
9	8769	60	Placebo	0	16	2/5/2020	71.2	HEIGHT	9.2	14.3		0	1
9	8793	60	Placebo	0	46	2/5/2020	97.9	HEIGHT	17.1	17		1	1
9	8834	48	Placebo	1	16	4/10/2019	69.8	LENGTH	7.75	12.7		1	1
9	8843	36	Placebo	0	47	5/12/2018	92	HEIGHT	12.7	14.2		1	1
9	8853	36	Placebo	0	22	5/12/2018	76.3	LENGTH	8.95	15		1	1
9	8858	60	Placebo	0	24	2/5/2020	73.3	HEIGHT	8.3	13.5		1	1
9	8863	36	Placebo	0	12	5/12/2018	66.8	LENGTH	7.75	14.8		1	1
9	8863	60	Placebo	0	28	2/5/2020	82.3	HEIGHT	12	15.3		0	1
9	8868	60	Placebo	0	3	2/5/2020	61.4	LENGTH	6.6	13		1	1
9	8888	36	Placebo	0	45	5/12/2018	96.3	HEIGHT	15	15.9		1	1
9	8890	60	Placebo	1	18	2/5/2020	76.9	HEIGHT	9.2	14.2		1	1
9	8899	36	Placebo	1	46	5/12/2018	98	HEIGHT	13.4	13.5		1	1
9	8931	48	Placebo	0	22	4/10/2019	79.3	LENGTH	9.35	14.2		1	1
9	8938	36	Placebo	1	47	5/12/2018	96.4	HEIGHT	13.15	13.7		1	1
9	8964	48	Placebo	0	43	4/10/2019	91.5	HEIGHT	13.5	14		1	1
9	8995	60	Placebo	0	42	2/5/2020	90.5	HEIGHT	13.8	16		1	1
9	8996	48	Placebo	1	33	4/10/2019	78.4	LENGTH	9.3	13.2		1	1
9	9008	60	Placebo	0	4	2/5/2020	68	LENGTH	6.5	15		1	1
9	9009	60	Placebo	0	32	2/5/2020	86.6	HEIGHT	12.2	14.2		1	1
9	9014	60	Placebo	1	20	2/5/2020	75.3	HEIGHT	8.8	14		1	1
9	9035	60	Placebo	0	11	2/5/2020	67.2	LENGTH	5.7	11.7		1	1
9	9043	48	Placebo	1	46	4/10/2019	93.1	HEIGHT	12.55	14		1	1
9	9043	60	Placebo	1	56	2/5/2020	100.7	HEIGHT	14.7	14.2		1	1
9	9071	60	Placebo	0	14	2/5/2020	73.2	HEIGHT	9.6	13.4		1	1
9	9113	48	Placebo	0	23	4/10/2019	78.3	LENGTH	8.55	12.5		1	1

9	9154	36	Placebo	0	9	5/12/2018	66.8	LENGTH	6.55	13		1	1
9	9154	48	Placebo	0	20	4/10/2019	77.9	LENGTH	8.9	13		1	1
9	9154	60	Placebo	0	29	2/5/2020	85.1	HEIGHT	11	14		0	1
9	9165	48	Placebo	0	10	4/10/2019	73.3	LENGTH	10.25	16		1	1
9	9170	48	Placebo	0	8	4/10/2019	66.8	LENGTH	7.3	12.5		1	1
9	9170	60	Placebo	0	18	2/5/2020	72.2	LENGTH	8.2	13.3		1	1
9	9187	60	Placebo	1	1	2/5/2020	52.5	LENGTH	4.4	12.2		1	1
9	9252	48	Placebo	0	35	4/10/2019	87.3	HEIGHT	11.25	13.5		1	1
9	9269	36	Placebo	0	14	5/12/2018	74.3	LENGTH	8.6	13.5		1	1
9	9290	48	Placebo	1	20	4/10/2019	78.2	LENGTH	9.85	14		1	1
9	9294	36	Placebo	1	20	5/12/2018	74.6	HEIGHT	7.7	12.7		1	1
9	9294	48	Placebo	1	31	4/10/2019	80.7	LENGTH	10.2	14.5		1	1
9	9305	36	Placebo	0	2	5/12/2018	59.8	LENGTH	6.2	13.5		1	1
9	9305	48	Placebo	0	13	4/10/2019	73.8	LENGTH	8.7	13.5		0	1
9	9305	60	Placebo	0	23	2/5/2020	81.7	HEIGHT	10.2	14.3		0	1
9	9344	60	Placebo	1	45	2/5/2020	94.4	HEIGHT	12	13		1	1
9	9345	60	Placebo	0	8	2/5/2020	67.5	LENGTH	8.1	14.5		1	1
9	9364	60	Placebo	1	48	2/5/2020	97.1	HEIGHT	13.6	14		1	1
9	9367	48	Placebo	0	45	4/10/2019	89.4	HEIGHT	11.4	13		1	1
9	9429	48	Placebo	0	11	4/10/2019	73.4	LENGTH	8.7	14		1	1
9	9429	60	Placebo	0	21	2/5/2020	81.2	HEIGHT	10.4	16		0	1
9	9445	48	Placebo	0	24	4/10/2019	82	HEIGHT	10.2	13		1	1
9	9450	48	Placebo	0	7	4/10/2019	67.3	LENGTH	7.25	13.7		1	1
9	9466	36	Placebo	0	50	5/12/2018	95.1	HEIGHT	12.75	14		1	1
9	9468	36	Placebo	0	37	5/12/2018	89.6	HEIGHT	11.8	13.3		1	1
9	9482	36	Placebo	1	11	5/12/2018	68.6	LENGTH	8.35	15.5		1	1
9	9482	48	Placebo	1	22	4/10/2019	80	HEIGHT	10.55	15.2		0	1
9	9482	60	Placebo	1	31	2/5/2020	86.9	HEIGHT	13.1	16.5		0	1
9	9488	60	Placebo	1	12	2/5/2020	73.2	HEIGHT	9.8	14.5		1	1
9	9498	60	Placebo	0	5	2/5/2020	57.2	LENGTH	4.5	10.5		1	1
9	9530	48	Placebo	1	57	4/10/2019	98	HEIGHT	13.75	14		1	1
9	9544	36	Placebo	1	23	5/12/2018	77.6	HEIGHT	9.95	13.5		1	1
9	9574	48	Placebo	0	14	4/10/2019	70.4	LENGTH	8.75	14		1	1
10	2431	24	Placebo	1	23	5/25/2017	80.2	HEIGHT	8.35	12		1	0
10	2431	48	Placebo	1	46	6/25/2019	94.6	HEIGHT	13.04545455	13.5		1	0
10	2432	12	Placebo	1	7	6/17/2016	68.8	LENGTH	6.55	13		1	0
10	2432	36	Placebo	1	29	5/30/2018	89.6	HEIGHT	11.1	14		0	0
10	2432	48	Placebo	1	40	5/18/2019	96.6	HEIGHT	12.95	14		1	0
10	2432	60	Placebo	1	49	2/26/2020	102	HEIGHT	14.22727273	14		1	0
10	2434	0	Placebo	1	11	3/17/2015	60.6	LENGTH	5.3	12		0	0

10	2434	12	Placebo	1	24	6/18/2016	70.7	HEIGHT	6.85	12	0	0
10	2439	0	Placebo	0	24	3/17/2015	79.5	HEIGHT	9.1	13	0	0
10	2439	12	Placebo	0	37	6/18/2016	91	HEIGHT	11.8	14	0	0
10	2439	24	Placebo	0	51	5/25/2017	98.5	HEIGHT	13.25	13	1	0
10	2439	36	Placebo	0	63	5/30/2018	105.1	HEIGHT	13.85	13	0	0
10	2440	0	Placebo	1	48	3/17/2015	101.2	HEIGHT	16.55	16.5	1	0
10	2440	12	Placebo	1	56	6/17/2016	108.2	HEIGHT	18	16	1	0
10	2440	36	Placebo	1	82	5/30/2018	120.7	HEIGHT	22.4	17	0	0
10	2440	48	Placebo	1	93	5/18/2019	124.7	HEIGHT	23.25	16.5	0	0
10	2440	60	Placebo	1	102	2/26/2020	127.9	HEIGHT	27.86363636	17.5	0	0
10	2443	0	Placebo	1	28	6/17/2015	86.7	HEIGHT	11.1	14	1	0
10	2443	24	Placebo	1	56	5/25/2017	101.7	HEIGHT	14.85	14.5	1	0
10	2443	48	Placebo	1	79	5/18/2019	112.9	HEIGHT	17.75	15	0	0
10	2445	0	Placebo	1	36	3/17/2015	91.1	HEIGHT	12.4	15.5	1	0
10	2445	12	Placebo	1	49	6/17/2016	101.1	HEIGHT	14.15	15.5	0	0
10	2445	24	Placebo	1	63	5/25/2017	108.6	HEIGHT	16.1	15	0	0
10	2445	36	Placebo	1	75	5/31/2018	115.2	HEIGHT	16.95	15.5	0	0
10	2446	24	Placebo	0	26	5/26/2017	72.3	HEIGHT	8.2	13	1	0
10	2446	36	Placebo	0	38	5/30/2018	78.7	HEIGHT	9.8	13.5	1	0
10	2447	12	Placebo	0	7	6/17/2016	71.5	HEIGHT	7.8	14	1	0
10	2449	12	Placebo	0	6	6/17/2016	71.8	LENGTH	7.5	14	0	0
10	2449	24	Placebo	0	17	5/25/2017	81.1	HEIGHT	9.1	13	0	0
10	2449	36	Placebo	0	29	5/31/2018	91	HEIGHT	10.95	12.5	1	0
10	2449	48	Placebo	0	40	5/18/2019	97.6	HEIGHT	12.7	13	1	0
10	2449	60	Placebo	0	49	2/26/2020	104.1	HEIGHT	14.81818182	13.7	1	0
10	2454	24	Placebo	0	13	5/26/2017	73.9	HEIGHT	7.6	12	1	0
10	2454	36	Placebo	0	25	6/13/2018	83.4	HEIGHT	9.818181818	14	1	0
10	2454	60	Placebo	0	45	3/21/2020	97.7	HEIGHT	12.9	14.3	1	0
10	2455	0	Placebo	1	22	3/17/2015	79.2	HEIGHT	8.65	13.5	1	0
10	2456	24	Placebo	0	8	5/25/2017	58.3	LENGTH	4.9	12	1	0
10	2456	36	Placebo	0	15	5/30/2018	71	LENGTH	6.05	11.5	0	0
10	2457	0	Placebo	0	2	3/17/2015	66.8	LENGTH	7.05	13.5	0	0
10	2457	12	Placebo	0	30	6/17/2016	79.4	LENGTH	10.2	15	0	0
10	2457	36	Placebo	0	48	5/30/2018	91.5	HEIGHT	13	14	0	0
10	2457	48	Placebo	0	59	5/18/2019	98.7	HEIGHT	14.85	14	1	0
10	2460	0	Placebo	1	48	6/17/2015	106.3	HEIGHT	14.25	14	1	0
10	2460	12	Placebo	1	66	6/17/2016	112.7	HEIGHT	15.55	14.5	0	0
10	2460	24	Placebo	1	80	5/25/2017	120.5	HEIGHT	16.75	13.5	0	0
10	2461	0	Placebo	0	54	3/17/2015	94.2	HEIGHT	11.05	13	0	0
10	2461	12	Placebo	0	56	6/17/2016	101.1	HEIGHT	12.3	13	1	0

10	2461	24	Placebo	0	70	5/25/2017	107.4	HEIGHT	13.4	12.5		0	0
10	2461	36	Placebo	0	82	5/30/2018	111.4	HEIGHT	13.95	12.5		0	0
10	2469	0	Placebo	0	5	3/17/2015	63.5	LENGTH	6.45	12.5		0	0
10	2469	12	Placebo	0	14	6/17/2016	77.8	HEIGHT	8.6	13.5		0	0
10	2469	24	Placebo	0	28	5/25/2017	85.2	HEIGHT	10.35	14		0	0
10	2469	36	Placebo	0	40	5/31/2018	94.9	HEIGHT	11.85	14		1	0
10	2470	0	Placebo	1	40	3/17/2015	84.7	HEIGHT	11.25	13.5		1	0
10	2470	12	Placebo	1	50	6/17/2016	100.7	HEIGHT	12.2	13.5		1	0
10	2470	36	Placebo	1	72	5/30/2018	109.5	HEIGHT	14.5	13.5		0	0
10	2471	0	Placebo	0	48	6/8/2015	103.1	HEIGHT	15.4	15		1	0
10	2471	48	Placebo	0	97	5/18/2019	116.9	HEIGHT	17.15	14		0	0
10	2472	0	Placebo	1	36	3/17/2015	89.9	HEIGHT	11.55	14.5		0	0
10	2473	12	Placebo	1	12	6/17/2016	71	HEIGHT	6.7	13		1	0
10	2473	24	Placebo	1	26	5/25/2017	80.7	HEIGHT	9.1	13		0	0
10	2473	36	Placebo	1	38	5/31/2018	89.4	HEIGHT	10.1	12.5		1	0
10	2474	24	Placebo	1	18	5/25/2017	80.3	HEIGHT	8.75	13		1	0
10	2477	12	Placebo	0	8	6/18/2016	65.7	LENGTH	6.6	13		1	0
10	2477	24	Placebo	0	12	5/25/2017	73	HEIGHT	7.55	12		0	0
10	2477	36	Placebo	0	24	5/30/2018	80.8	HEIGHT	10.05	14		1	0
10	2477	48	Placebo	0	34	5/18/2019	88.3	HEIGHT	11.25	13		1	0
10	2478	12	Placebo	1	2	6/17/2016	63.1	LENGTH	6.85	14		0	0
10	2478	24	Placebo	1	12	5/25/2017	76.6	LENGTH	9.1	14		0	0
10	2478	36	Placebo	1	24	5/30/2018	86.8	HEIGHT	11.2	15		1	0
10	2478	48	Placebo	1	34	5/18/2019	92.9	HEIGHT	11.9	14		1	0
10	2480	0	Placebo	0	36	3/17/2015	78.7	HEIGHT	7.85	12.5		0	0
10	2481	12	Placebo	1	8	6/17/2016	75.3	LENGTH	8.8	15		1	0
10	2481	24	Placebo	1	17	5/25/2017	85.6	HEIGHT	10.65	15		1	0
10	2481	36	Placebo	1	29	5/30/2018	95.3	HEIGHT	13.45	16		1	0
10	2481	48	Placebo	1	40	6/10/2019	103.1	HEIGHT	14.81818182	15.5		1	0
10	2482	0	Placebo	1	12	3/17/2015	69.9	HEIGHT	6.4	12	60	1	0
10	2482	12	Placebo	0	18	6/17/2016	81	HEIGHT	9	13.5	60	1	0
10	2483	0	Placebo	0	36	3/17/2015	90.6	HEIGHT	11.2	14		0	0
10	2483	12	Placebo	0	44	6/17/2016	99.4	HEIGHT	12.8	14		0	0
10	2483	24	Placebo	0	58	5/25/2017	106.1	HEIGHT	14.65	14		1	0
10	2483	36	Placebo	0	70	5/30/2018	111.1	HEIGHT	15.9	14		0	0
10	2483	48	Placebo	0	81	5/18/2019	114.7	HEIGHT	16.8	14		0	0
10	2485	0	Placebo	1	12	3/17/2015	67.1	LENGTH	7	14		0	0
10	2487	0	Placebo	1	36	3/17/2015	90.4	HEIGHT	11.85	15		0	0
10	2487	12	Placebo	1	44	6/17/2016	98.5	HEIGHT	13	15		1	0
10	2487	24	Placebo	1	58	5/25/2017	104.8	HEIGHT	15.15	15.5		1	0

10	2487	60	Placebo	1	90	3/21/2020	120.2	HEIGHT	19.95454545	15.8	0	0
10	2488	0	Placebo	1	36	3/17/2015	91.3	HEIGHT	12.85	15.5	0	0
10	2489	0	Placebo	0	36	3/17/2015	83.1	HEIGHT	9.1	14	0	0
10	2489	12	Placebo	0	42	6/17/2016	89.7	HEIGHT	9.9	12.5	0	0
10	2490	12	Placebo	0	8	6/17/2016	67	LENGTH	6.1	12	0	0
10	2490	24	Placebo	0	16	5/26/2017	72.9	HEIGHT	7.15	11	1	0
10	2490	36	Placebo	0	28	5/30/2018	77.8	HEIGHT	7.7	10	1	0
10	2490	48	Placebo	0	39	5/18/2019	83.5	LENGTH	11.25	13	1	0
10	2492	0	Placebo	0	18	3/17/2015	52.5	LENGTH	3.9	11	0	0
10	2492	12	Placebo	0	26	6/17/2016	75.1	HEIGHT	9.45	15.5	0	0
10	2492	24	Placebo	0	28	5/25/2017	83.2	HEIGHT	10.95	13.5	1	0
10	2492	36	Placebo	0	40	5/31/2018	93.5	HEIGHT	13.8	16	0	0
10	2492	48	Placebo	0	51	6/25/2019	100.3	HEIGHT	16.27272727	16	0	0
10	2494	0	Placebo	0	36	3/17/2015	89	HEIGHT	12.02	15	0	0
10	2494	12	Placebo	0	42	6/17/2016	100.5	HEIGHT	15	15	0	0
10	2494	24	Placebo	0	56	5/25/2017	109.9	HEIGHT	16.95	14.5	0	0
10	2494	36	Placebo	0	68	5/30/2018	116.4	HEIGHT	19.25	15	0	0
10	2494	48	Placebo	0	79	5/18/2019	120.9	HEIGHT	20.45	14	0	0
10	2497	0	Placebo	1	3	3/17/2015	55.9	LENGTH	4.95	13	1	0
10	2497	12	Placebo	1	14	6/17/2016	71.8	HEIGHT	7.55	13.5	0	0
10	2497	36	Placebo	1	42	5/31/2018	86.2	HEIGHT	10.5	14	0	0
10	2502	0	Placebo	1	6	3/17/2015	64.3	LENGTH	6.15	13.5	1	0
10	2502	12	Placebo	1	14	6/17/2016	78.3	LENGTH	8.9	14.5	0	0
10	2502	24	Placebo	1	28	5/25/2017	84.9	HEIGHT	10.5	14.5	0	0
10	2502	36	Placebo	1	40	5/30/2018	93.4	HEIGHT	12.55	15	1	0
10	2504	0	Placebo	1	12	3/17/2015	71.5	LENGTH	7.95	13.5	1	0
10	2504	12	Placebo	1	20	6/17/2016	79.1	HEIGHT	8.9	13	0	0
10	2504	24	Placebo	1	34	5/26/2017	87.2	HEIGHT	13	16	1	0
10	2504	36	Placebo	1	46	5/30/2018	96.5	HEIGHT	13.9	15	1	0
10	2508	0	Placebo	0	48	3/17/2015	91.8	HEIGHT	12	14.5	1	0
10	2508	24	Placebo	0	70	5/25/2017	105.2	HEIGHT	14.15	14	0	0
10	2508	36	Placebo	0	82	5/30/2018	111	HEIGHT	15.55	15	0	0
10	2509	0	Placebo	1	36	3/17/2015	81.4	HEIGHT	9.5	13.5	1	0
10	2509	12	Placebo	1	44	6/17/2016	87.3	HEIGHT	11.35	15	0	0
10	2509	24	Placebo	1	58	5/25/2017	94	HEIGHT	12.5	14.5	1	0
10	2509	36	Placebo	1	70	5/30/2018	101.5	HEIGHT	13.95	14.5	0	0
10	2509	48	Placebo	1	81	5/18/2019	106.8	HEIGHT	15.1	14.5	0	0
10	2509	60	Placebo	1	90	3/21/2020	110.6	HEIGHT	16.09090909	14	0	0
10	2510	12	Placebo	0	47	6/18/2016	132	HEIGHT	24.45	16	1	0
10	2515	24	Placebo	0	58	5/25/2017	110.5	HEIGHT	15.8	14	0	0

10	2515	48	Placebo	0	81	5/18/2019	121.1	HEIGHT	20.65	15	0	0
10	2516	12	Placebo	0	5	6/18/2016	65	LENGTH	5.9	12	0	0
10	2516	24	Placebo	0	17	5/25/2017	75.9	HEIGHT	7.75	12.5	1	0
10	2516	36	Placebo	0	29	5/30/2018	84.3	HEIGHT	9.55	13	1	0
10	2520	0	Placebo	0	2	3/17/2015	59.6	LENGTH	5.85	13.5	0	0
10	2520	12	Placebo	0	18	6/17/2016	77.1	HEIGHT	9.6	15	1	0
10	2520	24	Placebo	0	32	5/25/2017	86.9	HEIGHT	12.4	15.5	0	0
10	2520	36	Placebo	0	44	5/31/2018	94.4	HEIGHT	13.45	15	1	0
10	2521	0	Placebo	1	36	6/17/2015	89.9	HEIGHT	11.65	14	1	0
10	2521	24	Placebo	1	58	5/25/2017	105.2	HEIGHT	14.1	13.5	1	0
10	2523	0	Placebo	0	18	3/17/2015	87	HEIGHT	10.9	15	0	0
10	2523	12	Placebo	0	33	6/17/2016	94.6	HEIGHT	13.5	15	0	0
10	2524	12	Placebo	1	12	6/17/2016	65.5	LENGTH	5.55	11.5	1	0
10	2527	0	Placebo	1	36	3/17/2015	93.7	HEIGHT	13	15	0	0
10	2527	12	Placebo	1	49	6/17/2016	105.3	HEIGHT	15.15	15	1	0
10	2527	24	Placebo	1	63	5/25/2017	114.2	HEIGHT	16.95	15	0	0
10	2527	36	Placebo	1	75	5/30/2018	119.9	HEIGHT	17.9	14.5	0	0
10	2527	48	Placebo	1	86	5/18/2019	126.7	HEIGHT	20	14.5	0	0
10	2527	60	Placebo	1	95	2/26/2020	130.8	HEIGHT	22.68181818	15.4	0	0
10	2530	0	Placebo	0	54	3/17/2015	109.5	HEIGHT	17.35	16	0	0
10	2530	36	Placebo	0	133	5/31/2018	126.4	HEIGHT	23.6	17	0	0
10	2531	0	Placebo	0	36	3/17/2015	91	HEIGHT	12.45	15	0	0
10	2531	12	Placebo	0	57	6/18/2016	99.9	HEIGHT	13.4	14.5	1	0
10	2531	24	Placebo	0	68	5/25/2017	105.2	HEIGHT	14.8	14	0	0
10	2531	36	Placebo	0	80	5/30/2018	110.7	HEIGHT	16.35	14	0	0
10	2531	48	Placebo	0	91	5/18/2019	114.6	HEIGHT	17.4	14	0	0
10	2534	0	Placebo	1	48	3/17/2015	104.5	HEIGHT	13.95	14	0	0
10	2534	12	Placebo	1	72	6/17/2016	111.1	HEIGHT	16.1	15	0	0
10	2535	0	Placebo	0	8	3/17/2015	69	LENGTH	6.95	13.5	0	0
10	2535	12	Placebo	0	20	6/17/2016	84.3	LENGTH	10.45	15	0	0
10	2535	24	Placebo	0	34	5/25/2017	92.5	HEIGHT	12.65	14.5	1	0
10	2535	36	Placebo	0	46	5/30/2018	99.3	HEIGHT	14.15	15	1	0
10	2540	24	Placebo	0	10	5/25/2017	66.5	LENGTH	5.85	11	1	0
10	2540	36	Placebo	0	21	5/30/2018	78.8	LENGTH	8	12.5	1	0
10	2544	0	Placebo	0	12	3/17/2015	71.6	LENGTH	8	14	1	0
10	2544	12	Placebo	0	33	6/17/2016	82.7	HEIGHT	10.85	15	0	0
10	2544	24	Placebo	0	44	5/25/2017	90.8	HEIGHT	12.85	14	1	0
10	2544	36	Placebo	0	56	5/30/2018	99.3	HEIGHT	14.45	14.5	1	0
10	2545	0	Placebo	1	36	3/17/2015	83.2	HEIGHT	10.9	14.5	1	0
10	2545	12	Placebo	1	44	6/17/2016	94.2	HEIGHT	13.3	15.5	0	0

10	2545	24	Placebo	1	58	5/25/2017	100.4	HEIGHT	14.25	15.5		1	0
10	2545	36	Placebo	1	70	5/30/2018	107.7	HEIGHT	15.75	16		0	0
10	2547	0	Placebo	1	48	3/17/2015	98.5	HEIGHT	13.65	15		1	0
10	2547	12	Placebo	1	45	6/17/2016	108.7	HEIGHT	16.25	16		0	0
10	2547	24	Placebo	1	56	5/25/2017	116.2	HEIGHT	17.5	15		0	0
10	2547	36	Placebo	1	68	5/30/2018	121.9	HEIGHT	18.9	14.5		0	0
10	2548	12	Placebo	0	20	6/17/2016	83.6	HEIGHT	10.35	14.5	18	1	0
10	2550	0	Placebo	0	5	3/17/2015	70.1	LENGTH	8.35	14.5		0	0
10	2550	12	Placebo	0	14	6/18/2016	81.5	HEIGHT	9.6	13.5		0	0
10	2550	24	Placebo	0	28	5/25/2017	87.9	HEIGHT	12.8	15		1	0
10	2550	48	Placebo	0	51	6/10/2019	104.6	HEIGHT	16.77272727	16		1	0
10	2551	0	Placebo	1	18	3/17/2015	82.8	HEIGHT	9.85	15.5		1	0
10	2551	12	Placebo	1	42	6/17/2016	92.7	HEIGHT	12	15.5		0	0
10	2551	24	Placebo	1	56	5/25/2017	100.4	HEIGHT	13.35	14.5		1	0
10	2552	0	Placebo	0	36	3/17/2015	100.5	HEIGHT	13.7	14		1	0
10	2552	12	Placebo	0	68	6/17/2016	107.9	HEIGHT	15.1	14		0	0
10	2552	24	Placebo	0	82	5/26/2017	113.4	HEIGHT	16.3	13		0	0
10	2552	60	Placebo	0	114	3/21/2020	126.4	HEIGHT	20.68181818	14.5		0	0
10	2553	0	Placebo	0	24	3/17/2015	79.6	HEIGHT	8.35	14		0	0
10	2553	12	Placebo	0	38	6/17/2016	91.9	HEIGHT	10.95	13.5		0	0
10	2553	24	Placebo	0	52	5/25/2017	100.2	HEIGHT	12.6	13.5		0	0
10	2553	36	Placebo	0	64	5/31/2018	108.6	HEIGHT	13.8	14		0	0
10	2554	12	Placebo	1	9	6/17/2016	69.1	HEIGHT	7.1	14		0	0
10	2554	24	Placebo	1	23	5/25/2017	78.7	HEIGHT	9.1	14		1	0
10	2554	60	Placebo	1	55	2/26/2020	101.6	HEIGHT	13.59090909	13.2		0	0
10	2555	0	Placebo	1	48	3/17/2015	105.7	HEIGHT	14.35	14.5		0	0
10	2555	36	Placebo	1	98	5/30/2018	124.9	HEIGHT	19	14.5		0	0
10	2555	48	Placebo	1	109	5/18/2019	129.2	HEIGHT	21.25	15		0	0
10	2555	60	Placebo	1	118	2/26/2020	134.3	HEIGHT	23.27272727	15.2		0	0
10	2556	0	Placebo	1	24	3/17/2015	85.4	HEIGHT	11.35	15.5	6	1	0
10	2558	0	Placebo	0	24	3/17/2015	74.5	LENGTH	8.45	13.5		1	0
10	2558	12	Placebo	0	38	6/17/2016	83.3	HEIGHT	10.65	14.5		1	0
10	2559	0	Placebo	0	12	6/17/2015	76.5	LENGTH	8.2	13.5		1	0
10	2559	36	Placebo	0	46	5/31/2018	98.2	HEIGHT	12.45	13		1	0
10	2559	48	Placebo	0	57	6/25/2019	105.3	HEIGHT	13.72727273	13.7		1	0
10	2561	24	Placebo	0	23	5/25/2017	80.8	HEIGHT	8.4	12		1	0
10	2561	36	Placebo	0	35	6/13/2018	91.5	HEIGHT	10.72727273	14		1	0
10	2561	48	Placebo	0	46	5/18/2019	97.3	HEIGHT	11.6	12.5		1	0
10	2566	0	Placebo	0	24	3/17/2015	79.3	LENGTH	8.35	12		1	0
10	2566	12	Placebo	0	29	6/17/2016	84.4	HEIGHT	10.05	12.5		1	0

10	2566	24	Placebo	0	40	5/25/2017	91.8	HEIGHT	11.95	12.5	0	0
10	2566	36	Placebo	0	52	5/30/2018	96.3	HEIGHT	12.85	12	0	0
10	2566	48	Placebo	0	63	5/18/2019	104.2	HEIGHT	14.85	13	0	0
10	2567	12	Placebo	1	32	6/17/2016	92.9	HEIGHT	11.1	13	1	0
10	2567	36	Placebo	1	61	5/31/2018	106.3	HEIGHT	13.8	13.5	0	0
10	2569	0	Placebo	0	36	3/17/2015	84.5	HEIGHT	10.1	13	1	0
10	2569	12	Placebo	0	44	6/17/2016	92.1	HEIGHT	11.6	13.5	0	0
10	2569	36	Placebo	0	70	5/31/2018	104.2	HEIGHT	14.3	13	0	0
10	2571	0	Placebo	1	18	3/17/2015	79	HEIGHT	8.35	13.5	0	0
10	2573	0	Placebo	1	8	3/17/2015	66.2	LENGTH	7.65	14	1	0
10	2573	12	Placebo	1	20	6/17/2016	83	HEIGHT	11.2	17	0	0
10	2573	24	Placebo	1	34	5/26/2017	89.9	HEIGHT	13.25	15.5	1	0
10	2573	60	Placebo	1	66	3/21/2020	113	HEIGHT	18.55	15.6	0	0
10	2574	0	Placebo	1	54	3/17/2015	107.4	HEIGHT	14.7	14	1	0
10	2574	12	Placebo	1	66	6/17/2016	114.8	HEIGHT	16.9	14.5	0	0
10	2574	24	Placebo	1	80	5/25/2017	118.8	HEIGHT	17.8	14	0	0
10	2574	36	Placebo	1	92	5/30/2018	124.2	HEIGHT	18.95	14.5	0	0
10	2574	48	Placebo	1	103	6/25/2019	128.1	HEIGHT	21	13.5	0	0
10	2574	60	Placebo	1	112	2/26/2020	131.9	HEIGHT	22.81818182	14.6	0	0
10	2575	0	Placebo	0	36	3/17/2015	92.3	HEIGHT	13.95	15.5	1	0
10	2575	12	Placebo	0	44	6/17/2016	105	HEIGHT	14.05	15	1	0
10	2576	0	Placebo	0	54	3/17/2015	102.4	HEIGHT	16.1	15.5	0	0
10	2576	12	Placebo	0	67	6/17/2016	109	HEIGHT	17.7	16.5	0	0
10	2576	36	Placebo	0	93	5/30/2018	118.5	HEIGHT	20.05	15.5	0	0
10	2576	48	Placebo	0	104	6/25/2019	123.4	HEIGHT	24.09090909	16.5	0	0
10	2576	60	Placebo	0	113	3/21/2020	127.1	HEIGHT	26.68181818	17.9	0	0
10	2577	0	Placebo	1	24	3/17/2015	74.5	LENGTH	7.4	11.5	1	0
10	2577	12	Placebo	1	32	6/17/2016	85.4	HEIGHT	10.45	14	0	0
10	2577	36	Placebo	1	58	5/30/2018	100.7	HEIGHT	13.15	13.5	0	0
10	2577	48	Placebo	1	69	5/18/2019	106.7	HEIGHT	15.2	14	0	0
10	2577	60	Placebo	1	78	2/26/2020	112.1	HEIGHT	16.77272727	13.5	0	0
10	2578	0	Placebo	1	36	3/17/2015	89.5	HEIGHT	13.15	15.5	0	0
10	2578	12	Placebo	1	44	6/17/2016	98.6	HEIGHT	14.1	16	0	0
10	2578	24	Placebo	1	58	5/25/2017	105.5	HEIGHT	15.15	15	1	0
10	2578	48	Placebo	1	81	6/10/2019	116.7	HEIGHT	16.86363636	15.5	0	0
10	2582	0	Placebo	0	48	3/17/2015	86.3	HEIGHT	13.05	15.5	0	0
10	2582	12	Placebo	0	48	6/17/2016	94.4	HEIGHT	13.65	15	1	0
10	2582	36	Placebo	0	76	5/30/2018	105.7	HEIGHT	16.15	14	0	0
10	2582	48	Placebo	0	87	5/18/2019	110	HEIGHT	17.9	14.5	0	0
10	2582	60	Placebo	0	97	2/26/2020	114.9	HEIGHT	19.63636364	14.5	0	0

10	2583	12	Placebo	0	41	6/17/2016	90.3	HEIGHT	12.1	14		1	0
10	2584	0	Placebo	1	48	6/17/2015	101.2	HEIGHT	13.9	14.5		1	0
10	2584	12	Placebo	1	61	6/18/2016	108.4	HEIGHT	15.8	14		0	0
10	2584	24	Placebo	1	75	5/26/2017	115	HEIGHT	17.5	14.5		0	0
10	2585	24	Placebo	0	28	5/25/2017	78.9	HEIGHT	10.5	14		1	0
10	2585	48	Placebo	0	51	6/10/2019	95.6	HEIGHT	18.59090909	19.5		1	0
10	2589	0	Placebo	0	2	3/17/2015	57.6	LENGTH	6.25	13.5		0	0
10	2589	12	Placebo	0	14	6/17/2016	81.4	HEIGHT	10.7	15.5		1	0
10	2589	24	Placebo	0	28	5/26/2017	88.3	HEIGHT	13.1	15		0	0
10	2590	12	Placebo	0	1	6/17/2016	62.5	LENGTH	5.7	12.5		0	0
10	2591	0	Placebo	1	48	6/17/2015	107.3	HEIGHT	14.2	13.5		1	0
10	2591	24	Placebo	1	70	5/25/2017	114.2	HEIGHT	17	14.5		0	0
10	2591	60	Placebo	1	102	2/26/2020	126.5	HEIGHT	22.5	15.5		0	0
10	2593	0	Placebo	0	36	3/17/2015	90.3	LENGTH	12.1	15		1	0
10	2593	12	Placebo	0	44	6/17/2016	102.5	HEIGHT	13.85	15.5		0	0
10	2593	24	Placebo	0	58	5/25/2017	107.8	HEIGHT	17.25	16		1	0
10	2593	48	Placebo	0	81	6/10/2019	121.2	HEIGHT	20.54545455	17.4		0	0
10	2595	0	Placebo	0	5	3/17/2015	64.3	LENGTH	5.8	12		0	0
10	2595	24	Placebo	0	32	5/25/2017	82.7	HEIGHT	9.65	12.5		0	0
10	2595	36	Placebo	0	44	5/30/2018	94.4	HEIGHT	12.15	13.5		0	0
10	2595	48	Placebo	0	55	5/18/2019	102.4	HEIGHT	13.85	13.5		1	0
10	2597	0	Placebo	0	54	3/17/2015	95	HEIGHT	13.15	14		0	0
10	2597	12	Placebo	0	66	6/17/2016	102.9	HEIGHT	13.8	14		0	0
10	2597	24	Placebo	0	80	5/25/2017	109.3	HEIGHT	15.9	13.5		0	0
10	2597	48	Placebo	0	103	5/18/2019	120.3	HEIGHT	19.4	14		0	0
10	2597	60	Placebo	0	112	3/21/2020	123.1	HEIGHT	21.09090909	14		0	0
10	2598	0	Placebo	1	24	3/17/2015	77.1	HEIGHT	8	13		1	0
10	2598	12	Placebo	1	32	6/17/2016	85.3	HEIGHT	10.35	14		1	0
10	2598	24	Placebo	1	46	5/26/2017	92.5	HEIGHT	11.3	13		1	0
10	2603	12	Placebo	1	30	6/17/2016	82	HEIGHT	8.95	11.5	30	1	0
10	2603	24	Placebo	1	44	5/25/2017	91.5	HEIGHT	12.05	14	30	1	0
10	2604	0	Placebo	1	36	6/8/2015	84.4	HEIGHT	10.85	14.5		1	0
10	2604	12	Placebo	1	51	6/17/2016	89.1	HEIGHT	11.45	14		0	0
10	2604	24	Placebo	1	63	5/26/2017	96	HEIGHT	13.5	13		0	0
10	2605	0	Placebo	0	36	3/17/2015	96.6	HEIGHT	12.9	15		1	0
10	2605	12	Placebo	0	44	6/18/2016	105.5	HEIGHT	14.75	15		1	0
10	2605	24	Placebo	0	58	5/25/2017	109.9	HEIGHT	15	14		0	0
10	2605	36	Placebo	0	70	5/30/2018	116.4	HEIGHT	17.95	14.5		0	0
10	2606	0	Placebo	0	36	3/17/2015	92.9	HEIGHT	13.75	16.5		0	0
10	2606	12	Placebo	0	9	6/17/2016	102	HEIGHT	15.3	16.5		1	0

10	2606	24	Placebo	0	58	5/25/2017	108.6	HEIGHT	16.5	15.5		0	0
10	2606	48	Placebo	0	81	6/10/2019	124.3	HEIGHT	20.68181818	16		0	0
10	2607	0	Placebo	0	48	3/17/2015	91.3	HEIGHT	12.65	14.5		0	0
10	2607	12	Placebo	0	56	6/17/2016	99.9	HEIGHT	15.05	15.5		0	0
10	2607	24	Placebo	0	70	5/25/2017	106.1	HEIGHT	16.1	15		0	0
10	2607	36	Placebo	0	82	5/30/2018	110.9	HEIGHT	17.5	16		0	0
10	2607	60	Placebo	0	102	3/21/2020	120	HEIGHT	20.5	15		0	0
10	2608	0	Placebo	1	48	3/17/2015	91.4	HEIGHT	11.8	13.5		0	0
10	2608	36	Placebo	1	82	5/31/2018	110.5	HEIGHT	15.3	13		0	0
10	2608	48	Placebo	1	93	6/25/2019	114.5	HEIGHT	17.04545455	13.7		0	0
10	2613	0	Placebo	1	18	3/17/2015	78	HEIGHT	9.25	14		1	0
10	2613	12	Placebo	1	26	6/18/2016	87.2	HEIGHT	11.9	15.5		0	0
10	2614	0	Placebo	1	36	3/17/2015	85	HEIGHT	9.3	13.5		0	0
10	2614	12	Placebo	1	51	6/18/2016	97.2	HEIGHT	11.6	14.5		0	0
10	2614	24	Placebo	1	63	5/25/2017	104.5	HEIGHT	12.5	13		0	0
10	2614	48	Placebo	1	86	5/18/2019	118.5	HEIGHT	15	12.5		0	0
10	2615	0	Placebo	1	24	3/17/2015	76.3	LENGTH	8.4	13		1	0
10	2615	12	Placebo	1	32	6/17/2016	88.2	HEIGHT	10.7	15		1	0
10	2615	24	Placebo	1	46	5/25/2017	94.2	HEIGHT	12.7	14.5		0	0
10	2615	36	Placebo	1	58	5/30/2018	101.4	HEIGHT	14.3	15		1	0
10	2615	60	Placebo	1	78	3/21/2020	111.9	HEIGHT	18.09090909	15.3		0	0
10	2619	12	Placebo	0	44	6/17/2016	86.6	HEIGHT	10.7	13		1	0
10	2619	24	Placebo	0	53	5/26/2017	93	HEIGHT	11.6	12.5		1	0
10	2619	36	Placebo	0	65	5/30/2018	100	HEIGHT	13.25	13.5		0	0
10	2620	12	Placebo	1	7	6/17/2016	67.7	HEIGHT	6.45	12.5		1	0
10	2620	24	Placebo	1	21	5/25/2017	77.3	HEIGHT	7.5	11.5		1	0
10	2620	36	Placebo	1	33	5/30/2018	85.4	HEIGHT	9.3	12		1	0
10	2620	48	Placebo	1	44	5/18/2019	92.5	HEIGHT	10.55	13		0	0
10	2620	60	Placebo	1	53	3/21/2020	99.6	HEIGHT	12.27272727	12.9		1	0
10	2621	12	Placebo	1	14	6/17/2016	74.7	HEIGHT	8.9	14.5		1	0
10	2621	24	Placebo	1	28	5/25/2017	83.9	HEIGHT	11.2	14.5		1	0
10	2621	36	Placebo	1	40	6/13/2018	92.2	HEIGHT	12.59090909	16		1	0
10	2621	48	Placebo	1	51	5/18/2019	96.9	HEIGHT	14.05	15		1	0
10	2621	60	Placebo	1	60	3/21/2020	102.2	HEIGHT	15.72727273	15		0	0
10	2622	0	Placebo	0	3	6/17/2015	62.7	LENGTH	5.55	12	42	1	0
10	2622	12	Placebo	0	14	6/17/2016	71.9	HEIGHT	7.2	13	42	1	0
10	2622	24	Placebo	0	28	5/25/2017	78.4	HEIGHT	8.65	12	42	0	0
10	2623	0	Placebo	1	48	3/17/2015	106.3	HEIGHT	14.3	14		1	0
10	2623	12	Placebo	1	56	6/17/2016	114.9	HEIGHT	16.7	14		1	0
10	2623	24	Placebo	1	70	5/25/2017	121.5	HEIGHT	18.55	14.5		0	0

10	2624	0	Placebo	0	12	3/17/2015	73	LENGTH	7.85	14		0	0
10	2624	24	Placebo	0	34	5/25/2017	93.9	HEIGHT	12.6	14.5		1	0
10	2625	24	Placebo	0	58	5/25/2017	98.8	HEIGHT	12.25	12.5		1	0
10	2625	60	Placebo	0	90	3/21/2020	116.4	HEIGHT	17.59090909	13.2		0	0
10	2626	0	Placebo	0	24	3/17/2015	80	HEIGHT	8.7	13		1	0
10	2626	24	Placebo	0	45	5/25/2017	101.5	HEIGHT	13.4	14		0	0
10	2626	36	Placebo	0	57	5/31/2018	109.2	HEIGHT	15.3	14		1	0
10	2627	0	Placebo	0	12	6/17/2015	71.6	LENGTH	7.75	14		1	0
10	2627	12	Placebo	0	20	6/17/2016	78.9	HEIGHT	8.45	14		1	0
10	2627	24	Placebo	0	34	5/25/2017	87.6	HEIGHT	10.7	14.5		1	0
10	2627	36	Placebo	0	46	5/30/2018	95.2	HEIGHT	12.25	14		1	0
10	2627	60	Placebo	0	66	3/21/2020	106.9	HEIGHT	14.72727273	13.5		0	0
10	2628	0	Placebo	1	24	3/17/2015	79	HEIGHT	8.9	13		1	0
10	2630	0	Placebo	1	12	3/17/2015	71.6	LENGTH	8.1	14		0	0
10	2630	12	Placebo	1	27	6/18/2016	86.2	LENGTH	10.45	15		1	0
10	2631	0	Placebo	1	30	3/17/2015	83.4	HEIGHT	11.65	15.5		0	0
10	2631	12	Placebo	1	43	6/18/2016	91.4	HEIGHT	12.9	16		0	0
10	2631	36	Placebo	1	69	5/30/2018	105.6	HEIGHT	16.2	16		0	0
10	2631	48	Placebo	1	80	6/25/2019	111.3	HEIGHT	18.68181818	15.5		0	0
10	2631	60	Placebo	1	89	3/21/2020	117	HEIGHT	20.90909091	16.4		0	0
10	2632	24	Placebo	0	23	5/26/2017	83.7	HEIGHT	12.05	15.5		1	0
10	2633	12	Placebo	1	8	6/17/2016	71.3	LENGTH	6.1	12	24	1	0
10	2634	0	Placebo	1	54	3/17/2015	100.8	HEIGHT	14.3	15	12	1	0
10	2637	0	Placebo	1	36	3/17/2015	86.6	HEIGHT	9.35	13.5		0	0
10	2637	12	Placebo	1	48	6/17/2016	94.8	HEIGHT	11.05	13.5		1	0
10	2637	24	Placebo	1	62	5/25/2017	101	HEIGHT	11.65	13		0	0
10	2637	36	Placebo	1	73	5/30/2018	108.3	HEIGHT	13.35	13		0	0
10	2637	48	Placebo	1	84	5/18/2019	114.6	HEIGHT	15.7	13.5		0	0
10	2638	0	Placebo	1	12	3/17/2015	76.4	HEIGHT	9.25	14.5		0	0
10	2638	12	Placebo	1	25	6/18/2016	87.7	HEIGHT	12.9	16		0	0
10	2638	24	Placebo	1	39	5/26/2017	94.1	HEIGHT	13.55	14.5		1	0
10	2639	0	Placebo	1	36	3/17/2015	85.2	HEIGHT	10.5	14		0	0
10	2639	12	Placebo	1	42	6/17/2016	94.5	HEIGHT	12.8	15		1	0
10	2639	24	Placebo	1	56	5/25/2017	101.2	HEIGHT	14.45	14.5		1	0
10	2639	48	Placebo	1	79	6/25/2019	116.9	HEIGHT	18.13636364	14.5		0	0
10	2639	60	Placebo	1	88	2/26/2020	122.2	HEIGHT	19.63636364	14.8		0	0
10	2648	12	Placebo	0	12	6/17/2016	68.8	LENGTH	5.4	10.5	48	1	0
10	2649	0	Placebo	1	24	6/8/2015	79.6	HEIGHT	8.95	14		1	0
10	2649	12	Placebo	1	37	6/17/2016	88.1	HEIGHT	11.2	14		0	0
10	2649	48	Placebo	1	74	5/18/2019	106.4	HEIGHT	15.05	14		0	0

10	2649	60	Placebo	1	83	2/26/2020	110.8	HEIGHT	16.18181818	13.6		0	0
10	2650	0	Placebo	1	1	3/17/2015	55.3	LENGTH	5.15	13.5		0	0
10	2650	12	Placebo	1	12	6/18/2016	77.1	HEIGHT	9.95	15		0	0
10	2650	48	Placebo	1	49	6/25/2019	98.9	HEIGHT	14.59090909	14.5		0	0
10	2650	60	Placebo	1	58	3/21/2020	103.9	HEIGHT	16.13636364	15.3		1	0
10	8033	36	Placebo	0	6	5/31/2018	66.5	LENGTH	6.25	12		1	0
10	8033	48	Placebo	0	17	5/18/2019	79.4	LENGTH	8.95	13.5		1	0
10	8033	60	Placebo	0	26	2/26/2020	87.6	HEIGHT	11.18181818	13.7		0	0
10	8044	60	Placebo	0	36	2/26/2020	88.3	HEIGHT	12	14		1	0
10	8045	36	Placebo	1	24	5/30/2018	83.7	HEIGHT	8.9	13		1	0
10	8045	48	Placebo	1	34	5/18/2019	91	HEIGHT	10.5	13		1	0
10	8045	60	Placebo	1	44	2/26/2020	96.6	HEIGHT	11.40909091	13		0	0
10	8079	60	Placebo	0	21	2/26/2020	78.1	LENGTH	9.954545455	13.2		1	0
10	8171	36	Placebo	0	6	5/31/2018	65.4	LENGTH	7.3	13.5	42	1	0
10	8197	48	Placebo	1	28	6/10/2019	79.4	HEIGHT	8.909090909	13		1	0
10	8275	48	Placebo	1	16	5/18/2019	78.2	HEIGHT	8.6	13		1	0
10	8280	36	Placebo	1	11	5/31/2018	67.7	LENGTH	6.4	12		1	0
10	8360	48	Placebo	1	32	6/10/2019	90.6	LENGTH	11.86363636	15.5		1	0
10	8417	48	Placebo	1	28	6/25/2019	80.4	HEIGHT	8.409090909	12		1	0
10	8457	36	Placebo	0	24	5/31/2018	70.6	LENGTH	7.4	13		1	0
10	8572	60	Placebo	0	14	3/21/2020	73.1	HEIGHT	7.3	12.5		1	0
10	8810	48	Placebo	0	12	6/10/2019	78.5	LENGTH	8.5	15		1	0
10	8810	60	Placebo	0	21	3/21/2020	84.9	HEIGHT	11.27272727	14		0	0
10	8824	48	Placebo	1	4	5/18/2019	64.4	LENGTH	6.2	12		1	0
10	8878	36	Placebo	1	28	5/31/2018	84	HEIGHT	10.2	14		1	0
10	8923	36	Placebo	1	21	6/13/2018	77.6	HEIGHT	9.181818182	14		1	0
10	8923	48	Placebo	1	32	5/18/2019	83.3	HEIGHT	11	14		1	0
10	8967	60	Placebo	0	49	2/26/2020	97	HEIGHT	14.27272727	14.5		1	0
10	8985	36	Placebo	1	8	5/30/2018	72.5	LENGTH	8.3	15		1	0
10	8985	48	Placebo	1	16	5/18/2019	80.7	HEIGHT	10	14		0	0
10	8985	60	Placebo	1	25	2/26/2020	86.2	HEIGHT	11.95454545	14.1		0	0
10	9030	36	Placebo	0	15	6/13/2018	70.9	LENGTH	7.409090909	12.5		1	0
10	9047	36	Placebo	1	21	5/31/2018	78.8	LENGTH	8.55	12		1	0
10	9047	48	Placebo	1	32	6/25/2019	84.2	HEIGHT	9.590909091	12.5		1	0
10	9211	36	Placebo	0	46	5/30/2018	98.3	HEIGHT	14.55	16.5		1	0
10	9230	36	Placebo	0	3	6/13/2018	64.2	LENGTH	6.318181818	14		1	0
10	9230	48	Placebo	0	14	5/18/2019	75.1	LENGTH	8.65	13		0	0
10	9230	60	Placebo	0	23	2/26/2020	81.5	HEIGHT	10.09090909	13.5		1	0
10	9328	60	Placebo	0	12	3/21/2020	85.1	HEIGHT	11.27272727	13.3		1	0
10	9379	60	Placebo	1	10	2/26/2020	71.6	LENGTH	7.181818182	13		1	0

10	9412	60	Placebo	0	39	2/26/2020	97.1	HEIGHT	13	12.6	1	0
10	9442	36	Placebo	1	27	5/30/2018	80.1	LENGTH	8.35	13	1	0
10	9473	36	Placebo	1	9	5/30/2018	60.5	LENGTH	4.9	11	1	0
10	9535	48	Placebo	1	36	6/10/2019	94	HEIGHT	11.90909091	15	1	0
10	9535	60	Placebo	1	45	3/21/2020	100	HEIGHT	13.81818182	13.3	0	0
10	9559	60	Placebo	0	48	3/21/2020	100.4	HEIGHT	14.5	14	1	0
10	9573	36	Placebo	0	22	6/13/2018	79.9	HEIGHT	8.681818182	14	1	0
10	9573	48	Placebo	0	33	6/10/2019	88.2	HEIGHT	11.31818182	14.4	1	0
10	9573	60	Placebo	0	42	2/26/2020	94.6	HEIGHT	12.40909091	14.2	0	0
11	2666	12	Placebo	1	5	7/27/2016	72.9	LENGTH		18.1	1	0
11	2666	24	Placebo	1	19	5/22/2017	83.2	HEIGHT	10.3	13.5	0	0
11	2666	36	Placebo	1	31	6/10/2018	91.2	HEIGHT	11.65	13.5	0	0
11	2666	48	Placebo	1	42	5/15/2019	98.4	HEIGHT	13.9	14	1	0
11	2666	60	Placebo	1	51	2/14/2020	102.7	HEIGHT	15.05	14	0	0
11	2667	12	Placebo	1	48	7/27/2016	111	HEIGHT		17.9	1	0
11	2668	12	Placebo	0	11	7/27/2016	69.4	LENGTH		15.2	1	0
11	2668	36	Placebo	0	29	6/10/2018	84.8	HEIGHT	12.15	15	0	0
11	2668	48	Placebo	0	40	5/15/2019	91.8	HEIGHT	12.7	15	1	0
11	2668	60	Placebo	0	49	2/14/2020	98.6	HEIGHT	14.3	14.5	0	0
11	2669	0	Placebo	0	48	3/14/2015	110.5	HEIGHT	17.55	15.5	1	0
11	2669	12	Placebo	0	66	7/27/2016	119	HEIGHT		17.2	0	0
11	2669	24	Placebo	0	80	5/22/2017	124.9	HEIGHT	20.75	15.5	0	0
11	2669	36	Placebo	0	92	6/10/2018	130.3	HEIGHT	24.05	15	0	0
11	2669	60	Placebo	0	112	2/14/2020	139.6	HEIGHT	27.25	15.5	0	0
11	2670	0	Placebo	0	24	3/14/2015	80.9	HEIGHT	9.45	13	1	0
11	2670	12	Placebo	0	42	7/27/2016	91.6	HEIGHT		16.5	0	0
11	2670	24	Placebo	0	56	5/22/2017	96	HEIGHT	12.6	13.5	1	0
11	2670	36	Placebo	0	68	6/10/2018	102.9	HEIGHT	13.85	13	0	0
11	2670	60	Placebo	0	88	2/14/2020	111.4	HEIGHT	16.85	13	0	0
11	2671	0	Placebo	0	54	3/14/2015	105.3	HEIGHT	15.85	14.5	1	0
11	2671	12	Placebo	0	60	7/27/2016	112.3	HEIGHT		16.5	0	0
11	2671	24	Placebo	0	73	5/22/2017	116.2	HEIGHT	18.6	14.5	0	0
11	2671	48	Placebo	0	121	5/15/2019	123.7	HEIGHT	22.35	15	0	0
11	2673	0	Placebo	1	36	3/14/2015	110.9	HEIGHT	16.3	15	0	0
11	2673	12	Placebo	1	66	7/27/2016	120.2	HEIGHT		18.5	0	0
11	2673	48	Placebo	1	103	5/15/2019	134	HEIGHT	25.5	17	0	0
11	2674	0	Placebo	0	18	3/14/2015	78.5	HEIGHT	10.45	15	0	0
11	2674	24	Placebo	0	44	5/22/2017	95.3	HEIGHT	13.45	14.5	1	0
11	2674	36	Placebo	0	56	6/10/2018	103.6	HEIGHT	14.55	14	0	0
11	2674	48	Placebo	0	67	5/15/2019	109.3	HEIGHT	16.3	14	0	0

11	2675	0	Placebo	0	12	3/14/2015	71.8	LENGTH	8.7	15.5		1	0
11	2675	12	Placebo	0	21	7/27/2016	83.9	HEIGHT		17		1	0
11	2675	24	Placebo	0	34	5/22/2017	89.2	HEIGHT	12.3	15		0	0
11	2675	36	Placebo	0	47	6/10/2018	95.5	HEIGHT	13.6	14		0	0
11	2675	48	Placebo	0	58	5/15/2019	101	HEIGHT	14.75	14		0	0
11	2675	60	Placebo	0	67	2/14/2020	105.8	HEIGHT	15.5	14		0	0
11	2679	0	Placebo	0	2	3/14/2015	52.6	LENGTH	4.35	12	54	0	0
11	2679	48	Placebo	0	49	5/15/2019	97.4	HEIGHT	13.3	13	54	0	0
11	2680	12	Placebo	1	11	7/27/2016	76	HEIGHT		15		0	0
11	2680	24	Placebo	1	23	5/22/2017	83	HEIGHT	10.05	13.5		0	0
11	2680	36	Placebo	1	38	6/10/2018	91.7	HEIGHT	13	14		0	0
11	2680	60	Placebo	1	58	2/14/2020	105.3	HEIGHT	16.85	15.3		0	0
11	2681	0	Placebo	0	4	3/18/2015	65.6	LENGTH	7.8	15.5		0	0
11	2684	12	Placebo	1	21	7/27/2016	80.7	LENGTH		16.6		1	0
11	2684	24	Placebo	1	32	5/22/2017	87.9	HEIGHT	12.75	15		1	0
11	2684	36	Placebo	1	44	6/10/2018	98.8	HEIGHT	15.4	15.5		1	0
11	2684	48	Placebo	1	55	5/15/2019	105	HEIGHT	17.15	16		1	0
11	2686	24	Placebo	0	44	5/22/2017	98.5	HEIGHT	13.9	15.5		1	0
11	2688	0	Placebo	0	48	6/9/2015	105.2	HEIGHT	14.15	14		1	0
11	2688	12	Placebo	0	78	7/27/2016	110.3	HEIGHT		14		0	0
11	2688	36	Placebo	0	104	6/10/2018	121.1	HEIGHT	17.7	13.5		0	0
11	2691	0	Placebo	1	54	3/18/2015	100.9	HEIGHT	14.05	14.5		1	0
11	2691	24	Placebo	1	68	5/22/2017	111.8	HEIGHT	16.6	14		0	0
11	2691	36	Placebo	1	80	6/10/2018	116.5	HEIGHT	17.2	14.5		0	0
11	2691	60	Placebo	1	100	2/14/2020	123.2	HEIGHT	21.5	15.2		0	0
11	2697	0	Placebo	0	48	3/14/2015	98	HEIGHT	16.7	17.5		0	0
11	2697	12	Placebo	0	54	7/27/2016	108.6	HEIGHT		18.8		0	0
11	2697	24	Placebo	0	68	5/22/2017	113.8	HEIGHT	21.05	16.5		0	0
11	2697	48	Placebo	0	91	5/15/2019	123.4	HEIGHT	25.15	16.5		0	0
11	2697	60	Placebo	0	100	2/14/2020	127.2	HEIGHT	26.85	16.7		0	0
11	2698	12	Placebo	1	18	7/27/2016	90.8	HEIGHT		15.3		0	0
11	2698	36	Placebo	1	50	6/10/2018	103.6	HEIGHT	16.75	16		1	0
11	2699	24	Placebo	1	56	5/22/2017	99.6	HEIGHT	14.6	15		1	0
11	2701	0	Placebo	0	36	3/14/2015	83.5	HEIGHT	11.35	14		0	0
11	2701	12	Placebo	0	54	7/27/2016	94.1	HEIGHT		16.2		0	0
11	2701	24	Placebo	0	68	5/22/2017	99.3	HEIGHT	13.7	13.5		0	0
11	2701	36	Placebo	0	80	6/10/2018	104.8	HEIGHT	14.6	14		0	0
11	2703	12	Placebo	0	57	7/27/2016	103.4	HEIGHT		16		0	0
11	2704	0	Placebo	0	12	3/14/2015	64.1	LENGTH	5.7	11.5		0	0
11	2704	12	Placebo	0	18	7/27/2016	79.1	HEIGHT		13.8		0	0

11	2704	24	Placebo	0	32	5/22/2017	83.6	HEIGHT	10.75	14	1	0
11	2704	36	Placebo	0	44	6/10/2018	89.2	HEIGHT	11.95	14	0	0
11	2707	12	Placebo	0	10	7/27/2016	75.5	HEIGHT		16	0	0
11	2707	24	Placebo	0	23	5/22/2017	82.7	HEIGHT	11.4	16	1	0
11	2707	36	Placebo	0	37	6/10/2018	91.9	HEIGHT	13.95	17	0	0
11	2707	60	Placebo	0	57	2/14/2020	106.3	HEIGHT	17.55	17	0	0
11	2708	0	Placebo	1	48	3/14/2015	95.6	HEIGHT	11.55	12.5	0	0
11	2708	48	Placebo	1	98	5/15/2019	124.4	HEIGHT	17.35	12.5	0	0
11	2708	60	Placebo	1	107	2/14/2020	128.5	HEIGHT	19.9	12.7	0	0
11	2712	24	Placebo	1	12	5/22/2017	67.6	LENGTH	7.8	13.5	1	0
11	2712	36	Placebo	1	25	6/10/2018	82.1	LENGTH	12.4	17.5	0	0
11	2712	48	Placebo	1	36	5/15/2019	91.9	HEIGHT	14.1	17	0	0
11	2714	0	Placebo	1	8	3/14/2015	74.4	HEIGHT	8.15	13	1	0
11	2714	12	Placebo	1	15	7/27/2016	81.3	HEIGHT		16.1	0	0
11	2714	24	Placebo	1	28	5/22/2017	87	HEIGHT	11.7	15	1	0
11	2714	36	Placebo	1	41	6/10/2018	94.6	HEIGHT	13.4	14.5	1	0
11	2714	48	Placebo	1	52	5/15/2019	100.2	HEIGHT	15.15	14.5	0	0
11	2714	60	Placebo	1	61	2/14/2020	126.7	HEIGHT	26.35	17.8	0	0
11	2716	0	Placebo	1	24	3/14/2015	78.7	HEIGHT	9.35	14.5	0	0
11	2717	0	Placebo	1	24	3/18/2015	77.4	HEIGHT	9.4	13.5	0	0
11	2717	12	Placebo	1	33	7/27/2016	87.7	HEIGHT		16	1	0
11	2717	24	Placebo	1	46	5/22/2017	93	HEIGHT	12.85	15	0	0
11	2717	36	Placebo	1	59	6/10/2018	101.7	HEIGHT	14.75	14	1	0
11	2717	48	Placebo	1	70	5/15/2019	107	HEIGHT	15.5	13.5	0	0
11	2717	60	Placebo	1	79	2/14/2020	112.7	HEIGHT	17.1	15	0	0
11	2718	0	Placebo	0	24	3/14/2015	85.7	HEIGHT	11	14	1	0
11	2718	12	Placebo	0	54	7/27/2016	97.8	HEIGHT		16.5	0	0
11	2718	24	Placebo	0	51	5/22/2017	103.6	HEIGHT	14.4	14	0	0
11	2718	36	Placebo	0	63	6/10/2018	110.3	HEIGHT	16.25	14	0	0
11	2718	48	Placebo	0	74	5/15/2019	116.2	HEIGHT	17.5	14	0	0
11	2718	60	Placebo	0	83	2/14/2020	122.4	HEIGHT	19.5	14.5	0	0
11	2719	0	Placebo	0	36	3/14/2015	104.4	HEIGHT	16.8	16	1	0
11	2719	12	Placebo	0	48	7/27/2016	112.3	HEIGHT		17.9	0	0
11	2719	24	Placebo	0	62	5/22/2017	116	HEIGHT	20.75	16	0	0
11	2719	48	Placebo	0	86	5/15/2019	123.6	HEIGHT	23.85	16	0	0
11	2719	60	Placebo	0	95	2/14/2020	126.9	HEIGHT	25.05	17	0	0
11	2720	24	Placebo	0	56	5/22/2017	104.6	HEIGHT	16.25	16	1	0
11	2722	0	Placebo	1	36	3/14/2015	101.1	HEIGHT	15.2	15.5	1	0
11	2722	12	Placebo	1	54	7/27/2016	111.7	HEIGHT		16.9	0	0
11	2723	0	Placebo	0	42	3/14/2015	92.2	HEIGHT	12.4	15	0	0

11	2723	24	Placebo	0	68	5/22/2017	103.9	HEIGHT	15.55	14		0	0
11	2724	0	Placebo	0	48	3/14/2015	85.5	HEIGHT	11.5	15		1	0
11	2724	12	Placebo	0	42	7/27/2016	95.7	HEIGHT		17.3		1	0
11	2724	24	Placebo	0	56	5/22/2017	101.6	HEIGHT	14.45	14.5		0	0
11	2724	36	Placebo	0	68	6/10/2018	108.2	HEIGHT	16.25	14.5		0	0
11	2724	48	Placebo	0	79	5/15/2019	113.1	HEIGHT	17.5	14		0	0
11	2724	60	Placebo	0	88	2/14/2020	119.4	HEIGHT	20.2	15.1		0	0
11	2728	0	Placebo	1	24	3/18/2015	72.2	LENGTH	8.55	14.5		0	0
11	2730	24	Placebo	1	9	5/22/2017	60.2	HEIGHT	5	10.5		1	0
11	2730	36	Placebo	1	21	6/10/2018	76.5	LENGTH	11.05	15		0	0
11	2730	48	Placebo	1	32	5/15/2019	81.3	HEIGHT	12.6	16		1	0
11	2730	60	Placebo	1	41	2/14/2020	90.9	HEIGHT	16.15	17.5		0	0
11	2732	0	Placebo	1	18	3/14/2015	83.1	HEIGHT	9.65	13.5		0	0
11	2732	12	Placebo	1	30	7/27/2016	96.7	HEIGHT		16.5		0	0
11	2732	24	Placebo	1	44	5/22/2017	102.5	HEIGHT	14.1	13.5		0	0
11	2732	36	Placebo	1	56	6/10/2018	110.5	HEIGHT	16.15	13.5		0	0
11	2732	48	Placebo	1	67	5/15/2019	115.8	HEIGHT	16.85	13		0	0
11	2732	60	Placebo	1	76	2/14/2020	120.9	HEIGHT	18.2	13.5		0	0
11	2735	12	Placebo	1	0	7/27/2016	62.3	LENGTH		15.2	36	0	0
11	2735	24	Placebo	1	12	5/22/2017	75.4	HEIGHT	8.4	13.5	36	0	0
11	2737	12	Placebo	1	8	7/27/2016	70.2	LENGTH		17.9		0	0
11	2737	60	Placebo	1	53	2/14/2020	91.8	HEIGHT	12.05	14		0	0
11	2738	12	Placebo	1	9	7/27/2016	71.4	LENGTH		14		1	0
11	2738	24	Placebo	1	17	5/22/2017	80.8	HEIGHT	9.55	14		1	0
11	2738	36	Placebo	1	29	6/10/2018	89.9	HEIGHT	11.75	14.5		0	0
11	2739	0	Placebo	1	12	6/9/2015	79.9	HEIGHT	8.75	13		1	0
11	2739	12	Placebo	1	42	7/27/2016	88.9	HEIGHT		16.5		0	0
11	2739	24	Placebo	1	56	5/22/2017	94.9	HEIGHT	12.25	14.5		0	0
11	2739	36	Placebo	1	68	6/10/2018	103.8	HEIGHT	14.15	14		0	0
11	2740	12	Placebo	0	6	7/27/2016	69.5	LENGTH		14.5		0	0
11	2740	24	Placebo	0	21	5/22/2017	78.3	LENGTH	8.75	13		0	0
11	2740	36	Placebo	0	34	6/10/2018	87.7	LENGTH	11.2	14		1	0
11	2740	60	Placebo	0	54	2/14/2020	100.1	HEIGHT	14.35	14		0	0
11	2742	0	Placebo	0	48	3/14/2015	88.4	HEIGHT	12.05	14		0	0
11	2742	12	Placebo	0	66	7/27/2016	99.3	HEIGHT		16.5		0	0
11	2742	24	Placebo	0	80	5/22/2017	103.7	HEIGHT	14.6	13.5		0	0
11	2743	12	Placebo	1	21	7/27/2016	75.2	HEIGHT		16.1		1	0
11	2743	36	Placebo	1	40	6/10/2018	87.9	HEIGHT	10.95	13		1	0
11	2744	0	Placebo	1	48	3/14/2015	94.3	HEIGHT	12.3	14.5		1	0
11	2744	12	Placebo	1	57	7/27/2016	102.5	HEIGHT		16.5		0	0

11	2744	24	Placebo	1	70	5/22/2017	107	HEIGHT	15.8	14		0	0
11	2744	60	Placebo	1	103	2/14/2020	119.3	HEIGHT	20.75	15.5		0	0
11	2746	0	Placebo	1	54	3/14/2015	101.5	HEIGHT	16.85	15.5		1	0
11	2748	0	Placebo	1	48	6/9/2015	85.2	HEIGHT	10.5	14		0	0
11	2748	12	Placebo	1	42	7/27/2016	94.3	HEIGHT		16.6		0	0
11	2748	24	Placebo	1	76	5/22/2017	98.1	HEIGHT	13	14.5		0	0
11	2748	48	Placebo	1	99	5/15/2019	106.7	HEIGHT	15.75	15		0	0
11	2748	60	Placebo	1	108	2/14/2020	109.3	HEIGHT	17.45	15.4		0	0
11	2749	0	Placebo	0	42	3/14/2015	99.5	HEIGHT	13.95	15.5	12	0	0
11	2751	0	Placebo	1	54	3/14/2015	108	HEIGHT	16.15	15		0	0
11	2752	24	Placebo	1	11	5/22/2017	71.2	HEIGHT	7.3	13		1	0
11	2753	0	Placebo	0	10	3/14/2015	68.5	LENGTH	8.1	15		0	0
11	2753	12	Placebo	0	21	7/27/2016	75.2	HEIGHT		17.8		1	0
11	2753	24	Placebo	0	34	5/22/2017	79.3	HEIGHT	9.95	15.5		0	0
11	2753	36	Placebo	0	47	6/10/2018	82.7	HEIGHT	11	14		0	0
11	2753	48	Placebo	0	58	5/15/2019	84.6	HEIGHT	11.15	14		0	0
11	2755	12	Placebo	1	12	7/27/2016	77.2	HEIGHT		15		1	0
11	2755	24	Placebo	1	23	5/22/2017	81.7	HEIGHT	10.3	13.5		1	0
11	2755	36	Placebo	1	37	6/10/2018	91.3	HEIGHT	12.55	14		0	0
11	2755	48	Placebo	1	48	5/15/2019	96.9	HEIGHT	13.7	14.5		1	0
11	2756	0	Placebo	1	24	6/9/2015	88.1	HEIGHT	11.55	14.5		0	0
11	2757	0	Placebo	0	54	3/14/2015	113.4	HEIGHT	19.5	16.5		0	0
11	2757	12	Placebo	0	66	7/27/2016	118.1	LENGTH		18.6		0	0
11	2757	48	Placebo	0	104	5/15/2019	128.5	HEIGHT	25	16.5		0	0
11	2761	24	Placebo	1	12	5/22/2017	74.9	LENGTH	7.3	12		1	0
11	2761	36	Placebo	1	25	6/10/2018	83.9	HEIGHT	8.05	11		0	0
11	2762	24	Placebo	0	32	5/22/2017	91.4	HEIGHT	13.1	14		1	0
11	2763	0	Placebo	1	12	3/14/2015	72.8	HEIGHT	9.05	15		0	0
11	2763	12	Placebo	1	21	7/27/2016	84.3	HEIGHT		18.5		0	0
11	2763	24	Placebo	1	34	5/22/2017	92.5	HEIGHT	15.2	17		1	0
11	2763	36	Placebo	1	47	6/10/2018	100.5	HEIGHT	15.95	15.5		0	0
11	2763	48	Placebo	1	58	5/15/2019	103.3	HEIGHT	17	16		1	0
11	2763	60	Placebo	1	67	2/14/2020	107.2	HEIGHT	18.5	15.8		0	0
11	2764	0	Placebo	0	18	3/14/2015	72.4	HEIGHT	7.9	12		1	0
11	2764	24	Placebo	0	40	5/22/2017	91.9	HEIGHT	11.05	13		0	0
11	2764	36	Placebo	0	55	6/10/2018	100	HEIGHT	12.85	13		0	0
11	2764	48	Placebo	0	66	5/15/2019	105.3	HEIGHT	13.9	13.5		0	0
11	2764	60	Placebo	0	75	2/14/2020	109.5	HEIGHT	14.65	12.8		0	0
11	2766	0	Placebo	1	12	3/18/2015	68.2	LENGTH	7.25	13.5		0	0
11	2766	12	Placebo	1	18	7/27/2016	80.8	LENGTH		14.6		1	0

11	2766	24	Placebo	1	32	5/22/2017	85.8	HEIGHT	11	14.5		1	0
11	2766	36	Placebo	1	44	6/10/2018	92.6	HEIGHT	11.6	13		0	0
11	2766	60	Placebo	1	56	2/14/2020	102.2	HEIGHT	15.5	14		1	0
11	2771	0	Placebo	0	36	3/14/2015	102.7	HEIGHT	13	13.5		0	0
11	2771	12	Placebo	0	66	7/27/2016	111.6	HEIGHT		15.5		0	0
11	2771	24	Placebo	0	80	5/22/2017	116.2	HEIGHT	15.95	13.5		0	0
11	2772	12	Placebo	0	7	7/27/2016	73.4	HEIGHT		15		0	0
11	2772	24	Placebo	0	17	5/22/2017	80.9	LENGTH	10.25	14		1	0
11	2772	36	Placebo	0	29	6/10/2018	90.1	HEIGHT	11.8	13.5		1	0
11	2772	48	Placebo	0	40	5/15/2019	96.2	HEIGHT	12.9	13		1	0
11	2772	60	Placebo	0	49	2/14/2020	100.1	HEIGHT	14.3	12.6		1	0
11	2777	12	Placebo	1	42	7/27/2016	106.6	HEIGHT		17		1	0
11	2782	0	Placebo	0	36	3/14/2015	96.8	HEIGHT	14.15	15.5		0	0
11	2782	36	Placebo	0	71	6/10/2018	114	HEIGHT	19.15	15		0	0
11	2782	48	Placebo	0	82	5/15/2019	117.3	HEIGHT	20.6	15.5		0	0
11	2782	60	Placebo	0	91	2/14/2020	120.8	HEIGHT	22.65	16.2		0	0
11	2784	12	Placebo	0	18	7/27/2016	78.2	HEIGHT		14.5		0	0
11	2785	0	Placebo	1	48	3/18/2015	88.3	HEIGHT	10.25	12.5	12	0	0
11	2786	0	Placebo	1	18	3/14/2015	84.3	HEIGHT	10.9	15		1	0
11	2786	12	Placebo	1	45	7/27/2016	96.3	HEIGHT		17.7		0	0
11	2786	36	Placebo	1	68	6/10/2018	107.5	HEIGHT	16.1	14.5		0	0
11	2786	48	Placebo	1	79	5/15/2019	110.8	HEIGHT	17.15	15		0	0
11	2789	0	Placebo	1	18	3/14/2015	79.6	HEIGHT	9.6	14.5		0	0
11	2789	12	Placebo	1	33	7/27/2016	89.5	HEIGHT		16.7		0	0
11	2789	24	Placebo	1	44	5/22/2017	95.1	HEIGHT	13.05	15		1	0
11	2790	0	Placebo	1	36	3/18/2015	89.1	HEIGHT	12.5	15		0	0
11	2790	24	Placebo	1	56	5/22/2017	101.6	HEIGHT	16.15	15.5		1	0
11	2790	36	Placebo	1	68	6/10/2018	110.1	HEIGHT	17.7	15		0	0
11	2792	0	Placebo	0	7	3/14/2015	74.2	HEIGHT	7.35	11.5		0	0
11	2792	12	Placebo	0	15	7/27/2016	82.7	HEIGHT		12		1	0
11	2792	24	Placebo	0	28	5/22/2017	89.1	HEIGHT	10.9	12.5		0	0
11	2792	36	Placebo	0	41	6/10/2018	96.9	HEIGHT	11.5	11.5		0	0
11	2792	48	Placebo	0	52	5/15/2019	101.9	HEIGHT	13.4	12		1	0
11	2792	60	Placebo	0	61	2/14/2020	106.9	HEIGHT	14.7	13		0	0
11	2793	0	Placebo	1	48	6/9/2015	97.2	HEIGHT	11.8	12.5		1	0
11	2793	12	Placebo	1	57	7/27/2016	107.4	HEIGHT		14.3		1	0
11	2793	24	Placebo	1	63	5/22/2017	111.6	HEIGHT	14.95	12.5		0	0
11	2796	0	Placebo	0	48	3/14/2015	102.1	HEIGHT	16.35	15.5		0	0
11	2796	12	Placebo	0	54	7/27/2016	112.7	HEIGHT		17.5		1	0
11	2796	24	Placebo	0	69	5/22/2017	117.4	HEIGHT	21.05	16		0	0

11	2796	36	Placebo	0	81	6/10/2018	123.7	HEIGHT	22.05	16.5	0	0
11	2796	48	Placebo	0	92	5/15/2019	128.1	HEIGHT	25.05	16	0	0
11	2796	60	Placebo	0	101	2/14/2020	131.6	HEIGHT	25.65	16.5	0	0
11	2797	0	Placebo	1	48	3/14/2015	87.3	HEIGHT	12.05	15	0	0
11	2797	12	Placebo	1	66	7/27/2016	100.2	HEIGHT		19.1	0	0
11	2797	24	Placebo	1	80	5/22/2017	105	HEIGHT	16.3	16	0	0
11	2797	36	Placebo	1	92	6/10/2018	111.6	HEIGHT	17.95	14.5	0	0
11	2797	48	Placebo	1	103	5/15/2019	114.3	HEIGHT	19.1	16	0	0
11	2797	60	Placebo	1	112	2/14/2020	117.4	HEIGHT	20.3	15.5	0	0
11	2798	0	Placebo	0	24	3/14/2015	81.2	HEIGHT	10.75	14.5	1	0
11	2798	12	Placebo	0	42	7/27/2016	92.3	HEIGHT		17.4	1	0
11	2798	48	Placebo	0	79	5/15/2019	107.6	HEIGHT	15.8	14	0	0
11	2799	0	Placebo	0	12	3/14/2015	72.2	LENGTH	8.4	14.5	1	0
11	2799	12	Placebo	0	30	7/27/2016	84.4	HEIGHT		17.5	1	0
11	2799	24	Placebo	0	44	5/22/2017	90	HEIGHT	11.75	14	1	0
11	2799	36	Placebo	0	51	6/10/2018	98.3	HEIGHT	13.6	14.5	1	0
11	2799	48	Placebo	0	62	5/15/2019	104.2	HEIGHT	14.2	13.5	0	0
11	2799	60	Placebo	0	71	2/14/2020	109.6	HEIGHT	16.65	14	0	0
11	2800	0	Placebo	0	18	3/14/2015	79.2	HEIGHT	10.3	15.5	1	0
11	2800	12	Placebo	0	30	7/27/2016	90	HEIGHT		18	0	0
11	2805	0	Placebo	1	24	3/14/2015	82.1	HEIGHT	9.6	13	0	0
11	2807	0	Placebo	1	12	3/14/2015	69.7	HEIGHT	7.5	13	0	0
11	2807	12	Placebo	1	30	7/27/2016	82.3	HEIGHT		16.1	1	0
11	2807	24	Placebo	1	41	5/22/2017	86.6	HEIGHT	11.85	14	0	0
11	2807	36	Placebo	1	53	6/10/2018	95.5	HEIGHT	13.35	14	0	0
11	2807	48	Placebo	1	64	5/15/2019	100.9	HEIGHT	15.25	14	0	0
11	2808	12	Placebo	0	12	7/27/2016	77.5	HEIGHT		15.3	1	0
11	2808	36	Placebo	0	35	6/10/2018	91.2	HEIGHT	14.2	16	0	0
11	2808	48	Placebo	0	46	5/15/2019	97.3	HEIGHT	15.35	16	1	0
11	2810	12	Placebo	1	12	7/27/2016	74.7	HEIGHT		14.8	1	0
11	2810	24	Placebo	1	25	5/22/2017	82.6	HEIGHT	9.55	13	1	0
11	2810	36	Placebo	1	38	6/10/2018	91.6	HEIGHT	11.45	13	0	0
11	2810	48	Placebo	1	49	5/15/2019	98.7	HEIGHT	13.75	13.5	0	0
11	2812	0	Placebo	0	48	3/14/2015	104.8	HEIGHT	16.1	15	1	0
11	2812	12	Placebo	0	54	7/27/2016	112.9	HEIGHT		17.3	0	0
11	2812	24	Placebo	0	68	5/22/2017	117.9	HEIGHT	19.45	15.5	0	0
11	2812	48	Placebo	0	105	5/15/2019	128	HEIGHT	22.4	15	0	0
11	2812	60	Placebo	0	114	2/14/2020	132.9	HEIGHT	23.95	15.6	0	0
11	2815	12	Placebo	0	33	7/27/2016	92	HEIGHT		15.3	1	0
11	2815	36	Placebo	0	56	6/10/2018	103.8	HEIGHT	16.45	15	1	0

11	2816	12	Placebo	1	69	7/27/2016	101.7	HEIGHT		17	0	0
11	2821	0	Placebo	1	18	3/14/2015	73.8	LENGTH	9.05	15	0	0
11	2821	12	Placebo	1	30	7/27/2016	86.7	HEIGHT		18.2	0	0
11	2821	24	Placebo	1	44	5/22/2017	93.9	HEIGHT	13.4	15	1	0
11	2821	36	Placebo	1	56	6/10/2018	102.7	HEIGHT	16	15	1	0
11	2821	48	Placebo	1	67	5/15/2019	109.9	HEIGHT	18.25	16	0	0
11	2821	60	Placebo	1	76	2/14/2020	115	HEIGHT	19.65	15.5	0	0
11	2822	12	Placebo	1	65	7/27/2016	120.5	HEIGHT		18.5	0	0
11	2823	0	Placebo	1	24	3/18/2015	88.6	HEIGHT	11.25	14	0	0
11	2823	12	Placebo	1	42	7/27/2016	99.1	HEIGHT		14.9	1	0
11	2823	24	Placebo	1	67	5/22/2017	104.3	HEIGHT	13.7	14	0	0
11	2823	36	Placebo	1	79	6/10/2018	108.7	HEIGHT	15	13.5	0	0
11	2824	0	Placebo	1	30	3/14/2015	85.8	HEIGHT	13.6	17.5	1	0
11	2824	12	Placebo	1	39	7/27/2016	95.9	HEIGHT		19.8	0	0
11	2824	24	Placebo	1	52	5/22/2017	97.7	HEIGHT	16.1	13.5	1	0
11	2824	36	Placebo	1	65	6/10/2018	104.5	HEIGHT	18.35	17	0	0
11	2824	48	Placebo	1	76	5/15/2019	108.6	HEIGHT	19.9	17	0	0
11	2824	60	Placebo	1	85	2/14/2020	113.1	HEIGHT	22.3	16.7	0	0
11	2825	12	Placebo	1	54	7/27/2016	100.1	HEIGHT		17.3	0	0
11	2830	0	Placebo	0	48	3/18/2015	100	HEIGHT	16.4	15.5	0	0
11	2832	0	Placebo	0	18	3/14/2015	73.7	LENGTH	10	15.5	0	0
11	2832	12	Placebo	0	30	7/27/2016	88.1	HEIGHT		18.2	0	0
11	2832	48	Placebo	0	57	5/15/2019	106	HEIGHT	17.7	16	1	0
11	2833	0	Placebo	1	48	3/14/2015	101.4	HEIGHT	14.9	14.5	1	0
11	2833	24	Placebo	1	70	5/22/2017	113.6	HEIGHT	19.25	16	0	0
11	2833	36	Placebo	1	83	6/10/2018	118.2	HEIGHT	20.6	15.5	0	0
11	2833	60	Placebo	1	103	2/14/2020	124.8	HEIGHT	24.4	16.9	0	0
11	2835	12	Placebo	0	7	7/27/2016	73.8	HEIGHT		15.3	0	0
11	2835	48	Placebo	0	44	5/15/2019	99.8	HEIGHT	13.55	14	1	0
11	2836	0	Placebo	1	18	3/14/2015	69.8	LENGTH	6.9	12.5	1	0
11	2836	12	Placebo	1	30	7/27/2016	82.4	HEIGHT		14.4	1	0
11	2836	24	Placebo	1	39	5/22/2017	89.2	HEIGHT	10.85	13.5	1	0
11	2836	36	Placebo	1	51	6/10/2018	96.7	HEIGHT	11.9	13	0	0
11	2836	48	Placebo	1	62	5/15/2019	103.2	HEIGHT	13.65	13	0	0
11	2836	60	Placebo	1	71	2/14/2020	108.1	HEIGHT	15.35	13.4	0	0
11	2837	0	Placebo	0	48	3/14/2015	89.2	HEIGHT	13	15	1	0
11	2837	12	Placebo	0	54	7/27/2016	100.9	HEIGHT		17.2	0	0
11	2837	24	Placebo	0	68	5/22/2017	106.2	HEIGHT	16.15	14.5	0	0
11	2838	0	Placebo	0	12	3/14/2015	67.8	LENGTH	7.75	14.5	0	0
11	2838	12	Placebo	0	18	7/27/2016	86.3	HEIGHT		17.9	0	0

11	2838	36	Placebo	0	39	6/10/2018	98.9	HEIGHT	14.6	15	1	0
11	2838	48	Placebo	0	50	5/15/2019	103.3	HEIGHT	15.85	14	1	0
11	2838	60	Placebo	0	59	2/14/2020	108.3	HEIGHT	18.05	15.5	0	0
11	2839	12	Placebo	0	6	7/27/2016	71.7	LENGTH		16.8	1	0
11	2839	24	Placebo	0	20	5/22/2017	80.5	HEIGHT	10.15	14	1	0
11	2842	12	Placebo	1	8	7/27/2016	73.2	HEIGHT		15.2	0	0
11	2842	24	Placebo	1	29	5/22/2017	80.7	HEIGHT	9.7	13.5	1	0
11	2843	0	Placebo	0	12	3/14/2015	67.1	LENGTH	6.95	13	1	0
11	2843	36	Placebo	0	47	6/10/2018	93.8	HEIGHT	12.7	13.5	0	0
11	2843	48	Placebo	0	58	5/15/2019	98	HEIGHT	14	13.5	0	0
11	2843	60	Placebo	0	67	2/14/2020	103.4	HEIGHT	16.1	14.5	0	0
11	2846	0	Placebo	0	4	3/14/2015	61	LENGTH	5.8	12.5	0	0
11	2846	12	Placebo	0	12	7/27/2016	74.9	LENGTH		14.1	0	0
11	2848	12	Placebo	1	7	7/27/2016	74	HEIGHT		17.5	0	0
11	2848	24	Placebo	1	17	5/22/2017	80.6	HEIGHT	9.35	13.5	0	0
11	2848	36	Placebo	1	35	6/10/2018	87.4	HEIGHT	11.3	15	0	0
11	2848	48	Placebo	1	46	5/15/2019	93.1	HEIGHT	12.9	15	1	0
11	2848	60	Placebo	1	55	2/14/2020	97	HEIGHT	14.25	14.5	0	0
11	2850	24	Placebo	0	7	5/22/2017	66.5	LENGTH	6.6	13	1	0
11	2850	36	Placebo	0	20	6/10/2018	79.6	LENGTH	9.2	15	1	0
11	2850	48	Placebo	0	30	5/15/2019	84.8	LENGTH	11.65	15	0	0
11	2850	60	Placebo	0	40	2/14/2020	93.6	HEIGHT	14.4	15.9	0	0
11	2851	0	Placebo	1	1	3/14/2015	57.9	LENGTH	5.05	12	0	0
11	2851	12	Placebo	1	12	7/27/2016	80.4	HEIGHT		15.2	1	0
11	2851	24	Placebo	1	25	5/22/2017	87.7	HEIGHT	10.7	13.5	1	0
11	2851	36	Placebo	1	40	6/10/2018	98.3	HEIGHT	13.6	15	0	0
11	2851	48	Placebo	1	51	5/15/2019	105.9	HEIGHT	15.65	15	1	0
11	2851	60	Placebo	1	60	2/14/2020	110.7	HEIGHT	17.2	15.3	0	0
11	2852	12	Placebo	1	30	7/27/2016	83.2	HEIGHT		18.1	1	0
11	2852	24	Placebo	1	44	5/22/2017	89.6	HEIGHT	13.65	16	1	0
11	2854	0	Placebo	1	36	3/14/2015	87.6	HEIGHT	11.45	14.5	0	0
11	2854	12	Placebo	0	42	7/27/2016	100.5	HEIGHT		17.4	1	0
11	2854	24	Placebo	0	56	5/22/2017	105.4	HEIGHT	15.2	15	0	0
11	2854	36	Placebo	0	68	6/10/2018	112.8	HEIGHT	16.75	15	0	0
11	2854	48	Placebo	0	79	5/15/2019	118.3	HEIGHT	19.45	16	0	0
11	2854	60	Placebo	0	88	2/14/2020	123.7	HEIGHT	20.5	16	0	0
11	2855	0	Placebo	0	24	3/14/2015	88	HEIGHT	12.65	16	0	0
11	2855	12	Placebo	0	54	7/27/2016	100	HEIGHT		18.4	0	0
11	2855	48	Placebo	0	91	5/15/2019	118.5	HEIGHT	20.85	16	0	0
11	2860	0	Placebo	1	9	3/18/2015	69.3	LENGTH	7.4	13.5	0	0

11	2860	36	Placebo	1	44	6/10/2018	94.5	HEIGHT	13.55	15.5	0	0
11	2863	0	Placebo	1	36	3/14/2015	95	HEIGHT	14.6	16.5	1	0
11	2863	12	Placebo	1	54	7/27/2016	102.5	HEIGHT		17	1	0
11	2863	24	Placebo	1	68	5/22/2017	107.2	HEIGHT	15.75	14.5	0	0
11	2863	60	Placebo	1	96	2/14/2020	123.3	HEIGHT	21.7	15.3	0	0
11	2864	12	Placebo	0	18	7/27/2016	80	HEIGHT		16.2	1	0
11	2865	0	Placebo	0	4	3/14/2015	65.2	LENGTH	7.1	14	0	0
11	2865	12	Placebo	0	21	7/27/2016	81.2	HEIGHT		16.1	0	0
11	2865	36	Placebo	0	44	6/10/2018	96.2	HEIGHT	13.5	14	1	0
11	2865	48	Placebo	0	55	5/15/2019	101	HEIGHT	15.25	14.5	0	0
11	2865	60	Placebo	0	64	2/14/2020	106.1	HEIGHT	16.8	15	0	0
11	2867	0	Placebo	0	18	6/9/2015	76	HEIGHT	7.35	13	0	0
11	2868	0	Placebo	1	36	3/18/2015	81.7	HEIGHT	12.2	16.5	1	0
11	2868	12	Placebo	1	45	7/27/2016	94.2	HEIGHT		19.2	0	0
11	2868	24	Placebo	1	58	5/22/2017	97.2	HEIGHT	14.35	16	0	0
11	2868	36	Placebo	1	70	6/10/2018	103.5	HEIGHT	15.9	15.5	0	0
11	2868	48	Placebo	1	81	5/15/2019	107.8	HEIGHT	16.15	15	0	0
11	2868	60	Placebo	1	90	2/14/2020	111.5	HEIGHT	18.4	16.5	0	0
11	2869	0	Placebo	0	12	3/14/2015	68	LENGTH	8	15	0	0
11	2869	12	Placebo	0	21	7/27/2016	79.8	HEIGHT		16.4	0	0
11	2869	36	Placebo	0	47	6/10/2018	88.3	HEIGHT	11.55	13.5	0	0
11	2869	48	Placebo	0	58	5/15/2019	94.4	HEIGHT	13.4	14.5	0	0
11	2870	0	Placebo	1	54	6/9/2015	106.9	HEIGHT	15.25	14.5	0	0
11	2870	12	Placebo	1	78	7/27/2016	114.6	HEIGHT		16.7	0	0
11	2870	48	Placebo	1	115	5/15/2019	126	HEIGHT	22.5	16	0	0
11	2870	60	Placebo	1	124	2/14/2020	130.3	HEIGHT	24.3	16.5	0	0
11	2871	0	Placebo	1	24	3/14/2015	77.8	HEIGHT	10.25	14	1	0
11	2871	12	Placebo	1	35	7/27/2016	90	HEIGHT		17.8	0	0
11	2871	24	Placebo	1	46	5/22/2017	96.6	HEIGHT	15.85	16	1	0
11	2871	36	Placebo	1	59	6/10/2018	104.8	HEIGHT	17.6	15.5	1	0
11	2871	60	Placebo	1	79	2/14/2020	116.7	HEIGHT	22.45	16.5	0	0
11	2872	0	Placebo	1	36	3/14/2015	88.3	HEIGHT	13.45	16.5	0	0
11	2872	12	Placebo	1	54	7/27/2016	101.1	HEIGHT		18.3	0	0
11	2872	24	Placebo	1	68	5/22/2017	106.7	HEIGHT	17.6	17	0	0
11	2872	36	Placebo	1	74	6/10/2018	114.7	HEIGHT	20.2	16.5	0	0
11	2872	48	Placebo	1	85	5/15/2019	118.7	HEIGHT	21.75	16	0	0
11	2872	60	Placebo	1	94	2/14/2020	122.9	HEIGHT	24.45	16.9	0	0
11	2874	0	Placebo	0	36	3/14/2015	87.8	HEIGHT	12.75	16	0	0
11	2874	12	Placebo	0	54	7/27/2016	97.1	HEIGHT		15	0	0
11	2876	24	Placebo	1	23	5/22/2017	81.8	HEIGHT	9.35	13.5	1	0

11	2877	12	Placebo	0	9	7/27/2016	69.6	LENGTH		16.5	0	0
11	2877	24	Placebo	0	17	5/22/2017	76.2	HEIGHT	8.95	13.5	0	0
11	2880	0	Placebo	1	54	3/18/2015	101	HEIGHT	14.5	15.5	0	0
11	2880	12	Placebo	1	63	7/27/2016	108	HEIGHT		16.6	0	0
11	2880	24	Placebo	1	76	5/22/2017	112.1	HEIGHT	16.7	14.5	0	0
11	2880	36	Placebo	1	89	6/10/2018	116.7	HEIGHT	18.75	14.5	0	0
11	2880	48	Placebo	1	100	5/15/2019	121.4	HEIGHT	20.2	14.5	0	0
11	2880	60	Placebo	1	109	2/14/2020	124.5	HEIGHT	22.55	15.3	0	0
11	2881	0	Placebo	0	12	3/14/2015	75.2	HEIGHT	8	12.5	0	0
11	2881	12	Placebo	0	30	7/27/2016	88.3	HEIGHT		15.4	0	0
11	2881	24	Placebo	0	40	5/22/2017	93.2	HEIGHT	11.6	13	0	0
11	2881	36	Placebo	0	52	6/10/2018	99.7	HEIGHT	13.05	13	0	0
11	2881	48	Placebo	0	63	5/15/2019	105.3	HEIGHT	14.1	12.5	0	0
11	2882	0	Placebo	0	10	3/14/2015	65.5	LENGTH	5.85	11.5	1	0
11	2882	12	Placebo	0	18	7/27/2016	77	HEIGHT		16.6	1	0
11	2882	24	Placebo	0	32	5/22/2017	83.2	HEIGHT	10.4	14	1	0
11	2882	36	Placebo	0	46	6/10/2018	90.8	HEIGHT	12	14.5	0	0
11	2882	48	Placebo	0	57	5/15/2019	94.3	HEIGHT	13.65	13.5	1	0
11	2882	60	Placebo	0	66	2/14/2020	99.9	HEIGHT	14.75	14.5	0	0
11	2883	0	Placebo	0	24	3/14/2015	73.6	LENGTH	8.85	15	0	0
11	2883	12	Placebo	0	33	7/27/2016	85.2	HEIGHT		14.5	0	0
11	2883	24	Placebo	0	41	5/22/2017	89.7	HEIGHT	12.85	15	0	0
11	2883	48	Placebo	0	60	5/15/2019	106	HEIGHT	16.9	15	0	0
11	2883	60	Placebo	0	69	2/14/2020	111.9	HEIGHT	18.55	15.5	0	0
11	2889	0	Placebo	0	48	3/14/2015	118.2	HEIGHT	19.45	16.5	0	0
11	2889	24	Placebo	0	128	5/22/2017	127.9	HEIGHT	22.55	16	0	0
11	2891	0	Placebo	0	48	3/14/2015	107.5	HEIGHT	16.65	15	0	0
11	2891	12	Placebo	0	57	7/27/2016	116.7	HEIGHT		17.2	0	0
11	2891	24	Placebo	0	69	5/22/2017	120.9	HEIGHT	19.65	15	0	0
11	2891	48	Placebo	0	92	5/15/2019	130.3	HEIGHT	23.8	15	0	0
11	2891	60	Placebo	0	101	2/14/2020	132.4	HEIGHT	26.25	16	0	0
11	2893	0	Placebo	1	48	3/14/2015	98	HEIGHT	17	16	0	0
11	2893	12	Placebo	1	42	7/27/2016	106.5	HEIGHT		18.6	0	0
11	2893	48	Placebo	1	79	5/15/2019	118.8	HEIGHT	23	16.5	0	0
11	2895	0	Placebo	0	24	3/14/2015	78.5	HEIGHT	12.05	17	1	0
11	2895	12	Placebo	0	42	7/27/2016	88.9	HEIGHT		19.9	0	0
11	2895	24	Placebo	0	56	5/22/2017	95	HEIGHT	16.1	16	0	0
11	2895	36	Placebo	0	68	6/10/2018	100.2	HEIGHT	17.3	16.5	0	0
11	2895	48	Placebo	0	79	5/15/2019	104.1	HEIGHT	18.15	16	0	0
11	2895	60	Placebo	0	88	2/14/2020	108.2	HEIGHT	18.85	15.9	0	0

11	2896	0	Placebo	0	36	3/14/2015	91.4	HEIGHT	11.2	14.5		1	0
11	2896	12	Placebo	0	45	7/27/2016	103.3	HEIGHT		15.9		0	0
11	2896	24	Placebo	0	58	5/22/2017	108.7	HEIGHT	14.15	13.5		0	0
11	2896	36	Placebo	0	72	6/10/2018	115.3	HEIGHT	15.05	13.5		0	0
11	2896	48	Placebo	0	83	5/15/2019	119.4	HEIGHT	15.75	13		0	0
11	2896	60	Placebo	0	92	2/14/2020	122.6	HEIGHT	17.6	14		0	0
11	2899	0	Placebo	0	24	3/14/2015	83.1	HEIGHT	8.65	13		1	0
11	2901	0	Placebo	0	48	3/18/2015	92.2	HEIGHT	12.45	14.5		0	0
11	2901	12	Placebo	0	57	7/27/2016	100.2	HEIGHT		16.6		0	0
11	2901	36	Placebo	0	75	6/10/2018	110.3	HEIGHT	16.5	14		0	0
11	2901	48	Placebo	0	86	5/15/2019	114.1	HEIGHT	17.55	13.5		0	0
11	2901	60	Placebo	0	95	2/14/2020	117.8	HEIGHT	19.75	15		0	0
11	2902	0	Placebo	0	48	3/14/2015	100.2	HEIGHT	15.1	16		0	0
11	2902	12	Placebo	0	66	7/27/2016	109.4	HEIGHT		17.7		0	0
11	2902	24	Placebo	0	80	5/22/2017	112.2	HEIGHT	17.45	15		0	0
11	2902	36	Placebo	0	92	6/10/2018	116.9	HEIGHT	19.4	15		0	0
11	2902	48	Placebo	0	103	5/15/2019	118.9	HEIGHT	19.75	14.5		0	0
11	2902	60	Placebo	0	112	2/14/2020	123.2	HEIGHT	21.1	14.3		0	0
11	2903	0	Placebo	0	12	3/18/2015	77.1	LENGTH	8.6	13.5	48	1	0
11	2903	12	Placebo	0	30	7/27/2016	88.8	LENGTH		16	48	0	0
11	2903	36	Placebo	0	56	6/10/2018	102.1	HEIGHT	14.45	13.5	48	1	0
11	2904	12	Placebo	0	10	7/27/2016	75.5	HEIGHT		16.6		0	0
11	2904	24	Placebo	0	23	5/22/2017	80.7	HEIGHT	11.4	15.5		0	0
11	2904	36	Placebo	0	37	6/10/2018	89.2	HEIGHT	13.85	16.5		0	0
11	2904	60	Placebo	0	57	2/14/2020	103.8	HEIGHT	17.5	16.5		0	0
11	2909	0	Placebo	1	24	3/14/2015	81.3	HEIGHT	9.35	13.5		0	0
11	2909	24	Placebo	1	50	5/22/2017	96.6	HEIGHT	12.3	14.5		1	0
11	2909	36	Placebo	1	63	6/10/2018	105	HEIGHT	15.25	14.5		0	0
11	2909	60	Placebo	1	83	2/14/2020	116.9	HEIGHT	18.15	14.8		0	0
11	2910	0	Placebo	1	36	3/14/2015	86.6	HEIGHT	12.15	16	30	0	0
11	2911	12	Placebo	0	9	7/27/2016	74.5	HEIGHT		16.2		1	0
11	2911	36	Placebo	0	29	6/10/2018	93.3	HEIGHT	13.3	14.5		1	0
11	2911	60	Placebo	0	49	2/14/2020	103.1	HEIGHT	15.7	14.4		0	0
11	2912	0	Placebo	0	36	3/18/2015	93.8	HEIGHT	14.95	15		1	0
11	2914	12	Placebo	1	8	7/27/2016	72.6	HEIGHT		15.3		1	0
11	2914	24	Placebo	1	21	5/22/2017	81	HEIGHT	9.6	13.5		0	0
11	2914	36	Placebo	1	34	6/10/2018	89.5	HEIGHT	12.15	14		0	0
11	2914	48	Placebo	1	45	5/15/2019	96.2	HEIGHT	12.5	13		0	0
11	2915	0	Placebo	1	36	3/14/2015	97.3	HEIGHT	12.1	13		1	0
11	2915	24	Placebo	1	58	5/22/2017	112.5	HEIGHT	15.05	13		1	0

11	2915	36	Placebo	1	82	6/10/2018	119.2	HEIGHT	17.15	13	0	0
11	2915	48	Placebo	1	93	5/15/2019	122.9	HEIGHT	18.9	14	0	0
11	2915	60	Placebo	1	102	2/14/2020	127	HEIGHT	20.4	14	0	0
11	2916	0	Placebo	0	36	3/14/2015	104	HEIGHT	14.15	13.5	0	0
11	2916	12	Placebo	0	78	7/27/2016	110	HEIGHT		13.4	0	0
11	2918	12	Placebo	1	6	7/27/2016	67.3	LENGTH		15.1	0	0
11	2918	24	Placebo	1	20	5/22/2017	75.5	LENGTH	9.25	14	0	0
11	2918	36	Placebo	1	32	6/10/2018	84.1	HEIGHT	11.4	15	0	0
11	2918	48	Placebo	1	43	5/15/2019	90.4	HEIGHT	13.15	15	1	0
11	2920	12	Placebo	1	30	7/27/2016	99.6	HEIGHT		19.2	1	0
11	2920	36	Placebo	1	56	6/10/2018	110.4	HEIGHT	17.2	16	1	0
11	2921	12	Placebo	0	8	7/27/2016	71.2	HEIGHT		13.5	1	0
11	2921	24	Placebo	0	17	5/22/2017	76.3	HEIGHT	7.9	11.5	0	0
11	2921	48	Placebo	0	40	5/15/2019	89.4	HEIGHT	11.45	12.5	0	0
11	2921	60	Placebo	0	49	2/14/2020	94.4	HEIGHT	12.1	12.5	1	0
11	2922	0	Placebo	0	9	3/14/2015	64	LENGTH	5.5	12	1	0
11	2922	24	Placebo	0	35	5/22/2017	95.1	HEIGHT	14.05	16	1	0
11	2925	0	Placebo	1	48	3/14/2015	97.5	HEIGHT	13.2	13.5	1	0
11	2925	12	Placebo	1	63	7/27/2016	105.3	HEIGHT		16.5	0	0
11	2925	24	Placebo	1	74	5/22/2017	108.7	HEIGHT	15.35	13.5	0	0
11	2925	36	Placebo	1	87	6/10/2018	114.7	HEIGHT	17.6	13.5	0	0
11	2925	48	Placebo	1	98	5/15/2019	119.9	HEIGHT	18.95	14	0	0
11	2925	60	Placebo	1	107	2/14/2020	124.1	HEIGHT	20.8	14.4	0	0
11	2926	0	Placebo	1	1	3/14/2015	57.5	LENGTH	5.5	13.5	0	0
11	2926	12	Placebo	1	12	7/27/2016	75.6	HEIGHT		16	1	0
11	2926	24	Placebo	1	25	5/22/2017	83.6	HEIGHT	10.25	14	1	0
11	2926	36	Placebo	1	41	6/10/2018	92.2	HEIGHT	12.9	15	0	0
11	2926	48	Placebo	1	52	5/15/2019	99.4	HEIGHT	14.45	15	0	0
11	2926	60	Placebo	1	61	2/14/2020	104.1	HEIGHT	15.35	14.4	0	0
11	2928	12	Placebo	0	54	7/27/2016	105	HEIGHT		19.4	1	0
11	2931	12	Placebo	0	9	7/27/2016	66.2	LENGTH		15	0	0
11	2931	24	Placebo	0	17	5/22/2017	73.1	LENGTH	7.55	13	1	0
11	2933	12	Placebo	0	42	7/27/2016	104.7	HEIGHT		16.9	1	0
11	2934	0	Placebo	1	18	3/14/2015	69.3	LENGTH	8.25	14.5	0	0
11	2934	12	Placebo	1	18	7/27/2016	82	HEIGHT		17.5	0	0
11	2934	24	Placebo	1	32	5/22/2017	90.3	HEIGHT	13.85	16	0	0
11	2934	48	Placebo	1	59	5/15/2019	104.1	HEIGHT	16.25	15.5	0	0
11	2934	60	Placebo	1	68	2/14/2020	107.8	HEIGHT	16.85	15.2	0	0
11	2937	12	Placebo	0	69	7/27/2016	109	HEIGHT		16.4	0	0
11	2938	0	Placebo	0	24	3/14/2015	85.2	HEIGHT	11.75	15	0	0

11	2938	12	Placebo	0	42	7/27/2016	98	HEIGHT		17.7		1	0
11	2938	24	Placebo	0	56	5/22/2017	103.5	HEIGHT	15.55	15		1	0
11	2938	36	Placebo	0	68	6/10/2018	110.2	HEIGHT	17.1	14.5		0	0
11	2938	60	Placebo	0	88	2/14/2020	118.6	HEIGHT	21.55	15.5		0	0
11	2939	0	Placebo	0	36	3/14/2015	98.4	HEIGHT	13.05	14.5		1	0
11	2939	12	Placebo	0	45	7/27/2016	104.3	HEIGHT		16.7		0	0
11	2939	36	Placebo	0	71	6/10/2018	116.1	HEIGHT	18.05	15.5		0	0
11	2939	48	Placebo	0	82	5/15/2019	121.1	HEIGHT	19.85	15		0	0
11	2939	60	Placebo	0	91	2/14/2020	123.9	HEIGHT	21.8	15.5		0	0
11	2940	0	Placebo	0	12	3/14/2015	75.1	LENGTH	8.2	14		1	0
11	2940	36	Placebo	0	51	6/10/2018	101.4	HEIGHT	14.05	14		1	0
11	2941	12	Placebo	0	57	7/27/2016	101.1	HEIGHT		17.7		1	0
11	8011	60	Placebo	0	19	2/17/2020	83.9	HEIGHT	9.6	14.8		1	0
11	8113	60	Placebo	1	42	2/14/2020	90.5	HEIGHT	14.2	16		1	0
11	8124	36	Placebo	1	9	6/10/2018	68.6	LENGTH	7.05	12		1	0
11	8124	48	Placebo	1	16	5/15/2019	76.8	HEIGHT	8.4	12.5		0	0
11	8124	60	Placebo	1	25	2/14/2020	82.7	HEIGHT	10	12.9		0	0
11	8169	60	Placebo	0	3	2/17/2020	62.6	LENGTH	7.3	14		1	0
11	8183	36	Placebo	1	9	6/10/2018	69.3	LENGTH	7.25	13		1	0
11	8183	60	Placebo	1	29	2/14/2020	87	HEIGHT	11.95	15.3		0	0
11	8184	48	Placebo	0	4	5/15/2019	64.7	LENGTH	6.55	12.5		1	0
11	8184	60	Placebo	0	13	2/14/2020	73.6	LENGTH	7.6	11.6		1	0
11	8187	36	Placebo	1	53	6/10/2018	98.2	HEIGHT	14.25	15		1	0
11	8195	60	Placebo	0	6	2/17/2020	71.1	LENGTH	9.6	15		1	0
11	8225	36	Placebo	1	18	6/10/2018	78.7	LENGTH	8.6	12.5		1	0
11	8229	60	Placebo	0	38	2/14/2020	89.3	HEIGHT	13.15	14		1	0
11	8231	36	Placebo	0	56	6/10/2018	107.4	HEIGHT	18.7	16		1	0
11	8235	48	Placebo	1	6	5/15/2019	68.6	LENGTH	7.05	12		1	0
11	8235	60	Placebo	1	15	2/14/2020	79.3	HEIGHT	9.15	12		1	0
11	8238	60	Placebo	0	14	2/17/2020	73.6	HEIGHT	7.9	12.2		1	0
11	8272	48	Placebo	0	33	5/15/2019	83.1	HEIGHT	9.75	12		1	0
11	8294	36	Placebo	1	50	6/10/2018	100.3	HEIGHT	12.55	13		1	0
11	8302	36	Placebo	0	27	6/10/2018	92.8	LENGTH	11.9	14	60	1	0
11	8329	36	Placebo	0	4	6/10/2018	64.7	LENGTH	6.8	13.5		1	0
11	8329	48	Placebo	0	15	5/15/2019	74.9	LENGTH	8.9	13.5		0	0
11	8329	60	Placebo	0	24	2/14/2020	78.7	HEIGHT	9.65	12.5		0	0
11	8336	60	Placebo	1	38	2/14/2020	95.9	HEIGHT	13.6	14.6		1	0
11	8353	36	Placebo	1	21	6/10/2018	77.9	HEIGHT	9.35	14	54	1	0
11	8391	60	Placebo	1	7	2/14/2020	66.9	LENGTH	6.1	11.5		1	0
11	8398	36	Placebo	0	19	6/10/2018	75.4	LENGTH	7.7	14		1	0

11	8403	60	Placebo	0	23	2/14/2020	80.6	HEIGHT	12.1	15.9		1	0
11	8408	60	Placebo	1	23	2/17/2020	86.9	LENGTH	11.95	14.8		1	0
11	8410	36	Placebo	0	18	6/10/2018	76.7	LENGTH	9.9	15	54	1	0
11	8413	36	Placebo	1	12	6/10/2018	72	LENGTH	8.3	13.5		1	0
11	8413	48	Placebo	1	22	5/15/2019	81.6	HEIGHT	10.75	13		0	0
11	8413	60	Placebo	1	32	2/14/2020	89.8	HEIGHT	12.6	13.6		0	0
11	8438	48	Placebo	1	52	5/15/2019	96.5	HEIGHT	11.8	12.5		1	0
11	8450	48	Placebo	1	9	5/15/2019	65.8	LENGTH	6.15	12		1	0
11	8465	60	Placebo	1	26	2/14/2020	85.3	HEIGHT	11.65	14.8		1	0
11	8480	36	Placebo	0	1	6/10/2018	54.1	LENGTH	4.45	11.5		1	0
11	8480	48	Placebo	0	11	5/15/2019	73.7	LENGTH	9.55	14.5		1	0
11	8526	60	Placebo	1	54	2/14/2020	104	HEIGHT	17.4	15.5		1	0
11	8533	48	Placebo	0	6	5/15/2019	65.1	LENGTH	6.55	13		1	0
11	8533	60	Placebo	0	14	2/14/2020	74.7	HEIGHT	8.05	12.5		0	0
11	8535	60	Placebo	0	32	2/17/2020	88	HEIGHT	10.75	13.2		1	0
11	8567	36	Placebo	1	4	6/10/2018	66.1	LENGTH	7.4	14		1	0
11	8567	48	Placebo	1	15	5/15/2019	76.7	LENGTH	9.3	15		1	0
11	8567	60	Placebo	1	24	2/14/2020	82.2	HEIGHT	11.2	14.6		1	0
11	8597	60	Placebo	1	39	2/14/2020	93	HEIGHT	15.55	16.8		1	0
11	8634	60	Placebo	0	33	2/17/2020	83.4	HEIGHT	12.75	15.8		1	0
11	8656	36	Placebo	0	11	6/10/2018	71.2	LENGTH	8.1	14.5		1	0
11	8656	48	Placebo	0	18	5/15/2019	80.6	LENGTH	10.2	14		1	0
11	8677	36	Placebo	0	1	6/10/2018	53.5	LENGTH	3.5	9		1	0
11	8677	48	Placebo	0	12	5/15/2019	69.2	LENGTH	7.35	12.5		0	0
11	8677	60	Placebo	0	21	2/14/2020	77.9	HEIGHT	8.9	13		1	0
11	8734	36	Placebo	1	12	6/10/2018	77	LENGTH	8.4	13		1	0
11	8778	36	Placebo	0	4	6/10/2018	59.9	LENGTH	5.9	13		1	0
11	8778	48	Placebo	0	14	5/15/2019	73.2	LENGTH	7.8	12		0	0
11	8778	60	Placebo	0	24	2/14/2020	80.8	HEIGHT	9.45	13		1	0
11	8831	60	Placebo	0	24	2/17/2020	77.3	LENGTH	9.3	13		1	0
11	8839	60	Placebo	1	6	2/14/2020	67	LENGTH	7	11.8		1	0
11	8887	48	Placebo	1	37	5/15/2019	81.8	HEIGHT	10.75	14		1	0
11	8887	60	Placebo	1	46	2/14/2020	88.8	HEIGHT	14.45	16		1	0
11	8906	48	Placebo	0	34	5/15/2019	86.2	HEIGHT	10.5	12.5		1	0
11	8906	60	Placebo	0	43	2/14/2020	91.8	HEIGHT	11.85	13.2		1	0
11	8917	60	Placebo	1	40	2/14/2020	98.9	HEIGHT	14.5	14.2		1	0
11	8953	60	Placebo	1	56	2/14/2020	107.8	HEIGHT	15.8	13.2		1	0
11	8954	60	Placebo	0	48	2/17/2020	102.1	HEIGHT	15.65	15.5		1	0
11	8962	48	Placebo	0	9	5/15/2019	73.6	LENGTH	9.75	14.5		1	0
11	8984	60	Placebo	0	8	2/14/2020	64	LENGTH	5.55	10.8		1	0

11	8993	48	Placebo	0	16	5/15/2019	76.7	LENGTH	8.95	12	1	0
11	9018	48	Placebo	0	22	5/15/2019	78.7	HEIGHT	9.7	13.5	1	0
11	9020	48	Placebo	1	4	5/15/2019	60.3	LENGTH	7.2	14	1	0
11	9020	60	Placebo	1	12	2/14/2020	71.6	LENGTH	7.4	11.7	1	0
11	9024	60	Placebo	1	13	2/14/2020	72.8	HEIGHT	7.95	12.2	1	0
11	9039	60	Placebo	0	9	2/17/2020	71.7	LENGTH	9.8	14	1	0
11	9086	36	Placebo	0	19	6/10/2018	83.9	LENGTH	12.2	15.5	1	0
11	9086	48	Placebo	0	30	5/15/2019	91.5	HEIGHT	14.7	16	1	0
11	9086	60	Placebo	0	39	2/17/2020	97.9	HEIGHT	16.75	16	1	0
11	9112	48	Placebo	0	16	5/15/2019	75	LENGTH	7.55	11	1	0
11	9112	60	Placebo	0	25	2/14/2020	82.3	HEIGHT	9.55	12	1	0
11	9129	60	Placebo	0	19	2/17/2020	84.6	HEIGHT	12.2	14.5	1	0
11	9143	48	Placebo	0	21	5/15/2019	76.7	HEIGHT	7.5	11.5	1	0
11	9176	48	Placebo	0	17	5/15/2019	78.8	LENGTH	10.65	15	1	0
11	9189	48	Placebo	0	32	5/15/2019	86.4	HEIGHT	11.05	14	1	0
11	9203	60	Placebo	0	14	2/14/2020	73.2	LENGTH	7.7	12.5	1	0
11	9213	36	Placebo	1	21	6/10/2018	80.4	HEIGHT	10	14	1	0
11	9221	36	Placebo	1	12	6/10/2018	71.7	LENGTH	7.1	12	1	0
11	9260	48	Placebo	1	21	5/15/2019	71.5	LENGTH	6.25	11	1	0
11	9293	48	Placebo	1	35	5/15/2019	88.9	HEIGHT	10.45	13	1	0
11	9308	48	Placebo	0	15	5/15/2019	73.2	LENGTH	8.85	14	1	0
11	9314	60	Placebo	1	14	2/17/2020	72	HEIGHT	8.3	13.6	1	0
11	9323	60	Placebo	1	36	2/17/2020	88.2	HEIGHT	12.85	14.6	1	0
11	9396	36	Placebo	1	15	6/10/2018	75.7	LENGTH	9.55	14.5	1	0
11	9396	48	Placebo	1	26	5/15/2019	83.6	HEIGHT	12.2	15.5	1	0
12	2953	12	Placebo	0	10	7/16/2016	73.1	HEIGHT	8.95	14.5	1	1
12	2953	24	Placebo	0	23	5/14/2017	78.5	HEIGHT	9.590909091	15	0	1
12	2953	48	Placebo	0	46	5/17/2019	96.6	HEIGHT	14.55	14.5	0	1
12	2954	0	Placebo	0	36	3/12/2015	94.3	HEIGHT	14.15	16	0	1
12	2954	12	Placebo	0	46	7/16/2016	103.8	HEIGHT	15.35	15	0	1
12	2954	24	Placebo	0	56	5/14/2017	108.9	HEIGHT	17.13636364	15.5	0	1
12	2954	36	Placebo	0	68	6/20/2018	115.1	HEIGHT	19.05	15.5	0	1
12	2954	48	Placebo	0	79	5/17/2019	120.1	HEIGHT	20.45	15.5	0	1
12	2954	60	Placebo	0	88	2/15/2020	124.1	HEIGHT	22.45	15.7	0	1
12	2955	0	Placebo	0	12	3/12/2015	72	LENGTH	7.95	14	0	1
12	2955	12	Placebo	0	28	7/16/2016	85.3	HEIGHT	11.05	15	0	1
12	2956	0	Placebo	0	24	3/12/2015	83.1	HEIGHT	10.25	13.5	1	1
12	2957	12	Placebo	1	42	7/16/2016	100.2	HEIGHT	15.5	15.5	1	1
12	2959	0	Placebo	1	30	3/12/2015	77.2	HEIGHT	9.1	13.5	1	1
12	2961	24	Placebo	1	9	5/14/2017	66.4	LENGTH	7.318181818	13.5	1	1

12	2961	36	Placebo	1	22	6/20/2018	76.4	HEIGHT	8.85	13.5	1	1
12	2961	48	Placebo	1	33	5/17/2019	83	HEIGHT	10.45	14	0	1
12	2961	60	Placebo	1	42	2/15/2020	89.5	HEIGHT	12	14.2	0	1
12	2962	0	Placebo	1	48	3/12/2015	90.5	HEIGHT	12	14	0	1
12	2962	24	Placebo	1	72	5/14/2017	104.2	HEIGHT	14.22727273	13.5	0	1
12	2962	36	Placebo	1	84	6/20/2018	112	HEIGHT	15.35	14	0	1
12	2963	24	Placebo	0	56	5/14/2017	95.8	HEIGHT	13.18181818	15	1	1
12	2967	12	Placebo	1	24	7/16/2016	74.8	HEIGHT	7.9	13	1	1
12	2967	36	Placebo	1	50	6/20/2018	87.4	HEIGHT	10	13	1	1
12	2969	24	Placebo	1	15	5/14/2017	69.3	LENGTH	7.045454545	13.5	1	1
12	2969	36	Placebo	1	27	6/20/2018	78.3	HEIGHT	8.35	11.5	1	1
12	2969	60	Placebo	1	47	2/15/2020	91.8	HEIGHT	12.5	13.7	1	1
12	2970	12	Placebo	1	58	7/16/2016	92.5	HEIGHT	12	13.5	1	1
12	2971	12	Placebo	1	46	7/16/2016	102.3	HEIGHT	15.45	14.5	1	1
12	2973	0	Placebo	1	24	3/12/2015	82.2	HEIGHT	11.7	15.5	0	1
12	2973	24	Placebo	1	56	5/14/2017	99.4	HEIGHT	16.27272727	16	0	1
12	2973	36	Placebo	1	62	6/20/2018	105.9	HEIGHT	15.85	14.5	0	1
12	2973	48	Placebo	1	73	5/17/2019	110.6	HEIGHT	18.2	15.5	0	1
12	2974	0	Placebo	0	7	3/12/2015	67.7	LENGTH	7.4	14	1	1
12	2974	12	Placebo	0	18	7/16/2016	80.4	HEIGHT	9.65	14	0	1
12	2974	24	Placebo	0	32	5/14/2017	88.5	HEIGHT	11.22727273	15	0	1
12	2974	36	Placebo	0	45	6/20/2018	98.6	HEIGHT	13.2	14	0	1
12	2974	48	Placebo	0	56	5/17/2019	105.2	HEIGHT	14.45	14	1	1
12	2974	60	Placebo	0	65	2/15/2020	109.3	HEIGHT	16.2	14	0	1
12	2975	0	Placebo	1	9	3/12/2015	70.7	LENGTH	9.55	15	1	1
12	2975	12	Placebo	1	30	7/16/2016	80.4	HEIGHT	10.6	15	0	1
12	2975	24	Placebo	1	44	5/14/2017	86.8	HEIGHT	12.68181818	16.5	1	1
12	2975	36	Placebo	1	56	6/20/2018	96.2	HEIGHT	14.75	15.5	0	1
12	2975	48	Placebo	1	67	5/17/2019	102.4	HEIGHT	15.95	16	0	1
12	2975	60	Placebo	1	76	2/15/2020	107.2	HEIGHT	17.35	16	0	1
12	2976	0	Placebo	0	5	3/12/2015	62.1	LENGTH	6.8	13.5	0	1
12	2976	12	Placebo	0	18	7/16/2016	74.8	LENGTH	9.55	13.5	0	1
12	2977	0	Placebo	1	30	3/12/2015	86.6	HEIGHT	13.7	16	0	1
12	2977	12	Placebo	1	40	7/16/2016	98.2	LENGTH	15.75	16.8	0	1
12	2979	0	Placebo	1	48	3/12/2015	85.4	HEIGHT	11.05	13.5	0	1
12	2979	12	Placebo	1	54	7/16/2016	94.1	HEIGHT	12.6	14	0	1
12	2979	60	Placebo	1	100	2/15/2020	121.4	HEIGHT	20.6	15.2	0	1
12	2982	0	Placebo	0	48	3/12/2015	96.4	HEIGHT	12.95	13.5	0	1
12	2982	12	Placebo	0	70	7/16/2016	105.2	HEIGHT	15.3	14	0	1
12	2983	24	Placebo	1	44	5/14/2017	104.9	HEIGHT	14.22727273	14	1	1

12	2983	36	Placebo	1	56	6/20/2018	110.6	HEIGHT	15.5	13.5	1	1
12	2986	0	Placebo	1	24	3/12/2015	75.3	LENGTH	9.1	14.5	1	1
12	2986	12	Placebo	1	30	7/16/2016	84.3	HEIGHT	11.25	14	0	1
12	2986	24	Placebo	1	44	5/14/2017	92	HEIGHT	12.77272727	15.5	0	1
12	2986	36	Placebo	1	56	6/20/2018	101.4	HEIGHT	15.5	15	0	1
12	2986	48	Placebo	1	67	5/17/2019	106.7	HEIGHT	16.25	15	0	1
12	2986	60	Placebo	1	76	2/15/2020	110.5	HEIGHT	16.65	14.2	0	1
12	2988	0	Placebo	1	48	3/12/2015	93.5	HEIGHT	12.9	15	0	1
12	2988	12	Placebo	1	58	7/16/2016	103.6	HEIGHT	15.55	15	0	1
12	2988	24	Placebo	1	72	5/14/2017	108.4	HEIGHT	16.5	14.5	0	1
12	2988	36	Placebo	1	84	6/20/2018	115.6	HEIGHT	18.25	14.5	0	1
12	2988	48	Placebo	1	95	5/17/2019	120.1	HEIGHT	20.45	15	0	1
12	2988	60	Placebo	1	104	2/15/2020	123.5	HEIGHT	23.3	16	0	1
12	2990	12	Placebo	0	6	7/16/2016	64.6	LENGTH	7.35	15	0	1
12	2990	24	Placebo	0	12	5/14/2017	75.8	HEIGHT	9.727272727	15.5	1	1
12	2990	36	Placebo	0	24	6/20/2018	84.4	LENGTH	11.35	15	1	1
12	2990	48	Placebo	0	35	5/17/2019	92.7	HEIGHT	14.25	17	1	1
12	2990	60	Placebo	0	44	2/15/2020	99	HEIGHT	16.45	16.5	0	1
12	2993	0	Placebo	1	54	3/12/2015	90.6	HEIGHT	12.1	14	1	1
12	2993	24	Placebo	1	63	5/14/2017	103.9	HEIGHT	14.5	14.5	0	1
12	2993	48	Placebo	1	86	5/17/2019	116.9	HEIGHT	18	14	0	1
12	2994	12	Placebo	0	12	7/16/2016	75.2	HEIGHT	9.7	14	1	1
12	2994	24	Placebo	0	22	5/14/2017	84.5	HEIGHT	11.77272727	15	0	1
12	2994	48	Placebo	0	45	5/17/2019	102.5	HEIGHT	16.15	15	0	1
12	2996	12	Placebo	0	1	7/16/2016	56.1	LENGTH	6.5	14.5	0	1
12	2996	24	Placebo	0	11	5/14/2017	69.8	HEIGHT	9.136363636	14.5	1	1
12	2996	48	Placebo	0	32	5/17/2019	87.3	HEIGHT	12.2	14	1	1
12	2997	12	Placebo	0	46	7/16/2016	95.6	HEIGHT	15	16	1	1
12	2997	24	Placebo	0	56	5/14/2017	103	HEIGHT	15.36363636	14.5	1	1
12	2998	0	Placebo	0	36	3/12/2015	97	HEIGHT	14.1	14.5	1	1
12	2998	12	Placebo	0	46	7/16/2016	107.9	HEIGHT	16.8	15	0	1
12	2998	60	Placebo	0	88	2/15/2020	126.8	HEIGHT	22.3	15	0	1
12	2999	12	Placebo	1	1	7/16/2016	59.3	LENGTH	5.85	14	1	1
12	2999	24	Placebo	1	15	5/14/2017	69	LENGTH	7.636363636	13	0	1
12	2999	48	Placebo	1	39	5/17/2019	84.6	HEIGHT	10.7	14	0	1
12	2999	60	Placebo	1	48	2/15/2020	90.6	HEIGHT	12.4	14.7	0	1
12	3001	24	Placebo	0	56	5/14/2017	98.5	HEIGHT	15.63636364	16	1	1
12	3003	24	Placebo	1	1	5/14/2017	51.5	LENGTH	4.090909091	11.5	1	1
12	3006	24	Placebo	0	12	5/14/2017	67.9	LENGTH	7.454545455	13.5	1	1
12	3006	36	Placebo	0	24	6/20/2018	80.8	HEIGHT	10.7	15	0	1

12	3006	48	Placebo	0	35	5/17/2019	88.7	HEIGHT	12.95	15	1	1
12	3006	60	Placebo	0	44	2/15/2020	95.3	HEIGHT	14.15	15.2	0	1
12	3007	12	Placebo	0	42	7/16/2016	86	HEIGHT	11.15	14	0	1
12	3007	24	Placebo	0	56	5/14/2017	93.7	HEIGHT	11.81818182	14	0	1
12	3007	36	Placebo	0	58	6/20/2018	98.7	HEIGHT	12.85	13	1	1
12	3008	12	Placebo	0	42	7/16/2016	101.8	HEIGHT	14.55	14.5	1	1
12	3014	12	Placebo	1	12	7/16/2016	71.3	LENGTH	8	13	1	1
12	3014	36	Placebo	1	38	6/20/2018	88.6	HEIGHT	12.9	15	0	1
12	3014	48	Placebo	1	49	5/17/2019	94.7	HEIGHT	13.95	14	1	1
12	3014	60	Placebo	1	58	2/15/2020	99.8	HEIGHT	15.55	14.9	0	1
12	3015	0	Placebo	0	48	3/12/2015	79	HEIGHT	9.2	13.5	1	1
12	3017	12	Placebo	1	9	7/16/2016	74.3	LENGTH	8.95	14.5	0	1
12	3017	60	Placebo	1	55	2/15/2020	101.8	HEIGHT	16.4	15.7	0	1
12	3019	0	Placebo	0	36	3/12/2015	88.2	HEIGHT	12.5	14.5	1	1
12	3019	12	Placebo	0	49	7/16/2016	98.2	HEIGHT	15.6	15.5	1	1
12	3019	24	Placebo	0	60	5/14/2017	104	HEIGHT	15.77272727	15.5	0	1
12	3019	48	Placebo	0	83	5/17/2019	114.6	HEIGHT	18	14.5	0	1
12	3019	60	Placebo	0	92	2/15/2020	119.1	HEIGHT	20.7	15	0	1
12	3020	0	Placebo	0	5	3/12/2015	59.7	LENGTH	4.85	11	0	1
12	3020	12	Placebo	0	19	7/16/2016	70.3	LENGTH	7.8	14	0	1
12	3020	24	Placebo	0	30	5/14/2017	77.6	HEIGHT	9.5	14.5	0	1
12	3020	36	Placebo	0	42	6/20/2018	84.1	HEIGHT	10.75	13.5	1	1
12	3020	48	Placebo	0	53	5/17/2019	88.5	HEIGHT	11.25	13	1	1
12	3021	0	Placebo	0	2	3/12/2015	56.8	LENGTH	5.5	13.5	0	1
12	3021	12	Placebo	0	12	7/16/2016	77.7	HEIGHT	9.85	14.5	0	1
12	3021	36	Placebo	0	38	6/20/2018	96.6	HEIGHT	14.2	15	0	1
12	3021	48	Placebo	0	49	5/17/2019	103.5	HEIGHT	15.7	15	0	1
12	3021	60	Placebo	0	58	2/15/2020	109.7	HEIGHT	17.1	15	0	1
12	3022	0	Placebo	1	24	3/12/2015	82.3	HEIGHT	10.45	14.5	1	1
12	3022	12	Placebo	1	48	7/16/2016	93.3	HEIGHT	14.1	16	0	1
12	3022	24	Placebo	1	57	5/14/2017	100.9	HEIGHT	15.04545455	16	0	1
12	3024	12	Placebo	1	54	7/16/2016	118.5	HEIGHT	17.25	14.5	1	1
12	3026	0	Placebo	1	1	3/12/2015	56.7	LENGTH	4.65	12	0	1
12	3026	12	Placebo	1	12	7/16/2016	77.5	HEIGHT	8.58	13.5	0	1
12	3026	24	Placebo	1	25	5/14/2017	83.9	HEIGHT	10.18181818	14.5	0	1
12	3026	36	Placebo	1	38	6/20/2018	94	HEIGHT	12.4	15	0	1
12	3026	48	Placebo	1	49	5/17/2019	100.8	HEIGHT	13.7	15	1	1
12	3026	60	Placebo	1	58	2/15/2020	106	HEIGHT	14.9	14.2	0	1
12	3029	0	Placebo	1	36	3/12/2015	79.7	HEIGHT	11.65	15	0	1
12	3029	12	Placebo	1	49	7/16/2016	88.2	HEIGHT	13.75	15.5	1	1

12	3029	24	Placebo	1	60	5/14/2017	95	HEIGHT	13.72727273	15.5	1	1
12	3029	48	Placebo	1	83	5/17/2019	105.4	HEIGHT	16.9	15	0	1
12	3030	24	Placebo	0	8	5/14/2017	68.3	LENGTH	7.363636364	12	1	1
12	3030	36	Placebo	0	21	6/20/2018	81.7	HEIGHT	9.8	13	0	1
12	3030	60	Placebo	0	41	2/15/2020	93.6	HEIGHT	13.9	14	0	1
12	3031	12	Placebo	1	58	7/16/2016	115.4	HEIGHT	18	15.5	1	1
12	3032	0	Placebo	0	48	3/12/2015	102.4	HEIGHT	14.55	13.5	1	1
12	3032	12	Placebo	0	61	7/16/2016	109.4	HEIGHT	16.45	14.5	0	1
12	3032	24	Placebo	0	72	5/14/2017	113.8	HEIGHT	18	14.5	0	1
12	3032	36	Placebo	0	84	6/20/2018	118.5	HEIGHT	19.85	15	0	1
12	3032	48	Placebo	0	95	5/17/2019	122.8	HEIGHT	20.5	15.5	0	1
12	3032	60	Placebo	0	104	2/15/2020	125.1	HEIGHT	21.9	15.6	0	1
12	3035	12	Placebo	0	4	7/16/2016	67.4	LENGTH	7.2	13.5	0	1
12	3035	24	Placebo	0	17	5/14/2017	77.2	HEIGHT	9.545454545	13.5	1	1
12	3035	36	Placebo	0	29	6/20/2018	85.3	HEIGHT	11.25	14	0	1
12	3037	0	Placebo	1	24	3/12/2015	72.6	LENGTH	8.3	13	0	1
12	3038	24	Placebo	1	36	5/14/2017	81.6	HEIGHT	10.27272727	14	1	1
12	3040	0	Placebo	1	8	3/12/2015	62.8	LENGTH	5.95	12.5	1	1
12	3040	12	Placebo	1	16	7/16/2016	73.2	HEIGHT	8.3	13.5	1	1
12	3040	24	Placebo	1	34	5/14/2017	82.1	HEIGHT	10.63636364	16	1	1
12	3040	36	Placebo	1	46	6/20/2018	92.9	HEIGHT	13.4	15.5	0	1
12	3040	48	Placebo	1	57	5/17/2019	97.8	HEIGHT	15.05	16	0	1
12	3040	60	Placebo	1	66	2/15/2020	103.9	HEIGHT	16.35	16	0	1
12	3044	12	Placebo	0	54	7/16/2016	110.1	HEIGHT	17	15	1	1
12	3045	0	Placebo	0	48	3/12/2015	101	HEIGHT	16.1	16.5	1	1
12	3045	12	Placebo	0	66	7/16/2016	108.2	LENGTH	16.75	15.7	0	1
12	3045	24	Placebo	0	80	5/14/2017	114.5	HEIGHT	18.54545455	17	0	1
12	3045	36	Placebo	0	92	6/20/2018	119.6	HEIGHT	21.2	16	0	1
12	3045	48	Placebo	0	103	5/17/2019	123.4	HEIGHT	21.1	16	0	1
12	3045	60	Placebo	0	112	2/15/2020	125.7	HEIGHT	24.65	17	0	1
12	3046	0	Placebo	1	4	3/12/2015	59.4	LENGTH	6.05	14	0	1
12	3046	12	Placebo	1	19	7/16/2016	72	HEIGHT	9.2	13.5	0	1
12	3046	24	Placebo	1	30	5/14/2017	79	HEIGHT	9.727272727	15	0	1
12	3046	36	Placebo	1	42	6/20/2018	87.9	HEIGHT	12.3	14.5	1	1
12	3046	48	Placebo	1	53	5/17/2019	92.2	HEIGHT	12.6	14.5	1	1
12	3046	60	Placebo	1	62	2/15/2020	97.8	HEIGHT	14.6	14.5	0	1
12	3049	0	Placebo	0	54	3/12/2015	112.3	HEIGHT	16.9	14.5	1	1
12	3049	24	Placebo	0	92	5/14/2017	121.3	HEIGHT	19.86363636	15.5	0	1
12	3049	36	Placebo	0	104	6/20/2018	125.6	HEIGHT	20.7	15	0	1
12	3049	48	Placebo	0	115	5/17/2019	129.7	HEIGHT	22.8	15.5	0	1

12	3049	60	Placebo	0	124	2/15/2020	133.9	HEIGHT	24.2	15.4		0	1
12	3050	12	Placebo	1	8	7/16/2016	68.4	LENGTH	7.4	13.5		1	1
12	3050	24	Placebo	1	17	5/14/2017	74.7	HEIGHT	8	12.5		0	1
12	3050	36	Placebo	1	29	6/20/2018	81.3	HEIGHT	9.7	12.5		1	1
12	3050	48	Placebo	1	40	5/17/2019	88.9	HEIGHT	12.9	14		0	1
12	3051	0	Placebo	0	54	3/12/2015	110.4	HEIGHT	19.25	16.5		0	1
12	3051	12	Placebo	0	66	7/16/2016	116.6	HEIGHT	21.9	18		0	1
12	3051	24	Placebo	0	80	5/14/2017	120.7	HEIGHT	22.72727273	17.5		0	1
12	3051	36	Placebo	0	92	6/20/2018	125.1	HEIGHT	25.55	17.5		0	1
12	3051	60	Placebo	0	112	2/15/2020	131.2	HEIGHT	31.25	18.4		0	1
12	3053	0	Placebo	1	18	3/12/2015	72.6	HEIGHT	8.4	13.5	6	1	1
12	3054	0	Placebo	0	24	3/12/2015	82.9	HEIGHT	10.5	13.5		0	1
12	3054	12	Placebo	0	30	7/16/2016	93.1	HEIGHT	14.75	15.5		0	1
12	3054	24	Placebo	0	44	5/14/2017	102.2	HEIGHT	15.63636364	15		0	1
12	3054	36	Placebo	0	56	6/20/2018	109.7	HEIGHT	17.8	15.5		0	1
12	3054	48	Placebo	0	67	5/17/2019	112.5	HEIGHT	19	15.5		0	1
12	3054	60	Placebo	0	76	2/15/2020	117.6	HEIGHT	20.45	15.8		0	1
12	3056	0	Placebo	1	42	3/12/2015	87.2	HEIGHT	11.05	13.5		0	1
12	3056	12	Placebo	1	52	7/16/2016	96.7	HEIGHT	13	14		0	1
12	3056	24	Placebo	1	66	5/14/2017	101.5	HEIGHT	14.31818182	15		0	1
12	3056	60	Placebo	1	98	2/15/2020	117.2	HEIGHT	19.25	15		0	1
12	3057	0	Placebo	1	12	3/12/2015	65.3	LENGTH	6.4	11	12	1	1
12	3061	0	Placebo	1	36	3/12/2015	85.8	HEIGHT	11.75	14.5		0	1
12	3061	12	Placebo	1	46	7/16/2016	95.2	HEIGHT	12.95	14		0	1
12	3061	24	Placebo	1	56	5/14/2017	102	HEIGHT	13.77272727	13.5		1	1
12	3061	60	Placebo	1	88	2/15/2020	115.9	HEIGHT	17.95	14.3		0	1
12	3063	0	Placebo	1	24	3/12/2015	72.3	LENGTH	7.6	13	36	0	1
12	3063	12	Placebo	1	42	7/16/2016	81.7	HEIGHT	10.3	13.8	36	0	1
12	3063	24	Placebo	1	50	5/14/2017	87.8	HEIGHT	10.5	14	36	0	1
12	3065	12	Placebo	0	10	7/16/2016	74.2	HEIGHT	8.4	14		0	1
12	3065	24	Placebo	0	23	5/14/2017	81.5	HEIGHT	9.454545455	14.5		1	1
12	3065	60	Placebo	0	56	2/15/2020	100.4	HEIGHT	15.05	15		1	1
12	3066	12	Placebo	1	8	7/16/2016	72	HEIGHT	8.1	12		1	1
12	3066	24	Placebo	1	22	5/14/2017	80.2	HEIGHT	10.13636364	14.5		0	1
12	3066	36	Placebo	1	35	6/20/2018	90.9	HEIGHT	12.3	14		0	1
12	3066	48	Placebo	1	46	5/17/2019	98.6	HEIGHT	14.25	15		0	1
12	3066	60	Placebo	1	55	2/15/2020	103.9	HEIGHT	15.05	15.4		0	1
12	3067	0	Placebo	1	48	3/12/2015	95.2	HEIGHT	14.55	16		0	1
12	3067	12	Placebo	1	64	7/16/2016	104.4	HEIGHT	16.45	16		0	1
12	3068	24	Placebo	0	3	5/14/2017	55.7	LENGTH	5.318181818	13.5		1	1

12	3068	36	Placebo	0	15	6/20/2018	87.8	LENGTH	6.95	12		0	1
12	3068	48	Placebo	0	26	5/17/2019	75.3	HEIGHT	9.45	14		0	1
12	3068	60	Placebo	0	35	2/15/2020	86	HEIGHT	11.55	15		0	1
12	3071	12	Placebo	0	52	7/16/2016	105.3	HEIGHT	15.1	13.5		1	1
12	3072	0	Placebo	0	8	3/12/2015	71	LENGTH	8.35	14		0	1
12	3072	12	Placebo	0	16	7/16/2016	82.7	LENGTH	10.55	13.5		0	1
12	3072	24	Placebo	0	30	5/14/2017	91	HEIGHT	12.59090909	15		1	1
12	3072	36	Placebo	0	42	6/20/2018	102.2	HEIGHT	15.4	14		0	1
12	3072	48	Placebo	0	53	5/17/2019	110.7	HEIGHT	17.6	15		0	1
12	3072	60	Placebo	0	62	2/15/2020	115.2	HEIGHT	20	15.5		0	1
12	3075	0	Placebo	1	1	3/12/2015	59	LENGTH	5.3	11.5		0	1
12	3075	12	Placebo	1	12	7/16/2016	77.2	LENGTH	9.25	13.5		0	1
12	3075	36	Placebo	1	38	6/20/2018	96.4	HEIGHT	13.85	15		1	1
12	3075	48	Placebo	1	49	5/17/2019	102.8	HEIGHT	15.35	14.5		0	1
12	3075	60	Placebo	1	58	2/15/2020	107.1	HEIGHT	16.45	14.4		0	1
12	3077	0	Placebo	1	48	3/12/2015	111.7	HEIGHT	17.9	15.5		0	1
12	3078	12	Placebo	0	30	7/16/2016	82.4	HEIGHT	10.6	14.5	42	1	1
12	3081	0	Placebo	0	3	3/12/2015	61	LENGTH	6.35	13		0	1
12	3081	12	Placebo	0	12	7/16/2016	81.4	LENGTH	9.6	13.5		1	1
12	3082	0	Placebo	0	48	3/12/2015	110.3	HEIGHT	16.25	15.5		1	1
12	3082	12	Placebo	0	54	7/16/2016	117.9	HEIGHT	17.85	14.5		1	1
12	3082	24	Placebo	0	68	5/14/2017	122.3	HEIGHT	19.68181818	15		0	1
12	3082	36	Placebo	0	80	6/20/2018	126.7	HEIGHT	21.65	15		0	1
12	3082	48	Placebo	0	91	5/17/2019	129.6	HEIGHT	23.05	16		0	1
12	3082	60	Placebo	0	100	2/15/2020	132.5	HEIGHT	25.15	16.2		0	1
12	3083	0	Placebo	0	48	3/12/2015	98.7	HEIGHT	12.5	13		0	1
12	3083	12	Placebo	0	61	7/16/2016	106.7	HEIGHT	14.1	13		0	1
12	3083	24	Placebo	0	72	5/14/2017	111.9	HEIGHT	15.27272727	13.5		0	1
12	3083	36	Placebo	0	84	6/20/2018	119.2	HEIGHT	16.8	13		0	1
12	3083	48	Placebo	0	95	5/17/2019	125.9	HEIGHT	19.05	14		0	1
12	3084	0	Placebo	0	24	3/12/2015	77.4	HEIGHT	8.25	11.5		1	1
12	3084	12	Placebo	0	46	7/16/2016	83	HEIGHT	9.35	12.5		0	1
12	3085	0	Placebo	0	48	3/12/2015	80.3	HEIGHT	11.2	14		0	1
12	3085	12	Placebo	0	58	7/16/2016	93.9	HEIGHT	15.65	16.5		0	1
12	3085	24	Placebo	0	53	5/14/2017	100.8	HEIGHT	16.86363636	17		0	1
12	3085	36	Placebo	0	65	6/20/2018	109.3	HEIGHT	18.6	15.5		0	1
12	3085	48	Placebo	0	76	5/17/2019	114.2	HEIGHT	20.5	15.5		0	1
12	3085	60	Placebo	0	86	2/15/2020	117.3	HEIGHT	21.35	16		0	1
12	3086	0	Placebo	0	42	3/12/2015	97.5	HEIGHT	13.7	14		1	1
12	3086	12	Placebo	0	52	7/16/2016	104.6	HEIGHT	15.25	14.9		0	1

12	3086	24	Placebo	0	66	5/14/2017	109.3	HEIGHT	16.5	15	0	1
12	3086	36	Placebo	0	78	6/20/2018	113.8	HEIGHT	18.3	14.5	0	1
12	3086	48	Placebo	0	89	5/17/2019	117.9	HEIGHT	19.4	15	0	1
12	3086	60	Placebo	0	98	2/15/2020	121.7	HEIGHT	21.5	15.2	0	1
12	3089	0	Placebo	0	54	3/12/2015	112.7	HEIGHT	16.95	14	0	1
12	3089	24	Placebo	0	68	5/14/2017	126.5	HEIGHT	20.40909091	14.5	0	1
12	3089	36	Placebo	0	80	6/20/2018	132.5	HEIGHT	23.25	14.5	0	1
12	3089	48	Placebo	0	91	5/17/2019	136.2	HEIGHT	24.45	15.5	0	1
12	3089	60	Placebo	0	100	2/15/2020	141.1	HEIGHT	26.8	16.2	0	1
12	3091	0	Placebo	1	54	3/12/2015	119.9	HEIGHT	17.85	15	0	1
12	3091	12	Placebo	1	102	7/16/2016	125	HEIGHT	21.65	14	0	1
12	3091	36	Placebo	1	128	6/20/2018	134.1	HEIGHT	24.2	16.5	0	1
12	3091	48	Placebo	1	139	5/17/2019	136.4	HEIGHT	28.85	17	0	1
12	3091	60	Placebo	1	148	2/15/2020	141.6	HEIGHT	30.45	18	0	1
12	3093	0	Placebo	1	48	3/12/2015	93.6	HEIGHT	13.25	14.5	0	1
12	3093	24	Placebo	1	74	5/14/2017	110.5	HEIGHT	16.27272727	14	0	1
12	3093	36	Placebo	1	87	6/20/2018	117.6	HEIGHT	17.8	14	0	1
12	3093	48	Placebo	1	98	5/17/2019	123.3	HEIGHT	19.95	14.5	0	1
12	3093	60	Placebo	1	107	2/15/2020	127.1	HEIGHT	22.25	15.5	0	1
12	3097	0	Placebo	1	48	3/12/2015	92.3	HEIGHT	12.3	14.5	0	1
12	3097	12	Placebo	1	58	7/16/2016	103.3	HEIGHT	13.2	13.5	0	1
12	3097	24	Placebo	1	68	5/14/2017	109.6	HEIGHT	14.5	13.5	0	1
12	3097	36	Placebo	1	80	6/20/2018	117.5	HEIGHT	16.6	13.5	0	1
12	3097	48	Placebo	1	91	5/17/2019	122.3	HEIGHT	18.9	14	0	1
12	3098	0	Placebo	0	18	3/12/2015	78.7	HEIGHT	9.15	14.5	0	1
12	3098	12	Placebo	0	28	7/16/2016	86.2	HEIGHT	12.4	15.5	0	1
12	3098	24	Placebo	0	42	5/14/2017	93	HEIGHT	14.40909091	16	0	1
12	3098	36	Placebo	0	54	6/20/2018	100.7	HEIGHT	15.85	15.5	0	1
12	3098	60	Placebo	0	74	2/15/2020	113.3	HEIGHT	19.55	15.5	0	1
12	3100	0	Placebo	0	54	3/12/2015	114.5	HEIGHT	19	15	1	1
12	3103	24	Placebo	1	22	5/14/2017	76.4	HEIGHT	8.5	13	1	1
12	3103	60	Placebo	1	54	2/15/2020	94.2	HEIGHT	12.45	13.9	1	1
12	3107	0	Placebo	0	54	3/12/2015	106.5	HEIGHT	17.2	16.5	0	1
12	3107	60	Placebo	0	113	2/15/2020	147.2	HEIGHT	35.5	19.7	0	1
12	3108	0	Placebo	1	36	3/12/2015	81.2	HEIGHT	11.8	14.5	0	1
12	3108	36	Placebo	1	72	6/20/2018	105.2	HEIGHT	15.75	14	0	1
12	3108	60	Placebo	1	92	2/15/2020	111.8	HEIGHT	18.3	14.3	0	1
12	3110	0	Placebo	1	48	3/12/2015	81.2	HEIGHT	10.85	13.5	0	1
12	3110	24	Placebo	1	57	5/14/2017	95.9	HEIGHT	13.31818182	14	1	1
12	3113	0	Placebo	0	48	3/12/2015	105	HEIGHT	16.05	15.5	0	1

12	3113	12	Placebo	0	78	7/16/2016	111.3	HEIGHT	17.5	14.5	0	1
12	3113	24	Placebo	0	92	5/14/2017	116	HEIGHT	18.63636364	16	0	1
12	3113	36	Placebo	0	104	6/20/2018	121.1	HEIGHT	21	15.5	0	1
12	3113	48	Placebo	0	115	5/17/2019	124.6	HEIGHT	23.7	16	0	1
12	3113	60	Placebo	0	124	2/15/2020	127.2	HEIGHT	24.4	16.9	0	1
12	3114	0	Placebo	1	36	3/12/2015	90.3	HEIGHT	13	15	0	1
12	3117	0	Placebo	1	36	3/12/2015	94.2	HEIGHT	12.75	15.5	0	1
12	3119	0	Placebo	0	54	3/12/2015	106.4	HEIGHT	15.35	13.5	1	1
12	3119	24	Placebo	0	92	5/14/2017	118.5	HEIGHT	18.81818182	14.5	0	1
12	3119	36	Placebo	0	104	6/20/2018	123	HEIGHT	21.25	14.5	0	1
12	3121	12	Placebo	1	7	7/16/2016	66.3	LENGTH	7.5	14	0	1
12	3121	24	Placebo	1	17	5/14/2017	75.3	LENGTH	8.590909091	13	0	1
12	3121	48	Placebo	1	40	5/17/2019	89.8	HEIGHT	12.15	14.5	1	1
12	3122	24	Placebo	1	25	5/14/2017	86.7	HEIGHT	11.77272727	15	1	1
12	3123	0	Placebo	1	30	3/12/2015	74.2	HEIGHT	8.55	13.5	0	1
12	3125	12	Placebo	0	36	7/16/2016	79.3	HEIGHT	8.4	14	1	1
12	3128	0	Placebo	1	12	3/12/2015	68.4	HEIGHT	7.4	13.5	1	1
12	3128	12	Placebo	1	22	7/16/2016	80.4	HEIGHT	9.5	14	0	1
12	3128	24	Placebo	1	36	5/14/2017	90.4	HEIGHT	11.36363636	14.5	0	1
12	3128	36	Placebo	1	48	6/20/2018	98.2	HEIGHT	12.9	14	0	1
12	3128	48	Placebo	1	59	5/17/2019	106.5	HEIGHT	15.25	15	0	1
12	3128	60	Placebo	1	61	2/15/2020	112.6	HEIGHT	16.35	14.3	0	1
12	3131	0	Placebo	0	36	3/12/2015	91.2	HEIGHT	14.05	15	0	1
12	3131	12	Placebo	0	54	7/16/2016	101.2	HEIGHT	16.7	15	0	1
12	3131	24	Placebo	0	62	5/14/2017	108.7	HEIGHT	18.72727273	15.5	0	1
12	3131	36	Placebo	0	75	6/20/2018	117.4	HEIGHT	20.85	15	0	1
12	3131	48	Placebo	0	86	5/17/2019	122.1	HEIGHT	22.75	15	0	1
12	3131	60	Placebo	0	95	2/15/2020	124.7	HEIGHT	24.25	15.5	0	1
12	3135	12	Placebo	0	35	7/16/2016	87.3	LENGTH	11.95	15.5	1	1
12	3135	24	Placebo	0	49	5/14/2017	94.2	HEIGHT	14.31818182	15.5	1	1
12	3138	24	Placebo	0	9	5/14/2017	69.9	HEIGHT	7.181818182	12	1	1
12	3138	36	Placebo	0	21	6/20/2018	82.5	HEIGHT	10.05	12.5	0	1
12	3138	48	Placebo	0	32	5/17/2019	87.7	HEIGHT	11.3	13	0	1
12	3138	60	Placebo	0	41	2/15/2020	93.7	HEIGHT	13	13.2	0	1
12	3139	0	Placebo	0	24	3/12/2015	78.1	HEIGHT	10.35	14.5	0	1
12	3139	12	Placebo	0	34	7/16/2016	87.7	HEIGHT	12.2	14	0	1
12	3139	24	Placebo	0	48	5/14/2017	94.6	HEIGHT	12.72727273	15	0	1
12	3139	36	Placebo	0	63	6/20/2018	101.4	HEIGHT	13.95	13.5	0	1
12	3139	48	Placebo	0	73	5/17/2019	107.1	HEIGHT	15.9	14	0	1
12	3139	60	Placebo	0	83	2/15/2020	105.4	HEIGHT	15.15	14	0	1

12	3141	0	Placebo	0	54	3/12/2015	105.2	HEIGHT	16.5	15.5	1	1
12	3141	12	Placebo	0	70	7/16/2016	112.9	HEIGHT	19.25	16	0	1
12	3142	12	Placebo	0	7	7/16/2016	63	LENGTH	6.3	13	1	1
12	3143	12	Placebo	0	49	7/16/2016	102.2	HEIGHT	15.8	15.5	1	1
12	3149	12	Placebo	0	34	7/16/2016	87.6	HEIGHT	10.85	13.5	0	1
12	3149	24	Placebo	0	48	5/14/2017	94.6	HEIGHT	12	14.5	0	1
12	3149	36	Placebo	0	60	6/20/2018	102.7	HEIGHT	13.5	13.5	0	1
12	3149	48	Placebo	0	71	5/17/2019	107.6	HEIGHT	14.85	14	0	1
12	3149	60	Placebo	0	80	2/15/2020	112.3	HEIGHT	16.6	14.2	0	1
12	3152	0	Placebo	1	48	3/12/2015	90	HEIGHT	12.45	14.5	1	1
12	3152	12	Placebo	1	61	7/16/2016	97.2	HEIGHT	13.3	14.5	0	1
12	3153	0	Placebo	0	36	3/12/2015	94	HEIGHT	13.85	15.5	0	1
12	3153	12	Placebo	0	46	7/16/2016	103	HEIGHT	14.55	14	0	1
12	3153	24	Placebo	0	60	5/14/2017	108	HEIGHT	15.40909091	14.5	0	1
12	3153	36	Placebo	0	72	6/20/2018	113.3	HEIGHT	17.05	13.5	0	1
12	3153	48	Placebo	0	83	5/17/2019	117.3	HEIGHT	18.2	14	0	1
12	3153	60	Placebo	0	92	2/15/2020	121.1	HEIGHT	19.7	13.7	0	1
12	3155	0	Placebo	0	36	3/12/2015	92.2	HEIGHT	14	15.5	0	1
12	3155	12	Placebo	0	54	7/16/2016	103.2	HEIGHT	16.65	15	0	1
12	3155	24	Placebo	0	68	5/14/2017	109	HEIGHT	17.77272727	15.5	0	1
12	3155	36	Placebo	0	80	6/20/2018	117	HEIGHT	20.9	15	0	1
12	3155	60	Placebo	0	100	2/15/2020	126.1	HEIGHT	24.9	16.5	0	1
12	3157	0	Placebo	1	48	3/12/2015	113.7	HEIGHT	19.5	15.5	0	1
12	3157	12	Placebo	1	58	7/16/2016	122	HEIGHT	21.65	16	0	1
12	3157	36	Placebo	1	84	6/20/2018	132.1	HEIGHT	26.8	17	0	1
12	3157	48	Placebo	1	95	5/17/2019	136	HEIGHT	28.35	18	0	1
12	3157	60	Placebo	1	104	2/15/2020	138.4	HEIGHT	31.2	18.2	0	1
12	3158	0	Placebo	0	24	3/12/2015	78.4	HEIGHT	9.3	14	1	1
12	3159	0	Placebo	1	36	3/12/2015	86.7	HEIGHT	12.35	15.5	1	1
12	3159	24	Placebo	1	60	5/14/2017	105.1	HEIGHT	16.40909091	16.5	0	1
12	3159	36	Placebo	1	72	6/20/2018	112.4	HEIGHT	18.25	16	0	1
12	3159	48	Placebo	1	79	5/17/2019	118.3	HEIGHT	19.25	15.5	0	1
12	3159	60	Placebo	1	89	2/15/2020	122.7	HEIGHT	21.55	16.2	0	1
12	3160	24	Placebo	0	56	5/14/2017	104.5	HEIGHT	16.31818182	15	1	1
12	3161	24	Placebo	0	14	5/14/2017	69.1	LENGTH	5.409090909	9.5	1	1
12	3163	0	Placebo	0	42	3/12/2015	96.6	HEIGHT	15	16.5	1	1
12	3163	12	Placebo	0	70	7/16/2016	104.7	HEIGHT	16.05	16	0	1
12	3163	24	Placebo	0	80	5/14/2017	110.3	HEIGHT	17.81818182	16.5	0	1
12	3163	48	Placebo	0	103	5/17/2019	117.4	HEIGHT	21.45	17.5	0	1
12	3164	12	Placebo	1	40	7/16/2016	104.9	HEIGHT	16.75	15.5	1	1

12	3166	0	Placebo	1	36	3/12/2015	82.6	LENGTH	11.6	15.5		0	1
12	3166	12	Placebo	0	42	7/16/2016	91.1	HEIGHT	14.2	16		0	1
12	3166	24	Placebo	0	56	5/14/2017	97.1	HEIGHT	14.54545455	16		0	1
12	3166	36	Placebo	1	71	6/20/2018	103.3	HEIGHT	16.2	15		0	1
12	3166	48	Placebo	1	82	5/17/2019	107.5	HEIGHT	17.45	16		0	1
12	3166	60	Placebo	1	92	2/15/2020	110	HEIGHT	18.65	15.5		0	1
12	3168	12	Placebo	1	42	7/16/2016	88.8	HEIGHT	12.3	15		1	1
12	3171	0	Placebo	1	36	3/12/2015	89.2	HEIGHT	11.35	14		1	1
12	3171	12	Placebo	1	48	7/16/2016	100.2	HEIGHT	12.9	13.5		1	1
12	3171	24	Placebo	1	62	5/14/2017	105.9	HEIGHT	14.27272727	13.5		0	1
12	3171	60	Placebo	1	95	2/15/2020	121.7	HEIGHT	20	15.4		0	1
12	3172	24	Placebo	0	1	5/14/2017	56	LENGTH	5.272727273	13		1	1
12	3172	36	Placebo	0	14	6/20/2018	70.5	LENGTH	6.5	11		0	1
12	3172	48	Placebo	0	25	5/17/2019	79.2	LENGTH	8.95	12		0	1
12	3173	0	Placebo	0	7	3/12/2015	73.3	LENGTH	9.7	15	54	0	1
12	3173	12	Placebo	0	30	7/16/2016	84.7	HEIGHT	12.2	16	54	0	1
12	3173	24	Placebo	0	44	5/14/2017	88.5	HEIGHT	12.68181818	15.5	54	0	1
12	3173	36	Placebo	0	56	6/20/2018	97	HEIGHT	16.2	16	54	1	1
12	3173	48	Placebo	0	67	5/17/2019	101.7	HEIGHT	17.55	15	54	0	1
12	3174	0	Placebo	1	24	3/12/2015	84.2	HEIGHT	11.3	14		0	1
12	3174	12	Placebo	1	42	7/16/2016	93.7	HEIGHT	12.75	15		0	1
12	3174	24	Placebo	1	56	5/14/2017	100.6	HEIGHT	14.54545455	15		0	1
12	3174	36	Placebo	1	70	6/20/2018	109.2	HEIGHT	16.25	15		0	1
12	3174	48	Placebo	1	80	5/17/2019	115.1	HEIGHT	18.8	15		0	1
12	3174	60	Placebo	1	90	2/15/2020	119.6	HEIGHT	20.45	15.5		0	1
12	3176	0	Placebo	0	36	3/12/2015	75.1	HEIGHT	9.35	13.5		0	1
12	3176	24	Placebo	0	56	5/14/2017	90.2	HEIGHT	12.77272727	14.5		0	1
12	3176	48	Placebo	0	68	5/17/2019	103.5	HEIGHT	16.65	15		0	1
12	3176	60	Placebo	0	77	2/15/2020	107.4	HEIGHT	17.85	15.5		0	1
12	3180	12	Placebo	1	30	7/16/2016	88.7	HEIGHT	13.75	16		1	1
12	3180	36	Placebo	1	57	6/20/2018	104	HEIGHT	16.45	16		1	1
12	3181	12	Placebo	0	5	7/16/2016	61.1	LENGTH	5.45	12		0	1
12	3181	24	Placebo	0	12	5/14/2017	70.2	HEIGHT	6.772727273	12		0	1
12	3184	0	Placebo	1	48	3/12/2015	95.4	HEIGHT	14.9	15		1	1
12	3184	24	Placebo	1	68	5/14/2017	109.2	HEIGHT	17.72727273	15.5		0	1
12	3184	48	Placebo	1	91	5/17/2019	120.9	HEIGHT	21.7	14.5		0	1
12	3184	60	Placebo	1	100	2/15/2020	124	HEIGHT	24.3	16.5		0	1
12	3185	0	Placebo	0	24	3/12/2015	89.3	HEIGHT	12.85	13.5		0	1
12	3185	12	Placebo	0	34	7/16/2016	98.1	HEIGHT	14.35	14		0	1
12	3185	36	Placebo	0	60	6/20/2018	110.9	HEIGHT	17	13.5		0	1

12	3185	48	Placebo	0	71	5/17/2019	116.8	HEIGHT	19.4	14		0	1
12	3185	60	Placebo	0	80	2/15/2020	120.3	HEIGHT	21.6	15.5		0	1
12	3186	0	Placebo	1	36	3/12/2015	86.7	HEIGHT	9.85	12		1	1
12	3186	12	Placebo	1	49	7/16/2016	94.7	HEIGHT	10.75	12		1	1
12	3186	24	Placebo	1	60	5/14/2017	101.4	HEIGHT	11.68181818	12.5		0	1
12	3186	36	Placebo	1	72	6/20/2018	109.1	HEIGHT	12.85	11.5		0	1
12	3186	48	Placebo	1	83	5/17/2019	114.3	HEIGHT	14.8	12.5		0	1
12	3188	12	Placebo	1	6	7/16/2016	67.4	LENGTH	8.55	15.5		0	1
12	3189	12	Placebo	1	13	7/16/2016	70.4	LENGTH	8	14.5	54	0	1
12	3190	12	Placebo	1	34	7/16/2016	75.5	HEIGHT	9.65	15		1	1
12	3191	12	Placebo	0	11	7/16/2016	76.2	LENGTH	8.5	13.5		1	1
12	3191	60	Placebo	0	56	2/15/2020	100.7	HEIGHT	15.7	14.8		1	1
12	3195	0	Placebo	0	11	3/12/2015	68.5	LENGTH	8.25	15		1	1
12	3195	12	Placebo	0	34	7/16/2016	80.7	HEIGHT	11.55	15.5		1	1
12	3195	24	Placebo	0	44	5/14/2017	89.4	HEIGHT	12.18181818	15.5		0	1
12	3195	36	Placebo	0	56	6/20/2018	98.1	HEIGHT	14.3	14.5		1	1
12	3195	48	Placebo	0	67	5/17/2019	103.3	HEIGHT	14.85	14		0	1
12	3195	60	Placebo	0	76	2/15/2020	108.1	HEIGHT	16.95	14.2		0	1
12	3196	0	Placebo	1	54	3/12/2015	107.4	HEIGHT	15.35	14.5		0	1
12	3196	12	Placebo	1	64	7/16/2016	115.4	LENGTH	16.4	15.7		0	1
12	3196	24	Placebo	1	78	5/14/2017	121.1	HEIGHT	18.27272727	15		0	1
12	3196	36	Placebo	1	90	6/20/2018	125.7	HEIGHT	20	15		0	1
12	3196	48	Placebo	1	101	5/17/2019	131.1	HEIGHT	21.4	15		0	1
12	3196	60	Placebo	1	110	2/15/2020	134.3	HEIGHT	23.7	15.5		0	1
12	3199	0	Placebo	0	24	3/12/2015	81.3	HEIGHT	10.1	13		0	1
12	3199	12	Placebo	0	34	7/16/2016	87.5	HEIGHT	11.95	15		0	1
12	3199	24	Placebo	0	48	5/14/2017	93.6	HEIGHT	13.36363636	14.5		0	1
12	3200	12	Placebo	1	12	7/16/2016	69.8	HEIGHT	7.85	13		1	1
12	3200	24	Placebo	1	24	5/14/2017	78.5	HEIGHT	9.454545455	13		1	1
12	3200	36	Placebo	1	37	6/20/2018	85.4	HEIGHT	11	13		0	1
12	3200	48	Placebo	1	47	5/17/2019	94.1	HEIGHT	12.45	13		0	1
12	3200	60	Placebo	1	57	2/15/2020	92.2	HEIGHT	13.4	12.2		1	1
12	3202	0	Placebo	1	18	3/12/2015	80.2	LENGTH	10.85	15.5		1	1
12	3202	12	Placebo	1	30	7/16/2016	87.8	HEIGHT	12.1	15		0	1
12	3202	24	Placebo	1	44	5/14/2017	94.9	HEIGHT	13.04545455	14.5		1	1
12	3202	36	Placebo	1	56	6/20/2018	102.5	HEIGHT	14.45	14		0	1
12	3202	48	Placebo	1	67	5/17/2019	107.8	HEIGHT	15.55	14		0	1
12	3202	60	Placebo	1	76	2/15/2020	113.2	HEIGHT	17.7	14.6		0	1
12	3204	0	Placebo	1	54	3/12/2015	107.5	HEIGHT	14.8	13.5		0	1
12	3204	24	Placebo	1	80	5/14/2017	120.7	HEIGHT	18.95454545	15		0	1

12	3204	60	Placebo	1	113	2/15/2020	133.5	HEIGHT	23.75	15.7			0	1
12	3208	0	Placebo	1	54	3/12/2015	99.7	HEIGHT	14.05	14			1	1
12	3208	12	Placebo	1	59	7/16/2016	107.7	HEIGHT	15.75	14			0	1
12	3208	36	Placebo	1	101	6/20/2018	116.9	HEIGHT	19.05	15			0	1
12	3208	48	Placebo	1	112	5/17/2019	120.6	HEIGHT	20.35	15			0	1
12	3208	60	Placebo	1	121	2/15/2020	122.8	HEIGHT	21.45	15.5			0	1
12	3210	0	Placebo	0	12	3/12/2015	72	LENGTH	8.7	14			0	1
12	3210	12	Placebo	0	22	7/16/2016	79.6	HEIGHT	9.5	14			0	1
12	3210	24	Placebo	0	36	5/14/2017	88.9	HEIGHT	11.22727273	14.5			1	1
12	3211	0	Placebo	1	24	3/12/2015	77	LENGTH	9.05	14			0	1
12	3211	12	Placebo	1	37	7/16/2016	85.9	HEIGHT	10.95	14.5			0	1
12	3211	24	Placebo	1	48	5/14/2017	92.5	HEIGHT	11.68181818	14.5			0	1
12	3211	36	Placebo	1	60	6/20/2018	98.4	HEIGHT	13.6	14			0	1
12	3212	0	Placebo	0	36	3/12/2015	94.2	HEIGHT	13.9	15			1	1
12	3212	24	Placebo	0	80	5/14/2017	109.2	HEIGHT	16.5	14.5			0	1
12	3212	36	Placebo	0	92	6/20/2018	116.3	HEIGHT	18	14.5			0	1
12	3212	60	Placebo	0	112	2/15/2020	125.9	HEIGHT	22.6	15.5			0	1
12	3214	12	Placebo	1	40	7/17/2016	85	HEIGHT	13	11.1			1	1
12	3214	24	Placebo	1	54	5/14/2017	99.6	HEIGHT	11.40909091	13			1	1
12	3215	12	Placebo	1	2	7/16/2016	63.4	LENGTH	6.55	13.5			0	1
12	3215	36	Placebo	1	28	6/20/2018	78.7	HEIGHT	10.95	14			0	1
12	3215	60	Placebo	1	48	2/15/2020	91.1	HEIGHT	13.8	15			0	1
12	3218	12	Placebo	0	46	7/16/2016	92	HEIGHT	13.9	15			1	1
12	3218	24	Placebo	0	60	5/14/2017	96.9	HEIGHT	14.81818182	14.5			0	1
12	3218	36	Placebo	0	65	6/20/2018	103.9	HEIGHT	16.45	14			0	1
12	3218	48	Placebo	0	82	5/17/2019	108.4	HEIGHT	17.3	14			0	1
12	3218	60	Placebo	0	91	2/15/2020	113.6	HEIGHT	18.9	14.6			0	1
12	3219	12	Placebo	0	58	7/17/2016	95	HEIGHT	15	13			1	1
12	3221	12	Placebo	0	9	7/16/2016	74.9	LENGTH	10.1	16.5			0	1
12	3221	24	Placebo	0	17	5/14/2017	82.4	HEIGHT	9.818181818	15			1	1
12	3221	60	Placebo	0	55	2/15/2020	98.4	HEIGHT	15.85	16			0	1
12	3222	0	Placebo	0	36	3/12/2015	97.6	HEIGHT	15.55	15.5			0	1
12	3222	12	Placebo	0	66	7/16/2016	107	HEIGHT	17.05	15			0	1
12	3222	24	Placebo	0	80	5/14/2017	111.8	HEIGHT	18.04545455	15.5			0	1
12	3222	36	Placebo	0	92	6/20/2018	118.5	HEIGHT	19.85	15			0	1
12	3222	48	Placebo	0	103	5/17/2019	121.6	HEIGHT	21.9	15.5			0	1
12	3224	0	Placebo	1	18	3/12/2015	72.3	LENGTH	7.8	12.5			0	1
12	3224	12	Placebo	1	42	7/16/2016	84.6	HEIGHT	12.15	15.5			1	1
12	3224	24	Placebo	1	56	5/14/2017	94	HEIGHT	14	15			1	1
12	3224	36	Placebo	1	68	6/20/2018	102.6	HEIGHT	14.9	14			0	1

12	3224	48	Placebo	1	79	5/17/2019	107.1	HEIGHT	16.15	14		0	1
12	3224	60	Placebo	1	88	2/15/2020	112.5	HEIGHT	18.5	15		0	1
12	3228	12	Placebo	1	42	7/16/2016	90.2	HEIGHT	11.9	14		1	1
12	3232	0	Placebo	0	48	3/12/2015	101.1	HEIGHT	14.95	15.5	18	1	1
12	3233	24	Placebo	0	14	5/14/2017	75.3	LENGTH	9.818181818	15		1	1
12	3235	12	Placebo	1	9	7/16/2016	67.3	LENGTH	7.15	13.5		0	1
12	3235	24	Placebo	1	17	5/14/2017	74.8	HEIGHT	7.590909091	12		1	1
12	3235	36	Placebo	1	29	6/20/2018	82.8	LENGTH	10.05	14		1	1
12	3236	24	Placebo	1	42	5/14/2017	95.4	HEIGHT	15.04545455	17		1	1
12	3236	36	Placebo	1	54	6/20/2018	101.4	HEIGHT	15.95	16		1	1
12	3237	0	Placebo	0	24	3/12/2015	75.3	HEIGHT	8.75	12.5		0	1
12	3237	24	Placebo	0	60	5/14/2017	98.1	HEIGHT	12.31818182	14		0	1
12	3240	0	Placebo	1	48	3/12/2015	80.8	HEIGHT	12.3	15.5		0	1
12	3243	24	Placebo	0	10	5/14/2017	69.8	LENGTH	6.863636364	13.5		1	1
12	3243	36	Placebo	0	21	6/20/2018	78.3	LENGTH	7.35	12		0	1
12	3243	48	Placebo	0	32	5/17/2019	83	HEIGHT	9.15	13		0	1
12	3243	60	Placebo	0	41	2/15/2020	89.8	HEIGHT	10.35	13.8		0	1
12	3244	0	Placebo	0	8	3/12/2015	64.7	LENGTH	5.85	12		1	1
12	3244	24	Placebo	0	32	5/14/2017	81	HEIGHT	10	13.5		1	1
12	3244	36	Placebo	0	44	6/20/2018	88.5	HEIGHT	11.1	13		0	1
12	3244	48	Placebo	0	55	5/17/2019	95.1	HEIGHT	12.65	13		1	1
12	3245	0	Placebo	0	36	3/12/2015	87	HEIGHT	11.2	13.5		1	1
12	3245	12	Placebo	0	54	7/16/2016	96.5	HEIGHT	12.35	13.5		0	1
12	3245	24	Placebo	0	68	5/14/2017	103.3	HEIGHT	12.90909091	12.5		0	1
12	3245	36	Placebo	0	80	6/20/2018	110	HEIGHT	14.45	12.5		0	1
12	3245	48	Placebo	0	91	5/17/2019	113.7	HEIGHT	15.8	13		0	1
12	3245	60	Placebo	0	100	2/15/2020	119.3	HEIGHT	17.45	13.2		0	1
12	3246	0	Placebo	1	54	3/12/2015	108.4	HEIGHT	16.75	16		0	1
12	3246	36	Placebo	1	116	6/20/2018	125.7	HEIGHT	21.45	16		0	1
12	3246	60	Placebo	1	136	2/15/2020	131.6	HEIGHT	25.75	17.5		0	1
12	3250	24	Placebo	1	9	5/14/2017	70.5	LENGTH	8.318181818	15.5		1	1
12	3250	36	Placebo	1	21	6/20/2018	85.2	HEIGHT	11.25	15		1	1
12	3250	48	Placebo	1	32	5/17/2019	90.6	HEIGHT	12.65	15		0	1
12	3250	60	Placebo	1	41	2/15/2020	97	HEIGHT	14.2	15		0	1
12	3251	12	Placebo	0	11	7/16/2016	72.7	HEIGHT	7.95	13		0	1
12	3251	36	Placebo	0	35	6/20/2018	86.7	HEIGHT	11.35	13.5		0	1
12	3251	60	Placebo	0	56	2/15/2020	99.3	HEIGHT	13.7	14.5		1	1
12	3252	24	Placebo	0	25	5/14/2017	75.8	LENGTH	7.636363636	12		1	1
12	3252	48	Placebo	0	49	5/17/2019	102.2	HEIGHT	16.3	16		1	1
12	3254	0	Placebo	1	9	3/12/2015	66.8	LENGTH	6.75	13.5		0	1

12	3254	12	Placebo	1	19	7/16/2016	79.8	HEIGHT	8.95	13.5	0	1
12	3254	24	Placebo	1	30	5/14/2017	86.5	HEIGHT	10.68181818	13.5	0	1
12	3254	36	Placebo	1	47	6/20/2018	91.2	HEIGHT	10.15	11	0	1
12	3254	48	Placebo	1	57	5/17/2019	94.4	HEIGHT	11.9	13	0	1
12	3255	0	Placebo	1	5	3/12/2015	60.5	LENGTH	6.8	14.5	1	1
12	3255	12	Placebo	1	12	7/16/2016	72.6	HEIGHT	8.4	12.5	0	1
12	3255	24	Placebo	1	25	5/14/2017	79	HEIGHT	10.27272727	14.5	0	1
12	3255	36	Placebo	1	38	6/20/2018	88.8	HEIGHT	12.6	15	0	1
12	3255	48	Placebo	1	49	5/17/2019	95.3	HEIGHT	14	15	0	1
12	3255	60	Placebo	1	58	2/15/2020	101	HEIGHT	15.4	14.6	0	1
12	3259	12	Placebo	1	7	7/16/2016	70.4	LENGTH	7.35	12.5	0	1
12	3259	24	Placebo	1	17	5/14/2017	77.3	HEIGHT	8.954545455	13.5	1	1
12	3259	48	Placebo	1	40	5/17/2019	88.9	HEIGHT	13.45	15	1	1
12	8057	48	Placebo	1	10	5/17/2019	71.8	LENGTH	7.5	12.5	1	1
12	8077	48	Placebo	0	19	5/17/2019	73.3	HEIGHT	8.52	13.5	1	1
12	8077	60	Placebo	0	28	2/15/2020	81.9	HEIGHT	11.05	15	1	1
12	8084	48	Placebo	1	16	5/17/2019	69.4	LENGTH	7.7	13	1	1
12	8143	60	Placebo	1	36	2/15/2020	92.4	HEIGHT	11.6	14.2	1	1
12	8149	60	Placebo	1	35	2/15/2020	88.6	HEIGHT	11.6	13.4	1	1
12	8159	36	Placebo	0	56	6/20/2018	99.8	HEIGHT	14.25	14.5	1	1
12	8173	60	Placebo	0	19	2/15/2020	81.6	HEIGHT	9.3	12.5	1	1
12	8232	60	Placebo	0	23	2/15/2020	81.9	HEIGHT	9.7	13	1	1
12	8233	48	Placebo	0	40	5/17/2019	94	HEIGHT	14	15	1	1
12	8236	36	Placebo	1	16	6/20/2018	74.5	LENGTH	9.75	14	1	1
12	8241	36	Placebo	0	26	6/20/2018	79	HEIGHT	10.2	13	1	1
12	8299	36	Placebo	0	34	6/20/2018	89.4	HEIGHT	11.8	13.5	1	1
12	8303	60	Placebo	1	8	2/20/2020	66.3	LENGTH	6.772727273	12	1	1
12	8310	48	Placebo	0	45	5/17/2019	96.4	HEIGHT	13.8	15	1	1
12	8368	48	Placebo	0	55	5/17/2019	93.9	HEIGHT	11.3	13	1	1
12	8373	36	Placebo	1	15	6/20/2018	75.1	LENGTH	9.1	14	1	1
12	8394	60	Placebo	1	35	2/15/2020	84.3	HEIGHT	9.65	13.8	1	1
12	8412	60	Placebo	0	54	2/15/2020	99.9	HEIGHT	16.65	15.2	1	1
12	8424	48	Placebo	0	2	5/17/2019	63.3	LENGTH	7.6	15	1	1
12	8424	60	Placebo	0	11	2/15/2020	77.6	LENGTH	10.9	18.5	0	1
12	8439	48	Placebo	1	57	5/17/2019	107	HEIGHT	16.8	15	1	1
12	8479	48	Placebo	0	28	5/17/2019	92.6	HEIGHT	13.65	15.5	1	1
12	8479	60	Placebo	0	37	2/15/2020	97.6	HEIGHT	16.65	16.6	1	1
12	8493	48	Placebo	1	34	5/17/2019	79.1	HEIGHT	10.4	14.5	1	1
12	8493	60	Placebo	1	43	2/20/2020	87.7	HEIGHT	12	14	1	1
12	8503	48	Placebo	0	12	5/17/2019	74.4	HEIGHT	7.95	13	1	1

12	8589	60	Placebo	1	14	2/15/2020	77.9	LENGTH	9.45	14	1	1
12	8593	36	Placebo	1	9	6/20/2018	66.4	LENGTH	7.2	13	1	1
12	8593	48	Placebo	1	20	5/17/2019	79.1	LENGTH	10	14.5	1	1
12	8607	48	Placebo	0	6	5/17/2019	69.7	LENGTH	8.3	14	1	1
12	8608	48	Placebo	1	8	5/17/2019	64.6	LENGTH	5.05	10.5	1	1
12	8608	60	Placebo	1	14	2/15/2020	71.8	LENGTH	6.6	11	0	1
12	8671	36	Placebo	0	9	6/20/2018	67.3	HEIGHT	6.6	12	1	1
12	8671	48	Placebo	0	20	5/17/2019	75.2	HEIGHT	8.75	12.5	0	1
12	8671	60	Placebo	0	29	2/15/2020	80	LENGTH	10.6	12.8	0	1
12	8691	48	Placebo	0	16	5/17/2019	72.2	LENGTH	8.1	13	1	1
12	8697	36	Placebo	0	58	6/20/2018	98.5	HEIGHT	14.3	14	1	1
12	8698	36	Placebo	0	6	6/20/2018	64.1	LENGTH	6.9	12.5	1	1
12	8698	48	Placebo	0	15	5/17/2019	79.5	HEIGHT	10.7	15	0	1
12	8712	48	Placebo	1	45	5/17/2019	91	HEIGHT	12.1	14	1	1
12	8760	36	Placebo	1	21	6/20/2018	78.7	HEIGHT	9.3	13.5	1	1
12	8760	48	Placebo	1	32	5/17/2019	86.1	HEIGHT	11.2	15	1	1
12	8783	48	Placebo	0	45	5/17/2019	96.3	HEIGHT	14.15	14	1	1
12	8783	60	Placebo	0	55	2/15/2020	101.6	HEIGHT	15.8	14.2	1	1
12	8791	60	Placebo	0	11	2/15/2020	64.3	LENGTH	6.75	12.5	1	1
12	8796	48	Placebo	1	12	5/17/2019	71.6	LENGTH	7.6	13	1	1
12	8806	36	Placebo	1	13	6/20/2018	75.5	LENGTH	7.8	12	1	1
12	8808	36	Placebo	1	27	6/20/2018	83	HEIGHT	10.7	14	1	1
12	8854	48	Placebo	1	16	5/17/2019	78.8	HEIGHT	10.3	14.5	1	1
12	8895	36	Placebo	1	2	6/20/2018	58.2	LENGTH	4.4	10	1	1
12	8895	48	Placebo	1	12	5/17/2019	70.5	HEIGHT	6.4	11	0	1
12	8895	60	Placebo	1	22	2/15/2020	78.9	HEIGHT	8.85	11.5	1	1
12	8901	60	Placebo	0	31	2/15/2020	85.4	LENGTH	12	14.3	1	1
12	8926	48	Placebo	0	55	5/17/2019	98.7	HEIGHT	16.55	15.5	1	1
12	8971	36	Placebo	1	15	6/20/2018	77.6	HEIGHT	8.75	12	1	1
12	8977	60	Placebo	0	20	2/15/2020	74.2	HEIGHT	8.75	13.5	1	1
12	9000	60	Placebo	1	46	2/15/2020	87.6	HEIGHT	11.5	13	1	1
12	9010	60	Placebo	0	10	2/15/2020	70.9	LENGTH	7.85	13	1	1
12	9011	36	Placebo	1	2	6/20/2018	59.3	LENGTH	5.55	12	1	1
12	9011	48	Placebo	1	13	5/17/2019	73.7	LENGTH	8.6	13	0	1
12	9011	60	Placebo	1	23	2/15/2020	80.9	HEIGHT	10.45	12.9	0	1
12	9012	60	Placebo	0	21	2/15/2020	80.8	HEIGHT	10.5	15.3	1	1
12	9041	36	Placebo	0	24	6/20/2018	82.9	HEIGHT	10.25	13.5	1	1
12	9053	60	Placebo	0	47	2/20/2020	88.3	HEIGHT	12.72727273	15	1	1
12	9070	60	Placebo	0	32	2/15/2020	81	HEIGHT	11.4	14	1	1
12	9092	60	Placebo	0	58	2/15/2020	106.3	HEIGHT	17.85	16	1	1

12	9107	36	Placebo	1	11	6/20/2018	71.3	HEIGHT	7.2	12	1	1
12	9107	60	Placebo	1	31	2/15/2020	85.6	HEIGHT	10.8	13.5	0	1
12	9111	60	Placebo	1	9	2/15/2020	70.9	LENGTH	8.6	13.8	1	1
12	9131	36	Placebo	0	9	6/20/2018	69.6	LENGTH	8.5	14	1	1
12	9131	60	Placebo	0	27	2/15/2020	83.5	HEIGHT	13.5	15	1	1
12	9157	60	Placebo	1	48	2/15/2020	89.6	HEIGHT	12.85	15.2	1	1
12	9168	36	Placebo	1	25	6/20/2018	80.4	HEIGHT	9.1	13	1	1
12	9207	60	Placebo	1	59	2/15/2020	99.6	HEIGHT	16.85	16	1	1
12	9218	48	Placebo	1	57	5/17/2019	107.9	HEIGHT	15.4	13	1	1
12	9232	36	Placebo	1	9	6/20/2018	61.5	LENGTH	6.2	13	1	1
12	9232	60	Placebo	1	25	2/15/2020	80	HEIGHT	10.35	13.4	0	1
12	9291	48	Placebo	0	32	5/17/2019	82.7	HEIGHT	11.2	16	1	1
12	9303	36	Placebo	1	48	6/20/2018	82.8	HEIGHT	9.45	12.5	1	1
12	9315	60	Placebo	1	14	2/15/2020	70.4	LENGTH	7.9	12.2	1	1
12	9330	60	Placebo	0	56	2/15/2020	104.5	HEIGHT	16.9	14.7	1	1
12	9334	60	Placebo	0	26	2/15/2020	78.5	HEIGHT	8.75	12	1	1
12	9351	48	Placebo	1	22	5/17/2019	79.6	HEIGHT	9.7	12.5	1	1
12	9384	48	Placebo	1	47	5/17/2019	98.8	HEIGHT	13.55	13.5	1	1
12	9395	60	Placebo	0	20	2/15/2020	75.6	HEIGHT	9	14.3	1	1
12	9438	36	Placebo	1	44	6/20/2018	83.9	HEIGHT	7.85	10	1	1
12	9454	36	Placebo	1	12	6/20/2018	71.7	LENGTH	7.95	13	1	1
12	9454	48	Placebo	1	22	5/17/2019	74.5	LENGTH	7.05	10.5	1	1
12	9454	60	Placebo	1	32	2/15/2020	83.8	HEIGHT	11.65	14	0	1
12	9490	60	Placebo	0	12	2/15/2020	71.1	LENGTH	7.95	12.2	1	1
12	9533	60	Placebo	1	13	2/15/2020	70.2	LENGTH	6.8	11.9	1	1
12	9539	60	Placebo	1	13	2/20/2020	72.2	LENGTH	9	13	1	1
12	9560	36	Placebo	1	3	6/20/2018	60	LENGTH	5.7	11.5	1	1
12	9560	48	Placebo	1	12	5/17/2019	72.1	LENGTH	7.65	12.5	1	1
12	9560	60	Placebo	1	21	2/15/2020	78.7	HEIGHT	10.05	12.7	0	1
12	9572	48	Placebo	1	22	5/17/2019	78.6	HEIGHT	8.95	13	1	1
12	9572	60	Placebo	1	31	2/15/2020	83.7	HEIGHT	11.55	13.5	1	1
13	3273	0	Placebo	1	42	3/22/2015	92.1	HEIGHT	12.1	14.5	0	1
13	3273	12	Placebo	1	53	7/24/2016	101.3	HEIGHT		14.7	0	1
13	3273	36	Placebo	1	76	5/17/2018	111.9	HEIGHT	16.09090909	14.5	0	1
13	3276	0	Placebo	0	48	3/22/2015	94.7	HEIGHT	14.95	15.5	0	1
13	3276	12	Placebo	0	66	7/24/2016	104.2	HEIGHT		15.6	0	1
13	3276	24	Placebo	0	77	3/29/2017	109	HEIGHT	19.1	15.5	0	1
13	3276	36	Placebo	0	92	5/17/2018	114.8	HEIGHT	21.9	15.5	0	1
13	3276	48	Placebo	0	103	4/25/2019	118.6	HEIGHT	22.85	16	0	1
13	3276	60	Placebo	0	112	2/15/2020	122.8	HEIGHT	25.95	16.5	0	1

13	3279	12	Placebo	0	8	7/24/2016	65.2	HEIGHT		12	24	0	1
13	3280	12	Placebo	0	35	7/24/2016	98.5	HEIGHT		15.8		1	1
13	3282	0	Placebo	0	36	3/22/2015	92	HEIGHT	12.95	15.5	18	0	1
13	3284	12	Placebo	0	9	7/24/2016	76	HEIGHT		14.5		0	1
13	3285	0	Placebo	0	11	3/22/2015	72.3	LENGTH	7.55	14		0	1
13	3285	12	Placebo	0	33	7/24/2016	83.2	HEIGHT		13.5		0	1
13	3285	24	Placebo	0	41	3/29/2017	88.4	HEIGHT	10.05	13		0	1
13	3285	36	Placebo	0	56	5/17/2018	94.5	HEIGHT	11.5	13		0	1
13	3285	48	Placebo	0	67	4/25/2019	101.1	HEIGHT	12.25	13		0	1
13	3285	60	Placebo	0	76	2/15/2020	106.4	HEIGHT	13.9	13		0	1
13	3286	12	Placebo	1	6	7/24/2016	71.5	HEIGHT		15		1	1
13	3286	36	Placebo	1	29	5/17/2018	91.6	HEIGHT	14.1	15		0	1
13	3286	48	Placebo	1	40	4/25/2019	98	HEIGHT	15.2	15.5		1	1
13	3286	60	Placebo	1	50	2/15/2020	103.8	HEIGHT	16.45	15		0	1
13	3288	0	Placebo	0	18	3/22/2015	80.7	HEIGHT	10.5	15.5		1	1
13	3288	12	Placebo	0	29	7/24/2016	90.7	HEIGHT		16.6		0	1
13	3288	24	Placebo	0	37	3/29/2017	93.3	HEIGHT	14.2	15		0	1
13	3288	36	Placebo	0	52	5/17/2018	100.6	HEIGHT	16.36363636	16		0	1
13	3288	60	Placebo	0	61	2/15/2020	108.9	HEIGHT	19.7	15.8		0	1
13	3289	12	Placebo	1	8	7/25/2016	73.4	HEIGHT		13.6		0	1
13	3289	24	Placebo	1	14	3/29/2017	79.8	HEIGHT	9.55	13		0	1
13	3291	24	Placebo	0	9	3/29/2017	72.7	LENGTH	7.7	13.5		1	1
13	3293	12	Placebo	1	3	7/24/2016	59.6	HEIGHT		12.8		0	1
13	3293	24	Placebo	1	12	3/29/2017	74.7	HEIGHT	7.9	12		0	1
13	3293	36	Placebo	1	26	5/17/2018	87.1	HEIGHT	10.35	13		1	1
13	3293	60	Placebo	1	47	2/15/2020	99.5	HEIGHT	13.75	14		1	1
13	3294	0	Placebo	1	30	3/22/2015	84.8	LENGTH	12.1	15.5		0	1
13	3294	24	Placebo	1	41	3/31/2017	99.4	HEIGHT	17.15	17		1	1
13	3294	48	Placebo	1	67	4/25/2019	113.1	HEIGHT	20.05	15.5		0	1
13	3294	60	Placebo	1	76	2/15/2020	116.4	LENGTH	22	16		0	1
13	3295	0	Placebo	0	30	3/22/2015	91.6	HEIGHT	12.65	15		1	1
13	3295	12	Placebo	0	41	7/24/2016	100	HEIGHT		16.8		0	1
13	3295	36	Placebo	0	64	5/17/2018	110.5	HEIGHT	17	14.5		0	1
13	3295	48	Placebo	0	75	4/25/2019	114.7	HEIGHT	19.05	15.5		0	1
13	3295	60	Placebo	0	84	2/15/2020	118.5	HEIGHT	21.35	15.4		0	1
13	3296	12	Placebo	1	3	7/24/2016	64.3	HEIGHT		13.6	18	1	1
13	3297	0	Placebo	1	12	3/22/2015	72.1	LENGTH	7.15	13		1	1
13	3297	12	Placebo	1	34	7/24/2016	84.8	HEIGHT		12.8		1	1
13	3297	24	Placebo	1	41	3/29/2017	89.5	HEIGHT	10.45	12		0	1
13	3297	48	Placebo	1	62	4/25/2019	101.4	HEIGHT	12.7	12.5		0	1

13	3297	60	Placebo	1	71	2/15/2020	105.9	HEIGHT	13.75	12.7		0	1
13	3298	0	Placebo	1	54	3/22/2015	106.9	HEIGHT	15.35	15.5		0	1
13	3298	12	Placebo	1	66	7/24/2016	115	HEIGHT		16.3		0	1
13	3298	24	Placebo	1	77	3/29/2017	119.5	HEIGHT	19.8	16		0	1
13	3298	36	Placebo	1	101	5/17/2018	125.3	HEIGHT	21.77272727	17		0	1
13	3298	48	Placebo	1	112	4/25/2019	130.1	HEIGHT	24	17.5		0	1
13	3298	60	Placebo	1	121	2/15/2020	132.5	HEIGHT	26.1	18.3		0	1
13	3299	24	Placebo	1	6	3/29/2017	66.9	LENGTH	8.35	16	42	1	1
13	3300	0	Placebo	1	24	3/22/2015	82.7	HEIGHT	9.85	14.5		0	1
13	3300	12	Placebo	1	36	7/24/2016	93.6	HEIGHT		15.6		0	1
13	3300	36	Placebo	1	58	5/17/2018	103.6	HEIGHT	14.59090909	15		0	1
13	3300	60	Placebo	1	78	2/15/2020	114.2	HEIGHT	18.15	14.7		0	1
13	3301	0	Placebo	1	48	3/22/2015	98.7	HEIGHT	13.95	15.5		0	1
13	3301	12	Placebo	1	59	7/24/2016	109	HEIGHT		15.6		0	1
13	3301	48	Placebo	1	93	4/25/2019	123.5	HEIGHT	21.15	16.5		0	1
13	3303	12	Placebo	1	30	7/24/2016	107.7	HEIGHT		16		1	1
13	3304	12	Placebo	1	5	7/24/2016	67	HEIGHT		14.9		0	1
13	3304	24	Placebo	1	14	3/29/2017	75.8	HEIGHT	9.75	15.5		0	1
13	3304	36	Placebo	1	29	5/17/2018	83.5	HEIGHT	10.7	15		0	1
13	3304	60	Placebo	1	50	2/20/2020	98.4	HEIGHT	16.5	16.5		0	1
13	3305	12	Placebo	0	57	7/24/2016	118.3	HEIGHT		14.5		1	1
13	3306	0	Placebo	1	24	3/22/2015	78.3	LENGTH	9.7	14.5		0	1
13	3306	12	Placebo	1	36	7/24/2016	88	HEIGHT		15		0	1
13	3306	24	Placebo	1	42	3/29/2017	91	HEIGHT	12.15	14		0	1
13	3307	0	Placebo	0	2	3/22/2015	56.6	LENGTH	5.6	13.5		0	1
13	3307	24	Placebo	0	25	3/29/2017	79.8	HEIGHT	9.35	13.5		0	1
13	3307	36	Placebo	0	40	5/17/2018	83.9	HEIGHT	11.7	15.5		0	1
13	3307	48	Placebo	0	51	4/25/2019	89.7	HEIGHT	13	15.5		0	1
13	3307	60	Placebo	0	60	2/15/2020	93.7	HEIGHT	14.05	15		0	1
13	3311	12	Placebo	1	57	7/25/2016	119.3	HEIGHT		17.2		1	1
13	3313	0	Placebo	0	36	3/22/2015	94	HEIGHT	12.1	13		1	1
13	3313	24	Placebo	0	55	3/29/2017	107.3	HEIGHT	15.75	13.5		0	1
13	3313	36	Placebo	0	70	5/17/2018	113.2	HEIGHT	17.54545455	14		0	1
13	3313	48	Placebo	0	81	4/25/2019	118	HEIGHT	19.3	15		0	1
13	3313	60	Placebo	0	90	2/15/2020	122.2	HEIGHT	21.15	15		0	1
13	3315	12	Placebo	0	57	7/24/2016	109	HEIGHT		13.7		1	1
13	3316	0	Placebo	1	3	3/22/2015	60.1	LENGTH	6.2	13.5		1	1
13	3316	12	Placebo	1	17	7/24/2016	78.3	HEIGHT		15.5		0	1
13	3316	24	Placebo	1	25	3/29/2017	83	HEIGHT	10.1	14		0	1
13	3316	60	Placebo	1	50	2/15/2020	103.9	HEIGHT	16.45	14.7		0	1

13	3320	12	Placebo	0	59	7/24/2016	104.4	HEIGHT		15.6		1	1
13	3321	0	Placebo	0	24	3/22/2015	83.5	HEIGHT	12.15	16		0	1
13	3321	36	Placebo	0	64	5/17/2018	103.7	HEIGHT	17.09090909	16		0	1
13	3321	48	Placebo	0	75	4/25/2019	107.2	HEIGHT	19.05	16		0	1
13	3322	0	Placebo	0	48	3/22/2015	103.8	HEIGHT	14.9	14		1	1
13	3326	0	Placebo	1	48	3/22/2015	107.2	HEIGHT	16	14.5		1	1
13	3326	12	Placebo	1	56	7/24/2016	114	HEIGHT		15.6		0	1
13	3326	48	Placebo	1	98	4/25/2019	127.6	HEIGHT	24.85	16.5		0	1
13	3326	60	Placebo	1	107	2/15/2020	133.1	HEIGHT	25.45	16.5		0	1
13	3327	0	Placebo	0	36	3/22/2015	93.4	HEIGHT	14.25	14		1	1
13	3327	12	Placebo	0	44	7/24/2016	101.5	HEIGHT		13.8		0	1
13	3327	36	Placebo	0	78	5/17/2018	111.6	HEIGHT	19.5	14.5		0	1
13	3327	48	Placebo	0	88	4/25/2019	117	HEIGHT	21.2	14		0	1
13	3327	60	Placebo	0	98	2/15/2020	121.9	HEIGHT	22.7	14.2		0	1
13	3328	24	Placebo	1	14	3/31/2017	78.3	HEIGHT	9.45	15	30	1	1
13	3330	0	Placebo	1	12	3/22/2015	72.2	LENGTH	8.25	14		0	1
13	3330	36	Placebo	1	46	5/17/2018	96.9	HEIGHT	14.09090909	14.5		0	1
13	3330	48	Placebo	1	57	4/25/2019	104.6	HEIGHT	15.6	14.5		0	1
13	3330	60	Placebo	1	66	2/15/2020	107.2	HEIGHT	16.55	13.9		0	1
13	3331	24	Placebo	0	31	3/29/2017	89.4	HEIGHT	12.3	15		1	1
13	3331	36	Placebo	0	51	5/17/2018	97.2	HEIGHT	13.25	14		1	1
13	3332	24	Placebo	0	48	3/29/2017	101.2	HEIGHT	15.85	14.5		1	1
13	3334	0	Placebo	1	36	3/22/2015	92.5	HEIGHT	14.25	15.5		0	1
13	3334	12	Placebo	1	42	7/24/2016	103.3	HEIGHT		15.8		0	1
13	3334	24	Placebo	1	53	3/29/2017	108.4	HEIGHT	18.25	15		1	1
13	3334	36	Placebo	1	68	5/17/2018	113.6	HEIGHT	18.8	15		0	1
13	3338	24	Placebo	1	55	3/29/2017	103.5	HEIGHT	15.8	14.5		1	1
13	3340	12	Placebo	1	3	7/24/2016	68.6	HEIGHT		13.1	24	0	1
13	3343	12	Placebo	1	10	7/25/2016	73.4	HEIGHT		13.5		0	1
13	3343	24	Placebo	1	19	3/29/2017	77.9	HEIGHT	8.4	12.5		0	1
13	3343	36	Placebo	1	48	5/17/2018	88.8	HEIGHT	11.90909091	14		0	1
13	3343	60	Placebo	1	68	2/15/2020	102.9	HEIGHT	15.2	14.6		0	1
13	3344	0	Placebo	1	18	3/22/2015	80.8	HEIGHT	9.75	14	18	1	1
13	3345	0	Placebo	1	48	3/22/2015	100	HEIGHT	15.7	15.5		0	1
13	3345	12	Placebo	1	58	7/24/2016	107.7	HEIGHT		15.8		0	1
13	3345	24	Placebo	1	66	3/29/2017	110	HEIGHT	19.4	15.5		0	1
13	3345	48	Placebo	1	92	4/25/2019	118.5	HEIGHT	22.4	16.5		0	1
13	3345	60	Placebo	1	102	2/15/2020	122.5	HEIGHT	24.65	17		0	1
13	3346	0	Placebo	0	36	3/22/2015	96.5	HEIGHT	13.15	14.5		1	1
13	3346	12	Placebo	0	70	7/24/2016	103.5	HEIGHT		13.5		0	1

13	3346	24	Placebo	0	77	3/29/2017	106.4	HEIGHT	14.65	12.5		0	1
13	3346	36	Placebo	0	87	5/17/2018	112.4	HEIGHT	15.81818182	13		0	1
13	3346	48	Placebo	0	98	4/25/2019	116.8	HEIGHT	17.7	13.5		0	1
13	3346	60	Placebo	0	108	2/15/2020	120.6	HEIGHT	18.5	13.5		0	1
13	3347	0	Placebo	1	24	3/22/2015	80.5	HEIGHT	9.9	13.5		1	1
13	3347	12	Placebo	1	35	7/24/2016	92.9	HEIGHT		13.5		0	1
13	3347	24	Placebo	1	43	3/29/2017	96.5	HEIGHT	12.7	13.5		0	1
13	3347	36	Placebo	1	58	5/17/2018	104.4	HEIGHT	14	13		0	1
13	3347	48	Placebo	1	69	4/25/2019	110.7	HEIGHT	15.25	13.5		0	1
13	3350	24	Placebo	1	29	3/29/2017	84.5	HEIGHT	10.6	13		1	1
13	3350	36	Placebo	1	44	5/17/2018	92.6	HEIGHT	12.46	13.5		1	1
13	3351	12	Placebo	0	7	7/24/2016	70.2	HEIGHT		15.4		1	1
13	3351	36	Placebo	0	29	5/17/2018	84.5	HEIGHT	12.31818182	14.5		0	1
13	3351	48	Placebo	0	40	4/25/2019	90.6	HEIGHT	13.4	15		0	1
13	3351	60	Placebo	0	40	2/15/2020	96.5	HEIGHT	15.7	15.5		1	1
13	3352	0	Placebo	0	48	3/22/2015	102.1	HEIGHT	16.3	15		0	1
13	3353	12	Placebo	0	6	7/25/2016	68.8	LENGTH		14.8		0	1
13	3353	24	Placebo	0	13	3/29/2017	75.3	HEIGHT	9.05	14		0	1
13	3354	12	Placebo	1	32	7/25/2016	98	HEIGHT		15.6		1	1
13	3354	24	Placebo	1	50	3/29/2017	102.3	HEIGHT	15.95	15		1	1
13	3356	0	Placebo	1	36	3/22/2015	91.1	HEIGHT	13.65	15.5		0	1
13	3356	24	Placebo	1	53	3/29/2017	105.3	HEIGHT	16.7	14.5		1	1
13	3359	0	Placebo	0	2	3/22/2015	57.4	LENGTH	6.15	15.5	18	1	1
13	3360	0	Placebo	1	18	3/22/2015	76.9	LENGTH	9.85	14.5		0	1
13	3360	12	Placebo	1	34	7/24/2016	88.4	HEIGHT		15.2		0	1
13	3360	24	Placebo	1	41	3/29/2017	92.4	HEIGHT	13.8	14.5		0	1
13	3360	36	Placebo	1	56	5/17/2018	99.5	HEIGHT	15	15		1	1
13	3361	12	Placebo	1	42	7/25/2016	94.6	HEIGHT		12		1	1
13	3363	0	Placebo	1	10	3/22/2015	102.4	HEIGHT	16.05	15.5		0	1
13	3363	24	Placebo	1	65	3/29/2017	113.7	HEIGHT	19.6	16.5		0	1
13	3363	36	Placebo	1	80	5/17/2018	118.5	HEIGHT	21.05	16		0	1
13	3363	48	Placebo	1	91	4/25/2019	122.3	HEIGHT	22.35	17		0	1
13	3363	60	Placebo	1	100	2/15/2020	126	HEIGHT	24.3	17		0	1
13	3364	24	Placebo	0	31	3/29/2017	91.7	HEIGHT	12.85	13.5		1	1
13	3364	48	Placebo	0	57	4/25/2019	107.2	HEIGHT	17.4	14		1	1
13	3367	12	Placebo	0	12	7/24/2016	75.8	HEIGHT		14.2		1	1
13	3367	24	Placebo	0	19	3/29/2017	83	HEIGHT	11.6	14		0	1
13	3367	36	Placebo	0	39	5/17/2018	91.6	HEIGHT	14.18181818	15.5		0	1
13	3367	48	Placebo	0	50	4/25/2019	97.5	HEIGHT	15.6	15		0	1
13	3368	0	Placebo	1	48	3/22/2015	115.7	HEIGHT	17.25	13.5		1	1

13	3368	12	Placebo	1	56	7/24/2016	121.7	HEIGHT		15.3		0	1
13	3368	36	Placebo	1	82	5/17/2018	130.3	HEIGHT	22.86363636	15.5		0	1
13	3368	60	Placebo	1	102	2/15/2020	140	HEIGHT	28.5	17		0	1
13	3369	0	Placebo	1	48	3/22/2015	102.8	HEIGHT	14.7	15		0	1
13	3369	12	Placebo	1	56	7/24/2016	110.6	HEIGHT		15.8		0	1
13	3369	36	Placebo	1	82	5/17/2018	123.4	HEIGHT	20.6	15.5		0	1
13	3369	48	Placebo	1	93	4/25/2019	127.8	HEIGHT	22.75	15.5		0	1
13	3372	0	Placebo	0	36	3/22/2015	94.7	HEIGHT	14.35	15		0	1
13	3372	12	Placebo	0	33	7/24/2016	104.4	HEIGHT		15.1		0	1
13	3372	48	Placebo	0	97	4/25/2019	117.4	HEIGHT	21	16		0	1
13	3373	0	Placebo	0	36	6/11/2015	91.2	HEIGHT	10.85	12.5		0	1
13	3374	0	Placebo	0	48	3/22/2015	113.4	HEIGHT	19.15	16.5		1	1
13	3374	12	Placebo	0	84	7/24/2016	120.2	HEIGHT		16.5		0	1
13	3374	24	Placebo	0	91	3/29/2017	124.2	HEIGHT	22.8	17		0	1
13	3374	36	Placebo	0	106	5/17/2018	128.9	HEIGHT	24.95	17.5		0	1
13	3374	48	Placebo	0	117	4/25/2019	133.4	HEIGHT	27.55	18		0	1
13	3374	60	Placebo	0	126	2/15/2020	135.7	HEIGHT	29.25	19		0	1
13	3379	12	Placebo	0	33	7/24/2016	79.6	HEIGHT		14.2		1	1
13	3380	12	Placebo	0	54	7/24/2016	109.5	HEIGHT		13.6		1	1
13	3381	0	Placebo	1	36	3/22/2015	91.8	HEIGHT	11.35	13.5		0	1
13	3381	24	Placebo	1	53	3/29/2017	107.9	HEIGHT	15.1	14		0	1
13	3382	0	Placebo	1	48	3/22/2015	118.3	HEIGHT	21.95	17.5		0	1
13	3382	36	Placebo	1	100	5/17/2018	130.4	HEIGHT	27.31818182	19.5		0	1
13	3383	0	Placebo	1	54	3/22/2015	121.7	HEIGHT	23.15	17		0	1
13	3383	12	Placebo	1	69	7/25/2016	128	HEIGHT		19.3		0	1
13	3383	24	Placebo	1	78	3/29/2017	130.2	HEIGHT	28.65	19		0	1
13	3383	48	Placebo	1	103	4/25/2019	138.6	HEIGHT	34.25	20.5		0	1
13	3386	0	Placebo	0	36	3/22/2015	91.5	HEIGHT	12.4	14.5	36	0	1
13	3389	0	Placebo	1	30	3/22/2015	84.6	HEIGHT	10.1	13.5		1	1
13	3389	12	Placebo	1	40	7/24/2016	93.5	HEIGHT		14.1		0	1
13	3389	24	Placebo	1	48	3/29/2017	98.4	HEIGHT	13.25	14		0	1
13	3389	36	Placebo	1	63	5/17/2018	103.9	HEIGHT	14.5	14		0	1
13	3389	48	Placebo	1	74	4/25/2019	108.9	HEIGHT	15.5	13.5		0	1
13	3389	60	Placebo	1	84	2/15/2020	112.4	HEIGHT	17.3	14.3		0	1
13	3390	12	Placebo	0	30	7/24/2016	95.9	HEIGHT		14.7		1	1
13	3391	0	Placebo	0	12	3/22/2015	75.8	LENGTH	8.95	14.5		1	1
13	3391	12	Placebo	0	30	7/24/2016	89	HEIGHT		17		1	1
13	3391	24	Placebo	0	41	3/29/2017	93.9	HEIGHT	14.2	15		0	1
13	3391	36	Placebo	0	51	5/17/2018	102.2	HEIGHT	15.5	14		1	1
13	3391	48	Placebo	0	62	4/25/2019	107.7	HEIGHT	16.45	14		0	1

13	3391	60	Placebo	0	62	2/15/2020	111.4	HEIGHT	18.4	14.5	0	1
13	3393	0	Placebo	0	24	3/22/2015	93.6	HEIGHT	13.7	15	0	1
13	3393	12	Placebo	0	32	7/24/2016	102.3	HEIGHT		15.2	0	1
13	3393	24	Placebo	0	50	3/29/2017	107.8	HEIGHT	17.05	15	0	1
13	3393	36	Placebo	0	65	5/17/2018	112	HEIGHT	18.81818182	15	0	1
13	3393	48	Placebo	0	76	4/25/2019	115.7	HEIGHT	20	16	0	1
13	3393	60	Placebo	0	86	2/15/2020	119.8	HEIGHT	22.35	16	0	1
13	3396	0	Placebo	0	36	3/22/2015	83.2	HEIGHT	10.25	13	0	1
13	3396	12	Placebo	0	44	7/24/2016	91.8	HEIGHT		14.5	0	1
13	3396	24	Placebo	0	55	3/29/2017	97.5	HEIGHT	13.55	13.5	0	1
13	3399	0	Placebo	0	36	3/22/2015	83.2	HEIGHT	11.1	14.5	1	1
13	3399	12	Placebo	0	24	7/24/2016	95.7	HEIGHT		15.8	0	1
13	3399	60	Placebo	0	62	2/15/2020	116	HEIGHT	20.9	15.5	0	1
13	3400	12	Placebo	0	2	7/24/2016	70.2	HEIGHT		14.3	0	1
13	3400	60	Placebo	0	47	2/15/2020	101.3	HEIGHT	15.7	13.9	0	1
13	3401	0	Placebo	0	48	3/22/2015	103.5	HEIGHT	15.85	15	1	1
13	3401	12	Placebo	0	56	7/24/2016	110.8	HEIGHT		15.2	0	1
13	3401	24	Placebo	0	67	3/29/2017	113.7	HEIGHT	19.4	15.5	0	1
13	3402	0	Placebo	0	54	3/22/2015	113.4	HEIGHT	18.7	15	0	1
13	3402	12	Placebo	0	83	7/24/2016	121.2	HEIGHT		14.6	0	1
13	3402	24	Placebo	0	91	3/29/2017	124.5	HEIGHT	23.05	15	0	1
13	3402	36	Placebo	0	106	5/17/2018	131.4	HEIGHT	24.31818182	16	0	1
13	3402	48	Placebo	0	117	4/25/2019	136.4	HEIGHT	27.35	17	0	1
13	3404	12	Placebo	0	35	7/24/2016	89	HEIGHT		15.3	1	1
13	3404	24	Placebo	0	43	3/29/2017	93.7	HEIGHT	12.4	14	1	1
13	3405	12	Placebo	0	6	7/24/2016	69.7	HEIGHT		14.3	1	1
13	3405	36	Placebo	0	40	5/17/2018	87.6	HEIGHT	11.90909091	12.5	0	1
13	3405	48	Placebo	0	51	4/25/2019	95.3	HEIGHT	13.5	14	1	1
13	3407	0	Placebo	1	54	3/22/2015	101	HEIGHT	14	14.5	0	1
13	3407	12	Placebo	1	65	7/24/2016	109.5	HEIGHT		14.5	0	1
13	3407	36	Placebo	1	69	5/17/2018	118.7	HEIGHT	18.95454545	14.5	0	1
13	3407	48	Placebo	1	80	4/25/2019	122.6	HEIGHT	20.55	14.5	0	1
13	3407	60	Placebo	1	90	2/15/2020	124.3	HEIGHT	22.05	15	0	1
13	3408	0	Placebo	0	24	3/22/2015	87.6	LENGTH	12.2	16.5	1	1
13	3408	12	Placebo	0	37	7/24/2016	95	HEIGHT		16.5	0	1
13	3408	24	Placebo	0	48	3/29/2017	99.9	HEIGHT	16.4	15	0	1
13	3408	36	Placebo	0	62	5/17/2018	108.2	HEIGHT	18.31818182	15	0	1
13	3408	48	Placebo	0	73	4/25/2019	113.3	HEIGHT	18.5	15	0	1
13	3411	0	Placebo	0	48	3/22/2015	99.8	HEIGHT	14.5	13.5	1	1
13	3411	12	Placebo	0	59	7/24/2016	108.5	HEIGHT		14.8	0	1

13	3411	24	Placebo	0	67	3/29/2017	113.2	HEIGHT	17.7	13.5	0	1
13	3411	36	Placebo	0	82	5/17/2018	118.6	HEIGHT	19.81818182	14.5	0	1
13	3411	60	Placebo	0	102	2/15/2020	126.3	HEIGHT	23.25	14.8	0	1
13	3412	24	Placebo	1	55	3/29/2017	101.9	HEIGHT	12.9	13.5	1	1
13	3413	0	Placebo	0	24	6/11/2015	83.3	HEIGHT	10.95	14	0	1
13	3413	36	Placebo	0	58	5/17/2018	104.3	HEIGHT	15.75	14	0	1
13	3414	0	Placebo	0	24	3/22/2015	85.3	HEIGHT	11.35	14	0	1
13	3414	12	Placebo	0	45	7/24/2016	94.6	HEIGHT		15	0	1
13	3414	24	Placebo	0	53	3/29/2017	97.2	HEIGHT	14.35	13.5	1	1
13	3414	48	Placebo	0	79	4/25/2019	108.6	HEIGHT	16.7	14	0	1
13	3416	0	Placebo	0	54	3/22/2015	114.5	HEIGHT	18.05	14.5	0	1
13	3416	12	Placebo	0	65	7/24/2016	121.9	HEIGHT		15.6	0	1
13	3416	24	Placebo	0	73	3/29/2017	124.3	HEIGHT	22.5	15	0	1
13	3416	36	Placebo	0	88	5/17/2018	130	HEIGHT	24.31818182	16.5	0	1
13	3416	60	Placebo	0	108	2/15/2020	136.8	HEIGHT	28.4	16.7	0	1
13	3419	0	Placebo	0	48	3/22/2015	108.2	HEIGHT	15.25	13.5	1	1
13	3419	12	Placebo	0	59	7/24/2016	114.4	HEIGHT		14.3	1	1
13	3419	24	Placebo	0	67	3/29/2017	117.7	HEIGHT	18.95	14.5	0	1
13	3419	36	Placebo	0	82	5/17/2018	121.5	HEIGHT	19	14.5	0	1
13	3419	48	Placebo	0	93	4/25/2019	125.1	HEIGHT	20.75	15	0	1
13	3419	60	Placebo	0	102	2/15/2020	127.9	HEIGHT	22.65	15	0	1
13	3421	0	Placebo	0	36	6/11/2015	102.2	HEIGHT	14.35	13.5	0	1
13	3421	36	Placebo	0	70	5/17/2018	122.7	HEIGHT	20.5	14.5	0	1
13	3422	0	Placebo	0	12	3/22/2015	74.3	LENGTH	9.85	15	0	1
13	3422	12	Placebo	0	23	7/24/2016	86.4	HEIGHT		15.6	1	1
13	3422	24	Placebo	0	31	3/29/2017	91.3	HEIGHT	13.8	15	1	1
13	3422	48	Placebo	0	57	4/25/2019	104.2	HEIGHT	16.95	15.5	0	1
13	3422	60	Placebo	0	66	2/15/2020	108.3	HEIGHT	18.25	15	0	1
13	3423	24	Placebo	0	43	3/29/2017	93.3	HEIGHT	12.85	14.5	1	1
13	3426	0	Placebo	1	48	3/22/2015	97.5	HEIGHT	15.7	16	0	1
13	3426	12	Placebo	1	63	7/24/2016	104.7	HEIGHT		16.4	0	1
13	3426	36	Placebo	1	86	5/17/2018	113.5	HEIGHT	18.59090909	15	0	1
13	3426	48	Placebo	1	98	4/25/2019	117.9	HEIGHT	20.1	15.5	0	1
13	3428	0	Placebo	0	48	3/22/2015	105.2	HEIGHT	16.65	14.5	0	1
13	3428	12	Placebo	0	61	7/24/2016	112.6	HEIGHT		14.9	0	1
13	3428	24	Placebo	0	72	3/29/2017	115.9	HEIGHT	20.75	15.5	0	1
13	3428	48	Placebo	0	98	4/25/2019	124.1	HEIGHT	23.55	16	0	1
13	3431	0	Placebo	0	48	3/22/2015	109.6	HEIGHT	18.65	16.5	1	1
13	3431	36	Placebo	0	92	5/17/2018	123.8	HEIGHT	24.18181818	17	0	1
13	3431	48	Placebo	0	103	4/25/2019	127.5	HEIGHT	26	17	0	1

13	3431	60	Placebo	0	112	2/15/2020	133.1	HEIGHT	27.3	17.4	0	1
13	3435	0	Placebo	1	36	3/22/2015	92.1	HEIGHT	13.15	15.5	0	1
13	3435	12	Placebo	1	48	7/24/2016	103.2	HEIGHT		15.6	0	1
13	3435	24	Placebo	1	55	3/29/2017	107.8	HEIGHT	16.3	15.5	0	1
13	3435	36	Placebo	1	70	5/17/2018	114.4	HEIGHT	17.7	15	0	1
13	3435	48	Placebo	1	81	4/25/2019	119.3	HEIGHT	19.85	15.5	0	1
13	3435	60	Placebo	1	90	2/15/2020	123.1	HEIGHT	21.95	15.3	0	1
13	3438	24	Placebo	1	6	3/29/2017	62.6	LENGTH	7.1	14.5	1	1
13	3438	36	Placebo	1	21	5/17/2018	73.3	LENGTH	9.590909091	15	0	1
13	3438	48	Placebo	1	32	4/25/2019	79.9	HEIGHT	10.7	15	0	1
13	3439	0	Placebo	1	24	3/22/2015	81.2	HEIGHT	9.4	13.5	0	1
13	3439	12	Placebo	1	46	7/24/2016	91.4	HEIGHT		13.7	0	1
13	3439	24	Placebo	1	53	3/29/2017	94.7	HEIGHT	12.2	13.5	0	1
13	3439	36	Placebo	1	68	5/17/2018	99.7	HEIGHT	13.09090909	13	0	1
13	3439	48	Placebo	1	79	4/25/2019	105.5	HEIGHT	14.3	13.5	0	1
13	3440	0	Placebo	0	12	3/22/2015	66.5	LENGTH	5.55	11	1	1
13	3440	12	Placebo	0	23	7/24/2016	75.8	HEIGHT		13	0	1
13	3440	36	Placebo	0	46	5/17/2018	92.4	HEIGHT	12.09090909	13.5	1	1
13	3441	12	Placebo	0	12	7/24/2016	74.8	HEIGHT		13.9	1	1
13	3441	24	Placebo	0	20	3/29/2017	81.9	HEIGHT	10.8	14.5	0	1
13	3441	36	Placebo	0	35	5/17/2018	94.5	HEIGHT	12.40909091	14	0	1
13	3441	48	Placebo	0	46	4/25/2019	100.2	HEIGHT	14.45	14.5	0	1
13	3441	60	Placebo	0	55	2/15/2020	105.7	HEIGHT	15.95	14.5	0	1
13	3442	24	Placebo	1	41	3/29/2017	97	HEIGHT	12.45	12.5	1	1
13	3445	24	Placebo	1	3	3/31/2017	59	LENGTH	5.55	13	1	1
13	3450	24	Placebo	0	59	3/29/2017	97	HEIGHT	15.05	16.5	1	1
13	3451	0	Placebo	0	36	3/22/2015	89.2	HEIGHT	13.15	15.5	0	1
13	3451	24	Placebo	0	55	3/29/2017	104.2	HEIGHT	16.8	15.5	0	1
13	3451	36	Placebo	0	70	5/17/2018	109.2	HEIGHT	18.5	16	0	1
13	3453	0	Placebo	0	36	3/22/2015	87.2	HEIGHT	11.4	14.5	0	1
13	3453	12	Placebo	0	47	7/24/2016	97.5	HEIGHT		15.3	0	1
13	3453	24	Placebo	0	55	3/29/2017	100.4	HEIGHT	15.65	15	1	1
13	3453	36	Placebo	0	70	5/17/2018	107	HEIGHT	17.22727273	14.5	0	1
13	3455	0	Placebo	0	8	3/22/2015	70.8	LENGTH	7.7	14.5	0	1
13	3455	24	Placebo	0	31	3/29/2017	84.2	HEIGHT	10.75	14	0	1
13	3455	36	Placebo	0	46	5/17/2018	91.9	HEIGHT	12.90909091	14	0	1
13	3455	48	Placebo	0	57	4/25/2019	98.4	HEIGHT	14.4	15	0	1
13	3455	60	Placebo	0	67	2/15/2020	104.9	HEIGHT	16.45	15.1	0	1
13	3456	0	Placebo	0	34	3/22/2015	102.7	HEIGHT	14.6	13.5	0	1
13	3456	48	Placebo	0	79	4/25/2019	122.1	HEIGHT	21	14.5	0	1

13	3456	60	Placebo	0	88	2/15/2020	123.4	LENGTH	21.8	14.7	0	1
13	3457	0	Placebo	0	12	3/22/2015	72.1	LENGTH	8.7	14	1	1
13	3457	12	Placebo	0	30	7/25/2016	86.2	HEIGHT		15.6	0	1
13	3457	24	Placebo	0	41	3/29/2017	92.3	HEIGHT	13.3	15	0	1
13	3458	24	Placebo	1	20	3/29/2017	79.7	HEIGHT	10.05	14.5	1	1
13	3463	0	Placebo	0	48	3/22/2015	94.5	HEIGHT	13.7	15	1	1
13	3463	12	Placebo	0	59	7/24/2016	102.2	HEIGHT		14.8	0	1
13	3463	24	Placebo	0	67	3/29/2017	106.3	HEIGHT	17.85	15.5	0	1
13	3465	12	Placebo	1	20	7/24/2016	88.8	HEIGHT		15.8	1	1
13	3465	60	Placebo	1	56	2/15/2020	114.7	HEIGHT	19.35	15.3	1	1
13	3467	12	Placebo	0	53	7/25/2016	97.5	HEIGHT		14.1	1	1
13	3468	12	Placebo	1	9	7/24/2016	67.4	HEIGHT		12.5	0	1
13	3470	0	Placebo	1	36	3/22/2015	101.2	HEIGHT	14.3	15	1	1
13	3474	12	Placebo	1	9	7/24/2016	71.3	HEIGHT		15.2	0	1
13	3474	24	Placebo	1	14	3/29/2017	77.2	HEIGHT	10.1	14.5	0	1
13	3474	36	Placebo	1	36	5/17/2018	84.9	HEIGHT	10.68181818	13	0	1
13	3474	60	Placebo	1	57	2/15/2020	94.3	HEIGHT	13.45	13.3	0	1
13	3476	12	Placebo	1	24	7/25/2016	83	HEIGHT		15.3	1	1
13	3479	0	Placebo	0	36	3/22/2015	83.3	HEIGHT	11.95	14.5	1	1
13	3479	12	Placebo	0	48	7/24/2016	93.7	HEIGHT		16.4	0	1
13	3479	24	Placebo	0	55	3/29/2017	97.2	HEIGHT	15.1	15	0	1
13	3479	36	Placebo	0	70	5/17/2018	104	HEIGHT	16.36363636	15.5	0	1
13	3479	60	Placebo	0	90	2/15/2020	111.6	HEIGHT	19.15	15.1	0	1
13	3481	12	Placebo	0	8	7/24/2016	67.9	HEIGHT		15.2	0	1
13	3483	12	Placebo	0	3	7/25/2016	63.6	LENGTH		14.1	0	1
13	3483	24	Placebo	0	12	3/29/2017	73	HEIGHT	8.75	14.5	0	1
13	3483	36	Placebo	0	26	5/17/2018	85.3	HEIGHT	11.27272727	14.5	1	1
13	3483	60	Placebo	0	47	2/15/2020	96.9	HEIGHT	14.25	15	0	1
13	3484	0	Placebo	0	54	3/22/2015	95.9	HEIGHT	14.15	15	1	1
13	3484	12	Placebo	0	65	7/24/2016	103.8	HEIGHT		14.4	0	1
13	3484	24	Placebo	0	73	3/29/2017	106	HEIGHT	16.55	14.5	0	1
13	3484	36	Placebo	0	88	5/17/2018	111.9	HEIGHT	18.90909091	15	0	1
13	3484	48	Placebo	0	99	4/25/2019	114.3	HEIGHT	20.25	15.5	0	1
13	3488	12	Placebo	0	10	7/24/2016	74.9	HEIGHT		13.6	0	1
13	3488	24	Placebo	0	20	3/29/2017	79.8	HEIGHT	9.1	13.5	0	1
13	3488	36	Placebo	0	35	5/17/2018	90.3	HEIGHT	11.15	13.5	1	1
13	3488	60	Placebo	0	56	2/15/2020	103.2	HEIGHT	14.85	14.2	0	1
13	3490	0	Placebo	0	12	3/22/2015	79.6	HEIGHT	10.4	15.5	0	1
13	3490	12	Placebo	0	23	7/24/2016	89.4	HEIGHT		15.4	1	1
13	3490	24	Placebo	0	31	3/29/2017	93.2	HEIGHT	15.15	14.5	1	1

13	3490	36	Placebo	0	46	5/17/2018	104.6	HEIGHT	17.63636364	16	0	1
13	3491	12	Placebo	1	42	7/24/2016	118.3	HEIGHT		15.4	1	1
13	3494	12	Placebo	1	42	7/24/2016	98.3	HEIGHT		15.3	1	1
13	3497	0	Placebo	1	54	3/22/2015	104.1	HEIGHT	14.45	15	1	1
13	3504	24	Placebo	1	19	3/29/2017	81	HEIGHT	10.8	14	1	1
13	3504	36	Placebo	1	37	5/17/2018	88	HEIGHT	12.9	15	1	1
13	3505	12	Placebo	0	22	7/24/2016	86	HEIGHT		14.5	1	1
13	3505	24	Placebo	0	29	3/31/2017	89.7	HEIGHT	13.95	16.5	1	1
13	3508	12	Placebo	0	4	7/24/2016	65.4	HEIGHT		13.2	0	1
13	3508	24	Placebo	0	14	3/29/2017	72.7	HEIGHT	8.65	14	0	1
13	3508	36	Placebo	0	27	5/17/2018	79.7	HEIGHT	10	14	1	1
13	3508	60	Placebo	0	48	2/15/2020	91	HEIGHT	13.45	14.5	0	1
13	3511	0	Placebo	1	48	3/22/2015	96.7	HEIGHT	13.55	15	0	1
13	3511	24	Placebo	1	41	3/29/2017	110.3	HEIGHT	17.2	14.5	0	1
13	3511	36	Placebo	1	56	5/17/2018	118.3	HEIGHT	19	14.5	0	1
13	3511	48	Placebo	1	67	4/25/2019	122.6	HEIGHT	20.75	15	0	1
13	3511	60	Placebo	1	77	2/15/2020	126.5	HEIGHT	22.5	15.7	0	1
13	3512	12	Placebo	0	3	7/24/2016	63.1	HEIGHT		13.5	1	1
13	3512	24	Placebo	0	10	3/29/2017	70.7	HEIGHT	6.85	12	1	1
13	3512	36	Placebo	0	21	5/17/2018	81.6	HEIGHT	9.454545455	12.5	1	1
13	3512	48	Placebo	0	32	4/25/2019	88.4	HEIGHT	11.35	13	0	1
13	3513	12	Placebo	0	42	7/24/2016	107.6	HEIGHT		15.6	1	1
13	3515	0	Placebo	1	7	3/22/2015	69.6	LENGTH	7.5	14	0	1
13	3515	12	Placebo	1	18	7/24/2016	83.5	HEIGHT		12.2	0	1
13	3515	24	Placebo	1	29	3/29/2017	87.4	HEIGHT	11.1	12.5	1	1
13	3515	36	Placebo	1	44	5/17/2018	94	HEIGHT	12.15	13	0	1
13	3515	48	Placebo	1	55	4/25/2019	100.5	HEIGHT	14.25	13.5	0	1
13	3515	60	Placebo	1	56	2/15/2020	104.3	HEIGHT	15.2	13.7	0	1
13	3516	24	Placebo	1	25	3/29/2017	79.6	HEIGHT	10.35	13	1	1
13	3518	0	Placebo	0	24	3/22/2015	86.2	HEIGHT	11.95	14.5	1	1
13	3518	12	Placebo	0	45	7/24/2016	95.5	HEIGHT		15.1	0	1
13	3519	0	Placebo	1	12	3/22/2015	71.4	LENGTH	7.95	14	0	1
13	3519	12	Placebo	1	18	7/24/2016	83.2	HEIGHT		14.8	0	1
13	3519	24	Placebo	1	26	3/29/2017	86.5	HEIGHT	12.6	14.5	0	1
13	3519	36	Placebo	1	41	5/17/2018	95	HEIGHT	14.35	15	1	1
13	3519	48	Placebo	1	52	4/25/2019	100	HEIGHT	15.35	15	0	1
13	3519	60	Placebo	1	61	2/15/2020	105.1	HEIGHT	16.65	14.3	0	1
13	3520	0	Placebo	0	2	3/22/2015	56.7	LENGTH	5.05	12.5	0	1
13	3520	12	Placebo	0	14	7/24/2016	73	HEIGHT		13.1	0	1
13	3520	24	Placebo	0	25	3/29/2017	78.5	HEIGHT	9.5	14.5	0	1

13	3520	48	Placebo	0	51	4/25/2019	94	HEIGHT	13.1	14.5		0	1
13	3523	12	Placebo	0	2	7/24/2016	77.6	HEIGHT		14		0	1
13	3523	24	Placebo	0	20	3/29/2017	83.9	HEIGHT	10.3	13		1	1
13	3523	36	Placebo	0	35	5/17/2018	92.4	HEIGHT	11.55	13		0	1
13	3523	48	Placebo	0	46	4/25/2019	98.6	HEIGHT	14.25	14		1	1
13	3523	60	Placebo	0	35	2/15/2020	105	HEIGHT	15.85	13.3		1	1
13	3524	0	Placebo	1	24	3/22/2015	80.4	LENGTH	9.3	14		1	1
13	3524	12	Placebo	1	32	7/24/2016	91	HEIGHT		14.8		0	1
13	3524	24	Placebo	1	43	3/29/2017	96	HEIGHT	12.55	13.5		1	1
13	3524	36	Placebo	1	58	5/17/2018	102	HEIGHT	13.75	13		1	1
13	3524	48	Placebo	1	69	4/25/2019	107.1	HEIGHT	14.85	13		0	1
13	3524	60	Placebo	1	78	2/15/2020	112.2	HEIGHT	17.7	14.2		0	1
13	3527	0	Placebo	0	36	3/22/2015	101.7	HEIGHT	16.4	16		1	1
13	3527	36	Placebo	0	70	5/17/2018	119.5	HEIGHT	22.18181818	16.5		0	1
13	3533	0	Placebo	1	36	3/22/2015	88	HEIGHT	10.55	13.5		1	1
13	3533	12	Placebo	1	51	7/24/2016	98.2	HEIGHT		13.5		0	1
13	3533	48	Placebo	1	86	4/25/2019	114	HEIGHT	15.9	14		0	1
13	3535	24	Placebo	1	31	3/29/2017	86	HEIGHT	11.9	14.5		1	1
13	3537	24	Placebo	1	29	3/29/2017	86.7	HEIGHT	11.1	14.5		1	1
13	3540	24	Placebo	1	43	3/31/2017	91.5	HEIGHT	11.7	13.5		1	1
13	3541	0	Placebo	1	1	3/22/2015	55.4	LENGTH	4.75	12	42	0	1
13	3541	24	Placebo	1	20	3/29/2017	82	HEIGHT	10.95	14	42	0	1
13	3541	36	Placebo	1	35	5/17/2018	92	HEIGHT	12.68181818	14.5	42	0	1
13	3542	0	Placebo	0	6	3/22/2015	61	LENGTH	5.95	12		0	1
13	3542	12	Placebo	0	17	7/24/2016	74.5	HEIGHT		12.8		1	1
13	3542	24	Placebo	0	30	3/29/2017	76.7	HEIGHT	9.65	13.5		0	1
13	3542	36	Placebo	0	45	5/17/2018	88.3	HEIGHT	11.27272727	13		1	1
13	3542	48	Placebo	0	56	4/25/2019	95.6	HEIGHT	12.3	13		0	1
13	3542	60	Placebo	0	65	2/15/2020	99.9	HEIGHT	14	12.8		0	1
13	3543	0	Placebo	0	30	3/22/2015	83.9	HEIGHT	11.25	14.5		1	1
13	3543	12	Placebo	0	46	7/24/2016	95.3	HEIGHT		15.4		1	1
13	3543	24	Placebo	0	53	3/29/2017	100.4	HEIGHT	15.95	15.5		0	1
13	3543	36	Placebo	0	68	5/17/2018	107.6	HEIGHT	18.3	16		0	1
13	3543	48	Placebo	0	79	4/25/2019	113.1	HEIGHT	20.5	16		0	1
13	3543	60	Placebo	0	89	2/15/2020	116.8	HEIGHT	21.2	16		0	1
13	3548	0	Placebo	1	4	3/22/2015	61.3	LENGTH	6.15	12.5		1	1
13	3548	12	Placebo	1	18	7/24/2016	79.2	HEIGHT		13.8		0	1
13	3548	24	Placebo	1	25	3/29/2017	82.5	HEIGHT	11.3	14.5		0	1
13	3548	36	Placebo	1	40	5/17/2018	92.5	HEIGHT	12.63636364	14.5		0	1
13	3548	60	Placebo	1	60	2/15/2020	104.3	HEIGHT	16.2	14.3		0	1

13	3549	24	Placebo	1	20	3/29/2017	83.5	HEIGHT	10.3	13		1	1
13	3550	12	Placebo	0	35	7/25/2016	96.1	HEIGHT		14.3		1	1
13	3551	12	Placebo	1	23	7/24/2016	80.4	HEIGHT		14.5		1	1
13	3557	0	Placebo	0	36	3/22/2015	83.2	HEIGHT	10.45	14.5		1	1
13	3557	12	Placebo	0	42	7/24/2016	92.5	HEIGHT		15.7		1	1
13	3557	48	Placebo	0	67	4/25/2019	109.1	HEIGHT	17.05	15		0	1
13	3557	60	Placebo	0	76	2/15/2020	113.6	HEIGHT	18.3	15.2		0	1
13	3559	0	Placebo	0	4	6/11/2015	65.1	LENGTH	5.3	11	18	0	1
13	3561	0	Placebo	0	18	3/22/2015	77	LENGTH	9.5	15		1	1
13	3562	12	Placebo	1	12	7/24/2016	80	HEIGHT		13.8		1	1
13	3563	0	Placebo	1	24	3/22/2015	81.4	HEIGHT	8.8	13.5		0	1
13	3563	12	Placebo	1	35	7/24/2016	91.2	HEIGHT		13.4		0	1
13	3563	48	Placebo	1	72	4/25/2019	109.2	HEIGHT	15.75	13.5		0	1
13	3563	60	Placebo	1	82	2/15/2020	112.7	HEIGHT	16.4	12.9		0	1
13	3564	0	Placebo	0	54	3/22/2015	104.8	HEIGHT	17.4	15.5		0	1
13	3564	12	Placebo	0	46	7/24/2016	114.2	HEIGHT		16.3		1	1
13	3565	0	Placebo	0	36	3/22/2015	101.5	HEIGHT	16.45	15		1	1
13	3565	12	Placebo	0	44	7/24/2016	111.6	HEIGHT		16.1		1	1
13	3565	24	Placebo	0	55	3/29/2017	116	HEIGHT	20.95	16		1	1
13	3565	36	Placebo	0	70	5/17/2018	122	HEIGHT	22.25	15.5		0	1
13	3565	48	Placebo	0	81	4/25/2019	127.2	HEIGHT	25	16		0	1
13	3565	60	Placebo	0	90	2/15/2020	131.2	HEIGHT	26.45	16.7		0	1
13	3570	0	Placebo	1	54	3/22/2015	94.9	HEIGHT	14.5	15		1	1
13	3571	0	Placebo	0	36	3/22/2015	99.4	HEIGHT	15.9	14.5		0	1
13	3571	12	Placebo	0	30	7/24/2016	109.9	HEIGHT		15.2		0	1
13	3571	24	Placebo	0	41	3/29/2017	114	HEIGHT	20.15	15.5		0	1
13	3571	36	Placebo	0	72	5/17/2018	119.7	HEIGHT	20.95	15.5		0	1
13	3571	48	Placebo	0	83	4/25/2019	123.2	HEIGHT	22.9	15.5		0	1
13	3571	60	Placebo	0	92	2/15/2020	127.8	HEIGHT	24.5	16.3		0	1
13	3573	0	Placebo	1	12	3/22/2015	73.8	LENGTH	8.45	14		1	1
13	3573	12	Placebo	1	20	7/24/2016	88.1	HEIGHT		14.8		0	1
13	3573	24	Placebo	1	31	3/29/2017	92.2	HEIGHT	13.35	14.5		0	1
13	3574	24	Placebo	0	29	3/29/2017	85.4	HEIGHT	10.35	13.5		1	1
13	3575	24	Placebo	0	4	3/31/2017	68.3	LENGTH	7.45	13		1	1
13	3575	36	Placebo	0	15	5/17/2018	81.9	HEIGHT	9.7	13		0	1
13	3575	48	Placebo	0	26	4/25/2019	89.3	HEIGHT	11.55	13.5		1	1
13	3577	24	Placebo	1	32	3/31/2017	83	HEIGHT	11.4	14.5		1	1
13	3578	24	Placebo	0	55	3/29/2017	95.7	HEIGHT	15.85	16		1	1
13	3579	0	Placebo	0	36	3/22/2015	91.9	HEIGHT	12.55	14.5		0	1
13	3579	12	Placebo	0	47	7/24/2016	101.8	HEIGHT		14.4		0	1

13	3579	24	Placebo	0	55	3/29/2017	106.5	HEIGHT	14.3	14.5	0	1
13	3579	48	Placebo	0	81	4/25/2019	117.5	HEIGHT	18.65	15	0	1
13	3579	60	Placebo	0	90	2/15/2020	122.5	HEIGHT	20.45	14.7	0	1
13	3580	12	Placebo	1	6	7/24/2016	66.9	HEIGHT		13.2	0	1
13	3580	24	Placebo	1	12	3/29/2017	73.3	LENGTH	7.5	13	0	1
13	3580	36	Placebo	1	26	5/17/2018	83	HEIGHT	9.4	12.5	0	1
13	3581	0	Placebo	1	36	3/22/2015	88.5	HEIGHT	12.2	14.5	0	1
13	3581	24	Placebo	1	53	3/29/2017	103.6	HEIGHT	16.45	15.5	0	1
13	3582	0	Placebo	1	54	3/22/2015	101.7	HEIGHT	15.15	14.5	0	1
13	3582	12	Placebo	1	66	7/24/2016	111.2	HEIGHT		14.4	0	1
13	3582	36	Placebo	1	88	5/17/2018	120.8	HEIGHT	21.86363636	16	0	1
13	3583	0	Placebo	0	54	3/22/2015	111.6	HEIGHT	17.95	14.5	1	1
13	3583	24	Placebo	0	72	3/29/2017	124	HEIGHT	21.95	15	0	1
13	3584	12	Placebo	1	20	7/25/2016	77.4	HEIGHT		13.5	1	1
13	3585	24	Placebo	1	41	3/29/2017	87.5	HEIGHT	12.3	14.5	1	1
13	3586	0	Placebo	0	18	3/22/2015	77.6	HEIGHT	9.4	13	0	1
13	3586	12	Placebo	0	26	7/24/2016	86.6	HEIGHT		13.2	0	1
13	3586	36	Placebo	1	52	5/17/2018	103	HEIGHT	14.35	14	0	1
13	3586	48	Placebo	1	63	4/25/2019	109.6	HEIGHT	16.2	14	0	1
13	8034	36	Placebo	0	58	5/17/2018	97.7	HEIGHT	15.55	15.5	1	1
13	8043	48	Placebo	0	4	4/25/2019	60.6	LENGTH	5.65	12	1	1
13	8043	60	Placebo	0	12	2/15/2020	69.2	LENGTH	7.75	12.4	0	1
13	8048	60	Placebo	0	14	2/15/2020	75.5	LENGTH	9.5	14	1	1
13	8088	60	Placebo	0	56	2/15/2020	104.9	HEIGHT	14.65	13.7	1	1
13	8090	48	Placebo	0	27	4/25/2019	86.3	HEIGHT	13.1	16.5	1	1
13	8133	48	Placebo	1	52	4/25/2019	101.1	HEIGHT	14.5	14	1	1
13	8134	48	Placebo	0	27	4/25/2019	85.4	LENGTH	11.1	15	1	1
13	8135	36	Placebo	0	2	5/17/2018	59.6	LENGTH	5.227272727	12	1	1
13	8135	48	Placebo	0	12	4/25/2019	76.6	LENGTH	8.7	13	0	1
13	8135	60	Placebo	0	21	2/15/2020	83.2	HEIGHT	10.95	13.7	0	1
13	8139	48	Placebo	1	16	4/25/2019	71.4	LENGTH	7.5	12.5	1	1
13	8139	60	Placebo	1	25	2/20/2020	80.2	HEIGHT	10.5	13.3	1	1
13	8158	36	Placebo	1	8	5/17/2018	66.9	LENGTH	7.409090909	14.5	1	1
13	8158	60	Placebo	1	28	2/15/2020	82.3	HEIGHT	10.95	15	0	1
13	8175	48	Placebo	1	55	4/25/2019	102.3	HEIGHT	15.9	14.5	1	1
13	8180	48	Placebo	0	32	4/25/2019	87.2	HEIGHT	12.85	15	1	1
13	8208	48	Placebo	0	27	4/25/2019	84.1	LENGTH	9.75	13.5	1	1
13	8246	36	Placebo	0	17	5/17/2018	78.7	LENGTH	10.22727273	15	1	1
13	8313	48	Placebo	0	15	4/25/2019	76.4	LENGTH	8.65	13.5	1	1
13	8366	48	Placebo	1	1	4/25/2019	54.8	LENGTH	3.8	10.5	1	1

13	8379	36	Placebo	1	24	5/17/2018	83.6	HEIGHT	10.13636364	14.5		1	1
13	8427	48	Placebo	0	45	4/25/2019	95.8	HEIGHT	13.4	13.5		1	1
13	8432	48	Placebo	1	26	4/25/2019	85.7	HEIGHT	12.6	15.5		1	1
13	8432	60	Placebo	1	35	2/15/2020	92.4	HEIGHT	14.35	15.5		1	1
13	8488	36	Placebo	0	44	5/17/2018	98.8	HEIGHT	16	15.5		1	1
13	8516	48	Placebo	1	12	4/25/2019	70.5	LENGTH	7.85	14		1	1
13	8522	60	Placebo	1	14	2/20/2020	72.2	HEIGHT	9.05	13.9		1	1
13	8553	36	Placebo	0	40	5/17/2018	93.5	HEIGHT	12.04545455	13.5		1	1
13	8553	48	Placebo	0	51	4/25/2019	100.3	HEIGHT	15	14.5		1	1
13	8554	36	Placebo	0	44	5/17/2018	85.5	HEIGHT	10.59090909	13.5		1	1
13	8566	48	Placebo	0	32	4/25/2019	86.3	HEIGHT	11.15	13		1	1
13	8603	48	Placebo	1	12	4/25/2019	75.1	LENGTH	8.3	13.5	60	1	1
13	8611	60	Placebo	1	8	2/15/2020	67.1	LENGTH	4.909090909	16.3		1	1
13	8639	48	Placebo	0	38	4/25/2019	85.7	HEIGHT	11.75	13.5		1	1
13	8654	60	Placebo	1	21	2/15/2020	84.7	LENGTH	10.85	13.6		1	1
13	8673	36	Placebo	0	44	5/17/2018	96.4	HEIGHT	14.68181818	14.5		1	1
13	8675	48	Placebo	0	39	4/25/2019	103.1	HEIGHT	16.1	14.5		1	1
13	8701	36	Placebo	1	58	5/17/2018	104.6	HEIGHT	16.27272727	14.5		1	1
13	8701	48	Placebo	0	69	4/25/2019	109.2	HEIGHT	17.6	15		0	1
13	8701	60	Placebo	0	78	2/15/2020	112.8	HEIGHT	19.65	15		0	1
13	8718	36	Placebo	0	27	5/17/2018	79.1	LENGTH	9.2	13		1	1
13	8718	60	Placebo	1	48	2/20/2020	96.6	HEIGHT	13.9	14		1	1
13	8780	36	Placebo	1	12	5/17/2018	74.9	LENGTH	8.136363636	12.5		1	1
13	8780	48	Placebo	1	22	4/25/2019	84.6	LENGTH	9.9	13.5		1	1
13	8788	60	Placebo	1	23	2/20/2020	75.8	LENGTH	10	14		1	1
13	8792	36	Placebo	1	14	5/17/2018	64.5	LENGTH	5.454545455	10		1	1
13	8902	36	Placebo	1	12	5/17/2018	70.3	LENGTH	6.727272727	12		1	1
13	8902	60	Placebo	1	33	2/15/2020	88.2	HEIGHT	10.6	12.7		0	1
13	8913	36	Placebo	0	5	5/17/2018	66.6	LENGTH	7.6	14		1	1
13	8913	48	Placebo	0	14	4/25/2019	80.5	LENGTH	10.1	14.5		0	1
13	8913	60	Placebo	0	24	2/15/2020	86.3	HEIGHT	11.4	14		0	1
13	8955	60	Placebo	0	50	2/20/2020	95.4	HEIGHT	14.05	14.5	60	1	1
13	8972	36	Placebo	0	33	5/17/2018	82.6	HEIGHT	10.05	12		1	1
13	8972	60	Placebo	0	53	2/15/2020	95.5	HEIGHT	12.95	13		1	1
13	8991	36	Placebo	1	2	5/17/2018	54.6	LENGTH	5.045454545	12		1	1
13	8994	48	Placebo	1	42	4/25/2019	94.1	HEIGHT	12.5	13.5		1	1
13	9001	60	Placebo	0	20	2/15/2020	76.5	LENGTH	10	14.3		1	1
13	9004	36	Placebo	1	25	5/17/2018	80.9	HEIGHT	9.772727273	12		1	1
13	9013	48	Placebo	1	43	4/25/2019	91	HEIGHT	13.15	16		1	1
13	9013	60	Placebo	1	53	2/20/2020	96.8	HEIGHT	15.2	15		1	1

13	9022	60	Placebo	1	43	2/20/2020	97.8	HEIGHT	16.25	15		1	1
13	9044	60	Placebo	0	41	2/15/2020	96.2	HEIGHT	14.9	15.9		1	1
13	9046	48	Placebo	0	2	4/25/2019	53.2	LENGTH	4.5	12.5		1	1
13	9046	60	Placebo	0	11	2/15/2020	71.5	LENGTH	7.2	12.5		1	1
13	9049	36	Placebo	0	7	5/17/2018	63.5	LENGTH	5.7	12		1	1
13	9049	48	Placebo	0	15	4/25/2019	75	HEIGHT	7.8	12.5		1	1
13	9049	60	Placebo	0	25	2/15/2020	83.2	HEIGHT	9.8	13		0	1
13	9050	60	Placebo	1	37	2/15/2020	89.7	HEIGHT	13.6	15.7		1	1
13	9079	36	Placebo	1	39	5/17/2018	96.7	HEIGHT	14.9	15.5		1	1
13	9085	48	Placebo	1	15	4/25/2019	74.9	LENGTH	8.9	13.5		1	1
13	9093	48	Placebo	1	57	4/25/2019	95.3	HEIGHT	13.35	14		1	1
13	9132	48	Placebo	1	37	4/25/2019	88.8	HEIGHT	12.25	14		1	1
13	9164	36	Placebo	0	58	5/17/2018	102.7	HEIGHT	13.65	12.5		1	1
13	9173	60	Placebo	0	14	2/20/2020	81.7	HEIGHT	11.1	14.4		1	1
13	9179	36	Placebo	1	6	5/17/2018	65.2	LENGTH	7.181818182	13		1	1
13	9179	48	Placebo	1	15	4/25/2019	75.8	LENGTH	10.2	14		0	1
13	9219	60	Placebo	0	21	2/15/2020	71.2	LENGTH	8.4	11.9		1	1
13	9239	60	Placebo	0	41	2/15/2020	92.1	HEIGHT	12.25	14		1	1
13	9254	48	Placebo	1	17	4/25/2019	73.6	LENGTH	8.4	13.5	60	1	1
13	9262	60	Placebo	1	50	2/15/2020	99.9	HEIGHT	15.05	15		1	1
13	9268	60	Placebo	1	3	2/20/2020	61.9	LENGTH	5.8	11		1	1
13	9273	48	Placebo	0	51	4/25/2019	99.8	HEIGHT	13.9	13.5		1	1
13	9273	60	Placebo	0	33	2/15/2020	104.3	HEIGHT	15.05	14		1	1
13	9274	60	Placebo	0	56	2/15/2020	83.6	HEIGHT	10.1	13		1	1
13	9279	48	Placebo	0	15	4/25/2019	76.3	LENGTH	9.7	15		1	1
13	9283	48	Placebo	1	45	4/25/2019	74.7	HEIGHT	5.886363636	15		1	1
13	9288	36	Placebo	0	15	5/17/2018	73.5	HEIGHT	9.7	14		1	1
13	9288	60	Placebo	0	35	2/20/2020	86.4	HEIGHT	14.45	15.2		1	1
13	9292	48	Placebo	0	55	4/25/2019	111.7	HEIGHT	20.1	17		1	1
13	9298	48	Placebo	0	23	4/25/2019	80.5	HEIGHT	11.05	15.5		1	1
13	9298	60	Placebo	0	32	2/15/2020	87.2	HEIGHT	13	16.1		0	1
13	9309	36	Placebo	1	56	5/17/2018	93.3	HEIGHT	12.9	14		1	1
13	9333	60	Placebo	1	36	2/20/2020	88.3	HEIGHT	12.55	14		1	1
13	9338	60	Placebo	1	32	2/20/2020	87.6	HEIGHT	12.1	13.5		1	1
13	9349	36	Placebo	0	21	5/17/2018	83.1	LENGTH	11.18181818	14.5		1	1
13	9376	48	Placebo	1	12	4/25/2019	72.8	LENGTH	7.85	13.5		1	1
13	9418	60	Placebo	1	44	2/15/2020	99.6	HEIGHT	13.65	13.6		1	1
13	9421	60	Placebo	0	6	2/20/2020	68.5	LENGTH	7.6	13.7		1	1
13	9428	60	Placebo	1	21	2/15/2020	84.5	LENGTH	11.95	14.5		1	1
13	9439	48	Placebo	0	55	4/25/2019	100.8	HEIGHT	13.75	14		1	1

13	9449	48	Placebo	0	32	4/25/2019	95	HEIGHT	14.4	15		1	1
13	9452	60	Placebo	1	20	2/15/2020	76.2	LENGTH	8.6	12.9		1	1
13	9458	48	Placebo	0	10	4/25/2019	68.5	LENGTH	7.6	13.5		1	1
13	9458	60	Placebo	0	14	2/15/2020	74.2	HEIGHT	9.35	13.5		1	1
13	9521	60	Placebo	1	25	2/20/2020	85.7	HEIGHT	12.1	14		1	1
13	9550	60	Placebo	1	19	2/20/2020	79.2	HEIGHT	10.25	14.3		1	1
13	9564	60	Placebo	0	47	2/15/2020	101.3	HEIGHT	14.05	14		1	1
13	9579	60	Placebo	1	12	2/15/2020	72.4	LENGTH	7.4	12.2		1	1
14	3602	12	Placebo	1	32	7/23/2016	105.6	HEIGHT		14.5		1	1
14	3604	24	Placebo	1	21	5/6/2017	71.6	LENGTH	7.363636364	12.5		1	1
14	3605	24	Placebo	0	39	5/4/2017	83.1	HEIGHT	9.409090909	12		1	1
14	3607	0	Placebo	0	30	6/14/2015	90.1	HEIGHT	12.75	15	12	0	1
14	3610	24	Placebo	0	39	5/4/2017	82.6	HEIGHT	11.31818182	14.5		1	1
14	3611	12	Placebo	0	12	7/23/2016	78.5	HEIGHT		13.3		1	1
14	3611	48	Placebo	0	50	5/22/2019	97.4	HEIGHT	14.1	14		1	1
14	3611	60	Placebo	0	59	2/13/2020	102.8	HEIGHT	16.05	14		1	1
14	3615	0	Placebo	1	48	4/7/2015	90.2	HEIGHT	13.05	15.5		1	1
14	3615	12	Placebo	1	63	7/23/2016	100.3	HEIGHT		15		0	1
14	3615	24	Placebo	1	76	5/4/2017	105.7	HEIGHT	15.63636364	13.5		0	1
14	3615	36	Placebo	1	89	6/3/2018	112.2	HEIGHT	17.65	14.5		0	1
14	3615	48	Placebo	1	100	5/22/2019	117.6	HEIGHT	19.55	15		0	1
14	3615	60	Placebo	1	109	2/13/2020	121.8	HEIGHT	20.65	15		0	1
14	3616	12	Placebo	0	9	7/23/2016	72.3	LENGTH		13.5		0	1
14	3616	24	Placebo	0	22	5/4/2017	79.5	HEIGHT	9.545454545	14		0	1
14	3616	36	Placebo	0	35	6/3/2018	91	HEIGHT	12.35	15		1	1
14	3618	0	Placebo	1	24	4/7/2015	91.5	HEIGHT	12.85	14.5		1	1
14	3618	12	Placebo	1	39	7/23/2016	101.3	HEIGHT		14.5		1	1
14	3619	0	Placebo	0	12	4/7/2015	80.7	HEIGHT	10.85	15		0	1
14	3620	12	Placebo	0	12	7/23/2016	71	HEIGHT		14		1	1
14	3620	24	Placebo	0	25	5/4/2017	77	HEIGHT	9.818181818	13.5		0	1
14	3620	36	Placebo	0	38	6/3/2018	83.3	HEIGHT	10.85	13.5		0	1
14	3620	48	Placebo	0	48	5/22/2019	86.8	HEIGHT	11.7	13.5		0	1
14	3620	60	Placebo	0	58	2/13/2020	93.4	HEIGHT	13.09090909	13.5		0	1
14	3621	0	Placebo	1	12	4/7/2015	76.6	LENGTH	9.5	16		0	1
14	3621	12	Placebo	1	26	7/23/2016	90.1	HEIGHT		15.5		0	1
14	3621	24	Placebo	1	38	5/4/2017	100.1	HEIGHT	15.40909091	16		0	1
14	3621	36	Placebo	1	51	6/3/2018	109	HEIGHT	17.3	15.5		0	1
14	3621	48	Placebo	1	61	5/22/2019	117.2	HEIGHT	19.55	16		0	1
14	3622	12	Placebo	0	32	7/23/2016	92.3	HEIGHT		15.7		1	1
14	3622	24	Placebo	0	48	5/4/2017	98	HEIGHT	15.18181818	15		1	1

14	3624	12	Placebo	0	54	7/23/2016	113.5	HEIGHT		15		1	1
14	3627	12	Placebo	1	36	7/23/2016	91	HEIGHT		15.8		1	1
14	3627	24	Placebo	1	49	5/4/2017	96.6	HEIGHT	13.77272727	15.5		1	1
14	3628	0	Placebo	0	18	4/7/2015	81.5	HEIGHT	10.9	15	12	1	1
14	3629	12	Placebo	0	56	7/23/2016	117.2	HEIGHT		16.2		1	1
14	3632	0	Placebo	1	30	4/7/2015	74.4	HEIGHT	9.45	14	18	0	1
14	3634	12	Placebo	1	2	7/23/2016	68	LENGTH		10.9		0	1
14	3636	12	Placebo	0	30	7/23/2016	88.2	HEIGHT		13.1		1	1
14	3637	0	Placebo	0	48	4/7/2015	109.7	HEIGHT	15.7	15.5		1	1
14	3637	48	Placebo	0	99	5/22/2019	121.5	HEIGHT	21.3	15		0	1
14	3637	60	Placebo	0	109	2/13/2020	125.1	HEIGHT	23.6	16.1		0	1
14	3639	0	Placebo	0	36	4/7/2015	94.3	HEIGHT	15.05	16.5		1	1
14	3639	12	Placebo	0	58	7/23/2016	104.5	HEIGHT		16.9		1	1
14	3639	24	Placebo	0	60	5/4/2017	109.5	HEIGHT	18.13636364	15		0	1
14	3639	48	Placebo	0	91	5/22/2019	123	HEIGHT	23.05	15.5		0	1
14	3639	60	Placebo	0	101	2/13/2020	126.7	HEIGHT	24.15	15.3		0	1
14	3640	12	Placebo	1	51	7/23/2016	111.9	HEIGHT		14.6		1	1
14	3641	0	Placebo	0	54	4/7/2015	92.3	HEIGHT	12.75	15		0	1
14	3641	12	Placebo	0	51	7/23/2016	98.2	HEIGHT		14.1		0	1
14	3641	24	Placebo	0	64	5/4/2017	101.4	HEIGHT	15.13636364	15		0	1
14	3641	36	Placebo	0	152	6/3/2018	106.6	HEIGHT	16.35	15		0	1
14	3641	60	Placebo	0	172	2/13/2020	112.8	HEIGHT	18.22727273	15		0	1
14	3643	0	Placebo	1	12	4/7/2015	75.3	LENGTH	8.6	14	24	1	1
14	3643	12	Placebo	1	30	7/23/2016	96	HEIGHT		14	24	0	1
14	3648	0	Placebo	1	48	6/14/2015	105.1	HEIGHT	16.6	15		1	1
14	3648	48	Placebo	1	127	5/22/2019	128.9	HEIGHT	24.1	15		0	1
14	3649	0	Placebo	1	8	4/7/2015	73.3	LENGTH	9.1	14.5		1	1
14	3649	36	Placebo	1	44	6/3/2018	100.4	HEIGHT	16	15.5		0	1
14	3649	48	Placebo	1	55	5/22/2019	104.8	HEIGHT	17.55	15		1	1
14	3652	0	Placebo	0	48	4/7/2015	111.3	HEIGHT	18.85	16		1	1
14	3652	12	Placebo	0	62	7/23/2016	116.2	HEIGHT		17.1		0	1
14	3652	24	Placebo	0	75	5/4/2017	121.4	HEIGHT	23.22727273	17		0	1
14	3652	36	Placebo	0	88	6/3/2018	125.5	HEIGHT	25.1	17.5		0	1
14	3652	60	Placebo	0	108	2/13/2020	131.3	HEIGHT	29.25	18.5		0	1
14	3653	0	Placebo	1	4	4/7/2015	65.6	LENGTH	6.35	13.5	18	0	1
14	3653	12	Placebo	1	18	7/23/2016	75.3	HEIGHT		14	18	0	1
14	3655	0	Placebo	1	42	4/7/2015	100	HEIGHT	13.65	14.5		1	1
14	3656	0	Placebo	0	48	4/7/2015	87.3	HEIGHT	11.4	15		0	1
14	3656	12	Placebo	0	39	7/23/2016	99.5	HEIGHT		16		1	1
14	3658	0	Placebo	1	48	4/7/2015	107	HEIGHT	15.2	14		1	1

14	3658	24	Placebo	1	79	5/4/2017	120.1	HEIGHT	18.5	14.5	0	1
14	3658	36	Placebo	1	92	6/3/2018	124.7	HEIGHT	20.05	15	0	1
14	3658	48	Placebo	1	99	5/22/2019	128.7	HEIGHT	24.05	17	0	1
14	3660	12	Placebo	1	7	7/23/2016	71.7	LENGTH		14.5	0	1
14	3660	48	Placebo	1	39	5/22/2019	94.9	HEIGHT	13.9	15	1	1
14	3663	0	Placebo	1	48	4/7/2015	105.4	HEIGHT	15.3	14.5	0	1
14	3663	12	Placebo	1	58	7/23/2016	111.7	HEIGHT		15	1	1
14	3663	24	Placebo	1	67	5/4/2017	118.5	HEIGHT	19.13636364	14.5	0	1
14	3663	36	Placebo	1	80	6/3/2018	124	HEIGHT	20.5	14.5	0	1
14	3663	48	Placebo	1	91	5/22/2019	127.1	HEIGHT	22.05	15	0	1
14	3663	60	Placebo	1	100	2/13/2020	130.9	HEIGHT	23.95	15.2	0	1
14	3664	12	Placebo	0	51	7/23/2016	112.5	HEIGHT		15.8	1	1
14	3667	0	Placebo	1	36	4/7/2015	84.3	HEIGHT	9.4	13.5	1	1
14	3667	48	Placebo	1	79	5/22/2019	116.1	HEIGHT	16.65	14.5	0	1
14	3668	12	Placebo	1	36	7/23/2016	87.6	HEIGHT		14.3	1	1
14	3669	12	Placebo	1	6	7/23/2016	68.9	LENGTH		16	1	1
14	3669	24	Placebo	1	16	5/4/2017	77.6	HEIGHT	10.40909091	15.5	1	1
14	3669	60	Placebo	1	49	2/13/2020	102.9	HEIGHT	16.05	15.2	0	1
14	3670	0	Placebo	1	54	4/7/2015	119.3	HEIGHT	18.6	15.5	0	1
14	3670	36	Placebo	1	128	6/3/2018	135.5	HEIGHT	24.7	15.5	0	1
14	3670	60	Placebo	1	148	2/13/2020	144.1	HEIGHT	30.15	17	0	1
14	3671	0	Placebo	0	48	4/7/2015	85.9	HEIGHT	12.05	14.5	0	1
14	3671	12	Placebo	0	58	7/23/2016	94	HEIGHT		15.2	0	1
14	3671	48	Placebo	0	91	5/22/2019	112.7	HEIGHT	17.8	14.5	0	1
14	3675	24	Placebo	0	55	5/4/2017	102.2	HEIGHT	16.18181818	16	1	1
14	3676	0	Placebo	1	24	4/7/2015	82.2	HEIGHT	9.9	14.5	0	1
14	3676	12	Placebo	1	39	7/23/2016	90.3	HEIGHT		14.5	0	1
14	3676	24	Placebo	1	52	5/4/2017	96.9	HEIGHT	12.95454545	13.5	1	1
14	3676	36	Placebo	1	65	6/3/2018	105.2	HEIGHT	15.1	15	0	1
14	3676	48	Placebo	1	76	5/22/2019	112.9	HEIGHT	17.6	15	0	1
14	3676	60	Placebo	1	85	2/13/2020	117.9	HEIGHT	19.25	15.3	0	1
14	3679	12	Placebo	0	51	7/23/2016	111.3	HEIGHT		15.3	1	1
14	3680	12	Placebo	0	2	7/23/2016	64.4	LENGTH		15.6	0	1
14	3680	24	Placebo	0	12	5/4/2017	73.1	LENGTH	8.545454545	13.5	0	1
14	3680	36	Placebo	0	25	6/3/2018	83.3	HEIGHT	11.25	14.5	0	1
14	3680	60	Placebo	0	45	2/13/2020	96.2	HEIGHT	14.25	14.5	1	1
14	3682	0	Placebo	0	43	4/7/2015	105.2	HEIGHT	14.9	14.5	0	1
14	3684	12	Placebo	1	11	7/23/2016	63.3	LENGTH		13	1	1
14	3684	24	Placebo	1	19	5/4/2017	75.5	LENGTH	9.363636364	13.5	0	1
14	3684	48	Placebo	1	43	5/22/2019	96.3	HEIGHT	14	14.5	0	1

14	3684	60	Placebo	1	52	2/13/2020	102.6	HEIGHT	14.85	13.7	0	1
14	3685	12	Placebo	1	10	7/23/2016	66.8	LENGTH		13.8	0	1
14	3685	24	Placebo	1	16	5/4/2017	76	HEIGHT	9.090909091	13	0	1
14	3685	60	Placebo	1	49	2/13/2020	101.4	HEIGHT	14.63636364	13.5	0	1
14	3688	0	Placebo	0	2	4/7/2015	67.2	LENGTH	8.1	14	1	1
14	3690	0	Placebo	0	48	4/7/2015	108.9	HEIGHT	15.05	14	1	1
14	3690	12	Placebo	0	63	7/23/2016	115.6	HEIGHT		15.6	0	1
14	3690	24	Placebo	0	76	5/4/2017	120.5	HEIGHT	21.27272727	15	0	1
14	3690	36	Placebo	0	103	6/3/2018	127.2	HEIGHT	22.05	15.5	0	1
14	3690	60	Placebo	0	123	2/13/2020	133.8	HEIGHT	28.2	17	0	1
14	3691	0	Placebo	0	48	4/7/2015	100.1	HEIGHT	14.25	15.5	0	1
14	3691	12	Placebo	0	63	7/23/2016	106.2	HEIGHT		15.6	0	1
14	3691	24	Placebo	0	76	5/4/2017	111.4	HEIGHT	17.27272727	15	0	1
14	3691	36	Placebo	0	89	6/3/2018	115.2	HEIGHT	19.05	15.5	0	1
14	3691	48	Placebo	0	100	5/22/2019	120.2	HEIGHT	20.7	16	0	1
14	3691	60	Placebo	0	109	2/13/2020	123.3	HEIGHT	23.75	17	0	1
14	3692	0	Placebo	1	7	4/7/2015	67.5	LENGTH	7.9	15	0	1
14	3692	24	Placebo	1	33	5/4/2017	83.5	HEIGHT	10.86363636	13	0	1
14	3695	0	Placebo	0	48	4/7/2015	89.6	HEIGHT	11.8	13.5	0	1
14	3695	24	Placebo	0	75	5/4/2017	103.3	HEIGHT	14.22727273	13	0	1
14	3695	36	Placebo	0	88	6/3/2018	109.2	HEIGHT	16.2	13.5	0	1
14	3695	48	Placebo	0	99	5/22/2019	114.3	HEIGHT	17.35	14	0	1
14	3695	60	Placebo	0	108	2/13/2020	118.2	HEIGHT	19.1	14.3	0	1
14	3696	12	Placebo	1	9	7/23/2016	73.4	HEIGHT		13.5	0	1
14	3696	24	Placebo	1	22	5/4/2017	81.8	HEIGHT	10.81818182	14.5	0	1
14	3696	36	Placebo	1	35	6/3/2018	92.6	HEIGHT	13.05	14.5	0	1
14	3696	60	Placebo	1	55	2/13/2020	105.7	HEIGHT	16.25	14.4	0	1
14	3701	12	Placebo	1	42	7/23/2016	104.5	HEIGHT		14.5	1	1
14	3702	12	Placebo	0	39	7/23/2016	109.6	HEIGHT		15.6	1	1
14	3704	24	Placebo	1	9	5/4/2017	60.4	LENGTH	4.636363636	9	1	1
14	3704	36	Placebo	1	21	6/3/2018	67.5	LENGTH	5.45	9.5	0	1
14	3704	60	Placebo	1	41	2/13/2020	75.8	LENGTH	9.25	12	0	1
14	3706	0	Placebo	0	36	4/7/2015	84.1	HEIGHT	10.4	13.5	0	1
14	3706	12	Placebo	0	51	7/23/2016	94.6	HEIGHT		13.7	0	1
14	3706	24	Placebo	0	64	5/4/2017	99.4	HEIGHT	13.86363636	13.5	0	1
14	3706	36	Placebo	0	77	6/3/2018	106.2	HEIGHT	15.45	14	0	1
14	3706	60	Placebo	0	97	2/13/2020	116.1	HEIGHT	18.68181818	13.9	0	1
14	3708	0	Placebo	0	36	4/7/2015	94.4	HEIGHT	13.9	15	0	1
14	3708	12	Placebo	0	50	7/23/2016	102.5	HEIGHT		16.8	0	1
14	3708	24	Placebo	0	63	5/4/2017	109.5	HEIGHT	19	16	0	1

14	3708	36	Placebo	0	76	6/3/2018	117.7	HEIGHT	22.15	17	0	1
14	3708	48	Placebo	0	87	5/22/2019	122.7	HEIGHT	24	17	0	1
14	3708	60	Placebo	0	97	2/13/2020	125.5	HEIGHT	26.4	17.5	0	1
14	3709	0	Placebo	0	24	4/7/2015	74.7	HEIGHT	8.55	14	1	1
14	3709	12	Placebo	1	30	7/23/2016	83.5	HEIGHT		14.3	1	1
14	3709	24	Placebo	0	43	5/4/2017	91.9	HEIGHT	12.59090909	14	0	1
14	3709	36	Placebo	1	57	6/3/2018	99.7	HEIGHT	14.6	14.5	1	1
14	3709	48	Placebo	1	67	5/22/2019	105.1	HEIGHT	15.8	14	0	1
14	3709	60	Placebo	1	76	2/13/2020	110.4	HEIGHT	17.55	14	0	1
14	3710	12	Placebo	1	8	7/23/2016	68.5	HEIGHT		13	0	1
14	3710	24	Placebo	1	16	5/4/2017	74.3	HEIGHT	7.363636364	11.5	0	1
14	3710	36	Placebo	1	29	6/3/2018	80.3	HEIGHT	9.65	14	0	1
14	3710	60	Placebo	1	49	2/13/2020	95	HEIGHT	13.15	14.6	0	1
14	3711	0	Placebo	1	36	4/7/2015	91.7	HEIGHT	10.35	13	0	1
14	3711	12	Placebo	1	51	7/23/2016	102.3	HEIGHT		13.6	0	1
14	3711	24	Placebo	1	64	5/4/2017	107.7	HEIGHT	13.22727273	12.5	0	1
14	3711	36	Placebo	1	77	6/3/2018	113.3	HEIGHT	15.1	13	0	1
14	3711	60	Placebo	1	97	2/13/2020	123.8	HEIGHT	18.95	14.2	0	1
14	3712	0	Placebo	0	12	4/7/2015	73	HEIGHT	6.8	12	0	1
14	3713	0	Placebo	0	36	4/7/2015	99.8	HEIGHT	15.15	15	1	1
14	3713	12	Placebo	0	51	7/23/2016	107	HEIGHT		15	0	1
14	3713	24	Placebo	0	64	5/4/2017	112.8	HEIGHT	18.27272727	15.5	0	1
14	3713	36	Placebo	0	77	6/3/2018	117.7	HEIGHT	19.5	15.5	0	1
14	3713	48	Placebo	0	88	5/22/2019	120.9	HEIGHT	21.6	16	0	1
14	3713	60	Placebo	0	97	2/13/2020	125.1	HEIGHT	23.1	16	0	1
14	3714	0	Placebo	0	24	4/7/2015	76.2	HEIGHT	9.25	14	0	1
14	3714	12	Placebo	0	30	7/23/2016	98.4	HEIGHT		17.2	1	1
14	3714	48	Placebo	0	67	5/22/2019	109.8	HEIGHT	18.05	16.5	0	1
14	3714	60	Placebo	0	76	2/13/2020	113.9	HEIGHT	19.2	16	0	1
14	3716	0	Placebo	1	36	4/7/2015	107.9	HEIGHT	17.8	16.5	0	1
14	3717	12	Placebo	0	11	7/23/2016	76.2	LENGTH		14.5	0	1
14	3717	48	Placebo	0	48	5/22/2019	98.5	HEIGHT	15	15	1	1
14	3717	60	Placebo	0	57	2/13/2020	101.6	HEIGHT	15.9	15.6	0	1
14	3718	0	Placebo	1	36	4/7/2015	90.5	HEIGHT	12.65	14	1	1
14	3720	0	Placebo	1	8	4/7/2015	69.5	LENGTH	6.3	13	0	1
14	3720	12	Placebo	1	18	7/23/2016	78.5	HEIGHT		12.8	0	1
14	3720	24	Placebo	1	31	5/4/2017	85.1	HEIGHT	10.59090909	14	0	1
14	3721	0	Placebo	0	48	4/7/2015	93	HEIGHT	13.5	14.5	0	1
14	3721	24	Placebo	0	67	5/4/2017	105.4	HEIGHT	17.18181818	15	0	1
14	3721	48	Placebo	0	91	5/22/2019	115.9	HEIGHT	19.95	15	0	1

14	3721	60	Placebo	0	100	2/13/2020	117.8	HEIGHT	21.55	15		0	1
14	3722	24	Placebo	1	33	5/4/2017	87.8	HEIGHT	12.81818182	15		1	1
14	3722	36	Placebo	1	46	6/3/2018	97.4	HEIGHT	15.8	16		1	1
14	3724	24	Placebo	0	14	5/6/2017	72.6	LENGTH	7.727272727	13		1	1
14	3724	36	Placebo	0	27	6/3/2018	82	HEIGHT	9.6	13		1	1
14	3724	60	Placebo	0	47	2/13/2020	97.9	HEIGHT	13.4	13.4		1	1
14	3726	12	Placebo	0	42	7/23/2016	89.2	HEIGHT		16		1	1
14	3727	0	Placebo	0	36	4/7/2015	98.4	HEIGHT	13.3	14.5		1	1
14	3727	12	Placebo	0	42	7/23/2016	106.2	HEIGHT		15		0	1
14	3727	36	Placebo	0	68	6/3/2018	116.1	HEIGHT	18.75	14.5		0	1
14	3727	48	Placebo	0	79	5/22/2019	120.3	HEIGHT	20.2	15		0	1
14	3727	60	Placebo	0	88	2/13/2020	123.3	HEIGHT	22.5	16		0	1
14	3729	0	Placebo	0	7	4/7/2015	68.6	LENGTH	8.45	13		0	1
14	3729	12	Placebo	0	23	7/23/2016	81.3	HEIGHT		14.5		0	1
14	3729	24	Placebo	0	36	5/4/2017	89.3	HEIGHT	12.04545455	15		1	1
14	3729	60	Placebo	0	69	2/13/2020	108	HEIGHT	17	14.3		0	1
14	3732	0	Placebo	1	2	4/7/2015	66.1	LENGTH	7.45	13.5		0	1
14	3732	12	Placebo	1	22	7/23/2016	82	HEIGHT		14.6		0	1
14	3733	24	Placebo	0	9	5/4/2017	67.4	LENGTH	5.954545455	12		1	1
14	3733	60	Placebo	0	42	2/13/2020	93.6	HEIGHT	13.77272727	14.5		1	1
14	3734	0	Placebo	1	48	4/7/2015	101.3	HEIGHT	14	14.5		0	1
14	3734	12	Placebo	1	63	7/23/2016	111.1	HEIGHT		15.6		0	1
14	3734	24	Placebo	1	76	5/4/2017	116.3	HEIGHT	18.90909091	16		0	1
14	3734	36	Placebo	1	89	6/3/2018	122.4	HEIGHT	19.85	15		0	1
14	3734	48	Placebo	1	100	5/22/2019	137	HEIGHT	26.5	17		0	1
14	3734	60	Placebo	1	109	2/13/2020	128.9	HEIGHT	23.95	16		0	1
14	3735	12	Placebo	0	39	7/23/2016	93.2	HEIGHT		15.5		1	1
14	3738	0	Placebo	1	48	4/7/2015	104.1	HEIGHT	14.85	13.5		0	1
14	3738	12	Placebo	1	63	7/23/2016	111.8	HEIGHT		14.6		0	1
14	3738	24	Placebo	1	76	5/4/2017	117.8	HEIGHT	19.81818182	14		0	1
14	3738	60	Placebo	1	109	2/13/2020	129.1	HEIGHT	24.75	15.4		0	1
14	3742	12	Placebo	1	10	7/23/2016	74.8	HEIGHT		12.6		0	1
14	3742	24	Placebo	1	22	5/4/2017	82.8	HEIGHT	10.31818182	13.5		0	1
14	3742	36	Placebo	1	35	6/3/2018	90.7	HEIGHT	12.25	13.5		0	1
14	3744	24	Placebo	0	31	5/4/2017	85.3	HEIGHT	11.40909091	14.5	42	1	1
14	3745	0	Placebo	1	36	4/7/2015	78.4	HEIGHT	10.4	14		1	1
14	3746	12	Placebo	1	12	7/23/2016	79	HEIGHT		14.5		1	1
14	3746	24	Placebo	1	25	5/4/2017	86	HEIGHT	11.68181818	14		1	1
14	3746	36	Placebo	1	38	6/3/2018	95.7	HEIGHT	14.15	14.5		0	1
14	3746	48	Placebo	1	48	5/22/2019	102.9	HEIGHT	15.35	15		0	1

14	3746	60	Placebo	1	58	2/13/2020	107.6	HEIGHT	16.4	14	0	1
14	3748	0	Placebo	1	5	4/7/2015	62.6	LENGTH	6.15	13	0	1
14	3748	12	Placebo	1	15	7/23/2016	74.7	HEIGHT		13.5	0	1
14	3748	24	Placebo	1	28	5/4/2017	81.5	HEIGHT	10.68181818	13.5	0	1
14	3748	36	Placebo	1	41	6/3/2018	90.8	HEIGHT	12.45	14	0	1
14	3748	48	Placebo	1	52	5/22/2019	96.8	HEIGHT	14.7	14.5	0	1
14	3748	60	Placebo	1	61	2/13/2020	102.4	HEIGHT	15.85	14.2	0	1
14	3750	0	Placebo	0	48	4/7/2015	100.2	HEIGHT	13.45	15	1	1
14	3750	12	Placebo	0	54	7/23/2016	105.6	HEIGHT		15.9	0	1
14	3750	24	Placebo	0	67	5/4/2017	110.8	HEIGHT	15.77272727	15.5	0	1
14	3750	36	Placebo	0	80	6/3/2018	115	HEIGHT	17.4	16	0	1
14	3750	48	Placebo	0	91	5/22/2019	118.6	HEIGHT	18.2	16	0	1
14	3751	12	Placebo	0	50	7/23/2016	98.5	HEIGHT		14.9	1	1
14	3754	0	Placebo	0	36	6/14/2015	112.1	HEIGHT	17.95	15	1	1
14	3754	12	Placebo	0	86	7/23/2016	117	HEIGHT		15.4	0	1
14	3754	36	Placebo	0	112	6/3/2018	126.4	HEIGHT	21.8	15.5	0	1
14	3754	48	Placebo	0	123	5/22/2019	132.9	HEIGHT	25.3	16.5	0	1
14	3755	0	Placebo	0	36	4/7/2015	93.5	HEIGHT	13.05	14	1	1
14	3755	12	Placebo	0	54	7/23/2016	103.8	HEIGHT		14.5	1	1
14	3755	24	Placebo	0	67	5/4/2017	110.8	HEIGHT	17.36363636	14	0	1
14	3755	48	Placebo	0	91	5/22/2019	125.4	HEIGHT	22	15	0	1
14	3755	60	Placebo	0	100	2/13/2020	128.4	HEIGHT	23	15	0	1
14	3757	0	Placebo	0	18	4/7/2015	80.2	HEIGHT	8.85	13	0	1
14	3757	36	Placebo	0	56	6/3/2018	102.5	HEIGHT	15.5	13.5	0	1
14	3757	48	Placebo	0	67	5/22/2019	109.9	HEIGHT	17.45	14.5	0	1
14	3757	60	Placebo	0	76	2/13/2020	115.1	HEIGHT	18.25	14	0	1
14	3759	24	Placebo	1	43	5/4/2017	93	HEIGHT	13.09090909	16	1	1
14	3761	12	Placebo	1	6	7/23/2016	65.6	LENGTH		12.4	1	1
14	3761	60	Placebo	1	49	2/13/2020	95.4	HEIGHT	13.1	13.5	0	1
14	3762	0	Placebo	1	18	4/7/2015	74.3	HEIGHT	8.4	13.5	0	1
14	3762	12	Placebo	1	33	7/23/2016	82.3	HEIGHT		15.3	0	1
14	3762	24	Placebo	1	46	5/4/2017	88	HEIGHT	13.31818182	15.5	1	1
14	3762	36	Placebo	1	59	6/3/2018	94.1	HEIGHT	14.85	15	0	1
14	3762	48	Placebo	1	52	5/22/2019	101.2	HEIGHT	16.65	15	0	1
14	3763	24	Placebo	0	49	5/4/2017	104.5	HEIGHT	19.54545455	17	1	1
14	3764	0	Placebo	1	12	4/7/2015	70.1	LENGTH	7.05	13	0	1
14	3764	12	Placebo	1	27	7/23/2016	76.5	HEIGHT		13.5	0	1
14	3764	24	Placebo	1	40	5/4/2017	82.3	HEIGHT	10	14	0	1
14	3764	48	Placebo	1	51	5/22/2019	98.6	HEIGHT	14.95	15	0	1
14	3769	12	Placebo	1	4	7/23/2016	64	HEIGHT		11.8	0	1

14	3769	36	Placebo	1	29	6/3/2018	79.2	HEIGHT	8.35	12.5		0	1
14	3772	24	Placebo	1	55	5/4/2017	103.7	HEIGHT	16.72727273	15.5		1	1
14	3775	12	Placebo	0	8	7/23/2016	71.6	HEIGHT		11.5	24	0	1
14	3776	0	Placebo	1	54	4/7/2015	102.3	HEIGHT	15.11	13		0	1
14	3776	24	Placebo	1	69	5/4/2017	113.4	HEIGHT	18.40909091	14		0	1
14	3777	0	Placebo	0	48	4/7/2015	103.9	HEIGHT	16.35	15.5		1	1
14	3777	12	Placebo	0	63	7/23/2016	112.9	HEIGHT		16.7		0	1
14	3777	48	Placebo	0	100	5/22/2019	128.9	HEIGHT	26.5	17.5		0	1
14	3778	12	Placebo	1	6	7/23/2016	67.8	LENGTH		13		0	1
14	3778	24	Placebo	1	16	5/4/2017	72.8	LENGTH	8.454545455	14		1	1
14	3778	36	Placebo	1	29	6/3/2018	81.5	HEIGHT	10.6	15		0	1
14	3778	48	Placebo	1	39	5/22/2019	85.8	HEIGHT	12.65	16.5		1	1
14	3782	12	Placebo	1	54	7/23/2016	101.5	HEIGHT		17.7		1	1
14	3784	0	Placebo	1	18	4/7/2015	77.4	LENGTH	9.5	14		0	1
14	3784	12	Placebo	1	30	7/23/2016	88.9	HEIGHT		15.4		0	1
14	3784	24	Placebo	1	43	5/4/2017	97	HEIGHT	14.31818182	15.5		0	1
14	3784	48	Placebo	1	67	5/22/2019	110.8	HEIGHT	16.85	15		0	1
14	3786	12	Placebo	0	10	7/23/2016	74.2	HEIGHT		13.5		0	1
14	3786	24	Placebo	0	24	5/4/2017	83.1	HEIGHT	10.54545455	15.5		0	1
14	3786	36	Placebo	0	38	6/3/2018	92.7	HEIGHT	12.8	15.5		0	1
14	3787	12	Placebo	1	42	7/23/2016	80.7	HEIGHT		14.6	60	1	1
14	3788	0	Placebo	0	36	4/7/2015	94.4	HEIGHT	11.95	14.5		1	1
14	3788	12	Placebo	0	51	7/23/2016	100.6	HEIGHT		14.4		0	1
14	3788	60	Placebo	0	107	2/13/2020	121.3	HEIGHT	19.45454545	14.4		0	1
14	3789	0	Placebo	0	24	6/14/2015	81.1	HEIGHT	10.45	12.5		1	1
14	3789	48	Placebo	0	75	5/22/2019	108.3	HEIGHT	16.3	14		0	1
14	3790	12	Placebo	0	5	7/23/2016	75	HEIGHT		14.5		0	1
14	3790	24	Placebo	0	18	5/4/2017	81.6	LENGTH	11.45454545	15		0	1
14	3790	36	Placebo	0	32	6/3/2018	92.2	HEIGHT	14.65	15.5		0	1
14	3790	60	Placebo	0	52	2/13/2020	106.6	HEIGHT	19.15	16		0	1
14	3793	0	Placebo	0	18	4/7/2015	87	HEIGHT	10.45	14		1	1
14	3793	12	Placebo	0	42	7/23/2016	94.2	HEIGHT		14		0	1
14	3793	24	Placebo	0	55	5/4/2017	104.6	HEIGHT	15.86363636	15.5		0	1
14	3793	36	Placebo	0	68	6/3/2018	113.8	HEIGHT	17.85	15		0	1
14	3793	60	Placebo	0	88	2/13/2020	125.3	HEIGHT	21.45	15		0	1
14	3795	0	Placebo	0	36	4/7/2015	91.7	HEIGHT	13.95	15.5		0	1
14	3795	12	Placebo	0	54	7/23/2016	98.9	HEIGHT		16.1		0	1
14	3795	24	Placebo	0	67	5/4/2017	105.3	HEIGHT	16.31818182	15.5		0	1
14	3795	36	Placebo	0	80	6/3/2018	112	HEIGHT	17.8	15		0	1
14	3795	60	Placebo	0	101	2/13/2020	119	HEIGHT	20.5	15.2		0	1

14	3799	12	Placebo	1	-3	7/23/2016	59.6	LENGTH		14.7	0	1
14	3799	36	Placebo	1	21	6/3/2018	84.3	HEIGHT	10.8	15	0	1
14	3799	60	Placebo	1	41	2/13/2020	96.8	HEIGHT	13.27272727	14	0	1
14	3800	24	Placebo	1	52	5/4/2017	89.7	HEIGHT	12.90909091	15.5	1	1
14	3801	12	Placebo	1	48	7/23/2016	91.9	HEIGHT		13.7	1	1
14	3805	12	Placebo	0	58	7/23/2016	98.6	HEIGHT		13.5	1	1
14	3806	12	Placebo	1	58	7/23/2016	76	HEIGHT		14.5	1	1
14	3807	0	Placebo	1	24	4/7/2015	88.8	HEIGHT	13	15.5	0	1
14	3807	36	Placebo	1	80	6/3/2018	111.7	HEIGHT	18.7	16	0	1
14	3807	48	Placebo	1	91	5/22/2019	117.5	HEIGHT	20.75	16	0	1
14	3807	60	Placebo	1	100	2/13/2020	121.2	HEIGHT	23.3	16.7	0	1
14	3809	0	Placebo	0	24	4/7/2015	91.5	HEIGHT	11.65	14	0	1
14	3809	12	Placebo	0	33	7/23/2016	102	HEIGHT		14.6	0	1
14	3809	24	Placebo	0	43	5/4/2017	108.4	HEIGHT	16.40909091	15	1	1
14	3809	48	Placebo	0	67	5/22/2019	119.5	HEIGHT	19.7	15	0	1
14	3809	60	Placebo	0	76	2/13/2020	123.2	HEIGHT	22.2	15.5	0	1
14	3811	12	Placebo	0	7	7/23/2016	65	LENGTH		11.8	0	1
14	3811	24	Placebo	0	15	5/4/2017	75.7	HEIGHT	9.636363636	14	0	1
14	3811	36	Placebo	0	33	6/3/2018	83.6	HEIGHT	13.6	16	0	1
14	3811	60	Placebo	0	53	2/13/2020	100.6	HEIGHT	17.9	16.2	0	1
14	3813	0	Placebo	0	24	4/7/2015	78	HEIGHT	9.2	14	0	1
14	3813	12	Placebo	0	41	7/23/2016	84.8	HEIGHT		14.5	0	1
14	3813	24	Placebo	0	51	5/6/2017	92.5	HEIGHT	12.90909091	14	1	1
14	3813	60	Placebo	0	85	2/13/2020	109.3	HEIGHT	17.45	14.1	0	1
14	3820	0	Placebo	1	18	4/7/2015	77.2	LENGTH	7.75	12.5	0	1
14	3820	12	Placebo	1	35	7/23/2016	84.7	HEIGHT		13.9	0	1
14	3820	36	Placebo	1	58	6/3/2018	100.6	HEIGHT	12.8	13.5	0	1
14	3820	48	Placebo	1	69	5/22/2019	106.3	HEIGHT	14.25	13.5	0	1
14	3820	60	Placebo	1	78	2/13/2020	110.5	HEIGHT	15.25	13.9	0	1
14	3823	12	Placebo	0	3	7/23/2016	63.3	LENGTH		12.2	0	1
14	3823	24	Placebo	0	15	5/4/2017	76.5	HEIGHT	8.727272727	14	0	1
14	3823	36	Placebo	0	29	6/3/2018	88.3	HEIGHT	11.05	14	0	1
14	3823	60	Placebo	0	49	2/13/2020	101.8	HEIGHT	14.35	12.7	0	1
14	3824	0	Placebo	0	24	4/7/2015	93	HEIGHT	12.75	15.5	0	1
14	3824	36	Placebo	0	65	6/3/2018	109.9	HEIGHT	16.4	14.5	0	1
14	3824	48	Placebo	0	76	5/22/2019	113.2	HEIGHT	17.1	14	0	1
14	3824	60	Placebo	0	85	2/13/2020	115.4	HEIGHT	18.6	15.2	0	1
14	3825	0	Placebo	0	3	4/7/2015	64.3	HEIGHT	6.35	13	0	1
14	3825	12	Placebo	0	15	7/23/2016	75.8	HEIGHT		14.5	1	1
14	3825	24	Placebo	0	28	5/4/2017	80.1	HEIGHT	10.63636364	15.5	1	1

14	3825	36	Placebo	0	45	6/3/2018	88.2	HEIGHT	12.8	15	0	1
14	3825	60	Placebo	0	53	2/13/2020	99.5	HEIGHT	15.31818182	14.8	1	1
14	3831	24	Placebo	0	21	5/4/2017	81.7	HEIGHT	10.63636364	13.5	1	1
14	3834	24	Placebo	1	58	5/4/2017	103.7	HEIGHT	14.77272727	14	1	1
14	3837	0	Placebo	1	48	4/7/2015	108.9	HEIGHT	16.25	15	1	1
14	3837	12	Placebo	1	102	7/23/2016	115.9	HEIGHT		15	0	1
14	3837	24	Placebo	1	115	5/4/2017	122.3	HEIGHT	21.27272727	16	0	1
14	3837	36	Placebo	1	128	6/3/2018	128.5	HEIGHT	23	16.5	0	1
14	3837	48	Placebo	1	139	5/22/2019	132.2	HEIGHT	25.05	16	0	1
14	3837	60	Placebo	1	148	2/13/2020	136.7	HEIGHT	27.04545455	17.2	0	1
14	3840	12	Placebo	1	8	7/23/2016	72.2	HEIGHT		14.3	0	1
14	3840	24	Placebo	1	21	5/4/2017	79.1	HEIGHT	9.909090909	13.5	0	1
14	3840	36	Placebo	1	34	6/3/2018	88.7	HEIGHT	11.45	13.5	1	1
14	3840	60	Placebo	1	54	2/13/2020	98.4	HEIGHT	13.7	13.5	1	1
14	3841	0	Placebo	0	36	4/7/2015	97.9	HEIGHT	14.8	15	1	1
14	3841	24	Placebo	0	64	5/4/2017	108.8	HEIGHT	17.68181818	15	0	1
14	3841	36	Placebo	0	77	6/3/2018	114.3	HEIGHT	18.3	14	0	1
14	3841	48	Placebo	0	88	5/22/2019	118.4	HEIGHT	20.65	15	0	1
14	3842	0	Placebo	0	6	4/7/2015	65.1	LENGTH	5.65	11	1	1
14	3845	0	Placebo	0	18	4/7/2015	77.7	HEIGHT	8.95	14	0	1
14	3846	12	Placebo	0	-3	7/23/2016	62.3	LENGTH		13.5	1	1
14	3846	24	Placebo	0	11	5/4/2017	74.5	LENGTH	8.409090909	13	1	1
14	3846	36	Placebo	0	24	6/3/2018	86.1	HEIGHT	11.15	13.5	0	1
14	3846	48	Placebo	0	34	5/22/2019	93	HEIGHT	13.95	14.5	0	1
14	3846	60	Placebo	0	44	2/13/2020	100.6	HEIGHT	15.2	14.5	1	1
14	3847	0	Placebo	1	6	4/7/2015	65.3	LENGTH	5.8	11.5	0	1
14	3847	12	Placebo	1	15	7/23/2016	81.1	HEIGHT		12.8	1	1
14	3847	24	Placebo	1	28	5/4/2017	87	HEIGHT	10.81818182	14	1	1
14	3847	48	Placebo	1	52	5/22/2019	98.4	HEIGHT	13.9	15	1	1
14	3847	60	Placebo	1	54	2/13/2020	105.3	HEIGHT	15.09090909	14.2	0	1
14	3850	12	Placebo	0	42	7/23/2016	100.2	HEIGHT		16	1	1
14	3851	24	Placebo	1	46	5/4/2017	90.2	HEIGHT	10.95454545	13.5	1	1
14	3852	24	Placebo	0	44	5/4/2017	99.6	HEIGHT	15.77272727	15	1	1
14	3853	24	Placebo	1	2	5/6/2017	60.4	LENGTH	7.136363636	15	1	1
14	3853	36	Placebo	1	15	6/3/2018	75.5	LENGTH	9.25	14.5	0	1
14	3853	48	Placebo	1	26	5/22/2019	84.8	HEIGHT	11.45	14	0	1
14	3853	60	Placebo	1	36	2/13/2020	90.9	HEIGHT	14.04545455	15	1	1
14	3854	0	Placebo	0	48	4/7/2015	87.4	HEIGHT	12.05	14.5	1	1
14	3854	24	Placebo	0	55	5/4/2017	103.3	HEIGHT	15.27272727	15	0	1
14	3854	36	Placebo	0	69	6/3/2018	109	HEIGHT	15.95	14	0	1

14	3854	48	Placebo	0	79	5/22/2019	113.5	HEIGHT	17.7	14		0	1
14	3854	60	Placebo	0	88	2/13/2020	117.7	HEIGHT	18.35	14.5		0	1
14	3855	0	Placebo	1	54	4/7/2015	102	HEIGHT	15.25	15		1	1
14	3855	12	Placebo	1	56	7/23/2016	108.4	HEIGHT		16		1	1
14	3856	0	Placebo	1	4	6/14/2015	65.3	HEIGHT	6.2	14	42	0	1
14	3858	12	Placebo	1	2	7/23/2016	69.3	LENGTH		14.8	30	0	1
14	3860	12	Placebo	1	8	7/23/2016	69.3	LENGTH		12		0	1
14	3860	24	Placebo	1	16	5/4/2017	73.4	HEIGHT	8.045454545	12.5		0	1
14	3860	36	Placebo	1	29	6/3/2018	82.1	HEIGHT	10.25	14		0	1
14	3860	60	Placebo	1	49	2/13/2020	97.9	HEIGHT	13.54545455	13.5		0	1
14	3861	0	Placebo	0	12	4/7/2015	76.6	LENGTH	10.3	15		1	1
14	3861	12	Placebo	0	36	7/23/2016	90.7	HEIGHT		16.3		0	1
14	3861	60	Placebo	0	82	2/13/2020	121	HEIGHT	21.5	14.5		0	1
14	3862	24	Placebo	0	52	5/6/2017	102.9	HEIGHT	14.22727273	13		1	1
14	3863	12	Placebo	0	8	7/23/2016	72.1	HEIGHT		13.8		0	1
14	3863	24	Placebo	0	16	5/4/2017	80.5	HEIGHT	9.818181818	12.5		1	1
14	3863	36	Placebo	0	35	6/3/2018	88.6	HEIGHT	13.5	16		0	1
14	3863	60	Placebo	0	55	2/13/2020	106.8	HEIGHT	19.35	16.5		0	1
14	3864	12	Placebo	1	42	7/23/2016	98.1	HEIGHT		16.9		1	1
14	3865	0	Placebo	0	24	4/7/2015	98	HEIGHT	13.7	15		0	1
14	3865	12	Placebo	0	54	7/23/2016	105.3	HEIGHT		15.1		0	1
14	3865	24	Placebo	0	67	5/4/2017	111.6	HEIGHT	16.95454545	15		0	1
14	3865	36	Placebo	0	81	6/3/2018	116.5	HEIGHT	18.35	14.5		0	1
14	3865	48	Placebo	0	91	5/22/2019	120.2	HEIGHT	18.9	15		0	1
14	3865	60	Placebo	0	100	2/13/2020	123.5	HEIGHT	21.05	14.5		0	1
14	3866	12	Placebo	1	7	7/23/2016	71.2	HEIGHT		12.6		0	1
14	3866	24	Placebo	1	15	5/4/2017	80.8	HEIGHT	9	12.5		0	1
14	3867	0	Placebo	0	8	4/7/2015	72.7	LENGTH	9.3	15.5		0	1
14	3867	24	Placebo	0	34	5/4/2017	86.9	HEIGHT	13.59090909	16.5		0	1
14	3869	24	Placebo	0	51	5/4/2017	101.5	HEIGHT	15.68181818	15.5		1	1
14	3870	0	Placebo	1	24	4/7/2015	97.3	HEIGHT	13.55	13		0	1
14	3870	12	Placebo	1	39	7/23/2016	106.3	HEIGHT		15.5		0	1
14	3870	24	Placebo	1	52	5/4/2017	112	HEIGHT	17.86363636	15		0	1
14	3870	36	Placebo	1	65	6/3/2018	118.3	HEIGHT	19.45	15		0	1
14	3870	60	Placebo	1	85	2/13/2020	126.4	HEIGHT	23.2	16.2		0	1
14	3871	0	Placebo	0	48	4/7/2015	90.6	HEIGHT	12.9	15.5		0	1
14	3871	48	Placebo	0	99	5/22/2019	112.3	HEIGHT	18.9	15.5		0	1
14	3871	60	Placebo	0	108	2/13/2020	115.1	HEIGHT	20.7	16.3		0	1
14	3873	24	Placebo	1	2	5/4/2017	60.1	LENGTH	6.454545455	15		1	1
14	3873	48	Placebo	1	26	5/22/2019	84.8	HEIGHT	11	14		0	1

14	3873	60	Placebo	1	36	2/13/2020	90.9	HEIGHT	12.95	15.2	1	1
14	3874	12	Placebo	0	10	7/23/2016	78	HEIGHT		14.6	0	1
14	3874	24	Placebo	0	22	5/4/2017	83.6	HEIGHT	11.09090909	15.5	1	1
14	3874	36	Placebo	0	35	6/3/2018	92.5	HEIGHT	13.4	15	1	1
14	3876	24	Placebo	0	40	5/4/2017	96	HEIGHT	14.18181818	15.5	1	1
14	3876	36	Placebo	0	53	6/3/2018	105.3	HEIGHT	16.2	15	1	1
14	3880	0	Placebo	1	4	4/7/2015	67.3	LENGTH	7.2	13	0	1
14	3880	24	Placebo	1	27	5/4/2017	85.5	HEIGHT	11.90909091	13.5	0	1
14	3880	36	Placebo	1	41	6/3/2018	95.3	HEIGHT	14.35	14.5	1	1
14	3880	60	Placebo	1	61	2/13/2020	106.1	HEIGHT	17.7	15	0	1
14	3881	0	Placebo	1	6	4/7/2015	72.8	HEIGHT	8.85	15	0	1
14	3881	12	Placebo	1	18	7/23/2016	82	HEIGHT		13.6	0	1
14	3881	36	Placebo	1	44	6/3/2018	97.9	HEIGHT	14.55	16	0	1
14	3881	48	Placebo	1	54	5/22/2019	104.1	HEIGHT	15.05	14.5	0	1
14	3881	60	Placebo	1	56	2/13/2020	108.8	HEIGHT	16.65	15	0	1
14	3882	0	Placebo	1	24	4/7/2015	79.1	HEIGHT	9.5	14.5	0	1
14	3882	12	Placebo	1	38	7/23/2016	90.2	HEIGHT		15.9	0	1
14	3882	24	Placebo	1	51	5/4/2017	94.1	HEIGHT	13.95454545	15	0	1
14	3882	36	Placebo	1	64	6/3/2018	102.5	HEIGHT	15.1	15	0	1
14	3882	60	Placebo	1	84	2/13/2020	112.1	HEIGHT	19.7	16.2	0	1
14	3885	24	Placebo	0	10	5/4/2017	68.1	LENGTH	8.136363636	14	1	1
14	3885	60	Placebo	0	41	2/13/2020	88	HEIGHT	14.05	15.5	0	1
14	3888	0	Placebo	0	48	4/7/2015	105.1	HEIGHT	16.7	16	1	1
14	3888	36	Placebo	0	88	6/3/2018	122.3	HEIGHT	22.8	17	0	1
14	3888	48	Placebo	0	99	5/22/2019	125.9	HEIGHT	25.5	17.5	0	1
14	3888	60	Placebo	0	108	2/13/2020	128.1	HEIGHT	27.45	17	0	1
14	3890	0	Placebo	1	36	4/7/2015	83.3	HEIGHT	11.6	15.5	1	1
14	3890	12	Placebo	1	51	7/23/2016	90.8	HEIGHT		15.1	0	1
14	3890	24	Placebo	1	64	5/4/2017	95.1	HEIGHT	13.18181818	14	0	1
14	3890	36	Placebo	1	77	6/3/2018	101.2	HEIGHT	14.85	14.5	0	1
14	3890	60	Placebo	1	97	2/13/2020	107.6	HEIGHT	16.68181818	14.5	0	1
14	3891	0	Placebo	1	36	4/7/2015	87	HEIGHT	12.15	15	1	1
14	3891	48	Placebo	1	88	5/22/2019	112.9	HEIGHT	19	14.5	0	1
14	3892	24	Placebo	1	57	5/4/2017	99.5	HEIGHT	13.77272727	14	1	1
14	3897	0	Placebo	0	48	4/7/2015	110.7	HEIGHT	18.3	16	1	1
14	3897	12	Placebo	0	94	7/23/2016	115.4	HEIGHT		16.1	0	1
14	3897	24	Placebo	0	103	5/4/2017	121.7	HEIGHT	21.04545455	16	0	1
14	3897	48	Placebo	0	127	5/22/2019	128.8	HEIGHT	25	16.5	0	1
14	3897	60	Placebo	0	137	2/13/2020	134	HEIGHT	25.95	17	0	1
14	3900	24	Placebo	0	52	5/4/2017	105.7	HEIGHT	14.5	13.5	1	1

14	3902	24	Placebo	0	31	5/4/2017	84.9	HEIGHT	12.59090909	14.5	1	1
14	3903	0	Placebo	0	54	4/7/2015	108.2	HEIGHT	19.05	17	1	1
14	3903	48	Placebo	0	106	5/22/2019	129.9	HEIGHT	26.45	16.5	0	1
14	3905	0	Placebo	0	7	4/7/2015	71.5	LENGTH	7.05	12.5	0	1
14	3905	48	Placebo	0	57	5/22/2019	107	HEIGHT	14.2	13.5	1	1
14	3906	0	Placebo	0	36	4/7/2015	116	HEIGHT	20.75	16.5	0	1
14	3906	12	Placebo	0	51	7/23/2016	123	HEIGHT		18.4	0	1
14	3907	0	Placebo	1	36	4/7/2015	102	HEIGHT	14.7	14.5	1	1
14	3907	12	Placebo	1	67	7/23/2016	109.5	HEIGHT		15.5	0	1
14	3907	24	Placebo	1	76	5/4/2017	115.5	HEIGHT	18.72727273	15	0	1
14	3907	48	Placebo	1	100	5/22/2019	126.6	HEIGHT	22.35	16	0	1
14	3908	0	Placebo	1	36	4/7/2015	91.6	HEIGHT	12.75	15	0	1
14	3908	12	Placebo	1	42	7/23/2016	101.7	HEIGHT		15.2	0	1
14	3908	48	Placebo	1	85	5/22/2019	120.4	HEIGHT	20	15	0	1
14	3908	60	Placebo	1	94	2/13/2020	123.7	HEIGHT	21.86363636	15	0	1
14	3912	0	Placebo	1	54	4/7/2015	97.8	HEIGHT	12.5	14	0	1
14	3912	48	Placebo	1	101	5/22/2019	124.8	HEIGHT	21.9	15.5	0	1
14	3917	0	Placebo	1	54	4/7/2015	110.5	HEIGHT	17.95	16	0	1
14	3917	12	Placebo	1	69	7/23/2016	118.4	HEIGHT		16	0	1
14	3917	24	Placebo	1	82	5/4/2017	124.8	HEIGHT	22.45454545	16.5	0	1
14	3919	0	Placebo	1	18	4/7/2015	79.6	LENGTH	10.65	16	0	1
14	3919	12	Placebo	1	33	7/23/2016	91.2	HEIGHT		17	0	1
14	3919	36	Placebo	1	60	6/3/2018	103.4	HEIGHT	15.65	15	0	1
14	3919	48	Placebo	1	70	5/22/2019	109.7	HEIGHT	18.1	16	0	1
14	3925	12	Placebo	0	5	7/23/2016	65.5	LENGTH		12.1	0	1
14	3925	24	Placebo	0	12	5/4/2017	71.7	HEIGHT	7.318181818	12	0	1
14	3925	36	Placebo	0	25	6/3/2018	84.6	HEIGHT	10.95	14	0	1
14	3925	60	Placebo	0	45	2/13/2020	99.1	HEIGHT	13.6	14.2	0	1
14	3929	0	Placebo	0	8	4/7/2015	101.2	HEIGHT	15.8	15.5	0	1
14	3930	12	Placebo	0	9	7/23/2016	73.2	HEIGHT		15.1	1	1
14	3930	24	Placebo	0	22	5/4/2017	76.6	HEIGHT	9.818181818	14	0	1
14	3930	36	Placebo	0	35	6/3/2018	84.2	HEIGHT	11.65	15.5	0	1
14	3930	48	Placebo	0	46	5/22/2019	91.7	HEIGHT	13.85	15	0	1
14	3930	60	Placebo	0	55	2/13/2020	97.7	HEIGHT	15.65	15.9	0	1
14	3931	0	Placebo	0	30	4/7/2015	81.9	HEIGHT	10.95	14.5	0	1
14	3931	24	Placebo	0	58	5/4/2017	97.6	HEIGHT	13.95454545	13.5	0	1
14	3933	12	Placebo	1	8	7/23/2016	73.3	LENGTH		12	0	1
14	3933	24	Placebo	1	16	5/4/2017	82.9	HEIGHT	10.09090909	12.5	0	1
14	3933	36	Placebo	1	35	6/3/2018	90.9	HEIGHT	12.35	14	0	1
14	3933	48	Placebo	1	46	5/22/2019	98.7	HEIGHT	14.75	13.5	1	1

14	3934	0	Placebo	1	48	4/7/2015	91.7	HEIGHT	13.05	15.5		1	1
14	3934	12	Placebo	1	42	7/23/2016	100.1	HEIGHT		16.7		0	1
14	3934	24	Placebo	1	55	5/4/2017	106.9	HEIGHT	17.36363636	16		0	1
14	3934	36	Placebo	1	66	6/3/2018	112.1	HEIGHT	18.85	16		0	1
14	3934	48	Placebo	1	76	5/22/2019	115.2	HEIGHT	18.65	15		0	1
14	3934	60	Placebo	1	86	2/13/2020	119.2	HEIGHT	21.3	16.8		0	1
14	3936	0	Placebo	1	30	6/14/2015	81.9	HEIGHT	8.9	12.5		1	1
14	3936	12	Placebo	1	45	7/23/2016	90.5	LENGTH		14.8		0	1
14	3936	24	Placebo	1	58	5/4/2017	98.4	HEIGHT	14.13636364	14		0	1
14	3936	36	Placebo	1	70	6/3/2018	106.7	HEIGHT	16.3	15		0	1
14	3936	60	Placebo	1	90	2/13/2020	118.1	HEIGHT	19.8	14.5		0	1
14	3939	0	Placebo	0	24	4/7/2015	79.3	LENGTH	8.6	11.5		0	1
14	3939	12	Placebo	0	36	7/23/2016	89	HEIGHT		15.4		1	1
14	3939	24	Placebo	0	49	5/4/2017	94.8	HEIGHT	12.63636364	13.5		0	1
14	3939	36	Placebo	0	62	6/3/2018	102.5	HEIGHT	14.9	14		0	1
14	3939	60	Placebo	0	83	2/13/2020	112.6	HEIGHT	17.3	14.1		0	1
14	8035	36	Placebo	1	7	6/3/2018	67.7	LENGTH	6.9	12.5		1	1
14	8035	48	Placebo	1	18	5/22/2019	78.4	HEIGHT	9.2	13		0	1
14	8035	60	Placebo	1	27	2/13/2020	84.6	LENGTH	11.25	13.2		0	1
14	8037	48	Placebo	1	31	5/22/2019	84	LENGTH	12.5	15		1	1
14	8051	36	Placebo	1	25	6/3/2018	80.2	HEIGHT	10.75	14.5		1	1
14	8094	36	Placebo	0	19	6/3/2018	74.5	LENGTH	8.7	13		1	1
14	8094	60	Placebo	0	39	2/16/2020	90	HEIGHT	12.04545455	13		1	1
14	8127	48	Placebo	0	29	5/22/2019	90.6	HEIGHT	14.1	15		1	1
14	8142	60	Placebo	1	20	2/13/2020	80.3	LENGTH	10.3	14.4		1	1
14	8147	36	Placebo	1	35	6/3/2018	86.9	HEIGHT	11.85	14.5		1	1
14	8191	48	Placebo	1	21	5/22/2019	68.3	HEIGHT	7.85	13		1	1
14	8193	36	Placebo	1	41	6/3/2018	97.4	HEIGHT	12.65	13.5		1	1
14	8196	48	Placebo	0	48	5/22/2019	90.2	HEIGHT	12.8	14		1	1
14	8196	60	Placebo	0	58	2/13/2020	96.6	HEIGHT	14.95454545	14.4		1	1
14	8243	60	Placebo	1	21	2/16/2020	67.9	LENGTH	5.272727273	9.4		1	1
14	8245	48	Placebo	1	11	5/22/2019	68.8	LENGTH	7.45	13		1	1
14	8245	60	Placebo	1	19	2/13/2020	72.7	HEIGHT	8.8	13		0	1
14	8248	36	Placebo	0	35	6/3/2018	91	HEIGHT	12.7	14.5		1	1
14	8269	60	Placebo	1	43	2/13/2020	95.1	HEIGHT	12.1	12.5		1	1
14	8276	60	Placebo	1	43	2/13/2020	95.2	HEIGHT	12.9	14.3		1	1
14	8359	60	Placebo	0	33	2/16/2020	82.8	HEIGHT	11.45454545	12.5		1	1
14	8401	36	Placebo	0	21	6/3/2018	77.9	HEIGHT	9.65	14.5		1	1
14	8426	60	Placebo	1	19	2/13/2020	84.9	LENGTH	11.8	13.9		1	1
14	8429	36	Placebo	1	15	6/3/2018	77	LENGTH	9.35	14.5		1	1

14	8429	48	Placebo	1	25	5/22/2019	84.5	LENGTH	11.45	14		1	1
14	8431	60	Placebo	0	21	2/13/2020	78.9	HEIGHT	9.4	12.7		1	1
14	8441	48	Placebo	1	7	5/22/2019	70.6	LENGTH	7.35	13	54	1	1
14	8448	48	Placebo	0	48	5/22/2019	97.2	HEIGHT	14.1	15		1	1
14	8458	60	Placebo	0	29	2/16/2020	86.6	HEIGHT	12.59090909	15		1	1
14	8474	48	Placebo	0	32	5/22/2019	96.8	HEIGHT	15	15.5		1	1
14	8483	48	Placebo	1	1	5/22/2019	58.2	LENGTH	4.8	11.5		1	1
14	8483	60	Placebo	1	10	2/13/2020	68.7	LENGTH	7	12		0	1
14	8510	48	Placebo	0	10	5/22/2019	70.5	LENGTH	8.05	13		1	1
14	8511	48	Placebo	1	15	5/22/2019	75.9	LENGTH	8.5	13.5		1	1
14	8512	60	Placebo	1	9	2/16/2020	70.2	HEIGHT	7.409090909	13		1	1
14	8558	60	Placebo	1	5	2/13/2020	62.8	LENGTH	7	13.9		1	1
14	8576	48	Placebo	1	10	5/22/2019	74.2	HEIGHT	7.75	12.5		1	1
14	8576	60	Placebo	1	14	2/13/2020	81.1	LENGTH	10.7	13.7		0	1
14	8606	60	Placebo	1	10	2/16/2020	67.2	LENGTH	6.681818182	11.6		1	1
14	8665	36	Placebo	0	34	6/3/2018	89.1	HEIGHT	14.2	16		1	1
14	8676	36	Placebo	0	14	6/3/2018	73.3	LENGTH	8.05	14		1	1
14	8688	36	Placebo	1	36	6/3/2018	84.7	HEIGHT	11.3	14.5		1	1
14	8688	48	Placebo	1	47	5/22/2019	91.6	HEIGHT	12.95	14.5		1	1
14	8690	36	Placebo	0	21	6/3/2018	80.8	LENGTH	9.95	14		1	1
14	8690	48	Placebo	0	31	5/22/2019	86	HEIGHT	12	14.5		1	1
14	8694	48	Placebo	1	28	5/22/2019	83.1	HEIGHT	10.45	14		1	1
14	8763	60	Placebo	0	39	2/13/2020	89.8	HEIGHT	14.9	15		1	1
14	8775	48	Placebo	1	27	5/22/2019	84.2	HEIGHT	10.65	14		1	1
14	8789	36	Placebo	0	10	6/3/2018	68.8	LENGTH	7.7	14		1	1
14	8789	48	Placebo	0	21	5/22/2019	76.7	HEIGHT	9.3	13		1	1
14	8816	36	Placebo	0	56	6/3/2018	89.6	HEIGHT	11	12.5		1	1
14	8819	60	Placebo	1	11	2/16/2020	72.9	HEIGHT	7.954545455	13		1	1
14	8837	48	Placebo	1	21	5/22/2019	75.8	LENGTH	8.5	13.5		1	1
14	8860	48	Placebo	1	18	5/22/2019	75.5	LENGTH	9	12.5		1	1
14	8869	48	Placebo	1	30	5/22/2019	80.4	LENGTH	9.05	12.5		1	1
14	8879	36	Placebo	1	18	6/3/2018	74	HEIGHT	8.6	12.5	60	1	1
14	8879	48	Placebo	1	29	5/22/2019	80.6	HEIGHT	10.05	13	60	1	1
14	8882	36	Placebo	1	41	6/3/2018	94.9	HEIGHT	14.55	16		1	1
14	8897	60	Placebo	1	49	2/13/2020	98.6	HEIGHT	13.8	13.6		1	1
14	8957	36	Placebo	1	29	6/3/2018	87.5	HEIGHT	11.35	14.5		1	1
14	8959	36	Placebo	1	20	6/3/2018	79.1	HEIGHT	9.75	14.5		1	1
14	8959	60	Placebo	1	40	2/16/2020	90.5	HEIGHT	13.90909091	14.9		1	1
14	8969	60	Placebo	0	31	2/13/2020	83.8	HEIGHT	11	12.5		1	1
14	9007	48	Placebo	1	35	5/22/2019	91.8	HEIGHT	13.9	15.5		1	1

14	9007	60	Placebo	1	45	2/13/2020	97.5	HEIGHT	15.3	16		1	1
14	9017	60	Placebo	0	4	2/16/2020	64.1	LENGTH	6.181818182	12.5		1	1
14	9034	48	Placebo	1	56	5/22/2019	99.6	HEIGHT	13.8	13.5		1	1
14	9062	60	Placebo	0	23	2/16/2020	77.2	LENGTH	9.590909091	13.3		1	1
14	9097	36	Placebo	1	4	6/3/2018	59.2	LENGTH	5.65	12.5		1	1
14	9105	60	Placebo	1	43	2/13/2020	91.7	HEIGHT	12.18181818	13		1	1
14	9122	36	Placebo	1	59	6/3/2018	98.9	HEIGHT	13.55	13.5		1	1
14	9123	60	Placebo	0	44	2/13/2020	94.4	HEIGHT	14.59090909	15.5		1	1
14	9128	36	Placebo	0	15	6/3/2018	69.7	HEIGHT	7.45	12		1	1
14	9128	48	Placebo	0	26	5/22/2019	76.9	HEIGHT	9.5	13		1	1
14	9135	60	Placebo	1	45	2/16/2020	100.4	HEIGHT	16.90909091	16.9		1	1
14	9153	36	Placebo	1	44	6/3/2018	97.2	HEIGHT	13.75	15		1	1
14	9161	36	Placebo	0	21	6/3/2018	84.3	HEIGHT	10.85	13.5		1	1
14	9161	60	Placebo	0	41	2/13/2020	98.2	HEIGHT	15.1	15		1	1
14	9178	36	Placebo	1	46	6/3/2018	98	HEIGHT	13.8	14		1	1
14	9178	48	Placebo	1	57	5/22/2019	104.1	HEIGHT	14.9	13.5		1	1
14	9190	48	Placebo	1	9	5/22/2019	67.8	LENGTH	8.4	14		1	1
14	9190	60	Placebo	1	18	2/13/2020	76.2	LENGTH	10.5	14		1	1
14	9244	60	Placebo	0	14	2/16/2020	72.4	LENGTH	8.045454545	12.2		1	1
14	9281	48	Placebo	0	22	5/22/2019	77.4	LENGTH	8.4	13		1	1
14	9289	36	Placebo	0	24	6/3/2018	71.9	LENGTH	8.25	12		1	1
14	9289	60	Placebo	0	44	2/16/2020	86.7	HEIGHT	11.95454545	14		1	1
14	9296	36	Placebo	0	29	6/3/2018	89.1	HEIGHT	14.15	17		1	1
14	9297	36	Placebo	0	8	6/3/2018	70.4	LENGTH	8	12.5		1	1
14	9297	48	Placebo	0	19	5/22/2019	80.7	LENGTH	10	13.5		0	1
14	9297	60	Placebo	0	28	2/13/2020	87.8	HEIGHT	12.1	13.5		1	1
14	9320	48	Placebo	1	39	5/22/2019	92.6	HEIGHT	13.45	14		1	1
14	9339	48	Placebo	0	33	5/22/2019	90.3	HEIGHT	13.5	16		1	1
14	9372	36	Placebo	0	53	6/3/2018	92.8	HEIGHT	12.65	13		1	1
14	9386	36	Placebo	1	13	6/3/2018	72.6	LENGTH	8.35	14.5		1	1
14	9404	36	Placebo	1	10	6/3/2018	66.2	LENGTH	6.3	12		1	1
14	9404	60	Placebo	1	30	2/13/2020	84.2	LENGTH	10.4	13		0	1
14	9410	60	Placebo	1	17	2/13/2020	81.1	LENGTH	9.85	14.3		1	1
14	9422	36	Placebo	0	21	6/3/2018	76.6	LENGTH	8.65	12		1	1
14	9423	48	Placebo	1	52	5/22/2019	100.6	HEIGHT	15.45	15.5		1	1
14	9501	36	Placebo	1	13	6/3/2018	75.7	LENGTH	8.8	14	54	1	1
14	9501	48	Placebo	1	24	5/22/2019	86.9	LENGTH	11.5	15	54	1	1
14	9506	48	Placebo	0	18	5/22/2019	80.9	LENGTH	10.15	14		1	1
14	9515	60	Placebo	0	12	2/16/2020	84.9	HEIGHT	10.81818182	13.2		1	1
14	9553	60	Placebo	0	20	2/13/2020	79.9	LENGTH	9.3	13.2		1	1

15	3948	24	Placebo	0	47	6/12/2017	88.4	HEIGHT	10.1	12		1	0
15	3949	24	Placebo	0	32	6/12/2017	83.2	HEIGHT	9.65	12.5		1	0
15	3949	36	Placebo	0	44	5/27/2018	92	HEIGHT	11.9	14		1	0
15	3949	48	Placebo	0	55	5/24/2019	100.6	LENGTH	14.18181818	14		1	0
15	3949	60	Placebo	0	56	3/17/2020	106.4	HEIGHT	16.35	15		1	0
15	3950	0	Placebo	1	24	4/11/2015	89.7	HEIGHT	11	12.5		1	0
15	3951	0	Placebo	1	24	6/15/2015	74.1	LENGTH	9.05	13.5		1	0
15	3951	12	Placebo	1	35	7/8/2016	82.5	HEIGHT	11.4	15		1	0
15	3951	24	Placebo	1	47	6/12/2017	88.3	HEIGHT	12.9	15.5		1	0
15	3951	48	Placebo	1	70	5/24/2019	102.2	HEIGHT	17.18181818	15.5		0	0
15	3951	60	Placebo	1	79	2/25/2020	107.1	HEIGHT	18.13636364	15.2		0	0
15	3952	24	Placebo	0	6	6/12/2017	71.2	HEIGHT	8	13.5		1	0
15	3952	36	Placebo	0	15	5/27/2018	76.6	HEIGHT	8.7	12.5		1	0
15	3952	48	Placebo	0	26	5/24/2019	77.8	LENGTH	10	12.5		1	0
15	3952	60	Placebo	0	35	2/25/2020	86.3	HEIGHT	11.36363636	13.2		1	0
15	3953	0	Placebo	1	48	4/11/2015	88.5	HEIGHT	12.75	16		1	0
15	3953	12	Placebo	1	57	7/12/2016	97	HEIGHT	15.6	17		1	0
15	3953	24	Placebo	1	71	6/12/2017	103.3	HEIGHT	15.25	15		0	0
15	3953	48	Placebo	1	94	6/11/2019	114.6	HEIGHT	19.81818182	17.2		0	0
15	3953	60	Placebo	1	103	3/17/2020	119.7	HEIGHT	19.6	15		0	0
15	3955	0	Placebo	0	48	4/11/2015	99.9	HEIGHT	11.9	14		1	0
15	3955	12	Placebo	0	59	7/12/2016	106.9	HEIGHT	13.8	13.5		1	0
15	3955	24	Placebo	0	75	6/12/2017	113.2	HEIGHT	13.85	12.5		0	0
15	3957	12	Placebo	0	9	7/12/2016	73.6	LENGTH	7.65	13.5		0	0
15	3957	24	Placebo	0	23	6/12/2017	82.5	HEIGHT	8.5	12		1	0
15	3957	36	Placebo	0	35	5/27/2018	89.3	HEIGHT	11.5	13.5		1	0
15	3957	48	Placebo	0	46	6/11/2019	98.5	HEIGHT	13.63636364	15		1	0
15	3957	60	Placebo	0	55	2/25/2020	101.9	HEIGHT	14.36363636	13.8		1	0
15	3958	12	Placebo	1	12	7/12/2016	68.5	LENGTH	6.35	11	48	1	0
15	3958	24	Placebo	1	26	6/12/2017	74.5	HEIGHT	7.25	10.5	48	1	0
15	3958	36	Placebo	1	38	5/27/2018	82.6	HEIGHT	8.8	11.5	48	1	0
15	3960	0	Placebo	0	36	6/15/2015	83.9	HEIGHT	10.55	14		1	0
15	3960	24	Placebo	0	56	6/12/2017	98.5	HEIGHT	14.2	14.5		1	0
15	3961	0	Placebo	1	4	4/11/2015	63.2	LENGTH	6.15	13		1	0
15	3962	0	Placebo	1	36	4/11/2015	83.3	HEIGHT	10.45	15		1	0
15	3962	24	Placebo	1	59	6/12/2017	99.1	HEIGHT	13.4	14		1	0
15	3963	0	Placebo	1	48	4/11/2015	95.3	HEIGHT	14.15	14.5		1	0
15	3963	12	Placebo	1	54	7/8/2016	100.2	HEIGHT	14.95	15		1	0
15	3963	24	Placebo	1	68	6/12/2017	106.9	HEIGHT	16.2	12.5		0	0
15	3963	60	Placebo	1	100	3/17/2020	120.2	HEIGHT	20.05	15		0	0

15	3964	12	Placebo	0	57	7/8/2016	94	HEIGHT	11.5	13.5		1	0
15	3965	0	Placebo	1	24	6/15/2015	80.3	HEIGHT	8.25	12		1	0
15	3965	12	Placebo	1	35	7/8/2016	85.2	HEIGHT	10.1	13.5		1	0
15	3965	24	Placebo	1	47	6/12/2017	92.2	HEIGHT	11.4	13.5		1	0
15	3965	36	Placebo	1	59	5/27/2018	98.2	HEIGHT	12.3	13		1	0
15	3965	48	Placebo	1	70	5/24/2019	102.1	HEIGHT	13.15	13		0	0
15	3970	0	Placebo	0	12	4/11/2015	77.8	LENGTH	10.2	16		1	0
15	3970	12	Placebo	0	33	7/8/2016	85.9	HEIGHT	12.2	15.5		1	0
15	3970	24	Placebo	0	44	6/12/2017	93.1	HEIGHT	14.85	16.5		1	0
15	3970	36	Placebo	0	56	5/27/2018	99.2	HEIGHT	16.65	16.5		1	0
15	3970	48	Placebo	0	67	5/24/2019	105.7	HEIGHT	18.05	16		0	0
15	3970	60	Placebo	0	76	2/25/2020	111	HEIGHT	19.36363636	15.9		0	0
15	3971	0	Placebo	1	48	4/11/2015	93.4	HEIGHT	12.75	14.5		1	0
15	3971	12	Placebo	1	66	7/8/2016	101.7	HEIGHT	14.6	15		0	0
15	3971	24	Placebo	1	63	6/12/2017	108.8	HEIGHT	15.5	13.5		1	0
15	3972	12	Placebo	1	6	7/8/2016	67.5	LENGTH	6.6	12.5	30	1	0
15	3972	24	Placebo	1	17	6/12/2017	76.1	HEIGHT	7.85	11.5	30	1	0
15	3973	0	Placebo	0	48	4/11/2015	95.3	HEIGHT	13.05	14.5		1	0
15	3973	12	Placebo	0	57	7/12/2016	101.6	HEIGHT	13.5	14.5		1	0
15	3973	24	Placebo	0	71	6/12/2017	109.2	HEIGHT	15.15	14		0	0
15	3973	36	Placebo	0	83	5/27/2018	113.4	HEIGHT	16.8	14.5		0	0
15	3973	48	Placebo	0	94	6/11/2019	117.9	HEIGHT	18.81818182	15		0	0
15	3973	60	Placebo	0	103	3/17/2020	121.5	HEIGHT	20.75	14.5		0	0
15	3974	0	Placebo	0	36	4/11/2015	82.9	HEIGHT	9.5	13.5		1	0
15	3974	12	Placebo	0	39	7/12/2016	88	HEIGHT	11.25	14.5		1	0
15	3974	24	Placebo	0	53	6/12/2017	94.6	HEIGHT	11.9	14		1	0
15	3974	48	Placebo	0	76	5/24/2019	102.3	LENGTH	13.5	13		0	0
15	3975	0	Placebo	0	8	4/11/2015	67	LENGTH	7.45	15.5		1	0
15	3975	12	Placebo	0	15	7/12/2016	74.5	LENGTH	9.1	14.5		1	0
15	3975	36	Placebo	0	41	5/27/2018	89.6	HEIGHT	12.75	16		1	0
15	3975	48	Placebo	0	52	6/11/2019	96.1	HEIGHT	13.77272727	16		1	0
15	3975	60	Placebo	0	61	2/25/2020	100.5	HEIGHT	16.09090909	15.1		0	0
15	3977	0	Placebo	0	36	4/11/2015	75.2	HEIGHT	8.75	15		1	0
15	3977	12	Placebo	0	45	7/12/2016	80.5	HEIGHT	12.7	16		1	0
15	3977	24	Placebo	0	59	6/12/2017	89.1	HEIGHT	13	15		1	0
15	3977	48	Placebo	0	82	6/11/2019	99.4	HEIGHT	15.72727273	15.6		0	0
15	3977	60	Placebo	0	91	3/17/2020	104.7	HEIGHT	16.45	14.4		0	0
15	3980	0	Placebo	0	54	4/11/2015	94.5	HEIGHT	14.4	15		1	0
15	3980	12	Placebo	0	63	7/8/2016	100.6	HEIGHT	15.05	14.5		0	0
15	3980	24	Placebo	0	77	6/12/2017	107.1	HEIGHT	17.1	14.5		0	0

15	3980	36	Placebo	0	89	5/27/2018	111.9	HEIGHT	18.75	15		0	0
15	3980	48	Placebo	0	100	5/24/2019	107.2	HEIGHT	17.81818182	15		0	0
15	3980	60	Placebo	0	109	2/25/2020	121.8	HEIGHT	22.27272727	15.2		0	0
15	3981	12	Placebo	0	7	7/8/2016	70	LENGTH	7.5	15		1	0
15	3981	24	Placebo	0	17	6/12/2017	76.5	LENGTH	7.85	12.5		1	0
15	3981	60	Placebo	0	49	2/25/2020	88.3	HEIGHT	12.31818182	15		1	0
15	3982	24	Placebo	0	7	6/12/2017	67.6	LENGTH	6.25	12		1	0
15	3982	48	Placebo	0	26	6/11/2019	85.2	HEIGHT	10.36363636	14		1	0
15	3982	60	Placebo	0	35	2/25/2020	92.1	HEIGHT	11.45454545	13		1	0
15	3984	0	Placebo	1	24	4/11/2015	80.1	HEIGHT	10.25	14		1	0
15	3984	12	Placebo	1	30	7/8/2016	88.7	HEIGHT	12.05	15		0	0
15	3984	24	Placebo	1	44	6/12/2017	96.8	HEIGHT	13.45	14		1	0
15	3984	48	Placebo	1	67	5/24/2019	107.6	HEIGHT	15.72727273	13.5		0	0
15	3984	60	Placebo	1	76	3/17/2020	111.3	HEIGHT	17.15	14		0	0
15	3985	0	Placebo	0	3	4/11/2015	61.2	LENGTH	6.15	13		1	0
15	3985	12	Placebo	0	18	7/8/2016	73.3	LENGTH	8.75	14.5		1	0
15	3985	24	Placebo	0	29	6/12/2017	81.8	HEIGHT	10.8	14		1	0
15	3985	48	Placebo	0	52	5/24/2019	88.7	LENGTH	14.22727273	14.5		1	0
15	3986	12	Placebo	0	36	7/8/2016	90.1	HEIGHT	13.45	16		1	0
15	3986	24	Placebo	0	47	6/12/2017	94.8	HEIGHT	14	15		1	0
15	3986	48	Placebo	0	70	5/24/2019	99.4	LENGTH	17	16		0	0
15	3987	12	Placebo	1	45	7/12/2016	92.5	HEIGHT	12.15	14		1	0
15	3988	0	Placebo	0	24	4/11/2015	86.1	HEIGHT	10.5	14.5	30	1	0
15	3988	12	Placebo	0	35	7/27/2016	95.3	HEIGHT		14	30	1	0
15	3988	24	Placebo	0	46	6/12/2017	102.6	HEIGHT	12.65	13	30	1	0
15	3989	0	Placebo	0	44	4/11/2015	91.2	HEIGHT	15.15	16.5		1	0
15	3989	12	Placebo	0	45	7/8/2016	97.3	HEIGHT	16.6	17		1	0
15	3989	24	Placebo	0	56	6/12/2017	103.4	HEIGHT	17.25	16		1	0
15	3989	36	Placebo	0	68	5/27/2018	107.3	HEIGHT	18.9	16		0	0
15	3989	48	Placebo	0	79	5/24/2019	111.2	HEIGHT	20.85	17		0	0
15	3989	60	Placebo	0	88	2/25/2020	115.5	HEIGHT	22.77272727	16.5		0	0
15	3990	0	Placebo	1	36	6/15/2015	86	HEIGHT	12.5	16	12	1	0
15	3992	12	Placebo	1	54	7/8/2016	115.9	HEIGHT	21.05	17.5		1	0
15	3992	60	Placebo	1	100	2/25/2020	137.1	HEIGHT	32.22727273	19.3		0	0
15	3993	12	Placebo	0	11	7/27/2016	72.9	LENGTH		13.5		1	0
15	3993	24	Placebo	0	16	6/12/2017	83.7	HEIGHT	9.5	13.5		1	0
15	3993	48	Placebo	0	39	6/11/2019	101.3	HEIGHT	14.63636364	16.5		1	0
15	3995	24	Placebo	1	12	6/12/2017	65.8	LENGTH	6.3	11.5		1	0
15	3995	48	Placebo	1	34	6/11/2019	81.6	HEIGHT	8.681818182	12.5		1	0
15	3996	0	Placebo	0	54	4/11/2015	110.3	HEIGHT	16.65	15		1	0

15	3996	48	Placebo	0	100	6/11/2019	129.1	HEIGHT	24.18181818	17		0	0
15	3997	12	Placebo	0	2	7/8/2016	62.8	LENGTH	5.25	11.5	18	1	0
15	3999	0	Placebo	0	12	4/11/2015	67.7	LENGTH	7.35	13.5	6	1	0
15	4001	0	Placebo	1	36	4/11/2015	86.7	HEIGHT	10.5	13		1	0
15	4001	12	Placebo	1	45	7/12/2016	96.3	HEIGHT	13.25	15		1	0
15	4001	36	Placebo	1	71	5/27/2018	110.2	HEIGHT	15.55	13.5		0	0
15	4001	48	Placebo	1	82	6/11/2019	117.2	HEIGHT	17.95454545	15		0	0
15	4001	60	Placebo	1	91	2/25/2020	121.3	HEIGHT	18.95454545	14.2		0	0
15	4002	12	Placebo	0	26	7/8/2016	84.1	HEIGHT	10.2	14		1	0
15	4002	24	Placebo	0	40	6/12/2017	92.3	HEIGHT	11.75	13.5		1	0
15	4002	36	Placebo	0	52	5/27/2018	96.8	HEIGHT	12.45454545	13		1	0
15	4002	60	Placebo	0	72	2/25/2020	105.9	HEIGHT	14.68181818	12.2		0	0
15	4010	0	Placebo	0	48	4/11/2015	102.8	HEIGHT	14.2	13.5		1	0
15	4010	36	Placebo	0	113	5/27/2018	118.2	HEIGHT	19	15		0	0
15	4010	48	Placebo	0	124	5/24/2019	122.3	HEIGHT	21	15.5		0	0
15	4010	60	Placebo	0	133	2/25/2020	126.2	HEIGHT	23.5	16.2		0	0
15	4011	12	Placebo	0	7	7/8/2016	65.4	LENGTH	5.5	11	30	0	0
15	4011	24	Placebo	0	17	6/12/2017	72.7	LENGTH	7.45	11	30	1	0
15	4012	0	Placebo	0	48	4/11/2015	82.8	LENGTH	11.2	15.5		1	0
15	4012	12	Placebo	0	47	7/8/2016	92.5	HEIGHT	13.55	16.5		1	0
15	4015	0	Placebo	1	24	4/11/2015	82.3	HEIGHT	10.8	14.5		1	0
15	4015	12	Placebo	1	42	7/12/2016	93.3	HEIGHT	13.6	16		1	0
15	4015	24	Placebo	1	56	6/12/2017	101.5	HEIGHT	14.6	15		1	0
15	4015	36	Placebo	1	68	5/27/2018	107.6	HEIGHT	16.5	16		0	0
15	4015	48	Placebo	1	79	6/11/2019	113.9	LENGTH	19	17.5		0	0
15	4015	60	Placebo	1	88	2/25/2020	119.1	HEIGHT	20.5	15.6		0	0
15	4017	12	Placebo	1	1	7/8/2016	58.1	LENGTH	4.55	12		1	0
15	4018	24	Placebo	1	6	6/12/2017	63	LENGTH	7.1	15		1	0
15	4018	36	Placebo	1	15	5/27/2018	73.6	LENGTH	9.15	15.5		1	0
15	4018	48	Placebo	1	26	5/24/2019	82.6	LENGTH	11.1	15.5		1	0
15	4018	60	Placebo	1	35	2/25/2020	86.4	HEIGHT	12.86363636	16		1	0
15	4019	0	Placebo	1	24	4/11/2015	82.8	HEIGHT	11.7	15		1	0
15	4019	12	Placebo	1	45	7/8/2016	89.2	HEIGHT	13.1	16		1	0
15	4019	24	Placebo	1	56	6/12/2017	96.4	HEIGHT	15.05	15.5		1	0
15	4019	36	Placebo	1	68	5/27/2018	101.2	HEIGHT	15.6	16		0	0
15	4020	0	Placebo	0	36	4/11/2015	83.6	HEIGHT	12.25	16.5		1	0
15	4020	48	Placebo	0	98	5/24/2019	106	LENGTH	17.68181818	15.5		0	0
15	4023	0	Placebo	0	12	6/15/2015	74.2	LENGTH	7.15	11.5		1	0
15	4023	24	Placebo	0	44	6/12/2017	87.3	HEIGHT	11.3	14		1	0
15	4023	36	Placebo	0	56	5/27/2018	95.8	HEIGHT	13.45	15		1	0

15	4023	48	Placebo	0	67	5/24/2019	100	LENGTH	14.09090909	14.5		0	0
15	4023	60	Placebo	0	76	2/25/2020	106	HEIGHT	15.59090909	14.2		0	0
15	4027	12	Placebo	0	2	7/8/2016	66.9	LENGTH	6.2	14		1	0
15	4027	24	Placebo	0	12	6/12/2017	77.8	HEIGHT	8.8	13.5		1	0
15	4027	36	Placebo	0	24	5/27/2018	81.6	HEIGHT	9.727272727	13		1	0
15	4027	60	Placebo	0	44	2/25/2020	92	HEIGHT	13.77272727	15		1	0
15	4028	12	Placebo	0	53	7/12/2016	104.1	HEIGHT	13.7	13		1	0
15	4029	12	Placebo	1	8	7/8/2016	70.9	HEIGHT	7.5	13	54	0	0
15	4029	24	Placebo	1	17	6/12/2017	82.4	HEIGHT	10	13	54	1	0
15	4029	36	Placebo	1	29	5/27/2018	88.6	LENGTH	12.45	15	54	1	0
15	4029	48	Placebo	1	40	5/24/2019	95.7	HEIGHT	13.18181818	14	54	1	0
15	4033	0	Placebo	0	48	4/11/2015	85	HEIGHT	11	15.5		1	0
15	4034	0	Placebo	0	1	4/11/2015	61.1	LENGTH	6.1	12.5		1	0
15	4034	12	Placebo	0	12	7/8/2016	74.1	LENGTH	8.05	13.5		1	0
15	4036	12	Placebo	0	7	7/8/2016	74.4	LENGTH	8.55	14.5		1	0
15	4036	24	Placebo	0	17	6/12/2017	79.2	HEIGHT	8.3	11		1	0
15	4039	0	Placebo	0	36	4/11/2015	93.8	HEIGHT	14.2	16		1	0
15	4039	24	Placebo	0	59	6/12/2017	107.5	HEIGHT	16.6	14		1	0
15	4039	48	Placebo	0	82	5/24/2019	116.4	LENGTH	18.27272727	14		0	0
15	4039	60	Placebo	0	91	2/25/2020	121.9	HEIGHT	21.13636364	14.6		0	0
15	4041	0	Placebo	0	24	4/11/2015	78.7	HEIGHT	8.75	13.5		1	0
15	4041	24	Placebo	0	47	6/12/2017	91.9	HEIGHT	11.35	13.5		1	0
15	4041	36	Placebo	0	59	5/27/2018	98	HEIGHT	13.05	13.5		1	0
15	4041	48	Placebo	0	70	6/11/2019	117.6	HEIGHT	14.95454545	14		0	0
15	4041	60	Placebo	0	79	3/17/2020	111.1	HEIGHT	16.7	13.5		0	0
15	4043	0	Placebo	1	36	4/11/2015	86.4	HEIGHT	10.35	14.5		1	0
15	4043	60	Placebo	1	91	3/17/2020	112.7	HEIGHT	17.8	14.4		0	0
15	4044	0	Placebo	1	12	4/11/2015	73.2	HEIGHT	7.85	11.5		0	0
15	4044	12	Placebo	1	23	7/8/2016	80.2	HEIGHT	8.9	13		1	0
15	4044	24	Placebo	1	35	6/12/2017	84.7	HEIGHT	10.1	12.5		1	0
15	4044	36	Placebo	1	47	5/27/2018	92.3	HEIGHT	11.5	13.5		1	0
15	4044	48	Placebo	1	58	5/24/2019	96.1	LENGTH	13.18181818	13.5		1	0
15	4044	60	Placebo	1	56	2/25/2020	100.4	HEIGHT	14.27272727	13.4		1	0
15	4046	0	Placebo	0	54	6/15/2015	96.2	HEIGHT	12	14		1	0
15	4046	12	Placebo	0	63	7/12/2016	101.8	HEIGHT	13.1	13.5		0	0
15	4046	24	Placebo	0	77	6/12/2017	108.3	HEIGHT	13.9	13		0	0
15	4048	0	Placebo	0	48	4/11/2015	99.7	HEIGHT	15.75	15.5		1	0
15	4048	12	Placebo	0	54	7/8/2016	107.3	HEIGHT	17.35	16.5		1	0
15	4048	24	Placebo	0	68	6/12/2017	113.2	HEIGHT	18.55	15.5		0	0
15	4048	36	Placebo	0	80	5/27/2018	118.2	HEIGHT	20.40909091	16		0	0

15	4048	48	Placebo	0	91	5/24/2019	123.1	HEIGHT	21.8	15.5	0	0
15	4048	60	Placebo	0	100	2/25/2020	126.3	HEIGHT	24.45454545	16.3	0	0
15	4051	12	Placebo	0	44	7/8/2016	100.7	HEIGHT	12.35	13	1	0
15	4051	24	Placebo	0	58	6/12/2017	107.4	HEIGHT	13	12.5	1	0
15	4051	60	Placebo	0	90	2/25/2020	122.2	HEIGHT	17.5	13	0	0
15	4054	12	Placebo	0	33	7/8/2016	84.9	HEIGHT	10.35	13.5	1	0
15	4055	0	Placebo	0	36	4/11/2015	87.2	HEIGHT	11.5	15.5	1	0
15	4055	12	Placebo	0	47	7/8/2016	95.8	HEIGHT	12.5	14.5	1	0
15	4055	24	Placebo	0	59	6/12/2017	102.8	HEIGHT	14.35	14.5	1	0
15	4055	36	Placebo	0	71	5/27/2018	110.4	HEIGHT	16.35	14.5	0	0
15	4055	48	Placebo	0	82	5/24/2019	114	LENGTH	17.86363636	14.5	0	0
15	4055	60	Placebo	0	91	2/25/2020	117.9	HEIGHT	19.22727273	14.2	0	0
15	4056	0	Placebo	0	8	4/11/2015	65	LENGTH	6.85	13	1	0
15	4057	0	Placebo	0	36	4/11/2015	77.1	HEIGHT	8.4	12.5	1	0
15	4057	48	Placebo	0	82	5/24/2019	103.6	HEIGHT	14.54545455	13	0	0
15	4058	0	Placebo	0	24	4/11/2015	82.1	HEIGHT	11.45	15.5	1	0
15	4058	12	Placebo	0	33	7/8/2016	87.9	HEIGHT	12.85	15	1	0
15	4058	24	Placebo	0	47	6/12/2017	95.6	HEIGHT	14.8	14.5	1	0
15	4058	36	Placebo	0	59	5/27/2018	101.7	HEIGHT	16.05	15.5	1	0
15	4058	48	Placebo	0	70	5/24/2019	114.5	LENGTH	20.81818182	15.5	0	0
15	4058	60	Placebo	0	79	2/25/2020	112.5	HEIGHT	18.86363636	14.9	0	0
15	4059	12	Placebo	1	6	7/12/2016	61.3	LENGTH	4.85	11	1	0
15	4059	24	Placebo	1	17	6/12/2017	66.7	LENGTH	5.55	10.5	1	0
15	4059	36	Placebo	1	29	5/27/2018	73	LENGTH	7.2	11.5	1	0
15	4059	60	Placebo	1	49	3/17/2020	87.4	HEIGHT	12.95	14	1	0
15	4060	0	Placebo	1	36	4/11/2015	79.3	HEIGHT	7.65	13	1	0
15	4060	12	Placebo	1	45	7/12/2016	86.2	HEIGHT	10.25	13.5	1	0
15	4060	24	Placebo	1	53	6/12/2017	92	HEIGHT	11.5	13.5	1	0
15	4060	36	Placebo	1	65	5/27/2018	99.7	HEIGHT	12.95	13.5	0	0
15	4060	48	Placebo	1	76	6/11/2019	106.5	HEIGHT	15.81818182	15.5	0	0
15	4060	60	Placebo	1	85	2/25/2020	109.1	HEIGHT	16.86363636	15	0	0
15	4062	24	Placebo	1	11	6/12/2017	66	LENGTH	6.4	12	1	0
15	4062	48	Placebo	1	34	5/24/2019	81.6	HEIGHT	10.5	14	1	0
15	4062	60	Placebo	1	43	2/25/2020	86.6	HEIGHT	11.31818182	13.5	1	0
15	4063	0	Placebo	1	54	4/11/2015	101.5	HEIGHT	14.45	14	0	0
15	4063	12	Placebo	1	54	7/8/2016	108.1	HEIGHT	15.95	13.5	1	0
15	4063	24	Placebo	1	75	6/12/2017	114.9	HEIGHT	17.15	14	0	0
15	4063	36	Placebo	1	87	5/27/2018	121.2	HEIGHT	19.3	14.5	0	0
15	4063	48	Placebo	1	98	5/24/2019	124.5	HEIGHT	21.72727273	15	0	0
15	4063	60	Placebo	1	107	2/25/2020	128.4	HEIGHT	23.63636364	15.2	0	0

15	8020	60	Placebo	0	50	3/17/2020	86.2	HEIGHT	11.25	13.2		1	0
15	8067	60	Placebo	1	8	2/25/2020	68.7	LENGTH	8.545454545	14.3		1	0
15	8170	36	Placebo	0	15	5/27/2018	69.3	LENGTH	6.65	11.5		1	0
15	8170	48	Placebo	0	26	5/24/2019	72.9	LENGTH	7.863636364	12		1	0
15	8170	60	Placebo	0	35	3/17/2020	83.1	HEIGHT	9.85	12.9		1	0
15	8209	60	Placebo	0	7	3/17/2020	72	LENGTH	9.2	14.5		1	0
15	8286	36	Placebo	1	7	5/27/2018	67.6	LENGTH	6.85	12		1	0
15	8286	48	Placebo	1	18	5/24/2019	75.7	LENGTH	7.045454545	11.5		1	0
15	8286	60	Placebo	1	27	3/17/2020	78.5	LENGTH	7.65	11.4		1	0
15	8304	60	Placebo	0	26	2/25/2020	79.5	HEIGHT	9.227272727	12		1	0
15	8377	60	Placebo	1	14	2/25/2020	73.1	LENGTH	7.954545455	11.5		1	0
15	8383	60	Placebo	0	56	2/25/2020	97.2	LENGTH	14.68181818	14.6		1	0
15	8519	60	Placebo	1	50	3/17/2020	91.3	HEIGHT	12.45	13.4		1	0
15	8561	60	Placebo	1	12	3/17/2020	67.1	LENGTH	6	11.5		1	0
15	8585	60	Placebo	1	6	3/17/2020	67.9	LENGTH	7.45	12.7		1	0
15	8594	60	Placebo	1	8	3/17/2020	57.1	LENGTH	5.1	11.5		1	0
15	8605	48	Placebo	0	2	5/24/2019	56.1	LENGTH	5.5	12		1	0
15	8605	60	Placebo	0	11	2/25/2020	69.1	LENGTH	6.909090909	11.3		1	0
15	8669	60	Placebo	0	14	2/25/2020	75.7	HEIGHT	7.954545455	11.7		1	0
15	8692	36	Placebo	0	9	5/27/2018	65.6	LENGTH	5.75	12		1	0
15	8692	48	Placebo	0	16	5/24/2019	73.2	LENGTH	8.590909091	13.5		1	0
15	8692	60	Placebo	0	25	2/25/2020	80.8	HEIGHT	10.77272727	13.9		1	0
15	8696	48	Placebo	1	12	5/24/2019	72.2	LENGTH	8.6	14		1	0
15	8696	60	Placebo	1	20	2/25/2020	78.7	LENGTH	9.954545455	13		1	0
15	8705	48	Placebo	0	15	5/24/2019	64.1	LENGTH	4.2	8	54	1	0
15	8716	60	Placebo	1	56	2/25/2020	93.4	HEIGHT	12.5	13.5		1	0
15	8747	36	Placebo	1	15	5/27/2018	73.7	LENGTH	7.6	12.5		1	0
15	8747	48	Placebo	1	26	5/24/2019	82.5	LENGTH	8.863636364	12.5		1	0
15	8747	60	Placebo	1	35	2/25/2020	88.5	LENGTH	10.81818182	12		1	0
15	8753	60	Placebo	1	20	2/25/2020	72	LENGTH	7.5	12.5		1	0
15	8835	60	Placebo	0	56	2/25/2020	88.7	HEIGHT	11.86363636	13.2		1	0
15	8846	60	Placebo	1	18	2/25/2020	82.2	HEIGHT	9.5	12		1	0
15	8872	48	Placebo	1	12	5/24/2019	72.1	LENGTH	8.181818182	13.5		1	0
15	8872	60	Placebo	1	21	2/25/2020	77.8	HEIGHT	9.318181818	13.5		1	0
15	8919	60	Placebo	1	10	3/17/2020	68	LENGTH	7.05	12.5		1	0
15	9072	36	Placebo	0	15	5/27/2018	74.1	LENGTH	8.85	14		1	0
15	9072	48	Placebo	0	26	6/11/2019	81.6	HEIGHT	9.772727273	14.3		1	0
15	9072	60	Placebo	0	35	3/17/2020	86.4	HEIGHT	12.5	15.2		1	0
15	9163	48	Placebo	0	10	5/24/2019	83.7	LENGTH	12.55	16		1	0
15	9177	48	Placebo	0	5	5/24/2019	57.6	LENGTH	4.272727273	10		1	0

15	9177	60	Placebo	0	12	3/17/2020	67.9	LENGTH	6.75	11		1	0
15	9193	48	Placebo	0	9	6/11/2019	63.8	LENGTH	5.681818182	12		1	0
15	9193	60	Placebo	0	14	3/17/2020	67.8	HEIGHT	6.85	11.5		1	0
15	9278	60	Placebo	1	4	2/25/2020	56	LENGTH	4.363636364	10.2		1	0
15	9326	60	Placebo	0	26	3/17/2020	88.7	HEIGHT	13.3	15		1	0
15	9476	60	Placebo	0	11	2/25/2020	67.4	LENGTH	6.318181818	11.5		1	0
15	9576	60	Placebo	1	19	2/25/2020	70.6	LENGTH	8.181818182	12		1	0
16	4078	0	Azithromycin	0	24	3/26/2015	86.3	HEIGHT	10.8	14		1	0
16	4078	12	Azithromycin	0	39	6/11/2016	97.3	HEIGHT	14.15	15		1	0
16	4078	24	Azithromycin	0	50	3/22/2017	103.3	HEIGHT	14.7	14.5		0	0
16	4078	36	Azithromycin	0	65	5/18/2018	113.5	HEIGHT	17.5	14.5		0	0
16	4078	48	Azithromycin	0	76	4/18/2019	118.3	HEIGHT	19.35	15		0	0
16	4078	60	Azithromycin	0	85	2/10/2020	122.3	HEIGHT	21.15	15.5		0	0
16	4079	0	Azithromycin	0	36	3/26/2015	87.7	HEIGHT	13.45	16		1	0
16	4080	12	Azithromycin	1	12	6/11/2016	75.4	HEIGHT	8.85	14.5		1	0
16	4080	24	Azithromycin	1	23	3/22/2017	84.7	HEIGHT	10.8	14.5		1	0
16	4080	36	Azithromycin	1	37	5/18/2018	94.8	HEIGHT	14	15.5		1	0
16	4080	48	Azithromycin	1	48	4/18/2019	102.6	HEIGHT	15.65	15.5		0	0
16	4080	60	Azithromycin	1	58	2/10/2020	108.4	HEIGHT	18.6	16		1	0
16	4082	12	Azithromycin	0	12	6/11/2016	74.9	HEIGHT	7.8	12	54	1	0
16	4082	24	Azithromycin	0	21	3/22/2017	81.3	HEIGHT	10.35	13.5	54	1	0
16	4082	36	Azithromycin	0	35	5/18/2018	93.2	HEIGHT	13.45454545	14.5	54	1	0
16	4083	12	Azithromycin	0	4	6/11/2016	62.8	LENGTH	6.15	12	18	1	0
16	4084	12	Azithromycin	1	54	6/11/2016	105.9	HEIGHT	14.5	14.5		1	0
16	4085	0	Azithromycin	1	48	3/26/2015	98.7	HEIGHT	14.05	15		1	0
16	4085	12	Azithromycin	1	66	6/11/2016	108.1	HEIGHT	16.15	15.5		0	0
16	4085	24	Azithromycin	1	77	3/22/2017	112.4	HEIGHT	18.2	15.5		0	0
16	4087	0	Azithromycin	1	36	3/26/2015	92.3	HEIGHT	14.8	16.5		1	0
16	4087	12	Azithromycin	1	54	6/11/2016	104.3	HEIGHT	17.85	17		1	0
16	4087	24	Azithromycin	1	65	3/22/2017	110.3	HEIGHT	20.45	17.5		0	0
16	4087	36	Azithromycin	1	80	5/18/2018	117.2	HEIGHT	21.18181818	16.5		0	0
16	4087	48	Azithromycin	1	91	4/18/2019	121.6	HEIGHT	21.6	16		0	0
16	4087	60	Azithromycin	1	100	2/10/2020	127.3	HEIGHT	26.4	17.7		0	0
16	4088	0	Azithromycin	1	36	3/26/2015	97.3	HEIGHT	13.1	14		1	0
16	4088	12	Azithromycin	1	51	6/11/2016	106.4	HEIGHT	14.55	14		1	0
16	4088	24	Azithromycin	1	71	3/22/2017	111.9	HEIGHT	15.35	13		0	0
16	4088	36	Azithromycin	1	86	5/18/2018	117.5	HEIGHT	17.68181818	14		0	0
16	4088	48	Azithromycin	1	97	4/18/2019	123	HEIGHT	19.25	14.5		0	0
16	4088	60	Azithromycin	1	107	2/10/2020	126.4	HEIGHT	21.45	15		0	0
16	4092	24	Azithromycin	0	7	3/22/2017	70.4	LENGTH	9.5	16		1	0

16	4092	36	Azithromycin	0	22	5/18/2018	82.7	HEIGHT	10.86363636	15.5		1	0
16	4092	48	Azithromycin	0	33	4/18/2019	87.6	HEIGHT	13.25	16		1	0
16	4092	60	Azithromycin	0	42	2/10/2020	93.9	HEIGHT	14.9	16.5		1	0
16	4093	24	Azithromycin	1	14	3/22/2017	82.8	HEIGHT	11.25	15		1	0
16	4093	36	Azithromycin	1	33	5/18/2018	91.4	HEIGHT	14.22727273	16		1	0
16	4093	48	Azithromycin	1	44	4/18/2019	97.7	HEIGHT	15.2	16		1	0
16	4093	60	Azithromycin	1	53	2/10/2020	101.7	HEIGHT	16.7	16.5		1	0
16	4098	24	Azithromycin	1	24	3/22/2017	92	HEIGHT	12.35	14		1	0
16	4098	36	Azithromycin	1	51	5/18/2018	101.3	HEIGHT	13.95454545	14		1	0
16	4101	0	Azithromycin	0	48	3/26/2015	103.8	HEIGHT	19.45	16.5		0	0
16	4101	12	Azithromycin	0	66	6/12/2016	112.3	HEIGHT	21	16.5		0	0
16	4101	24	Azithromycin	0	77	3/22/2017	117.4	HEIGHT	22.2	16		0	0
16	4101	36	Azithromycin	0	92	5/18/2018	122.6	HEIGHT	23.86363636	16.5		0	0
16	4105	0	Azithromycin	0	18	3/26/2015	78.8	HEIGHT	9.4	14		0	0
16	4105	12	Azithromycin	0	33	6/11/2016	86.5	HEIGHT	11.85	14.5		1	0
16	4105	36	Azithromycin	0	59	5/18/2018	98.2	HEIGHT	14.95454545	14.5		1	0
16	4105	48	Azithromycin	0	56	4/18/2019	104.3	HEIGHT	16	14.5		0	0
16	4105	60	Azithromycin	0	66	2/10/2020	108.4	HEIGHT	17.2	14.6		0	0
16	4106	12	Azithromycin	1	27	6/11/2016	82	HEIGHT	11.5	15		1	0
16	4109	0	Azithromycin	0	3	3/26/2015	66.6	LENGTH	6.75	13	60	1	0
16	4109	12	Azithromycin	0	30	6/11/2016	78.6	HEIGHT	10.1	15	60	1	0
16	4109	24	Azithromycin	0	26	3/22/2017	82.3	HEIGHT	10.95	14.5	60	0	0
16	4109	36	Azithromycin	0	41	5/18/2018	88	HEIGHT	11.95454545	14.5	60	1	0
16	4109	48	Azithromycin	0	52	4/18/2019	94.9	HEIGHT	13.45	15	60	1	0
16	4116	12	Azithromycin	0	11	6/11/2016	75.3	HEIGHT	8.65	14.5		1	0
16	4116	24	Azithromycin	0	20	3/22/2017	82.5	HEIGHT	10.7	14		1	0
16	4116	48	Azithromycin	0	46	4/18/2019	98.4	HEIGHT	13.1	15		1	0
16	4116	60	Azithromycin	0	56	2/10/2020	102.5	HEIGHT	14.6	15		1	0
16	4118	12	Azithromycin	0	30	6/11/2016	92.9	HEIGHT	12.65	14.5		1	0
16	4120	0	Azithromycin	0	24	3/26/2015	83.4	HEIGHT	10.45	14		1	0
16	4120	12	Azithromycin	0	42	6/11/2016	89.6	HEIGHT	11.65	13.5		0	0
16	4120	24	Azithromycin	0	53	3/22/2017	95.7	HEIGHT	14.05	14.5		1	0
16	4126	12	Azithromycin	0	18	6/11/2016	85.4	LENGTH	12.1	14.5		1	0
16	4128	24	Azithromycin	1	53	3/22/2017	93.5	HEIGHT	14.3	16		1	0
16	4129	0	Azithromycin	1	36	3/26/2015	92.1	HEIGHT	13.35	15		1	0
16	4129	12	Azithromycin	1	51	6/11/2016	97.3	HEIGHT	14	14.5		0	0
16	4129	24	Azithromycin	1	62	3/22/2017	103.8	HEIGHT	17.1	14.5		0	0
16	4130	0	Azithromycin	1	12	3/26/2015	69.3	LENGTH	7.4	12.5		1	0
16	4130	12	Azithromycin	1	25	6/11/2016	81.1	HEIGHT	9.45	14		1	0
16	4131	24	Azithromycin	1	42	3/22/2017	89.7	HEIGHT	12.9	14		1	0

16	4133	0	Azithromycin	1	30	3/26/2015	86.9	HEIGHT	10.5	14		1	0
16	4133	12	Azithromycin	1	45	6/11/2016	94.8	HEIGHT	11.65	13.5		0	0
16	4133	24	Azithromycin	1	56	3/22/2017	100.6	HEIGHT	12.45	13		1	0
16	4133	36	Azithromycin	1	71	5/18/2018	107.1	HEIGHT	14.36363636	14		0	0
16	4133	48	Azithromycin	1	82	4/18/2019	112	HEIGHT	14.8	13.5		0	0
16	4133	60	Azithromycin	1	91	2/10/2020	115.6	HEIGHT	16.35	14		0	0
16	4134	12	Azithromycin	1	42	6/11/2016	108.7	HEIGHT	18.85	15.5		1	0
16	4134	24	Azithromycin	1	53	3/22/2017	114.7	HEIGHT	19.7	14.5		1	0
16	4135	12	Azithromycin	1	36	6/11/2016	63.7	LENGTH	6.8	14		1	0
16	4135	24	Azithromycin	1	12	3/22/2017	75.1	HEIGHT	8.85	14		1	0
16	4135	36	Azithromycin	1	24	5/18/2018	85.8	HEIGHT	11.22727273	14.5		0	0
16	4135	48	Azithromycin	1	34	4/18/2019	93.2	HEIGHT	12.5	15		1	0
16	4135	60	Azithromycin	1	44	2/10/2020	99.2	HEIGHT	15.75	15.5		1	0
16	4138	0	Azithromycin	0	48	3/26/2015	94.7	HEIGHT	15.45	17.5		1	0
16	4138	12	Azithromycin	0	54	6/11/2016	101.5	HEIGHT	16.85	16.5		1	0
16	4138	24	Azithromycin	0	65	3/22/2017	106.2	HEIGHT	18.85	16		0	0
16	4138	36	Azithromycin	0	80	5/18/2018	111.7	HEIGHT	20.95454545	16		0	0
16	4138	48	Azithromycin	0	91	4/18/2019	115.5	HEIGHT	23.3	18		0	0
16	4138	60	Azithromycin	0	100	2/10/2020	120.1	HEIGHT	24.9	18.5		0	0
16	4139	24	Azithromycin	1	41	3/22/2017	92.6	HEIGHT	14.5	15		1	0
16	4139	36	Azithromycin	1	56	5/18/2018	101.2	HEIGHT	17.09090909	16.5		1	0
16	4140	0	Azithromycin	1	24	3/26/2015	88.2	HEIGHT	11.15	14		1	0
16	4140	12	Azithromycin	1	42	6/11/2016	97.1	HEIGHT	13.65	14		0	0
16	4140	24	Azithromycin	1	53	3/22/2017	102.2	HEIGHT	15	14.5		1	0
16	4140	36	Azithromycin	1	37	5/18/2018	108.5	HEIGHT	16.81818182	15		1	0
16	4140	48	Azithromycin	1	48	4/18/2019	113.7	HEIGHT	18.05	15.5		1	0
16	4140	60	Azithromycin	1	58	2/10/2020	117.6	HEIGHT	19.45	15.5		0	0
16	4141	0	Azithromycin	1	24	3/26/2015	71.8	LENGTH	8.7	15		1	0
16	4144	0	Azithromycin	0	48	3/26/2015	88.7	HEIGHT	13.65	15.5	24	0	0
16	4144	12	Azithromycin	0	54	6/11/2016	100.9	HEIGHT	16.45	16.5	24	1	0
16	4144	48	Azithromycin	0	91	4/18/2019	118	HEIGHT	21.95	18	24	0	0
16	4144	60	Azithromycin	0	100	2/10/2020	121.5	HEIGHT	24.05	17.5	24	0	0
16	4145	0	Azithromycin	1	48	3/26/2015	105.3	HEIGHT	16.2	15		1	0
16	4145	12	Azithromycin	1	78	6/11/2016	111.3	HEIGHT	17.65	15.5		0	0
16	4145	36	Azithromycin	1	104	5/18/2018	120	HEIGHT	21.54545455	15.5		0	0
16	4145	48	Azithromycin	1	115	4/18/2019	123.7	HEIGHT	23.7	17		0	0
16	4145	60	Azithromycin	0	124	2/10/2020	127.2	HEIGHT	25.55	18		0	0
16	4150	12	Azithromycin	1	30	6/11/2016	81	HEIGHT	10.8	15		1	0
16	4150	36	Azithromycin	1	40	5/18/2018	97.6	HEIGHT	14.95454545	15		1	0
16	4150	48	Azithromycin	1	51	4/18/2019	102.2	HEIGHT	16.25	16.5		1	0

16	4151	12	Azithromycin	0	48	6/11/2016	102.5	HEIGHT	15.25	15		1	0
16	4151	24	Azithromycin	0	59	3/22/2017	108.5	HEIGHT	17.35	14.5		1	0
16	4152	0	Azithromycin	0	4	3/26/2015	66	HEIGHT	7.75	15.5		1	0
16	4152	12	Azithromycin	0	15	6/11/2016	74.7	HEIGHT	8.7	14		1	0
16	4152	24	Azithromycin	0	26	3/22/2017	80.8	HEIGHT	10.7	14.5		1	0
16	4152	36	Azithromycin	0	41	5/18/2018	88.9	HEIGHT	12.45454545	14.5		1	0
16	4152	48	Azithromycin	0	52	4/18/2019	94.3	HEIGHT	13.45	14.5		0	0
16	4153	0	Azithromycin	0	48	3/26/2015	105.2	HEIGHT	16.45	15		1	0
16	4153	12	Azithromycin	0	63	6/11/2016	108.8	LENGTH	17.65	15		0	0
16	4153	36	Azithromycin	0	104	5/18/2018	120.4	HEIGHT	21.68181818	16		0	0
16	4153	48	Azithromycin	0	115	4/18/2019	124.5	HEIGHT	22.9	16		0	0
16	4153	60	Azithromycin	0	124	2/10/2020	127.3	HEIGHT	24.7	17		0	0
16	4154	12	Azithromycin	1	32	6/11/2016	91.7	HEIGHT	13.5	14		1	0
16	4155	24	Azithromycin	0	2	3/22/2017	60.8	LENGTH	6.35	12.5		1	0
16	4155	36	Azithromycin	0	17	5/18/2018	76.5	LENGTH	9.636363636	13		1	0
16	4155	48	Azithromycin	0	28	4/18/2019	84.8	HEIGHT	11.95	14.5		0	0
16	4155	60	Azithromycin	0	38	2/10/2020	91	HEIGHT	13.55	15		1	0
16	4156	0	Azithromycin	1	48	3/26/2015	99.9	HEIGHT	15.15	16		1	0
16	4156	12	Azithromycin	1	57	6/11/2016	107.8	HEIGHT	16.95	15.5		1	0
16	4156	24	Azithromycin	1	68	3/22/2017	113.8	HEIGHT	18.45	15.5		0	0
16	4156	36	Azithromycin	1	87	5/18/2018	119.3	HEIGHT	21.04545455	15		0	0
16	4156	48	Azithromycin	1	98	4/18/2019	124.5	HEIGHT	22.05	16.5		0	0
16	4156	60	Azithromycin	1	108	2/10/2020	128.6	HEIGHT	24.95	17		0	0
16	4164	12	Azithromycin	0	51	6/11/2016	89.4	LENGTH	11.3	14.5		1	0
16	4164	24	Azithromycin	0	53	3/22/2017	94.7	HEIGHT	12.4	13		1	0
16	4165	12	Azithromycin	1	44	6/11/2016	98.8	HEIGHT	15.45	15.5		1	0
16	4165	24	Azithromycin	1	53	3/22/2017	103.9	HEIGHT	16.4	15.5		1	0
16	4167	24	Azithromycin	1	3	3/22/2017	54	LENGTH	6.7	12.5		1	0
16	4167	36	Azithromycin	1	17	5/18/2018	81.7	HEIGHT	10.18181818	14		1	0
16	4167	48	Azithromycin	1	28	4/18/2019	90.8	HEIGHT	12.75	15		1	0
16	4167	60	Azithromycin	1	38	2/10/2020	98.2	HEIGHT	14.8	15.5		1	0
16	4168	0	Azithromycin	1	6	3/26/2015	69.9	LENGTH	8.85	15	18	1	0
16	4169	0	Azithromycin	1	12	3/26/2015	80.3	HEIGHT	10.35	14.5		1	0
16	4169	12	Azithromycin	1	27	6/11/2016	89.4	HEIGHT	12.7	15.5		1	0
16	4169	24	Azithromycin	1	38	3/22/2017	95.2	HEIGHT	14.25	15		0	0
16	4169	48	Azithromycin	1	64	4/18/2019	108.5	HEIGHT	17.8	15.5		0	0
16	4169	60	Azithromycin	1	73	2/10/2020	113	HEIGHT	20.4	15.1		0	0
16	4170	0	Azithromycin	1	5	3/26/2015	68.1	LENGTH	8.3	14.5		0	0
16	4170	36	Azithromycin	1	45	5/18/2018	94.3	HEIGHT	14.09090909	16		1	0
16	4170	48	Azithromycin	1	56	4/18/2019	101.2	HEIGHT	15.2	15.5		1	0

16	4170	60	Azithromycin	1	65	2/10/2020	107.4	HEIGHT	17	15		0	0
16	4172	12	Azithromycin	1	3	6/11/2016	64.9	LENGTH	7.1	14.5		0	0
16	4172	24	Azithromycin	1	14	3/22/2017	76	HEIGHT	9.25	14.5		1	0
16	4172	48	Azithromycin	1	39	4/18/2019	93.6	HEIGHT	13.7	15.5		1	0
16	4172	60	Azithromycin	1	49	2/10/2020	79.8	HEIGHT	7.522727273	16.5		1	0
16	4174	12	Azithromycin	0	7	6/11/2016	69	LENGTH	7.05	13		1	0
16	4174	24	Azithromycin	0	13	3/22/2017	77.9	HEIGHT	8.75	13.5		1	0
16	4174	36	Azithromycin	0	32	5/18/2018	86.3	HEIGHT	10.63636364	13		1	0
16	4174	48	Azithromycin	0	43	4/18/2019	94	HEIGHT	12.35	14		1	0
16	4174	60	Azithromycin	0	53	2/10/2020	100.2	HEIGHT	13.7	13.5		1	0
16	4176	24	Azithromycin	1	20	3/22/2017	80.4	HEIGHT	10.2	13.5	30	1	0
16	4177	0	Azithromycin	1	48	3/26/2015	86.7	HEIGHT	12.25	15		1	0
16	4177	12	Azithromycin	1	42	6/11/2016	95.9	HEIGHT	13.75	15.5		0	0
16	4177	24	Azithromycin	1	53	3/22/2017	101.5	HEIGHT	15.2	15		1	0
16	4177	36	Azithromycin	1	72	5/18/2018	109.1	HEIGHT	17.22727273	15		0	0
16	4177	48	Azithromycin	1	83	4/18/2019	114.1	HEIGHT	18.4	15.5		0	0
16	4177	60	Azithromycin	1	93	2/10/2020	117.8	HEIGHT	19.95	15.5		0	0
16	4178	0	Azithromycin	1	48	3/26/2015	108.1	HEIGHT	16.5	14.5		1	0
16	4178	12	Azithromycin	1	63	6/11/2016	113.3	HEIGHT	18.5	15		0	0
16	4178	36	Azithromycin	1	89	5/18/2018	123	HEIGHT	23.40909091	16.5		0	0
16	4179	24	Azithromycin	1	3	3/22/2017	57.8	LENGTH	5.7	13		1	0
16	4179	36	Azithromycin	1	17	5/18/2018	76.1	HEIGHT	9.772727273	14.5		1	0
16	4179	48	Azithromycin	1	28	4/18/2019	85	HEIGHT	11.3	14		1	0
16	4180	0	Azithromycin	0	18	3/26/2015	75	HEIGHT	8	12		1	0
16	4180	12	Azithromycin	0	33	6/11/2016	82.3	HEIGHT	10.4	14		1	0
16	4180	36	Azithromycin	0	59	5/18/2018	96.6	HEIGHT	13.45454545	13.5		1	0
16	4180	48	Azithromycin	0	70	4/18/2019	99.7	HEIGHT	13.5	13		0	0
16	4180	60	Azithromycin	0	79	2/10/2020	102.2	HEIGHT	14.95	13.5		0	0
16	4181	24	Azithromycin	1	37	3/22/2017	88.1	HEIGHT	11.85	14		1	0
16	4181	36	Azithromycin	1	52	5/18/2018	97.5	HEIGHT	14.27272727	15		1	0
16	4183	0	Azithromycin	0	36	3/26/2015	87.5	HEIGHT	12	15		1	0
16	4183	12	Azithromycin	0	42	6/11/2016	99.8	HEIGHT	14.2	14.5		1	0
16	4186	0	Azithromycin	1	24	3/26/2015	88.8	HEIGHT	10.9	14.5		1	0
16	4186	24	Azithromycin	1	50	3/22/2017	97.5	HEIGHT	16.4	15.5		0	0
16	4186	36	Azithromycin	1	66	5/18/2018	104.2	HEIGHT	17.95454545	16.5		0	0
16	4186	48	Azithromycin	1	77	4/18/2019	109.8	HEIGHT	18.7	16.5		0	0
16	4186	60	Azithromycin	1	87	2/10/2020	113.3	HEIGHT	21.55	17.1		0	0
16	4188	0	Azithromycin	1	5	3/26/2015	68.6	LENGTH	7.2	13.5		1	0
16	4188	12	Azithromycin	1	15	6/11/2016	81.3	LENGTH	9.15	13.5		0	0
16	4188	24	Azithromycin	1	26	3/22/2017	89	HEIGHT	11.7	14		0	0

16	4190	0	Azithromycin	1	9	3/26/2015	66.6	LENGTH	6.3	13.5	1	0
16	4190	12	Azithromycin	1	30	6/11/2016	76.1	HEIGHT	7.8	13	1	0
16	4190	36	Azithromycin	1	56	5/18/2018	90.2	HEIGHT	11.31818182	13.5	1	0
16	4192	12	Azithromycin	1	32	6/11/2016	84.9	HEIGHT	12.65	16	1	0
16	4192	24	Azithromycin	1	41	3/22/2017	96.3	HEIGHT	12.4	13.5	1	0
16	4196	0	Azithromycin	0	24	3/26/2015	78.8	HEIGHT	10.4	14	1	0
16	4199	0	Azithromycin	0	48	3/26/2015	103.2	HEIGHT	16.35	15	0	0
16	4199	12	Azithromycin	0	63	6/11/2016	109.1	HEIGHT	17.4	15.5	0	0
16	4199	24	Azithromycin	0	74	3/22/2017	112.3	HEIGHT	19.05	15	0	0
16	4199	36	Azithromycin	0	89	5/18/2018	116.2	HEIGHT	20.95454545	16	0	0
16	4201	0	Azithromycin	1	42	3/26/2015	98.7	HEIGHT	15.2	15.5	1	0
16	4201	12	Azithromycin	1	59	6/11/2016	105.8	HEIGHT	16.95	15.5	0	0
16	4201	24	Azithromycin	1	68	3/22/2017	110.3	HEIGHT	17.05	13.5	0	0
16	4201	36	Azithromycin	1	83	5/18/2018	115.2	HEIGHT	19.04545455	15	0	0
16	4201	48	Azithromycin	1	94	4/18/2019	118.2	HEIGHT	20.1	14.5	0	0
16	4201	60	Azithromycin	1	103	2/10/2020	120.5	HEIGHT	21.05	15	0	0
16	4202	0	Azithromycin	1	24	3/26/2015	79.6	HEIGHT	10.85	16.5	1	0
16	4202	12	Azithromycin	1	30	6/11/2016	88.3	HEIGHT	12.95	15	1	0
16	4202	24	Azithromycin	0	41	3/22/2017	94.9	HEIGHT	14.55	15	1	0
16	4202	36	Azithromycin	0	56	5/18/2018	102.2	HEIGHT	16.40909091	16	1	0
16	4202	48	Azithromycin	0	67	4/18/2019	107.4	HEIGHT	17.1	15.5	0	0
16	4202	60	Azithromycin	0	76	2/10/2020	111.4	HEIGHT	19.15	16	0	0
16	4203	12	Azithromycin	1	56	6/11/2016	107	HEIGHT	14.75	14.5	1	0
16	4205	0	Azithromycin	1	24	3/26/2015	77.9	HEIGHT	10.15	13	1	0
16	4205	12	Azithromycin	1	39	6/11/2016	88	LENGTH	12.9	15	1	0
16	4205	24	Azithromycin	1	41	3/22/2017	92.5	HEIGHT	13.6	14.5	1	0
16	4205	36	Azithromycin	1	56	5/18/2018	100.3	HEIGHT	15.04545455	14	1	0
16	4205	48	Azithromycin	1	67	4/18/2019	105.2	HEIGHT	16.5	14.5	0	0
16	4205	60	Azithromycin	1	76	2/10/2020	108.9	HEIGHT	17.8	15	0	0
16	4209	0	Azithromycin	1	48	3/26/2015	106.6	HEIGHT	17.75	15	1	0
16	4209	12	Azithromycin	1	63	6/12/2016	111	HEIGHT	18.25	15.2	0	0
16	4209	48	Azithromycin	1	98	4/18/2019	125.4	HEIGHT	24.9	17.5	0	0
16	4210	12	Azithromycin	1	10	6/11/2016	69	HEIGHT	7.3	13.5	1	0
16	4210	24	Azithromycin	1	20	3/22/2017	78.4	HEIGHT	9	12.5	1	0
16	4210	36	Azithromycin	1	36	5/18/2018	84.2	HEIGHT	12.13636364	14.5	1	0
16	4210	48	Azithromycin	1	47	4/18/2019	92.7	HEIGHT	12.9	15.5	1	0
16	4210	60	Azithromycin	1	56	2/10/2020	98.2	HEIGHT	14.2	15	1	0
16	4211	0	Azithromycin	1	54	3/26/2015	111.7	HEIGHT	19.05	16.5	1	0
16	4211	12	Azithromycin	1	66	6/11/2016	118	HEIGHT	20.7	16	0	0
16	4211	24	Azithromycin	1	77	3/22/2017	122.2	HEIGHT	22.9	16	0	0

16	4211	36	Azithromycin	1	114	5/18/2018	128.6	HEIGHT	25.31818182	17	0	0
16	4211	48	Azithromycin	1	125	4/18/2019	132.4	HEIGHT	28	17	0	0
16	4211	60	Azithromycin	1	134	2/10/2020	135.2	HEIGHT	30.3	17.5	0	0
16	4212	24	Azithromycin	0	53	3/22/2017	103.6	HEIGHT	16.8	15.5	1	0
16	4215	0	Azithromycin	1	18	3/26/2015	82	HEIGHT	9.65	13	1	0
16	4215	12	Azithromycin	1	33	6/11/2016	91.9	HEIGHT	12	14	0	0
16	4220	24	Azithromycin	0	3	3/22/2017	57.1	LENGTH	5.55	12.5	1	0
16	4220	36	Azithromycin	0	15	5/18/2018	75	LENGTH	8.818181818	12.5	1	0
16	4220	48	Azithromycin	0	26	4/18/2019	84.2	HEIGHT	10.95	14	1	0
16	4220	60	Azithromycin	0	35	2/10/2020	90.4	HEIGHT	13.05	14.2	0	0
16	4222	0	Azithromycin	1	24	3/26/2015	84.4	HEIGHT	10.45	14.5	1	0
16	4222	12	Azithromycin	1	39	6/11/2016	89.7	LENGTH	12.2	15	0	0
16	4222	24	Azithromycin	1	53	3/22/2017	96.4	HEIGHT	13.7	14.5	1	0
16	4222	36	Azithromycin	1	68	5/18/2018	103.8	HEIGHT	15.63636364	14.5	0	0
16	4222	48	Azithromycin	1	75	4/18/2019	109.9	HEIGHT	17.75	15	0	0
16	4222	60	Azithromycin	1	85	2/10/2020	114.9	HEIGHT	20.65	16	0	0
16	4223	0	Azithromycin	0	6	3/26/2015	70.5	LENGTH	7.7	14	0	0
16	4223	12	Azithromycin	0	20	6/11/2016	80.9	LENGTH	9.7	14	1	0
16	4223	24	Azithromycin	0	29	3/22/2017	87.2	HEIGHT	11.4	15	1	0
16	4223	36	Azithromycin	0	44	5/18/2018	95.3	HEIGHT	14.13636364	15	1	0
16	4223	60	Azithromycin	0	64	2/10/2020	105.1	HEIGHT	16.3	15.3	0	0
16	4226	24	Azithromycin	0	29	3/22/2017	86.1	HEIGHT	11.95	16	1	0
16	4226	36	Azithromycin	0	44	5/18/2018	95.5	HEIGHT	13.81818182	15	1	0
16	4227	0	Azithromycin	1	48	3/26/2015	100.8	HEIGHT	13.95	14	1	0
16	4227	24	Azithromycin	1	89	3/22/2017	110.2	HEIGHT	17.05	14.5	0	0
16	4227	36	Azithromycin	1	104	5/18/2018	114.7	HEIGHT	18.40909091	15	0	0
16	4227	48	Azithromycin	1	115	4/18/2019	119	HEIGHT	20.05	16	0	0
16	4227	60	Azithromycin	1	124	2/10/2020	121.7	HEIGHT	22.65	15.7	0	0
16	4230	12	Azithromycin	1	51	6/11/2016	85.6	LENGTH	10.3	14	1	0
16	4230	24	Azithromycin	1	53	3/22/2017	90.7	HEIGHT	11.5	13.5	1	0
16	4231	0	Azithromycin	1	12	3/26/2015	71.5	LENGTH	8.75	15	1	0
16	4236	24	Azithromycin	0	7	3/22/2017	67.3	LENGTH	7.7	13.5	1	0
16	4238	0	Azithromycin	1	48	3/26/2015	95.9	HEIGHT	12.5	13.5	1	0
16	4238	12	Azithromycin	1	63	6/11/2016	100.4	HEIGHT	13.1	13	0	0
16	4238	24	Azithromycin	1	74	3/22/2017	106.9	HEIGHT	14.85	13.5	0	0
16	4238	36	Azithromycin	1	89	5/18/2018	113.1	HEIGHT	17.18181818	14	0	0
16	4238	48	Azithromycin	1	100	4/18/2019	117.3	HEIGHT	19	15	0	0
16	4238	60	Azithromycin	1	109	2/10/2020	122.6	HEIGHT	20.75	15	0	0
16	4240	0	Azithromycin	1	36	3/26/2015	84.9	HEIGHT	10.8	14.5	1	0
16	4240	24	Azithromycin	1	26	3/22/2017	99.2	HEIGHT	14.35	14.5	1	0

16	4240	36	Azithromycin	1	41	5/18/2018	106.9	HEIGHT	15.77272727	15		1	0
16	4240	48	Azithromycin	1	52	4/18/2019	110.6	HEIGHT	17.45	15.5		1	0
16	4240	60	Azithromycin	1	61	2/10/2020	114.3	HEIGHT	19.1	16.1		0	0
16	4241	12	Azithromycin	1	3	6/11/2016	63.3	LENGTH	7.1	13.5		1	0
16	4241	24	Azithromycin	1	13	3/22/2017	74.1	LENGTH	9.4	14.5		1	0
16	4241	36	Azithromycin	1	28	5/18/2018	85.5	HEIGHT	11.36363636	14.5		1	0
16	4241	48	Azithromycin	1	39	4/18/2019	93.2	HEIGHT	12.75	15		1	0
16	4241	60	Azithromycin	1	49	2/10/2020	98.6	HEIGHT	15.4	15		0	0
16	4243	12	Azithromycin	0	3	6/11/2016	66.1	LENGTH	6.7	13.5		0	0
16	4243	36	Azithromycin	0	27	5/18/2018	88.9	HEIGHT	11.90909091	15		1	0
16	4243	48	Azithromycin	0	38	4/18/2019	96.2	HEIGHT	13.9	16.5		1	0
16	4243	60	Azithromycin	0	48	2/10/2020	103	HEIGHT	15.9	16		1	0
16	4246	0	Azithromycin	0	48	3/26/2015	113.4	HEIGHT	17.65	14.5		1	0
16	4246	12	Azithromycin	0	78	6/11/2016	120	HEIGHT	20.3	14.5		0	0
16	4246	36	Azithromycin	0	108	5/18/2018	129.5	HEIGHT	24.68181818	16		0	0
16	4246	48	Azithromycin	0	119	4/18/2019	134.6	HEIGHT	27	16.5		0	0
16	4246	60	Azithromycin	0	129	2/10/2020	137.2	HEIGHT	29.05	16.7		0	0
16	4248	12	Azithromycin	0	9	6/11/2016	67.3	LENGTH	7.15	14	60	0	0
16	4248	24	Azithromycin	0	14	3/22/2017	76.3	HEIGHT	8.5	13	60	1	0
16	4248	36	Azithromycin	0	31	5/18/2018	85.4	HEIGHT	10.59090909	15	60	1	0
16	4248	48	Azithromycin	0	42	4/18/2019	92.6	HEIGHT	12	16	60	1	0
16	4249	0	Azithromycin	0	9	3/26/2015	66.2	LENGTH	5.35	11	6	0	0
16	4250	0	Azithromycin	1	36	3/26/2015	88.7	HEIGHT	12.4	15.5		1	0
16	4250	12	Azithromycin	1	51	6/11/2016	96.6	HEIGHT	12.35	14		1	0
16	4250	24	Azithromycin	1	62	3/22/2017	103.1	HEIGHT	14.55	14		0	0
16	4250	36	Azithromycin	1	77	5/18/2018	109.4	HEIGHT	16.27272727	15		0	0
16	4250	48	Azithromycin	1	88	4/18/2019	115.1	HEIGHT	18.35	15		0	0
16	4250	60	Azithromycin	1	97	2/10/2020	120.2	HEIGHT	19.95	15.5		0	0
16	4251	0	Azithromycin	0	36	3/26/2015	91.7	HEIGHT	12.55	15		1	0
16	4251	12	Azithromycin	0	54	6/11/2016	99	HEIGHT	15.65	16		1	0
16	4251	24	Azithromycin	0	65	3/22/2017	104.8	HEIGHT	17.65	16.5		0	0
16	4251	36	Azithromycin	0	80	5/18/2018	111	HEIGHT	19.40909091	16.5		0	0
16	4251	48	Azithromycin	0	91	4/18/2019	118.6	HEIGHT	21.9	17		0	0
16	4251	60	Azithromycin	0	100	2/10/2020	123.6	HEIGHT	23.95	17		0	0
16	4252	0	Azithromycin	0	36	3/26/2015	101.4	HEIGHT	15.4	14.5		0	0
16	4252	12	Azithromycin	0	54	6/11/2016	107.8	HEIGHT	16.75	15.5		1	0
16	4252	24	Azithromycin	0	65	3/22/2017	113.3	HEIGHT	18.8	14.5		0	0
16	4256	24	Azithromycin	1	6	3/22/2017	66.1	LENGTH	6.65	12.5		1	0
16	4256	36	Azithromycin	1	20	5/18/2018	82.9	HEIGHT	9.818181818	14		1	0
16	4256	48	Azithromycin	1	31	4/18/2019	90.6	HEIGHT	12.15	15.5		1	0

16	4256	60	Azithromycin	1	41	2/10/2020	97.1	HEIGHT	13.9	15.5	0	0
16	4257	24	Azithromycin	0	53	3/22/2017	96.6	HEIGHT	15	15	1	0
16	4259	12	Azithromycin	1	12	6/11/2016	69.4	HEIGHT	7.05	12.5	1	0
16	4259	36	Azithromycin	1	37	5/18/2018	85.5	HEIGHT	9.818181818	13	1	0
16	8006	36	Azithromycin	1	2	5/18/2018	59.8	LENGTH	6.5	15	1	0
16	8006	48	Azithromycin	1	13	4/18/2019	73.7	LENGTH	8.85	15.5	1	0
16	8006	60	Azithromycin	1	23	3/8/2020	79.6	HEIGHT	9.55	13.5	1	0
16	8046	48	Azithromycin	0	3	4/18/2019	60.6	LENGTH	5.7	13.5	1	0
16	8046	60	Azithromycin	0	12	2/10/2020	72.6	HEIGHT	7.55	12	1	0
16	8070	60	Azithromycin	0	10	2/10/2020	70.5	LENGTH	8.25	14.5	1	0
16	8099	36	Azithromycin	0	3	5/18/2018	66.8	LENGTH	7.136363636	13	1	0
16	8099	48	Azithromycin	0	12	4/18/2019	80.8	LENGTH	10.05	15	1	0
16	8099	60	Azithromycin	0	21	2/10/2020	88.9	LENGTH	12.75	15	1	0
16	8156	36	Azithromycin	0	23	5/18/2018	80.5	HEIGHT	8.727272727	13	1	0
16	8156	60	Azithromycin	0	43	3/8/2020	92	HEIGHT	12.15	14.5	1	0
16	8212	60	Azithromycin	0	11	3/8/2020	79.1	HEIGHT	8.8	14	1	0
16	8220	36	Azithromycin	0	2	5/18/2018	64	LENGTH	8.863636364	17.5	1	0
16	8220	48	Azithromycin	0	13	4/18/2019	78.8	LENGTH	11.45	17	1	0
16	8220	60	Azithromycin	0	23	2/10/2020	86.1	HEIGHT	12.8	16	1	0
16	8224	36	Azithromycin	0	15	5/18/2018	74	HEIGHT	8.818181818	14	1	0
16	8224	60	Azithromycin	0	37	2/10/2020	86.7	HEIGHT	12.45	15.8	1	0
16	8265	48	Azithromycin	0	22	4/18/2019	79.5	LENGTH	9.3	13	1	0
16	8265	60	Azithromycin	0	32	2/10/2020	82.9	HEIGHT	11.3	13.5	1	0
16	8323	48	Azithromycin	1	15	4/18/2019	80.7	LENGTH	10.4	15	1	0
16	8323	60	Azithromycin	1	25	2/10/2020	85.3	HEIGHT	12.7	15.5	1	0
16	8331	48	Azithromycin	1	15	4/18/2019	75	LENGTH	8.65	13.5	1	0
16	8355	36	Azithromycin	1	2	5/18/2018	56.4	LENGTH	5.045454545	11.5	1	0
16	8355	48	Azithromycin	1	13	4/18/2019	68.3	LENGTH	7.55	13.5	1	0
16	8355	60	Azithromycin	1	23	2/10/2020	77.4	HEIGHT	10.3	14.5	1	0
16	8492	60	Azithromycin	0	3	2/10/2020	64.9	HEIGHT	6.8	13.5	1	0
16	8520	48	Azithromycin	0	10	4/18/2019	67	LENGTH	7.15	14	1	0
16	8520	60	Azithromycin	0	19	3/8/2020	78.6	HEIGHT	9.55	14	0	0
16	8530	36	Azithromycin	1	3	5/18/2018	59.6	LENGTH	5.727272727	12	1	0
16	8530	48	Azithromycin	1	12	4/18/2019	74.5	HEIGHT	7.9	12.5	0	0
16	8530	60	Azithromycin	1	23	2/10/2020	79.7	HEIGHT	10.1	13.6	1	0
16	8531	48	Azithromycin	1	10	4/18/2019	67.9	LENGTH	7	13.5	1	0
16	8531	60	Azithromycin	1	19	3/8/2020	77.4	LENGTH	9	12.5	0	0
16	8681	60	Azithromycin	0	32	2/10/2020	89.3	HEIGHT	15.15	17.5	1	0
16	8682	48	Azithromycin	1	3	4/18/2019	57.9	LENGTH	5.25	13	1	0
16	8682	60	Azithromycin	1	12	2/10/2020	67.8	HEIGHT	7.35	13	1	0

16	8708	48	Azithromycin	1	15	4/18/2019	75.7	LENGTH	9.35	15		1	0
16	8722	48	Azithromycin	0	8	4/18/2019	72.2	LENGTH	8.5	14		1	0
16	8722	60	Azithromycin	0	14	2/10/2020	79.2	HEIGHT	9.85	14.3		0	0
16	8826	36	Azithromycin	0	4	5/18/2018	62.7	LENGTH	7.409090909	14.5		1	0
16	8826	48	Azithromycin	0	15	4/18/2019	74.8	LENGTH	8.9	14		1	0
16	8826	60	Azithromycin	0	24	2/10/2020	82.9	HEIGHT	11.35	14.2		1	0
16	8827	48	Azithromycin	1	36	4/18/2019	102.7	HEIGHT	15.75	15		1	0
16	8827	60	Azithromycin	1	45	2/10/2020	108.4	HEIGHT	16.95	14.5		1	0
16	8949	60	Azithromycin	1	8	2/10/2020	67.2	LENGTH	7.4	12.5		1	0
16	8978	48	Azithromycin	1	39	4/18/2019	97.1	HEIGHT	15.15	16		1	0
16	8978	60	Azithromycin	1	49	2/10/2020	101.7	HEIGHT	17.25	15.5		1	0
16	9015	60	Azithromycin	0	42	2/10/2020	101.4	HEIGHT	16.1	15		1	0
16	9067	36	Azithromycin	0	12	5/18/2018	72.9	LENGTH	8	14.5		1	0
16	9147	60	Azithromycin	0	2	2/10/2020	61.6	LENGTH	6.7	13.5		1	0
16	9198	48	Azithromycin	1	6	4/18/2019	62.6	LENGTH	5.8	13		1	0
16	9198	60	Azithromycin	1	14	2/10/2020	70.8	LENGTH	7.75	13.3		1	0
16	9200	60	Azithromycin	0	43	2/10/2020	95.9	HEIGHT	15.65	16.5		1	0
16	9205	48	Azithromycin	1	8	4/18/2019	67.9	LENGTH	7.25	12.5		1	0
16	9205	60	Azithromycin	1	14	3/8/2020	78.3	LENGTH	9.45	13		1	0
16	9227	36	Azithromycin	0	18	5/18/2018	76.2	HEIGHT	9.090909091	14		1	0
16	9227	48	Azithromycin	0	29	4/18/2019	83.3	HEIGHT	10.3	13.5		1	0
16	9227	60	Azithromycin	0	39	2/10/2020	88.6	HEIGHT	11.7	14		1	0
16	9247	48	Azithromycin	1	1	4/18/2019	56	LENGTH	4.8	11.5		1	0
16	9247	60	Azithromycin	1	10	2/10/2020	71.9	LENGTH	7.1	12		1	0
16	9256	36	Azithromycin	1	1	5/18/2018	53.3	LENGTH	5.045454545	12.5		1	0
16	9256	48	Azithromycin	1	11	4/18/2019	74.2	LENGTH	9.1	14		1	0
16	9263	48	Azithromycin	0	12	4/18/2019	76.9	LENGTH	10.6	15.5		1	0
16	9263	60	Azithromycin	0	21	3/8/2020	84.3	HEIGHT	12.95	14.5		1	0
16	9382	36	Azithromycin	0	15	5/18/2018	72.6	HEIGHT	7.818181818	13		1	0
16	9382	48	Azithromycin	0	26	4/18/2019	77.6	HEIGHT	9.35	14		1	0
16	9382	60	Azithromycin	0	38	2/10/2020	81.6	HEIGHT	11	14.3		1	0
16	9414	48	Azithromycin	0	8	4/18/2019	69	LENGTH	7.6	14.5		1	0
16	9414	60	Azithromycin	0	17	2/10/2020	77.5	LENGTH	9.75	14		1	0
16	9451	60	Azithromycin	0	30	2/10/2020	85.5	HEIGHT	10.5	13		1	0
17	4269	0	Azithromycin	0	12	3/28/2015	72	LENGTH	8.4	13	12	1	0
17	4270	0	Azithromycin	0	8	3/28/2015	73.6	LENGTH	10.3	16		1	0
17	4270	24	Azithromycin	0	43	4/27/2017	91.9	HEIGHT	15.25	16		1	0
17	4270	36	Azithromycin	0	56	5/16/2018	99.6	HEIGHT	17.65	16		1	0
17	4270	48	Azithromycin	0	67	4/22/2019	103.6	HEIGHT	17.55	14.5		0	0
17	4270	60	Azithromycin	0	76	2/11/2020	108.4	HEIGHT	21.45	17		0	0

17	4271	24	Azithromycin	1	4	5/18/2017	59.3	LENGTH	5	11.5	1	0
17	4271	48	Azithromycin	1	27	4/22/2019	83.3	HEIGHT	11	14	1	0
17	4276	12	Azithromycin	1	24	6/13/2016	95.4	HEIGHT	13.7	15.5	1	0
17	4276	48	Azithromycin	1	60	4/22/2019	113.6	HEIGHT	20.3	16	0	0
17	4277	0	Azithromycin	1	48	3/28/2015	101	HEIGHT	14.8	15	1	0
17	4277	12	Azithromycin	1	54	6/13/2016	109	HEIGHT	16.25	15.5	1	0
17	4277	24	Azithromycin	1	67	4/25/2017	115.6	HEIGHT	17.09090909	14.5	0	0
17	4277	36	Azithromycin	1	80	5/16/2018	122.2	HEIGHT	20.6	16	0	0
17	4277	48	Azithromycin	1	91	4/22/2019	126.5	HEIGHT	22.85	16.5	0	0
17	4277	60	Azithromycin	1	100	2/11/2020	131.6	HEIGHT	25.6	17.5	0	0
17	4279	0	Azithromycin	1	18	3/28/2015	71.5	LENGTH	7.2	12.5	1	0
17	4279	24	Azithromycin	1	37	4/25/2017	92.5	HEIGHT	12.5	15	1	0
17	4279	36	Azithromycin	1	50	5/16/2018	100.8	HEIGHT	13.5	14	1	0
17	4279	48	Azithromycin	1	51	4/22/2019	105	HEIGHT	15.05	15	0	0
17	4279	60	Azithromycin	1	61	2/11/2020	111.8	HEIGHT	17.1	14.5	0	0
17	4280	12	Azithromycin	0	10	6/13/2016	74.9	LENGTH	8.7	15.5	1	0
17	4280	24	Azithromycin	0	21	4/25/2017	79.8	HEIGHT	10.63636364	14.5	1	0
17	4280	36	Azithromycin	0	34	5/16/2018	89.1	HEIGHT	12.75	15.5	1	0
17	4280	48	Azithromycin	0	45	4/22/2019	96.4	HEIGHT	14.5	14	1	0
17	4280	60	Azithromycin	0	55	2/11/2020	101.8	HEIGHT	15.3	14.5	1	0
17	4282	0	Azithromycin	0	36	3/28/2015	99.8	HEIGHT	14.7	14.5	1	0
17	4282	12	Azithromycin	0	47	6/13/2016	105.4	HEIGHT	16.5	15.5	1	0
17	4282	24	Azithromycin	0	58	4/27/2017	109.7	HEIGHT	18.1	15	1	0
17	4282	36	Azithromycin	0	71	5/16/2018	113.7	HEIGHT	18.65	15.5	0	0
17	4283	12	Azithromycin	0	7	6/14/2016	69.3	LENGTH	7.45	13.5	1	0
17	4283	24	Azithromycin	0	16	4/25/2017	79.3	HEIGHT	10.09090909	14	1	0
17	4283	48	Azithromycin	0	39	4/22/2019	93.4	HEIGHT	13.5	14.5	1	0
17	4283	60	Azithromycin	0	49	2/11/2020	98.4	HEIGHT	15.2	14.5	1	0
17	4284	0	Azithromycin	0	48	3/28/2015	101.8	HEIGHT	15.9	15	1	0
17	4284	12	Azithromycin	0	54	6/13/2016	106.7	HEIGHT	16.85	15.5	1	0
17	4284	24	Azithromycin	0	70	4/27/2017	111.8	HEIGHT	19.05	16.5	0	0
17	4284	36	Azithromycin	0	83	5/16/2018	116.1	HEIGHT	20.3	16.5	0	0
17	4284	48	Azithromycin	0	94	4/22/2019	118.4	HEIGHT	22.95	17	0	0
17	4284	60	Azithromycin	0	103	2/11/2020	122.2	HEIGHT	24.4	17.5	0	0
17	4285	0	Azithromycin	1	36	3/28/2015	101.5	HEIGHT	13.35	13.5	1	0
17	4285	12	Azithromycin	1	48	6/13/2016	108.5	HEIGHT	15.1	13.5	1	0
17	4285	24	Azithromycin	1	61	4/25/2017	113	HEIGHT	16.36363636	14.5	0	0
17	4285	36	Azithromycin	1	74	5/16/2018	120	HEIGHT	19.6	15	0	0
17	4285	48	Azithromycin	1	85	4/22/2019	124.4	HEIGHT	21.9	15	0	0
17	4285	60	Azithromycin	1	94	2/11/2020	127.8	HEIGHT	23.25	16	0	0

17	4286	12	Azithromycin	0	56	6/13/2016	124	HEIGHT	22.85	17	1	0
17	4286	48	Azithromycin	0	91	4/22/2019	135.6	HEIGHT	29.85	18	0	0
17	4288	12	Azithromycin	1	51	6/13/2016	94.3	HEIGHT	12.5	14.5	1	0
17	4290	12	Azithromycin	0	51	6/13/2016	97.5	HEIGHT	14.65	15.5	1	0
17	4290	48	Azithromycin	0	86	4/22/2019	113.7	HEIGHT	19.45	15	0	0
17	4290	60	Azithromycin	0	95	2/11/2020	116.4	HEIGHT	20.9	15.8	0	0
17	4291	0	Azithromycin	1	8	3/28/2015	66.3	LENGTH	6.85	14	1	0
17	4291	12	Azithromycin	1	18	6/13/2016	80	LENGTH	9.05	14.5	1	0
17	4291	24	Azithromycin	1	31	5/18/2017	88.7	HEIGHT	11.13636364	13.5	1	0
17	4291	36	Azithromycin	1	44	5/16/2018	94.9	HEIGHT	12.5	14.5	1	0
17	4291	48	Azithromycin	1	55	4/22/2019	100.5	HEIGHT	13.75	14	1	0
17	4292	0	Azithromycin	1	36	3/28/2015	82.2	HEIGHT	11	14	1	0
17	4292	12	Azithromycin	1	42	6/13/2016	87	HEIGHT	13.3	15	1	0
17	4292	36	Azithromycin	1	68	5/16/2018	109.5	HEIGHT	16.95	15.5	0	0
17	4292	48	Azithromycin	1	79	4/22/2019	114	HEIGHT	18.5	15	0	0
17	4293	12	Azithromycin	1	4	6/13/2016	64.4	LENGTH	6.65	13.5	1	0
17	4293	24	Azithromycin	1	16	4/25/2017	73.8	HEIGHT	7.909090909	13.5	1	0
17	4293	36	Azithromycin	1	30	5/16/2018	85.4	HEIGHT	10.7	15	1	0
17	4295	12	Azithromycin	1	42	6/13/2016	87.6	HEIGHT	11.25	14	1	0
17	4295	24	Azithromycin	1	55	4/25/2017	94.5	HEIGHT	12.90909091	14.5	1	0
17	4295	48	Azithromycin	1	79	4/22/2019	105.7	HEIGHT	15.95	15	0	0
17	4295	60	Azithromycin	1	88	2/11/2020	109.7	HEIGHT	17.5	15.3	0	0
17	4296	0	Azithromycin	0	7	3/28/2015	67.7	LENGTH	6.45	12.5	1	0
17	4296	12	Azithromycin	0	8	6/13/2016	80.4	HEIGHT	8.65	13.5	1	0
17	4296	24	Azithromycin	0	16	4/25/2017	88.3	HEIGHT	10.72727273	13.5	1	0
17	4296	36	Azithromycin	0	46	5/16/2018	95.6	HEIGHT	12.9	13.5	1	0
17	4296	48	Azithromycin	0	57	4/22/2019	100.3	HEIGHT	14.4	13.5	1	0
17	4297	0	Azithromycin	0	36	3/28/2015	95.3	HEIGHT	15.6	16	1	0
17	4297	12	Azithromycin	0	42	6/13/2016	102.4	HEIGHT	17	17	1	0
17	4297	24	Azithromycin	0	55	4/27/2017	108.6	HEIGHT	18.9	16.5	1	0
17	4297	36	Azithromycin	0	68	5/16/2018	113.1	HEIGHT	20.1	17	0	0
17	4297	48	Azithromycin	0	79	4/22/2019	118	HEIGHT	23.05	17	0	0
17	4297	60	Azithromycin	0	88	2/11/2020	121.9	HEIGHT	24.6	17.9	0	0
17	4298	0	Azithromycin	1	3	3/28/2015	60.6	LENGTH	5.45	13.5	1	0
17	4298	12	Azithromycin	1	17	6/13/2016	74.3	LENGTH	8.2	14.5	1	0
17	4298	36	Azithromycin	1	41	5/16/2018	89.6	HEIGHT	11.35	15	1	0
17	4298	60	Azithromycin	1	61	2/11/2020	102.6	HEIGHT	14.05	15	0	0
17	4299	0	Azithromycin	1	7	3/28/2015	74.9	HEIGHT	8.75	14	1	0
17	4299	12	Azithromycin	1	17	6/14/2016	82.6	HEIGHT	11.85	15.5	1	0
17	4300	12	Azithromycin	1	1	6/13/2016	56.7	LENGTH	4.45	11.5	1	0

17	4300	24	Azithromycin	1	12	4/25/2017	75.5	HEIGHT	9.272727273	15	1	0
17	4300	36	Azithromycin	1	39	5/16/2018	86.3	HEIGHT	11.3	14.5	1	0
17	4300	48	Azithromycin	1	50	4/22/2019	94.3	HEIGHT	14.1	14.5	1	0
17	4301	12	Azithromycin	1	50	6/14/2016	86.4	HEIGHT	11.15	15	1	0
17	4301	36	Azithromycin	1	59	5/16/2018	102	HEIGHT	13.3	13.5	1	0
17	4302	12	Azithromycin	0	8	6/13/2016	70.9	HEIGHT	8.4	14.5	1	0
17	4302	24	Azithromycin	0	16	5/18/2017	80.7	HEIGHT	9.6	13	1	0
17	4302	36	Azithromycin	0	35	5/16/2018	88.5	HEIGHT	11.55	13.5	1	0
17	4302	48	Azithromycin	0	46	4/22/2019	94.2	HEIGHT	12.3	13	1	0
17	4302	60	Azithromycin	0	55	2/11/2020	100.2	HEIGHT	14.35	13.6	1	0
17	4303	0	Azithromycin	0	24	3/28/2015	89.5	HEIGHT	13.15	16	1	0
17	4303	12	Azithromycin	0	54	6/13/2016	98.9	HEIGHT	15.4	16.5	1	0
17	4303	24	Azithromycin	0	67	4/25/2017	105.5	HEIGHT	16.72727273	16.5	0	0
17	4303	36	Azithromycin	0	80	5/16/2018	111	HEIGHT	18.9	16	0	0
17	4303	48	Azithromycin	0	91	4/22/2019	115.8	HEIGHT	20.15	15.5	0	0
17	4303	60	Azithromycin	0	100	2/11/2020	119.9	HEIGHT	23.3	17.5	0	0
17	4304	24	Azithromycin	0	3	4/25/2017	59.7	LENGTH	5.5	13	1	0
17	4304	36	Azithromycin	0	16	5/16/2018	74.7	LENGTH	8.25	13	1	0
17	4304	48	Azithromycin	0	27	4/22/2019	81.6	HEIGHT	10.25	14	1	0
17	4307	12	Azithromycin	0	26	6/13/2016	85.9	LENGTH	12.1	15.5	1	0
17	4307	24	Azithromycin	0	37	5/18/2017	94.1	HEIGHT	13.54545455	14.5	1	0
17	4307	48	Azithromycin	0	61	4/22/2019	107.6	HEIGHT	17.55	14.5	0	0
17	4310	0	Azithromycin	0	36	3/28/2015	100.1	HEIGHT	17.65	17	1	0
17	4310	12	Azithromycin	0	45	6/13/2016	108.5	HEIGHT	19.95	17.5	1	0
17	4310	24	Azithromycin	0	58	4/25/2017	114.9	HEIGHT	22.27272727	18	1	0
17	4310	36	Azithromycin	0	71	5/16/2018	120.5	HEIGHT	24.15	17.5	0	0
17	4310	48	Azithromycin	0	82	4/22/2019	124.6	HEIGHT	26.15	17	0	0
17	4310	60	Azithromycin	0	91	2/11/2020	127.9	HEIGHT	27.7	18.2	0	0
17	4313	24	Azithromycin	1	4	5/18/2017	59.7	LENGTH	7.090909091	14	1	0
17	4313	36	Azithromycin	1	17	5/16/2018	74.5	HEIGHT	9.6	15	1	0
17	4313	48	Azithromycin	1	27	4/22/2019	82.8	HEIGHT	11.45	14.5	1	0
17	4313	60	Azithromycin	1	37	2/11/2020	87.4	HEIGHT	13.1	15	1	0
17	4314	12	Azithromycin	1	7	6/13/2016	70.4	HEIGHT	7.3	13.5	1	0
17	4315	0	Azithromycin	0	24	3/28/2015	83.2	HEIGHT	11.3	15.5	1	0
17	4315	12	Azithromycin	0	33	6/13/2016	92	HEIGHT	12.7	15.5	1	0
17	4315	24	Azithromycin	0	46	4/25/2017	99	HEIGHT	14.40909091	15.5	1	0
17	4315	36	Azithromycin	0	59	5/16/2018	103.9	HEIGHT	14.9	14	1	0
17	4315	48	Azithromycin	0	70	4/22/2019	109.4	HEIGHT	17.2	14.5	0	0
17	4315	60	Azithromycin	0	79	2/11/2020	112.8	HEIGHT	19	16	0	0
17	4316	12	Azithromycin	1	8	6/13/2016	73.3	LENGTH	7.45	13	1	0

17	4316	48	Azithromycin	1	40	4/22/2019	96.9	HEIGHT	13.6	13.5	1	0
17	4316	60	Azithromycin	1	49	2/11/2020	103.4	HEIGHT	15.1	13.5	1	0
17	4318	24	Azithromycin	0	4	5/18/2017	60.6	LENGTH	6.909090909	13.5	1	0
17	4318	36	Azithromycin	0	17	5/16/2018	73.4	LENGTH	8.2	13	1	0
17	4318	48	Azithromycin	0	27	4/22/2019	82.5	HEIGHT	10.6	13	1	0
17	4318	60	Azithromycin	0	37	2/11/2020	90.1	HEIGHT	12.65	14.5	1	0
17	4319	0	Azithromycin	1	48	3/28/2015	106.6	HEIGHT	15.4	14.5	1	0
17	4319	12	Azithromycin	1	57	6/13/2016	112.8	HEIGHT	17.5	16.5	1	0
17	4319	24	Azithromycin	1	70	4/25/2017	117.7	HEIGHT	19.09090909	17	0	0
17	4319	36	Azithromycin	1	83	5/16/2018	123.4	HEIGHT	20.65	17	0	0
17	4319	48	Azithromycin	1	94	4/22/2019	126.1	HEIGHT	23.8	17	0	0
17	4319	60	Azithromycin	1	103	2/11/2020	128.5	HEIGHT	26.1	18.5	0	0
17	4321	24	Azithromycin	1	43	4/27/2017	90.3	HEIGHT	14.2	15	1	0
17	4321	36	Azithromycin	1	56	5/16/2018	97.7	HEIGHT	16.6	16	1	0
17	4322	12	Azithromycin	0	39	6/14/2016	104.7	HEIGHT	15.85	15.5	1	0
17	4322	24	Azithromycin	0	52	4/25/2017	110.6	HEIGHT	17.09090909	15.5	1	0
17	4323	0	Azithromycin	1	36	3/28/2015	105.1	HEIGHT	15.2	14.5	1	0
17	4323	12	Azithromycin	1	47	6/13/2016	111.3	HEIGHT	16.8	15.5	1	0
17	4323	24	Azithromycin	1	58	4/25/2017	115.7	HEIGHT	19	16	1	0
17	4323	36	Azithromycin	1	89	5/16/2018	119.6	HEIGHT	19.85	16	0	0
17	4323	48	Azithromycin	1	82	4/22/2019	122.8	HEIGHT	22.2	17	0	0
17	4324	0	Azithromycin	0	12	3/28/2015	66.5	LENGTH	7.35	14	1	0
17	4327	0	Azithromycin	1	12	3/28/2015	84.6	HEIGHT	10.95	14.5	1	0
17	4327	12	Azithromycin	1	23	6/13/2016	92.5	HEIGHT	12.55	15	1	0
17	4327	24	Azithromycin	1	34	4/25/2017	99.5	HEIGHT	13.95454545	15.5	1	0
17	4327	36	Azithromycin	1	47	5/16/2018	106.1	HEIGHT	15.35	15	1	0
17	4327	48	Azithromycin	1	58	4/22/2019	106.5	HEIGHT	17.1	15.5	1	0
17	4329	12	Azithromycin	0	57	6/13/2016	107.1	HEIGHT	17	16.5	1	0
17	4330	12	Azithromycin	1	39	6/13/2016	88.7	HEIGHT	10.9	13.5	1	0
17	4330	36	Azithromycin	1	58	5/16/2018	100.7	HEIGHT	14.45	14.5	1	0
17	4330	48	Azithromycin	1	57	4/22/2019	105.6	HEIGHT	14.8	14	1	0
17	4333	0	Azithromycin	0	36	3/28/2015	95.8	HEIGHT	14.85	16	1	0
17	4333	12	Azithromycin	0	47	6/13/2016	104.6	HEIGHT	17.35	16	1	0
17	4333	24	Azithromycin	0	58	4/25/2017	111.4	HEIGHT	18.68181818	15.5	1	0
17	4335	0	Azithromycin	1	54	3/28/2015	102.5	HEIGHT	13.85	13.5	1	0
17	4335	12	Azithromycin	1	54	6/13/2016	108.7	HEIGHT	16.6	15	1	0
17	4335	24	Azithromycin	1	67	4/25/2017	114.5	HEIGHT	19	15.5	0	0
17	4335	36	Azithromycin	1	80	5/16/2018	119.4	HEIGHT	20.3	15	0	0
17	4335	48	Azithromycin	1	91	4/22/2019	121.6	HEIGHT	21.5	15.5	0	0
17	4341	24	Azithromycin	1	3	4/25/2017	53.8	LENGTH	4.136363636	12	1	0

17	4341	36	Azithromycin	1	14	5/16/2018	72.2	HEIGHT	8.25	14	1	0
17	4341	48	Azithromycin	1	24	4/22/2019	81.3	LENGTH	9.75	13	1	0
17	4344	24	Azithromycin	0	58	4/27/2017	114.6	HEIGHT	19.45	15.5	1	0
17	4345	24	Azithromycin	0	4	5/18/2017	64.1	LENGTH	6.227272727	12.5	1	0
17	4345	48	Azithromycin	0	27	4/22/2019	88.8	HEIGHT	12.3	14.5	1	0
17	4349	0	Azithromycin	1	18	3/28/2015	84.2	HEIGHT	11.1	15	1	0
17	4349	36	Azithromycin	1	53	5/16/2018	109.1	HEIGHT	18	15.5	1	0
17	4349	60	Azithromycin	1	73	2/11/2020	119.5	HEIGHT	22.25	16.5	0	0
17	4351	12	Azithromycin	0	39	6/13/2016	93.9	HEIGHT	13.1	15	1	0
17	4354	24	Azithromycin	0	16	4/25/2017	72	HEIGHT	9.227272727	15	1	0
17	4354	36	Azithromycin	0	24	5/16/2018	83.5	HEIGHT	10.7	14	1	0
17	4354	48	Azithromycin	0	35	4/22/2019	89.6	HEIGHT	13.2	15.5	1	0
17	4354	60	Azithromycin	0	45	2/11/2020	97.6	HEIGHT	15	15.8	1	0
17	4356	24	Azithromycin	1	36	4/25/2017	90.7	HEIGHT	12.68181818	14.5	1	0
17	4356	36	Azithromycin	1	48	5/16/2018	97.5	HEIGHT	13.5	14	1	0
17	4356	48	Azithromycin	1	58	4/22/2019	101.9	HEIGHT	15	14.5	1	0
17	4357	24	Azithromycin	0	10	4/25/2017	75.9	HEIGHT	10.36363636	16	1	0
17	4357	36	Azithromycin	0	23	5/16/2018	87	HEIGHT	12.95	15	1	0
17	4357	48	Azithromycin	0	34	4/22/2019	95.2	HEIGHT	15.9	16.5	1	0
17	4360	0	Azithromycin	1	12	3/28/2015	66.7	LENGTH	6.35	13	1	0
17	4360	60	Azithromycin	1	67	2/11/2020	105.3	HEIGHT	12.95	13.2	0	0
17	4362	0	Azithromycin	0	-4	3/28/2015	82.4	HEIGHT	10.6	14.5	1	0
17	4362	36	Azithromycin	0	32	5/16/2018	83.7	HEIGHT	12.45	15	1	0
17	4363	24	Azithromycin	1	3	4/25/2017	57	LENGTH	4.590909091	12	1	0
17	4363	36	Azithromycin	1	16	5/16/2018	71.9	LENGTH	7.75	13.5	1	0
17	4363	48	Azithromycin	1	27	4/22/2019	80.5	LENGTH	9.5	14	1	0
17	4364	0	Azithromycin	1	36	3/28/2015	88.3	HEIGHT	11.4	14.5	1	0
17	4364	12	Azithromycin	1	42	6/13/2016	95.5	HEIGHT	13.3	14.5	1	0
17	4364	24	Azithromycin	1	55	4/25/2017	103.9	HEIGHT	15.5	14.5	1	0
17	4364	36	Azithromycin	1	68	5/16/2018	109.2	HEIGHT	17.75	14.5	0	0
17	4366	24	Azithromycin	1	52	4/25/2017	92.1	HEIGHT	12.77272727	14.5	1	0
17	4366	48	Azithromycin	1	76	4/22/2019	103.8	HEIGHT	15.2	14	0	0
17	4366	60	Azithromycin	1	85	2/11/2020	109.2	HEIGHT	16.95	14.5	0	0
17	4368	12	Azithromycin	1	9	6/13/2016	66.9	LENGTH	7.2	14.5	1	0
17	4368	24	Azithromycin	0	16	4/25/2017	72.2	LENGTH	7.272727273	13.5	1	0
17	4368	36	Azithromycin	0	33	5/16/2018	76.9	LENGTH	7.95	13.5	1	0
17	4368	48	Azithromycin	0	44	4/22/2019	80.5	LENGTH	8.6	13.5	1	0
17	4369	12	Azithromycin	1	6	6/13/2016	70.9	HEIGHT	8.65	14	1	0
17	8055	60	Azithromycin	1	3	2/11/2020	75.7	HEIGHT	8.65	14	1	0
17	8261	48	Azithromycin	0	4	4/22/2019	65.1	LENGTH	6.7	13	1	0

17	8339	48	Azithromycin	1	51	4/22/2019	93.8	HEIGHT	13.65	14	1	0
17	8339	60	Azithromycin	1	61	2/11/2020	98.6	HEIGHT	14.7	14.3	0	0
17	8376	60	Azithromycin	0	55	2/11/2020	97.9	HEIGHT	16.2	15.2	1	0
17	8534	36	Azithromycin	1	12	5/16/2018	69.6	LENGTH	7.65	13.5	1	0
17	8598	60	Azithromycin	1	4	2/11/2020	64.3	HEIGHT	7.05	14	1	0
17	8777	48	Azithromycin	0	72	4/22/2019	110.3	HEIGHT	17.2	15	0	0
17	8777	60	Azithromycin	0	81	2/11/2020	114.8	HEIGHT	18.75	15.2	0	0
17	8797	48	Azithromycin	1	8	4/22/2019	66.3	LENGTH	7.5	14.5	1	0
17	8797	60	Azithromycin	1	14	2/11/2020	76.3	HEIGHT	9.15	14.3	1	0
17	8870	60	Azithromycin	0	25	2/11/2020	84.7	HEIGHT	12	14	1	0
17	8946	36	Azithromycin	0	19	5/16/2018	76.5	HEIGHT	8.15	13	1	0
17	8946	48	Azithromycin	0	30	4/22/2019	81.2	HEIGHT	9.65	12.5	1	0
17	8961	48	Azithromycin	0	1	4/22/2019	56	LENGTH	5	12.5	1	0
17	8961	60	Azithromycin	0	10	2/11/2020	74.2	HEIGHT	8.8	14	1	0
17	9005	36	Azithromycin	1	15	5/16/2018	80.4	HEIGHT	10.3	14	1	0
17	9005	48	Azithromycin	1	25	4/22/2019	88	HEIGHT	13	15	1	0
17	9088	48	Azithromycin	0	2	4/22/2019	61.4	LENGTH	5.1	12	1	0
17	9088	60	Azithromycin	0	11	2/11/2020	75.2	LENGTH	9.65	13.5	1	0
17	9090	36	Azithromycin	0	15	5/16/2018	74.3	HEIGHT	9.2	14	1	0
17	9090	48	Azithromycin	0	25	4/22/2019	83.2	HEIGHT	13.2	14	1	0
17	9090	60	Azithromycin	0	35	2/11/2020	91.5	HEIGHT	14.55	15	1	0
17	9215	48	Azithromycin	0	7	4/22/2019	67.7	LENGTH	7.4	12.5	1	0
17	9215	60	Azithromycin	0	14	2/11/2020	78.7	LENGTH	10.8	14.2	1	0
17	9551	48	Azithromycin	0	39	4/22/2019	92.3	HEIGHT	13.4	14	1	0
17	9580	48	Azithromycin	0	18	4/22/2019	81.3	HEIGHT	10.45	14.5	1	0
17	9580	60	Azithromycin	0	27	2/11/2020	88.1	HEIGHT	12.45	14.1	1	0
18	4382	0	Azithromycin	0	48	3/31/2015	82.1	HEIGHT	9.35	13	1	0
18	4382	12	Azithromycin	0	46	7/22/2016	89.6	HEIGHT		13.5	0	0
18	4382	24	Azithromycin	0	54	4/6/2017	94.9	HEIGHT	11.7	13.5	0	0
18	4382	36	Azithromycin	0	68	5/25/2018	100.9	HEIGHT	13.45	13	0	0
18	4382	48	Azithromycin	0	79	4/20/2019	105.7	HEIGHT	15.5	13.5	0	0
18	4382	60	Azithromycin	0	88	2/27/2020	112.1	HEIGHT	16.4	15	0	0
18	4383	0	Azithromycin	1	36	3/31/2015	90.1	HEIGHT	10.35	13.5	1	0
18	4383	12	Azithromycin	1	49	7/22/2016	98.1	HEIGHT		13	0	0
18	4383	24	Azithromycin	1	61	4/6/2017	103.3	HEIGHT	12.5	11.5	0	0
18	4383	36	Azithromycin	1	75	5/25/2018	110.5	HEIGHT	13.65	13	0	0
18	4383	48	Azithromycin	1	85	4/20/2019	114.3	HEIGHT	15.3	12.5	0	0
18	4383	60	Azithromycin	1	95	2/27/2020	126	HEIGHT	17	14	0	0
18	4385	12	Azithromycin	0	18	7/23/2016	72.9	LENGTH		12	1	0
18	4387	0	Azithromycin	1	24	3/31/2015	81.9	LENGTH	9.65	14	0	0

18	4388	0	Azithromycin	0	36	3/31/2015	92.1	HEIGHT	12.2	14.5	1	0
18	4388	12	Azithromycin	0	54	7/22/2016	101.3	HEIGHT		14	0	0
18	4388	24	Azithromycin	0	67	4/6/2017	106.9	HEIGHT	14.9	14	0	0
18	4388	36	Azithromycin	0	80	5/25/2018	113.5	HEIGHT	16.72727273	14	0	0
18	4388	48	Azithromycin	0	91	4/20/2019	119	HEIGHT	18.6	15	0	0
18	4388	60	Azithromycin	0	100	2/27/2020	123.5	HEIGHT	21.4	15.4	0	0
18	4396	24	Azithromycin	1	24	4/6/2017	84.4	HEIGHT	13.35	15.5	1	0
18	4398	0	Azithromycin	1	5	6/13/2015	73.2	LENGTH	7.25	13	0	0
18	4399	0	Azithromycin	0	48	3/31/2015	114.6	HEIGHT	18.2	15	0	0
18	4399	36	Azithromycin	0	87	5/25/2018	128.9	HEIGHT	22.63636364	15	0	0
18	4400	0	Azithromycin	1	4	6/13/2015	66.1	LENGTH	8.65	15	0	0
18	4400	12	Azithromycin	1	12	7/22/2016	77.8	HEIGHT		15	0	0
18	4400	24	Azithromycin	1	24	4/6/2017	81.7	HEIGHT	11.95	15	0	0
18	4400	36	Azithromycin	1	43	5/25/2018	90.6	HEIGHT	13.22727273	15.5	0	0
18	4400	48	Azithromycin	1	54	4/20/2019	97.8	HEIGHT	14.9	15.5	0	0
18	4400	60	Azithromycin	1	64	2/27/2020	103.2	HEIGHT	16.1	15	0	0
18	4402	0	Azithromycin	1	18	3/31/2015	85	HEIGHT	10.95	14.5	1	0
18	4402	36	Azithromycin	1	57	5/25/2018	103.3	HEIGHT	13.72727273	13	0	0
18	4404	0	Azithromycin	1	24	3/31/2015	82.9	HEIGHT	10.15	13.5	0	0
18	4405	0	Azithromycin	0	36	3/31/2015	79.7	HEIGHT	10.05	13.5	1	0
18	4405	12	Azithromycin	0	48	7/22/2016	87.4	HEIGHT		13.5	1	0
18	4405	24	Azithromycin	0	60	4/6/2017	92.5	HEIGHT	12.15	13	1	0
18	4405	36	Azithromycin	0	73	5/25/2018	98.5	HEIGHT	13.13636364	14	0	0
18	4405	48	Azithromycin	0	84	4/20/2019	101.1	HEIGHT	14.45	13	0	0
18	4405	60	Azithromycin	0	94	2/27/2020	105.1	HEIGHT	15.8	15	0	0
18	4406	0	Azithromycin	1	12	3/31/2015	75.5	LENGTH	8.45	13	0	0
18	4406	12	Azithromycin	1	25	7/22/2016	87.7	HEIGHT		14	0	0
18	4406	24	Azithromycin	1	38	4/6/2017	93.8	HEIGHT	12.35	14.5	0	0
18	4406	36	Azithromycin	1	52	5/25/2018	102.5	HEIGHT	13.85	14	1	0
18	4406	48	Azithromycin	1	63	4/20/2019	107.5	HEIGHT	15.05	13.5	0	0
18	4406	60	Azithromycin	1	72	2/27/2020	113.6	HEIGHT	16.95	13.5	0	0
18	4407	12	Azithromycin	0	54	7/22/2016	95.2	HEIGHT		15	1	0
18	4409	12	Azithromycin	1	12	7/22/2016	68.3	HEIGHT		14	1	0
18	4409	24	Azithromycin	1	21	4/6/2017	72.9	HEIGHT	8.55	13.5	0	0
18	4409	36	Azithromycin	1	36	5/25/2018	81.1	HEIGHT	10.45	14	1	0
18	4409	48	Azithromycin	1	47	4/20/2019	87.8	HEIGHT	12.5	15	0	0
18	4409	60	Azithromycin	1	56	2/27/2020	93.2	HEIGHT	13.4	15.2	0	0
18	4410	0	Azithromycin	0	48	3/31/2015	96.2	HEIGHT	12.95	15	0	0
18	4410	12	Azithromycin	0	48	7/22/2016	104.3	HEIGHT		14	0	0
18	4410	24	Azithromycin	0	61	4/6/2017	108.3	HEIGHT	15.1	14	0	0

18	4410	36	Azithromycin	0	74	5/25/2018	114.3	HEIGHT	16.68181818	14.5		0	0
18	4410	48	Azithromycin	0	85	4/20/2019	118.4	HEIGHT	18.3	14.5		0	0
18	4410	60	Azithromycin	0	94	3/12/2020	122.2	HEIGHT	21	15.1		0	0
18	4411	0	Azithromycin	0	6	3/31/2015	68.1	LENGTH	7.05	13.5		1	0
18	4411	12	Azithromycin	0	18	7/22/2016	78.9	HEIGHT		15		1	0
18	4411	24	Azithromycin	0	30	4/6/2017	84.5	HEIGHT	11.4	15		0	0
18	4411	36	Azithromycin	0	44	5/25/2018	90.4	HEIGHT	12.25	15		1	0
18	4411	48	Azithromycin	0	55	4/20/2019	96.2	HEIGHT	13	15		1	0
18	4411	60	Azithromycin	0	64	2/27/2020	103	HEIGHT	14.65	14.5		0	0
18	4412	0	Azithromycin	1	18	3/31/2015	76.2	HEIGHT	8.05	12.5		0	0
18	4412	12	Azithromycin	1	26	7/22/2016	86	HEIGHT		13.5		0	0
18	4415	0	Azithromycin	1	48	3/31/2015	92.5	HEIGHT	12.35	13.5		1	0
18	4415	12	Azithromycin	1	58	7/22/2016	103.8	HEIGHT		15		0	0
18	4415	24	Azithromycin	1	67	4/7/2017	110.2	HEIGHT	17.3	15		0	0
18	4415	48	Azithromycin	1	91	4/20/2019	120.6	HEIGHT	20.95	15		0	0
18	4415	60	Azithromycin	1	100	2/27/2020	126.6	HEIGHT	23.85	16.4		0	0
18	4416	0	Azithromycin	0	30	3/31/2015	92.7	HEIGHT	13.8	15.5		1	0
18	4416	12	Azithromycin	0	42	7/22/2016	101.3	HEIGHT		16.5		0	0
18	4417	0	Azithromycin	0	48	3/31/2015	100.5	HEIGHT	12.8	12.5		0	0
18	4417	12	Azithromycin	0	61	7/22/2016	109.1	HEIGHT		13		0	0
18	4417	36	Azithromycin	0	85	5/25/2018	119.5	HEIGHT	18.72727273	14		0	0
18	4417	48	Azithromycin	0	96	4/20/2019	123.3	HEIGHT	19.85	13.5		0	0
18	4426	12	Azithromycin	1	42	7/22/2016	98.5	HEIGHT		14		1	0
18	4429	0	Azithromycin	1	12	3/31/2015	74.2	LENGTH	7.7	12.5		0	0
18	4429	12	Azithromycin	1	30	7/22/2016	88	HEIGHT		13		0	0
18	4429	24	Azithromycin	1	42	4/6/2017	94.5	HEIGHT	11.9	14		0	0
18	4429	36	Azithromycin	1	56	5/25/2018	105.7	HEIGHT	13.90909091	14		0	0
18	4429	48	Azithromycin	1	56	4/20/2019	114.8	HEIGHT	15.2	13.5		1	0
18	4432	0	Azithromycin	1	5	3/31/2015	60.6	LENGTH	5.7	13		1	0
18	4432	36	Azithromycin	1	43	5/25/2018	92	HEIGHT	11.27272727	13.5		1	0
18	4433	0	Azithromycin	1	36	3/31/2015	84.4	HEIGHT	10.9	15		1	0
18	4433	12	Azithromycin	1	47	7/22/2016	93.6	HEIGHT		14.5		1	0
18	4433	24	Azithromycin	1	56	4/6/2017	98.3	HEIGHT	12	13.5		1	0
18	4433	48	Azithromycin	1	83	4/20/2019	108.6	HEIGHT	14.5	13.5		0	0
18	4433	60	Azithromycin	1	93	2/27/2020	114	HEIGHT	16.7	14.2		0	0
18	4434	0	Azithromycin	0	24	3/31/2015	87.2	HEIGHT	12.65	16.5		0	0
18	4434	12	Azithromycin	0	30	7/22/2016	96.4	HEIGHT		15.5		0	0
18	4434	24	Azithromycin	0	42	4/6/2017	102.1	HEIGHT	15.75	16		0	0
18	4434	36	Azithromycin	0	56	5/25/2018	110.4	HEIGHT	18.31818182	15.5		0	0
18	4436	0	Azithromycin	0	48	3/31/2015	81.8	HEIGHT	9	13		0	0

18	4436	12	Azithromycin	0	46	7/22/2016	89.1	HEIGHT		14		0	0
18	4436	24	Azithromycin	0	54	4/6/2017	94.6	HEIGHT	11.5	13.5		1	0
18	4436	36	Azithromycin	0	68	5/25/2018	101.4	HEIGHT	13	13		0	0
18	4436	48	Azithromycin	0	79	4/20/2019	105.8	HEIGHT	14.4	13.5		0	0
18	4436	60	Azithromycin	0	88	2/27/2020	110.8	HEIGHT	15.7	13		0	0
18	4437	0	Azithromycin	1	24	3/31/2015	83.5	HEIGHT	9.1	13		0	0
18	4438	0	Azithromycin	1	36	3/31/2015	84	HEIGHT	9	12.5		1	0
18	4438	12	Azithromycin	1	54	7/22/2016	91.9	HEIGHT		12.5		0	0
18	4438	24	Azithromycin	1	66	4/7/2017	97.6	HEIGHT	10.95	12		0	0
18	4438	36	Azithromycin	1	80	5/25/2018	104.9	HEIGHT	12.90909091	12.5		0	0
18	4438	48	Azithromycin	1	91	4/20/2019	109.4	HEIGHT	13.9	12.5		0	0
18	4439	0	Azithromycin	0	24	3/31/2015	82.2	HEIGHT	9.45	14		1	0
18	4439	12	Azithromycin	0	37	7/22/2016	92	HEIGHT		13.5		0	0
18	4439	24	Azithromycin	0	49	4/7/2017	97.7	HEIGHT	13.55	14		0	0
18	4444	0	Azithromycin	1	10	3/31/2015	72.1	LENGTH	7.75	13.5		0	0
18	4444	24	Azithromycin	1	32	4/7/2017	90.6	HEIGHT	11.05	13.5		0	0
18	4444	36	Azithromycin	1	46	5/25/2018	100.6	HEIGHT	13.45	14.5		0	0
18	4444	48	Azithromycin	1	56	4/20/2019	105.4	HEIGHT	14.75	14		1	0
18	4445	24	Azithromycin	1	1	4/6/2017	51.4	LENGTH	3.9	11		1	0
18	4445	36	Azithromycin	1	11	5/25/2018	73	LENGTH	7.8	13.5		0	0
18	4445	48	Azithromycin	1	22	4/20/2019	80.1	HEIGHT	9.5	13.5		0	0
18	4445	60	Azithromycin	1	32	2/27/2020	88.1	HEIGHT	12	15.2		0	0
18	4446	12	Azithromycin	0	38	7/22/2016	97.4	HEIGHT		13.5		1	0
18	4446	24	Azithromycin	0	50	4/7/2017	101.7	HEIGHT	13	13.5		1	0
18	4448	0	Azithromycin	0	24	3/31/2015	83.7	HEIGHT	9.4	12.5		0	0
18	4448	36	Azithromycin	1	42	5/25/2018	122.5	HEIGHT	20.13636364	14.5		0	0
18	4448	48	Azithromycin	1	111	4/20/2019	125.7	HEIGHT	22.25	15.5		0	0
18	4448	60	Azithromycin	1	121	3/6/2020	127.9	HEIGHT	23	15		0	0
18	4449	0	Azithromycin	0	24	3/31/2015	88.9	HEIGHT	10.9	13.5	18	1	0
18	4453	12	Azithromycin	1	3	7/22/2016	63.4	LENGTH		13		1	0
18	4455	0	Azithromycin	0	24	3/31/2015	81.9	HEIGHT	9.75	13		1	0
18	4456	0	Azithromycin	1	36	6/13/2015	93.6	HEIGHT	13.8	15		1	0
18	4457	12	Azithromycin	1	36	7/22/2016	97.7	HEIGHT		14		1	0
18	4457	24	Azithromycin	1	49	4/6/2017	104	HEIGHT	13.7	13.5		1	0
18	4459	12	Azithromycin	0	-3	7/22/2016	58.9	LENGTH		13.5		0	0
18	4459	24	Azithromycin	0	10	4/6/2017	72.2	LENGTH	8.9	16		0	0
18	4459	36	Azithromycin	0	21	5/25/2018	85.8	HEIGHT	10.54545455	14		0	0
18	4460	12	Azithromycin	1	9	7/22/2016	71.8	HEIGHT		13		0	0
18	4460	24	Azithromycin	1	21	4/6/2017	81.1	HEIGHT	10.5	15		0	0
18	4460	36	Azithromycin	1	39	5/25/2018	90.8	HEIGHT	13.45454545	15		1	0

18	4460	60	Azithromycin	1	59	3/6/2020	104	HEIGHT	16.5	14.9		0	0
18	4461	12	Azithromycin	1	50	7/22/2016	107.9	HEIGHT		13.5		1	0
18	4464	0	Azithromycin	0	12	3/31/2015	61.2	LENGTH	5.7	11.5	24	1	0
18	4473	0	Azithromycin	0	48	3/31/2015	96.7	HEIGHT	13.95	14		1	0
18	4473	24	Azithromycin	0	66	4/6/2017	107.8	HEIGHT	17.1	14.5		0	0
18	4473	36	Azithromycin	0	80	5/25/2018	113.3	HEIGHT	17.63636364	15		0	0
18	4473	48	Azithromycin	0	91	4/20/2019	116.6	HEIGHT	19.25	14.5		0	0
18	4474	0	Azithromycin	1	36	3/31/2015	90.7	HEIGHT	12.15	14		0	0
18	4474	12	Azithromycin	1	39	7/22/2016	103	HEIGHT		15		0	0
18	4474	24	Azithromycin	1	49	4/6/2017	108.5	HEIGHT	16.15	14.5		1	0
18	4474	48	Azithromycin	1	73	4/20/2019	121	HEIGHT	20.4	15		0	0
18	4475	0	Azithromycin	0	24	3/31/2015	79.1	LENGTH	8.6	13		1	0
18	4476	0	Azithromycin	1	24	3/31/2015	77.4	HEIGHT	9.05	14.5		1	0
18	4476	24	Azithromycin	1	49	4/6/2017	92.4	HEIGHT	12.55	15		0	0
18	4478	0	Azithromycin	1	48	3/31/2015	86.7	HEIGHT	10.2	14		0	0
18	4485	12	Azithromycin	1	46	7/22/2016	98.4	HEIGHT		12.5		1	0
18	4487	0	Azithromycin	1	24	3/31/2015	82.9	HEIGHT	10.1	14		1	0
18	4487	12	Azithromycin	1	36	7/22/2016	91.7	HEIGHT		14.5		0	0
18	4487	48	Azithromycin	1	69	4/20/2019	109.1	HEIGHT	16.45	15		0	0
18	4487	60	Azithromycin	1	78	3/6/2020	114.5	HEIGHT	17.55	15.6		0	0
18	4492	24	Azithromycin	1	37	4/6/2017	98.2	HEIGHT	12.55	14		1	0
18	4492	36	Azithromycin	1	50	5/25/2018	105.6	HEIGHT	14.25	13.5		1	0
18	4493	12	Azithromycin	1	9	7/22/2016	68.9	LENGTH		13.5		0	0
18	4493	60	Azithromycin	1	48	3/6/2020	103	HEIGHT	14.8	15.5		1	0
18	4494	0	Azithromycin	0	48	3/31/2015	92.9	HEIGHT	12.55	14		0	0
18	4494	12	Azithromycin	0	48	7/22/2016	103.5	HEIGHT		14		0	0
18	4494	36	Azithromycin	0	74	5/25/2018	111.7	HEIGHT	16.8	14		0	0
18	4494	48	Azithromycin	0	85	4/20/2019	116.4	HEIGHT	19.9	16.5		0	0
18	4494	60	Azithromycin	0	94	2/27/2020	121.5	HEIGHT	22.15	16.5		0	0
18	4497	24	Azithromycin	1	7	4/6/2017	68	LENGTH	7.5	15		1	0
18	4497	36	Azithromycin	1	15	5/25/2018	80.8	HEIGHT	9.272727273	14		0	0
18	4503	12	Azithromycin	0	7	7/23/2016	68.9	LENGTH		13.5		0	0
18	4504	0	Azithromycin	1	24	3/31/2015	80.4	LENGTH	7.75	11.5		0	0
18	4504	12	Azithromycin	1	30	7/22/2016	88.7	HEIGHT		12		0	0
18	4504	36	Azithromycin	1	56	5/25/2018	103.7	HEIGHT	12.36363636	13		0	0
18	4504	48	Azithromycin	1	67	4/24/2019	109.4	HEIGHT	13.65	13		0	0
18	4505	12	Azithromycin	1	5	7/22/2016	72.4	LENGTH		12.5		1	0
18	4506	0	Azithromycin	0	8	3/31/2015	70.6	LENGTH	6.75	12		1	0
18	4506	12	Azithromycin	0	18	7/23/2016	82.3	HEIGHT		11.5		1	0
18	4506	24	Azithromycin	0	35	4/6/2017	87	HEIGHT	10.35	13		1	0

18	4506	36	Azithromycin	0	49	5/25/2018	94.3	HEIGHT	11.36363636	12.5	1	0
18	4506	60	Azithromycin	0	69	3/6/2020	107.4	HEIGHT	14.4	13	0	0
18	4507	0	Azithromycin	1	5	3/31/2015	60.8	LENGTH	4.75	10.5	1	0
18	4507	12	Azithromycin	1	18	7/22/2016	76.6	HEIGHT		12.5	1	0
18	4507	24	Azithromycin	1	33	4/6/2017	83	HEIGHT	8.7	12.5	1	0
18	4507	36	Azithromycin	1	45	5/25/2018	93	HEIGHT	10.95454545	12.5	0	0
18	4507	48	Azithromycin	1	55	4/20/2019	98.9	HEIGHT	11.4	12	1	0
18	4507	60	Azithromycin	1	65	2/27/2020	105.6	HEIGHT	12.9	11.8	0	0
18	4508	12	Azithromycin	1	24	7/22/2016	84	HEIGHT		13.5	1	0
18	4509	0	Azithromycin	0	48	3/31/2015	90.3	HEIGHT	11.25	13	0	0
18	4510	24	Azithromycin	1	43	4/6/2017	101.4	HEIGHT	16.1	16	1	0
18	4511	0	Azithromycin	0	36	3/31/2015	80.2	HEIGHT	10.1	13.5	1	0
18	4511	24	Azithromycin	0	55	4/6/2017	95.5	HEIGHT	13.45	14	1	0
18	4511	48	Azithromycin	0	79	4/20/2019	104.9	HEIGHT	15.75	13.5	0	0
18	4511	60	Azithromycin	0	89	2/27/2020	107.9	HEIGHT	17.35	14.5	0	0
18	4512	0	Azithromycin	0	36	6/13/2015	83.3	HEIGHT	10.25	14	1	0
18	4512	12	Azithromycin	0	36	7/22/2016	91.2	HEIGHT		14	0	0
18	4512	24	Azithromycin	0	49	4/6/2017	96.6	HEIGHT	14.65	15.5	0	0
18	4512	36	Azithromycin	0	62	5/25/2018	105.7	HEIGHT	16	14.5	0	0
18	4512	48	Azithromycin	0	73	4/20/2019	110	HEIGHT	16.9	15	0	0
18	4512	60	Azithromycin	0	82	2/27/2020	116.8	HEIGHT	19.5	14.7	0	0
18	4513	0	Azithromycin	0	48	3/31/2015	92.2	HEIGHT	13	15	1	0
18	4516	0	Azithromycin	0	36	3/31/2015	87.2	HEIGHT	11.5	13	0	0
18	4516	12	Azithromycin	0	53	7/22/2016	97.2	HEIGHT		15	0	0
18	4516	60	Azithromycin	0	95	2/27/2020	121.5	HEIGHT	21.45	15.5	0	0
18	4518	0	Azithromycin	1	24	3/31/2015	79.2	HEIGHT	10.2	14	0	0
18	4518	12	Azithromycin	1	45	7/22/2016	90	HEIGHT		14.5	1	0
18	4519	12	Azithromycin	0	38	7/23/2016	93	HEIGHT		16.5	1	0
18	4519	24	Azithromycin	0	50	4/6/2017	98.2	HEIGHT	14.65	16.5	1	0
18	4521	24	Azithromycin	1	31	4/6/2017	92.9	HEIGHT	10.8	13.5	1	0
18	4522	0	Azithromycin	1	12	3/31/2015	66.8	LENGTH	6.45	13	0	0
18	4526	0	Azithromycin	0	24	3/31/2015	75	HEIGHT	7.4	12.5	1	0
18	4526	12	Azithromycin	0	24	7/22/2016	83.7	HEIGHT		13	0	0
18	4526	24	Azithromycin	0	36	4/6/2017	90.7	HEIGHT	11.5	14.5	0	0
18	4526	36	Azithromycin	0	49	5/25/2018	100.3	HEIGHT	13.18181818	14.5	1	0
18	4526	48	Azithromycin	0	60	4/20/2019	106.2	HEIGHT	14.45	14	0	0
18	4526	60	Azithromycin	0	70	2/27/2020	111.3	HEIGHT	15.85	13.7	0	0
18	4531	0	Azithromycin	1	48	3/31/2015	103.6	HEIGHT	13.65	13	1	0
18	4531	48	Azithromycin	1	97	4/24/2019	121.3	HEIGHT	18.9	14.5	0	0
18	4535	0	Azithromycin	1	9	3/31/2015	68.8	LENGTH	7.95	14.5	1	0

18	4535	12	Azithromycin	1	14	7/22/2016	79.4	HEIGHT		14	0	0
18	4535	36	Azithromycin	1	40	5/25/2018	96.8	HEIGHT	13.27272727	14.5	1	0
18	4535	48	Azithromycin	1	51	4/24/2019	102.3	HEIGHT	14.5	14	1	0
18	4535	60	Azithromycin	1	60	2/27/2020	99.5	HEIGHT	15.15	15.7	0	0
18	4538	24	Azithromycin	0	20	4/7/2017	81.5	HEIGHT	9.35	13	1	0
18	4538	48	Azithromycin	0	44	4/20/2019	96.8	HEIGHT	13.35	13.5	1	0
18	4538	60	Azithromycin	0	54	3/6/2020	102.7	HEIGHT	14.95	14.3	1	0
18	4540	12	Azithromycin	0	3	7/22/2016	65.8	LENGTH		13	1	0
18	4540	24	Azithromycin	0	12	4/6/2017	76.5	HEIGHT	8.6	14	0	0
18	4540	36	Azithromycin	0	27	5/25/2018	86.9	HEIGHT	11.18181818	14	0	0
18	4540	48	Azithromycin	0	37	4/20/2019	94.6	HEIGHT	12.3	14	0	0
18	4540	60	Azithromycin	0	47	2/27/2020	101.6	HEIGHT	14.8	14.5	1	0
18	4542	24	Azithromycin	1	30	4/6/2017	86	HEIGHT	11.5	15	1	0
18	4542	36	Azithromycin	1	44	5/25/2018	94.6	HEIGHT	13.09090909	15	1	0
18	4542	48	Azithromycin	1	55	4/20/2019	106.4	HEIGHT	15.95	14	0	0
18	4542	60	Azithromycin	1	68	2/27/2020	110	HEIGHT	18.43	14.5	0	0
18	4543	0	Azithromycin	1	12	3/31/2015	61.3	LENGTH	5.4	11.5	0	0
18	4543	12	Azithromycin	1	24	7/22/2016	75.7	LENGTH		13	1	0
18	4543	24	Azithromycin	1	36	4/6/2017	81.7	HEIGHT	9.95	14	0	0
18	4543	48	Azithromycin	1	60	4/20/2019	94.4	HEIGHT	12.2	14	0	0
18	4543	60	Azithromycin	1	70	2/27/2020	99.1	HEIGHT	13.45	14.1	0	0
18	4544	0	Azithromycin	1	48	3/31/2015	100.9	HEIGHT	12.25	12.5	0	0
18	4544	12	Azithromycin	1	54	7/22/2016	107.2	HEIGHT		13	0	0
18	4544	24	Azithromycin	1	66	4/6/2017	112.2	HEIGHT	14.45	13	0	0
18	4544	36	Azithromycin	1	80	5/25/2018	117.6	HEIGHT	16	13.5	0	0
18	4544	48	Azithromycin	1	91	4/20/2019	120.3	HEIGHT	16.7	13	0	0
18	4546	0	Azithromycin	0	24	3/31/2015	76.9	HEIGHT	9.2	13.5	0	0
18	4548	24	Azithromycin	1	42	4/6/2017	92.5	HEIGHT	10.15	12.5	1	0
18	4549	0	Azithromycin	1	36	3/31/2015	117.8	HEIGHT	18.25	15	1	0
18	4549	12	Azithromycin	1	66	7/22/2016	121.5	HEIGHT		16	0	0
18	4549	24	Azithromycin	1	79	4/6/2017	123.9	HEIGHT	19.45	15	0	0
18	4549	36	Azithromycin	1	92	5/25/2018	127.6	HEIGHT	23.81818182	16.5	0	0
18	4549	48	Azithromycin	1	103	4/20/2019	130.9	HEIGHT	23.7	16.5	0	0
18	4549	60	Azithromycin	1	112	3/6/2020	132.7	HEIGHT	25.65	17.4	0	0
18	4551	24	Azithromycin	0	10	4/6/2017	62.5	LENGTH	4.35	9.5	1	0
18	4552	0	Azithromycin	1	24	3/31/2015	82	HEIGHT	10.65	14	1	0
18	4552	12	Azithromycin	1	36	7/22/2016	90.5	HEIGHT		14.5	0	0
18	4552	24	Azithromycin	1	44	4/6/2017	95.2	HEIGHT	13.65	14	0	0
18	4552	36	Azithromycin	1	72	5/25/2018	101.9	HEIGHT	15.13636364	14	0	0
18	4553	0	Azithromycin	0	24	3/31/2015	67.3	LENGTH	6.65	14	0	0

18	4554	0	Azithromycin	1	48	3/31/2015	98.9	HEIGHT	14.7	16	1	0
18	4554	12	Azithromycin	1	60	7/22/2016	106.4	HEIGHT		16.5	0	0
18	4555	12	Azithromycin	1	48	7/22/2016	94.3	HEIGHT		14	1	0
18	4555	24	Azithromycin	1	60	4/6/2017	99.5	HEIGHT	14.05	14.5	1	0
18	4555	36	Azithromycin	1	73	5/25/2018	108.3	HEIGHT	15.95454545	14	0	0
18	4555	48	Azithromycin	1	84	4/24/2019	113	HEIGHT	18.15	14.5	0	0
18	4555	60	Azithromycin	1	94	2/27/2020	113.9	HEIGHT	16	13.5	0	0
18	4556	0	Azithromycin	0	8	3/31/2015	72	LENGTH	7.75	14	0	0
18	4556	12	Azithromycin	0	18	7/23/2016	86.3	HEIGHT		13	0	0
18	4556	24	Azithromycin	0	35	4/6/2017	90.7	HEIGHT	11.6	14	1	0
18	4556	36	Azithromycin	0	49	5/25/2018	99.3	HEIGHT	12.95454545	13.5	1	0
18	4556	60	Azithromycin	0	69	3/6/2020	112	HEIGHT	16.9	14.2	0	0
18	4557	0	Azithromycin	1	48	3/31/2015	87	HEIGHT	10.8	14.5	1	0
18	4557	12	Azithromycin	1	56	7/22/2016	96.7	HEIGHT		14.5	1	0
18	4557	48	Azithromycin	1	81	4/20/2019	114.4	HEIGHT	17.4	14	0	0
18	4557	60	Azithromycin	1	91	2/27/2020	118.5	HEIGHT	19.25	16	0	0
18	4558	0	Azithromycin	0	30	3/31/2015	83.6	HEIGHT	9.75	12	0	0
18	4558	24	Azithromycin	0	55	4/7/2017	98.2	HEIGHT	15.7	16	0	0
18	4558	60	Azithromycin	0	89	2/27/2020	111.1	HEIGHT	18.25	14.4	0	0
18	4561	0	Azithromycin	0	48	3/31/2015	98.3	HEIGHT	13.4	14.5	1	0
18	4561	24	Azithromycin	0	73	4/6/2017	109.4	HEIGHT	16.6	14.5	0	0
18	4562	24	Azithromycin	1	14	4/6/2017	55.3	LENGTH	4.35	10	1	0
18	4566	24	Azithromycin	1	9	4/7/2017	72.9	LENGTH	10.1	15	1	0
18	4567	0	Azithromycin	0	10	3/31/2015	70.6	LENGTH	7	13.5	0	0
18	4567	12	Azithromycin	0	24	7/22/2016	82	HEIGHT		14	0	0
18	4567	24	Azithromycin	0	37	4/6/2017	87.9	HEIGHT	10.2	14	0	0
18	4567	36	Azithromycin	0	50	5/25/2018	94.5	HEIGHT	11.5	13.5	1	0
18	4570	0	Azithromycin	0	18	3/31/2015	75.9	HEIGHT	8.95	13.5	0	0
18	4571	12	Azithromycin	0	7	7/22/2016	71.5	LENGTH		12	1	0
18	4571	24	Azithromycin	0	15	4/6/2017	80.3	HEIGHT	10.3	13.5	0	0
18	4571	36	Azithromycin	0	28	5/25/2018	92	HEIGHT	13.40909091	15.5	1	0
18	4571	48	Azithromycin	0	39	4/20/2019	97.5	HEIGHT	14.65	14	0	0
18	4571	60	Azithromycin	0	48	2/27/2020	105.1	HEIGHT	17.15	15.2	0	0
18	4574	0	Azithromycin	0	5	3/31/2015	63.9	LENGTH	6.55	13.5	0	0
18	4574	12	Azithromycin	0	22	7/22/2016	76.2	HEIGHT		14.5	1	0
18	4574	24	Azithromycin	0	31	4/6/2017	81	HEIGHT	9.8	13.5	0	0
18	4574	36	Azithromycin	0	45	5/25/2018	87.3	HEIGHT	11.15	14.5	0	0
18	4574	48	Azithromycin	0	55	4/20/2019	92.2	HEIGHT	12.35	14	0	0
18	4574	60	Azithromycin	0	58	2/27/2020	99.7	HEIGHT	13.75	14	1	0
18	4575	24	Azithromycin	1	55	4/6/2017	95.1	HEIGHT	9.9	11.5	1	0

18	4575	60	Azithromycin	1	88	3/6/2020	107.7	HEIGHT	12.55	12	0	0
18	4577	24	Azithromycin	1	36	4/6/2017	91.7	HEIGHT	12.5	15	1	0
18	4579	12	Azithromycin	1	18	7/22/2016	78.7	HEIGHT		12.5	1	0
18	4582	0	Azithromycin	0	12	3/31/2015	65.3	LENGTH	6.2	11.5	0	0
18	4582	12	Azithromycin	0	18	7/22/2016	77.5	HEIGHT		12	0	0
18	4582	24	Azithromycin	0	30	4/6/2017	82	HEIGHT	9.55	13.5	1	0
18	4582	36	Azithromycin	0	44	5/25/2018	89.8	HEIGHT	10.63636364	12.5	1	0
18	4582	48	Azithromycin	0	55	4/20/2019	96.5	HEIGHT	12.35	13	0	0
18	4583	0	Azithromycin	1	36	3/31/2015	85.2	HEIGHT	10	13.5	0	0
18	4583	12	Azithromycin	1	42	7/22/2016	95.2	HEIGHT		12.5	0	0
18	4583	24	Azithromycin	1	54	4/6/2017	100.1	HEIGHT	12.25	13	0	0
18	4583	36	Azithromycin	1	68	5/25/2018	106.9	HEIGHT	13.27272727	13	0	0
18	4583	48	Azithromycin	1	79	4/20/2019	112.1	HEIGHT	14.4	12.5	0	0
18	4583	60	Azithromycin	1	88	3/12/2020	116.9	HEIGHT	16.2	14.6	0	0
18	4586	0	Azithromycin	1	36	3/31/2015	85.6	HEIGHT	10.8	13.5	0	0
18	4586	12	Azithromycin	1	42	7/22/2016	91.8	HEIGHT		13	0	0
18	4586	24	Azithromycin	1	50	4/6/2017	101.2	HEIGHT	13.7	14	0	0
18	4586	48	Azithromycin	1	75	4/20/2019	112.4	HEIGHT	16.95	14	0	0
18	4586	60	Azithromycin	1	84	2/27/2020	117	HEIGHT	18.65	15	0	0
18	4587	12	Azithromycin	1	10	7/23/2016	67.9	LENGTH		11	1	0
18	4587	24	Azithromycin	1	15	4/6/2017	80	HEIGHT	8.55	13.5	0	0
18	4588	0	Azithromycin	0	36	3/31/2015	94.5	HEIGHT	13.1	15	0	0
18	4588	12	Azithromycin	0	42	7/22/2016	106.8	HEIGHT		15.5	0	0
18	4588	24	Azithromycin	0	55	4/6/2017	109.8	HEIGHT	16.4	15	1	0
18	4588	36	Azithromycin	0	68	5/25/2018	116.7	HEIGHT	17.8	15	0	0
18	4588	48	Azithromycin	0	79	4/20/2019	120.3	HEIGHT	19.35	14.5	0	0
18	4588	60	Azithromycin	0	88	2/27/2020	126.1	HEIGHT	22.45	16	0	0
18	4589	0	Azithromycin	1	36	3/31/2015	84.7	HEIGHT	9.3	12	1	0
18	4589	36	Azithromycin	1	70	5/25/2018	116.6	HEIGHT	19	16	0	0
18	4589	48	Azithromycin	1	81	4/20/2019	120.9	HEIGHT	20.3	16.5	0	0
18	4589	60	Azithromycin	1	90	2/27/2020	125.4	HEIGHT	21.45	16	0	0
18	4590	24	Azithromycin	0	43	4/7/2017	96.3	HEIGHT	13.15	13.5	1	0
18	4590	36	Azithromycin	0	56	6/11/2018	104.3	HEIGHT	14.6	14	1	0
18	4591	0	Azithromycin	0	8	3/31/2015	67.7	LENGTH	7.1	13	0	0
18	4592	0	Azithromycin	0	36	3/31/2015	87.8	HEIGHT	10.8	13	1	0
18	4593	24	Azithromycin	0	3	4/6/2017	69.1	LENGTH	6.6	13	1	0
18	4593	36	Azithromycin	0	27	5/25/2018	79.3	LENGTH	8.045454545	12.5	1	0
18	4593	48	Azithromycin	0	37	4/20/2019	87.9	HEIGHT	9.45	13	0	0
18	4593	60	Azithromycin	0	47	3/12/2020	93.9	HEIGHT	11.6	14	0	0
18	4594	0	Azithromycin	0	36	3/31/2015	85.4	HEIGHT	10.65	14	0	0

18	4594	24	Azithromycin	0	67	4/6/2017	101.9	HEIGHT	15.25	15	0	0
18	4594	36	Azithromycin	0	80	5/25/2018	108.9	HEIGHT	15.72727273	14.5	0	0
18	4594	60	Azithromycin	0	101	2/27/2020	119	HEIGHT	19.4	14	0	0
18	4596	0	Azithromycin	0	42	3/31/2015	100.7	HEIGHT	14.85	15	1	0
18	4596	12	Azithromycin	0	50	7/23/2016	107.3	HEIGHT		14	1	0
18	4596	24	Azithromycin	0	62	4/6/2017	112.7	HEIGHT	17.25	15.5	0	0
18	4596	36	Azithromycin	0	76	5/25/2018	118.1	HEIGHT	19.59090909	15.5	0	0
18	4599	0	Azithromycin	1	12	6/13/2015	70.6	LENGTH	8.2	15	0	0
18	4599	12	Azithromycin	1	18	7/22/2016	82.7	HEIGHT		13.5	0	0
18	4599	24	Azithromycin	1	31	4/6/2017	88.3	HEIGHT	11	14.5	1	0
18	4599	36	Azithromycin	1	44	5/25/2018	97	HEIGHT	13.35	15	1	0
18	4599	48	Azithromycin	1	55	4/20/2019	103.8	HEIGHT	15.15	15	0	0
18	4599	60	Azithromycin	1	64	2/27/2020	110.8	HEIGHT	16.95	16.5	0	0
18	4600	12	Azithromycin	0	-3	7/22/2016	52.5	LENGTH		11	0	0
18	4600	24	Azithromycin	0	9	4/6/2017	70.6	LENGTH	6.85	13	1	0
18	4600	48	Azithromycin	0	33	4/20/2019	87.3	HEIGHT	11	13.5	0	0
18	4600	60	Azithromycin	0	43	2/27/2020	95	HEIGHT	13.45	14	0	0
18	4602	0	Azithromycin	0	8	3/31/2015	68.8	LENGTH	6.75	13.5	0	0
18	4602	12	Azithromycin	0	18	7/22/2016	79.5	HEIGHT		13	0	0
18	4602	24	Azithromycin	0	30	4/6/2017	84.5	HEIGHT	9.15	13	0	0
18	4602	36	Azithromycin	0	44	5/25/2018	92	HEIGHT	10.27272727	12	0	0
18	4602	48	Azithromycin	0	55	4/20/2019	97.6	HEIGHT	11.2	12.5	0	0
18	4602	60	Azithromycin	0	58	3/12/2020	105.3	HEIGHT	13.55	13.3	0	0
18	4606	0	Azithromycin	1	52	3/31/2015	97.4	HEIGHT	13.85	14	1	0
18	4606	24	Azithromycin	1	67	4/6/2017	106.8	HEIGHT	14.85	14	0	0
18	4606	36	Azithromycin	1	80	5/25/2018	113.8	HEIGHT	17.55	14	0	0
18	4606	48	Azithromycin	1	91	4/20/2019	116.5	HEIGHT	18.4	15	0	0
18	4606	60	Azithromycin	1	100	2/27/2020	121.1	HEIGHT	20.3	14.5	0	0
18	4607	24	Azithromycin	1	54	4/6/2017	97.3	HEIGHT	13.4	14.5	1	0
18	4608	12	Azithromycin	0	48	7/23/2016	100	HEIGHT		13	1	0
18	4609	0	Azithromycin	0	48	3/31/2015	99	HEIGHT	12.95	13.5	0	0
18	4610	0	Azithromycin	1	42	3/31/2015	100.1	HEIGHT	13.1	13.5	0	0
18	4612	12	Azithromycin	1	12	7/23/2016	72	HEIGHT		12	1	0
18	4612	24	Azithromycin	1	24	4/6/2017	77.4	HEIGHT	8.15	12.5	0	0
18	4612	36	Azithromycin	1	37	5/25/2018	87	HEIGHT	9.636363636	12.5	1	0
18	4612	60	Azithromycin	1	58	2/27/2020	99.2	HEIGHT	12.9	13	0	0
18	4614	0	Azithromycin	1	12	3/31/2015	71.6	LENGTH	7.3	12.5	0	0
18	4616	0	Azithromycin	1	48	3/31/2015	98.2	HEIGHT	13.3	14	0	0
18	4616	12	Azithromycin	1	54	7/22/2016	106.5	HEIGHT		13	0	0
18	4616	24	Azithromycin	1	66	4/6/2017	110	HEIGHT	16.35	14.5	0	0

18	4616	36	Azithromycin	1	79	5/25/2018	116.2	HEIGHT	17.68181818	15	0	0
18	4616	48	Azithromycin	1	90	4/20/2019	120.3	HEIGHT	19.4	14.5	0	0
18	4616	60	Azithromycin	1	100	2/27/2020	123.5	HEIGHT	21.6	15.5	0	0
18	4618	24	Azithromycin	0	7	4/6/2017	65.9	LENGTH	7.95	15	1	0
18	4618	60	Azithromycin	0	40	3/6/2020	93	HEIGHT	14.25	16.4	1	0
18	4619	12	Azithromycin	1	9	7/22/2016	73.3	LENGTH		13	0	0
18	4619	24	Azithromycin	1	21	4/6/2017	79.6	HEIGHT	8.5	13.5	1	0
18	4619	36	Azithromycin	1	35	5/25/2018	86.5	HEIGHT	10.04545455	13.5	0	0
18	4620	24	Azithromycin	0	48	4/6/2017	94.6	HEIGHT	13.1	15.5	1	0
18	4621	0	Azithromycin	0	36	3/31/2015	93.7	HEIGHT	11.25	11.5	0	0
18	4621	12	Azithromycin	0	52	7/23/2016	106.8	HEIGHT		12.5	1	0
18	4621	24	Azithromycin	0	60	4/6/2017	111.4	HEIGHT	15.5	12.5	0	0
18	4622	0	Azithromycin	1	36	3/31/2015	92.4	HEIGHT	13.3	16	1	0
18	4622	12	Azithromycin	1	57	7/22/2016	100.6	HEIGHT		16	0	0
18	4622	24	Azithromycin	1	67	4/6/2017	104.7	HEIGHT	16.85	16	0	0
18	4622	36	Azithromycin	1	80	5/25/2018	110.3	HEIGHT	17.1	15.5	0	0
18	4622	48	Azithromycin	1	91	4/20/2019	114.6	HEIGHT	18.95	15.5	0	0
18	4622	60	Azithromycin	1	100	2/27/2020	117.7	HEIGHT	21.85	17.5	0	0
18	4623	0	Azithromycin	1	7	3/31/2015	59	LENGTH	4.8	12.5	1	0
18	4625	12	Azithromycin	0	36	7/22/2016	99.3	HEIGHT		13.5	1	0
18	4628	0	Azithromycin	0	36	3/31/2015	94.2	HEIGHT	11.7	13	0	0
18	4633	12	Azithromycin	1	58	7/22/2016	95.7	HEIGHT		14	1	0
18	4634	0	Azithromycin	0	3	3/31/2015	59.5	LENGTH	6.4	13.5	1	0
18	4634	24	Azithromycin	0	20	4/6/2017	78.2	HEIGHT	11.65	16	0	0
18	4634	36	Azithromycin	0	34	5/25/2018	87.9	HEIGHT	12.77272727	15.5	0	0
18	4634	48	Azithromycin	0	44	4/20/2019	92.6	HEIGHT	13.25	14.5	0	0
18	4634	60	Azithromycin	0	54	2/27/2020	97.7	HEIGHT	14.7	14.5	1	0
18	4636	0	Azithromycin	0	12	6/13/2015	74.4	LENGTH	9	15	0	0
18	4636	12	Azithromycin	0	30	7/22/2016	85.1	HEIGHT		15	1	0
18	4636	24	Azithromycin	0	43	4/6/2017	89.2	HEIGHT	12	14.5	0	0
18	4636	36	Azithromycin	0	52	5/25/2018	97.8	HEIGHT	14.5	15.5	0	0
18	4636	48	Azithromycin	0	63	4/20/2019	102.7	HEIGHT	14.95	14.5	0	0
18	4636	60	Azithromycin	0	72	2/27/2020	109.3	HEIGHT	17.85	15.7	0	0
18	4639	0	Azithromycin	0	10	3/31/2015	71.3	LENGTH	7.1	12.5	1	0
18	4639	12	Azithromycin	0	21	7/22/2016	83.7	HEIGHT		14	0	0
18	4639	36	Azithromycin	0	44	6/11/2018	99.5	HEIGHT	15.55	15.5	1	0
18	4639	48	Azithromycin	0	55	4/20/2019	104.6	HEIGHT	17.6	15.5	1	0
18	4639	60	Azithromycin	0	58	2/27/2020	109.5	HEIGHT	19.5	16.2	0	0
18	4641	0	Azithromycin	0	36	6/13/2015	87.4	HEIGHT	14	17.5	1	0
18	4641	24	Azithromycin	0	54	4/6/2017	99.9	HEIGHT	17.05	17	1	0

18	4641	36	Azithromycin	0	68	5/25/2018	106.6	HEIGHT	17.86363636	16.5	0	0
18	4641	48	Azithromycin	0	79	4/20/2019	109.9	HEIGHT	18.5	15.5	0	0
18	4641	60	Azithromycin	0	88	2/27/2020	114.9	HEIGHT	20.5	16.3	0	0
18	4642	12	Azithromycin	1	12	7/24/2016	70.8	HEIGHT		12.6	1	0
18	4642	36	Azithromycin	1	34	5/25/2018	88.1	HEIGHT	10.18181818	12.5	0	0
18	4642	48	Azithromycin	1	45	4/20/2019	94	HEIGHT	12.15	13.5	1	0
18	4645	0	Azithromycin	0	30	3/31/2015	96.4	HEIGHT	14.9	15	1	0
18	4645	12	Azithromycin	0	41	7/22/2016	104.6	HEIGHT		14.5	1	0
18	4645	24	Azithromycin	0	50	4/6/2017	108.3	HEIGHT	16.95	14.5	0	0
18	4645	36	Azithromycin	0	64	5/25/2018	112.5	HEIGHT	17.75	15	0	0
18	4645	48	Azithromycin	0	75	4/20/2019	115.4	HEIGHT	19.55	15	0	0
18	4645	60	Azithromycin	0	84	2/27/2020	119.6	HEIGHT	22.5	15.5	0	0
18	4646	12	Azithromycin	1	12	7/22/2016	78.7	HEIGHT		13.5	1	0
18	4646	24	Azithromycin	1	24	4/6/2017	84	HEIGHT	10.6	14	1	0
18	4646	36	Azithromycin	1	37	5/25/2018	92.8	HEIGHT	12.5	15	1	0
18	4646	60	Azithromycin	1	58	2/27/2020	88.5	HEIGHT	11.45	13	1	0
18	4647	24	Azithromycin	1	31	4/6/2017	82.4	HEIGHT	10	12.5	1	0
18	4649	12	Azithromycin	0	33	7/23/2016	94.5	HEIGHT		15.5	1	0
18	4650	0	Azithromycin	0	8	3/31/2015	73.3	LENGTH	8.9	15	0	0
18	4650	12	Azithromycin	0	23	7/22/2016	86.2	HEIGHT		14	1	0
18	4650	24	Azithromycin	0	32	4/6/2017	91.5	HEIGHT	13.1	15	0	0
18	4650	36	Azithromycin	0	46	5/25/2018	100.5	HEIGHT	15.3	16	1	0
18	4650	48	Azithromycin	0	57	4/20/2019	106.6	HEIGHT	17.25	16	0	0
18	4650	60	Azithromycin	0	67	2/27/2020	112.1	HEIGHT	18.55	15.2	0	0
18	4651	12	Azithromycin	1	12	7/24/2016	75	HEIGHT		13.1	1	0
18	4651	24	Azithromycin	1	21	4/6/2017	80.7	LENGTH	8.05	12.5	0	0
18	4651	48	Azithromycin	1	45	4/20/2019	97	HEIGHT	12.85	14	0	0
18	4651	60	Azithromycin	1	55	3/6/2020	104.8	HEIGHT	14.65	14.5	0	0
18	4652	24	Azithromycin	1	34	4/7/2017	83.9	HEIGHT	9.4	13	1	0
18	4652	48	Azithromycin	1	58	4/20/2019	97.7	HEIGHT	11.65	12.5	1	0
18	8068	60	Azithromycin	0	12	3/6/2020	75.8	LENGTH	10	15.5	1	0
18	8085	48	Azithromycin	0	47	4/20/2019	88.6	HEIGHT	13.65	15.5	1	0
18	8223	48	Azithromycin	0	38	4/20/2019	84.2	HEIGHT	11.45	14	1	0
18	8223	60	Azithromycin	0	48	2/27/2020	71.9	HEIGHT	6.090909091	15.3	1	0
18	8308	60	Azithromycin	1	11	3/12/2020	68.8	LENGTH	8	15	1	0
18	8328	48	Azithromycin	0	12	4/20/2019	70.6	LENGTH	8.3	13.5	1	0
18	8343	48	Azithromycin	0	12	4/20/2019	73.4	HEIGHT	8.95	14	1	0
18	8371	48	Azithromycin	0	23	4/24/2019	74.5	LENGTH	9.45	14.5	1	0
18	8461	36	Azithromycin	0	15	5/25/2018	76.8	LENGTH	8.409090909	13	1	0
18	8484	48	Azithromycin	1	10	4/20/2019	64.2	HEIGHT	7.1	14	1	0

18	8539	36	Azithromycin	0	26	6/11/2018	72.9	LENGTH	7.2	12	1	0
18	8592	60	Azithromycin	0	38	3/6/2020	88.2	HEIGHT	12.9	14.9	1	0
18	8595	36	Azithromycin	1	21	5/25/2018	74.6	LENGTH	8.5	14	1	0
18	8595	48	Azithromycin	1	32	4/24/2019	82.4	HEIGHT	9.35	14	1	0
18	8637	48	Azithromycin	1	1	4/24/2019	57	LENGTH	5.15	13	1	0
18	8637	60	Azithromycin	1	11	2/27/2020	70.9	LENGTH	7.65	13.2	0	0
18	8641	48	Azithromycin	0	15	4/24/2019	75.7	LENGTH	9.5	13.5	1	0
18	8674	60	Azithromycin	0	31	2/27/2020	84.6	HEIGHT	10.8	14.2	1	0
18	8699	60	Azithromycin	0	31	3/14/2020	89.4	HEIGHT	13.75	17	1	0
18	8770	36	Azithromycin	1	3	5/25/2018	66.2	HEIGHT	6.15	13	1	0
18	8770	48	Azithromycin	1	12	4/20/2019	76.9	HEIGHT	8.9	13.5	0	0
18	8774	36	Azithromycin	1	35	6/11/2018	85.9	LENGTH	11.15	14	1	0
18	8801	48	Azithromycin	0	43	4/20/2019	91.7	HEIGHT	11.95	14	1	0
18	8801	60	Azithromycin	0	52	3/12/2020	97.9	HEIGHT	13.75	15	1	0
18	8844	36	Azithromycin	1	9	5/25/2018	66	LENGTH	5.909090909	11.5	1	0
18	8844	48	Azithromycin	1	20	4/20/2019	74.1	HEIGHT	7.6	12.5	1	0
18	8844	60	Azithromycin	1	29	2/27/2020	82.3	HEIGHT	9.55	13.5	0	0
18	8865	60	Azithromycin	0	3	3/14/2020	64.3	LENGTH	6.8	15.6	1	0
18	8944	36	Azithromycin	0	1	5/25/2018	54.5	LENGTH	4	11	1	0
18	8973	36	Azithromycin	1	15	5/25/2018	71.1	LENGTH	8.136363636	14.5	1	0
18	8973	48	Azithromycin	1	26	4/20/2019	75.7	HEIGHT	8.65	13	1	0
18	8983	48	Azithromycin	1	15	4/20/2019	78.7	HEIGHT	9.25	14.5	1	0
18	8983	60	Azithromycin	1	25	3/14/2020	87.6	HEIGHT	11.85	14.5	1	0
18	9003	36	Azithromycin	0	11	6/11/2018	77	LENGTH	9.8	14.5	1	0
18	9003	48	Azithromycin	0	22	4/20/2019	83.1	HEIGHT	10.95	14.5	1	0
18	9003	60	Azithromycin	0	34	2/27/2020	88.6	HEIGHT	12.85	14.7	0	0
18	9064	48	Azithromycin	0	15	4/20/2019	106.4	HEIGHT	14.45	13	1	0
18	9083	48	Azithromycin	0	2	4/24/2019	59.6	LENGTH	5.7	13	1	0
18	9083	60	Azithromycin	0	11	3/12/2020	74.3	LENGTH	8.2	13.5	1	0
18	9091	36	Azithromycin	0	50	5/25/2018	99.4	HEIGHT	14.04545455	14.5	1	0
18	9140	48	Azithromycin	0	24	4/20/2019	83.3	HEIGHT	10.6	15	1	0
18	9159	60	Azithromycin	0	3	2/27/2020	66.6	LENGTH	6.95	14.2	1	0
18	9188	60	Azithromycin	1	31	3/6/2020	84.6	HEIGHT	11.25	15	1	0
18	9222	60	Azithromycin	1	58	3/6/2020	96.2	HEIGHT	13.55	14.5	1	0
18	9238	36	Azithromycin	0	23	5/25/2018	82.8	HEIGHT	9.409090909	13.5	1	0
18	9238	48	Azithromycin	0	34	4/20/2019	90.5	HEIGHT	11.75	14.5	1	0
18	9246	36	Azithromycin	1	34	5/25/2018	85.3	HEIGHT	11	14	1	0
18	9287	36	Azithromycin	0	7	5/25/2018	64.8	LENGTH	7.136363636	13.5	1	0
18	9287	48	Azithromycin	0	14	4/20/2019	75.5	HEIGHT	9.7	15	1	0
18	9287	60	Azithromycin	0	24	2/27/2020	83	HEIGHT	11.25	13.5	0	0

18	9356	48	Azithromycin	1	34	4/20/2019	90.1	HEIGHT	10.4	12.5		1	0
18	9366	48	Azithromycin	0	30	4/24/2019	80.9	HEIGHT	9.95	13.5		1	0
18	9366	60	Azithromycin	0	40	2/27/2020	89.1	HEIGHT	11.75	14		1	0
18	9393	60	Azithromycin	1	14	3/14/2020	76.2	LENGTH	9.15	14.6		1	0
18	9408	48	Azithromycin	1	34	4/20/2019	92	HEIGHT	13.15	14.5		1	0
18	9408	60	Azithromycin	1	44	3/6/2020	99.3	HEIGHT	14.55	15.7		1	0
18	9416	60	Azithromycin	1	23	3/12/2020	85.6	HEIGHT	11.85	15.5		1	0
18	9420	48	Azithromycin	0	21	4/24/2019	76.6	LENGTH	7.85	12.5		1	0
18	9432	48	Azithromycin	1	12	4/20/2019	70.5	LENGTH	8.05	15		1	0
18	9432	60	Azithromycin	1	21	3/6/2020	78.1	HEIGHT	8.9	13		1	0
18	9437	36	Azithromycin	1	11	5/25/2018	72.8	LENGTH	8	13		1	0
18	9441	36	Azithromycin	0	21	5/25/2018	77	LENGTH	8.75	13.5		1	0
18	9448	36	Azithromycin	0	21	5/25/2018	80.6	HEIGHT	9.727272727	14.5		1	0
18	9448	48	Azithromycin	0	34	4/24/2019	85.4	LENGTH	11.5	14.5		1	0
18	9461	36	Azithromycin	0	18	5/25/2018	76.4	HEIGHT	9.863636364	15		1	0
18	9471	36	Azithromycin	1	15	5/25/2018	73.6	LENGTH	6.954545455	11.5		1	0
18	9471	60	Azithromycin	1	35	3/6/2020	89.2	HEIGHT	11.5	13.4		1	0
18	9478	48	Azithromycin	0	54	4/20/2019	98.4	HEIGHT	14.1	14.5		1	0
18	9478	60	Azithromycin	0	58	3/6/2020	103.8	HEIGHT	15.9	13.9		1	0
18	9505	48	Azithromycin	0	3	4/24/2019	62.6	LENGTH	6.35	13.5		1	0
18	9575	36	Azithromycin	1	27	5/25/2018	79.4	HEIGHT	9.181818182	13.5		1	0
19	4664	24	Azithromycin	0	21	4/3/2017	84.2	HEIGHT	11.1	14	30	1	0
19	4673	0	Azithromycin	0	36	3/30/2015	89	HEIGHT	11.05	13		1	0
19	4673	12	Azithromycin	0	50	6/13/2016	97.2	HEIGHT	12.3	14		1	0
19	4673	48	Azithromycin	0	87	4/23/2019	111.9	HEIGHT	16.5	14		0	0
19	4673	60	Azithromycin	0	96	2/12/2020	115.9	HEIGHT	16.9	14.1		0	0
19	4675	0	Azithromycin	0	24	3/30/2015	80.9	HEIGHT	9.65	13	18	1	0
19	4680	12	Azithromycin	0	9	6/13/2016	71.1	HEIGHT	7.35	13		1	0
19	4680	24	Azithromycin	0	21	4/3/2017	79.5	HEIGHT	9	14		1	0
19	4680	36	Azithromycin	0	36	6/12/2018	85.7	HEIGHT	10.35	13		1	0
19	4680	60	Azithromycin	0	56	2/12/2020	98	HEIGHT	13.5	14.1		1	0
19	4681	0	Azithromycin	1	36	3/30/2015	87.7	HEIGHT	11.2	13.5	6	1	0
19	4682	0	Azithromycin	1	12	3/30/2015	75.6	HEIGHT	8.9	13.5		1	0
19	4682	12	Azithromycin	1	28	6/13/2016	83.1	HEIGHT	10.65	14		1	0
19	4682	24	Azithromycin	1	38	4/3/2017	90.6	HEIGHT	12.65	13.5		1	0
19	4682	36	Azithromycin	1	52	6/12/2018	98.8	HEIGHT	15.09090909	14		1	0
19	4682	48	Azithromycin	1	63	4/23/2019	102.4	HEIGHT	15.45	14		0	0
19	4686	12	Azithromycin	1	12	6/13/2016	66.9	HEIGHT	6.45	13		1	0
19	4686	24	Azithromycin	1	24	4/3/2017	72.1	HEIGHT	8.15	13		1	0
19	4686	36	Azithromycin	1	37	6/12/2018	78.9	LENGTH	8.05	12		1	0

19	4686	60	Azithromycin	1	58	2/12/2020	89.7	HEIGHT	10.45	12.2	1	0
19	4688	0	Azithromycin	0	48	3/30/2015	93.2	HEIGHT	10.45	13	1	0
19	4688	36	Azithromycin	0	88	6/12/2018	108.8	HEIGHT	13.68181818	13	0	0
19	4688	48	Azithromycin	0	99	4/23/2019	112.5	HEIGHT	15.15	14	0	0
19	4688	60	Azithromycin	0	108	2/12/2020	116.6	HEIGHT	16.4	13.1	0	0
19	4691	0	Azithromycin	0	36	3/30/2015	79.7	HEIGHT	9.35	13	1	0
19	4691	12	Azithromycin	0	52	6/14/2016	86.6	HEIGHT	10.5	13	1	0
19	4691	24	Azithromycin	0	62	4/3/2017	89.4	HEIGHT	10.35	12	0	0
19	4691	48	Azithromycin	0	87	4/23/2019	101.9	HEIGHT	14.85	13	0	0
19	4691	60	Azithromycin	0	96	2/12/2020	105.7	HEIGHT	15.5	13.3	0	0
19	4694	12	Azithromycin	0	27	6/13/2016	93.4	HEIGHT	12.6	15	1	0
19	4694	24	Azithromycin	0	39	4/8/2017	98.9	HEIGHT	13.65	14	1	0
19	4694	48	Azithromycin	0	64	4/23/2019	112	HEIGHT	16.65	14	0	0
19	4699	0	Azithromycin	0	48	3/30/2015	111.6	HEIGHT	16.1	14	1	0
19	4700	0	Azithromycin	0	12	3/30/2015	67.5	LENGTH	8.5	14	1	0
19	4700	12	Azithromycin	0	26	6/14/2016	82.9	LENGTH	10.35	15	1	0
19	4700	24	Azithromycin	0	38	4/3/2017	88.9	HEIGHT	12.15	14	1	0
19	4700	36	Azithromycin	0	52	6/12/2018	98.9	HEIGHT	14	14.5	1	0
19	4700	48	Azithromycin	0	63	4/23/2019	105.9	HEIGHT	15.8	14.5	0	0
19	4700	60	Azithromycin	0	72	2/12/2020	111.2	HEIGHT	17.5	14.5	0	0
19	4701	12	Azithromycin	0	24	6/13/2016	85.2	HEIGHT	10.6	13.5	1	0
19	4701	36	Azithromycin	0	47	6/12/2018	98.9	HEIGHT	12.95454545	13	1	0
19	4701	48	Azithromycin	0	58	4/23/2019	103.6	HEIGHT	14.8	13	1	0
19	4701	60	Azithromycin	0	68	2/12/2020	107.6	HEIGHT	15.3	12.5	0	0
19	4702	0	Azithromycin	1	12	3/30/2015	79.8	LENGTH	11.05	15	1	0
19	4702	60	Azithromycin	1	61	2/12/2020	119.3	HEIGHT	21.75	16	0	0
19	4703	12	Azithromycin	0	39	6/13/2016	98.4	HEIGHT	12.4	13.5	1	0
19	4703	36	Azithromycin	0	62	6/12/2018	109.6	HEIGHT	14.45454545	13	0	0
19	4703	48	Azithromycin	0	73	4/23/2019	113.4	HEIGHT	15.7	13.5	0	0
19	4703	60	Azithromycin	0	83	2/12/2020	117.4	HEIGHT	16.75	14	0	0
19	4705	12	Azithromycin	1	4	6/14/2016	64.3	LENGTH	6.7	13.5	1	0
19	4705	36	Azithromycin	1	28	6/12/2018	86.5	HEIGHT	9.863636364	15.5	1	0
19	4705	48	Azithromycin	1	39	4/23/2019	90.5	HEIGHT	11.65	13.5	1	0
19	4705	60	Azithromycin	1	49	2/12/2020	94.4	HEIGHT	12.5	13.7	1	0
19	4706	12	Azithromycin	1	44	6/13/2016	98.1	HEIGHT	11.4	13.5	1	0
19	4706	24	Azithromycin	1	56	4/3/2017	105.3	HEIGHT	13	13	1	0
19	4706	36	Azithromycin	1	70	6/12/2018	111.7	HEIGHT	14	13	0	0
19	4706	48	Azithromycin	1	81	4/23/2019	115.8	HEIGHT	15.05	14	0	0
19	4706	60	Azithromycin	1	90	2/12/2020	120.5	HEIGHT	16.9	14.1	0	0
19	4709	12	Azithromycin	1	12	6/13/2016	77.3	LENGTH	8.75	14	1	0

19	4714	0	Azithromycin	0	12	3/30/2015	72.6	HEIGHT	8	13	1	0
19	4714	24	Azithromycin	0	38	4/3/2017	92.3	HEIGHT	12.15	14	1	0
19	4714	36	Azithromycin	0	52	6/12/2018	99.7	HEIGHT	13.36363636	14	1	0
19	4714	48	Azithromycin	0	63	4/23/2019	106.6	HEIGHT	15.25	14	0	0
19	4714	60	Azithromycin	0	61	2/12/2020	111.1	HEIGHT	15.8	13	0	0
19	4715	12	Azithromycin	1	44	6/14/2016	91.4	HEIGHT	14.3	17	1	0
19	4717	24	Azithromycin	1	6	4/3/2017	63.6	LENGTH	5.8	12	1	0
19	4717	36	Azithromycin	1	19	6/12/2018	76.6	LENGTH	8.772727273	13	1	0
19	4717	48	Azithromycin	1	30	4/23/2019	84.1	HEIGHT	9.95	12.5	1	0
19	4717	60	Azithromycin	1	39	2/12/2020	91.2	HEIGHT	11.4	12.5	1	0
19	4720	0	Azithromycin	0	48	3/30/2015	99.8	HEIGHT	13.35	13.5	1	0
19	4720	12	Azithromycin	0	62	6/13/2016	106.9	HEIGHT	14.3	14	0	0
19	4720	24	Azithromycin	0	74	4/3/2017	112.3	HEIGHT	16.25	13.5	0	0
19	4720	36	Azithromycin	0	88	6/12/2018	117.9	HEIGHT	18	13.5	0	0
19	4720	48	Azithromycin	0	99	4/23/2019	121.9	HEIGHT	19.4	14	0	0
19	4720	60	Azithromycin	0	108	2/12/2020	124	HEIGHT	20.75	13.2	0	0
19	4721	0	Azithromycin	0	5	3/30/2015	65.6	LENGTH	6.25	12	1	0
19	4721	24	Azithromycin	0	27	4/3/2017	84.4	HEIGHT	9.55	13	1	0
19	4721	48	Azithromycin	0	52	4/23/2019	96.3	HEIGHT	11.7	12.5	1	0
19	4721	60	Azithromycin	0	61	2/12/2020	100.4	HEIGHT	13.6	13.7	0	0
19	4725	12	Azithromycin	0	45	6/13/2016	110.9	HEIGHT	15.65	15	1	0
19	4725	24	Azithromycin	0	57	4/8/2017	114.6	HEIGHT	16.65	14	1	0
19	4725	48	Azithromycin	0	81	4/23/2019	123.4	HEIGHT	20.55	15	0	0
19	4726	12	Azithromycin	0	27	6/13/2016	96.9	HEIGHT	13.15	15	1	0
19	4726	60	Azithromycin	0	61	2/12/2020	122.9	LENGTH	20.4	14.2	0	0
19	4727	0	Azithromycin	0	4	3/30/2015	64.8	LENGTH	7.55	14	1	0
19	4727	36	Azithromycin	0	40	6/12/2018	93	HEIGHT	12.77272727	14.5	1	0
19	4727	48	Azithromycin	0	51	4/23/2019	97.3	HEIGHT	15.05	15.5	1	0
19	4727	60	Azithromycin	0	60	2/12/2020	103.2	HEIGHT	16.3	15.5	0	0
19	4729	24	Azithromycin	1	45	4/3/2017	95.8	HEIGHT	12.9	17	1	0
19	4729	36	Azithromycin	1	58	6/12/2018	105.3	HEIGHT	14.59090909	14	1	0
19	4732	12	Azithromycin	0	3	6/13/2016	66.6	LENGTH	6.7	14	1	0
19	4732	24	Azithromycin	0	15	4/3/2017	79.7	HEIGHT	9.3	13.5	1	0
19	4732	36	Azithromycin	0	28	6/12/2018	89.5	HEIGHT	12.22727273	14.5	1	0
19	4732	48	Azithromycin	0	39	4/23/2019	96.6	HEIGHT	14.15	14.5	1	0
19	4734	0	Azithromycin	1	24	3/30/2015	82.2	HEIGHT	9.5	13.5	1	0
19	4734	12	Azithromycin	1	38	6/14/2016	91.3	HEIGHT	11.15	14	1	0
19	4734	36	Azithromycin	1	64	6/12/2018	103.4	HEIGHT	13.59090909	14	0	0
19	4734	48	Azithromycin	1	75	4/23/2019	106.3	HEIGHT	15.25	14	0	0
19	4734	60	Azithromycin	1	84	2/12/2020	109.8	HEIGHT	16.3	14.1	0	0

19	4738	0	Azithromycin	1	12	3/30/2015	69.4	LENGTH	7.5	13.5		1	0
19	4738	12	Azithromycin	1	28	6/14/2016	76.4	HEIGHT	8.5	12.5		1	0
19	4739	0	Azithromycin	0	12	3/30/2015	82	HEIGHT	11.35	15.5		1	0
19	4739	48	Azithromycin	0	56	4/23/2019	107.8	HEIGHT	18	16		1	0
19	4739	60	Azithromycin	0	56	2/12/2020	112.3	HEIGHT	19.8	16.5		1	0
19	4740	0	Azithromycin	0	24	3/30/2015	83.8	HEIGHT	8.95	13		1	0
19	4740	12	Azithromycin	1	24	6/13/2016	92.1	HEIGHT	11.5	15		1	0
19	4740	24	Azithromycin	1	36	4/3/2017	98.3	HEIGHT	14.1	14.5		1	0
19	4740	48	Azithromycin	1	61	4/23/2019	111.7	HEIGHT	17.45	15		0	0
19	4740	60	Azithromycin	1	70	2/12/2020	116.3	HEIGHT	19.05	14.5		0	0
19	4741	0	Azithromycin	0	48	3/30/2015	93.5	HEIGHT	11.5	13.5		1	0
19	4741	24	Azithromycin	0	76	4/3/2017	105.1	HEIGHT	13.25	13		0	0
19	4741	36	Azithromycin	0	89	6/12/2018	111.3	HEIGHT	14.59090909	13.5		0	0
19	4741	48	Azithromycin	0	100	4/23/2019	115.6	HEIGHT	15.5	13.5		0	0
19	4741	60	Azithromycin	0	104	2/12/2020	117.9	HEIGHT	16.5	13.5		0	0
19	4742	24	Azithromycin	0	54	4/3/2017	104.4	HEIGHT	12.9	13		1	0
19	4742	36	Azithromycin	0	68	6/12/2018	108.6	HEIGHT	13.72727273	12.5		0	0
19	4742	48	Azithromycin	0	79	4/23/2019	112	HEIGHT	15.7	13.5		0	0
19	4745	12	Azithromycin	1	14	6/13/2016	74.2	HEIGHT	7.5	14		1	0
19	4745	24	Azithromycin	1	27	4/3/2017	83.3	HEIGHT	9.5	14		1	0
19	4749	0	Azithromycin	0	48	3/30/2015	99.8	HEIGHT	13.55	14		1	0
19	4751	0	Azithromycin	0	48	3/30/2015	102.6	HEIGHT	15.2	15		1	0
19	4751	12	Azithromycin	0	66	6/14/2016	109.8	HEIGHT	16.4	15		0	0
19	4751	48	Azithromycin	0	100	4/23/2019	121.9	HEIGHT	19.25	14		0	0
19	4753	24	Azithromycin	1	21	4/3/2017	81.2	HEIGHT	9.4	13.5	36	1	0
19	4756	0	Azithromycin	1	36	3/30/2015	84	HEIGHT	9.25	12.5		1	0
19	4756	12	Azithromycin	1	42	6/13/2016	91.3	HEIGHT	10.95	13.5		1	0
19	4756	24	Azithromycin	1	54	4/3/2017	96.6	HEIGHT	11.55	12.5		1	0
19	4756	48	Azithromycin	1	78	4/23/2019	107.2	HEIGHT	14.35	13.5		0	0
19	4756	60	Azithromycin	1	87	2/12/2020	112.3	HEIGHT	16.2	13.6		0	0
19	4757	0	Azithromycin	1	5	3/30/2015	70.1	LENGTH	7.05	11.5	42	1	0
19	4759	0	Azithromycin	0	48	3/30/2015	99.4	HEIGHT	13.5	14		1	0
19	4759	48	Azithromycin	0	79	4/23/2019	123.7	HEIGHT	21.05	14		0	0
19	4760	12	Azithromycin	0	2	6/14/2016	57.4	LENGTH	4.95	13	48	1	0
19	4763	0	Azithromycin	1	36	3/30/2015	93.6	HEIGHT	12.35	13.5		1	0
19	4763	12	Azithromycin	1	52	6/13/2016	101.5	HEIGHT	13.8	14.5		1	0
19	4763	24	Azithromycin	1	62	4/3/2017	107.1	HEIGHT	14.45	13.5		0	0
19	4763	36	Azithromycin	1	76	6/12/2018	113.8	HEIGHT	17.09090909	14		0	0
19	4763	48	Azithromycin	1	87	4/23/2019	119.5	HEIGHT	18.3	14		0	0
19	4763	60	Azithromycin	1	96	2/12/2020	123.6	HEIGHT	20.45	14		0	0

19	4764	12	Azithromycin	0	12	6/14/2016	65.4	LENGTH	5.65	12		1	0
19	4764	48	Azithromycin	0	48	4/23/2019	88.7	HEIGHT	11	13		1	0
19	4764	60	Azithromycin	0	58	2/12/2020	94.2	HEIGHT	12.05	13.1		1	0
19	4765	0	Azithromycin	1	36	3/30/2015	86.4	HEIGHT	11.85	15		1	0
19	4767	24	Azithromycin	0	42	4/3/2017	98.4	HEIGHT	13.9	14		1	0
19	4769	0	Azithromycin	0	3	3/30/2015	61.1	LENGTH	5.95	12.5	18	1	0
19	4769	12	Azithromycin	0	16	6/14/2016	72.2	LENGTH	7.85	13	18	1	0
19	4771	0	Azithromycin	1	48	3/30/2015	106.7	HEIGHT	15.25	14		1	0
19	4771	12	Azithromycin	1	64	6/13/2016	111	HEIGHT	15.55	14.5		0	0
19	4772	12	Azithromycin	1	11	6/13/2016	72.6	HEIGHT	7.7	14		1	0
19	4772	24	Azithromycin	1	21	4/3/2017	80.3	LENGTH	9.3	13		1	0
19	4772	36	Azithromycin	1	34	6/12/2018	91.9	HEIGHT	11.63636364	14.5		1	0
19	4772	60	Azithromycin	1	55	2/12/2020	103.1	HEIGHT	13.8	13.5		1	0
19	4774	0	Azithromycin	1	54	3/30/2015	113.8	HEIGHT	18.3	15		1	0
19	4774	24	Azithromycin	1	80	4/3/2017	126.5	HEIGHT	21	15		0	0
19	4775	0	Azithromycin	0	36	3/30/2015	98.1	HEIGHT	15	15		1	0
19	4775	12	Azithromycin	0	52	6/13/2016	105.1	HEIGHT	15.85	15		1	0
19	4775	24	Azithromycin	0	62	4/3/2017	110.5	HEIGHT	17.1	14.5		0	0
19	4775	36	Azithromycin	0	76	6/12/2018	112.8	HEIGHT	17.3	14		0	0
19	4775	48	Azithromycin	0	87	4/23/2019	114.5	HEIGHT	19.4	15		0	0
19	4775	60	Azithromycin	0	96	2/12/2020	117.5	HEIGHT	20.35	15.3		0	0
19	4776	0	Azithromycin	1	24	3/30/2015	83.4	HEIGHT	10.35	15.5	36	1	0
19	4776	12	Azithromycin	1	42	6/14/2016	93.2	HEIGHT	12.25	15	36	1	0
19	4778	0	Azithromycin	0	24	3/30/2015	88.7	HEIGHT	11	14		1	0
19	4778	48	Azithromycin	0	76	4/23/2019	118.9	HEIGHT	18.65	14		0	0
19	4778	60	Azithromycin	0	86	2/12/2020	120.8	HEIGHT	19.55	13.6		0	0
19	4780	24	Azithromycin	0	15	4/3/2017	76.7	HEIGHT	7.75	12.5		1	0
19	4780	36	Azithromycin	0	28	6/12/2018	82.5	HEIGHT	9	12.5		1	0
19	4781	0	Azithromycin	1	24	3/30/2015	83.2	HEIGHT	11.25	16		1	0
19	4781	36	Azithromycin	1	63	6/12/2018	106.9	HEIGHT	17.35	17		0	0
19	4781	48	Azithromycin	1	74	4/23/2019	111	HEIGHT	18.3	15		0	0
19	4782	12	Azithromycin	0	9	6/13/2016	98.2	HEIGHT	12.15	14		1	0
19	4782	36	Azithromycin	0	54	6/12/2018	112.4	HEIGHT	15.22727273	13.5		1	0
19	4782	48	Azithromycin	0	65	4/23/2019	117.5	HEIGHT	16.75	14		0	0
19	8076	48	Azithromycin	1	51	4/23/2019	98.3	HEIGHT	13.2	14		1	0
19	8076	60	Azithromycin	1	61	2/12/2020	101.6	HEIGHT	14.75	14.5		0	0
19	8117	36	Azithromycin	0	18	6/12/2018	79.5	LENGTH	8.363636364	12.5		1	0
19	8117	48	Azithromycin	0	28	4/23/2019	85	HEIGHT	10.15	14		1	0
19	8117	60	Azithromycin	0	38	2/12/2020	90.2	LENGTH	10.95	13.8		1	0
19	8140	36	Azithromycin	0	4	6/12/2018	61.6	LENGTH	4.85	11		1	0

19	8144	36	Azithromycin	0	18	6/12/2018	80.2	LENGTH	10.05	13.5		1	0
19	8144	48	Azithromycin	0	28	4/23/2019	86.1	HEIGHT	11.1	13		1	0
19	8242	36	Azithromycin	1	40	6/12/2018	93.1	HEIGHT	13.15	14	42	1	0
19	8285	48	Azithromycin	0	12	4/23/2019	70.7	LENGTH	7.2	12		1	0
19	8357	60	Azithromycin	0	12	2/12/2020	74.8	LENGTH	7.25	12.5		1	0
19	8443	48	Azithromycin	1	4	4/23/2019	66.4	LENGTH	7	13		1	0
19	8463	36	Azithromycin	0	7	6/12/2018	62.1	LENGTH	5.590909091	11.5		1	0
19	8540	48	Azithromycin	0	39	4/23/2019	72.1	LENGTH	7.05	11		1	0
19	8752	36	Azithromycin	1	11	6/12/2018	71.9	LENGTH	7.909090909	13.5		1	0
19	8752	48	Azithromycin	1	21	4/23/2019	79.1	LENGTH	9.4	14		1	0
19	8752	60	Azithromycin	1	31	2/12/2020	86.3	HEIGHT	11.9	14.1		1	0
19	8809	36	Azithromycin	0	18	6/12/2018	88	HEIGHT	11.35	13.5		1	0
19	8809	48	Azithromycin	0	28	4/23/2019	94.4	HEIGHT	13.45	14		1	0
19	8825	36	Azithromycin	1	7	6/12/2018	60.8	LENGTH	4.181818182	9.5	42	1	0
19	8850	48	Azithromycin	1	2	4/23/2019	58.9	LENGTH	5.05	11		1	0
19	8850	60	Azithromycin	1	11	2/12/2020	72.1	HEIGHT	7.8	12.5		1	0
19	8990	36	Azithromycin	0	12	6/12/2018	74.8	LENGTH	9.45	14.5		1	0
19	8990	48	Azithromycin	0	22	4/23/2019	79.5	HEIGHT	11.05	14		1	0
19	8990	60	Azithromycin	0	32	2/12/2020	85.9	HEIGHT	12.9	15.2		1	0
19	9181	48	Azithromycin	1	14	4/23/2019	81.5	HEIGHT	8.8	12		1	0
19	9181	60	Azithromycin	1	25	2/12/2020	85.2	HEIGHT	10	12.2		1	0
19	9220	48	Azithromycin	1	93	4/23/2019	123.7	HEIGHT	20.1	14.5		0	0
19	9485	36	Azithromycin	0	12	6/12/2018	53.2	LENGTH	3.045454545	8		1	0
19	9485	48	Azithromycin	0	22	4/23/2019	58.5	LENGTH	3.8	8.5		1	0
19	9487	48	Azithromycin	1	70	4/23/2019	109.4	HEIGHT	15.4	13		0	0
19	9526	48	Azithromycin	1	45	4/23/2019	92.8	HEIGHT	11.3	12.5		1	0
20	4793	12	Azithromycin	1	56	5/15/2016	101.2	HEIGHT	15.85	15		1	0
20	4794	12	Azithromycin	1	3	5/13/2016	63	LENGTH	5.65	12.5		0	0
20	4794	24	Azithromycin	1	14	3/17/2017	74.3	LENGTH	7.95	13		1	0
20	4794	36	Azithromycin	1	28	5/14/2018	82.6	HEIGHT	10.4	14.2		1	0
20	4794	48	Azithromycin	1	39	4/12/2019	89.9	HEIGHT	11.75	13.3		1	0
20	4794	60	Azithromycin	1	49	2/6/2020	96.9	HEIGHT	13.4	14.1		1	0
20	4797	0	Azithromycin	1	48	3/11/2015	92.9	HEIGHT	13.9	15.5		0	0
20	4797	12	Azithromycin	1	60	6/12/2016	101.8	HEIGHT	15.15	14		0	0
20	4797	36	Azithromycin	1	86	5/14/2018	114	HEIGHT	18.75	14.4		0	0
20	4797	48	Azithromycin	1	97	4/12/2019	117.9	HEIGHT	20.4	15		0	0
20	4798	0	Azithromycin	0	12	3/11/2015	72.5	LENGTH	8.6	15	6	1	0
20	4799	0	Azithromycin	1	48	3/11/2015	89.6	HEIGHT	12.3	15.4		1	0
20	4799	12	Azithromycin	1	62	5/13/2016	96.2	LENGTH	12.65	14.5		0	0
20	4799	24	Azithromycin	1	73	3/17/2017	101.5	HEIGHT	14.7	14.5		0	0

20	4799	36	Azithromycin	1	87	5/14/2018	106	HEIGHT	16.3	15		0	0
20	4799	48	Azithromycin	1	98	4/12/2019	111	HEIGHT	17.75	14.9		0	0
20	4801	12	Azithromycin	0	32	5/13/2016	90.7	LENGTH	13.4	15.5		1	0
20	4801	24	Azithromycin	0	49	3/17/2017	98	HEIGHT	14.55	14.2		1	0
20	4802	24	Azithromycin	0	6	3/17/2017	68.8	LENGTH	7.95	14		1	0
20	4802	60	Azithromycin	0	38	2/6/2020	93.9	HEIGHT	15.2	15.5		1	0
20	4804	0	Azithromycin	0	32	3/11/2015	83.4	HEIGHT	9.95	14.7		1	0
20	4805	24	Azithromycin	1	5	3/17/2017	62.3	LENGTH	5.6	12.5		1	0
20	4805	36	Azithromycin	1	14	5/14/2018	75	HEIGHT	8.45	13		0	0
20	4805	48	Azithromycin	1	25	4/12/2019	82	HEIGHT	10.25	13.3		1	0
20	4805	60	Azithromycin	1	35	2/6/2020	90.2	HEIGHT	12.55	15		1	0
20	4806	0	Azithromycin	0	48	3/11/2015	106.8	HEIGHT	16.6	15.7		0	0
20	4806	12	Azithromycin	0	62	5/13/2016	112	HEIGHT	17.55	16		0	0
20	4806	24	Azithromycin	0	73	3/17/2017	118.5	HEIGHT	19.7	15.4		0	0
20	4806	36	Azithromycin	0	87	5/14/2018	123.5	HEIGHT	20.7	16		0	0
20	4806	48	Azithromycin	0	98	4/12/2019	127.8	HEIGHT	23.1	17		0	0
20	4808	0	Azithromycin	1	9	3/11/2015	68.6	LENGTH	7.1	13.5	6	1	0
20	4811	0	Azithromycin	0	48	3/11/2015	94.3	HEIGHT	15.4	16.5		1	0
20	4811	12	Azithromycin	0	54	6/12/2016	102.8	HEIGHT	16.45	15.5		1	0
20	4811	24	Azithromycin	0	65	3/17/2017	107.5	HEIGHT	17.7	15.1		0	0
20	4811	36	Azithromycin	0	80	5/14/2018	112.9	HEIGHT	19.8	15		0	0
20	4811	48	Azithromycin	0	91	4/12/2019	117.1	HEIGHT	22.05	15.3		0	0
20	4811	60	Azithromycin	0	100	2/6/2020	122.1	HEIGHT	23.4	16.1		0	0
20	4812	0	Azithromycin	1	6	3/11/2015	67.5	LENGTH	6.55	13		1	0
20	4812	12	Azithromycin	1	18	6/12/2016	79.4	HEIGHT	9.9	14.5		1	0
20	4812	24	Azithromycin	1	33	3/17/2017	83.5	HEIGHT	10.5	13		1	0
20	4812	36	Azithromycin	1	47	5/14/2018	94	HEIGHT	13.65	14.5		1	0
20	4812	48	Azithromycin	1	58	4/12/2019	102.3	HEIGHT	14.9	14.6		1	0
20	4812	60	Azithromycin	1	68	2/6/2020	107	HEIGHT	15.8	14.5		0	0
20	4813	24	Azithromycin	0	10	3/17/2017	73.9	HEIGHT	8.05	15.5		1	0
20	4813	36	Azithromycin	0	24	5/14/2018	82	HEIGHT	10	13.8		1	0
20	4813	60	Azithromycin	0	45	2/6/2020	96	HEIGHT	13.9	14.5		1	0
20	4814	12	Azithromycin	1	20	5/13/2016	72.3	LENGTH	8	13		0	0
20	4814	24	Azithromycin	1	31	3/17/2017	82.1	HEIGHT	10.45	15.4		1	0
20	4814	36	Azithromycin	1	45	5/14/2018	92	HEIGHT	13.55	15.5		1	0
20	4814	48	Azithromycin	1	56	4/12/2019	98.8	HEIGHT	15.5	15.7		1	0
20	4817	12	Azithromycin	1	44	5/13/2016	98	HEIGHT	14.5	15.5		1	0
20	4817	24	Azithromycin	1	53	3/17/2017	105.3	HEIGHT	16.15	14.5		1	0
20	4820	0	Azithromycin	1	24	3/11/2015	83.7	HEIGHT	9.65	13.1		1	0
20	4820	24	Azithromycin	1	41	3/17/2017	98.5	HEIGHT	13.15	13.5		1	0

20	4820	36	Azithromycin	1	68	5/14/2018	107	HEIGHT	15.05	13.5		0	0
20	4821	12	Azithromycin	1	5	5/13/2016	62.3	LENGTH	5.05	11.5		1	0
20	4821	24	Azithromycin	1	14	3/17/2017	70	HEIGHT	7.3	12.5		0	0
20	4821	36	Azithromycin	1	28	5/14/2018	76	HEIGHT	9.8	13.3		0	0
20	4821	48	Azithromycin	1	28	4/12/2019	86.4	HEIGHT	12.15	14.5		1	0
20	4822	24	Azithromycin	0	53	3/17/2017	106	HEIGHT	17.85	15.5		1	0
20	4823	0	Azithromycin	0	30	3/11/2015	83.7	HEIGHT	11.65	16.6		0	0
20	4823	12	Azithromycin	0	44	5/13/2016	95	LENGTH	13.55	16.5		1	0
20	4823	24	Azithromycin	0	60	3/17/2017	101.6	HEIGHT	15.35	15.7		0	0
20	4823	36	Azithromycin	0	74	5/14/2018	107	HEIGHT	15.85	14		0	0
20	4823	48	Azithromycin	0	85	4/12/2019	114.9	HEIGHT	18.5	15.6		0	0
20	4823	60	Azithromycin	0	95	2/6/2020	118.8	HEIGHT	20.5	17		0	0
20	4824	0	Azithromycin	0	18	3/11/2015	78.1	HEIGHT	8.3	12.5	30	0	0
20	4824	12	Azithromycin	0	32	5/13/2016	86.2	HEIGHT	10.9	14.5	30	1	0
20	4824	24	Azithromycin	0	43	3/17/2017	96	HEIGHT	12.6	13.5	30	1	0
20	4825	12	Azithromycin	0	54	6/12/2016	96.7	HEIGHT	15.15	14.5		1	0
20	4826	0	Azithromycin	0	12	3/11/2015	78	LENGTH	10.95	17		0	0
20	4826	12	Azithromycin	0	26	5/13/2016	85.2	LENGTH	11.85	16.5		0	0
20	4826	36	Azithromycin	0	51	5/14/2018	97	HEIGHT	15.15	16		0	0
20	4826	48	Azithromycin	0	62	4/12/2019	100.9	HEIGHT	16	15		0	0
20	4826	60	Azithromycin	0	72	2/6/2020	105.2	HEIGHT	17	16.4		0	0
20	4828	0	Azithromycin	0	30	3/11/2015	95.7	HEIGHT	13.85	16.1		0	0
20	4829	0	Azithromycin	1	30	3/11/2015	89.1	HEIGHT	12	15		1	0
20	4829	12	Azithromycin	1	54	5/13/2016	97.9	HEIGHT	13.75	15.5		1	0
20	4830	0	Azithromycin	0	36	3/11/2015	93.3	HEIGHT	14	15.9		1	0
20	4830	24	Azithromycin	0	61	3/17/2017	105	HEIGHT	16.2	14.2		0	0
20	4830	36	Azithromycin	0	75	5/14/2018	110	HEIGHT	16.8	14.5		0	0
20	4830	48	Azithromycin	0	86	4/12/2019	114	HEIGHT	19.6	15		0	0
20	4830	60	Azithromycin	0	96	2/6/2020	117.2	HEIGHT	21.1	16		0	0
20	4832	0	Azithromycin	0	48	3/11/2015	98.6	HEIGHT	15.35	15.1		1	0
20	4832	24	Azithromycin	0	73	3/17/2017	114	HEIGHT	18.75	14.6		0	0
20	4832	36	Azithromycin	0	87	5/14/2018	121.4	HEIGHT	20.8	15		0	0
20	4832	48	Azithromycin	0	98	4/12/2019	126.7	HEIGHT	23.4	15		0	0
20	4832	60	Azithromycin	0	108	2/6/2020	131.8	HEIGHT	25.5	16.5		0	0
20	4835	0	Azithromycin	0	24	3/11/2015	81.7	HEIGHT	10.75	14.5		1	0
20	4835	48	Azithromycin	0	73	4/12/2019	109.9	HEIGHT	17.2	14		0	0
20	4835	60	Azithromycin	0	83	2/6/2020	114.8	HEIGHT	18.9	15		0	0
20	4836	0	Azithromycin	1	36	3/11/2015	97.5	HEIGHT	16.4	17.1		0	0
20	4836	12	Azithromycin	1	50	5/13/2016	104.5	HEIGHT	17.4	16.5		0	0
20	4836	24	Azithromycin	1	68	3/17/2017	111.9	HEIGHT	19.9	15.7		0	0

20	4836	36	Azithromycin	1	82	5/14/2018	119	HEIGHT	20.75	16		0	0
20	4839	0	Azithromycin	1	2	3/11/2015	59.9	LENGTH	4.9	10.9		0	0
20	4839	12	Azithromycin	1	14	5/13/2016	75.8	LENGTH	7.75	12.5		0	0
20	4839	24	Azithromycin	1	25	3/17/2017	85	HEIGHT	9.95	13		1	0
20	4841	12	Azithromycin	1	8	5/13/2016	72	LENGTH	7.3	13.5		1	0
20	4841	24	Azithromycin	1	14	3/17/2017	83	HEIGHT	10.3	13.4		1	0
20	4841	36	Azithromycin	1	36	5/14/2018	92.3	HEIGHT	12.6	14.7		1	0
20	4841	48	Azithromycin	1	46	4/12/2019	100.2	HEIGHT	15	14.9		0	0
20	4843	0	Azithromycin	0	48	3/11/2015	99.3	HEIGHT	15.3	13.9		0	0
20	4843	36	Azithromycin	0	98	5/14/2018	116	HEIGHT	20.35	16.5		0	0
20	4843	48	Azithromycin	0	109	4/12/2019	121	HEIGHT	21.6	16.6		0	0
20	4843	60	Azithromycin	0	119	2/6/2020	123.5	HEIGHT	23.3	18.1		0	0
20	4844	0	Azithromycin	0	24	3/11/2015	75.8	LENGTH	9.3	13.6		1	0
20	4844	12	Azithromycin	0	36	6/12/2016	86.5	HEIGHT	11.7	13.5		0	0
20	4844	24	Azithromycin	0	47	3/17/2017	92	HEIGHT	12.95	14		1	0
20	4844	36	Azithromycin	0	50	5/14/2018	99.3	HEIGHT	14.13636364	15.1		1	0
20	4844	48	Azithromycin	0	61	4/12/2019	104.8	HEIGHT	15.35	13.9		0	0
20	4845	0	Azithromycin	1	6	3/11/2015	66.6	LENGTH	7.35	13.5	24	0	0
20	4845	12	Azithromycin	1	24	5/13/2016	74.6	LENGTH	8.1	13	24	1	0
20	4846	0	Azithromycin	1	48	3/11/2015	107.6	HEIGHT	16.35	15.3		0	0
20	4846	24	Azithromycin	1	77	3/17/2017	119	HEIGHT	18.55	14		0	0
20	4846	36	Azithromycin	1	92	5/14/2018	124	HEIGHT	20.1	14.1		0	0
20	4846	48	Azithromycin	1	103	4/12/2019	127.9	HEIGHT	21.85	14.6		0	0
20	4848	24	Azithromycin	0	12	3/17/2017	74.3	LENGTH	9.55	15.3		1	0
20	4848	36	Azithromycin	0	26	5/14/2018	81.1	HEIGHT	11.55	14.5		0	0
20	4848	48	Azithromycin	0	37	4/12/2019	89.5	HEIGHT	13.45	15		0	0
20	4852	0	Azithromycin	0	55	3/11/2015	96.1	HEIGHT	13.8	15.3		1	0
20	4852	12	Azithromycin	0	71	5/13/2016	104	HEIGHT	14.15	15.5		0	0
20	4852	24	Azithromycin	0	82	3/17/2017	106	HEIGHT	15.6	14.3		0	0
20	4852	36	Azithromycin	0	96	5/14/2018	111.9	HEIGHT	17.95	15		0	0
20	4852	48	Azithromycin	0	107	4/12/2019	116.4	HEIGHT	19.25	15.5		0	0
20	4852	60	Azithromycin	0	117	2/6/2020	121	HEIGHT	21.15	15.5		0	0
20	4854	0	Azithromycin	0	18	3/11/2015	71.3	LENGTH	7.4	13.4		1	0
20	4854	24	Azithromycin	0	43	3/17/2017	86.5	HEIGHT	11.5	14.5		1	0
20	4854	36	Azithromycin	0	57	5/14/2018	93.9	HEIGHT	11.55	12.5		0	0
20	4854	60	Azithromycin	0	72	2/6/2020	103.7	HEIGHT	15.3	14.5		0	0
20	4856	0	Azithromycin	1	30	3/11/2015	90.1	HEIGHT	13.25	15.5		0	0
20	4856	12	Azithromycin	1	42	6/12/2016	99.3	HEIGHT	15.5	14		0	0
20	4856	24	Azithromycin	1	64	3/17/2017	105	HEIGHT	15.75	13		0	0
20	4856	36	Azithromycin	1	79	5/14/2018	111	HEIGHT	17.15	12.7		0	0

20	4856	48	Azithromycin	1	89	4/12/2019	116.4	HEIGHT	19.15	13.7	0	0
20	4856	60	Azithromycin	1	99	2/6/2020	120.9	HEIGHT	21.5	15.3	0	0
20	4859	24	Azithromycin	0	28	3/17/2017	82.5	HEIGHT	10.85	13.5	1	0
20	4861	0	Azithromycin	1	36	6/2/2015	105.4	HEIGHT	15	15.5	1	0
20	4861	36	Azithromycin	1	98	5/14/2018	124.4	HEIGHT	20.28	15.5	0	0
20	4861	48	Azithromycin	1	109	4/12/2019	129.4	HEIGHT	22.35	16	0	0
20	4861	60	Azithromycin	1	119	2/6/2020	134.9	HEIGHT	26	18	0	0
20	4863	12	Azithromycin	0	14	5/13/2016	80.4	LENGTH	11.1	17.5	1	0
20	4863	24	Azithromycin	0	25	3/17/2017	87.2	HEIGHT	13.7	16.9	1	0
20	4863	36	Azithromycin	0	40	5/14/2018	99.3	HEIGHT	16.40909091	16.8	1	0
20	4863	48	Azithromycin	0	51	4/12/2019	106.4	HEIGHT	18.4	16.2	1	0
20	4863	60	Azithromycin	0	61	2/6/2020	111.3	HEIGHT	19.2	16	0	0
20	4867	0	Azithromycin	0	48	3/11/2015	100.5	HEIGHT	17.45	16.5	1	0
20	4867	12	Azithromycin	0	54	6/12/2016	110	HEIGHT	20.55	16.5	0	0
20	4867	24	Azithromycin	0	65	3/17/2017	115.5	HEIGHT	21.95	16.5	0	0
20	4867	36	Azithromycin	0	108	5/14/2018	122.5	HEIGHT	22.45	15.7	0	0
20	4867	48	Azithromycin	0	118	4/12/2019	127	HEIGHT	26.85	17.5	0	0
20	4867	60	Azithromycin	0	128	2/6/2020	131.9	HEIGHT	29.2	19.5	0	0
20	4869	24	Azithromycin	1	7	3/17/2017	67.9	LENGTH	8.05	14.5	1	0
20	4869	36	Azithromycin	1	21	5/14/2018	79	HEIGHT	10.15	13.5	1	0
20	4869	48	Azithromycin	1	32	4/12/2019	87.8	HEIGHT	12.3	13.3	0	0
20	4870	0	Azithromycin	1	36	3/11/2015	78.6	HEIGHT	8.75	13.6	0	0
20	4870	12	Azithromycin	1	50	6/12/2016	87.3	HEIGHT	9.7	12.5	0	0
20	4870	24	Azithromycin	1	61	3/17/2017	94.3	HEIGHT	10.8	12.5	0	0
20	4870	36	Azithromycin	1	75	5/14/2018	100.5	HEIGHT	11.6	12.2	0	0
20	4870	48	Azithromycin	1	86	4/12/2019	105.4	HEIGHT	12.8	12.8	0	0
20	4874	12	Azithromycin	0	7	5/13/2016	70.9	LENGTH	7.95	14	0	0
20	4874	24	Azithromycin	0	18	3/17/2017	81.4	HEIGHT	10.4	14.8	0	0
20	4874	36	Azithromycin	0	32	5/14/2018	92.3	HEIGHT	13.5	16.1	1	0
20	4874	48	Azithromycin	0	43	4/12/2019	98.9	HEIGHT	15	15	1	0
20	4874	60	Azithromycin	0	53	2/6/2020	105.1	HEIGHT	16.1	15	1	0
20	4877	12	Azithromycin	0	20	5/13/2016	73.7	LENGTH	9	14.5	0	0
20	4877	36	Azithromycin	0	50	5/14/2018	90	HEIGHT	13.4	15	1	0
20	4879	0	Azithromycin	1	20	3/11/2015	77.4	HEIGHT	8.75	13.4	1	0
20	4879	12	Azithromycin	1	36	6/12/2016	85.5	HEIGHT	10.8	13.5	1	0
20	4879	36	Azithromycin	1	62	5/14/2018	98.9	HEIGHT	13.4	14	0	0
20	4879	48	Azithromycin	1	73	4/12/2019	103.8	HEIGHT	14.45	14	0	0
20	4879	60	Azithromycin	1	83	2/6/2020	107.1	HEIGHT	15.9	15	0	0
20	4882	0	Azithromycin	0	42	3/11/2015	89.1	HEIGHT	12.55	17	1	0
20	4882	12	Azithromycin	0	36	5/13/2016	95.6	LENGTH	14.1	16	1	0

20	4882	24	Azithromycin	0	47	3/17/2017	102.4	HEIGHT	15	14.9	1	0
20	4882	36	Azithromycin	0	96	5/14/2018	110	HEIGHT	15.4	13.3	0	0
20	4882	48	Azithromycin	0	106	4/12/2019	116.5	HEIGHT	19.3	14.4	0	0
20	4882	60	Azithromycin	0	116	2/6/2020	121.9	HEIGHT	22	15.5	0	0
20	4884	0	Azithromycin	1	18	3/11/2015	84.5	HEIGHT	12	14.7	1	0
20	4884	12	Azithromycin	1	49	5/13/2016	95.1	HEIGHT	14.55	15.5	0	0
20	4884	24	Azithromycin	1	61	3/17/2017	103.5	HEIGHT	17	16.5	0	0
20	4884	36	Azithromycin	1	68	5/14/2018	113	HEIGHT	19.7	16.2	0	0
20	4884	48	Azithromycin	1	79	4/12/2019	119.5	HEIGHT	21.7	16.5	0	0
20	4887	24	Azithromycin	0	4	3/17/2017	63.3	LENGTH	6.1	13	1	0
20	4887	60	Azithromycin	1	39	2/6/2020	89.9	HEIGHT	13.5	15	1	0
20	4890	12	Azithromycin	0	9	5/13/2016	74.8	LENGTH	9.3	14.5	1	0
20	4890	24	Azithromycin	0	20	3/17/2017	85.2	HEIGHT	12.2	15.5	1	0
20	4890	36	Azithromycin	0	34	5/14/2018	94.1	HEIGHT	14.55	15.3	1	0
20	4890	48	Azithromycin	0	45	4/12/2019	103.8	HEIGHT	17.4	16.3	0	0
20	4893	12	Azithromycin	1	12	5/13/2016	71.2	LENGTH	7.9	16	1	0
20	4893	36	Azithromycin	1	37	5/14/2018	86.9	HEIGHT	11.8	14.8	1	0
20	4895	24	Azithromycin	0	23	3/17/2017	75.5	HEIGHT	8.2	12.5	1	0
20	4895	36	Azithromycin	0	37	5/14/2018	83	HEIGHT	10.5	14	1	0
20	4896	0	Azithromycin	0	36	3/11/2015	97.4	HEIGHT	15.1	16.1	1	0
20	4896	12	Azithromycin	0	54	5/13/2016	105.3	HEIGHT	15.95	15.5	1	0
20	4896	24	Azithromycin	0	72	3/17/2017	112	HEIGHT	17.95	15	0	0
20	4896	60	Azithromycin	0	107	2/6/2020	129.4	HEIGHT	25.3	18	0	0
20	4897	0	Azithromycin	0	48	6/3/2015	93.8	HEIGHT	15.1	16.2	1	0
20	4897	12	Azithromycin	0	61	5/13/2016	99.4	HEIGHT	16.5	15	0	0
20	4897	24	Azithromycin	0	73	3/17/2017	104.9	HEIGHT	17.8	15	0	0
20	4897	36	Azithromycin	0	87	5/14/2018	111	HEIGHT	19.4	15.3	0	0
20	4897	48	Azithromycin	0	98	4/12/2019	116.9	HEIGHT	19.6	15	0	0
20	4897	60	Azithromycin	0	108	2/6/2020	123	HEIGHT	23.8	17	0	0
20	4898	0	Azithromycin	1	22	3/11/2015	88.2	HEIGHT	11.6	13.8	0	0
20	4898	24	Azithromycin	1	65	3/17/2017	103	HEIGHT	15.05	13	0	0
20	4898	36	Azithromycin	1	80	5/14/2018	109.9	HEIGHT	16.35	14.4	0	0
20	4898	48	Azithromycin	1	91	4/12/2019	115.4	HEIGHT	17.95	14	0	0
20	4901	12	Azithromycin	0	38	5/13/2016	98.9	LENGTH	14.85	17	1	0
20	4901	36	Azithromycin	0	63	5/14/2018	112.5	HEIGHT	17.4	14.5	0	0
20	4901	48	Azithromycin	0	76	4/12/2019	118.9	HEIGHT	20.15	15	0	0
20	4901	60	Azithromycin	0	86	2/6/2020	122.9	HEIGHT	20.9	15	0	0
20	4902	0	Azithromycin	0	36	3/11/2015	91.5	HEIGHT	11.8	14.6	0	0
20	4902	12	Azithromycin	0	51	5/13/2016	98.4	HEIGHT	12.15	13.5	0	0
20	4902	24	Azithromycin	0	61	3/17/2017	104.3	LENGTH	13.65	13.9	0	0

20	4902	36	Azithromycin	0	75	5/14/2018	111	HEIGHT	15.25	13.5		0	0
20	4902	48	Azithromycin	0	86	4/12/2019	115.4	HEIGHT	17.15	13.3		0	0
20	4902	60	Azithromycin	0	96	2/6/2020	120.6	HEIGHT	19.4	14.5		0	0
20	4903	0	Azithromycin	0	17	3/11/2015	82.4	HEIGHT	12.1	15.5		0	0
20	4903	12	Azithromycin	0	32	5/13/2016	92.3	LENGTH	14.45	16.5		1	0
20	4903	24	Azithromycin	0	43	3/17/2017	101	HEIGHT	17	16.5		0	0
20	4903	36	Azithromycin	0	58	5/14/2018	107.5	HEIGHT	18.25	14.6		1	0
20	4903	48	Azithromycin	0	69	4/12/2019	113.4	HEIGHT	19.45	14.5		0	0
20	4904	0	Azithromycin	0	48	3/11/2015	110.4	HEIGHT	17.45	15.6		1	0
20	4904	12	Azithromycin	0	62	5/13/2016	117.8	HEIGHT	19.85	15.8		0	0
20	4904	24	Azithromycin	0	73	3/17/2017	123.8	HEIGHT	21.4	16		0	0
20	4907	12	Azithromycin	0	33	5/13/2016	83	HEIGHT	10.85	14		1	0
20	4907	36	Azithromycin	0	59	5/14/2018	101	HEIGHT	14.3	14.3		1	0
20	4908	12	Azithromycin	0	11	5/13/2016	69	LENGTH	7.35	15		0	0
20	4908	24	Azithromycin	0	20	3/17/2017	79.2	HEIGHT	9.45	13.5		1	0
20	4908	36	Azithromycin	0	36	5/14/2018	85.5	HEIGHT	11.35	14.1		1	0
20	4908	48	Azithromycin	0	47	4/12/2019	90.3	HEIGHT	12.8	14		1	0
20	4908	60	Azithromycin	0	56	2/6/2020	78.4	HEIGHT	8.6	12		1	0
20	4910	0	Azithromycin	1	42	3/11/2015	90.4	HEIGHT	13.55	15.5		0	0
20	4910	12	Azithromycin	1	56	5/13/2016	96.3	HEIGHT	15.15	16		1	0
20	4912	12	Azithromycin	1	4	5/13/2016	60.4	LENGTH	5.25	13		1	0
20	4912	36	Azithromycin	1	28	5/14/2018	86	HEIGHT	9.9	13.7		0	0
20	4914	0	Azithromycin	1	12	3/11/2015	78.8	LENGTH	9.5	14.8		1	0
20	4914	12	Azithromycin	1	31	6/12/2016	91.5	HEIGHT	13.2	15		0	0
20	4914	24	Azithromycin	1	60	3/17/2017	98.2	HEIGHT	15.1	16		0	0
20	4914	36	Azithromycin	1	74	5/14/2018	108	HEIGHT	17.8	15.5		0	0
20	4914	48	Azithromycin	1	85	4/12/2019	113.8	HEIGHT	18.65	15.7		0	0
20	4915	0	Azithromycin	1	31	3/11/2015	74.4	LENGTH	8.2	13.2		0	0
20	4915	12	Azithromycin	1	47	5/13/2016	81	HEIGHT	9.85	14.5		1	0
20	4915	36	Azithromycin	1	73	5/14/2018	97	HEIGHT	13	13.5		0	0
20	4915	48	Azithromycin	1	83	4/12/2019	103	HEIGHT	14.25	14		0	0
20	4916	0	Azithromycin	0	54	3/11/2015	108.1	HEIGHT	15.2	17		1	0
20	4916	36	Azithromycin	0	114	5/14/2018	125.8	HEIGHT	21.7	15.2		0	0
20	4916	48	Azithromycin	0	124	4/12/2019	130.9	HEIGHT	23	16		0	0
20	4921	0	Azithromycin	0	48	6/3/2015	109.5	HEIGHT	17.55	15.5		1	0
20	4921	36	Azithromycin	0	87	5/14/2018	127.4	HEIGHT	21	14		0	0
20	4921	48	Azithromycin	0	98	4/12/2019	131	HEIGHT	26.05	16		0	0
20	4921	60	Azithromycin	0	108	2/6/2020	136.1	HEIGHT	28.3	16.7		0	0
20	4922	0	Azithromycin	1	36	3/11/2015	112.9	HEIGHT	17.7	15.4		0	0
20	4922	36	Azithromycin	1	109	5/14/2018	122	HEIGHT	18.95	15		0	0

20	4923	12	Azithromycin	1	5	5/13/2016	63.6	LENGTH	6.15	13		0	0
20	4923	24	Azithromycin	1	16	3/17/2017	74.7	HEIGHT	8.9	13.3		1	0
20	4923	36	Azithromycin	1	31	5/14/2018	86	HEIGHT	11.6	14.4		1	0
20	4923	48	Azithromycin	1	42	4/12/2019	93.9	HEIGHT	13.4	14.5		1	0
20	4927	12	Azithromycin	1	6	5/15/2016	71.8	LENGTH	9.35	15		1	0
20	4929	12	Azithromycin	0	20	5/13/2016	79.5	LENGTH	9	15		0	0
20	4929	24	Azithromycin	0	36	3/17/2017	84.8	LENGTH	9.9	13.5		1	0
20	4929	36	Azithromycin	0	48	5/14/2018	90	HEIGHT	11.5	13.7		1	0
20	4930	12	Azithromycin	0	56	5/13/2016	103	HEIGHT	14.15	14		1	0
20	4932	12	Azithromycin	1	1	5/13/2016	63.3	LENGTH	7.85	17		0	0
20	4932	24	Azithromycin	0	12	3/17/2017	78.8	LENGTH	10.15	14.5		1	0
20	4932	36	Azithromycin	1	26	5/14/2018	87.5	HEIGHT	13.55	16		1	0
20	4932	48	Azithromycin	0	37	4/12/2019	95.8	HEIGHT	16.75	16		1	0
20	4932	60	Azithromycin	0	47	2/6/2020	103.4	HEIGHT	17.9	16.2		0	0
20	4934	12	Azithromycin	0	14	6/12/2016	71.8	LENGTH	8.25	14	60	1	0
20	4934	24	Azithromycin	0	25	3/17/2017	81.4	HEIGHT	10.35	14.1	60	1	0
20	4934	48	Azithromycin	0	47	4/12/2019	101.6	HEIGHT	14.5	13.8	60	1	0
20	4935	0	Azithromycin	0	48	3/11/2015	113.4	HEIGHT	23.25	18.8		0	0
20	4935	12	Azithromycin	0	60	6/12/2016	119.4	HEIGHT	24.7	18		0	0
20	4935	24	Azithromycin	0	72	3/17/2017	124	HEIGHT	26	17.7		0	0
20	4935	48	Azithromycin	0	97	4/12/2019	135.1	HEIGHT	32.65	19		0	0
20	4935	60	Azithromycin	0	107	2/6/2020	140	HEIGHT	36	25		0	0
20	4936	12	Azithromycin	1	62	5/13/2016	102.5	HEIGHT	14.9	16.5		0	0
20	4937	24	Azithromycin	0	5	3/17/2017	71.5	LENGTH	7.65	13.4		1	0
20	4937	60	Azithromycin	0	35	2/6/2020	97	HEIGHT	13.9	13.7		0	0
20	4940	0	Azithromycin	1	25	3/11/2015	77.4	HEIGHT	8.9	14.7	18	1	0
20	4940	12	Azithromycin	1	42	5/13/2016	84.2	HEIGHT	10.6	15	18	1	0
20	4942	12	Azithromycin	0	5	5/13/2016	65.3	LENGTH	5.85	12		0	0
20	4942	24	Azithromycin	0	14	3/17/2017	74.6	LENGTH	7.65	12.7		1	0
20	4942	36	Azithromycin	0	26	5/14/2018	84.9	HEIGHT	9.545454545	13		1	0
20	4942	48	Azithromycin	0	37	4/12/2019	90.8	HEIGHT	11.2	12.5		1	0
20	4946	0	Azithromycin	1	8	3/11/2015	71.8	LENGTH	7.65	14		0	0
20	4946	12	Azithromycin	1	24	5/13/2016	84.1	LENGTH	10.6	13.9		1	0
20	4946	24	Azithromycin	1	35	3/17/2017	91.5	HEIGHT	13.25	15		0	0
20	4946	60	Azithromycin	1	71	2/6/2020	110.4	HEIGHT	18.5	17		0	0
20	4947	0	Azithromycin	0	24	3/11/2015	79	LENGTH	8.8	13.4		1	0
20	4947	12	Azithromycin	0	36	5/13/2016	92.1	LENGTH	12.35	15		1	0
20	4947	24	Azithromycin	0	47	3/17/2017	98	HEIGHT	13.25	13.5		1	0
20	4947	36	Azithromycin	0	59	5/14/2018	104	HEIGHT	16.2	14.6		1	0
20	4947	48	Azithromycin	0	70	4/12/2019	108.4	HEIGHT	16.45	13.3		0	0

20	4947	60	Azithromycin	0	80	2/6/2020	114.4	HEIGHT	17.9	14		0	0
20	4948	0	Azithromycin	0	48	3/11/2015	104.6	HEIGHT	17.4	15.7		1	0
20	4948	12	Azithromycin	0	62	5/13/2016	111.1	HEIGHT	19.45	16.5		0	0
20	4948	24	Azithromycin	0	73	3/17/2017	118	HEIGHT	22.15	16.5		0	0
20	4948	36	Azithromycin	0	96	5/14/2018	124	HEIGHT	23.75	16.7		0	0
20	4948	48	Azithromycin	0	106	4/12/2019	129.9	HEIGHT	27.85	17.2		0	0
20	4951	0	Azithromycin	0	24	3/11/2015	65.2	LENGTH	6.9	13.6		1	0
20	4951	12	Azithromycin	0	18	5/13/2016	78	LENGTH	9.15	15.7		1	0
20	4951	24	Azithromycin	0	29	3/17/2017	84.6	HEIGHT	11.35	14.2		1	0
20	4956	0	Azithromycin	0	24	3/11/2015	77.6	LENGTH	9.05	13.8		1	0
20	4956	24	Azithromycin	0	49	3/17/2017	92	HEIGHT	14.7	15		1	0
20	4956	36	Azithromycin	0	63	5/14/2018	102	HEIGHT	15.3	14.5		0	0
20	4956	48	Azithromycin	0	74	4/12/2019	108.1	HEIGHT	19.15	16.4		0	0
20	4956	60	Azithromycin	0	84	2/6/2020	113.6	HEIGHT	20.5	16.5		0	0
20	4957	12	Azithromycin	1	7	5/15/2016	65.5	LENGTH	6.55	12		0	0
20	4960	12	Azithromycin	1	8	5/13/2016	68.2	LENGTH	8.35	16.9		0	0
20	4960	24	Azithromycin	1	20	3/17/2017	77.4	HEIGHT	10.35	15.1		1	0
20	4960	36	Azithromycin	1	34	5/14/2018	87.5	HEIGHT	12.9	15		1	0
20	4960	48	Azithromycin	1	45	4/12/2019	94.8	HEIGHT	13.8	14.5		1	0
20	4963	24	Azithromycin	1	23	3/17/2017	84.2	HEIGHT	10.1	13.5		1	0
20	4963	48	Azithromycin	1	48	4/14/2019	99.5	HEIGHT	13.15	14.1		1	0
20	4965	0	Azithromycin	0	6	3/11/2015	87.6	HEIGHT	13.2	17		1	0
20	4965	24	Azithromycin	0	31	3/17/2017	105.3	HEIGHT	16.85	16		1	0
20	4965	36	Azithromycin	0	49	5/14/2018	102.6	HEIGHT	15.27272727	14.6		1	0
20	4965	48	Azithromycin	0	60	4/12/2019	108.3	HEIGHT	17.7	14.5		0	0
20	4965	60	Azithromycin	0	70	2/6/2020	112.7	HEIGHT	18.9	14.5		0	0
20	4968	0	Azithromycin	0	31	3/11/2015	93.3	HEIGHT	14.15	14.9		1	0
20	4968	12	Azithromycin	0	42	5/13/2016	104.5	HEIGHT	15.8	16.5		0	0
20	4968	24	Azithromycin	0	58	3/17/2017	110	HEIGHT	17.65	15.5		1	0
20	4968	36	Azithromycin	0	72	5/14/2018	115.5	HEIGHT	19.65	14.8		0	0
20	4968	48	Azithromycin	0	83	4/12/2019	120.7	HEIGHT	22.15	15		0	0
20	4968	60	Azithromycin	0	92	2/6/2020	124.6	HEIGHT	22.6	15.5		0	0
20	4970	0	Azithromycin	1	4	3/11/2015	61.6	LENGTH	5.7	12.5	30	0	0
20	4970	12	Azithromycin	1	14	5/13/2016	75	HEIGHT	8.25	14	30	1	0
20	4972	12	Azithromycin	1	11	5/13/2016	67.1	LENGTH	5.7	11.5		0	0
20	4973	12	Azithromycin	1	25	5/13/2016	78.7	LENGTH	8.8	13		0	0
20	4974	0	Azithromycin	0	6	3/11/2015	71.9	LENGTH	9.2	15.3	18	1	0
20	4974	12	Azithromycin	0	20	5/13/2016	79.9	LENGTH	10.9	15.5	18	1	0
20	4976	0	Azithromycin	0	48	3/11/2015	97.7	HEIGHT	14.25	15.1		0	0
20	4976	24	Azithromycin	0	65	3/17/2017	109.7	HEIGHT	17.25	14.5		0	0

20	4976	36	Azithromycin	0	80	5/14/2018	116	HEIGHT	18.6	14.2	0	0
20	4976	48	Azithromycin	0	91	4/12/2019	119.9	HEIGHT	20.5	15	0	0
20	4976	60	Azithromycin	0	100	2/6/2020	124.3	HEIGHT	23.1	15.7	0	0
20	4977	12	Azithromycin	1	4	5/13/2016	60.9	LENGTH	5.9	14	1	0
20	4977	36	Azithromycin	1	29	5/14/2018	82.5	HEIGHT	10.75	13.5	0	0
20	4977	48	Azithromycin	1	40	4/12/2019	88	HEIGHT	11.9	14	1	0
20	4977	60	Azithromycin	1	50	2/6/2020	93.5	HEIGHT	14.1	14.7	1	0
20	4978	12	Azithromycin	1	20	5/13/2016	82.2	LENGTH	10.95	16	1	0
20	4978	24	Azithromycin	1	31	3/17/2017	88.5	HEIGHT	13.4	17	1	0
20	4978	36	Azithromycin	1	46	5/14/2018	99.3	LENGTH	16.15	17	1	0
20	4978	48	Azithromycin	1	57	4/12/2019	107.1	HEIGHT	17.15	16.2	1	0
20	4980	0	Azithromycin	0	36	3/11/2015	95.9	HEIGHT	16.25	16.5	0	0
20	4986	12	Azithromycin	0	2	5/13/2016	55.4	LENGTH	5.85	13	1	0
20	4986	24	Azithromycin	0	12	3/17/2017	71	LENGTH	7.65	12.5	0	0
20	4986	36	Azithromycin	0	26	5/14/2018	78.8	LENGTH	10.3	14	1	0
20	4986	60	Azithromycin	0	47	2/6/2020	93.5	LENGTH	15	15.2	1	0
20	4987	0	Azithromycin	1	54	3/11/2015	103	HEIGHT	14.9	15	0	0
20	4987	12	Azithromycin	1	66	6/12/2016	109.9	HEIGHT	16.9	15	0	0
20	4987	36	Azithromycin	1	110	5/14/2018	122	HEIGHT	20.05	14	0	0
20	4987	48	Azithromycin	1	118	4/12/2019	125.7	HEIGHT	23.15	15	0	0
20	4988	0	Azithromycin	0	38	3/11/2015	87.7	HEIGHT	11.9	14.9	1	0
20	4988	12	Azithromycin	0	42	5/13/2016	94.9	HEIGHT	13.2	14.5	1	0
20	4988	24	Azithromycin	0	53	3/17/2017	102.4	HEIGHT	14.15	13.3	0	0
20	4988	36	Azithromycin	0	79	5/14/2018	107	HEIGHT	16.15	14	0	0
20	4988	48	Azithromycin	0	90	4/12/2019	111.8	HEIGHT	17.15	13.5	0	0
20	4988	60	Azithromycin	0	100	2/6/2020	116.2	HEIGHT	19.1	14.5	0	0
20	4990	24	Azithromycin	1	6	3/17/2017	64.9	LENGTH	8.3	15.5	1	0
20	4990	36	Azithromycin	1	19	5/14/2018	81	HEIGHT	12.27272727	16.5	1	0
20	4990	48	Azithromycin	1	30	4/12/2019	87.4	HEIGHT	13.15	14.6	1	0
20	4990	60	Azithromycin	1	39	2/6/2020	91.2	HEIGHT	13.3	15	1	0
20	4991	0	Azithromycin	0	48	3/11/2015	106.5	HEIGHT	15.85	15	1	0
20	4991	12	Azithromycin	0	63	5/13/2016	111.9	HEIGHT	17.65	15.5	0	0
20	4991	24	Azithromycin	0	73	3/17/2017	117	HEIGHT	19.9	16	0	0
20	4991	36	Azithromycin	0	87	5/14/2018	124	HEIGHT	20.35	16.5	0	0
20	4991	48	Azithromycin	0	98	4/12/2019	127.8	HEIGHT	24.1	16	0	0
20	4992	0	Azithromycin	1	12	3/11/2015	67.5	LENGTH	6.8	13.2	1	0
20	4992	24	Azithromycin	1	37	3/17/2017	87.2	HEIGHT	12.35	16	1	0
20	4992	36	Azithromycin	1	51	5/14/2018	96	HEIGHT	14.68181818	17	1	0
20	4992	48	Azithromycin	1	62	4/12/2019	102.8	HEIGHT	16.5	16.5	0	0
20	4992	60	Azithromycin	1	72	2/6/2020	107.9	HEIGHT	18.1	17	0	0

20	4994	0	Azithromycin	1	40	3/11/2015	85.6	HEIGHT	11.65	14.8		1	0
20	4994	12	Azithromycin	1	56	5/13/2016	96	HEIGHT	13.2	15.5		1	0
20	4994	24	Azithromycin	1	67	3/17/2017	104	HEIGHT	16.65	15.8		0	0
20	4994	36	Azithromycin	1	81	5/14/2018	112	HEIGHT	18.5	15		0	0
20	4994	48	Azithromycin	1	92	4/12/2019	116.8	HEIGHT	19.55	15.5		0	0
20	4994	60	Azithromycin	1	102	2/6/2020	121.2	HEIGHT	21.7	16.4		0	0
20	4998	0	Azithromycin	0	30	3/11/2015	96.5	HEIGHT	14.6	15.7		1	0
20	4998	12	Azithromycin	0	54	6/12/2016	103.5	HEIGHT	15.95	14.5		0	0
20	4998	36	Azithromycin	0	80	5/14/2018	114.5	HEIGHT	19.6	15		0	0
20	4998	48	Azithromycin	0	91	4/12/2019	118.9	HEIGHT	21.1	15.5		0	0
20	4998	60	Azithromycin	0	100	2/6/2020	122.1	HEIGHT	23.3	15.9		0	0
20	4999	0	Azithromycin	0	18	3/11/2015	77.5	LENGTH	11.15	18		0	0
20	4999	12	Azithromycin	0	32	5/13/2016	87.1	HEIGHT	10.56	16		0	0
20	4999	24	Azithromycin	0	43	3/17/2017	92.5	HEIGHT	15.1	17		1	0
20	4999	36	Azithromycin	0	56	5/14/2018	100.4	HEIGHT	16.15	15.5		1	0
20	4999	48	Azithromycin	0	67	4/12/2019	107.1	HEIGHT	18.1	16.3		0	0
20	5003	0	Azithromycin	1	48	3/11/2015	88.4	HEIGHT	12.4	14.5		0	0
20	5003	12	Azithromycin	1	62	5/13/2016	96.1	HEIGHT	14.85	15		0	0
20	5003	24	Azithromycin	1	73	3/17/2017	101.8	HEIGHT	16.15	15		0	0
20	5003	36	Azithromycin	1	87	5/14/2018	108.5	HEIGHT	19.35	16		0	0
20	5003	48	Azithromycin	1	98	4/12/2019	115	HEIGHT	21.95	16.2		0	0
20	8072	60	Azithromycin	0	11	2/6/2020	71	LENGTH	8	13.5		1	0
20	8152	36	Azithromycin	0	10	5/14/2018	71.3	LENGTH	8.25	13.5		1	0
20	8160	60	Azithromycin	0	8	2/6/2020	68.6	LENGTH	7.7	13.2		1	0
20	8207	48	Azithromycin	1	2	4/14/2019	58	LENGTH	6.1	14.5		1	0
20	8281	60	Azithromycin	1	37	2/6/2020	86.1	HEIGHT	11.9	14.5		1	0
20	8293	48	Azithromycin	0	13	4/12/2019	66.7	LENGTH	5.5	9.4		1	0
20	8362	36	Azithromycin	0	36	5/14/2018	93.3	HEIGHT	17.04545455	17.1		1	0
20	8362	48	Azithromycin	0	47	4/14/2019	99.8	HEIGHT	19.15	18.5		1	0
20	8381	48	Azithromycin	1	8	4/12/2019	71.5	LENGTH	7.55	12.9		1	0
20	8381	60	Azithromycin	1	18	2/6/2020	80.7	HEIGHT	9.5	14		1	0
20	8387	60	Azithromycin	0	10	2/6/2020	66.5	LENGTH	7.3	12.5		1	0
20	8482	36	Azithromycin	0	2	5/14/2018	59.3	LENGTH	6.05	13		1	0
20	8482	48	Azithromycin	0	13	4/12/2019	71.9	LENGTH	7.75	13		1	0
20	8482	60	Azithromycin	0	23	2/6/2020	95.7	HEIGHT	14.2	14.7		0	0
20	8502	36	Azithromycin	0	8	5/14/2018	66.7	LENGTH	6.4	12.5	48	1	0
20	8564	36	Azithromycin	1	12	5/14/2018	72	LENGTH	7.909090909	13.6		1	0
20	8564	48	Azithromycin	1	22	4/12/2019	78.3	LENGTH	9.35	13		1	0
20	8564	60	Azithromycin	1	32	2/6/2020	85.3	HEIGHT	11.9	14		1	0
20	8602	60	Azithromycin	0	2	2/6/2020	58.9	HEIGHT	4.9	13		1	0

20	8616	48	Azithromycin	1	1	4/14/2019	54.4	LENGTH	3.9	10		1	0
20	8618	48	Azithromycin	0	9	4/12/2019	70.3	LENGTH	7.9	12.5		1	0
20	8640	36	Azithromycin	0	10	5/14/2018	72.5	LENGTH	7.95	13.6		1	0
20	8647	48	Azithromycin	1	12	4/14/2019	63.7	LENGTH	5.75	11.3		1	0
20	8648	36	Azithromycin	1	10	5/14/2018	67.4	LENGTH	7.227272727	13.2		1	0
20	8648	48	Azithromycin	1	21	4/12/2019	73.3	LENGTH	7.8	11.2		1	0
20	8664	48	Azithromycin	0	16	4/12/2019	75	HEIGHT	9.55	14		1	0
20	8706	36	Azithromycin	0	2	5/14/2018	58.1	LENGTH	4.318181818	10.5		1	0
20	8724	48	Azithromycin	0	12	4/12/2019	73.3	LENGTH	8.7	13		1	0
20	8724	60	Azithromycin	0	22	2/6/2020	80.2	HEIGHT	10.8	13.9		1	0
20	8750	48	Azithromycin	1	34	4/12/2019	82.5	HEIGHT	9.45	11.6		1	0
20	8751	48	Azithromycin	0	12	4/12/2019	72.2	LENGTH	7.25	11.5		1	0
20	8798	36	Azithromycin	0	33	5/14/2018	92.5	HEIGHT	14.6	16.3		1	0
20	8798	48	Azithromycin	0	44	4/12/2019	99.5	HEIGHT	16.35	16		1	0
20	8891	36	Azithromycin	0	2	5/14/2018	59.3	LENGTH	5.25	11	60	1	0
20	8891	48	Azithromycin	0	13	4/12/2019	68.8	LENGTH	7.25	11.3	60	1	0
20	8937	36	Azithromycin	1	117	5/14/2018	132.4	HEIGHT	22.55	14		0	0
20	8937	48	Azithromycin	1	128	4/12/2019	134	HEIGHT	24.7	16.5		0	0
20	8937	60	Azithromycin	1	138	2/6/2020	139.1	HEIGHT	26.8	17.7		0	0
20	8963	48	Azithromycin	0	8	4/14/2019	65.6	LENGTH	7.85	15		1	0
20	8979	36	Azithromycin	1	26	5/14/2018	80.5	HEIGHT	10.5	14		1	0
20	8979	48	Azithromycin	1	37	4/12/2019	89.4	HEIGHT	12.5	15		1	0
20	9031	60	Azithromycin	1	2	2/6/2020	55.9	LENGTH	5.4	11.5		1	0
20	9102	60	Azithromycin	0	4	2/6/2020	60.1	LENGTH	5.1	10.5		1	0
20	9133	36	Azithromycin	0	1	5/14/2018	67.3	LENGTH	6.65	10.5		1	0
20	9149	36	Azithromycin	1	12	5/14/2018	70.8	LENGTH	7.75	13		1	0
20	9204	36	Azithromycin	0	14	5/14/2018	75.4	LENGTH	8.136363636	14.4	48	1	0
20	9253	36	Azithromycin	1	7	5/14/2018	66.3	LENGTH	7	12.5		1	0
20	9253	48	Azithromycin	1	15	4/12/2019	75.3	LENGTH	9.05	13		1	0
20	9253	60	Azithromycin	1	25	2/6/2020	80.4	HEIGHT	10.8	13.9		1	0
20	9299	48	Azithromycin	1	2	4/14/2019	56.8	LENGTH	5	13.5	60	1	0
20	9310	36	Azithromycin	1	48	5/14/2018	97.5	HEIGHT	12.55	13.4		1	0
20	9355	48	Azithromycin	0	2	4/12/2019	56.3	LENGTH	5.45	11.5		1	0
20	9359	48	Azithromycin	0	45	4/14/2019	91.2	HEIGHT	12.5	14.5		1	0
20	9359	60	Azithromycin	0	55	2/6/2020	96.3	HEIGHT	13	13.5		1	0
20	9401	36	Azithromycin	1	3	5/14/2018	61.3	LENGTH	6.15	13		1	0
20	9401	48	Azithromycin	1	14	4/12/2019	73.2	LENGTH	9.15	13.2		1	0
20	9496	36	Azithromycin	0	2	5/14/2018	58.4	LENGTH	5.5	13.6		1	0
20	9496	48	Azithromycin	0	13	4/12/2019	72.3	LENGTH	8.5	13.5		1	0
20	9561	48	Azithromycin	0	23	4/12/2019	79.2	LENGTH	9.6	13.4		1	0

21	5044	12	Azithromycin	0	8	6/15/2016	63.8	LENGTH	6.35	12.5		1	0
21	5044	24	Azithromycin	0	15	4/26/2017	72.3	HEIGHT	9.85	15.5		0	0
21	5044	36	Azithromycin	0	34	5/26/2018	83.7	HEIGHT	14.36363636	18.5		1	0
21	5044	48	Azithromycin	0	45	5/20/2019	93.2	HEIGHT	15.95454545	18		1	0
21	5044	60	Azithromycin	0	54	2/16/2020	99.7	HEIGHT	16.95	17.2		1	0
21	5045	12	Azithromycin	0	1	6/15/2016	61.6	LENGTH	6.3	13		0	0
21	5045	24	Azithromycin	0	12	4/26/2017	75.8	LENGTH	10.15	15		1	0
21	5045	36	Azithromycin	0	25	5/26/2018	83.4	HEIGHT	12.6	15		1	0
21	5045	48	Azithromycin	0	35	5/20/2019	90.9	HEIGHT	14.77272727	16		1	0
21	5045	60	Azithromycin	0	47	2/16/2020	97.5	HEIGHT	15.75	15.5		0	0
21	5047	12	Azithromycin	1	30	6/15/2016	92.2	HEIGHT	13.2	16		1	0
21	5047	24	Azithromycin	1	43	4/26/2017	99.3	HEIGHT	14.7	16.5		1	0
21	5052	0	Azithromycin	0	24	4/8/2015	83.7	HEIGHT	10.35	14.5		1	0
21	5052	24	Azithromycin	0	43	4/26/2017	100.2	HEIGHT	14.8	16		1	0
21	5052	36	Azithromycin	0	54	5/26/2018	104.9	HEIGHT	15.95454545	16		1	0
21	5052	48	Azithromycin	0	64	5/20/2019	111.2	HEIGHT	17.68181818	16.5		0	0
21	5052	60	Azithromycin	0	74	2/16/2020	118.1	HEIGHT	20.1	16.2		0	0
21	5053	12	Azithromycin	1	38	6/15/2016	87.7	HEIGHT	11.45	14.5		1	0
21	5053	48	Azithromycin	1	63	5/20/2019	106.2	HEIGHT	15.22727273	14		0	0
21	5054	0	Azithromycin	0	31	4/8/2015	88.5	HEIGHT	10.75	13		1	0
21	5056	12	Azithromycin	1	30	6/15/2016	85.2	HEIGHT	11.5	15	30	1	0
21	5057	24	Azithromycin	0	13	4/26/2017	71.1	LENGTH	7.85	13		1	0
21	5057	60	Azithromycin	0	46	2/16/2020	98.1	HEIGHT	14.65	15		1	0
21	5058	0	Azithromycin	0	43	4/8/2015	79.2	HEIGHT	9.55	14		1	0
21	5058	48	Azithromycin	0	89	5/27/2019	105.5	HEIGHT	15.81818182	15		0	0
21	5058	60	Azithromycin	0	98	2/16/2020	110.4	HEIGHT	17.5	15.4		0	0
21	5065	12	Azithromycin	1	12	6/15/2016	75.8	LENGTH	8.25	13		1	0
21	5065	24	Azithromycin	1	24	4/26/2017	82.8	HEIGHT	9.95	15		1	0
21	5065	36	Azithromycin	1	38	5/26/2018	93.3	HEIGHT	12.15	14		1	0
21	5067	24	Azithromycin	0	7	4/26/2017	66.4	LENGTH	6.95	13		1	0
21	5067	36	Azithromycin	0	15	5/26/2018	78.5	LENGTH	9.409090909	14		1	0
21	5067	48	Azithromycin	0	26	5/20/2019	85.4	HEIGHT	11	14		1	0
21	5067	60	Azithromycin	0	35	2/16/2020	91.7	HEIGHT	12.55	14.3		1	0
21	5069	12	Azithromycin	0	44	6/15/2016	92.4	HEIGHT	13.5	14.5		1	0
21	5069	24	Azithromycin	0	57	4/26/2017	98.5	HEIGHT	14.35	14		1	0
21	5069	48	Azithromycin	0	81	5/20/2019	111.4	HEIGHT	17.86363636	15		0	0
21	5070	0	Azithromycin	1	55	4/8/2015	95.5	HEIGHT	14.75	16		1	0
21	5070	12	Azithromycin	1	54	6/15/2016	101.9	HEIGHT	15.75	15.5		0	0
21	5070	24	Azithromycin	1	57	4/26/2017	107.6	HEIGHT	16.6	15		1	0
21	5070	36	Azithromycin	1	81	5/26/2018	114.4	HEIGHT	17.77272727	14.5		0	0

21	5070	48	Azithromycin	1	92	5/20/2019	117.9	HEIGHT	20	15	0	0
21	5070	60	Azithromycin	1	101	2/16/2020	120.9	HEIGHT	21.25	15.5	0	0
21	5073	0	Azithromycin	1	11	4/8/2015	71.2	LENGTH	7.55	13.5	1	0
21	5073	24	Azithromycin	1	31	4/26/2017	90.2	HEIGHT	12.05	14	1	0
21	5073	60	Azithromycin	0	64	2/16/2020	109.7	HEIGHT	15.65	14	0	0
21	5074	12	Azithromycin	1	12	6/15/2016	73	HEIGHT	8.55	14.5	1	0
21	5074	24	Azithromycin	1	24	4/26/2017	81.6	HEIGHT	11	15	1	0
21	5074	48	Azithromycin	1	48	5/20/2019	97.2	HEIGHT	14	14.5	1	0
21	5075	12	Azithromycin	1	38	6/15/2016	88.7	HEIGHT	13.05	17	1	0
21	5075	48	Azithromycin	1	75	5/20/2019	109.2	HEIGHT	18.59090909	16	0	0
21	5076	24	Azithromycin	0	13	4/26/2017	69.5	LENGTH	7.25	12	1	0
21	5076	60	Azithromycin	0	46	2/20/2020	95.5	HEIGHT	14.63636364	14.5	1	0
21	5077	24	Azithromycin	0	3	4/26/2017	61.8	LENGTH	6.3	13	1	0
21	5081	0	Azithromycin	1	36	4/8/2015	97.5	HEIGHT	14.6	16	1	0
21	5081	12	Azithromycin	1	54	6/15/2016	103.7	HEIGHT	15.8	16	1	0
21	5081	24	Azithromycin	1	67	4/26/2017	109.3	HEIGHT	16.7	16	0	0
21	5081	36	Azithromycin	1	78	5/26/2018	115.8	HEIGHT	18.27272727	16.5	0	0
21	5081	48	Azithromycin	1	89	5/20/2019	120.6	HEIGHT	19.77272727	16.5	0	0
21	5081	60	Azithromycin	1	98	2/16/2020	125	HEIGHT	21.4	16.1	0	0
21	5082	12	Azithromycin	1	50	6/15/2016	111	HEIGHT	18.1	16	1	0
21	5082	48	Azithromycin	1	113	5/20/2019	129.4	HEIGHT	26.6	18	0	0
21	5084	12	Azithromycin	0	20	6/15/2016	78.1	HEIGHT	9.85	14.5	1	0
21	5085	0	Azithromycin	0	55	4/8/2015	105.5	HEIGHT	17.2	16.5	1	0
21	5085	12	Azithromycin	0	63	6/15/2016	111.9	HEIGHT	19.7	16.5	0	0
21	5085	24	Azithromycin	0	76	4/26/2017	117.4	HEIGHT	20.7	17	0	0
21	5085	36	Azithromycin	0	89	5/26/2018	123.5	HEIGHT	22.31818182	15.5	0	0
21	5085	48	Azithromycin	0	100	5/20/2019	128.3	HEIGHT	26.22727273	17.5	0	0
21	5085	60	Azithromycin	0	110	2/16/2020	133.2	HEIGHT	27.75	17	0	0
21	5086	12	Azithromycin	0	50	6/15/2016	100.3	HEIGHT	15.65	15	1	0
21	5086	48	Azithromycin	0	87	5/20/2019	118.7	HEIGHT	19.86363636	15	0	0
21	5088	12	Azithromycin	1	10	6/15/2016	71.8	LENGTH	7.85	14.5	1	0
21	5088	24	Azithromycin	1	15	4/26/2017	81	HEIGHT	9.1	14	0	0
21	5088	36	Azithromycin	1	26	5/26/2018	90.5	HEIGHT	11.72727273	14	1	0
21	5088	60	Azithromycin	1	46	2/16/2020	103.1	HEIGHT	14.95	14	1	0
21	5090	12	Azithromycin	1	26	6/15/2016	84.9	HEIGHT	10.9	15	1	0
21	5090	24	Azithromycin	1	39	4/26/2017	92	HEIGHT	13	14.5	1	0
21	5090	36	Azithromycin	1	52	5/26/2018	99.6	HEIGHT	14.8	14.5	1	0
21	5090	48	Azithromycin	1	63	5/20/2019	106.6	HEIGHT	16.3	14.5	0	0
21	5091	24	Azithromycin	1	31	4/26/2017	92.3	HEIGHT	13.45	15.5	1	0
21	5091	36	Azithromycin	1	44	5/26/2018	100	HEIGHT	15.5	15	1	0

21	5091	48	Azithromycin	1	55	5/20/2019	106.3	HEIGHT	17.63636364	15.5	1	0
21	5093	0	Azithromycin	0	55	4/8/2015	105.7	HEIGHT	16.1	15.5	1	0
21	5093	12	Azithromycin	0	54	6/15/2016	111.8	HEIGHT	16.95	15	1	0
21	5093	24	Azithromycin	0	67	4/26/2017	117.3	HEIGHT	19.25	15	0	0
21	5093	36	Azithromycin	0	80	5/26/2018	124.1	HEIGHT	21.63636364	15.5	0	0
21	5093	48	Azithromycin	0	91	5/20/2019	128.7	HEIGHT	23.59090909	16	0	0
21	5093	60	Azithromycin	0	100	2/16/2020	132.8	HEIGHT	25.25	16.5	0	0
21	5096	24	Azithromycin	1	4	4/26/2017	61.8	LENGTH	6.35	14	1	0
21	5100	24	Azithromycin	1	51	4/26/2017	98	HEIGHT	15.7	16	1	0
21	5100	48	Azithromycin	1	75	5/20/2019	111.1	HEIGHT	17.54545455	15	0	0
21	5103	24	Azithromycin	0	10	4/26/2017	72.3	LENGTH	8.3	13.5	1	0
21	5103	36	Azithromycin	0	23	5/26/2018	83.5	LENGTH	10.45454545	14.5	1	0
21	5103	48	Azithromycin	0	34	5/20/2019	89.4	HEIGHT	12.90909091	15	1	0
21	5103	60	Azithromycin	0	43	2/16/2020	95.9	HEIGHT	14.4	15	1	0
21	5105	12	Azithromycin	0	4	6/15/2016	64.6	LENGTH	6.15	12.5	0	0
21	5105	24	Azithromycin	0	8	4/26/2017	75.7	HEIGHT	8.05	13	1	0
21	5105	36	Azithromycin	1	29	5/26/2018	84.6	HEIGHT	10.13636364	12.5	1	0
21	5105	48	Azithromycin	0	27	5/20/2019	90.8	HEIGHT	11.18181818	13	1	0
21	5105	60	Azithromycin	0	37	2/16/2020	97.6	HEIGHT	12.55	12.5	0	0
21	5109	12	Azithromycin	0	50	6/15/2016	113.9	HEIGHT	16.75	14	1	0
21	5109	48	Azithromycin	0	87	5/20/2019	130.6	HEIGHT	22.31818182	14	0	0
21	5112	12	Azithromycin	0	12	6/15/2016	74.8	HEIGHT	9.1	15	1	0
21	5112	24	Azithromycin	0	24	4/26/2017	84.5	HEIGHT	10.9	15	1	0
21	5112	36	Azithromycin	0	42	5/26/2018	94.3	HEIGHT	12.81818182	15	1	0
21	5112	48	Azithromycin	0	53	5/20/2019	101.5	HEIGHT	15.18181818	15	1	0
21	5112	60	Azithromycin	0	62	2/16/2020	106.3	HEIGHT	17.25	15.1	0	0
21	5113	0	Azithromycin	0	55	4/8/2015	96.6	HEIGHT	14.25	15	1	0
21	5113	12	Azithromycin	0	66	6/15/2016	102.6	HEIGHT	15.2	15	0	0
21	5113	24	Azithromycin	0	79	4/26/2017	107.1	HEIGHT	17	15.5	0	0
21	5113	36	Azithromycin	0	92	5/26/2018	113.3	HEIGHT	18.09090909	15	0	0
21	5113	48	Azithromycin	0	103	5/20/2019	117.5	HEIGHT	19.5	15.5	0	0
21	5113	60	Azithromycin	0	112	2/16/2020	120.7	HEIGHT	22	16	0	0
21	5114	0	Azithromycin	0	36	4/8/2015	89.1	HEIGHT	12.2	14	1	0
21	5114	12	Azithromycin	0	42	6/15/2016	96.5	HEIGHT	12.75	14.5	1	0
21	5114	24	Azithromycin	0	55	4/26/2017	103.1	HEIGHT	13.6	13.5	1	0
21	5114	36	Azithromycin	0	68	5/26/2018	109.2	HEIGHT	15.35	13.5	0	0
21	5114	48	Azithromycin	0	79	5/20/2019	114.8	HEIGHT	16.81818182	13.5	0	0
21	5114	60	Azithromycin	0	88	2/16/2020	120.3	HEIGHT	18.9	13.6	0	0
21	5118	24	Azithromycin	0	36	4/26/2017	87.8	HEIGHT	12.35	14.5	1	0
21	5119	12	Azithromycin	1	42	6/15/2016	90.3	HEIGHT	12.15	15.5	1	0

21	5119	24	Azithromycin	1	55	4/26/2017	98.1	HEIGHT	13.6	15		1	0
21	5119	48	Azithromycin	1	74	5/20/2019	109.3	HEIGHT	14.5	15		0	0
21	5121	12	Azithromycin	0	9	6/15/2016	70.5	HEIGHT	8.5	15.5		1	0
21	5121	36	Azithromycin	0	34	5/26/2018	88.2	HEIGHT	12.90909091	16		1	0
21	5121	48	Azithromycin	0	45	5/20/2019	94.3	HEIGHT	14.5	16.5		1	0
21	5122	0	Azithromycin	1	48	4/8/2015	99.2	HEIGHT	15.05	15.5		1	0
21	5122	12	Azithromycin	1	54	6/15/2016	104.9	HEIGHT	16.05	16.5		1	0
21	5122	24	Azithromycin	1	67	4/26/2017	111.9	HEIGHT	17.65	16.5		0	0
21	5122	36	Azithromycin	1	80	5/26/2018	116.2	HEIGHT	19.45454545	16		0	0
21	5122	48	Azithromycin	1	91	5/27/2019	120.4	HEIGHT	21.63636364	16		0	0
21	5122	60	Azithromycin	1	100	2/16/2020	123.8	HEIGHT	22.2	16.2		0	0
21	5123	0	Azithromycin	0	48	4/8/2015	102.4	HEIGHT	14.8	14.5		1	0
21	5123	12	Azithromycin	0	62	6/15/2016	107.4	HEIGHT	16.5	15.5		0	0
21	5123	24	Azithromycin	0	75	4/26/2017	112.8	HEIGHT	18.3	15		0	0
21	5123	36	Azithromycin	0	88	5/26/2018	118.4	HEIGHT	19.77272727	15.5		0	0
21	5123	48	Azithromycin	0	99	5/20/2019	122.8	HEIGHT	21.77272727	15.5		0	0
21	5123	60	Azithromycin	0	108	2/16/2020	126.4	HEIGHT	23.95	16		0	0
21	5124	12	Azithromycin	1	7	6/15/2016	69.8	HEIGHT	7.8	14.5		1	0
21	5124	36	Azithromycin	1	33	5/26/2018	86.4	HEIGHT	11.36363636	14		1	0
21	5125	0	Azithromycin	0	31	4/8/2015	81.4	HEIGHT	10.7	14.5	48	1	0
21	5125	12	Azithromycin	0	39	6/15/2016	90.5	HEIGHT	13.05	16	48	0	0
21	5125	24	Azithromycin	0	52	4/26/2017	97.2	HEIGHT	13.65	14.5	48	1	0
21	5126	24	Azithromycin	1	8	4/26/2017	63.6	LENGTH	6.45	13.5		1	0
21	5126	36	Azithromycin	1	21	5/26/2018	73.8	LENGTH	8	13.5		1	0
21	5126	48	Azithromycin	1	32	5/20/2019	81.5	HEIGHT	10.27272727	14		1	0
21	5126	60	Azithromycin	1	41	2/16/2020	88.7	HEIGHT	12	14.8		1	0
21	5128	0	Azithromycin	1	16	4/8/2015	67.6	LENGTH	6.6	12		1	0
21	5128	12	Azithromycin	1	12	6/15/2016	79.2	HEIGHT	9.65	14		1	0
21	5128	24	Azithromycin	0	24	4/26/2017	87.6	HEIGHT	11.9	14.5		1	0
21	5128	48	Azithromycin	0	48	5/20/2019	102.2	HEIGHT	15.13636364	15		1	0
21	5129	0	Azithromycin	0	31	4/8/2015	73.5	LENGTH	9.25	15		1	0
21	5129	12	Azithromycin	0	42	6/15/2016	83.6	HEIGHT	12.65	16.5		1	0
21	5129	24	Azithromycin	0	55	4/26/2017	92.1	HEIGHT	14.65	16.5		1	0
21	5129	36	Azithromycin	0	68	5/26/2018	102.2	HEIGHT	16.36363636	16		0	0
21	5129	48	Azithromycin	0	79	5/20/2019	108.6	HEIGHT	17.22727273	15		0	0
21	5129	60	Azithromycin	0	88	2/16/2020	113.5	HEIGHT	18.7	15.4		0	0
21	5130	24	Azithromycin	1	4	4/26/2017	50.2	LENGTH	3.6	11.5		1	0
21	5132	0	Azithromycin	0	42	4/8/2015	98	HEIGHT	13.05	14.5		1	0
21	5132	12	Azithromycin	0	66	6/15/2016	101.4	HEIGHT	14	15		0	0
21	5132	24	Azithromycin	0	79	4/26/2017	106.6	HEIGHT	15.3	14		0	0

21	5132	36	Azithromycin	0	92	5/26/2018	110.6	HEIGHT	16.75	15	0	0
21	5132	48	Azithromycin	0	103	5/20/2019	114.9	HEIGHT	17.81818182	15	0	0
21	5132	60	Azithromycin	0	112	2/16/2020	118.2	HEIGHT	19.35	15.5	0	0
21	5133	24	Azithromycin	0	24	4/26/2017	87.6	HEIGHT	12.1	13.5	1	0
21	5133	36	Azithromycin	0	37	5/26/2018	95.1	HEIGHT	13.68181818	14	1	0
21	5133	48	Azithromycin	0	47	5/20/2019	101.6	HEIGHT	15.22727273	14	1	0
21	5133	60	Azithromycin	0	57	2/16/2020	105.6	HEIGHT	17.15	15.5	1	0
21	5134	0	Azithromycin	0	12	4/8/2015	70.4	LENGTH	7.9	13.5	1	0
21	5134	12	Azithromycin	0	30	6/15/2016	79.4	HEIGHT	10.8	16	1	0
21	5134	24	Azithromycin	0	43	4/26/2017	87.3	HEIGHT	12.55	16	1	0
21	5134	36	Azithromycin	0	50	5/26/2018	95.7	HEIGHT	14.63636364	16	1	0
21	5134	48	Azithromycin	0	61	5/20/2019	102.1	HEIGHT	16	15.5	0	0
21	5134	60	Azithromycin	0	71	2/16/2020	107.9	HEIGHT	18	17	0	0
21	5137	0	Azithromycin	0	24	4/8/2015	89.7	HEIGHT	11.95	15.5	1	0
21	5137	12	Azithromycin	0	38	6/15/2016	97.6	HEIGHT	13.75	14.5	1	0
21	5137	24	Azithromycin	0	51	4/26/2017	103.7	HEIGHT	15.85	15	1	0
21	5137	36	Azithromycin	0	64	5/26/2018	110.9	HEIGHT	16.90909091	15	0	0
21	5137	48	Azithromycin	0	75	5/20/2019	116.2	HEIGHT	18.40909091	14.5	0	0
21	5137	60	Azithromycin	0	84	2/16/2020	120.8	HEIGHT	21.1	15.4	0	0
21	5141	0	Azithromycin	1	10	4/8/2015	59.1	LENGTH	4.95	11.5	1	0
21	5142	0	Azithromycin	1	43	4/8/2015	87.7	HEIGHT	11.4	14	1	0
21	5142	24	Azithromycin	1	64	4/26/2017	102.1	HEIGHT	14.4	14	0	0
21	5142	36	Azithromycin	1	77	5/26/2018	109.4	HEIGHT	16.31818182	15	0	0
21	5142	48	Azithromycin	1	83	5/20/2019	113.7	HEIGHT	17.27272727	14	0	0
21	5143	24	Azithromycin	1	13	4/26/2017	72.5	LENGTH	8.05	14	1	0
21	5143	36	Azithromycin	1	26	5/26/2018	86.4	HEIGHT	10.09090909	13	1	0
21	5143	48	Azithromycin	1	37	5/20/2019	93	HEIGHT	12.13636364	14	1	0
21	5143	60	Azithromycin	1	46	2/16/2020	99.6	HEIGHT	13.5	14.3	1	0
21	5144	0	Azithromycin	1	55	4/8/2015	106.5	HEIGHT	15.45	14.5	1	0
21	5144	12	Azithromycin	1	62	6/15/2016	111.8	HEIGHT	16.55	15	0	0
21	5144	24	Azithromycin	1	75	4/26/2017	117.3	HEIGHT	17.75	15	0	0
21	5144	48	Azithromycin	1	99	5/20/2019	127.2	HEIGHT	22.40909091	16	0	0
21	5144	60	Azithromycin	1	108	2/16/2020	131.4	HEIGHT	24.15	16.5	0	0
21	5145	12	Azithromycin	0	30	6/15/2016	86.9	HEIGHT	11.3	15	1	0
21	5145	24	Azithromycin	0	43	4/26/2017	93.2	HEIGHT	13.3	15	1	0
21	5145	48	Azithromycin	0	71	5/20/2019	105.3	HEIGHT	15.86363636	14.5	0	0
21	5146	12	Azithromycin	0	50	6/15/2016	98.2	HEIGHT	13	14	1	0
21	5146	48	Azithromycin	0	91	5/20/2019	113.6	HEIGHT	17.54545455	14	0	0
21	5147	24	Azithromycin	0	11	4/26/2017	71.3	LENGTH	8.4	14	1	0
21	5147	36	Azithromycin	0	24	5/26/2018	83.2	HEIGHT	11.77272727	12.5	1	0

21	5147	48	Azithromycin	0	35	5/20/2019	91.1	HEIGHT	14.09090909	16		1	0
21	5147	60	Azithromycin	0	44	2/16/2020	97.9	HEIGHT	15.9	16		1	0
21	5148	0	Azithromycin	1	55	4/8/2015	93.4	HEIGHT	14.5	16.5		1	0
21	5148	12	Azithromycin	1	54	6/15/2016	100.9	HEIGHT	16.2	16		1	0
21	5148	24	Azithromycin	1	67	4/26/2017	106.1	HEIGHT	17.7	15.5		0	0
21	5148	36	Azithromycin	1	80	5/26/2018	112.1	HEIGHT	20.09090909	16.5		0	0
21	5150	24	Azithromycin	1	8	4/26/2017	63.6	LENGTH	5.85	11.5		1	0
21	5150	36	Azithromycin	1	21	5/26/2018	74.9	LENGTH	7.545454545	12		1	0
21	5150	60	Azithromycin	1	41	2/16/2020	89.5	HEIGHT	10.5	12.5		1	0
21	5154	12	Azithromycin	0	32	6/15/2016	95	HEIGHT	13.65	15.5		1	0
21	5154	48	Azithromycin	0	87	5/20/2019	111	HEIGHT	18.40909091	15.5		0	0
21	5155	0	Azithromycin	1	43	4/8/2015	103.5	HEIGHT	15.65	15		1	0
21	5155	24	Azithromycin	1	64	4/26/2017	114.4	HEIGHT	17.75	14		0	0
21	5158	0	Azithromycin	1	36	4/8/2015	86	HEIGHT	12.35	15		1	0
21	5158	12	Azithromycin	1	42	6/15/2016	93.1	HEIGHT	13.3	15.5		1	0
21	5158	24	Azithromycin	1	55	4/26/2017	99.9	HEIGHT	15.25	15		0	0
21	5158	60	Azithromycin	1	88	2/16/2020	117	HEIGHT	19.95	15.4		0	0
21	5161	24	Azithromycin	0	45	4/26/2017	85.8	HEIGHT	11.1	13.5		1	0
21	5161	36	Azithromycin	0	58	5/26/2018	92.9	HEIGHT	12.36363636	14		1	0
21	5161	48	Azithromycin	0	69	5/20/2019	97.6	HEIGHT	12.95454545	13		0	0
21	5164	24	Azithromycin	0	51	4/26/2017	89.5	HEIGHT	11.1	13.5		1	0
21	5164	48	Azithromycin	0	56	5/20/2019	102.6	HEIGHT	15.04545455	14		1	0
21	5165	0	Azithromycin	1	48	4/8/2015	95.9	HEIGHT	13.3	14.5		1	0
21	5165	12	Azithromycin	1	54	6/15/2016	102.6	HEIGHT	14.95	15		1	0
21	5165	24	Azithromycin	1	67	4/26/2017	108.5	HEIGHT	16.95	15		0	0
21	5165	36	Azithromycin	1	80	5/26/2018	114.5	HEIGHT	18.45454545	15		0	0
21	5165	48	Azithromycin	1	91	5/20/2019	108.9	HEIGHT	20.22727273	15.5		0	0
21	5165	60	Azithromycin	1	100	2/16/2020	122.5	HEIGHT	21.65	16		0	0
21	5171	12	Azithromycin	0	12	6/15/2016	78.4	LENGTH	8.75	15	24	1	0
21	5179	12	Azithromycin	1	18	6/15/2016	79.4	HEIGHT	9.25	15		1	0
21	5181	12	Azithromycin	0	42	6/15/2016	98.6	HEIGHT	13.95	14		1	0
21	5181	24	Azithromycin	0	55	4/26/2017	106.6	HEIGHT	15.4	14		1	0
21	5181	48	Azithromycin	0	79	5/20/2019	119.5	HEIGHT	19.77272727	15		0	0
21	5182	0	Azithromycin	0	43	4/8/2015	92.8	HEIGHT	13.35	15		1	0
21	5182	12	Azithromycin	0	54	6/15/2016	99.4	HEIGHT	15.15	16		1	0
21	5182	24	Azithromycin	0	67	4/26/2017	105.7	HEIGHT	16.4	15		0	0
21	5182	48	Azithromycin	0	91	5/20/2019	116.9	HEIGHT	20.72727273	16		0	0
21	5183	0	Azithromycin	0	55	4/8/2015	98.5	HEIGHT	14.7	15		1	0
21	5183	12	Azithromycin	0	72	6/15/2016	104.4	HEIGHT	17.25	16.5		0	0
21	5183	24	Azithromycin	0	84	4/26/2017	110.6	HEIGHT	18.8	17		0	0

21	5183	36	Azithromycin	0	98	5/26/2018	116	HEIGHT	20.18181818	17		0	0
21	5183	48	Azithromycin	0	108	5/20/2019	119	HEIGHT	22.81818182	17		0	0
21	5183	60	Azithromycin	0	118	2/16/2020	122.2	HEIGHT	24.7	17.5		0	0
21	5184	0	Azithromycin	0	10	4/8/2015	74.7	LENGTH	8.1	13.5		1	0
21	5184	12	Azithromycin	0	20	6/15/2016	84.4	HEIGHT	11.45	15.5		1	0
21	5184	24	Azithromycin	0	33	4/26/2017	93.3	HEIGHT	14.35	16		1	0
21	5184	36	Azithromycin	0	46	5/26/2018	101.2	HEIGHT	15.63636364	15.5		1	0
21	5184	48	Azithromycin	0	57	5/20/2019	108.1	HEIGHT	16.95454545	15.2		1	0
21	5184	60	Azithromycin	0	66	2/16/2020	112.8	HEIGHT	18.75	15.5		0	0
21	5185	12	Azithromycin	0	14	6/15/2016	75.4	LENGTH	8	13		1	0
21	5185	24	Azithromycin	0	28	4/26/2017	81.7	HEIGHT	10.6	14		1	0
21	5185	36	Azithromycin	0	41	5/26/2018	90.1	HEIGHT	12.75	15		1	0
21	5185	48	Azithromycin	0	51	5/20/2019	96.1	HEIGHT	13.81818182	15		1	0
21	5186	12	Azithromycin	1	14	6/15/2016	82.4	HEIGHT	11.1	15.5		1	0
21	5186	36	Azithromycin	1	44	5/26/2018	100.4	HEIGHT	15.7	15		1	0
21	5186	48	Azithromycin	1	54	5/20/2019	106.4	HEIGHT	16.40909091	15.5		1	0
21	5189	12	Azithromycin	0	50	6/15/2016	109.6	HEIGHT	16.35	15		1	0
21	5189	48	Azithromycin	0	79	5/20/2019	124.8	HEIGHT	21.3	15		0	0
21	5191	12	Azithromycin	1	32	6/15/2016	95	HEIGHT	13.25	15		1	0
21	5191	36	Azithromycin	1	58	5/26/2018	110.6	HEIGHT	17.27272727	15.5		1	0
21	5191	48	Azithromycin	1	69	5/20/2019	118	HEIGHT	20	15.5		0	0
21	5192	12	Azithromycin	0	54	6/15/2016	108.5	HEIGHT	18.35	16.5		1	0
21	5192	48	Azithromycin	0	91	5/20/2019	125.1	HEIGHT	23.81818182	16		0	0
21	5195	0	Azithromycin	1	55	4/8/2015	93.6	HEIGHT	12.5	14.5		1	0
21	5195	12	Azithromycin	1	66	6/15/2016	98.9	HEIGHT	13.55	14.5		0	0
21	5195	24	Azithromycin	1	79	4/26/2017	103.6	HEIGHT	14.8	14.5		0	0
21	5195	36	Azithromycin	1	92	5/26/2018	109.6	HEIGHT	16.95454545	15		0	0
21	5195	48	Azithromycin	1	103	5/20/2019	114.1	HEIGHT	18.36363636	15.5		0	0
21	5195	60	Azithromycin	1	112	2/16/2020	117	HEIGHT	19.85	16		0	0
21	5198	0	Azithromycin	0	55	4/8/2015	101.6	HEIGHT	14.1	14		1	0
21	5198	12	Azithromycin	0	42	6/15/2016	107.6	HEIGHT	15.85	14.5		0	0
21	5198	24	Azithromycin	0	55	4/26/2017	114	HEIGHT	18.05	14.5		1	0
21	5198	36	Azithromycin	0	68	5/26/2018	120.2	HEIGHT	19.36363636	15		0	0
21	5198	48	Azithromycin	0	79	5/20/2019	124.1	HEIGHT	21.81818182	15.5		0	0
21	5198	60	Azithromycin	0	88	2/16/2020	127.6	HEIGHT	23.55	15.5		0	0
21	8028	48	Azithromycin	0	10	5/20/2019	74.5	LENGTH	9.5	15	60	1	0
21	8041	36	Azithromycin	0	1	5/26/2018	57.5	LENGTH	6.181818182	13.5		1	0
21	8041	48	Azithromycin	0	11	5/20/2019	77.5	LENGTH	10.40909091	14.5		1	0
21	8041	60	Azithromycin	0	21	2/16/2020	85.8	HEIGHT	12.75	14.7		1	0
21	8047	48	Azithromycin	0	6	5/20/2019	63.5	LENGTH	6.772727273	13		1	0

21	8098	36	Azithromycin	1	15	5/26/2018	74.6	LENGTH	7.5	12	1	0
21	8098	48	Azithromycin	1	26	5/20/2019	84.2	HEIGHT	10.25	13	1	0
21	8101	36	Azithromycin	1	7	5/26/2018	62.2	LENGTH	6	12	1	0
21	8101	48	Azithromycin	1	18	5/20/2019	70.9	LENGTH	7.409090909	12.5	1	0
21	8101	60	Azithromycin	1	27	2/16/2020	77.5	LENGTH	9	13	1	0
21	8116	36	Azithromycin	1	13	5/26/2018	66.9	LENGTH	6.681818182	12.5	1	0
21	8116	48	Azithromycin	1	24	5/20/2019	75.2	LENGTH	8.136363636	12.5	1	0
21	8122	60	Azithromycin	1	19	2/16/2020	83.5	LENGTH	11.1	15.5	1	0
21	8211	48	Azithromycin	1	35	5/20/2019	82.1	LENGTH	10.13636364	14	1	0
21	8211	60	Azithromycin	1	45	2/16/2020	88.7	HEIGHT	11.55	14.8	1	0
21	8218	36	Azithromycin	1	23	5/26/2018	76.7	LENGTH	8.772727273	14	1	0
21	8218	48	Azithromycin	1	34	5/27/2019	83.2	HEIGHT	10.95454545	14.5	1	0
21	8218	60	Azithromycin	1	43	2/16/2020	89.2	HEIGHT	14.25	15.5	1	0
21	8222	60	Azithromycin	1	10	2/16/2020	64.6	LENGTH	7.9	14	1	0
21	8259	60	Azithromycin	0	10	2/16/2020	66.9	LENGTH	7.1	13.5	1	0
21	8266	60	Azithromycin	0	41	2/16/2020	88.6	HEIGHT	11.25	13	1	0
21	8268	36	Azithromycin	1	34	5/26/2018	92.6	HEIGHT	12.27272727	14	1	0
21	8295	60	Azithromycin	0	41	2/16/2020	90	HEIGHT	12.3	15	1	0
21	8324	36	Azithromycin	1	24	5/26/2018	78.2	LENGTH	8.863636364	12.5	1	0
21	8445	60	Azithromycin	0	5	2/16/2020	65.4	LENGTH	7.15	13	1	0
21	8481	36	Azithromycin	1	10	5/26/2018	67.7	HEIGHT	7.454545455	13	1	0
21	8481	60	Azithromycin	1	30	2/16/2020	83.7	HEIGHT	10.75	14.2	1	0
21	8500	60	Azithromycin	0	10	2/16/2020	70.6	LENGTH	8.2	13.1	1	0
21	8506	36	Azithromycin	1	12	5/26/2018	73.7	LENGTH	8.363636364	13.5	1	0
21	8506	48	Azithromycin	1	22	5/20/2019	82	HEIGHT	10.18181818	14	1	0
21	8514	60	Azithromycin	1	36	2/16/2020	89.7	HEIGHT	12.05	13.9	1	0
21	8517	60	Azithromycin	0	2	2/16/2020	59.3	LENGTH	6.35	12.5	1	0
21	8518	60	Azithromycin	1	8	2/20/2020	64.2	LENGTH	6.454545455	11.2	1	0
21	8538	60	Azithromycin	1	12	2/16/2020	71.8	HEIGHT	8	13.2	1	0
21	8624	36	Azithromycin	1	13	5/26/2018	70.6	LENGTH	6.636363636	12	1	0
21	8624	48	Azithromycin	1	24	5/20/2019	77	LENGTH	8.454545455	12.2	1	0
21	8624	60	Azithromycin	1	33	2/16/2020	86.6	HEIGHT	10.4	13.2	1	0
21	8661	36	Azithromycin	1	14	5/26/2018	73.9	LENGTH	8.318181818	13	1	0
21	8661	48	Azithromycin	1	25	5/20/2019	81.1	HEIGHT	11.40909091	14	1	0
21	8661	60	Azithromycin	1	34	2/16/2020	89	HEIGHT	13.55	14.5	1	0
21	8713	48	Azithromycin	0	10	5/20/2019	76.2	HEIGHT	9	14	1	0
21	8754	60	Azithromycin	0	9	2/16/2020	70.7	HEIGHT	7.7	12.5	1	0
21	8867	36	Azithromycin	1	21	5/26/2018	76.5	HEIGHT	7.636363636	12	1	0
21	8867	60	Azithromycin	1	41	2/16/2020	90.9	HEIGHT	11.5	13	1	0
21	9006	36	Azithromycin	1	10	5/26/2018	71.8	LENGTH	7.727272727	12.5	1	0

21	9006	48	Azithromycin	1	15	5/20/2019	98.8	HEIGHT	10.09090909	14.5	1	0
21	9033	60	Azithromycin	0	42	2/20/2020	93.1	HEIGHT	11.5	12.5	1	0
21	9036	36	Azithromycin	1	56	5/26/2018	96.8	HEIGHT	13.8	15	1	0
21	9036	48	Azithromycin	1	56	5/20/2019	102.4	HEIGHT	14.77272727	15	1	0
21	9094	60	Azithromycin	1	9	2/16/2020	70.9	LENGTH	8.85	13.6	1	0
21	9167	60	Azithromycin	1	3	2/16/2020	59.2	LENGTH	6.227272727	13.5	1	0
21	9199	48	Azithromycin	0	12	5/20/2019	75.8	LENGTH	9.090909091	14	1	0
21	9199	60	Azithromycin	0	22	2/16/2020	81.5	LENGTH	10.45	14.5	1	0
21	9233	60	Azithromycin	1	37	2/16/2020	88	HEIGHT	11.6	14.4	1	0
21	9389	48	Azithromycin	1	12	5/20/2019	64.5	LENGTH	6.590909091	12	1	0
21	9389	60	Azithromycin	1	21	2/16/2020	74.9	HEIGHT	9.9	14	1	0
21	9407	60	Azithromycin	0	6	2/16/2020	66.9	LENGTH	7.55	13.5	1	0
21	9462	60	Azithromycin	0	21	2/16/2020	79.6	HEIGHT	9.8	13.2	1	0
21	9504	48	Azithromycin	0	12	5/20/2019	73.7	LENGTH	8.818181818	14	1	0
21	9547	36	Azithromycin	0	59	5/26/2018	105.4	HEIGHT	15.31818182	14.5	1	0
21	9554	36	Azithromycin	1	18	5/26/2018	76.3	HEIGHT	9.1	13.5	1	0
21	9554	48	Azithromycin	1	28	5/20/2019	83.5	HEIGHT	11.81818182	14.5	1	0
22	5209	0	Azithromycin	0	7	4/10/2015	72.9	LENGTH	8.45	13.5	0	1
22	5209	12	Azithromycin	0	18	7/26/2016	82.3	HEIGHT		17.9	0	1
22	5209	24	Azithromycin	0	31	5/5/2017	87.6	HEIGHT	11.7	14	0	1
22	5209	36	Azithromycin	0	37	6/2/2018	93.5	HEIGHT	12.3	14	0	1
22	5209	48	Azithromycin	0	48	5/27/2019	101.7	HEIGHT	14.90909091	14.5	0	1
22	5209	60	Azithromycin	0	57	2/18/2020	108.1	HEIGHT	17.15	14.8	0	1
22	5210	12	Azithromycin	0	10	7/26/2016	66.5	LENGTH		14.7	0	1
22	5210	36	Azithromycin	0	29	6/2/2018	82	HEIGHT	10.3	13.5	0	1
22	5211	12	Azithromycin	0	10	7/26/2016	76.5	LENGTH		19	0	1
22	5211	24	Azithromycin	0	22	5/5/2017	83.7	HEIGHT	11.65	15.5	0	1
22	5211	36	Azithromycin	0	35	6/2/2018	92.9	HEIGHT	14.45	16	0	1
22	5212	0	Azithromycin	0	24	4/10/2015	87.7	HEIGHT	12.7	15.5	0	1
22	5212	12	Azithromycin	0	42	7/26/2016	97.5	HEIGHT		18.7	0	1
22	5212	24	Azithromycin	0	55	5/5/2017	102.3	HEIGHT	16	15	0	1
22	5212	36	Azithromycin	0	68	6/2/2018	110.3	HEIGHT	17.3	15.5	0	1
22	5212	48	Azithromycin	0	79	5/26/2019	114.9	HEIGHT	18.45454545	15	0	1
22	5212	60	Azithromycin	0	88	2/18/2020	118.8	HEIGHT	21.15	15.4	0	1
22	5214	12	Azithromycin	0	39	7/28/2016	91.5	HEIGHT		13.2	1	1
22	5215	12	Azithromycin	1	34	7/26/2016	86.6	HEIGHT		18.5	1	1
22	5216	0	Azithromycin	1	12	4/10/2015	78.6	LENGTH	8.75	13	0	1
22	5216	12	Azithromycin	1	27	7/26/2016	89	HEIGHT		17.5	0	1
22	5216	60	Azithromycin	1	90	2/18/2020	117.4	HEIGHT	19.3	15.4	0	1
22	5218	12	Azithromycin	1	6	7/26/2016	65.4	LENGTH		15	0	1

22	5218	36	Azithromycin	1	29	6/2/2018	74.5	LENGTH	7.95	13		0	1
22	5219	12	Azithromycin	1	11	7/26/2016	81.4	HEIGHT		17.4		0	1
22	5219	36	Azithromycin	1	34	6/2/2018	85.5	HEIGHT	10.15	12.5		0	1
22	5219	60	Azithromycin	1	54	2/18/2020	98.9	HEIGHT	13.95	14		0	1
22	5221	12	Azithromycin	1	21	7/26/2016	85.1	HEIGHT		19.1	18	1	1
22	5222	24	Azithromycin	0	54	5/5/2017	106.6	HEIGHT	15.86363636	15.5		1	1
22	5223	12	Azithromycin	0	3	7/26/2016	72.3	LENGTH		17.2		0	1
22	5223	24	Azithromycin	0	16	5/5/2017	76	LENGTH	7.454545455	11.5		0	1
22	5223	36	Azithromycin	0	29	6/2/2018	84	LENGTH	10.05	14.5		0	1
22	5223	48	Azithromycin	0	39	5/26/2019	90.7	HEIGHT	13.45454545	16		1	1
22	5223	60	Azithromycin	0	49	2/18/2020	97.1	HEIGHT	14.05	15.5		1	1
22	5224	12	Azithromycin	0	12	7/26/2016	80.1	HEIGHT		18.8		1	1
22	5224	24	Azithromycin	0	25	5/5/2017	84.5	HEIGHT	12.5	16		0	1
22	5224	36	Azithromycin	0	38	6/2/2018	93.2	HEIGHT	14.6	17		0	1
22	5224	48	Azithromycin	0	49	5/27/2019	100.6	HEIGHT	16.45454545	15.5		0	1
22	5228	0	Azithromycin	1	5	4/10/2015	70.8	LENGTH	8.9	15		1	1
22	5228	12	Azithromycin	1	19	7/26/2016	84.6	HEIGHT		19.5		0	1
22	5228	36	Azithromycin	1	41	6/2/2018	97.3	HEIGHT	15.45	16		1	1
22	5228	48	Azithromycin	1	52	5/26/2019	103.4	HEIGHT	17.31818182	16.5		0	1
22	5229	0	Azithromycin	1	36	4/10/2015	87.3	HEIGHT	11.5	14.5		0	1
22	5231	0	Azithromycin	0	36	4/10/2015	101.3	HEIGHT	15.75	15.5		0	1
22	5232	0	Azithromycin	1	48	4/10/2015	93.1	HEIGHT	12.45	14.5		1	1
22	5235	12	Azithromycin	1	18	7/28/2016	70.2	HEIGHT		11		1	1
22	5235	48	Azithromycin	1	55	5/26/2019	92.3	HEIGHT	12.27272727	13		1	1
22	5236	12	Azithromycin	0	18	7/26/2016	73.1	LENGTH		16.2		1	1
22	5236	36	Azithromycin	0	44	6/2/2018	89.7	HEIGHT	12.95	16		1	1
22	5237	12	Azithromycin	0	6	7/26/2016	70.8	LENGTH		16.2		0	1
22	5237	36	Azithromycin	0	29	6/2/2018	85.9	HEIGHT	12.15	15		0	1
22	5237	60	Azithromycin	0	49	2/18/2020	102	HEIGHT	15.25	14.2		0	1
22	5240	0	Azithromycin	1	48	4/10/2015	111	HEIGHT	17.55	14		0	1
22	5241	0	Azithromycin	0	4	4/10/2015	66.7	LENGTH	5.95	11	36	0	1
22	5241	12	Azithromycin	0	18	7/26/2016	72.5	LENGTH		15	36	0	1
22	5241	24	Azithromycin	0	31	5/5/2017	77.6	LENGTH	10.09090909	14.5	36	0	1
22	5242	12	Azithromycin	1	9	7/26/2016	69.8	LENGTH		14.2		0	1
22	5242	24	Azithromycin	1	22	5/5/2017	78.5	HEIGHT	8.545454545	14		0	1
22	5246	0	Azithromycin	1	24	4/10/2015	76.2	HEIGHT	9.8	14		0	1
22	5254	12	Azithromycin	1	7	7/26/2016	70.9	LENGTH		15.6		0	1
22	5254	24	Azithromycin	1	21	5/5/2017	79.7	HEIGHT	11.55	15.5		0	1
22	5255	0	Azithromycin	0	3	4/10/2015	62.3	LENGTH	6.3	13		1	1
22	5255	12	Azithromycin	0	12	7/26/2016	75.3	HEIGHT		14.5		0	1

22	5255	24	Azithromycin	0	25	5/5/2017	82.7	HEIGHT	10.31818182	13.5		1	1
22	5255	36	Azithromycin	0	38	6/2/2018	89.9	HEIGHT	12.6	14		0	1
22	5255	48	Azithromycin	0	49	5/26/2019	94.8	HEIGHT	13.68181818	14		0	1
22	5256	24	Azithromycin	1	21	5/5/2017	71.9	HEIGHT	7.272727273	13		1	1
22	5257	24	Azithromycin	1	3	5/5/2017	61.7	LENGTH	6.5	14	60	1	1
22	5257	36	Azithromycin	1	16	6/2/2018	76.7	HEIGHT	9.4	14	60	0	1
22	5257	48	Azithromycin	1	27	5/27/2019	82.7	HEIGHT	11.59090909	15	60	0	1
22	5258	0	Azithromycin	1	30	4/10/2015	88.4	HEIGHT	11.85	15		0	1
22	5258	12	Azithromycin	1	42	7/26/2016	98.2	HEIGHT		16.7		1	1
22	5259	0	Azithromycin	1	36	4/10/2015	81.2	HEIGHT	9.4	12.5		0	1
22	5261	0	Azithromycin	0	12	6/10/2015	76.6	LENGTH	9.15	14		0	1
22	5261	12	Azithromycin	0	30	7/26/2016	84.5	HEIGHT		18		0	1
22	5261	24	Azithromycin	0	34	5/5/2017	90.2	HEIGHT	12.45	15		0	1
22	5261	36	Azithromycin	0	47	6/2/2018	98.1	HEIGHT	14.35	14.5		0	1
22	5261	48	Azithromycin	0	57	5/27/2019	103.8	HEIGHT	15.40909091	14		0	1
22	5261	60	Azithromycin	0	67	2/18/2020	108.3	HEIGHT	16	13.5		0	1
22	5262	24	Azithromycin	0	39	5/5/2017	85.9	HEIGHT	11.5	14.5		1	1
22	5263	0	Azithromycin	0	19	4/10/2015	66	LENGTH	6.85	13		0	1
22	5266	24	Azithromycin	1	1	5/5/2017	50.6	LENGTH	3.954545455	11.5		1	1
22	5266	60	Azithromycin	1	34	2/18/2020	79.5	HEIGHT	10.3	14.5		0	1
22	5269	0	Azithromycin	0	24	4/10/2015	84.9	HEIGHT	11.25	14		0	1
22	5269	12	Azithromycin	0	43	7/26/2016	94.2	HEIGHT		19.8		1	1
22	5269	24	Azithromycin	0	52	5/5/2017	99.2	HEIGHT	17.09090909	17		0	1
22	5271	12	Azithromycin	1	46	7/26/2016	93.5	HEIGHT		17.4		1	1
22	5276	0	Azithromycin	0	36	4/10/2015	106.4	HEIGHT	16	14		0	1
22	5276	60	Azithromycin	0	98	2/18/2020	127	HEIGHT	25.95	17		0	1
22	5277	12	Azithromycin	1	0	7/26/2016	62.5	LENGTH		15.2		0	1
22	5277	24	Azithromycin	1	14	5/5/2017	71.5	LENGTH	7.55	13		0	1
22	5277	36	Azithromycin	1	27	6/2/2018	80.4	HEIGHT	9.95	14.5		1	1
22	5277	60	Azithromycin	1	48	2/18/2020	89.7	HEIGHT	13.75	14.2		0	1
22	5278	0	Azithromycin	1	48	4/10/2015	94.7	HEIGHT	12.95	14.5		0	1
22	5278	12	Azithromycin	1	54	7/26/2016	102.2	HEIGHT		17.2		0	1
22	5278	24	Azithromycin	1	67	5/5/2017	107.2	HEIGHT	15.5	14.5		0	1
22	5278	36	Azithromycin	1	80	6/2/2018	112.2	HEIGHT	16.9	15		0	1
22	5278	48	Azithromycin	1	91	5/26/2019	117	HEIGHT	18.95454545	15		0	1
22	5278	60	Azithromycin	1	101	2/18/2020	120.9	HEIGHT	20.3	14.9		0	1
22	5280	0	Azithromycin	1	48	4/10/2015	89.9	HEIGHT	11.55	13.5		1	1
22	5280	12	Azithromycin	1	57	7/26/2016	98.8	HEIGHT		16.6		0	1
22	5280	24	Azithromycin	1	70	5/5/2017	104.3	HEIGHT	15.72727273	13		0	1
22	5280	36	Azithromycin	1	83	6/2/2018	110.9	HEIGHT	17.1	15		0	1

22	5280	48	Azithromycin	1	94	5/26/2019	116.2	HEIGHT	18.45454545	15		0	1
22	5280	60	Azithromycin	1	103	2/18/2020	121.7	HEIGHT	20.25	14.8		0	1
22	5284	0	Azithromycin	1	6	4/10/2015	66	LENGTH	6.15	12		0	1
22	5284	24	Azithromycin	1	31	5/5/2017	85.5	HEIGHT	11.85	14.5		0	1
22	5284	48	Azithromycin	1	55	5/27/2019	105.1	HEIGHT	16.5	15.5		0	1
22	5284	60	Azithromycin	1	55	2/18/2020	110.2	HEIGHT	17.25	14.5		1	1
22	5286	0	Azithromycin	1	48	4/10/2015	88.7	HEIGHT	12.3	14		1	1
22	5292	0	Azithromycin	0	36	4/10/2015	85.5	HEIGHT	10.65	14		1	1
22	5292	48	Azithromycin	0	88	5/26/2019	110	HEIGHT	17.40909091	14.5		0	1
22	5293	24	Azithromycin	1	43	5/5/2017	98.9	HEIGHT	15.05	15		1	1
22	5294	24	Azithromycin	1	55	5/5/2017	105.6	HEIGHT	16.13636364	14.5		1	1
22	5299	0	Azithromycin	0	36	4/10/2015	91.7	HEIGHT	12.75	14.5		1	1
22	5299	12	Azithromycin	0	54	7/26/2016	99.9	HEIGHT		17.6		0	1
22	5299	24	Azithromycin	0	58	5/5/2017	105	HEIGHT	16	15.5		0	1
22	5299	36	Azithromycin	0	71	6/2/2018	110.6	HEIGHT	18	16		0	1
22	5299	48	Azithromycin	0	81	5/26/2019	115.2	HEIGHT	19.72727273	15		0	1
22	5299	60	Azithromycin	0	91	2/18/2020	118.4	HEIGHT	20.2	15.2		0	1
22	5300	0	Azithromycin	0	48	4/10/2015	79.8	HEIGHT	8.8	12.5		0	1
22	5302	24	Azithromycin	0	17	5/5/2017	75.7	HEIGHT	8.4	12		1	1
22	5303	24	Azithromycin	1	55	5/5/2017	96.4	HEIGHT	14.59090909	15		1	1
22	5309	12	Azithromycin	1	12	7/26/2016	74.4	LENGTH		14		1	1
22	5309	24	Azithromycin	1	21	5/5/2017	77.7	HEIGHT	8.545454545	13		0	1
22	5309	36	Azithromycin	1	34	6/2/2018	83.2	LENGTH	9.65	13.5		0	1
22	5309	48	Azithromycin	1	45	5/26/2019	88.2	HEIGHT	11.18181818	14		0	1
22	5309	60	Azithromycin	1	54	2/21/2020	94.8	HEIGHT	12.54545455	13.5		0	1
22	5312	0	Azithromycin	0	48	4/10/2015	98.3	HEIGHT	14.25	14.5		0	1
22	5317	12	Azithromycin	1	39	7/26/2016	103.4	HEIGHT		18.2		1	1
22	5319	0	Azithromycin	0	48	4/10/2015	91.7	HEIGHT	14.5	15.5		0	1
22	5319	36	Azithromycin	0	75	6/2/2018	110.7	HEIGHT	18.75	15.5		0	1
22	5319	48	Azithromycin	0	86	5/26/2019	116.6	HEIGHT	21.13636364	16.5		0	1
22	5320	24	Azithromycin	1	55	5/5/2017	91.2	HEIGHT	12.22727273	14		1	1
22	5321	0	Azithromycin	0	24	4/10/2015	78.5	LENGTH	9.2	14		0	1
22	5321	12	Azithromycin	0	34	7/26/2016	86.7	HEIGHT		18.5		0	1
22	5321	24	Azithromycin	0	43	5/5/2017	91.9	HEIGHT	14.13636364	16		0	1
22	5321	36	Azithromycin	0	56	6/2/2018	98.7	HEIGHT	15.9	16		0	1
22	5321	48	Azithromycin	0	67	5/26/2019	114	HEIGHT	17.40909091	16		0	1
22	5322	0	Azithromycin	1	24	4/10/2015	74.5	LENGTH	7.7	11.5		0	1
22	5323	12	Azithromycin	1	4	7/26/2016	66.2	LENGTH		16.3	24	0	1
22	5324	0	Azithromycin	0	24	4/10/2015	76.5	LENGTH	8.55	13		1	1
22	5324	12	Azithromycin	0	54	7/26/2016	84.3	HEIGHT		19.5		0	1

22	5324	24	Azithromycin	0	67	5/5/2017	90.2	HEIGHT	15	17		0	1
22	5324	36	Azithromycin	0	80	6/2/2018	101.3	HEIGHT	16.25	16		0	1
22	5324	48	Azithromycin	0	91	5/26/2019	108.3	HEIGHT	17.86363636	15.5		0	1
22	5324	60	Azithromycin	0	100	2/18/2020	113.6	HEIGHT	19.05	15.4		0	1
22	5325	0	Azithromycin	1	48	6/10/2015	92.7	HEIGHT	12.9	15.5		1	1
22	5327	0	Azithromycin	1	43	4/10/2015	77.5	HEIGHT	8.85	13		1	1
22	5327	24	Azithromycin	1	64	5/5/2017	93	HEIGHT	12.36363636	14.5		0	1
22	5328	24	Azithromycin	0	55	5/5/2017	101.6	HEIGHT	15.77272727	14.5		1	1
22	5333	0	Azithromycin	1	24	4/10/2015	73.7	HEIGHT	7.8	13		1	1
22	5333	12	Azithromycin	1	39	7/26/2016	82.4	HEIGHT		17.5		1	1
22	5333	24	Azithromycin	1	52	5/5/2017	89	HEIGHT	11.13636364	13.5		0	1
22	5333	36	Azithromycin	1	65	6/2/2018	96.3	HEIGHT	12	13.5		0	1
22	5333	48	Azithromycin	1	76	5/26/2019	101.6	HEIGHT	12.72727273	13		0	1
22	5334	12	Azithromycin	1	2	7/26/2016	63.5	LENGTH		14	30	0	1
22	5334	24	Azithromycin	1	16	5/5/2017	68	LENGTH	5.818181818	11.5	30	0	1
22	5335	12	Azithromycin	1	1	7/26/2016	62.8	LENGTH		15		0	1
22	5335	36	Azithromycin	1	29	6/2/2018	82.6	HEIGHT	10.95	14		0	1
22	5335	60	Azithromycin	1	49	2/18/2020	96.9	HEIGHT	14.9	14.5		0	1
22	5338	0	Azithromycin	1	36	6/10/2015	81.4	HEIGHT	11.8	16		1	1
22	5341	0	Azithromycin	1	48	4/10/2015	94.2	HEIGHT	11.9	13.5		1	1
22	5343	24	Azithromycin	0	64	5/5/2017	95.3	HEIGHT	14.1	15		1	1
22	5344	12	Azithromycin	0	9	7/26/2016	80.5	LENGTH		18.2		0	1
22	5344	24	Azithromycin	0	71	5/5/2017	85	HEIGHT	12.8	16.5		0	1
22	5344	36	Azithromycin	0	48	6/2/2018	91.4	HEIGHT	14.35	16.5		0	1
22	5346	0	Azithromycin	1	48	4/10/2015	106.3	HEIGHT	16.9	15.5		0	1
22	5347	0	Azithromycin	0	36	4/10/2015	102.9	HEIGHT	17.05	17		1	1
22	5347	36	Azithromycin	0	78	6/2/2018	120.4	HEIGHT	21.15	16		0	1
22	5349	24	Azithromycin	0	11	5/5/2017	73.4	LENGTH	7.954545455	14		1	1
22	5349	36	Azithromycin	0	21	6/2/2018	82.9	LENGTH	10.3	14.5		0	1
22	5350	0	Azithromycin	0	36	4/10/2015	106.3	HEIGHT	15.65	14		0	1
22	5355	12	Azithromycin	1	3	7/26/2016	66.3	HEIGHT		15.2		1	1
22	5355	24	Azithromycin	1	16	5/5/2017	73.5	LENGTH	9.136363636	14.5		0	1
22	5355	36	Azithromycin	1	29	6/2/2018	79.5	HEIGHT	9.25	12.5		0	1
22	5355	48	Azithromycin	1	39	5/27/2019	87	HEIGHT	13.5	15		0	1
22	5355	60	Azithromycin	1	49	2/18/2020	93.3	HEIGHT	15.15	15.4		0	1
22	5357	0	Azithromycin	0	3	4/10/2015	61.2	LENGTH	5.1	11.5		0	1
22	5358	0	Azithromycin	1	24	4/10/2015	81.9	HEIGHT	9.55	13.5		0	1
22	5359	0	Azithromycin	0	42	4/10/2015	97	HEIGHT	16.25	18		0	1
22	5359	24	Azithromycin	0	70	5/5/2017	109.8	HEIGHT	20.59090909	18		0	1
22	5369	0	Azithromycin	0	48	4/10/2015	116	HEIGHT	20.1	17		0	1

22	5369	12	Azithromycin	0	94	7/28/2016	123.4	HEIGHT		17		0	1
22	5369	24	Azithromycin	0	103	5/5/2017	126.2	HEIGHT	24.27272727	17		0	1
22	5369	36	Azithromycin	0	116	6/2/2018	129.6	HEIGHT	26.35	18		0	1
22	5369	48	Azithromycin	0	127	5/26/2019	133.6	HEIGHT	28.54545455	17		0	1
22	5369	60	Azithromycin	0	136	2/18/2020	137.2	HEIGHT	31.6	18.5		0	1
22	5370	0	Azithromycin	0	36	4/10/2015	104.2	HEIGHT	14.75	14.5		0	1
22	5370	12	Azithromycin	0	49	7/26/2016	111.4	HEIGHT		17.4		0	1
22	5370	24	Azithromycin	0	62	5/5/2017	116.5	HEIGHT	18.18181818	15		1	1
22	5371	12	Azithromycin	0	2	7/26/2016	65.7	LENGTH		17.2		0	1
22	5371	24	Azithromycin	0	15	5/5/2017	73.3	HEIGHT	8.409090909	13		0	1
22	5371	36	Azithromycin	0	28	6/2/2018	83.4	HEIGHT	10.85	14		0	1
22	5371	60	Azithromycin	0	48	2/18/2020	96.2	HEIGHT	13.85	14.4		0	1
22	5372	12	Azithromycin	0	39	7/26/2016	94.2	HEIGHT		18.5		1	1
22	5375	0	Azithromycin	1	36	4/10/2015	84.5	HEIGHT	11.55	15.5		0	1
22	5375	12	Azithromycin	1	30	7/26/2016	95.8	HEIGHT		18.2		0	1
22	5375	24	Azithromycin	1	43	5/5/2017	100.6	HEIGHT	14.90909091	15.5		0	1
22	5375	36	Azithromycin	1	56	6/2/2018	108.7	HEIGHT	15.85	15		1	1
22	5375	48	Azithromycin	1	67	5/26/2019	113.6	HEIGHT	17.59090909	15		0	1
22	5375	60	Azithromycin	1	76	2/18/2020	118.5	HEIGHT	19.7	15.5		0	1
22	5376	12	Azithromycin	1	12	7/26/2016	62.5	LENGTH		15.1		0	1
22	5376	24	Azithromycin	1	25	5/5/2017	71.7	HEIGHT	7.5	13		0	1
22	5376	36	Azithromycin	1	27	6/2/2018	81.3	LENGTH	9.8	14		1	1
22	5376	48	Azithromycin	1	38	5/26/2019	88.6	HEIGHT	11.77272727	14.5		1	1
22	5376	60	Azithromycin	1	47	2/18/2020	93.6	HEIGHT	12.65	14		0	1
22	5380	12	Azithromycin	0	9	7/26/2016	72.5	LENGTH		17	30	1	1
22	5380	24	Azithromycin	0	22	5/5/2017	75.8	HEIGHT	8.1	12	30	1	1
22	5381	0	Azithromycin	1	36	4/10/2015	89.3	HEIGHT	10.9	13.5		1	1
22	5381	12	Azithromycin	1	58	7/26/2016	97.3	HEIGHT		16.7		0	1
22	5381	24	Azithromycin	1	67	5/5/2017	101	HEIGHT	14.18181818	14.5		0	1
22	5381	36	Azithromycin	1	80	6/2/2018	105.8	HEIGHT	16.2	14		0	1
22	5381	48	Azithromycin	1	91	5/26/2019	110.5	HEIGHT	17	13		0	1
22	5381	60	Azithromycin	1	100	2/18/2020	114.3	HEIGHT	18.1	14.3		0	1
22	5383	0	Azithromycin	0	48	4/10/2015	91.8	HEIGHT	14.45	16		0	1
22	5383	12	Azithromycin	0	63	7/26/2016	100.1	HEIGHT		17.9		0	1
22	5383	24	Azithromycin	0	76	5/5/2017	104.8	HEIGHT	17.9	15.5		0	1
22	5383	36	Azithromycin	0	89	6/2/2018	111.3	HEIGHT	19.7	16		0	1
22	5383	48	Azithromycin	0	100	5/26/2019	114.2	HEIGHT	21.04545455	15.5		0	1
22	5384	0	Azithromycin	0	6	4/10/2015	66.6	LENGTH	6.95	13.5	6	1	1
22	5385	0	Azithromycin	0	36	4/10/2015	92.8	HEIGHT	11.65	13.5		0	1
22	5385	12	Azithromycin	0	36	7/26/2016	101.4	HEIGHT		16.5		0	1

22	5385	24	Azithromycin	0	49	5/5/2017	105.6	HEIGHT	15.5	14	0	1
22	5385	36	Azithromycin	0	62	6/2/2018	111.7	HEIGHT	16.55	14	0	1
22	5385	60	Azithromycin	0	82	2/18/2020	120.4	HEIGHT	19.55	14	0	1
22	5386	12	Azithromycin	1	46	7/28/2016	103.2	HEIGHT		14.2	1	1
22	5388	12	Azithromycin	0	7	7/26/2016	68.4	LENGTH		18.3	0	1
22	5388	24	Azithromycin	0	16	5/5/2017	76.5	HEIGHT	9.863636364	14	0	1
22	5388	36	Azithromycin	0	29	6/2/2018	85	HEIGHT	11.35	15	1	1
22	5388	48	Azithromycin	0	39	5/26/2019	91.6	HEIGHT	13.18181818	15	1	1
22	5388	60	Azithromycin	0	49	2/18/2020	97.1	HEIGHT	14.2	15	1	1
22	5390	12	Azithromycin	1	30	7/28/2016	85.6	HEIGHT		14.6	1	1
22	5392	0	Azithromycin	1	6	4/10/2015	67.9	LENGTH	6.8	12	0	1
22	5393	24	Azithromycin	0	53	5/5/2017	100.5	HEIGHT	15.31818182	15	1	1
22	5398	0	Azithromycin	0	48	4/10/2015	83.7	HEIGHT	10.7	13.5	1	1
22	5401	0	Azithromycin	0	24	4/10/2015	74.1	LENGTH	8.95	14.5	0	1
22	5401	12	Azithromycin	0	39	7/26/2016	83.3	HEIGHT		17.5	0	1
22	5401	24	Azithromycin	0	52	5/5/2017	87.9	HEIGHT	11.7	14.5	0	1
22	5401	36	Azithromycin	0	65	6/2/2018	93.7	HEIGHT	13.7	15	0	1
22	5401	60	Azithromycin	0	85	2/18/2020	104.5	HEIGHT	16.45	15	0	1
22	5402	0	Azithromycin	0	12	4/10/2015	73.5	HEIGHT	8	13	0	1
22	5402	24	Azithromycin	0	40	5/5/2017	92.8	HEIGHT	12.90909091	14.5	1	1
22	5402	36	Azithromycin	0	53	6/2/2018	99.7	HEIGHT	14.3	14.5	0	1
22	5402	48	Azithromycin	0	64	5/26/2019	105.5	HEIGHT	16.63636364	14.5	0	1
22	5402	60	Azithromycin	0	73	2/18/2020	110.9	HEIGHT	18.85	15	0	1
22	5403	24	Azithromycin	0	10	5/5/2017	69.8	LENGTH	7.55	13	1	1
22	5403	48	Azithromycin	0	34	5/27/2019	87.9	HEIGHT	13.90909091	15.5	0	1
22	5404	24	Azithromycin	1	3	5/5/2017	55.5	LENGTH	5.045454545	13	1	1
22	5404	36	Azithromycin	1	15	6/2/2018	70.7	LENGTH	7.1	12.5	0	1
22	5404	48	Azithromycin	1	26	5/27/2019	74.6	LENGTH	8.045454545	12.5	0	1
22	5404	60	Azithromycin	1	35	2/18/2020	81.7	HEIGHT	11.05	15	0	1
22	5405	0	Azithromycin	1	36	4/10/2015	70.7	LENGTH	8.15	13.5	0	1
22	5406	0	Azithromycin	1	12	4/10/2015	77.2	HEIGHT	8.35	12.5	0	1
22	5406	24	Azithromycin	1	43	5/5/2017	90.6	HEIGHT	11.63636364	13	0	1
22	5406	36	Azithromycin	1	56	6/2/2018	98.7	HEIGHT	13.3	14	0	1
22	5406	48	Azithromycin	1	67	5/26/2019	104.1	HEIGHT	14.22727273	13	0	1
22	5406	60	Azithromycin	1	76	2/18/2020	108.8	HEIGHT	14.75	12.8	0	1
22	5408	24	Azithromycin	1	33	5/5/2017	84.1	HEIGHT	10.27272727	12.5	1	1
22	5408	48	Azithromycin	1	57	5/26/2019	102.3	HEIGHT	13.27272727	13	1	1
22	5409	0	Azithromycin	0	12	4/10/2015	82.2	HEIGHT	12.55	16.5	0	1
22	5409	60	Azithromycin	0	73	2/18/2020	117.2	HEIGHT	23.1	17.2	0	1
22	5410	12	Azithromycin	0	46	7/26/2016	90.6	HEIGHT		17	1	1

22	5412	0	Azithromycin	1	43	4/10/2015	86.1	HEIGHT	10.5	13.5		1	1
22	5412	12	Azithromycin	1	30	7/26/2016	94.6	HEIGHT		18.6		0	1
22	5412	24	Azithromycin	1	43	5/5/2017	100.9	HEIGHT	16.86363636	17		0	1
22	5412	36	Azithromycin	1	56	6/2/2018	110	HEIGHT	17.7	17		0	1
22	5412	48	Azithromycin	1	67	5/26/2019	114.7	HEIGHT	20.59090909	16.5		0	1
22	5413	0	Azithromycin	0	36	4/10/2015	85.2	HEIGHT	11.1	14		0	1
22	5413	12	Azithromycin	0	42	7/26/2016	94.8	HEIGHT		18.8		0	1
22	5413	36	Azithromycin	0	68	6/2/2018	109.3	HEIGHT	20.5	17		0	1
22	5413	48	Azithromycin	0	79	5/26/2019	114.9	HEIGHT	22.22727273	16		0	1
22	5413	60	Azithromycin	0	88	2/18/2020	120.3	HEIGHT	22.85	16		0	1
22	5414	0	Azithromycin	0	2	4/10/2015	67.6	LENGTH	6.85	12.5		0	1
22	5414	48	Azithromycin	0	50	5/27/2019	99.5	HEIGHT	15.18181818	15		0	1
22	5417	0	Azithromycin	0	18	4/10/2015	79	LENGTH	9.55	13.5		0	1
22	5417	12	Azithromycin	0	30	7/26/2016	87.2	HEIGHT		16.2		0	1
22	5417	24	Azithromycin	0	43	5/5/2017	94.1	HEIGHT	14	15		0	1
22	5417	36	Azithromycin	0	56	6/2/2018	101.7	HEIGHT	15.35	15		0	1
22	5417	48	Azithromycin	0	67	5/26/2019	107.6	HEIGHT	17.59090909	15		0	1
22	5417	60	Azithromycin	0	76	2/18/2020	111.4	HEIGHT	18.95	15.5		0	1
22	5418	0	Azithromycin	1	48	4/10/2015	111.8	HEIGHT	16.7	15		1	1
22	5419	12	Azithromycin	0	6	7/26/2016	70.8	LENGTH		14.8		0	1
22	5419	24	Azithromycin	0	16	5/5/2017	75.4	HEIGHT	8.863636364	13		0	1
22	5419	36	Azithromycin	0	29	6/2/2018	84.2	HEIGHT	12.15	15		1	1
22	5419	48	Azithromycin	0	39	5/26/2019	92	HEIGHT	14.63636364	15		1	1
22	5419	60	Azithromycin	0	49	2/18/2020	99.5	HEIGHT	17.15	16.2		0	1
22	5421	12	Azithromycin	0	7	7/26/2016	71.2	HEIGHT		17.7	30	1	1
22	5425	12	Azithromycin	1	42	7/26/2016	101.3	HEIGHT		17.9		1	1
22	5426	12	Azithromycin	1	9	7/26/2016	66.5	LENGTH		12.4		0	1
22	5426	36	Azithromycin	1	35	6/2/2018	76.8	LENGTH	9.8	14		0	1
22	5427	0	Azithromycin	1	25	4/10/2015	86	HEIGHT	11	13.5	12	1	1
22	5427	36	Azithromycin	1	59	6/2/2018	110.7	HEIGHT	16.45	14.5	12	0	1
22	5427	48	Azithromycin	1	70	5/26/2019	116.1	HEIGHT	18	15	12	0	1
22	5427	60	Azithromycin	1	79	2/18/2020	120.3	HEIGHT	19.8	14.6	12	0	1
22	5431	0	Azithromycin	1	31	4/10/2015	77.1	HEIGHT	8.65	12.5		1	1
22	5434	0	Azithromycin	1	36	4/10/2015	97.7	HEIGHT	13.5	15.5		0	1
22	5436	0	Azithromycin	0	48	4/10/2015	95.5	HEIGHT	12.05	13		0	1
22	5437	0	Azithromycin	1	43	4/10/2015	88.5	HEIGHT	12.35	14.5		0	1
22	5438	0	Azithromycin	0	30	6/10/2015	88.7	HEIGHT	14.1	16		1	1
22	5438	24	Azithromycin	0	64	5/5/2017	102.9	HEIGHT	16.72727273	16		0	1
22	5438	60	Azithromycin	0	97	2/18/2020	117	HEIGHT	22.25	16.9		0	1
22	5439	12	Azithromycin	1	7	7/26/2016	71.3	HEIGHT		15.3		0	1

22	5439	24	Azithromycin	1	16	5/5/2017	82.7	HEIGHT	9.545454545	14		1	1
22	5439	36	Azithromycin	1	29	6/2/2018	92.2	HEIGHT	12.85	15		1	1
22	5439	60	Azithromycin	1	49	2/18/2020	104.7	HEIGHT	16.1	15		0	1
22	5441	24	Azithromycin	1	21	5/5/2017	81.7	HEIGHT	10.13636364	14		1	1
22	5443	0	Azithromycin	0	48	4/10/2015	99.4	HEIGHT	14.6	14.5		1	1
22	5443	12	Azithromycin	0	70	7/26/2016	108	HEIGHT		16.9		0	1
22	5443	24	Azithromycin	0	79	5/5/2017	113	HEIGHT	17.27272727	15		0	1
22	5443	36	Azithromycin	0	92	6/2/2018	117.5	HEIGHT	18.65	14.5		0	1
22	5444	12	Azithromycin	0	39	7/26/2016	96	HEIGHT		15.6		1	1
22	5444	24	Azithromycin	0	52	5/5/2017	101.3	HEIGHT	16.63636364	15.5		1	1
22	5448	12	Azithromycin	1	42	7/26/2016	80.3	LENGTH		14.4		1	1
22	5452	12	Azithromycin	0	58	7/26/2016	105.2	HEIGHT		16.8		1	1
22	5453	12	Azithromycin	0	3	7/26/2016	61.5	LENGTH		16.8		0	1
22	5453	24	Azithromycin	0	16	5/5/2017	70	HEIGHT	7.363636364	13		0	1
22	5453	36	Azithromycin	0	29	6/2/2018	76.5	LENGTH	8.7	13		0	1
22	5453	48	Azithromycin	0	40	5/26/2019	79.9	HEIGHT	9.545454545	12.5		1	1
22	5453	60	Azithromycin	0	49	2/18/2020	84.8	HEIGHT	12.65	14.4		0	1
22	5458	0	Azithromycin	1	30	4/10/2015	82.7	HEIGHT	9.15	12.5		0	1
22	5458	12	Azithromycin	1	49	7/26/2016	95.6	HEIGHT		17.4		1	1
22	5459	12	Azithromycin	0	9	7/26/2016	71.3	LENGTH		11.5	30	0	1
22	5460	0	Azithromycin	0	24	4/10/2015	81	HEIGHT	11.3	15		0	1
22	5460	24	Azithromycin	0	52	5/5/2017	99.5	HEIGHT	17.1	17		0	1
22	5461	0	Azithromycin	1	48	4/10/2015	110.3	HEIGHT	16.05	14.5		1	1
22	5461	12	Azithromycin	1	70	7/26/2016	116.6	HEIGHT		18.4		0	1
22	5461	24	Azithromycin	1	79	5/5/2017	119.8	HEIGHT	20.31818182	14.5		0	1
22	5462	0	Azithromycin	1	12	6/10/2015	77.1	LENGTH	9.65	13.5		0	1
22	5462	12	Azithromycin	1	27	7/26/2016	84.2	HEIGHT		16.6		1	1
22	5462	24	Azithromycin	1	40	5/5/2017	91.1	HEIGHT	13.25	15		0	1
22	5462	36	Azithromycin	1	53	6/2/2018	97.7	HEIGHT	15.3	16		0	1
22	5462	48	Azithromycin	1	64	5/26/2019	103.4	HEIGHT	16.18181818	14.5		0	1
22	5462	60	Azithromycin	1	73	2/18/2020	107.1	HEIGHT	17.65	15		0	1
22	5464	12	Azithromycin	1	11	7/26/2016	65.7	LENGTH		13.8		0	1
22	5464	24	Azithromycin	1	22	5/5/2017	71.2	HEIGHT	6.954545455	14		0	1
22	5464	36	Azithromycin	1	35	6/2/2018	80	HEIGHT	9.5	14		0	1
22	5464	60	Azithromycin	1	55	2/18/2020	95.3	HEIGHT	13.45	15.5		0	1
22	5465	12	Azithromycin	0	42	7/26/2016	102.3	HEIGHT		17.3		1	1
22	5466	12	Azithromycin	0	58	7/26/2016	128.4	HEIGHT		19.2		1	1
22	5467	0	Azithromycin	0	48	4/10/2015	102.6	HEIGHT	15.35	15		1	1
22	5467	12	Azithromycin	0	66	7/26/2016	109.5	HEIGHT		18		0	1
22	5467	24	Azithromycin	0	79	5/5/2017	113.1	HEIGHT	17.72727273	15.5		0	1

22	5467	36	Azithromycin	0	92	6/2/2018	118.4	HEIGHT	20.05	16	0	1
22	5467	48	Azithromycin	0	103	5/27/2019	122.7	HEIGHT	22.27272727	17	0	1
22	5467	60	Azithromycin	0	112	2/18/2020	126.3	HEIGHT	22.8	16.5	0	1
22	5468	0	Azithromycin	0	36	4/10/2015	106.4	HEIGHT	16.65	14.5	0	1
22	5470	12	Azithromycin	0	27	7/26/2016	84.2	HEIGHT		16.6	1	1
22	5471	0	Azithromycin	0	48	4/10/2015	90.3	HEIGHT	12.35	14.5	0	1
22	5474	24	Azithromycin	1	34	5/5/2017	77.4	HEIGHT	8.045454545	11	1	1
22	5474	48	Azithromycin	1	58	5/26/2019	94.9	HEIGHT	12.86363636	13.5	1	1
22	5475	12	Azithromycin	0	30	7/26/2016	93.2	HEIGHT		16.8	1	1
22	5478	12	Azithromycin	0	43	7/26/2016	99.2	HEIGHT		16.9	1	1
22	5480	24	Azithromycin	1	35	5/5/2017	90.2	HEIGHT	13.5	15.5	1	1
22	5481	0	Azithromycin	0	24	4/10/2015	84.5	HEIGHT	12.1	15	1	1
22	5483	0	Azithromycin	0	48	4/10/2015	89.8	HEIGHT	13.2	16	0	1
22	5483	48	Azithromycin	0	91	5/27/2019	113.2	HEIGHT	20.31818182	16.5	0	1
22	5484	0	Azithromycin	1	24	4/10/2015	78.6	LENGTH	9.7	14.5	1	1
22	5484	12	Azithromycin	1	30	7/26/2016	89.3	HEIGHT		19.8	0	1
22	5484	24	Azithromycin	1	43	5/5/2017	96.2	HEIGHT	14.77272727	16.5	0	1
22	5484	36	Azithromycin	1	61	6/2/2018	103.4	HEIGHT	16.1	16	0	1
22	5484	48	Azithromycin	1	72	5/26/2019	108.7	HEIGHT	17.95454545	16	0	1
22	5484	60	Azithromycin	1	81	2/18/2020	113.8	HEIGHT	19.05	16.5	0	1
22	5485	24	Azithromycin	0	43	5/5/2017	99.4	HEIGHT	14.77272727	15	1	1
22	5486	24	Azithromycin	1	7	5/5/2017	60.5	LENGTH	5.909090909	13	1	1
22	5486	48	Azithromycin	1	26	5/27/2019	80.6	LENGTH	11.72727273	15	0	1
22	5486	60	Azithromycin	1	35	2/18/2020	88.8	HEIGHT	13.3	14.2	0	1
22	5487	0	Azithromycin	1	36	4/10/2015	100.2	HEIGHT	14.8	14.5	0	1
22	5487	24	Azithromycin	1	64	5/5/2017	114.7	HEIGHT	18.35	15.5	0	1
22	5487	36	Azithromycin	1	77	6/2/2018	119.1	HEIGHT	20.5	15.5	0	1
22	5487	48	Azithromycin	1	88	5/26/2019	124.1	HEIGHT	23.04545455	16.5	0	1
22	5487	60	Azithromycin	1	97	2/18/2020	128	HEIGHT	26.3	17.2	0	1
22	5489	0	Azithromycin	0	36	4/10/2015	79.7	HEIGHT	9.8	13	1	1
22	5489	24	Azithromycin	0	55	5/5/2017	98.7	HEIGHT	14.95454545	14.5	0	1
22	5489	48	Azithromycin	0	79	5/27/2019	112.6	HEIGHT	19.5	15.5	0	1
22	5489	60	Azithromycin	0	88	2/18/2020	117.4	HEIGHT	22.2	15.7	0	1
22	5490	0	Azithromycin	1	11	4/10/2015	73.1	LENGTH	7.4	12.5	1	1
22	5490	12	Azithromycin	1	34	7/28/2016	82.9	HEIGHT		12.2	0	1
22	5490	24	Azithromycin	1	38	5/5/2017	85.5	HEIGHT	10.45	14	0	1
22	5490	36	Azithromycin	1	51	6/2/2018	91.6	HEIGHT	11.6	13.5	0	1
22	5490	48	Azithromycin	1	62	5/26/2019	97.6	HEIGHT	12.13636364	12.5	0	1
22	5490	60	Azithromycin	1	71	2/18/2020	100.5	HEIGHT	15.05	14	0	1
22	5494	24	Azithromycin	1	43	5/5/2017	101.7	HEIGHT	15.25	15	1	1

22	5498	12	Azithromycin	0	18	7/26/2016	79.6	HEIGHT		14.5		1	1
22	5499	0	Azithromycin	1	36	4/10/2015	84.8	HEIGHT	10.5	13.5		0	1
22	5501	24	Azithromycin	1	55	5/5/2017	102	HEIGHT	18.65	17.5		1	1
22	5502	12	Azithromycin	0	11	7/26/2016	77	LENGTH		15.8		0	1
22	5502	24	Azithromycin	0	24	5/5/2017	83.8	HEIGHT	10.15	14.5		0	1
22	5503	12	Azithromycin	1	1	7/26/2016	63.5	LENGTH		14		0	1
22	5505	0	Azithromycin	0	12	4/10/2015	70.4	LENGTH	6.4	11.5		0	1
22	5509	24	Azithromycin	1	55	5/5/2017	100.7	HEIGHT	17.1	17		1	1
22	5510	12	Azithromycin	0	1	7/26/2016	67.4	LENGTH		16.5	30	0	1
22	5512	24	Azithromycin	0	43	5/5/2017	102	HEIGHT	14	15		1	1
22	5513	12	Azithromycin	0	9	7/26/2016	70.6	LENGTH		16.5		1	1
22	5513	24	Azithromycin	0	22	5/5/2017	78.6	HEIGHT	8.590909091	12.5		1	1
22	5513	60	Azithromycin	0	55	2/18/2020	100.9	HEIGHT	13.25	13		0	1
22	5514	0	Azithromycin	0	9	4/10/2015	75.8	HEIGHT	10.2	15		1	1
22	5514	12	Azithromycin	0	25	7/26/2016	86.9	HEIGHT		17.5		0	1
22	5514	36	Azithromycin	0	51	6/2/2018	97.3	HEIGHT	14.2	15		0	1
22	5514	48	Azithromycin	0	62	5/26/2019	102.2	HEIGHT	15.36363636	15		0	1
22	5514	60	Azithromycin	0	71	2/18/2020	105.3	HEIGHT	16.3	14		0	1
22	5516	0	Azithromycin	0	12	4/10/2015	75.3	LENGTH	9	14		1	1
22	5516	12	Azithromycin	0	30	7/26/2016	84	HEIGHT		16.8		0	1
22	5516	24	Azithromycin	0	43	5/5/2017	92.1	HEIGHT	13.95	15.5		0	1
22	5516	36	Azithromycin	0	56	6/2/2018	100.4	HEIGHT	15.85	15.5		0	1
22	5516	48	Azithromycin	0	67	5/26/2019	107	HEIGHT	18.09090909	15.5		0	1
22	5516	60	Azithromycin	0	76	2/18/2020	113.3	HEIGHT	18.85	14.4		0	1
22	5517	12	Azithromycin	1	18	7/26/2016	73.5	HEIGHT		16.7		1	1
22	5517	36	Azithromycin	1	44	6/2/2018	86.5	HEIGHT	11.55	15.5		0	1
22	5518	24	Azithromycin	0	10	5/5/2017	67.6	LENGTH	7.272727273	14.5	54	1	1
22	5518	48	Azithromycin	0	34	5/26/2019	85.7	HEIGHT	11	13.5	54	0	1
22	5519	24	Azithromycin	1	21	5/5/2017	73.6	HEIGHT	9.318181818	13.5		1	1
22	5519	60	Azithromycin	1	53	2/18/2020	100	HEIGHT	14.15	14.5		1	1
22	5520	0	Azithromycin	0	24	4/10/2015	101.6	HEIGHT	14	14.5		1	1
22	5523	0	Azithromycin	0	24	4/10/2015	81	HEIGHT	9.6	13.5	12	1	1
22	5525	0	Azithromycin	0	8	4/10/2015	66	LENGTH	7.25	13.5		1	1
22	5525	24	Azithromycin	0	31	5/5/2017	84.9	HEIGHT	12.25	16		0	1
22	5525	36	Azithromycin	0	44	6/2/2018	93.9	HEIGHT	14.85	16		0	1
22	5525	60	Azithromycin	0	64	2/18/2020	105.8	HEIGHT	17.05	14.5		0	1
22	5526	24	Azithromycin	1	52	5/5/2017	110.5	HEIGHT	17	15.5		1	1
22	5528	0	Azithromycin	1	2	4/10/2015	63.5	HEIGHT	5.8	12.5		1	1
22	5528	24	Azithromycin	1	31	5/5/2017	83.6	HEIGHT	8.7	12		0	1
22	5528	36	Azithromycin	1	44	6/2/2018	91.5	HEIGHT	11.85	14.5		1	1

22	5528	48	Azithromycin	1	55	5/26/2019	97.2	HEIGHT	13.81818182	14.5		0	1
22	5528	60	Azithromycin	1	56	2/18/2020	100.4	HEIGHT	14.2	14.2		0	1
22	5529	12	Azithromycin	1	1	7/26/2016	64.8	LENGTH		17.2		0	1
22	5529	24	Azithromycin	1	14	5/5/2017	72.1	LENGTH	7.4	13		0	1
22	5529	36	Azithromycin	1	27	6/2/2018	75.5	HEIGHT	8.45	13		0	1
22	5531	0	Azithromycin	1	36	4/10/2015	92.8	HEIGHT	12.55	15		1	1
22	5531	24	Azithromycin	1	64	5/5/2017	108.7	HEIGHT	16.22727273	15.5		0	1
22	5531	36	Azithromycin	1	77	6/2/2018	113.5	HEIGHT	17.25	15		0	1
22	5531	48	Azithromycin	1	88	5/26/2019	118.5	HEIGHT	20.27272727	16		0	1
22	5531	60	Azithromycin	1	97	2/18/2020	123.1	HEIGHT	21.65	16.3		0	1
22	5533	0	Azithromycin	1	24	4/10/2015	86.2	HEIGHT	10.85	14		0	1
22	5533	24	Azithromycin	1	55	5/5/2017	103.1	HEIGHT	15.40909091	15.5		1	1
22	5533	36	Azithromycin	1	68	6/2/2018	111.5	HEIGHT	17.95	15.5		0	1
22	5533	48	Azithromycin	1	79	5/26/2019	117.2	HEIGHT	20.31818182	16		0	1
22	5534	12	Azithromycin	1	27	7/26/2016	94.5	HEIGHT		17.6		1	1
22	5535	0	Azithromycin	0	48	4/10/2015	113.5	HEIGHT	17.85	15		1	1
22	5536	12	Azithromycin	1	6	7/26/2016	64.7	LENGTH		14.6		0	1
22	5536	48	Azithromycin	1	39	5/26/2019	82.5	LENGTH	9.863636364	12.5		1	1
22	5536	60	Azithromycin	1	49	2/18/2020	88.5	HEIGHT	12.5	13.5		0	1
22	5537	24	Azithromycin	0	21	5/5/2017	81.2	HEIGHT	11.3	16		1	1
22	5538	12	Azithromycin	1	9	7/26/2016	68.9	LENGTH		15		0	1
22	5538	24	Azithromycin	1	22	5/5/2017	78.3	HEIGHT	8.590909091	13		0	1
22	5538	36	Azithromycin	1	29	6/2/2018	87.2	HEIGHT	10.2	13.5		0	1
22	5538	48	Azithromycin	1	40	5/26/2019	91.2	HEIGHT	12.22727273	13.5		1	1
22	5538	60	Azithromycin	1	49	2/18/2020	96.3	HEIGHT	13.7	14.8		1	1
22	5539	12	Azithromycin	1	7	7/26/2016	71.8	LENGTH		18		0	1
22	5539	48	Azithromycin	1	39	5/26/2019	92.5	HEIGHT	14.36363636	15		1	1
22	5539	60	Azithromycin	1	49	2/18/2020	98.1	HEIGHT	16.25	14.7		0	1
22	5542	0	Azithromycin	0	24	4/10/2015	97	HEIGHT	12.55	13		1	1
22	5544	12	Azithromycin	0	54	7/26/2016	108.5	HEIGHT		18.7		1	1
22	5546	0	Azithromycin	0	24	4/10/2015	89.2	HEIGHT	13.15	15.5	24	0	1
22	5549	12	Azithromycin	1	42	7/26/2016	97.4	HEIGHT		17.7		1	1
22	5550	0	Azithromycin	0	48	4/10/2015	106.7	HEIGHT	16.25	15		0	1
22	5550	12	Azithromycin	0	54	7/26/2016	115.4	HEIGHT		18.6		0	1
22	5550	24	Azithromycin	0	67	5/5/2017	120.3	HEIGHT	20.05	16		0	1
22	5550	36	Azithromycin	0	80	6/2/2018	125.5	HEIGHT	22.4	16		0	1
22	5551	12	Azithromycin	0	15	7/28/2016	79.8	LENGTH		14.5		1	1
22	5551	48	Azithromycin	0	52	5/26/2019	97.2	HEIGHT	14.68181818	14		1	1
22	5552	12	Azithromycin	0	54	7/26/2016	97.1	HEIGHT		18.3		1	1
22	5554	0	Azithromycin	1	6	4/10/2015	71.1	LENGTH	7.9	13.5		0	1

22	5554	12	Azithromycin	1	21	7/26/2016	85.8	HEIGHT		16.6		0	1
22	5554	24	Azithromycin	1	34	5/5/2017	91	HEIGHT	13.77272727	15.5		0	1
22	5554	36	Azithromycin	1	47	6/2/2018	98.7	HEIGHT	15.4	15.5		1	1
22	5554	48	Azithromycin	1	58	5/27/2019	105.6	HEIGHT	16.63636364	15		0	1
22	5554	60	Azithromycin	1	67	2/18/2020	111.2	HEIGHT	18	15.2		0	1
22	5556	24	Azithromycin	0	10	5/5/2017	74.3	LENGTH	8.75	14.5	30	1	1
22	5557	0	Azithromycin	1	8	4/10/2015	65.6	LENGTH	6.05	12		0	1
22	5559	12	Azithromycin	0	54	7/26/2016	116.2	HEIGHT		19		1	1
22	5561	24	Azithromycin	1	1	5/5/2017	51.3	LENGTH	4.681818182	11		1	1
22	5561	36	Azithromycin	1	14	6/2/2018	75	LENGTH	9.5	15.5		0	1
22	5561	48	Azithromycin	1	25	5/26/2019	83.1	HEIGHT	11.54545455	15		0	1
22	5561	60	Azithromycin	1	34	2/18/2020	89.4	LENGTH	12.95	15.7		1	1
22	5564	0	Azithromycin	1	48	4/10/2015	91	HEIGHT	14.45	15		0	1
22	5564	24	Azithromycin	1	76	5/5/2017	104.2	HEIGHT	16.95	15.5		0	1
22	5564	36	Azithromycin	1	89	6/2/2018	108.5	HEIGHT	18.45	15.5		0	1
22	5564	48	Azithromycin	1	100	5/26/2019	114.2	HEIGHT	20.45454545	15		0	1
22	5564	60	Azithromycin	1	109	2/18/2020	118.6	HEIGHT	20.9	15.5		0	1
22	5565	0	Azithromycin	1	30	4/10/2015	92.2	HEIGHT	11.6	13.5	18	1	1
22	5566	0	Azithromycin	0	12	4/10/2015	74.8	LENGTH	7.35	11.5		1	1
22	5567	12	Azithromycin	1	9	7/26/2016	68.1	HEIGHT		17		0	1
22	5567	24	Azithromycin	1	22	5/5/2017	76.9	HEIGHT	8.363636364	13.5		0	1
22	5567	36	Azithromycin	1	35	6/2/2018	83.9	HEIGHT	11.1	14		0	1
22	5568	0	Azithromycin	0	12	4/10/2015	69.2	LENGTH	6.65	12.5	18	0	1
22	5569	0	Azithromycin	0	36	6/10/2015	88.1	HEIGHT	12.45	14		0	1
22	5569	12	Azithromycin	0	42	7/26/2016	97.2	HEIGHT		16		0	1
22	5569	24	Azithromycin	0	55	5/5/2017	103.5	HEIGHT	13.95	14		0	1
22	5569	36	Azithromycin	0	68	6/2/2018	109.4	HEIGHT	16.05	13.5		0	1
22	5569	48	Azithromycin	0	79	5/26/2019	114.5	HEIGHT	18.13636364	15		0	1
22	8017	48	Azithromycin	1	10	5/26/2019	67.6	LENGTH	7.409090909	13		1	1
22	8019	60	Azithromycin	1	31	2/18/2020	83.9	HEIGHT	12.7	15.4		1	1
22	8082	60	Azithromycin	1	41	2/21/2020	89.5	HEIGHT	13.77272727	16		1	1
22	8086	60	Azithromycin	0	31	2/18/2020	74.2	LENGTH	6.1	10.5		1	1
22	8121	36	Azithromycin	0	11	6/2/2018	69.1	LENGTH	6.9	12		1	1
22	8121	48	Azithromycin	0	22	5/26/2019	78.2	LENGTH	9.863636364	14		0	1
22	8145	48	Azithromycin	0	52	5/26/2019	94.6	HEIGHT	13.68181818	14.5		1	1
22	8150	60	Azithromycin	0	14	2/18/2020	75.8	LENGTH	9.1	14		1	1
22	8176	60	Azithromycin	1	9	2/18/2020	68.5	LENGTH	7.8	12.5		1	1
22	8199	60	Azithromycin	0	22	2/21/2020	83.8	HEIGHT	11.86363636	14.5		1	1
22	8213	36	Azithromycin	1	41	6/2/2018	82.2	HEIGHT	9.6	12.5		1	1
22	8213	48	Azithromycin	1	52	5/26/2019	88.9	HEIGHT	10.45454545	13		1	1

22	8251	36	Azithromycin	0	6	6/2/2018	64.5	LENGTH	6.85	14	1	1
22	8288	48	Azithromycin	0	28	5/26/2019	80	LENGTH	8.909090909	13.5	1	1
22	8289	36	Azithromycin	0	38	6/2/2018	88.7	HEIGHT	12.9	16	1	1
22	8297	48	Azithromycin	0	45	5/26/2019	89.8	HEIGHT	12	14	1	1
22	8314	60	Azithromycin	0	27	2/18/2020	88.2	HEIGHT	14.3	17	1	1
22	8332	48	Azithromycin	0	49	5/26/2019	99.6	LENGTH	16	16	1	1
22	8361	60	Azithromycin	1	14	2/18/2020	74.9	LENGTH	9.95	14.5	1	1
22	8389	36	Azithromycin	1	25	6/2/2018	83.2	LENGTH	10.2	13.5	1	1
22	8389	48	Azithromycin	1	35	5/26/2019	88.7	HEIGHT	11.81818182	13.5	1	1
22	8404	60	Azithromycin	0	32	2/21/2020	77.7	LENGTH	10.90909091	15	1	1
22	8421	36	Azithromycin	1	56	6/2/2018	99.6	HEIGHT	13.2	14	1	1
22	8425	48	Azithromycin	1	26	5/26/2019	84.1	HEIGHT	12.40909091	16	1	1
22	8430	36	Azithromycin	1	7	6/2/2018	61.4	LENGTH	4.5	9.5	1	1
22	8430	48	Azithromycin	1	18	5/27/2019	71	LENGTH	5.954545455	10	0	1
22	8430	60	Azithromycin	1	27	2/18/2020	74.7	LENGTH	7.9	11.9	0	1
22	8434	36	Azithromycin	0	56	6/2/2018	96.7	HEIGHT	14.4	15.5	1	1
22	8446	36	Azithromycin	0	44	6/2/2018	96.9	HEIGHT	13.9	15.5	1	1
22	8449	36	Azithromycin	0	15	6/2/2018	73.5	LENGTH	8.4	13.5	1	1
22	8464	60	Azithromycin	0	43	2/18/2020	97.1	HEIGHT	15.85	15.5	1	1
22	8487	48	Azithromycin	1	16	5/26/2019	77.2	HEIGHT	9.409090909	14.5	1	1
22	8501	60	Azithromycin	0	20	2/18/2020	81.7	HEIGHT	10.3	13.5	1	1
22	8570	60	Azithromycin	1	10	2/18/2020	68.7	LENGTH	6.85	12	1	1
22	8580	48	Azithromycin	0	59	5/26/2019	97.8	HEIGHT	13.63636364	13.5	1	1
22	8586	48	Azithromycin	0	55	5/26/2019	95.6	HEIGHT	13.68181818	14	1	1
22	8635	48	Azithromycin	1	49	5/26/2019	99.6	HEIGHT	16.13636364	16.5	1	1
22	8679	60	Azithromycin	1	44	2/21/2020	97.7	HEIGHT	14.31818182	14.1	1	1
22	8693	36	Azithromycin	1	56	6/2/2018	92.8	HEIGHT	14.5	14.5	1	1
22	8709	48	Azithromycin	1	21	5/26/2019	78.3	HEIGHT	8.909090909	13	1	1
22	8711	36	Azithromycin	1	15	6/2/2018	83.7	LENGTH	11.9	15.5	1	1
22	8711	48	Azithromycin	1	26	5/26/2019	93.6	HEIGHT	14	15	1	1
22	8721	36	Azithromycin	1	39	6/2/2018	95.2	LENGTH	15.65	18	1	1
22	8723	36	Azithromycin	1	35	6/2/2018	89.1	HEIGHT	14.2	16	1	1
22	8748	36	Azithromycin	1	15	6/2/2018	74.3	LENGTH	8.3	13	1	1
22	8748	60	Azithromycin	1	35	2/18/2020	89.2	HEIGHT	13.1	14.5	1	1
22	8756	60	Azithromycin	0	14	2/18/2020	81	HEIGHT	10	13.5	1	1
22	8771	60	Azithromycin	1	31	2/21/2020	85.2	HEIGHT	12.86363636	15	1	1
22	8772	60	Azithromycin	1	32	2/18/2020	91.9	HEIGHT	13.35	13.8	1	1
22	8779	48	Azithromycin	0	31	5/26/2019	79.9	HEIGHT	10.04545455	14.5	1	1
22	8802	36	Azithromycin	1	44	6/2/2018	96.8	HEIGHT	12.95	14	1	1
22	8818	36	Azithromycin	0	25	6/2/2018	81	HEIGHT	9.9	13.5	1	1

22	8822	48	Azithromycin	1	32	5/26/2019	84.2	HEIGHT	11.68181818	14.5		1	1
22	8874	48	Azithromycin	0	55	5/26/2019	102.6	HEIGHT	15.13636364	14.5		0	1
22	8875	60	Azithromycin	0	31	2/21/2020	81.8	HEIGHT	7.954545455	10.2		1	1
22	8876	60	Azithromycin	0	31	2/21/2020	85.2	HEIGHT	12.5	14		1	1
22	8881	48	Azithromycin	0	44	5/26/2019	99.1	HEIGHT	15.77272727	15.5		1	1
22	8910	60	Azithromycin	0	54	2/21/2020	98.1	HEIGHT	14.09090909	13.5		1	1
22	8912	36	Azithromycin	0	27	6/2/2018	80.7	HEIGHT	10.6	14		1	1
22	8920	60	Azithromycin	0	25	2/18/2020	89	HEIGHT	13.3	14.9		1	1
22	8933	48	Azithromycin	1	7	5/26/2019	68.6	LENGTH	7.409090909	13		1	1
22	8934	60	Azithromycin	0	8	2/21/2020	64	LENGTH	6.363636364	11.6		1	1
22	8986	60	Azithromycin	0	14	2/18/2020	80.9	HEIGHT	9.9	14		1	1
22	9019	36	Azithromycin	1	16	6/2/2018	75.6	LENGTH	8.95	15		1	1
22	9055	36	Azithromycin	1	13	6/2/2018	70.4	HEIGHT	8.15	14		1	1
22	9055	60	Azithromycin	1	33	2/18/2020	83.2	HEIGHT	11.95	15		1	1
22	9063	36	Azithromycin	1	53	6/2/2018	110.1	HEIGHT	17.3	14.5		1	1
22	9134	48	Azithromycin	1	10	5/26/2019	67.6	LENGTH	7.136363636	12.5		1	1
22	9137	60	Azithromycin	0	31	2/21/2020	85	HEIGHT	11.86363636	13.7		1	1
22	9142	60	Azithromycin	0	6	2/18/2020	66.6	LENGTH	5.85	10.5		1	1
22	9144	36	Azithromycin	1	53	6/2/2018	108.6	HEIGHT	16.3	14.5		1	1
22	9145	36	Azithromycin	0	1	6/2/2018	58.2	LENGTH	5.65	13.5		1	1
22	9145	60	Azithromycin	0	21	2/18/2020	81.4	HEIGHT	11.05	15		0	1
22	9160	60	Azithromycin	0	35	2/21/2020	83.2	HEIGHT	12.09090909	14		1	1
22	9175	36	Azithromycin	1	34	6/2/2018	83.8	HEIGHT	9.5	12		1	1
22	9175	60	Azithromycin	1	54	2/18/2020	94.2	HEIGHT	12.05	12.9		1	1
22	9209	60	Azithromycin	0	23	2/21/2020	70.7	LENGTH	7.909090909	12.5		1	1
22	9275	48	Azithromycin	0	18	5/26/2019	76.2	LENGTH	8.090909091	12.5		1	1
22	9327	60	Azithromycin	1	14	2/18/2020	70.2	HEIGHT	8.15	13		1	1
22	9336	48	Azithromycin	1	8	5/26/2019	73.7	LENGTH	10.27272727	16.5		1	1
22	9336	60	Azithromycin	1	14	2/18/2020	80.7	HEIGHT	11.45	15.6		0	1
22	9380	48	Azithromycin	1	21	5/26/2019	75.3	LENGTH	9.818181818	14		1	1
22	9392	48	Azithromycin	0	24	5/26/2019	91.5	HEIGHT	13.22727273	14		1	1
22	9397	60	Azithromycin	1	32	2/18/2020	75.4	HEIGHT	8.95	13.5		1	1
22	9400	36	Azithromycin	1	12	6/2/2018	72.3	LENGTH	8.6	13.5		1	1
22	9402	48	Azithromycin	1	35	5/26/2019	82.9	HEIGHT	11.18181818	14.5		1	1
22	9419	36	Azithromycin	1	25	6/2/2018	76.5	LENGTH	6.45	11		1	1
22	9419	48	Azithromycin	1	35	5/26/2019	78.5	HEIGHT	7.045454545	11		1	1
22	9431	36	Azithromycin	1	10	6/2/2018	66.3	LENGTH	6.1	10.5	54	1	1
22	9456	36	Azithromycin	0	47	6/2/2018	94.5	HEIGHT	13.7	14		1	1
22	9477	48	Azithromycin	1	45	5/26/2019	95	HEIGHT	14.22727273	15		1	1
22	9484	60	Azithromycin	0	26	2/18/2020	86.3	LENGTH	12.35	14.6		1	1

22	9491	48	Azithromycin	1	48	5/26/2019	111.6	HEIGHT	17.59090909	15.5	1	1
22	9499	36	Azithromycin	1	38	6/2/2018	89.5	HEIGHT	12.95	15	1	1
22	9503	60	Azithromycin	1	19	2/21/2020	78.7	LENGTH	10.45454545	14	1	1
22	9522	60	Azithromycin	0	35	2/21/2020	77.1	LENGTH	9.5	12.5	1	1
22	9545	48	Azithromycin	0	40	5/26/2019	90.6	HEIGHT	12.22727273	15	1	1
22	9583	36	Azithromycin	0	15	6/2/2018	73.2	HEIGHT	7.5	13	1	1
23	5582	0	Azithromycin	0	12	4/12/2015	69	LENGTH	7.55	14	1	1
23	5582	12	Azithromycin	0	26	7/20/2016	78.6	HEIGHT		13	0	1
23	5582	24	Azithromycin	0	40	6/10/2017	83.3	HEIGHT	10.4	14	0	1
23	5582	36	Azithromycin	0	52	6/18/2018	89.1	HEIGHT	12.68181818	14.5	0	1
23	5582	48	Azithromycin	0	63	4/30/2019	93.4	LENGTH	13.85	15	0	1
23	5582	60	Azithromycin	0	72	2/24/2020	98.7	HEIGHT	15.86363636	15.5	0	1
23	5583	0	Azithromycin	0	36	4/12/2015	86.2	HEIGHT	10.2	12	0	1
23	5583	24	Azithromycin	0	64	6/11/2017	100.7	HEIGHT	13.8	13	0	1
23	5583	36	Azithromycin	0	76	6/18/2018	107.4	HEIGHT	14.90909091	12.5	0	1
23	5583	48	Azithromycin	0	87	4/30/2019	112.5	HEIGHT	15.5	12.5	0	1
23	5588	0	Azithromycin	1	48	4/12/2015	99.5	HEIGHT	14.1	14.5	1	1
23	5588	48	Azithromycin	1	99	4/29/2019	123.2	HEIGHT	21.86363636	15.5	0	1
23	5588	60	Azithromycin	1	108	3/9/2020	127.3	HEIGHT	23.75	16	0	1
23	5589	12	Azithromycin	0	56	7/24/2016	98	HEIGHT		13.5	1	1
23	5592	0	Azithromycin	1	36	4/12/2015	82.2	HEIGHT	9.3	12.5	0	1
23	5592	12	Azithromycin	1	54	7/20/2016	89.1	HEIGHT		13.5	0	1
23	5592	24	Azithromycin	1	68	6/11/2017	97.3	HEIGHT	12.8	13.5	0	1
23	5592	36	Azithromycin	1	80	6/18/2018	101.5	HEIGHT	13.77272727	13.5	0	1
23	5592	48	Azithromycin	1	91	4/29/2019	103.3	HEIGHT	14.40909091	13.5	0	1
23	5592	60	Azithromycin	1	100	2/24/2020	107.7	HEIGHT	14.68181818	13.2	0	1
23	5593	0	Azithromycin	1	48	4/12/2015	97.4	HEIGHT	11.9	12.5	0	1
23	5593	12	Azithromycin	1	62	7/20/2016	103	HEIGHT		13	0	1
23	5593	24	Azithromycin	1	76	6/11/2017	109.1	HEIGHT	15.55	13.5	0	1
23	5593	48	Azithromycin	1	99	4/29/2019	117.3	HEIGHT	17.15	13.5	0	1
23	5593	60	Azithromycin	1	108	2/24/2020	121.1	HEIGHT	18.77272727	13.4	0	1
23	5594	12	Azithromycin	1	20	7/20/2016	68.5	LENGTH		12.5	1	1
23	5596	0	Azithromycin	1	54	6/14/2015	109.6	HEIGHT	16.95	15	0	1
23	5596	12	Azithromycin	1	62	7/21/2016	116.2	HEIGHT		15.5	0	1
23	5596	36	Azithromycin	1	88	6/19/2018	130.8	HEIGHT	24.90909091	16	0	1
23	5596	48	Azithromycin	1	99	4/29/2019	127.6	HEIGHT	22.85	16.5	0	1
23	5598	0	Azithromycin	0	36	4/12/2015	94.5	HEIGHT	12.7	14	1	1
23	5598	12	Azithromycin	0	50	7/21/2016	105.2	HEIGHT		14.5	0	1
23	5598	24	Azithromycin	0	64	6/10/2017	111.5	HEIGHT	16.35	14.5	0	1
23	5598	36	Azithromycin	0	76	6/19/2018	117.5	HEIGHT	18.13636364	14	0	1

23	5598	48	Azithromycin	0	87	4/29/2019	119.9	HEIGHT	19.7	14		0	1
23	5598	60	Azithromycin	0	96	3/9/2020	126	HEIGHT	22.2	15		0	1
23	5599	12	Azithromycin	0	12	7/20/2016	70.7	LENGTH		13		1	1
23	5599	24	Azithromycin	0	25	6/10/2017	78.5	HEIGHT	8.4	12.5		0	1
23	5599	36	Azithromycin	0	37	6/18/2018	84.3	LENGTH	10.22727273	13		0	1
23	5599	48	Azithromycin	0	48	6/8/2019	93.2	HEIGHT	12.13636364	14		0	1
23	5599	60	Azithromycin	0	58	2/24/2020	98.3	HEIGHT	13.36363636	13.2		0	1
23	5600	0	Azithromycin	0	36	5/10/2015	96.3	HEIGHT	13.95	15		0	1
23	5600	12	Azithromycin	0	51	7/20/2016	105.6	HEIGHT		14		0	1
23	5600	24	Azithromycin	0	82	6/10/2017	111.5	HEIGHT	16.1	14		0	1
23	5600	48	Azithromycin	0	105	4/29/2019	120.1	HEIGHT	19.35	14		0	1
23	5601	24	Azithromycin	0	17	6/10/2017	76.8	HEIGHT	8.4	12.5		1	1
23	5601	48	Azithromycin	0	40	4/29/2019	90.4	HEIGHT	12.85	14		1	1
23	5603	12	Azithromycin	1	7	7/21/2016	69.3	LENGTH		13.5		0	1
23	5606	0	Azithromycin	0	48	5/10/2015	73.1	HEIGHT	7.5	12		0	1
23	5606	24	Azithromycin	0	58	6/11/2017	90.2	HEIGHT	12.1	14.5		0	1
23	5606	36	Azithromycin	0	70	6/18/2018	96	HEIGHT	13.36363636	15		0	1
23	5606	48	Azithromycin	0	81	4/29/2019	101.1	HEIGHT	15	15		0	1
23	5606	60	Azithromycin	0	90	3/9/2020	105.9	HEIGHT	16.25	15		0	1
23	5608	12	Azithromycin	0	36	7/20/2016	93.7	HEIGHT		14		1	1
23	5609	12	Azithromycin	0	3	7/20/2016	70.9	LENGTH		12	54	0	1
23	5609	24	Azithromycin	0	12	6/10/2017	80	HEIGHT	9.45	13.5	54	0	1
23	5609	36	Azithromycin	0	24	6/18/2018	87.2	HEIGHT	12.5	14.5	54	0	1
23	5610	12	Azithromycin	0	24	7/24/2016	71.8	LENGTH		12		1	1
23	5613	24	Azithromycin	1	44	6/11/2017	100.8	HEIGHT	12.65	13		1	1
23	5614	12	Azithromycin	0	11	7/20/2016	75.5	LENGTH		12.5		0	1
23	5614	24	Azithromycin	0	17	6/11/2017	83.6	HEIGHT	10.6	14		1	1
23	5614	36	Azithromycin	0	29	6/18/2018	91.5	HEIGHT	12.95454545	15		1	1
23	5614	48	Azithromycin	0	40	6/8/2019	98.6	HEIGHT	14.18181818	14.7		1	1
23	5614	60	Azithromycin	0	49	2/24/2020	104.5	HEIGHT	14.63636364	13.7		0	1
23	5617	12	Azithromycin	0	33	7/21/2016	79.7	LENGTH		12		1	1
23	5618	24	Azithromycin	0	5	6/11/2017	64.5	LENGTH	6.3	12.5		1	1
23	5618	36	Azithromycin	0	17	6/18/2018	75.3	LENGTH	8.181818182	12.5		0	1
23	5618	48	Azithromycin	0	28	4/30/2019	79.4	HEIGHT	8.35	12		0	1
23	5620	0	Azithromycin	0	24	4/12/2015	77.6	HEIGHT	9.4	13		0	1
23	5620	36	Azithromycin	0	64	6/19/2018	97.5	HEIGHT	13.86363636	12.5		0	1
23	5620	48	Azithromycin	0	75	6/8/2019	102.5	HEIGHT	15.81818182	13.6		0	1
23	5620	60	Azithromycin	0	84	3/13/2020	105.4	HEIGHT	16.9	13.6		0	1
23	5622	12	Azithromycin	1	54	7/20/2016	86.3	HEIGHT		13		1	1
23	5625	12	Azithromycin	0	18	7/24/2016	79.9	LENGTH		12.5		1	1

23	5625	24	Azithromycin	0	32	6/13/2017	86.8	HEIGHT	9.1	12		1	1
23	5625	36	Azithromycin	0	44	6/18/2018	94.5	HEIGHT	10.72727273	11.5		1	1
23	5625	48	Azithromycin	0	55	4/29/2019	99.9	HEIGHT	12	12		1	1
23	5627	0	Azithromycin	0	42	4/12/2015	88.3	HEIGHT	10.9	13		1	1
23	5627	12	Azithromycin	0	57	7/20/2016	97.3	HEIGHT		13.5		0	1
23	5627	24	Azithromycin	0	71	6/10/2017	100.4	HEIGHT	13.05	13.5		0	1
23	5627	48	Azithromycin	0	94	6/8/2019	112.8	HEIGHT	16.63636364	14		0	1
23	5628	0	Azithromycin	0	54	5/10/2015	97	HEIGHT	12.85	14		0	1
23	5628	36	Azithromycin	0	95	6/18/2018	116.7	HEIGHT	17.18181818	14		0	1
23	5629	0	Azithromycin	0	48	6/14/2015	89.3	HEIGHT	11	13		0	1
23	5631	24	Azithromycin	0	2	6/11/2017	59.7	LENGTH	4.6	11.5		1	1
23	5632	0	Azithromycin	1	6	4/12/2015	66.7	LENGTH	6.85	12.5		0	1
23	5632	48	Azithromycin	1	57	6/8/2019	96.7	LENGTH	12.72727273	13.2		1	1
23	5636	24	Azithromycin	0	4	6/10/2017	59.1	LENGTH	6	12.5	42	1	1
23	5636	36	Azithromycin	0	15	6/18/2018	71.2	LENGTH	7.227272727	12	42	1	1
23	5637	0	Azithromycin	1	48	4/12/2015	91	HEIGHT	11.9	13.5		1	1
23	5637	48	Azithromycin	1	99	4/29/2019	117.8	HEIGHT	18.45	15		0	1
23	5641	0	Azithromycin	1	36	5/10/2015	81.7	HEIGHT	10.3	14		0	1
23	5641	24	Azithromycin	1	70	6/11/2017	95.7	HEIGHT	13.75	14		0	1
23	5641	36	Azithromycin	1	82	6/18/2018	101.1	HEIGHT	14.68181818	14		0	1
23	5641	48	Azithromycin	1	93	4/29/2019	107.6	HEIGHT	16.4	15		0	1
23	5641	60	Azithromycin	1	103	2/24/2020	112.5	HEIGHT	17.18181818	14		0	1
23	5642	0	Azithromycin	1	24	5/10/2015	81.2	HEIGHT	9.5	13.5		1	1
23	5642	36	Azithromycin	1	70	6/19/2018	104.7	HEIGHT	13.90909091	13.5		0	1
23	5642	60	Azithromycin	1	91	3/9/2020	113	HEIGHT	16.25	14		0	1
23	5644	0	Azithromycin	1	48	4/12/2015	94.8	HEIGHT	11.55	14		0	1
23	5644	12	Azithromycin	1	72	7/20/2016	103.5	HEIGHT		15		0	1
23	5644	48	Azithromycin	1	80	4/29/2019	122.6	HEIGHT	17.22727273	14.5		0	1
23	5644	60	Azithromycin	1	90	2/24/2020	126.8	HEIGHT	19.95454545	15		0	1
23	5646	12	Azithromycin	1	96	7/20/2016	108.5	HEIGHT		15		1	1
23	5647	0	Azithromycin	1	24	4/12/2015	75.5	HEIGHT	9.55	15	18	0	1
23	5647	12	Azithromycin	1	41	7/20/2016	85.8	LENGTH		16	18	0	1
23	5648	0	Azithromycin	1	24	5/10/2015	85.3	HEIGHT	10.55	13		1	1
23	5648	12	Azithromycin	1	42	7/20/2016	93.7	HEIGHT		13.5		0	1
23	5648	36	Azithromycin	1	64	6/19/2018	107.2	HEIGHT	15	13.5		0	1
23	5648	48	Azithromycin	1	75	4/30/2019	111.3	HEIGHT	16.6	14		0	1
23	5650	0	Azithromycin	0	54	4/12/2015	99.9	HEIGHT	14.3	15.5		0	1
23	5650	24	Azithromycin	0	83	6/10/2017	110.8	HEIGHT	17.2	15.5		0	1
23	5650	48	Azithromycin	0	106	4/29/2019	121.2	HEIGHT	21.54545455	16		0	1
23	5650	60	Azithromycin	0	115	2/24/2020	124.5	HEIGHT	23.04545455	16.5		0	1

23	5651	12	Azithromycin	1	20	7/20/2016	84.2	HEIGHT		14.5		1	1
23	5652	12	Azithromycin	1	14	7/24/2016	71.9	LENGTH		12		1	1
23	5652	36	Azithromycin	1	40	6/18/2018	86.1	LENGTH	10.09090909	12.5		1	1
23	5654	0	Azithromycin	1	24	4/12/2015	79	HEIGHT	9.5	14		0	1
23	5654	12	Azithromycin	1	39	7/20/2016	88.5	HEIGHT		14		0	1
23	5654	24	Azithromycin	1	53	6/10/2017	96.2	HEIGHT	12.45	14		1	1
23	5654	48	Azithromycin	1	76	4/29/2019	107.3	HEIGHT	14.95	13.5		0	1
23	5656	12	Azithromycin	1	1	7/20/2016	59.2	HEIGHT		10.5		0	1
23	5656	24	Azithromycin	1	15	6/10/2017	67.3	HEIGHT	6.15	11		0	1
23	5656	36	Azithromycin	1	27	6/18/2018	76.4	LENGTH	8.227272727	12		0	1
23	5658	12	Azithromycin	1	18	7/27/2016	70.6	LENGTH		11		1	1
23	5659	0	Azithromycin	0	48	4/12/2015	86.1	HEIGHT	9.7	12.5		0	1
23	5661	0	Azithromycin	0	24	4/12/2015	83	HEIGHT	8.9	12.5		0	1
23	5661	12	Azithromycin	0	38	7/20/2016	89.2	HEIGHT		13.5		0	1
23	5661	24	Azithromycin	0	52	6/11/2017	96.7	HEIGHT	12.75	13		0	1
23	5661	36	Azithromycin	0	64	6/18/2018	101.9	HEIGHT	14.31818182	13.5		0	1
23	5661	48	Azithromycin	0	75	4/29/2019	105.6	HEIGHT	14.86363636	13.5		0	1
23	5661	60	Azithromycin	0	84	2/24/2020	110.9	HEIGHT	15.09090909	12.3		0	1
23	5664	24	Azithromycin	0	58	6/10/2017	98.6	HEIGHT	12.85	14		1	1
23	5666	0	Azithromycin	1	36	5/10/2015	82.5	HEIGHT	9.65	13		1	1
23	5666	12	Azithromycin	1	51	7/20/2016	87.2	HEIGHT		13		1	1
23	5669	0	Azithromycin	1	48	5/10/2015	92.1	HEIGHT	9.9	11		1	1
23	5669	12	Azithromycin	1	66	7/20/2016	101.7	HEIGHT		12		0	1
23	5669	48	Azithromycin	1	99	4/30/2019	117.7	HEIGHT	16.25	13.5		0	1
23	5672	0	Azithromycin	0	48	4/12/2015	99.9	HEIGHT	15.65	15		0	1
23	5672	12	Azithromycin	0	67	7/20/2016	106.3	HEIGHT		14.5		0	1
23	5672	24	Azithromycin	0	77	6/11/2017	112.3	HEIGHT	17.3	14.5		0	1
23	5673	0	Azithromycin	1	48	4/12/2015	116.6	HEIGHT	16.15	13.5		0	1
23	5675	0	Azithromycin	0	54	6/14/2015	96.8	HEIGHT	13.5	14.5		1	1
23	5676	0	Azithromycin	0	24	4/12/2015	70.7	LENGTH	6.8	11	48	1	1
23	5676	12	Azithromycin	0	43	7/20/2016	76.9	LENGTH		12	48	0	1
23	5678	0	Azithromycin	0	36	4/12/2015	90.7	HEIGHT	12.1	13.5		1	1
23	5679	12	Azithromycin	0	1	7/20/2016	54.3	LENGTH		10		0	1
23	5679	24	Azithromycin	0	11	6/11/2017	69.3	HEIGHT	6.3	11		0	1
23	5679	36	Azithromycin	0	21	6/18/2018	77.8	LENGTH	8.090909091	11.5		0	1
23	5679	48	Azithromycin	0	32	4/30/2019	83.2	HEIGHT	9.25	12		1	1
23	5680	0	Azithromycin	1	30	5/10/2015	73.2	HEIGHT	7.35	11.5	24	1	1
23	5681	0	Azithromycin	1	48	4/12/2015	91	HEIGHT	10.85	13.5		0	1
23	5681	48	Azithromycin	1	81	4/29/2019	113.9	HEIGHT	15.81818182	14		0	1
23	5683	0	Azithromycin	0	3	4/12/2015	67.4	LENGTH	7	12.5		0	1

23	5683	12	Azithromycin	0	14	7/20/2016	77.2	HEIGHT		12.5		0	1
23	5683	24	Azithromycin	0	28	6/11/2017	81.7	HEIGHT	9	12		0	1
23	5683	36	Azithromycin	0	40	6/18/2018	87	HEIGHT	10.40909091	12		1	1
23	5683	48	Azithromycin	0	51	4/29/2019	91.9	HEIGHT	11.25	12		0	1
23	5683	60	Azithromycin	0	60	2/24/2020	95.9	HEIGHT	11.81818182	11.3		0	1
23	5684	24	Azithromycin	0	28	6/13/2017	80.5	HEIGHT	10.15	13.5		1	1
23	5685	24	Azithromycin	1	3	6/10/2017	62.8	LENGTH	6.2	12		1	1
23	5685	36	Azithromycin	1	15	6/18/2018	79.8	LENGTH	9.045454545	12.5		1	1
23	5685	48	Azithromycin	1	26	4/30/2019	86.5	HEIGHT	11.55	14		0	1
23	5685	60	Azithromycin	1	35	3/9/2020	94.9	HEIGHT	14	15.5		0	1
23	5686	24	Azithromycin	1	6	6/13/2017	71.6	HEIGHT	7.8	13.5		1	1
23	5686	60	Azithromycin	1	35	3/9/2020	93.5	HEIGHT	12.4	14		1	1
23	5687	12	Azithromycin	1	6	7/20/2016	69.7	LENGTH		14		0	1
23	5687	24	Azithromycin	1	17	6/10/2017	77.2	HEIGHT	8.8	14		1	1
23	5687	36	Azithromycin	1	29	6/18/2018	86.5	LENGTH	11.86363636	14.5		1	1
23	5687	48	Azithromycin	1	40	6/8/2019	94.2	HEIGHT	13.77272727	16		1	1
23	5687	60	Azithromycin	1	49	2/24/2020	98.7	HEIGHT	15	14.9		0	1
23	5688	24	Azithromycin	0	52	6/13/2017	101.6	HEIGHT	14.15	14		1	1
23	5689	0	Azithromycin	1	48	4/12/2015	93.9	HEIGHT	11.85	12.5		0	1
23	5689	60	Azithromycin	1	106	3/9/2020	121.4	LENGTH	21.7	16.5		0	1
23	5690	0	Azithromycin	1	54	4/12/2015	102.4	HEIGHT	14.15	15		0	1
23	5690	12	Azithromycin	1	66	7/20/2016	109.9	HEIGHT		15		0	1
23	5690	24	Azithromycin	1	80	6/10/2017	114.6	HEIGHT	17.55	15.5		0	1
23	5690	36	Azithromycin	1	92	6/18/2018	118.8	HEIGHT	19.40909091	15.5		0	1
23	5690	48	Azithromycin	1	103	4/29/2019	124.5	HEIGHT	21.40909091	16		0	1
23	5691	12	Azithromycin	0	30	7/20/2016	106.3	HEIGHT		15.5		0	1
23	5691	36	Azithromycin	0	57	6/18/2018	115.7	HEIGHT	19.54545455	16		1	1
23	5691	48	Azithromycin	0	67	4/30/2019	121	HEIGHT	21.95	16		0	1
23	5692	0	Azithromycin	1	42	4/12/2015	94.4	HEIGHT	12.4	15.5		0	1
23	5692	24	Azithromycin	1	70	6/10/2017	108.6	HEIGHT	14.75	14.5		0	1
23	5692	48	Azithromycin	1	75	4/29/2019	116.3	HEIGHT	17.6	15.5		0	1
23	5693	0	Azithromycin	1	48	4/12/2015	102.8	HEIGHT	13.9	14	60	0	1
23	5693	36	Azithromycin	1	94	6/19/2018	120.3	HEIGHT	19.40909091	15.5	60	0	1
23	5694	0	Azithromycin	0	12	4/12/2015	77.2	HEIGHT	8.6	13		0	1
23	5695	0	Azithromycin	0	36	4/12/2015	78.8	HEIGHT	8.15	12		0	1
23	5695	12	Azithromycin	0	54	7/20/2016	86.7	HEIGHT		12.5		1	1
23	5695	24	Azithromycin	0	68	6/11/2017	91.8	HEIGHT	10.75	12		0	1
23	5695	36	Azithromycin	0	80	6/18/2018	98.1	HEIGHT	12.04545455	12		0	1
23	5695	48	Azithromycin	0	91	4/29/2019	101.9	HEIGHT	12.45	12		0	1
23	5695	60	Azithromycin	0	100	2/24/2020	106.2	HEIGHT	14.09090909	12.4		0	1

23	5701	0	Azithromycin	1	36	6/14/2015	85.1	HEIGHT	9.55	12		1	1
23	5704	0	Azithromycin	0	12	6/14/2015	69.2	LENGTH	7.3	12.5		0	1
23	5704	12	Azithromycin	0	26	7/20/2016	79.8	LENGTH		13.5		0	1
23	5704	36	Azithromycin	0	52	6/18/2018	90.1	LENGTH	11.04545455	13		0	1
23	5704	48	Azithromycin	0	63	4/29/2019	94.2	HEIGHT	11.77272727	13		0	1
23	5704	60	Azithromycin	0	72	3/9/2020	100	HEIGHT	12.7	13.4		0	1
23	5705	0	Azithromycin	0	12	4/12/2015	72.5	HEIGHT	8	13		0	1
23	5705	12	Azithromycin	0	26	7/20/2016	83.6	HEIGHT		13.5		0	1
23	5705	24	Azithromycin	0	40	6/10/2017	91.7	HEIGHT	11.5	13		0	1
23	5705	36	Azithromycin	0	52	6/18/2018	100	HEIGHT	13.36363636	13		1	1
23	5705	48	Azithromycin	0	63	4/29/2019	106.8	HEIGHT	15.55	14		0	1
23	5705	60	Azithromycin	0	72	2/24/2020	111.7	HEIGHT	16.90909091	14.5		0	1
23	5707	0	Azithromycin	0	24	5/10/2015	77.3	HEIGHT	8.4	13		1	1
23	5710	0	Azithromycin	0	36	6/14/2015	92.8	HEIGHT	14.6	15.5	6	0	1
23	5711	12	Azithromycin	0	1	7/20/2016	62.4	LENGTH		12.5		0	1
23	5711	24	Azithromycin	0	12	6/10/2017	74.6	HEIGHT	8.15	13.5		0	1
23	5711	36	Azithromycin	0	24	6/18/2018	80.4	HEIGHT	10.13636364	13.5		1	1
23	5711	60	Azithromycin	0	44	2/24/2020	91.9	HEIGHT	12.86363636	14		0	1
23	5713	12	Azithromycin	0	30	7/27/2016	102	HEIGHT		15		1	1
23	5716	24	Azithromycin	1	46	6/10/2017	91.2	HEIGHT	11.25	12.5		1	1
23	5717	24	Azithromycin	1	46	6/13/2017	90.5	HEIGHT	11.1	13		1	1
23	5719	12	Azithromycin	1	12	7/21/2016	72.2	HEIGHT		13.5		1	1
23	5719	24	Azithromycin	1	22	6/11/2017	79	HEIGHT	9.55	13.5		0	1
23	5719	36	Azithromycin	1	34	6/18/2018	85.2	HEIGHT	13.40909091	17		0	1
23	5719	48	Azithromycin	1	45	4/29/2019	92.2	HEIGHT	15.65	17.5		0	1
23	5719	60	Azithromycin	1	54	2/24/2020	98.4	HEIGHT	16	15.9		1	1
23	5721	0	Azithromycin	1	4	6/14/2015	60.7	LENGTH	4.85	11		1	1
23	5721	36	Azithromycin	1	40	6/18/2018	86.9	HEIGHT	10.54545455	13		0	1
23	5722	0	Azithromycin	0	48	5/10/2015	100.6	HEIGHT	14.85	14		0	1
23	5722	24	Azithromycin	0	68	6/10/2017	113.8	HEIGHT	18.5	14.5		0	1
23	5722	36	Azithromycin	0	80	6/18/2018	118.7	HEIGHT	21.54545455	14.5		0	1
23	5728	0	Azithromycin	0	36	4/12/2015	80.4	HEIGHT	8.7	12		1	1
23	5728	12	Azithromycin	0	50	7/20/2016	86.7	HEIGHT		13		0	1
23	5728	60	Azithromycin	0	96	3/9/2020	109.4	HEIGHT	17.15	15.2		0	1
23	5731	12	Azithromycin	1	38	7/21/2016	77.5	LENGTH		13		1	1
23	5731	24	Azithromycin	1	52	6/11/2017	93.2	HEIGHT	12.45	13.5		1	1
23	5732	12	Azithromycin	0	42	7/24/2016	99.3	HEIGHT		14.5		1	1
23	5733	0	Azithromycin	1	24	4/12/2015	85.2	HEIGHT	11.15	13		1	1
23	5733	12	Azithromycin	1	39	7/27/2016	92.5	HEIGHT		14		1	1
23	5733	36	Azithromycin	1	59	6/18/2018	105.4	HEIGHT	16.63636364	15		0	1

23	5733	60	Azithromycin	1	80	3/9/2020	115.2	HEIGHT	21.15	16.2		0	1
23	5734	12	Azithromycin	0	54	7/27/2016	106.8	HEIGHT		15.5		1	1
23	5737	0	Azithromycin	0	48	5/10/2015	93.9	HEIGHT	12.75	13		0	1
23	5737	60	Azithromycin	0	108	2/24/2020	105.8	HEIGHT	17.09090909	14.6		0	1
23	5739	0	Azithromycin	1	24	4/12/2015	85.1	HEIGHT	10.8	14.5		1	1
23	5741	0	Azithromycin	1	48	4/12/2015	90.7	HEIGHT	12.25	13.5		1	1
23	5741	24	Azithromycin	1	77	6/10/2017	105.8	HEIGHT	13.95	13		0	1
23	5741	36	Azithromycin	1	89	6/19/2018	112.8	HEIGHT	15.5	13		0	1
23	5741	48	Azithromycin	1	100	6/8/2019	118	HEIGHT	17.45454545	14		0	1
23	5741	60	Azithromycin	1	109	2/24/2020	122	HEIGHT	19.09090909	13.5		0	1
23	5743	12	Azithromycin	0	8	7/20/2016	63.9	LENGTH		10.5		0	1
23	5743	36	Azithromycin	0	29	6/18/2018	77.5	LENGTH	7.181818182	11		1	1
23	5743	48	Azithromycin	0	40	4/29/2019	82.5	HEIGHT	8.727272727	12		1	1
23	5746	12	Azithromycin	0	44	7/21/2016	88.4	HEIGHT		13		1	1
23	5747	0	Azithromycin	0	48	4/12/2015	94.2	HEIGHT	13.1	14.5		1	1
23	5750	12	Azithromycin	1	21	7/27/2016	78.8	HEIGHT		13.5		1	1
23	5750	60	Azithromycin	1	55	3/13/2020	98.9	HEIGHT	13.8	15		1	1
23	5751	12	Azithromycin	0	54	7/20/2016	87.2	HEIGHT		13		1	1
23	5753	0	Azithromycin	1	24	4/12/2015	70.9	LENGTH	7.55	12		0	1
23	5753	12	Azithromycin	1	38	7/21/2016	76.3	LENGTH		12		0	1
23	5753	36	Azithromycin	1	64	6/18/2018	117.4	HEIGHT	16.77272727	14		0	1
23	5753	48	Azithromycin	1	75	4/29/2019	92.3	HEIGHT	11.1	12		0	1
23	5754	0	Azithromycin	1	48	6/14/2015	101.2	HEIGHT	13.5	13.5		1	1
23	5756	0	Azithromycin	1	24	6/14/2015	83.5	LENGTH	9.8	12.5		0	1
23	5756	12	Azithromycin	1	42	7/21/2016	91.1	HEIGHT		13		0	1
23	5756	24	Azithromycin	1	58	6/10/2017	97.3	HEIGHT	11.4	12.5		0	1
23	5756	36	Azithromycin	1	70	6/18/2018	104.4	HEIGHT	13.86363636	12.5		0	1
23	5756	48	Azithromycin	1	81	4/29/2019	110.5	HEIGHT	15.9	14		0	1
23	5757	12	Azithromycin	1	1	7/20/2016	57.7	LENGTH		12		0	1
23	5757	24	Azithromycin	1	15	6/10/2017	70.2	HEIGHT	6.7	12.5		1	1
23	5757	36	Azithromycin	1	27	6/18/2018	78.3	LENGTH	8.318181818	12		0	1
23	5761	0	Azithromycin	0	6	4/12/2015	64.9	LENGTH	5.95	11.5	6	0	1
23	5762	0	Azithromycin	0	30	6/14/2015	78.5	HEIGHT	9.1	13		0	1
23	5762	24	Azithromycin	0	53	6/11/2017	94.2	HEIGHT	11.85	13.5		1	1
23	5762	36	Azithromycin	0	65	6/18/2018	97.6	HEIGHT	13.27272727	14		0	1
23	5762	48	Azithromycin	0	76	4/30/2019	103.7	HEIGHT	15.1	14.5		0	1
23	5764	24	Azithromycin	0	34	6/13/2017	81.6	HEIGHT	10.6	14.5		1	1
23	5767	0	Azithromycin	1	54	4/12/2015	110.4	HEIGHT	15.85	14		1	1
23	5767	36	Azithromycin	1	94	6/19/2018	126.2	HEIGHT	20.95454545	15		0	1
23	5767	48	Azithromycin	1	105	4/29/2019	130.1	HEIGHT	23.5	15.5		0	1

23	5767	60	Azithromycin	1	114	2/24/2020	131.7	HEIGHT	26.09090909	15.5		0	1
23	5769	0	Azithromycin	0	12	6/14/2015	71	LENGTH	7.55	12.5		1	1
23	5769	48	Azithromycin	0	63	4/29/2019	105.1	HEIGHT	16.35	15		0	1
23	5770	0	Azithromycin	1	24	4/12/2015	73.9	HEIGHT	6.8	11		0	1
23	5770	12	Azithromycin	1	30	7/20/2016	84.2	HEIGHT		13		0	1
23	5770	24	Azithromycin	1	44	6/11/2017	92.7	HEIGHT	11.3	13		0	1
23	5770	36	Azithromycin	1	56	6/18/2018	97.7	HEIGHT	12.31818182	12		0	1
23	5770	48	Azithromycin	1	67	4/29/2019	103.2	HEIGHT	14	12.5		0	1
23	5770	60	Azithromycin	1	76	2/24/2020	107.7	HEIGHT	14.59090909	12.2		0	1
23	5771	0	Azithromycin	0	54	6/14/2015	90.3	HEIGHT	11.6	14		0	1
23	5771	36	Azithromycin	0	106	6/18/2018	117.2	HEIGHT	18.86363636	14.5		0	1
23	5771	48	Azithromycin	0	117	4/29/2019	121.1	HEIGHT	21.81818182	15		0	1
23	5771	60	Azithromycin	0	127	2/24/2020	124.4	HEIGHT	23.13636364	15.2		0	1
23	5772	12	Azithromycin	1	45	7/21/2016	85.2	HEIGHT		15		1	1
23	5772	24	Azithromycin	1	56	6/11/2017	91.4	HEIGHT	13.3	16		1	1
23	5773	0	Azithromycin	1	12	4/12/2015	73.6	HEIGHT	8.3	13		0	1
23	5773	12	Azithromycin	1	15	7/20/2016	86	HEIGHT		15		0	1
23	5773	24	Azithromycin	1	25	6/11/2017	92.7	HEIGHT	13.3	14.5		1	1
23	5773	48	Azithromycin	1	48	4/30/2019	106.4	HEIGHT	16.7	16		1	1
23	5776	12	Azithromycin	1	5	7/20/2016	61.8	LENGTH		11.5		0	1
23	5776	24	Azithromycin	1	22	6/11/2017	70.3	LENGTH	7.25	13		1	1
23	5781	0	Azithromycin	1	8	4/12/2015	72.4	HEIGHT	7.1	13		1	1
23	5781	12	Azithromycin	1	23	7/20/2016	85.9	LENGTH		13		0	1
23	5781	48	Azithromycin	1	57	4/29/2019	99.7	HEIGHT	13.31818182	14		0	1
23	5783	12	Azithromycin	0	18	7/21/2016	76.1	LENGTH		13	54	1	1
23	5783	36	Azithromycin	0	34	6/18/2018	89.6	HEIGHT	12.31818182	13	54	1	1
23	5783	48	Azithromycin	0	45	6/9/2019	98.3	HEIGHT	14.5	13.4	54	1	1
23	5784	12	Azithromycin	0	54	7/24/2016	100.1	HEIGHT		13.5		1	1
23	5786	12	Azithromycin	1	1	7/20/2016	61.5	LENGTH		11.5		0	1
23	5786	36	Azithromycin	1	24	6/18/2018	81.5	LENGTH	9.5	13		0	1
23	5786	48	Azithromycin	1	34	4/29/2019	88	HEIGHT	10.2	12.5		1	1
23	5790	12	Azithromycin	0	7	7/20/2016	66.6	LENGTH		11.5		0	1
23	5790	48	Azithromycin	0	57	6/9/2019	84.9	HEIGHT	10.45454545	13.3		1	1
23	5790	60	Azithromycin	0	57	2/24/2020	89.9	HEIGHT	11.22727273	12.8		1	1
23	5791	12	Azithromycin	1	11	7/20/2016	69.6	LENGTH		12.5		0	1
23	5791	36	Azithromycin	1	29	6/18/2018	88.1	HEIGHT	11.36363636	14		0	1
23	5791	48	Azithromycin	1	40	4/29/2019	94	HEIGHT	11.6	12.5		1	1
23	5791	60	Azithromycin	1	49	2/24/2020	101.4	HEIGHT	14	13.5		0	1
23	5793	24	Azithromycin	0	5	6/10/2017	66	LENGTH	6.35	12.5		1	1
23	5793	36	Azithromycin	0	15	6/19/2018	77.7	HEIGHT	8.818181818	13		0	1

23	5793	60	Azithromycin	0	35	2/24/2020	88.4	HEIGHT	12.27272727	13.3		0	1
23	5795	0	Azithromycin	1	12	4/12/2015	74.1	HEIGHT	8	13	30	1	1
23	5795	12	Azithromycin	1	27	7/20/2016	80.7	HEIGHT		12.5	30	0	1
23	5795	24	Azithromycin	1	41	6/10/2017	86.6	HEIGHT	10.3	14	30	0	1
23	5800	24	Azithromycin	0	6	6/10/2017	62	LENGTH	5.35	11.5		1	1
23	5800	36	Azithromycin	0	17	6/18/2018	72.7	LENGTH	6.545454545	11		0	1
23	5800	48	Azithromycin	0	28	4/29/2019	79.7	LENGTH	8.181818182	12.5		0	1
23	5801	0	Azithromycin	1	24	4/12/2015	77.5	HEIGHT	8.65	13.5		0	1
23	5801	48	Azithromycin	1	75	4/30/2019	96.1	HEIGHT	13.5	14		0	1
23	5801	60	Azithromycin	1	84	3/9/2020	101.2	HEIGHT	14.8	14.8		0	1
23	5802	12	Azithromycin	1	38	7/24/2016	86.2	HEIGHT		14		1	1
23	5805	24	Azithromycin	1	52	6/13/2017	106.3	HEIGHT	15.6	14.5		1	1
23	5806	0	Azithromycin	0	24	5/10/2015	84.7	HEIGHT	10.55	13.5		1	1
23	5808	12	Azithromycin	0	5	7/20/2016	66.5	LENGTH		14		0	1
23	5808	24	Azithromycin	0	17	6/10/2017	74.7	HEIGHT	8.9	14		0	1
23	5808	36	Azithromycin	0	29	6/18/2018	83.2	LENGTH	10.54545455	15		0	1
23	5809	0	Azithromycin	0	54	4/12/2015	76.7	HEIGHT	8.35	12.5	24	0	1
23	5810	0	Azithromycin	1	36	4/12/2015	85.4	HEIGHT	9.8	12.5		1	1
23	5810	12	Azithromycin	1	39	7/20/2016	92.7	HEIGHT		13.5		0	1
23	5810	24	Azithromycin	1	49	6/11/2017	99.6	HEIGHT	13.1	13		0	1
23	5810	48	Azithromycin	1	72	4/30/2019	108.8	HEIGHT	15.85	14		0	1
23	5813	0	Azithromycin	1	54	5/10/2015	100.6	HEIGHT	12.6	14		0	1
23	5816	0	Azithromycin	1	24	5/10/2015	77.4	HEIGHT	8.4	12.5		0	1
23	5816	12	Azithromycin	1	42	7/20/2016	85.1	HEIGHT		14.5		0	1
23	5816	24	Azithromycin	1	58	6/10/2017	92	HEIGHT	13.1	14.5		0	1
23	5816	36	Azithromycin	1	70	6/18/2018	97.6	HEIGHT	14.5	15		0	1
23	5820	24	Azithromycin	1	40	6/10/2017	93.9	HEIGHT	11.75	14		1	1
23	5822	0	Azithromycin	1	12	4/12/2015	71.8	HEIGHT	7.6	12.5		1	1
23	5823	0	Azithromycin	1	24	4/12/2015	80.5	HEIGHT	11.7	15.5		1	1
23	5823	60	Azithromycin	1	84	3/9/2020	115.8	HEIGHT	21.45	16.8		0	1
23	5824	0	Azithromycin	0	42	4/12/2015	91.4	HEIGHT	10.6	13		0	1
23	5824	12	Azithromycin	0	61	7/20/2016	96.3	HEIGHT		12		0	1
23	5827	0	Azithromycin	0	4	4/12/2015	68.1	LENGTH	7.1	13		0	1
23	5827	12	Azithromycin	0	14	7/24/2016	76.1	LENGTH		13.5		1	1
23	5827	24	Azithromycin	0	28	6/11/2017	84.3	HEIGHT	9.85	13		0	1
23	5827	36	Azithromycin	0	40	6/18/2018	92.2	HEIGHT	12.45454545	14		1	1
23	5827	48	Azithromycin	0	51	4/30/2019	96.4	HEIGHT	12.85	14		0	1
23	5828	0	Azithromycin	1	5	6/14/2015	66.9	LENGTH	7.2	13		1	1
23	5828	12	Azithromycin	1	18	7/21/2016	77.3	HEIGHT		13		0	1
23	5828	36	Azithromycin	1	41	6/18/2018	92.3	HEIGHT	12.40909091	13		1	1

23	5828	48	Azithromycin	1	52	6/8/2019	98.2	HEIGHT	13.63636364	14		1	1
23	5831	0	Azithromycin	1	12	6/14/2015	69.5	LENGTH	7.8	14		0	1
23	5832	0	Azithromycin	1	24	4/12/2015	76.1	HEIGHT	8.3	13		0	1
23	5832	12	Azithromycin	1	39	7/20/2016	84	HEIGHT		14		0	1
23	5832	24	Azithromycin	1	53	6/10/2017	92.5	HEIGHT	11.4	13		1	1
23	5832	36	Azithromycin	1	65	6/18/2018	99.6	HEIGHT	12.63636364	13.5		0	1
23	5832	48	Azithromycin	1	76	6/8/2019	104.1	HEIGHT	14.31818182	14.2		0	1
23	5833	0	Azithromycin	1	48	4/12/2015	85.2	HEIGHT	10.7	14		0	1
23	5833	12	Azithromycin	1	62	7/20/2016	93.2	HEIGHT		13.5		0	1
23	5835	24	Azithromycin	0	58	6/10/2017	91.4	HEIGHT	11.2	12.5		1	1
23	5837	24	Azithromycin	1	7	6/11/2017	65.5	LENGTH	5.6	11		1	1
23	5837	36	Azithromycin	1	17	6/18/2018	76.4	LENGTH	8.181818182	12		0	1
23	5837	48	Azithromycin	1	28	4/29/2019	82.8	LENGTH	9.3	12		0	1
23	5837	60	Azithromycin	1	37	2/24/2020	89.4	LENGTH	10.59090909	11.3		0	1
23	5841	12	Azithromycin	1	38	7/21/2016	92.7	HEIGHT		14.5		1	1
23	5842	24	Azithromycin	0	67	6/10/2017	98.8	HEIGHT	13.9	15		1	1
23	5844	12	Azithromycin	1	72	7/21/2016	104	HEIGHT		14.5		0	1
23	5844	24	Azithromycin	1	82	6/10/2017	111	HEIGHT	15.75	14.5		0	1
23	5844	36	Azithromycin	1	94	6/19/2018	117.1	HEIGHT	17.72727273	14		0	1
23	5844	60	Azithromycin	1	115	2/24/2020	125	HEIGHT	22.40909091	15.7		0	1
23	5846	12	Azithromycin	1	6	7/20/2016	66.8	LENGTH		13.5		0	1
23	5846	36	Azithromycin	1	29	6/18/2018	83	HEIGHT	9.545454545	13		0	1
23	5849	0	Azithromycin	0	48	4/12/2015	96.4	HEIGHT	14.05	16		1	1
23	5849	12	Azithromycin	0	63	7/20/2016	101.3	HEIGHT		14		0	1
23	5849	36	Azithromycin	0	89	6/18/2018	109.5	HEIGHT	16.54545455	14.5		0	1
23	5850	12	Azithromycin	1	38	7/27/2016	77.7	HEIGHT		11.5		1	1
23	5850	24	Azithromycin	1	52	6/10/2017	83.8	HEIGHT	9.4	11		1	1
23	5852	0	Azithromycin	0	24	4/12/2015	83.4	HEIGHT	10.1	13.5		1	1
23	5852	48	Azithromycin	0	75	6/8/2019	112.1	HEIGHT	18.86363636	15.3		0	1
23	5853	12	Azithromycin	0	30	7/20/2016	87.3	HEIGHT		14		1	1
23	5853	36	Azithromycin	0	56	6/18/2018	97.9	HEIGHT	13.81818182	13		1	1
23	5854	12	Azithromycin	0	42	7/27/2016	95.1	HEIGHT		14.5		1	1
23	5855	0	Azithromycin	0	48	4/12/2015	90.4	HEIGHT	13.65	15.5		1	1
23	5855	36	Azithromycin	0	88	6/18/2018	110.5	HEIGHT	18.40909091	15		0	1
23	5856	24	Azithromycin	1	50	6/13/2017	90.1	HEIGHT	12.3	13.5		1	1
23	5863	0	Azithromycin	1	8	4/12/2015	67.8	LENGTH	6.65	12	54	1	1
23	5863	12	Azithromycin	1	20	7/20/2016	78.3	HEIGHT		12.5	54	0	1
23	5863	24	Azithromycin	1	34	6/10/2017	85.2	HEIGHT	9.6	12	54	0	1
23	5863	36	Azithromycin	1	47	6/18/2018	92.3	HEIGHT	11.31818182	12.5	54	1	1
23	5863	48	Azithromycin	1	58	4/29/2019	97.4	HEIGHT	12.75	12.5	54	0	1

23	5864	24	Azithromycin	1	63	6/13/2017	114.7	HEIGHT	15.4	13		1	1
23	5865	0	Azithromycin	0	48	6/14/2015	93	HEIGHT	13.05	14.5		0	1
23	5867	0	Azithromycin	1	60	4/12/2015	103.1	HEIGHT	13.7	14.5		1	1
23	5867	48	Azithromycin	1	111	4/29/2019	126.5	HEIGHT	20.5	14.5		0	1
23	5868	0	Azithromycin	1	36	4/12/2015	95	HEIGHT	11.65	13.5		0	1
23	5868	48	Azithromycin	1	87	6/8/2019	120.3	LENGTH	18.81818182	14.9		0	1
23	5869	12	Azithromycin	0	12	7/20/2016	73.8	HEIGHT		13.5		1	1
23	5869	48	Azithromycin	0	45	4/30/2019	95.7	HEIGHT	13.15	13.5		0	1
23	5871	12	Azithromycin	0	42	7/20/2016	106.5	HEIGHT		14.5		1	1
23	5874	0	Azithromycin	1	36	5/10/2015	83.6	HEIGHT	10.7	14.5		0	1
23	5874	12	Azithromycin	1	51	7/21/2016	93.8	HEIGHT		14.5		0	1
23	5876	0	Azithromycin	1	48	4/12/2015	97.5	HEIGHT	15.25	16		0	1
23	5876	12	Azithromycin	1	54	7/21/2016	104.5	HEIGHT		15		0	1
23	5876	36	Azithromycin	1	82	6/18/2018	115.5	HEIGHT	20.36363636	16		0	1
23	5877	12	Azithromycin	1	5	7/20/2016	59.1	LENGTH		10.5		0	1
23	5877	24	Azithromycin	1	12	6/10/2017	73	HEIGHT	6.85	11.5		0	1
23	5877	36	Azithromycin	1	24	6/18/2018	82.5	HEIGHT	8.681818182	12		0	1
23	5882	0	Azithromycin	0	24	4/12/2015	78.1	HEIGHT	8.3	12.5	18	0	1
23	5882	12	Azithromycin	0	39	7/20/2016	83.4	HEIGHT		12.5	18	1	1
23	5883	12	Azithromycin	1	54	7/20/2016	95	HEIGHT		13		1	1
23	5885	0	Azithromycin	0	5	4/12/2015	66.6	LENGTH	6.5	12.5		0	1
23	5885	12	Azithromycin	0	30	7/21/2016	79.9	HEIGHT		14		0	1
23	5886	24	Azithromycin	0	44	6/10/2017	89.7	HEIGHT	11.55	12.5		1	1
23	5887	12	Azithromycin	1	7	7/20/2016	63.6	LENGTH		10.5		1	1
23	5887	24	Azithromycin	1	17	6/10/2017	72.5	HEIGHT	7.3	12		1	1
23	5887	36	Azithromycin	1	29	6/18/2018	82.3	LENGTH	9.772727273	13.5		0	1
23	5887	48	Azithromycin	1	40	4/29/2019	87.3	HEIGHT	11.25	14		0	1
23	5887	60	Azithromycin	1	49	2/24/2020	93.6	HEIGHT	11.95454545	13.5		1	1
23	5890	24	Azithromycin	1	22	6/11/2017	81.5	HEIGHT	11.25	16.5		1	1
23	5892	12	Azithromycin	0	18	7/24/2016	89.4	HEIGHT		14.5		1	1
23	5892	24	Azithromycin	0	34	6/13/2017	97	HEIGHT	13.25	13		1	1
23	5893	0	Azithromycin	0	24	5/10/2015	82.1	HEIGHT	10.35	14.5		0	1
23	5893	24	Azithromycin	0	46	6/10/2017	100.2	HEIGHT	14.1	14.5		1	1
23	5893	48	Azithromycin	0	69	4/29/2019	112.1	HEIGHT	17.5	14.5		0	1
23	5894	0	Azithromycin	0	48	5/10/2015	89.8	HEIGHT	11.35	13.5		1	1
23	5894	12	Azithromycin	1	30	7/20/2016	96.7	HEIGHT		13.5		0	1
23	5894	36	Azithromycin	0	57	6/18/2018	108.8	HEIGHT	15.63636364	14		0	1
23	5894	48	Azithromycin	0	67	4/30/2019	113.2	HEIGHT	18.2	15		0	1
23	5895	0	Azithromycin	0	36	5/10/2015	76.8	HEIGHT	8.35	13		0	1
23	5895	36	Azithromycin	0	56	6/18/2018	92.9	HEIGHT	11.18181818	13		0	1

23	5895	48	Azithromycin	0	56	4/30/2019	97.8	HEIGHT	12.4	13		1	1
23	5896	12	Azithromycin	1	42	7/24/2016	107.6	HEIGHT		14		1	1
23	5897	0	Azithromycin	1	24	4/12/2015	87.6	HEIGHT	11.85	13.5		1	1
23	5897	12	Azithromycin	1	41	7/20/2016	99.6	HEIGHT		16		0	1
23	5897	24	Azithromycin	1	52	6/11/2017	106.9	HEIGHT	16.7	15		1	1
23	5897	36	Azithromycin	1	64	6/19/2018	114.2	HEIGHT	18.09090909	15		0	1
23	5897	48	Azithromycin	1	75	4/30/2019	117.8	HEIGHT	20.05	15.5		0	1
23	5898	0	Azithromycin	1	12	5/10/2015	77.3	LENGTH	10.3	15.5		1	1
23	5898	12	Azithromycin	1	26	7/21/2016	88.7	HEIGHT		15.5		0	1
23	5898	36	Azithromycin	1	58	6/18/2018	102.5	HEIGHT	17.36363636	16		0	1
23	5899	0	Azithromycin	1	10	5/10/2015	66.8	LENGTH	6.8	13.5	30	1	1
23	5900	0	Azithromycin	0	36	4/12/2015	92.7	HEIGHT	13.5	15.5		0	1
23	5900	24	Azithromycin	0	64	6/10/2017	104.7	HEIGHT	15.95	15		0	1
23	5900	36	Azithromycin	0	76	6/18/2018	109.2	HEIGHT	17.54545455	15		0	1
23	5900	60	Azithromycin	0	96	2/24/2020	116.9	HEIGHT	20.36363636	15.3		0	1
23	5901	0	Azithromycin	0	36	4/12/2015	92.1	HEIGHT	10.85	13		1	1
23	8042	60	Azithromycin	0	15	3/9/2020	74.1	LENGTH	7.4	12.5		1	1
23	8093	60	Azithromycin	0	8	3/13/2020	69.6	LENGTH	7.2	13.5		1	1
23	8108	48	Azithromycin	1	48	4/30/2019	91.6	HEIGHT	13.05	14.5		1	1
23	8115	36	Azithromycin	0	12	6/19/2018	72.8	LENGTH	8.681818182	12.5		1	1
23	8115	48	Azithromycin	0	23	6/8/2019	80.6	HEIGHT	10.59090909	14		0	1
23	8115	60	Azithromycin	0	32	3/13/2020	85.4	HEIGHT	11.55	14.9		0	1
23	8146	60	Azithromycin	1	33	3/9/2020	90.3	HEIGHT	11.55	14.1		1	1
23	8148	48	Azithromycin	0	36	6/8/2019	86.1	LENGTH	10.63636364	14.7		1	1
23	8167	36	Azithromycin	0	56	6/18/2018	91	HEIGHT	12	13.5		1	1
23	8182	36	Azithromycin	0	29	6/19/2018	80.5	LENGTH	9.45	12.5		1	1
23	8192	60	Azithromycin	1	51	3/9/2020	87.9	HEIGHT	13.85	14		1	1
23	8194	48	Azithromycin	0	54	6/9/2019	105.1	HEIGHT	15.36363636	15		1	1
23	8201	36	Azithromycin	1	23	6/18/2018	77	LENGTH	8.5	13		1	1
23	8206	60	Azithromycin	1	44	3/13/2020	90.9	HEIGHT	12.95	15		1	1
23	8258	48	Azithromycin	1	55	4/29/2019	105.3	HEIGHT	15.15	13.5		1	1
23	8270	60	Azithromycin	0	40	3/13/2020	92.5	HEIGHT	12.9	15		1	1
23	8279	36	Azithromycin	1	23	6/19/2018	100	HEIGHT	13.35	13.5		1	1
23	8282	60	Azithromycin	0	6	3/9/2020	59.1	LENGTH	5.7	13		1	1
23	8284	48	Azithromycin	1	45	4/29/2019	86.7	HEIGHT	10.25	12.5		1	1
23	8306	48	Azithromycin	1	18	4/29/2019	79.5	HEIGHT	9.909090909	13		1	1
23	8370	36	Azithromycin	0	44	6/18/2018	83.3	HEIGHT	9.136363636	11.5		1	1
23	8370	60	Azithromycin	0	55	2/24/2020	93.2	HEIGHT	12.18181818	13		1	1
23	8375	60	Azithromycin	0	25	2/24/2020	90.1	HEIGHT	13	14.5		1	1
23	8380	48	Azithromycin	0	12	4/30/2019	66.5	LENGTH	5.1	11		1	1

23	8388	60	Azithromycin	1	47	3/9/2020	102	HEIGHT	15.4	15		1	1
23	8396	48	Azithromycin	0	55	4/29/2019	107.9	HEIGHT	15.55	14.5		1	1
23	8400	60	Azithromycin	0	39	3/9/2020	92.4	HEIGHT	12.25	14		1	1
23	8444	36	Azithromycin	0	15	6/18/2018	75.1	LENGTH	8.5	13		1	1
23	8444	60	Azithromycin	0	35	2/24/2020	88.8	HEIGHT	11.90909091	13.7		1	1
23	8476	60	Azithromycin	0	24	3/13/2020	77.2	LENGTH	9.75	16		1	1
23	8497	60	Azithromycin	1	33	3/9/2020	77.9	HEIGHT	9.25	13.5		1	1
23	8507	60	Azithromycin	0	19	2/24/2020	96.6	HEIGHT	10.45454545	14		1	1
23	8528	48	Azithromycin	1	18	4/29/2019	67	LENGTH	5.954545455	11		1	1
23	8550	36	Azithromycin	1	45	6/19/2018	93	HEIGHT	12.5	14		1	1
23	8552	36	Azithromycin	0	26	6/19/2018	85.9	LENGTH	11.40909091	14.5		1	1
23	8557	60	Azithromycin	0	57	3/9/2020	102.4	LENGTH	16.15	14.6		1	1
23	8559	60	Azithromycin	0	21	3/9/2020	83.2	HEIGHT	9.4	13		1	1
23	8563	36	Azithromycin	1	25	6/18/2018	81.5	HEIGHT	9	12.5		1	1
23	8582	48	Azithromycin	1	16	6/8/2019	75.8	LENGTH	8.136363636	13.4		1	1
23	8610	48	Azithromycin	0	14	4/29/2019	76.6	LENGTH	9.25	13		1	1
23	8663	48	Azithromycin	1	34	4/30/2019	82.2	HEIGHT	10.85	14		1	1
23	8703	36	Azithromycin	0	53	6/19/2018	98.9	HEIGHT	14.55	14.5		1	1
23	8717	36	Azithromycin	1	21	6/18/2018	75.1	LENGTH	7.272727273	12	54	1	1
23	8805	48	Azithromycin	1	14	4/29/2019	76.1	HEIGHT	8.5	13		1	1
23	8805	60	Azithromycin	1	23	3/13/2020	82.7	LENGTH	10.65	14.3		1	1
23	8862	48	Azithromycin	1	21	4/29/2019	81.3	LENGTH	7.65	11.5		1	1
23	8877	36	Azithromycin	1	5	6/18/2018	62.5	LENGTH	5.681818182	12		1	1
23	8877	60	Azithromycin	1	25	2/24/2020	76.5	HEIGHT	9.772727273	13		0	1
23	8889	60	Azithromycin	0	55	3/13/2020	96.4	HEIGHT	13.15	15		1	1
23	8898	60	Azithromycin	1	3	3/13/2020	55.3	LENGTH	4.6	11.4		1	1
23	8905	60	Azithromycin	0	27	2/24/2020	87.7	HEIGHT	12.63636364	15.1		1	1
23	8911	48	Azithromycin	0	8	4/30/2019	68.8	LENGTH	6.9	12		1	1
23	8918	60	Azithromycin	0	44	2/24/2020	93.7	HEIGHT	16.36363636	16.5		1	1
23	8941	60	Azithromycin	1	57	2/24/2020	107.8	HEIGHT	15.5	14.3		1	1
23	8975	36	Azithromycin	1	23	6/18/2018	83.5	HEIGHT	9.227272727	12		1	1
23	8988	60	Azithromycin	0	7	3/13/2020	69.3	LENGTH	6.95	13.5		1	1
23	9002	48	Azithromycin	0	32	4/29/2019	78.7	LENGTH	11.25	13.5		1	1
23	9038	36	Azithromycin	0	57	6/18/2018	106.9	HEIGHT	14	13		1	1
23	9051	36	Azithromycin	0	9	6/18/2018	69.8	LENGTH	7.818181818	12.5		1	1
23	9051	48	Azithromycin	0	20	6/8/2019	78.2	LENGTH	9.772727273	14		1	1
23	9051	60	Azithromycin	0	29	2/24/2020	82.3	HEIGHT	11.22727273	13.5		0	1
23	9052	60	Azithromycin	1	27	3/13/2020	81.3	HEIGHT	10.2	14.5		1	1
23	9075	48	Azithromycin	0	4	4/30/2019	52.4	LENGTH	3.85	11.5		1	1
23	9082	60	Azithromycin	1	35	2/24/2020	87.5	HEIGHT	11.31818182	13.3		1	1

23	9087	36	Azithromycin	0	2	6/18/2018	58.2	LENGTH	4.818181818	11.5		1	1
23	9087	48	Azithromycin	0	12	4/30/2019	73.1	LENGTH	7.6	13		0	1
23	9104	48	Azithromycin	0	29	4/29/2019	78.8	HEIGHT	9.727272727	13		1	1
23	9124	36	Azithromycin	1	35	6/18/2018	86	HEIGHT	10.31818182	13	42	1	1
23	9150	60	Azithromycin	0	27	3/9/2020	78	HEIGHT	8.6	12.3		1	1
23	9186	48	Azithromycin	1	18	4/30/2019	79.2	HEIGHT	9.45	14		1	1
23	9191	60	Azithromycin	1	39	3/9/2020	86.7	HEIGHT	10.55	13.5		1	1
23	9258	36	Azithromycin	0	15	6/18/2018	71.7	LENGTH	7.545454545	13		1	1
23	9264	36	Azithromycin	1	13	6/19/2018	70.8	LENGTH	7	12		1	1
23	9264	48	Azithromycin	1	24	4/29/2019	80.1	LENGTH	8.1	12.5		1	1
23	9272	60	Azithromycin	0	27	2/24/2020	79.6	LENGTH	9.954545455	12.7		1	1
23	9276	48	Azithromycin	0	18	6/8/2019	70.7	LENGTH	6.545454545	13		1	1
23	9276	60	Azithromycin	0	27	3/9/2020	73.5	HEIGHT	8.25	12.8		1	1
23	9284	36	Azithromycin	0	12	6/18/2018	63.4	LENGTH	5	10.5		1	1
23	9284	48	Azithromycin	0	23	4/29/2019	72.1	LENGTH	8.318181818	13.5		0	1
23	9321	60	Azithromycin	1	39	3/9/2020	86.1	HEIGHT	11.6	14.5		1	1
23	9322	36	Azithromycin	0	34	6/18/2018	91.8	LENGTH	12.5	15.5		1	1
23	9322	60	Azithromycin	0	55	2/24/2020	105.7	HEIGHT	15.59090909	14.5		1	1
23	9324	48	Azithromycin	1	23	6/9/2019	91.1	HEIGHT	12.36363636	15.5		1	1
23	9394	36	Azithromycin	0	2	6/19/2018	57.1	LENGTH	5.25	12		1	1
23	9394	48	Azithromycin	0	12	6/8/2019	74.8	LENGTH	8.181818182	14.5		0	1
23	9394	60	Azithromycin	0	21	2/24/2020	83.6	LENGTH	10.22727273	13.7		0	1
23	9430	60	Azithromycin	0	12	3/9/2020	73.5	HEIGHT	8.7	13.5		1	1
23	9516	60	Azithromycin	1	30	3/9/2020	80.1	HEIGHT	8.85	12.5		1	1
23	9529	60	Azithromycin	0	35	3/13/2020	86.2	LENGTH	11.35	14.8		1	1
23	9534	36	Azithromycin	1	57	6/18/2018	104.6	HEIGHT	14.59090909	14.5		1	1
23	9557	60	Azithromycin	1	51	2/24/2020	96.2	HEIGHT	12.22727273	14		1	1
23	9581	48	Azithromycin	1	45	4/29/2019	87.9	HEIGHT	11.95	14		1	1
24	5915	0	Azithromycin	1	36	4/4/2015	103.2	HEIGHT	14.45	14.5		0	0
24	5915	12	Azithromycin	1	52	6/27/2016	109.5	HEIGHT	17.05	15.5		0	0
24	5915	24	Azithromycin	1	62	4/4/2017	114.8	HEIGHT	17.85	15.5		0	0
24	5915	36	Azithromycin	1	76	5/23/2018	121.2	HEIGHT	20.2	16		0	0
24	5915	48	Azithromycin	1	86	4/28/2019	123.3	HEIGHT	21.4	16		0	0
24	5915	60	Azithromycin	1	96	2/19/2020	128.6	HEIGHT	23.5	17		0	0
24	5916	12	Azithromycin	1	10	6/27/2016	69.3	HEIGHT	6.45	12		0	0
24	5916	24	Azithromycin	1	22	4/4/2017	71.8	LENGTH	7.1	11.5		0	0
24	5916	36	Azithromycin	1	35	5/23/2018	78.9	LENGTH	10.55	13.5		0	0
24	5916	48	Azithromycin	1	46	4/28/2019	90.2	HEIGHT	14.55	15		1	0
24	5916	60	Azithromycin	1	56	2/19/2020	101.6	HEIGHT	16.65	14.4		1	0
24	5918	0	Azithromycin	1	48	4/4/2015	98	HEIGHT	13.85	15		0	0

24	5918	36	Azithromycin	1	104	5/23/2018	118.8	HEIGHT	20.05	16.5	0	0
24	5918	60	Azithromycin	1	125	2/19/2020	128	HEIGHT	23.3	17.5	0	0
24	5919	0	Azithromycin	1	48	4/4/2015	97.5	HEIGHT	12.2	13.5	0	0
24	5919	12	Azithromycin	1	54	6/20/2016	106.9	HEIGHT	15.4	15	1	0
24	5919	24	Azithromycin	1	66	4/4/2017	109.9	HEIGHT	16.25	15.5	0	0
24	5919	36	Azithromycin	1	80	5/23/2018	116.5	HEIGHT	18.5	15.5	0	0
24	5919	48	Azithromycin	1	91	4/28/2019	120.6	HEIGHT	18.8	14.5	0	0
24	5920	0	Azithromycin	1	48	4/4/2015	117.2	HEIGHT	19.45	16	0	0
24	5920	36	Azithromycin	1	104	5/23/2018	134	HEIGHT	26.5	17.5	0	0
24	5920	48	Azithromycin	1	115	4/28/2019	138.3	HEIGHT	29.35	18	0	0
24	5921	12	Azithromycin	1	4	6/20/2016	63.3	LENGTH	6.25	14	1	0
24	5921	24	Azithromycin	1	16	4/4/2017	69.4	HEIGHT	7.1	14	1	0
24	5921	48	Azithromycin	1	41	4/28/2019	86	HEIGHT	11.4	15	1	0
24	5922	0	Azithromycin	1	54	4/4/2015	118.4	HEIGHT	20.45	17	0	0
24	5923	0	Azithromycin	1	36	4/4/2015	80.7	HEIGHT	10.4	14	0	0
24	5923	48	Azithromycin	1	88	4/28/2019	107.5	HEIGHT	15.95	14	0	0
24	5924	0	Azithromycin	1	36	4/4/2015	91.6	HEIGHT	11.9	14	1	0
24	5924	12	Azithromycin	1	53	6/27/2016	99.3	HEIGHT	13.8	14.5	0	0
24	5924	24	Azithromycin	1	66	4/4/2017	105.2	HEIGHT	14.9	14.5	0	0
24	5924	48	Azithromycin	1	90	4/28/2019	118.1	HEIGHT	18.9	14.5	0	0
24	5925	0	Azithromycin	0	36	4/4/2015	96.7	HEIGHT	10.55	12.5	0	0
24	5925	12	Azithromycin	0	53	6/20/2016	105.6	HEIGHT	12.4	13	1	0
24	5925	24	Azithromycin	0	64	4/4/2017	110.5	HEIGHT	13.8	13	0	0
24	5925	36	Azithromycin	0	77	5/23/2018	116.4	HEIGHT	16	14	0	0
24	5925	48	Azithromycin	0	88	4/28/2019	121.6	HEIGHT	17.35	15.5	0	0
24	5925	60	Azithromycin	0	98	2/19/2020	125.7	HEIGHT	19.95	14.5	0	0
24	5926	24	Azithromycin	1	17	4/4/2017	74.7	HEIGHT	7.9	12.5	1	0
24	5926	36	Azithromycin	1	31	5/23/2018	81.2	LENGTH	9.45	13.5	1	0
24	5927	0	Azithromycin	1	36	4/4/2015	86.2	HEIGHT	10.9	14	1	0
24	5927	12	Azithromycin	1	54	6/20/2016	92.5	HEIGHT	12.1	14	1	0
24	5927	24	Azithromycin	1	64	4/4/2017	98.9	HEIGHT	13.6	14.5	0	0
24	5927	36	Azithromycin	1	78	5/23/2018	104.3	HEIGHT	15.55	14.5	0	0
24	5927	48	Azithromycin	1	89	4/28/2019	109.3	HEIGHT	16.7	14.5	0	0
24	5927	60	Azithromycin	1	98	2/19/2020	113.8	HEIGHT	19.15	15.5	0	0
24	5928	0	Azithromycin	1	36	4/4/2015	93.7	HEIGHT	12.8	15	0	0
24	5928	12	Azithromycin	1	42	6/27/2016	101.6	HEIGHT	15.15	16	0	0
24	5928	24	Azithromycin	1	55	4/4/2017	108.1	HEIGHT	15.9	15.5	0	0
24	5928	36	Azithromycin	1	68	5/23/2018	114.2	HEIGHT	18.55	16.5	0	0
24	5928	48	Azithromycin	1	79	4/28/2019	119.6	HEIGHT	20.7	17	0	0
24	5928	60	Azithromycin	1	89	2/19/2020	124.9	HEIGHT	23.8	17	0	0

24	5929	0	Azithromycin	0	36	4/4/2015	108.8	HEIGHT	17.45	15		1	0
24	5929	12	Azithromycin	0	51	6/27/2016	113.1	HEIGHT	18.2	16		0	0
24	5929	24	Azithromycin	0	64	4/4/2017	117.6	HEIGHT	19.1	15.5		0	0
24	5929	48	Azithromycin	0	88	4/28/2019	126.3	HEIGHT	23	16		0	0
24	5929	60	Azithromycin	0	98	2/19/2020	131.1	HEIGHT	26	16.5		0	0
24	5934	0	Azithromycin	1	36	4/4/2015	94.9	HEIGHT	13.5	16		0	0
24	5934	12	Azithromycin	1	52	6/20/2016	102	HEIGHT	15.9	16		1	0
24	5934	24	Azithromycin	1	62	4/4/2017	108.9	HEIGHT	16.7	15.5		0	0
24	5934	48	Azithromycin	1	86	4/28/2019	120.3	HEIGHT	20.6	16.5		0	0
24	5935	12	Azithromycin	0	57	6/20/2016	110.8	HEIGHT	18.35	15		1	0
24	5936	0	Azithromycin	0	12	4/4/2015	73.8	HEIGHT	7.5	13		0	0
24	5936	12	Azithromycin	0	36	6/20/2016	82.4	HEIGHT	10.25	14.5		1	0
24	5936	36	Azithromycin	0	62	5/23/2018	98.8	HEIGHT	12.85	14		0	0
24	5936	48	Azithromycin	0	73	4/28/2019	102.6	HEIGHT	13.8	13.5		0	0
24	5936	60	Azithromycin	0	83	2/19/2020	108	HEIGHT	15.85	14		0	0
24	5937	0	Azithromycin	1	48	4/4/2015	103.3	HEIGHT	14.4	14.5		0	0
24	5937	24	Azithromycin	1	76	4/4/2017	115.7	HEIGHT	18.5	15		0	0
24	5937	36	Azithromycin	1	89	5/23/2018	120.2	HEIGHT	19.75	15		0	0
24	5937	48	Azithromycin	1	100	4/28/2019	125.4	HEIGHT	21.3	15		0	0
24	5940	0	Azithromycin	1	36	4/4/2015	95	HEIGHT	15.1	17	12	0	0
24	5942	12	Azithromycin	0	54	6/20/2016	92.5	HEIGHT	15.35	15	48	1	0
24	5943	0	Azithromycin	1	54	4/4/2015	110.7	HEIGHT	19.85	16.5		0	0
24	5943	24	Azithromycin	1	82	4/4/2017	119.9	HEIGHT	23.65	17.5		0	0
24	5943	36	Azithromycin	1	95	5/23/2018	124.3	HEIGHT	26.6	18.5		0	0
24	5943	48	Azithromycin	1	106	4/28/2019	128	HEIGHT	28.25	19		0	0
24	5943	60	Azithromycin	1	116	2/19/2020	132.3	HEIGHT	31.05	19		0	0
24	5944	24	Azithromycin	1	6	4/4/2017	64.3	LENGTH	6.9	14		1	0
24	5944	36	Azithromycin	1	15	5/23/2018	71.9	LENGTH	7.55	12		1	0
24	5947	12	Azithromycin	0	4	6/27/2016	66.3	LENGTH	6.85	15.5		0	0
24	5947	24	Azithromycin	0	16	4/4/2017	73.2	LENGTH	8.35	13.5		0	0
24	5947	36	Azithromycin	0	31	5/23/2018	83.5	HEIGHT	11	15		1	0
24	5947	48	Azithromycin	0	41	4/28/2019	90.2	HEIGHT	12.45	15		1	0
24	5947	60	Azithromycin	0	51	2/19/2020	97.6	HEIGHT	13.4	14		0	0
24	5949	0	Azithromycin	0	12	4/4/2015	78	HEIGHT	8.65	13.5		1	0
24	5949	12	Azithromycin	0	28	6/20/2016	88.8	HEIGHT	11.35	14.5		1	0
24	5949	24	Azithromycin	0	38	4/4/2017	94.9	HEIGHT	12.45	14		1	0
24	5950	0	Azithromycin	1	54	4/4/2015	102.7	HEIGHT	13.8	14.5		0	0
24	5950	36	Azithromycin	1	116	5/23/2018	118.1	HEIGHT	18.85	15.5		0	0
24	5950	48	Azithromycin	1	127	4/28/2019	121.5	HEIGHT	20.05	15.5		0	0
24	5951	0	Azithromycin	0	18	4/4/2015	79.5	HEIGHT	10.15	14.5		1	0

24	5951	12	Azithromycin	0	32	6/20/2016	86.4	HEIGHT	11.9	15		1	0
24	5951	24	Azithromycin	0	44	4/4/2017	93.4	HEIGHT	13.45	15		1	0
24	5951	36	Azithromycin	0	64	5/23/2018	100.5	HEIGHT	15.6	15		0	0
24	5951	60	Azithromycin	0	84	2/19/2020	109.5	HEIGHT	18.8	15.3		0	0
24	5953	0	Azithromycin	1	36	4/4/2015	92.5	HEIGHT	12.8	14		1	0
24	5953	24	Azithromycin	1	68	4/4/2017	110.2	HEIGHT	17.4	15		0	0
24	5953	36	Azithromycin	1	82	5/23/2018	118.2	HEIGHT	19.2	15		0	0
24	5959	24	Azithromycin	1	21	4/4/2017	80.5	HEIGHT	12.3	18		1	0
24	5959	48	Azithromycin	1	46	4/28/2019	97.9	HEIGHT	16.85	17		1	0
24	5959	60	Azithromycin	1	55	2/19/2020	104.9	HEIGHT	18.85	16		0	0
24	5961	0	Azithromycin	0	12	4/4/2015	78.8	HEIGHT	10.85	17.5		1	0
24	5961	24	Azithromycin	0	54	4/4/2017	94.7	HEIGHT	13.15	15		1	0
24	5961	36	Azithromycin	0	68	5/23/2018	103.6	HEIGHT	15.9	16		0	0
24	5962	12	Azithromycin	1	12	6/20/2016	74.6	LENGTH	8.85	13		1	0
24	5962	24	Azithromycin	1	24	4/4/2017	81.9	HEIGHT	10.45	14.5		0	0
24	5962	36	Azithromycin	1	37	5/23/2018	90.3	HEIGHT	12.35	14.5		1	0
24	5962	48	Azithromycin	1	48	4/28/2019	96.9	HEIGHT	13.3	13.5		0	0
24	5962	60	Azithromycin	1	58	2/19/2020	102	HEIGHT	15.1	14.4		0	0
24	5964	0	Azithromycin	1	24	4/4/2015	77.2	HEIGHT	8.65	13.5		1	0
24	5964	24	Azithromycin	1	54	4/4/2017	93	HEIGHT	12.3	14.5		1	0
24	5964	36	Azithromycin	1	68	5/23/2018	103.5	HEIGHT	13.7	14		0	0
24	5966	12	Azithromycin	1	7	6/27/2016	67.1	HEIGHT	7.7	13.5		0	0
24	5966	48	Azithromycin	1	40	4/28/2019	94.8	HEIGHT	13.75	15		1	0
24	5966	60	Azithromycin	1	50	2/19/2020	101.7	HEIGHT	15.65	14.4		1	0
24	5968	0	Azithromycin	1	36	4/4/2015	93.2	HEIGHT	14.55	16.5		0	0
24	5968	36	Azithromycin	1	66	5/23/2018	115.6	HEIGHT	21.1	17.5		0	0
24	5968	48	Azithromycin	1	76	4/28/2019	121.3	HEIGHT	22.85	16		0	0
24	5968	60	Azithromycin	1	86	2/19/2020	126.9	HEIGHT	25.95	17.2		0	0
24	5969	0	Azithromycin	1	12	4/4/2015	75.2	HEIGHT	8.8	15	60	0	0
24	5969	12	Azithromycin	1	30	6/20/2016	86.8	HEIGHT	11.35	15.5	60	1	0
24	5969	24	Azithromycin	1	42	4/4/2017	94.3	HEIGHT	13.2	15	60	1	0
24	5969	36	Azithromycin	1	56	5/23/2018	103.6	HEIGHT	15.25	15.5	60	0	0
24	5970	0	Azithromycin	1	36	4/4/2015	83	HEIGHT	9.8	13		0	0
24	5970	12	Azithromycin	1	50	6/27/2016	92.2	HEIGHT	11.2	12.5		0	0
24	5970	24	Azithromycin	1	62	4/4/2017	97.5	HEIGHT	11.8	12		0	0
24	5970	36	Azithromycin	1	76	5/23/2018	104.8	HEIGHT	13.75	13		0	0
24	5970	60	Azithromycin	1	96	2/19/2020	116.5	HEIGHT	17.3	17.3		0	0
24	5978	24	Azithromycin	0	24	4/4/2017	81.2	HEIGHT	12.3	16		1	0
24	5979	0	Azithromycin	0	54	4/4/2015	108.3	HEIGHT	16.85	15.5		0	0
24	5979	12	Azithromycin	0	78	6/27/2016	114.5	HEIGHT	18.85	15.5		0	0

24	5979	48	Azithromycin	0	115	4/28/2019	127.6	HEIGHT	24.35	16.5		0	0
24	5979	60	Azithromycin	0	125	2/19/2020	133.9	HEIGHT	26.65	16.9		0	0
24	5982	24	Azithromycin	1	12	4/4/2017	70.7	LENGTH	7.45	14		1	0
24	5982	36	Azithromycin	1	26	5/23/2018	79.3	HEIGHT	10.15	15.5		0	0
24	5982	48	Azithromycin	1	37	4/28/2019	86	HEIGHT	12.15	15.5		1	0
24	5982	60	Azithromycin	1	46	2/19/2020	93.5	HEIGHT	13.9	16.2		0	0
24	5985	12	Azithromycin	0	6	6/27/2016	64.3	HEIGHT	6.15	13.5		0	0
24	5985	24	Azithromycin	0	16	4/4/2017	70	LENGTH	7.1	12.5		0	0
24	5985	36	Azithromycin	0	30	5/23/2018	82.3	HEIGHT	10.2	15		0	0
24	5985	48	Azithromycin	0	41	4/28/2019	87.1	HEIGHT	11.5	14.5		1	0
24	5988	24	Azithromycin	0	24	4/4/2017	75.8	HEIGHT	8.75	13		1	0
24	5988	36	Azithromycin	0	37	5/23/2018	88.1	HEIGHT	11.2	14		1	0
24	5989	0	Azithromycin	1	36	4/4/2015	90.7	HEIGHT	12.5	14		0	0
24	5989	12	Azithromycin	1	54	6/27/2016	101	HEIGHT	15.4	15.5		0	0
24	5989	36	Azithromycin	1	80	5/23/2018	112.8	HEIGHT	19.25	16		0	0
24	5989	48	Azithromycin	1	91	4/28/2019	118.1	HEIGHT	20.55	15.5		0	0
24	5989	60	Azithromycin	1	101	2/19/2020	123.1	HEIGHT	24.4	17		0	0
24	5990	24	Azithromycin	1	4	4/4/2017	60.1	LENGTH	6.45	15		1	0
24	5990	36	Azithromycin	1	19	5/23/2018	73.8	LENGTH	8.8	14		0	0
24	5990	48	Azithromycin	1	30	4/28/2019	80.6	HEIGHT	10.35	14.5		1	0
24	5990	60	Azithromycin	1	39	2/19/2020	87.2	HEIGHT	12.2	15		1	0
24	5994	0	Azithromycin	1	12	4/4/2015	64.7	LENGTH	6.5	13.5		0	0
24	5994	12	Azithromycin	1	27	6/20/2016	77.7	HEIGHT	8.45	14		1	0
24	5994	24	Azithromycin	1	40	4/4/2017	83.4	HEIGHT	9.95	13.5		0	0
24	5995	0	Azithromycin	1	12	4/4/2015	79.3	HEIGHT	9.15	13.5		1	0
24	5995	12	Azithromycin	1	30	6/20/2016	88.6	HEIGHT	11.35	14		1	0
24	5995	24	Azithromycin	1	42	4/4/2017	94	HEIGHT	13	14.5		0	0
24	5995	36	Azithromycin	1	56	5/23/2018	102.9	HEIGHT	14.55	14		1	0
24	5995	48	Azithromycin	1	67	4/28/2019	108	HEIGHT	16.65	14		0	0
24	5995	60	Azithromycin	1	77	2/19/2020	113.9	HEIGHT	18.8	15		0	0
24	5999	0	Azithromycin	0	12	4/4/2015	76.6	HEIGHT	8.95	14.5		0	0
24	5999	12	Azithromycin	0	27	6/27/2016	83.9	LENGTH	11.8	14		0	0
24	5999	24	Azithromycin	0	42	4/4/2017	91.2	HEIGHT	13.5	15.5		1	0
24	5999	36	Azithromycin	0	55	5/23/2018	98.9	HEIGHT	15.55	16		1	0
24	5999	48	Azithromycin	0	66	4/28/2019	105.2	HEIGHT	16.8	16		0	0
24	5999	60	Azithromycin	0	76	2/19/2020	111.5	HEIGHT	18.8	15.7		0	0
24	6000	0	Azithromycin	0	30	4/4/2015	92.5	HEIGHT	14.4	16.5	18	0	0
24	6000	12	Azithromycin	0	45	6/27/2016	99.7	HEIGHT	15.95	16	18	0	0
24	6001	0	Azithromycin	0	8	4/4/2015	77.6	LENGTH	10.95	16		0	0
24	6001	24	Azithromycin	0	37	4/4/2017	95	HEIGHT	16.75	17.5		0	0

24	6001	36	Azithromycin	0	51	5/23/2018	105.3	HEIGHT	19.05	18	1	0
24	6001	48	Azithromycin	0	61	4/28/2019	111.3	HEIGHT	19.25	16	0	0
24	6001	60	Azithromycin	0	71	2/19/2020	116.4	HEIGHT	21.5	15.5	0	0
24	6002	12	Azithromycin	1	53	6/20/2016	97.6	HEIGHT	11.85	14	1	0
24	6003	0	Azithromycin	1	12	4/4/2015	68.3	LENGTH	6.55	11	1	0
24	6003	24	Azithromycin	1	40	4/4/2017	86.5	HEIGHT	10.35	12.5	1	0
24	6003	36	Azithromycin	1	53	5/23/2018	95.7	HEIGHT	12.85	13	0	0
24	6003	48	Azithromycin	1	57	4/28/2019	102.3	HEIGHT	13.95	13	0	0
24	6003	60	Azithromycin	1	67	2/19/2020	109.5	HEIGHT	16.6	13.5	0	0
24	6005	0	Azithromycin	0	36	4/4/2015	88.5	HEIGHT	12.3	15.5	0	0
24	6005	12	Azithromycin	0	54	6/27/2016	96.5	HEIGHT	14.7	15.5	0	0
24	6005	24	Azithromycin	0	67	4/4/2017	100.5	HEIGHT	15.35	15	0	0
24	6005	36	Azithromycin	0	80	5/23/2018	107.7	HEIGHT	17.5	15.5	0	0
24	6006	24	Azithromycin	1	52	4/4/2017	95.7	HEIGHT	13.6	14	1	0
24	6008	24	Azithromycin	0	30	4/4/2017	95	HEIGHT	13.5	15	1	0
24	6008	48	Azithromycin	0	56	4/28/2019	109.6	HEIGHT	16.25	15	1	0
24	6008	60	Azithromycin	0	56	2/19/2020	116.2	HEIGHT	17.6	14.3	1	0
24	6010	24	Azithromycin	1	54	4/4/2017	98.3	HEIGHT	13.9	14.5	1	0
24	6011	12	Azithromycin	0	2	6/27/2016	61.8	LENGTH	6.55	14	0	0
24	6011	24	Azithromycin	0	13	4/4/2017	72.7	HEIGHT	8.9	15.5	0	0
24	6011	36	Azithromycin	0	27	5/23/2018	81.7	HEIGHT	10.55	14.5	0	0
24	6011	48	Azithromycin	0	38	4/28/2019	84.7	HEIGHT	12.05	16	1	0
24	6011	60	Azithromycin	1	47	2/19/2020	92.3	HEIGHT	14.2	16	1	0
24	6012	12	Azithromycin	1	5	6/27/2016	64.7	LENGTH	7.45	14.5	0	0
24	6012	24	Azithromycin	1	16	4/4/2017	74.2	HEIGHT	9.25	15.5	1	0
24	6012	48	Azithromycin	1	40	4/28/2019	88.8	HEIGHT	12.5	15.5	1	0
24	6012	60	Azithromycin	1	50	2/19/2020	95.2	HEIGHT	13.75	15.7	0	0
24	6013	0	Azithromycin	1	30	4/4/2015	92	HEIGHT	12.1	14.5	0	0
24	6013	12	Azithromycin	1	42	6/20/2016	99.9	HEIGHT	14.55	14.5	1	0
24	6014	0	Azithromycin	1	48	4/4/2015	96.9	HEIGHT	14.55	15	0	0
24	6014	24	Azithromycin	1	74	4/4/2017	109.1	HEIGHT	17.15	15	0	0
24	6018	0	Azithromycin	1	54	4/4/2015	103.4	HEIGHT	13.85	13.5	1	0
24	6019	0	Azithromycin	0	36	4/4/2015	109.4	HEIGHT	16.8	15	0	0
24	6019	12	Azithromycin	0	54	6/27/2016	115.3	HEIGHT	18.95	15.5	0	0
24	6019	24	Azithromycin	0	64	4/4/2017	120	HEIGHT	21.25	17	0	0
24	6019	48	Azithromycin	0	88	4/28/2019	128	HEIGHT	24.3	16.5	0	0
24	6020	0	Azithromycin	1	2	4/4/2015	59.1	LENGTH	5.4	12.5	1	0
24	6020	12	Azithromycin	1	18	6/27/2016	73.5	HEIGHT	8.1	14.5	0	0
24	6020	24	Azithromycin	1	31	4/4/2017	78.2	HEIGHT	9.75	15	0	0
24	6020	36	Azithromycin	1	44	5/23/2018	88	HEIGHT	12.1	16.5	0	0

24	6020	60	Azithromycin	1	55	2/19/2020	97.8	HEIGHT	14	15.2		1	0
24	6021	24	Azithromycin	1	8	4/4/2017	62.4	LENGTH	6.3	13.5		1	0
24	6021	36	Azithromycin	1	22	5/23/2018	75.4	HEIGHT	7.95	13		1	0
24	6021	48	Azithromycin	1	33	4/28/2019	77.6	LENGTH	8.85	13		0	0
24	6021	60	Azithromycin	1	42	2/19/2020	85.4	HEIGHT	10.05	12.8		0	0
24	6022	0	Azithromycin	1	48	4/4/2015	115.7	HEIGHT	17.8	14		1	0
24	6022	12	Azithromycin	1	65	6/27/2016	120.9	HEIGHT	20.6	15.5		0	0
24	6022	24	Azithromycin	1	76	4/4/2017	124	HEIGHT	21.9	16.5		0	0
24	6022	36	Azithromycin	1	89	5/23/2018	128.4	HEIGHT	24	16.5		0	0
24	6022	48	Azithromycin	1	100	4/28/2019	131.4	HEIGHT	26.4	16.5		0	0
24	6023	0	Azithromycin	0	54	4/4/2015	131	HEIGHT	24.15	16.5		1	0
24	6023	48	Azithromycin	0	151	4/28/2019	147	HEIGHT	37.15	18.5		0	0
24	6024	24	Azithromycin	0	16	4/4/2017	75.2	HEIGHT	8.65	14		0	0
24	6024	36	Azithromycin	0	28	5/23/2018	85.4	HEIGHT	10.85	15		0	0
24	6026	0	Azithromycin	0	48	4/4/2015	83.1	HEIGHT	10.15	14.5		1	0
24	6026	12	Azithromycin	0	30	6/20/2016	92	LENGTH	12.95	15.5		1	0
24	6026	24	Azithromycin	0	42	4/4/2017	98.5	HEIGHT	14.85	15.5		0	0
24	6026	36	Azithromycin	0	56	5/23/2018	105.1	HEIGHT	16.45	15.5		1	0
24	6026	48	Azithromycin	0	67	4/28/2019	109.6	HEIGHT	16.95	14		0	0
24	6026	60	Azithromycin	0	77	2/19/2020	113.9	HEIGHT	19.35	15.2		0	0
24	6028	24	Azithromycin	0	4	4/4/2017	64.1	LENGTH	6.1	14.5		1	0
24	6028	36	Azithromycin	0	15	5/23/2018	76.3	LENGTH	9.9	15.5		1	0
24	6028	48	Azithromycin	0	26	4/28/2019	82.1	HEIGHT	11.4	15.5		1	0
24	6028	60	Azithromycin	0	35	2/19/2020	89	HEIGHT	14.5	17.3		1	0
24	6031	0	Azithromycin	1	48	4/4/2015	97.4	HEIGHT	13	14		1	0
24	6031	12	Azithromycin	1	42	6/27/2016	105.1	HEIGHT	14.55	14		0	0
24	6031	24	Azithromycin	1	54	4/4/2017	110.3	HEIGHT	16.35	14		0	0
24	6031	36	Azithromycin	1	68	5/23/2018	116.5	HEIGHT	18.05	14.5		0	0
24	6031	48	Azithromycin	1	79	4/28/2019	122.3	HEIGHT	19.5	14.5		0	0
24	6031	60	Azithromycin	1	89	2/19/2020	127.6	HEIGHT	22.85	15.1		0	0
24	6032	12	Azithromycin	1	10	6/20/2016	69.6	LENGTH	7.85	14.5		1	0
24	6032	24	Azithromycin	1	16	4/4/2017	77.8	HEIGHT	9.1	14.5		0	0
24	6032	36	Azithromycin	1	34	5/23/2018	85.6	HEIGHT	10.8	14		1	0
24	6032	48	Azithromycin	1	44	4/28/2019	92.9	HEIGHT	12.7	14		1	0
24	6032	60	Azithromycin	1	54	2/19/2020	99.2	HEIGHT	14.1	14		0	0
24	6036	0	Azithromycin	0	42	4/4/2015	86.4	HEIGHT	11.45	14		1	0
24	6036	36	Azithromycin	0	68	5/23/2018	107.4	HEIGHT	16	14.5		0	0
24	6036	48	Azithromycin	0	79	4/28/2019	111.7	HEIGHT	16.9	14		0	0
24	6036	60	Azithromycin	0	89	2/19/2020	117.2	HEIGHT	19.8	14		0	0
24	6041	12	Azithromycin	0	12	6/20/2016	70	HEIGHT	6.9	12.5	42	1	0

24	6041	36	Azithromycin	0	37	5/23/2018	84.2	HEIGHT	10.5	14.5	42	0	0
24	6046	24	Azithromycin	0	8	4/4/2017	65.1	LENGTH	6.8	13.5		1	0
24	6046	36	Azithromycin	0	21	5/23/2018	77.6	LENGTH	9.35	13.5		1	0
24	6046	48	Azithromycin	0	32	4/28/2019	85.8	HEIGHT	10.75	13		0	0
24	6046	60	Azithromycin	0	41	2/19/2020	92.1	HEIGHT	13.25	13.2		0	0
24	6051	24	Azithromycin	0	22	4/4/2017	73.8	HEIGHT	9.5	15		0	0
24	6051	36	Azithromycin	0	40	5/23/2018	81.4	HEIGHT	10.95	15.5		1	0
24	6051	48	Azithromycin	0	51	4/28/2019	90.3	HEIGHT	13	15.5		1	0
24	6051	60	Azithromycin	0	60	2/19/2020	96	HEIGHT	14.4	15.2		0	0
24	6055	0	Azithromycin	0	48	4/4/2015	98.3	HEIGHT	14.85	15		1	0
24	6055	12	Azithromycin	0	62	6/27/2016	105	HEIGHT	16.15	15.5		0	0
24	6055	24	Azithromycin	0	74	4/4/2017	110.3	HEIGHT	18.2	15.5		0	0
24	6055	36	Azithromycin	0	88	5/23/2018	117.2	HEIGHT	19.7	15.5		0	0
24	6055	48	Azithromycin	0	98	4/28/2019	121.6	HEIGHT	22.25	16		0	0
24	6057	0	Azithromycin	0	36	4/4/2015	105.7	HEIGHT	15.8	14.5		0	0
24	6057	48	Azithromycin	0	88	4/28/2019	125.2	HEIGHT	23.3	16		0	0
24	6059	12	Azithromycin	1	30	6/20/2016	81.5	LENGTH	10.1	14		1	0
24	6063	0	Azithromycin	0	12	4/4/2015	73.6	HEIGHT	8	13		1	0
24	6063	12	Azithromycin	0	29	6/27/2016	84.1	HEIGHT	10.6	14		0	0
24	6063	24	Azithromycin	0	40	4/4/2017	91.4	HEIGHT	11.75	14		1	0
24	6065	0	Azithromycin	1	36	4/4/2015	87.6	HEIGHT	10.95	13.5	18	0	0
24	6066	0	Azithromycin	0	36	4/4/2015	97	HEIGHT	13.1	13.5		1	0
24	6066	12	Azithromycin	0	42	6/20/2016	105.5	HEIGHT	14.55	13.5		1	0
24	6066	24	Azithromycin	0	54	4/4/2017	109.2	HEIGHT	15.9	14		1	0
24	6066	36	Azithromycin	0	68	5/23/2018	115	HEIGHT	17.3	13.5		0	0
24	6066	48	Azithromycin	0	79	4/28/2019	118.9	HEIGHT	19.75	14		0	0
24	6066	60	Azithromycin	0	89	2/19/2020	125.3	HEIGHT	21.8	14.6		0	0
24	6068	0	Azithromycin	1	36	4/4/2015	78.3	HEIGHT	11.05	15		0	0
24	6068	12	Azithromycin	1	51	6/20/2016	88.2	HEIGHT	13.65	16		1	0
24	6068	24	Azithromycin	1	64	4/4/2017	93.7	HEIGHT	14.95	16		0	0
24	6068	36	Azithromycin	1	77	5/23/2018	101.1	HEIGHT	17.2	16		0	0
24	6069	0	Azithromycin	1	11	4/4/2015	71.5	LENGTH	7.9	14.5		0	0
24	6069	12	Azithromycin	1	24	6/20/2016	80.3	HEIGHT	9.1	14.5		1	0
24	6069	24	Azithromycin	1	36	4/4/2017	86.7	HEIGHT	11.05	14.5		1	0
24	6069	36	Azithromycin	1	49	5/23/2018	94.8	HEIGHT	13.05	15.5		1	0
24	6069	48	Azithromycin	1	60	4/28/2019	100.7	HEIGHT	13.65	14		0	0
24	6069	60	Azithromycin	1	70	2/19/2020	106.6	HEIGHT	15.6	15		0	0
24	6070	0	Azithromycin	0	8	4/4/2015	66.6	LENGTH	6.35	12.5		1	0
24	6070	24	Azithromycin	0	32	4/4/2017	82.3	HEIGHT	10.7	15.5		0	0
24	6070	36	Azithromycin	0	46	5/23/2018	90.6	HEIGHT	12.75	15.5		0	0

24	6070	60	Azithromycin	0	55	2/19/2020	101.4	HEIGHT	14.75	15	0	0
24	6071	0	Azithromycin	1	30	4/4/2015	86.8	HEIGHT	11.5	15.5	0	0
24	6072	0	Azithromycin	0	24	4/4/2015	85.9	HEIGHT	11.55	15	1	0
24	6072	24	Azithromycin	0	55	4/4/2017	104.9	HEIGHT	17	16	1	0
24	6072	36	Azithromycin	0	68	5/23/2018	113.8	HEIGHT	19.25	16	0	0
24	6072	48	Azithromycin	0	79	4/28/2019	119.3	HEIGHT	22.6	16	0	0
24	6073	0	Azithromycin	0	54	4/4/2015	118.5	HEIGHT	19.2	15.5	0	0
24	6073	48	Azithromycin	0	106	4/28/2019	135	HEIGHT	28.2	17.5	0	0
24	6074	0	Azithromycin	0	54	4/4/2015	97.7	HEIGHT	13.35	14	0	0
24	6074	12	Azithromycin	0	68	6/27/2016	104.6	HEIGHT	15.3	14.5	0	0
24	6074	24	Azithromycin	0	82	4/4/2017	108.4	HEIGHT	16.85	14.5	0	0
24	6074	36	Azithromycin	0	95	5/23/2018	113.3	HEIGHT	18	16	0	0
24	6074	48	Azithromycin	0	106	4/28/2019	117.6	HEIGHT	20.05	15.5	0	0
24	6075	0	Azithromycin	0	48	6/13/2015	97.9	HEIGHT	14.8	14.5	1	0
24	6075	48	Azithromycin	0	103	4/28/2019	123	HEIGHT	22.6	16	0	0
24	6075	60	Azithromycin	0	112	2/19/2020	127.6	HEIGHT	24.65	16.5	0	0
24	6077	0	Azithromycin	0	24	4/4/2015	86.5	HEIGHT	10.85	14.5	0	0
24	6077	12	Azithromycin	0	40	6/20/2016	99.1	HEIGHT	13.1	15	1	0
24	6077	24	Azithromycin	0	50	4/4/2017	99.9	HEIGHT	14.85	15.5	1	0
24	6077	36	Azithromycin	0	64	5/23/2018	107.6	HEIGHT	15.95	14.5	0	0
24	6077	48	Azithromycin	0	74	4/28/2019	111.4	HEIGHT	17.35	15	0	0
24	6077	60	Azithromycin	0	84	2/19/2020	117.4	HEIGHT	18.8	15	0	0
24	6081	12	Azithromycin	1	3	6/20/2016	65.4	LENGTH	6.2	13	1	0
24	6081	24	Azithromycin	1	12	4/4/2017	74.8	HEIGHT	8.15	14	0	0
24	6081	36	Azithromycin	1	30	5/23/2018	85.4	HEIGHT	10.35	14	0	0
24	6081	48	Azithromycin	1	41	4/28/2019	93.4	HEIGHT	12.05	13	0	0
24	6081	60	Azithromycin	1	50	2/19/2020	98.4	HEIGHT	13	13	0	0
24	6083	0	Azithromycin	0	54	4/4/2015	108	HEIGHT	16.6	15.5	0	0
24	6083	12	Azithromycin	0	78	6/27/2016	113.5	HEIGHT	18.1	16	0	0
24	6083	60	Azithromycin	0	125	2/19/2020	127.5	HEIGHT	25.4	17.8	0	0
24	6084	0	Azithromycin	0	54	4/4/2015	101.5	HEIGHT	17.05	16	0	0
24	6084	12	Azithromycin	0	60	6/27/2016	108.7	HEIGHT	17.75	15.5	0	0
24	6084	24	Azithromycin	0	73	4/4/2017	112.2	HEIGHT	18.8	16	0	0
24	6084	36	Azithromycin	0	86	5/23/2018	117.4	HEIGHT	21.35	16.5	0	0
24	6084	48	Azithromycin	0	97	4/28/2019	120.4	HEIGHT	22.65	17	0	0
24	6084	60	Azithromycin	0	107	2/19/2020	124.8	HEIGHT	26	17.5	0	0
24	6088	0	Azithromycin	1	54	4/4/2015	96.1	HEIGHT	11.8	13.5	1	0
24	6088	12	Azithromycin	1	68	6/27/2016	103.1	HEIGHT	13.4	13.5	0	0
24	6088	24	Azithromycin	1	80	4/4/2017	108.4	HEIGHT	14.6	14	0	0
24	6088	36	Azithromycin	1	94	5/23/2018	114.2	HEIGHT	15.8	13.5	0	0

24	6088	60	Azithromycin	1	114	2/19/2020	123.4	HEIGHT	18.95	14.3		0	0
24	6089	0	Azithromycin	0	36	4/4/2015	85	HEIGHT	11.1	13.5		0	0
24	6089	12	Azithromycin	0	51	6/27/2016	93.4	HEIGHT	13.25	14		0	0
24	6089	24	Azithromycin	0	64	4/4/2017	101.1	HEIGHT	14.8	14.5		0	0
24	6089	48	Azithromycin	0	88	4/28/2019	113.8	HEIGHT	18.6	14.5		0	0
24	6089	60	Azithromycin	0	98	2/19/2020	120.4	HEIGHT	22.25	15.2		0	0
24	6091	0	Azithromycin	1	24	4/4/2015	92.7	HEIGHT	14.85	17	18	1	0
24	6091	12	Azithromycin	1	42	6/20/2016	101	HEIGHT	16.6	17	18	1	0
24	6092	0	Azithromycin	1	54	4/4/2015	104.6	HEIGHT	16.3	15		0	0
24	6092	12	Azithromycin	1	69	6/27/2016	112.5	HEIGHT	18.4	16		0	0
24	6092	24	Azithromycin	1	82	4/4/2017	117	HEIGHT	20.5	16		0	0
24	6092	48	Azithromycin	1	106	4/28/2019	126.6	HEIGHT	24.55	16		0	0
24	6092	60	Azithromycin	1	116	2/19/2020	132.1	HEIGHT	27.2	17		0	0
24	6093	0	Azithromycin	1	36	4/4/2015	93.8	HEIGHT	12.35	14.5		1	0
24	6093	12	Azithromycin	1	56	6/20/2016	102.9	HEIGHT	14.3	14.5		1	0
24	6093	24	Azithromycin	1	67	4/4/2017	108.9	HEIGHT	15.2	14		0	0
24	6093	36	Azithromycin	1	80	5/23/2018	116.7	HEIGHT	17.4	14		0	0
24	6093	48	Azithromycin	1	91	4/28/2019	121.3	HEIGHT	18.05	13.5		0	0
24	6093	60	Azithromycin	1	101	2/19/2020	126.7	HEIGHT	21.3	15		0	0
24	6095	0	Azithromycin	0	24	4/4/2015	76.4	HEIGHT	7.3	12		1	0
24	6095	12	Azithromycin	0	41	6/20/2016	83	HEIGHT	7.95	12.5		1	0
24	6095	24	Azithromycin	0	52	4/4/2017	88.5	HEIGHT	9.25	13		0	0
24	6095	48	Azithromycin	0	76	4/28/2019	101.1	HEIGHT	11.6	12		0	0
24	6095	60	Azithromycin	0	86	2/19/2020	107.3	HEIGHT	13.55	13		0	0
24	6096	0	Azithromycin	1	42	4/4/2015	88.5	HEIGHT	11.7	14.5		0	0
24	6096	12	Azithromycin	1	54	6/20/2016	97.2	HEIGHT	13.1	14.5		1	0
24	6096	24	Azithromycin	1	66	4/4/2017	102	HEIGHT	14.85	15		0	0
24	6096	48	Azithromycin	1	91	4/28/2019	113.7	HEIGHT	17.55	15.5		0	0
24	6097	0	Azithromycin	1	54	4/4/2015	106.8	HEIGHT	16.35	15		0	0
24	6097	12	Azithromycin	1	78	6/27/2016	114.5	HEIGHT	19.15	16		0	0
24	6098	0	Azithromycin	1	36	4/4/2015	90.3	HEIGHT	13.5	15.5	48	1	0
24	6098	12	Azithromycin	1	50	6/20/2016	99.9	HEIGHT	14.9	16	48	1	0
24	6098	24	Azithromycin	1	64	4/4/2017	104.2	HEIGHT	16.2	15.5	48	0	0
24	6098	36	Azithromycin	1	77	5/23/2018	110.5	HEIGHT	18.7	16	48	0	0
24	6099	24	Azithromycin	1	26	4/4/2017	75.8	HEIGHT	8.65	13.5		1	0
24	6099	36	Azithromycin	1	40	5/23/2018	83.2	HEIGHT	10.4	13.5		1	0
24	6100	0	Azithromycin	0	54	4/4/2015	101	HEIGHT	14.05	13.5		0	0
24	6100	12	Azithromycin	0	69	6/27/2016	110.2	HEIGHT	16.05	14		0	0
24	6100	24	Azithromycin	0	82	4/4/2017	115.2	HEIGHT	16.85	14		0	0
24	6102	0	Azithromycin	0	24	4/4/2015	71.9	LENGTH	7.55	12		1	0

24	6102	12	Azithromycin	0	42	6/20/2016	80	HEIGHT	9.9	14.5	1	0
24	6104	0	Azithromycin	0	36	4/4/2015	88.8	HEIGHT	12.5	15	0	0
24	6104	24	Azithromycin	0	54	4/4/2017	105	HEIGHT	15.55	15.5	0	0
24	6104	36	Azithromycin	0	68	5/23/2018	112.8	HEIGHT	17.25	15	0	0
24	6104	48	Azithromycin	0	79	4/28/2019	116.9	HEIGHT	19.5	15.5	0	0
24	6105	24	Azithromycin	1	24	4/4/2017	74.5	HEIGHT	9.85	14.5	1	0
24	6105	60	Azithromycin	1	58	2/19/2020	99.7	HEIGHT	15.85	15.5	1	0
24	6106	0	Azithromycin	0	54	4/4/2015	118.5	HEIGHT	20.5	16	0	0
24	6109	0	Azithromycin	1	7	4/4/2015	102.9	HEIGHT	12.75	13.5	1	0
24	6109	12	Azithromycin	1	60	6/27/2016	111	HEIGHT	15.2	15	0	0
24	6109	24	Azithromycin	1	73	4/4/2017	116.3	HEIGHT	15.9	14	0	0
24	6109	48	Azithromycin	1	97	4/28/2019	128.3	HEIGHT	20.4	15	0	0
24	6109	60	Azithromycin	1	107	2/19/2020	133.4	HEIGHT	24.2	16.9	0	0
24	6110	0	Azithromycin	0	24	4/4/2015	75.5	HEIGHT	9.15	13.5	0	0
24	6110	24	Azithromycin	0	50	4/4/2017	87.8	HEIGHT	12.3	14	1	0
24	6110	48	Azithromycin	0	74	4/28/2019	104.2	HEIGHT	15	14	0	0
24	6111	0	Azithromycin	0	54	4/4/2015	117.5	HEIGHT	18.85	15.5	0	0
24	6111	12	Azithromycin	0	72	6/27/2016	123.6	HEIGHT	21.5	16.5	0	0
24	6111	24	Azithromycin	0	82	4/4/2017	126.5	HEIGHT	23.75	16.5	0	0
24	6113	0	Azithromycin	0	4	4/4/2015	68.1	LENGTH	7.9	14	0	0
24	6113	24	Azithromycin	0	30	4/4/2017	81.9	HEIGHT	13.75	18.5	1	0
24	6113	36	Azithromycin	0	44	5/23/2018	93.6	HEIGHT	16.25	16.5	1	0
24	6114	24	Azithromycin	1	12	4/4/2017	71.3	LENGTH	8.2	15.5	1	0
24	6114	48	Azithromycin	1	36	4/28/2019	84.2	HEIGHT	10.6	14.5	0	0
24	6115	24	Azithromycin	0	4	4/4/2017	64	LENGTH	7.1	15	1	0
24	6115	60	Azithromycin	0	35	2/19/2020	92.9	HEIGHT	14	15.5	0	0
24	6119	24	Azithromycin	0	16	4/4/2017	75.3	HEIGHT	8.85	14.5	1	0
24	6119	36	Azithromycin	0	29	5/23/2018	84.1	HEIGHT	10.7	14.5	1	0
24	6120	0	Azithromycin	1	36	4/4/2015	63.1	LENGTH	6.55	13.5	0	0
24	6122	0	Azithromycin	0	12	4/4/2015	74.4	HEIGHT	8.35	14	1	0
24	6122	12	Azithromycin	0	30	6/20/2016	84.9	HEIGHT	11.05	15	1	0
24	6122	24	Azithromycin	0	43	4/4/2017	92.6	HEIGHT	13.3	15.5	1	0
24	6122	48	Azithromycin	0	67	4/28/2019	108.6	HEIGHT	17.3	15	0	0
24	6123	0	Azithromycin	0	36	4/4/2015	92	HEIGHT	12.15	14.5	0	0
24	6123	12	Azithromycin	0	54	6/27/2016	100	HEIGHT	14.3	14	0	0
24	6123	24	Azithromycin	0	66	4/4/2017	105.4	HEIGHT	16.15	14.5	0	0
24	6123	48	Azithromycin	0	91	4/28/2019	116.1	HEIGHT	19.85	15	0	0
24	6123	60	Azithromycin	0	101	2/19/2020	121	HEIGHT	21.6	15.5	0	0
24	6125	12	Azithromycin	0	9	6/27/2016	72.8	HEIGHT	8.1	13.5	0	0
24	6125	24	Azithromycin	0	21	4/4/2017	79.1	HEIGHT	10.05	14.5	1	0

24	6125	36	Azithromycin	0	35	5/23/2018	87	HEIGHT	11.95	15	0	0
24	6125	48	Azithromycin	0	46	4/28/2019	91.9	HEIGHT	12.45	14	1	0
24	6125	60	Azithromycin	0	55	2/19/2020	98.2	HEIGHT	14.15	14	1	0
24	6126	0	Azithromycin	0	24	4/4/2015	74.4	HEIGHT	8.85	14	1	0
24	6126	12	Azithromycin	0	38	6/20/2016	82.5	HEIGHT	11.3	15	1	0
24	6126	24	Azithromycin	0	50	4/4/2017	91	HEIGHT	13.85	16	1	0
24	6126	36	Azithromycin	0	64	5/23/2018	99.8	HEIGHT	15.7	15.5	0	0
24	6126	48	Azithromycin	0	74	4/28/2019	104.5	HEIGHT	16.95	15.5	0	0
24	6127	0	Azithromycin	0	8	4/4/2015	72	LENGTH	9	15	0	0
24	6127	12	Azithromycin	0	24	6/20/2016	84.3	HEIGHT	11.8	14.5	1	0
24	6127	24	Azithromycin	0	37	4/4/2017	90.7	HEIGHT	12.95	15.5	1	0
24	6127	36	Azithromycin	0	49	5/23/2018	98.2	HEIGHT	15	16	1	0
24	6127	60	Azithromycin	0	69	2/19/2020	110.3	HEIGHT	18.3	15	0	0
24	6128	0	Azithromycin	0	7	4/4/2015	71.9	LENGTH	8.45	14	0	0
24	6128	12	Azithromycin	0	42	6/27/2016	84	HEIGHT	10.1	14.5	0	0
24	6128	24	Azithromycin	0	54	4/4/2017	89	HEIGHT	12.85	15	1	0
24	6128	48	Azithromycin	0	79	4/28/2019	105.5	HEIGHT	16.15	15	0	0
24	6128	60	Azithromycin	0	89	2/19/2020	112.2	HEIGHT	19.05	15.6	0	0
24	6129	0	Azithromycin	0	36	6/13/2015	82.9	HEIGHT	10.15	12.5	1	0
24	6129	12	Azithromycin	0	42	6/20/2016	91.9	HEIGHT	12.25	13.5	1	0
24	6129	48	Azithromycin	0	79	4/28/2019	109.5	HEIGHT	16.65	13.5	0	0
24	6129	60	Azithromycin	0	89	2/19/2020	115.7	HEIGHT	18.5	13.7	0	0
24	6130	0	Azithromycin	0	30	4/4/2015	88.3	HEIGHT	12.2	14.5	1	0
24	6130	24	Azithromycin	0	58	4/4/2017	103.7	HEIGHT	16.1	15	0	0
24	6130	36	Azithromycin	0	71	5/23/2018	109.6	HEIGHT	17.4	15	0	0
24	6130	48	Azithromycin	0	82	4/28/2019	115.1	HEIGHT	19.65	15	0	0
24	6131	0	Azithromycin	0	30	4/4/2015	82.6	HEIGHT	11.3	15	1	0
24	6131	24	Azithromycin	0	49	4/4/2017	97.5	HEIGHT	13.45	15	1	0
24	6131	36	Azithromycin	0	62	5/23/2018	102.3	HEIGHT	14.9	15	0	0
24	6131	48	Azithromycin	0	73	4/28/2019	106.7	HEIGHT	16.65	14.5	0	0
24	6132	0	Azithromycin	0	11	4/4/2015	71.1	LENGTH	7.5	12.5	0	0
24	6132	12	Azithromycin	0	24	6/20/2016	76.7	HEIGHT	8.6	12.5	1	0
24	6132	36	Azithromycin	0	41	5/23/2018	93.9	HEIGHT	13	14	0	0
24	6132	48	Azithromycin	0	52	4/28/2019	100.6	HEIGHT	15.1	14	1	0
24	6132	60	Azithromycin	0	61	2/19/2020	105.9	HEIGHT	16.05	13.5	0	0
24	6133	24	Azithromycin	0	11	4/4/2017	71.7	LENGTH	7.75	14	1	0
24	6133	36	Azithromycin	0	25	5/23/2018	82.4	HEIGHT	9.6	14	0	0
24	6133	60	Azithromycin	0	45	2/19/2020	94.2	HEIGHT	12.55	14	0	0
24	6135	0	Azithromycin	0	24	4/4/2015	80.2	HEIGHT	11.4	15.5	1	0
24	6135	24	Azithromycin	0	47	4/4/2017	96.1	HEIGHT	15.6	16	0	0

24	6135	36	Azithromycin	0	61	5/23/2018	102.8	HEIGHT	17.15	16.5	0	0
24	6135	48	Azithromycin	0	72	4/28/2019	107.6	HEIGHT	18.85	15.5	0	0
24	6136	0	Azithromycin	0	54	4/4/2015	123.5	HEIGHT	23.35	17	0	0
24	6136	12	Azithromycin	0	69	6/27/2016	129.3	HEIGHT	27.1	19	0	0
24	6136	24	Azithromycin	0	82	4/4/2017	135.2	HEIGHT	29.45	18.5	0	0
24	6136	48	Azithromycin	0	106	4/28/2019	145.4	HEIGHT	35.75	20	0	0
24	6136	60	Azithromycin	0	116	2/19/2020	147.8	HEIGHT	40	20.5	0	0
24	6137	0	Azithromycin	0	36	4/4/2015	83.3	HEIGHT	9.95	14.5	0	0
24	6137	12	Azithromycin	0	51	6/27/2016	91.4	HEIGHT	11.75	15	0	0
24	6137	36	Azithromycin	0	77	5/23/2018	103.7	HEIGHT	13.3	13.5	0	0
24	6137	48	Azithromycin	0	88	4/28/2019	108.3	HEIGHT	14.7	13.5	0	0
24	6139	12	Azithromycin	1	9	6/27/2016	67.4	HEIGHT	6.1	12	0	0
24	6139	24	Azithromycin	1	16	4/4/2017	75.2	HEIGHT	7.55	12.5	0	0
24	6139	48	Azithromycin	1	40	4/28/2019	90.3	HEIGHT	11.1	13.5	1	0
24	6139	60	Azithromycin	1	50	2/19/2020	96.6	HEIGHT	12.1	13.5	1	0
24	6140	0	Azithromycin	1	48	4/4/2015	125.3	HEIGHT	25.25	18.5	1	0
24	6140	36	Azithromycin	1	97	5/23/2018	140.2	HEIGHT	35.15	21.5	0	0
24	6140	48	Azithromycin	1	108	4/28/2019	145.8	HEIGHT	38.95	22	0	0
24	6147	0	Azithromycin	1	54	4/4/2015	114.6	HEIGHT	18.55	16	0	0
24	6147	12	Azithromycin	1	72	6/27/2016	122	HEIGHT	21.8	17	0	0
24	6147	24	Azithromycin	1	82	4/4/2017	125.2	HEIGHT	23.8	16.5	0	0
24	6147	48	Azithromycin	1	106	4/28/2019	134.6	HEIGHT	28.1	17.5	0	0
24	6148	0	Azithromycin	1	36	4/4/2015	100.9	HEIGHT	15.85	16	0	0
24	6148	12	Azithromycin	1	51	6/20/2016	108.2	HEIGHT	17.8	16.5	1	0
24	6148	24	Azithromycin	1	64	4/4/2017	112.6	HEIGHT	20.1	17	0	0
24	6148	36	Azithromycin	1	77	5/23/2018	118.2	HEIGHT	22.8	18	0	0
24	6148	48	Azithromycin	1	88	4/28/2019	123.1	HEIGHT	24.7	17.5	0	0
24	6148	60	Azithromycin	1	98	2/19/2020	128.4	HEIGHT	28.05	18.3	0	0
24	6149	0	Azithromycin	1	48	4/4/2015	112.2	HEIGHT	16.6	14	0	0
24	6149	48	Azithromycin	1	98	4/28/2019	131	HEIGHT	23.65	15	0	0
24	6150	24	Azithromycin	1	16	4/4/2017	74.4	HEIGHT	7.75	13.5	1	0
24	6150	36	Azithromycin	1	34	5/23/2018	86.3	HEIGHT	11	15	1	0
24	6150	60	Azithromycin	1	54	2/19/2020	99.4	HEIGHT	13.95	14.5	0	0
24	6151	0	Azithromycin	1	42	4/4/2015	93.9	HEIGHT	11.95	13.5	0	0
24	6151	12	Azithromycin	1	57	6/20/2016	102.9	HEIGHT	13.9	14	1	0
24	6151	24	Azithromycin	1	70	4/4/2017	107.5	HEIGHT	15.3	14	0	0
24	6151	36	Azithromycin	1	83	5/23/2018	113.7	HEIGHT	17.4	14.5	0	0
24	6151	48	Azithromycin	1	94	4/28/2019	118.1	HEIGHT	19.1	14.5	0	0
24	6151	60	Azithromycin	1	104	2/19/2020	122.6	HEIGHT	21.1	15	0	0
24	6152	0	Azithromycin	1	30	4/4/2015	85.1	HEIGHT	11	15	1	0

24	6152	12	Azithromycin	1	42	6/20/2016	92.6	HEIGHT	12.95	16	1	0
24	6152	24	Azithromycin	1	55	4/4/2017	97	HEIGHT	13.55	15.5	0	0
24	6152	36	Azithromycin	1	68	5/23/2018	103	HEIGHT	15.15	15.5	0	0
24	6152	48	Azithromycin	1	79	4/28/2019	107.5	HEIGHT	16.2	15	0	0
24	6152	60	Azithromycin	1	89	2/19/2020	111.8	HEIGHT	17.7	15.5	0	0
24	6153	0	Azithromycin	0	54	4/4/2015	112.1	HEIGHT	16.5	13	1	0
24	6153	24	Azithromycin	0	66	4/4/2017	122.7	HEIGHT	20.2	14	0	0
24	6153	48	Azithromycin	0	91	4/28/2019	131.2	HEIGHT	25.35	14.5	0	0
24	6153	60	Azithromycin	0	101	2/19/2020	136.4	HEIGHT	26.75	15	0	0
24	6155	0	Azithromycin	0	24	4/4/2015	81.2	HEIGHT	11	15	0	0
24	6156	0	Azithromycin	0	36	4/4/2015	91.4	HEIGHT	14	16.5	1	0
24	6156	24	Azithromycin	0	54	4/4/2017	106.5	HEIGHT	17.85	15.5	0	0
24	6156	36	Azithromycin	0	68	5/23/2018	114.3	HEIGHT	20.15	16.5	0	0
24	6157	0	Azithromycin	0	36	4/4/2015	93.9	HEIGHT	12.9	14	0	0
24	6157	12	Azithromycin	0	54	6/27/2016	102.4	HEIGHT	14	14	0	0
24	6157	24	Azithromycin	0	66	4/4/2017	106.7	HEIGHT	14.8	15	0	0
24	6157	36	Azithromycin	0	80	5/23/2018	112.7	HEIGHT	17.1	15	0	0
24	6157	48	Azithromycin	0	91	4/28/2019	116.9	HEIGHT	19.45	15	0	0
24	6157	60	Azithromycin	0	101	2/19/2020	122.8	HEIGHT	21.75	16	0	0
24	6158	12	Azithromycin	1	10	6/27/2016	68.6	HEIGHT	7.2	13.5	0	0
24	6158	24	Azithromycin	1	19	4/4/2017	74.5	HEIGHT	8.2	14.5	0	0
24	6158	48	Azithromycin	1	43	4/28/2019	92.8	HEIGHT	12.05	14	1	0
24	6158	60	Azithromycin	1	53	2/19/2020	101.1	HEIGHT	14.8	14	1	0
24	6159	0	Azithromycin	1	48	4/4/2015	100.2	HEIGHT	16.2	16.5	1	0
24	6159	12	Azithromycin	1	63	6/27/2016	108.3	HEIGHT	17.95	17	0	0
24	6159	24	Azithromycin	1	76	4/4/2017	113.7	HEIGHT	19.95	16	0	0
24	6159	36	Azithromycin	1	89	5/23/2018	120.3	HEIGHT	23.1	17	0	0
24	6159	48	Azithromycin	1	95	4/28/2019	125.2	HEIGHT	26	18	0	0
24	6159	60	Azithromycin	1	104	2/19/2020	131.3	HEIGHT	28.95	18	0	0
24	8007	36	Azithromycin	1	12	5/23/2018	73.4	HEIGHT	8.25	13	1	0
24	8007	48	Azithromycin	1	23	4/28/2019	83.5	LENGTH	10.6	13.5	1	0
24	8007	60	Azithromycin	1	32	2/19/2020	91.9	HEIGHT	13.95	15	1	0
24	8029	60	Azithromycin	0	8	2/19/2020	68.6	LENGTH	8.1	13	1	0
24	8096	60	Azithromycin	1	31	2/19/2020	84.9	HEIGHT	11.3	13.7	1	0
24	8114	60	Azithromycin	1	15	2/19/2020	76.5	HEIGHT	9.2	14	1	0
24	8126	48	Azithromycin	1	10	4/28/2019	67.2	LENGTH	8.5	16	1	0
24	8126	60	Azithromycin	1	15	2/19/2020	76.5	HEIGHT	10.65	15.5	0	0
24	8132	48	Azithromycin	1	17	4/28/2019	72.3	LENGTH	6.8	11	1	0
24	8163	60	Azithromycin	1	5	2/19/2020	66	HEIGHT	9	14.6	1	0
24	8217	48	Azithromycin	1	48	4/28/2019	99.4	HEIGHT	17.25	17	1	0

24	8230	60	Azithromycin	0	3	2/19/2020	63.8	LENGTH	7.1	13.2		1	0
24	8305	60	Azithromycin	0	15	2/19/2020	66.5	LENGTH	7.4	12.5		1	0
24	8311	36	Azithromycin	1	1	5/23/2018	56.9	LENGTH	4.7	11		1	0
24	8311	48	Azithromycin	1	12	4/28/2019	69.2	LENGTH	6.25	11		0	0
24	8316	36	Azithromycin	0	19	5/23/2018	72.2	LENGTH	7.55	13.5		1	0
24	8342	36	Azithromycin	0	15	5/23/2018	71.6	LENGTH	7.3	12.5		1	0
24	8374	36	Azithromycin	1	11	5/23/2018	71.9	LENGTH	9.3	15.5		1	0
24	8374	48	Azithromycin	1	22	4/28/2019	80	LENGTH	10.7	14.5		0	0
24	8374	60	Azithromycin	1	31	2/19/2020	87.6	HEIGHT	13.8	16.5		0	0
24	8419	36	Azithromycin	0	4	5/23/2018	61.1	LENGTH	6.05	13		1	0
24	8419	48	Azithromycin	0	12	4/28/2019	74.1	LENGTH	8.75	14		1	0
24	8447	36	Azithromycin	1	36	5/23/2018	89.3	HEIGHT	10.9	13.5		1	0
24	8455	48	Azithromycin	0	3	4/28/2019	61.4	LENGTH	6.6	14.5		1	0
24	8455	60	Azithromycin	0	12	2/19/2020	71.9	LENGTH	9.75	14.2		1	0
24	8498	60	Azithromycin	0	15	2/19/2020	70.7	LENGTH	7.4	13		1	0
24	8504	36	Azithromycin	0	19	5/23/2018	72.1	LENGTH	7.85	12.5		1	0
24	8504	48	Azithromycin	0	30	4/28/2019	79.9	LENGTH	9.8	13		1	0
24	8541	36	Azithromycin	0	10	5/23/2018	68.7	LENGTH	7.5	12.5		1	0
24	8549	48	Azithromycin	1	18	4/28/2019	74.6	LENGTH	7.5	12.5		1	0
24	8571	36	Azithromycin	0	44	5/23/2018	96.7	HEIGHT	13.15	15		1	0
24	8613	36	Azithromycin	1	4	5/23/2018	63.2	LENGTH	6.55	14		1	0
24	8613	48	Azithromycin	1	15	4/28/2019	79.2	HEIGHT	10	14.5		1	0
24	8627	60	Azithromycin	0	50	2/19/2020	98	HEIGHT	13.95	14.5		1	0
24	8629	48	Azithromycin	1	15	4/28/2019	75.1	LENGTH	7.9	12		1	0
24	8629	60	Azithromycin	1	24	2/19/2020	85.1	HEIGHT	9.35	12		1	0
24	8636	60	Azithromycin	1	5	2/19/2020	61.1	HEIGHT	6.95	13.4		1	0
24	8651	48	Azithromycin	1	7	4/28/2019	66.5	LENGTH	7.05	13		1	0
24	8658	36	Azithromycin	1	12	5/23/2018	66.3	LENGTH	7.3	13	42	1	0
24	8811	36	Azithromycin	1	6	5/23/2018	64	HEIGHT	6.85	14		1	0
24	8811	48	Azithromycin	1	15	4/28/2019	75.5	LENGTH	9.7	15		1	0
24	8811	60	Azithromycin	1	25	2/19/2020	82.9	HEIGHT	11.4	14.5		0	0
24	8823	48	Azithromycin	0	15	4/28/2019	77	LENGTH	8.4	13		1	0
24	8823	60	Azithromycin	0	25	2/19/2020	82.5	HEIGHT	9.95	13.3		1	0
24	8828	36	Azithromycin	1	9	5/23/2018	71.7	LENGTH	8.6	15.5		1	0
24	8838	36	Azithromycin	1	25	5/23/2018	81.9	HEIGHT	10.5	14		1	0
24	8840	36	Azithromycin	0	53	5/23/2018	99.3	HEIGHT	15.15	15		1	0
24	8885	48	Azithromycin	1	48	4/28/2019	97	HEIGHT	12.75	14.5		1	0
24	8885	60	Azithromycin	1	58	2/19/2020	103.5	HEIGHT	15.4	14.3		1	0
24	8892	36	Azithromycin	0	21	5/23/2018	77.4	HEIGHT	9.3	12.5		1	0
24	8892	48	Azithromycin	0	32	4/28/2019	82.9	HEIGHT	10.9	13.5		1	0

24	8907	36	Azithromycin	1	15	5/23/2018	72.5	HEIGHT	7.3	13		1	0
24	8907	48	Azithromycin	1	26	4/28/2019	78.2	HEIGHT	9	13.5		1	0
24	8907	60	Azithromycin	1	36	2/19/2020	87.1	HEIGHT	11.45	13.7		1	0
24	8908	36	Azithromycin	0	15	5/23/2018	75.5	LENGTH	9.35	13		1	0
24	8925	60	Azithromycin	0	14	2/19/2020	74.8	LENGTH	7.8	11.7		1	0
24	8966	48	Azithromycin	1	11	4/28/2019	73.3	LENGTH	8.9	14.5		1	0
24	8976	60	Azithromycin	0	7	2/19/2020	67.5	HEIGHT	8.15	13.5		1	0
24	8992	60	Azithromycin	1	33	2/19/2020	87.6	HEIGHT	12.7	15		1	0
24	9048	60	Azithromycin	0	33	2/19/2020	88.2	HEIGHT	11.9	14.4		1	0
24	9095	36	Azithromycin	1	12	5/23/2018	67.8	LENGTH	7.75	13.5	42	1	0
24	9101	36	Azithromycin	1	15	5/23/2018	74.8	HEIGHT	8.05	13		1	0
24	9183	60	Azithromycin	1	55	2/19/2020	103.8	HEIGHT	15.2	14.4		1	0
24	9216	48	Azithromycin	0	14	4/28/2019	76.5	LENGTH	9.6	15		1	0
24	9217	60	Azithromycin	0	51	2/19/2020	102.7	HEIGHT	14.8	14		1	0
24	9226	60	Azithromycin	0	10	2/19/2020	72.1	HEIGHT	8.85	13.3		1	0
24	9250	60	Azithromycin	0	25	2/19/2020	85	HEIGHT	10.95	13.4		1	0
24	9302	48	Azithromycin	1	11	4/28/2019	74.4	LENGTH	8.25	14		1	0
24	9302	60	Azithromycin	1	15	2/19/2020	83	HEIGHT	10.15	13.5		0	0
24	9319	36	Azithromycin	0	29	5/23/2018	85.7	HEIGHT	11.1	14		1	0
24	9319	48	Azithromycin	0	40	4/28/2019	91.4	HEIGHT	12.9	14		1	0
24	9358	48	Azithromycin	0	10	4/28/2019	68.7	LENGTH	6.8	12		1	0
24	9358	60	Azithromycin	0	15	2/19/2020	76.7	LENGTH	8.6	13		1	0
24	9378	60	Azithromycin	1	18	2/19/2020	80.3	HEIGHT	10.1	14.3		1	0
24	9443	36	Azithromycin	0	4	5/23/2018	59.2	LENGTH	6	13.5		1	0
24	9443	48	Azithromycin	0	15	4/28/2019	72.1	LENGTH	9.25	16		1	0
24	9443	60	Azithromycin	0	24	2/19/2020	81.7	LENGTH	10.9	15.3		1	0
24	9457	36	Azithromycin	1	6	5/23/2018	65.6	LENGTH	6.8	13		1	0
24	9457	60	Azithromycin	1	26	2/19/2020	82.5	HEIGHT	10	14		1	0
24	9552	48	Azithromycin	1	5	4/28/2019	58.2	LENGTH	5.5	12		1	0
24	9552	60	Azithromycin	1	15	2/19/2020	67.4	LENGTH	7.1	12		1	0
24	9578	60	Azithromycin	1	3	2/19/2020	61.3	LENGTH	5.85	13		1	0
25	6170	0	Azithromycin	0	30	3/13/2015	94.4	HEIGHT	13.2	15		1	0
25	6170	24	Azithromycin	0	57	5/21/2017	109.5	HEIGHT	17.95454545	14.5		1	0
25	6171	0	Azithromycin	1	36	6/16/2015	87	HEIGHT	10.5	13.5		0	0
25	6171	12	Azithromycin	1	42	6/27/2016	93.5	HEIGHT	11.8	14		0	0
25	6171	24	Azithromycin	1	56	5/21/2017	99.2	HEIGHT	13.22727273	14.5		1	0
25	6171	36	Azithromycin	1	68	6/21/2018	105	HEIGHT	14.3	13.5		0	0
25	6171	48	Azithromycin	1	79	5/16/2019	109.2	HEIGHT	15.2	14		0	0
25	6171	60	Azithromycin	1	88	2/11/2020	112.7	HEIGHT	16.9	14		0	0
25	6172	0	Azithromycin	0	54	3/13/2015	104.2	HEIGHT	17.3	16.5		0	0

25	6172	12	Azithromycin	0	78	6/27/2016	111.3	HEIGHT	19.5	16	0	0
25	6172	24	Azithromycin	0	92	5/21/2017	116.3	HEIGHT	20.31818182	16	0	0
25	6172	36	Azithromycin	0	104	6/21/2018	120	HEIGHT	23.1	16.5	0	0
25	6172	48	Azithromycin	0	115	5/16/2019	122.2	HEIGHT	24.15	17.5	0	0
25	6172	60	Azithromycin	0	124	2/11/2020	125.5	HEIGHT	25.55	17	0	0
25	6173	24	Azithromycin	0	3	5/21/2017	63	LENGTH	7.045454545	15	1	0
25	6173	36	Azithromycin	0	15	6/21/2018	78.5	LENGTH	10.8	14.5	1	0
25	6173	48	Azithromycin	0	26	5/16/2019	85.7	HEIGHT	12.85	16	1	0
25	6173	60	Azithromycin	0	35	2/11/2020	92.9	HEIGHT	15	15.5	1	0
25	6175	0	Azithromycin	0	24	3/13/2015	89.2	HEIGHT	14.3	15.5	1	0
25	6175	12	Azithromycin	0	37	6/21/2016	99.4	HEIGHT	15.6	15	1	0
25	6175	24	Azithromycin	0	51	5/21/2017	106	HEIGHT	17.86363636	15	1	0
25	6177	0	Azithromycin	1	36	6/9/2015	93.4	HEIGHT	13.95	14.5	1	0
25	6179	12	Azithromycin	1	9	6/21/2016	69.3	LENGTH	7.5	13	1	0
25	6179	24	Azithromycin	1	12	5/21/2017	80.7	HEIGHT	10.63636364	14	0	0
25	6179	36	Azithromycin	1	24	6/21/2018	88.5	HEIGHT	12.3	14	1	0
25	6179	48	Azithromycin	1	35	5/16/2019	97.2	HEIGHT	15.35	15	1	0
25	6179	60	Azithromycin	1	44	2/11/2020	104.7	HEIGHT	16.90909091	14.5	1	0
25	6184	0	Azithromycin	1	54	3/13/2015	91.7	HEIGHT	12.3	14.5	1	0
25	6185	0	Azithromycin	0	54	6/16/2015	105.2	HEIGHT	16.1	15	0	0
25	6185	12	Azithromycin	0	54	6/21/2016	112.4	HEIGHT	18.45	15.5	1	0
25	6185	24	Azithromycin	0	68	5/21/2017	118.6	HEIGHT	20.54545455	15.5	0	0
25	6188	0	Azithromycin	0	30	3/13/2015	88.3	HEIGHT	13.7	16	1	0
25	6188	24	Azithromycin	0	56	5/21/2017	106.5	HEIGHT	17.5	15	1	0
25	6188	36	Azithromycin	0	74	6/21/2018	112.4	HEIGHT	19	15.5	0	0
25	6188	48	Azithromycin	0	85	5/16/2019	119.1	HEIGHT	21.2	16	0	0
25	6188	60	Azithromycin	0	94	2/11/2020	123.2	HEIGHT	22.1	15.3	0	0
25	6191	0	Azithromycin	0	24	3/13/2015	71.6	LENGTH	8.1	14.5	0	0
25	6191	36	Azithromycin	0	63	6/21/2018	121.4	HEIGHT	21.45	14	0	0
25	6192	24	Azithromycin	0	3	5/21/2017	54.5	LENGTH	3.681818182	8.5	1	0
25	6192	36	Azithromycin	0	12	6/21/2018	68.3	LENGTH	7.15	12	1	0
25	6192	48	Azithromycin	0	22	5/16/2019	76.5	HEIGHT	9.45	13	1	0
25	6192	60	Azithromycin	0	36	3/16/2020	83.7	HEIGHT	11.6	14	1	0
25	6194	12	Azithromycin	0	1	6/21/2016	61.8	LENGTH	6.65	13.5	1	0
25	6194	24	Azithromycin	0	15	5/21/2017	74.4	HEIGHT	9.136363636	14.5	1	0
25	6194	36	Azithromycin	0	27	6/21/2018	79.4	LENGTH	9.95	13	1	0
25	6194	48	Azithromycin	0	38	5/16/2019	82.6	HEIGHT	11.2	14	1	0
25	6194	60	Azithromycin	0	47	2/11/2020	90.6	HEIGHT	12.9	14	1	0
25	6195	0	Azithromycin	1	24	3/13/2015	75.3	HEIGHT	8.35	13	1	0
25	6195	48	Azithromycin	1	67	5/16/2019	102	HEIGHT	14	14	0	0

25	6197	0	Azithromycin	0	12	3/13/2015	71.2	LENGTH	8.45	14.5	0	0
25	6197	12	Azithromycin	0	30	6/21/2016	82	HEIGHT	10.15	15	1	0
25	6197	24	Azithromycin	0	44	5/21/2017	90.6	HEIGHT	12.81818182	15	1	0
25	6197	36	Azithromycin	0	52	6/21/2018	100.3	HEIGHT	14.75	14.5	1	0
25	6197	48	Azithromycin	0	63	5/16/2019	105.1	HEIGHT	16.45	15	0	0
25	6197	60	Azithromycin	0	72	2/11/2020	110.8	HEIGHT	17.2	15	0	0
25	6198	0	Azithromycin	0	48	6/16/2015	91.9	HEIGHT	13.95	15	0	0
25	6198	12	Azithromycin	0	63	6/27/2016	98.8	HEIGHT	15.5	15.5	0	0
25	6198	24	Azithromycin	0	75	5/21/2017	105.1	HEIGHT	17	16	0	0
25	6198	36	Azithromycin	0	87	6/21/2018	111.3	HEIGHT	19.25	16	0	0
25	6198	48	Azithromycin	0	98	5/16/2019	117.1	HEIGHT	21.2	16	0	0
25	6198	60	Azithromycin	0	107	2/11/2020	120.3	HEIGHT	22.5	16.2	0	0
25	6199	12	Azithromycin	1	45	7/10/2016	96.5	LENGTH	17.45	15.9	1	0
25	6199	24	Azithromycin	1	56	5/21/2017	104.9	HEIGHT	16.90909091	16	1	0
25	6199	48	Azithromycin	1	79	5/16/2019	116	HEIGHT	19.8	16	0	0
25	6199	60	Azithromycin	1	88	2/11/2020	119.2	HEIGHT	20.75	15.5	0	0
25	6200	0	Azithromycin	0	24	6/16/2015	77.7	LENGTH	8.85	13.5	1	0
25	6201	12	Azithromycin	0	20	6/21/2016	81	HEIGHT	10.1	14.5	1	0
25	6201	36	Azithromycin	0	49	6/21/2018	94.5	HEIGHT	12.85	14	1	0
25	6201	48	Azithromycin	0	59	5/16/2019	99.3	HEIGHT	14.05	14.5	1	0
25	6201	60	Azithromycin	0	69	2/11/2020	103.7	HEIGHT	15.45454545	14.2	0	0
25	6202	24	Azithromycin	1	44	5/21/2017	97.4	HEIGHT	14.45454545	16	1	0
25	6202	36	Azithromycin	1	56	6/21/2018	104.6	HEIGHT	16.3	15	1	0
25	6202	48	Azithromycin	1	57	5/16/2019	109.9	HEIGHT	17.8	15.5	1	0
25	6204	24	Azithromycin	0	4	5/21/2017	63.8	LENGTH	6.772727273	14.5	1	0
25	6204	36	Azithromycin	0	15	6/21/2018	74.5	LENGTH	8.8	13	1	0
25	6204	48	Azithromycin	0	26	5/16/2019	78.2	LENGTH	8.95	13.5	1	0
25	6204	60	Azithromycin	0	35	3/16/2020	84.7	HEIGHT	11.55	14.5	1	0
25	6206	12	Azithromycin	1	54	7/10/2016	98.1	LENGTH	21.3	14.4	1	0
25	6206	48	Azithromycin	1	91	5/16/2019	111.5	HEIGHT	16.65	14	0	0
25	6206	60	Azithromycin	1	100	2/11/2020	114.2	HEIGHT	18.45	14.5	0	0
25	6207	0	Azithromycin	0	48	3/13/2015	106.7	HEIGHT	17.35	15.5	1	0
25	6208	0	Azithromycin	0	18	3/13/2015	78.3	HEIGHT	8.95	15	1	0
25	6208	12	Azithromycin	0	31	6/21/2016	87.1	HEIGHT	11.3	15.5	1	0
25	6208	24	Azithromycin	0	45	5/21/2017	94.6	HEIGHT	12.95454545	15.5	1	0
25	6208	36	Azithromycin	0	57	6/21/2018	100.6	HEIGHT	13.8	14	1	0
25	6208	48	Azithromycin	0	57	5/16/2019	104.3	HEIGHT	14.55	14	1	0
25	6208	60	Azithromycin	0	66	2/11/2020	105.8	HEIGHT	15.09090909	14.5	0	0
25	6211	0	Azithromycin	1	36	3/13/2015	92.2	HEIGHT	13.5	15.5	1	0
25	6211	12	Azithromycin	1	51	6/21/2016	102	HEIGHT	15.35	15	1	0

25	6211	24	Azithromycin	1	63	5/21/2017	108.2	HEIGHT	17.54545455	16		0	0
25	6211	36	Azithromycin	1	75	6/21/2018	113.6	HEIGHT	18.35	15		0	0
25	6211	48	Azithromycin	1	86	5/16/2019	116.8	HEIGHT	19.95	15.5		0	0
25	6211	60	Azithromycin	1	95	2/11/2020	118.5	HEIGHT	21.1	15		0	0
25	6217	24	Azithromycin	0	13	5/21/2017	75	LENGTH	9.272727273	14	30	1	0
25	6219	12	Azithromycin	0	50	6/21/2016	91	LENGTH	12.15	14		1	0
25	6220	0	Azithromycin	0	12	3/13/2015	71.8	LENGTH	10.45	17.5		1	0
25	6220	12	Azithromycin	0	30	6/21/2016	85.5	HEIGHT	12.45	15.5		1	0
25	6220	24	Azithromycin	0	44	5/21/2017	89.8	HEIGHT	14.81818182	16.5		1	0
25	6220	36	Azithromycin	0	56	6/21/2018	96.9	HEIGHT	15.85	16		1	0
25	6220	48	Azithromycin	0	57	5/16/2019	101.9	HEIGHT	16.75	15.5		1	0
25	6220	60	Azithromycin	0	66	2/11/2020	105.9	HEIGHT	18.9	16		0	0
25	6223	0	Azithromycin	1	24	3/13/2015	84.4	HEIGHT	11.25	14.5		0	0
25	6223	24	Azithromycin	1	51	5/21/2017	100.8	HEIGHT	14.27272727	14		1	0
25	6223	60	Azithromycin	1	83	3/16/2020	116	HEIGHT	20.25	15.7		0	0
25	6227	0	Azithromycin	0	36	3/13/2015	93.4	HEIGHT	13.75	15.5		0	0
25	6227	12	Azithromycin	0	54	6/27/2016	105	HEIGHT	16.3	15		0	0
25	6227	24	Azithromycin	0	62	5/21/2017	110.2	HEIGHT	17.13636364	14.5		0	0
25	6227	36	Azithromycin	0	74	6/21/2018	115.9	HEIGHT	19.1	13.5		0	0
25	6227	48	Azithromycin	0	85	5/16/2019	121.2	HEIGHT	20.85	15		0	0
25	6227	60	Azithromycin	0	94	2/11/2020	123.3	HEIGHT	22.5	15		0	0
25	6234	0	Azithromycin	0	12	3/13/2015	65.5	LENGTH	5.4	11		0	0
25	6234	12	Azithromycin	0	27	6/27/2016	73.7	HEIGHT	7.9	12		0	0
25	6234	24	Azithromycin	0	39	5/21/2017	81.8	HEIGHT	10.31818182	13		1	0
25	6234	36	Azithromycin	0	49	6/21/2018	91	HEIGHT	11.6	12.5		1	0
25	6234	48	Azithromycin	0	60	5/16/2019	96.8	HEIGHT	13.15	13		0	0
25	6234	60	Azithromycin	0	69	2/11/2020	101.6	HEIGHT	14.8	13		0	0
25	6235	0	Azithromycin	0	8	3/13/2015	72.5	LENGTH	8.4	13	18	1	0
25	6236	12	Azithromycin	0	12	6/21/2016	75.2	LENGTH	9.8	15		1	0
25	6236	24	Azithromycin	0	23	5/21/2017	85.3	HEIGHT	11.72727273	14.5		1	0
25	6236	36	Azithromycin	0	35	6/21/2018	93.2	HEIGHT	13.45	14.5		1	0
25	6236	48	Azithromycin	0	46	5/16/2019	100.1	HEIGHT	15.4	15		1	0
25	6236	60	Azithromycin	0	55	2/11/2020	106.3	HEIGHT	18.27272727	15.2		1	0
25	6238	0	Azithromycin	1	48	3/13/2015	103.2	HEIGHT	16.65	16.5		0	0
25	6238	12	Azithromycin	1	69	6/27/2016	112.5	HEIGHT	19.15	16.5		0	0
25	6238	24	Azithromycin	1	80	5/21/2017	121	HEIGHT	20.77272727	16.5		0	0
25	6238	36	Azithromycin	1	92	6/21/2018	126.9	HEIGHT	23.4	15.5		0	0
25	6238	48	Azithromycin	1	103	5/16/2019	131.5	HEIGHT	25.65	17		0	0
25	6238	60	Azithromycin	1	103	2/11/2020	136.8	HEIGHT	27	16.3		0	0
25	6239	12	Azithromycin	0	14	6/21/2016	79.1	LENGTH	9.5	14.5		1	0

25	6239	24	Azithromycin	0	26	5/21/2017	87.8	HEIGHT	11.72727273	15		1	0
25	6239	36	Azithromycin	0	38	6/21/2018	94.3	HEIGHT	13.75	15		1	0
25	6239	48	Azithromycin	0	49	5/16/2019	98.4	HEIGHT	14.15	14.5		1	0
25	6239	60	Azithromycin	0	58	3/16/2020	103.1	HEIGHT	15.15	13.7		1	0
25	6242	12	Azithromycin	1	42	6/21/2016	95.5	HEIGHT	12.75	14.5		1	0
25	6243	24	Azithromycin	0	1	5/21/2017	53.6	LENGTH	3.863636364	10		1	0
25	6243	36	Azithromycin	0	13	6/21/2018	72.7	LENGTH	7.65	12		1	0
25	6243	48	Azithromycin	0	24	5/16/2019	79.7	LENGTH	9.5	13		1	0
25	6243	60	Azithromycin	0	33	2/11/2020	85.9	HEIGHT	12.1	14		1	0
25	6246	0	Azithromycin	1	12	3/13/2015	68.3	LENGTH	6.2	12.5		1	0
25	6246	12	Azithromycin	1	27	6/21/2016	78	HEIGHT	8.5	13.5		1	0
25	6246	24	Azithromycin	1	39	5/21/2017	86.8	HEIGHT	10.40909091	14		1	0
25	6246	36	Azithromycin	1	47	6/21/2018	95.1	HEIGHT	12.4	14		1	0
25	6248	0	Azithromycin	0	36	3/13/2015	86	HEIGHT	13.45	16		0	0
25	6248	12	Azithromycin	0	42	6/27/2016	95.5	HEIGHT	15.4	16		0	0
25	6248	24	Azithromycin	0	56	5/21/2017	100.8	HEIGHT	16.36363636	15		1	0
25	6248	36	Azithromycin	0	77	6/21/2018	105.7	HEIGHT	16.75	14.5		0	0
25	6252	12	Azithromycin	1	6	6/27/2016	62.9	LENGTH	6.5	12.5	30	0	0
25	6252	24	Azithromycin	1	12	5/21/2017	74.8	HEIGHT	9.772727273	15	30	1	0
25	6253	0	Azithromycin	1	10	3/13/2015	69.5	LENGTH	8.1	13.5		1	0
25	6253	12	Azithromycin	1	18	6/27/2016	85.6	HEIGHT	11.4	15		0	0
25	6254	0	Azithromycin	1	48	3/13/2015	113.4	HEIGHT	19.75	16.5		1	0
25	6254	12	Azithromycin	1	78	6/27/2016	120.1	HEIGHT	23.7	18		0	0
25	6254	36	Azithromycin	1	107	6/21/2018	130.5	HEIGHT	26.05	17.5		0	0
25	6254	48	Azithromycin	1	118	5/16/2019	131.1	HEIGHT	28.2	18		0	0
25	6254	60	Azithromycin	1	128	2/11/2020	138.4	HEIGHT	29.45	18		0	0
25	6256	12	Azithromycin	1	49	7/10/2016	96.6	LENGTH	22.1	16.3		1	0
25	6256	48	Azithromycin	1	86	5/16/2019	112.6	HEIGHT	20.7	15.5		0	0
25	6256	60	Azithromycin	1	95	2/11/2020	118.2	HEIGHT	21.75	15.5		0	0
25	6259	0	Azithromycin	1	54	6/16/2015	88.3	HEIGHT	10.85	14		0	0
25	6260	12	Azithromycin	0	12	6/21/2016	75.4	HEIGHT	9.25	15		1	0
25	6260	24	Azithromycin	0	26	5/21/2017	82.3	HEIGHT	10.90909091	15		1	0
25	6260	48	Azithromycin	0	49	5/16/2019	95.9	HEIGHT	14.45	15		1	0
25	6260	60	Azithromycin	0	58	3/16/2020	100.9	HEIGHT	15.35	15.3		1	0
25	6262	0	Azithromycin	1	48	3/13/2015	102.3	HEIGHT	16.75	16.5		1	0
25	6262	12	Azithromycin	1	63	6/27/2016	110.9	HEIGHT	19.35	17		0	0
25	6262	24	Azithromycin	1	75	5/21/2017	117.7	HEIGHT	19.68181818	16		0	0
25	6262	60	Azithromycin	1	107	2/11/2020	124.9	HEIGHT	25.4	17.5		0	0
25	6265	0	Azithromycin	1	12	6/9/2015	73.1	LENGTH	7.4	13		1	0
25	6266	0	Azithromycin	0	36	3/13/2015	87.4	HEIGHT	13.35	17		0	0

25	6266	12	Azithromycin	0	51	6/21/2016	96.9	HEIGHT	14.9	16.5	1	0
25	6266	24	Azithromycin	0	63	5/21/2017	102	HEIGHT	15.86363636	15	0	0
25	6266	48	Azithromycin	0	86	5/16/2019	110.4	HEIGHT	18.15	15.5	0	0
25	6266	60	Azithromycin	0	95	2/11/2020	114.7	HEIGHT	20.59090909	15	0	0
25	6268	0	Azithromycin	0	36	3/13/2015	98.9	HEIGHT	15.75	15.5	1	0
25	6268	12	Azithromycin	0	49	6/21/2016	107.1	HEIGHT	18.1	15	1	0
25	6273	0	Azithromycin	0	48	3/13/2015	105.8	HEIGHT	17	15.5	1	0
25	6273	12	Azithromycin	0	66	6/27/2016	114.5	HEIGHT	19.45	16	0	0
25	6273	24	Azithromycin	0	80	5/21/2017	119.2	HEIGHT	20.86363636	16	0	0
25	6273	36	Azithromycin	0	105	6/21/2018	122.4	HEIGHT	22.3	16.5	0	0
25	6273	48	Azithromycin	0	116	5/16/2019	126.1	HEIGHT	25.65	17	0	0
25	6273	60	Azithromycin	0	125	2/11/2020	128.8	HEIGHT	26.05	16.5	0	0
25	6275	0	Azithromycin	0	7	3/13/2015	63.3	LENGTH	6.6	13.5	1	0
25	6275	12	Azithromycin	0	18	6/27/2016	76.4	HEIGHT	10.3	14	0	0
25	6275	24	Azithromycin	0	32	5/21/2017	86.1	HEIGHT	13.5	14.5	1	0
25	6275	60	Azithromycin	0	64	3/16/2020	108.8	HEIGHT	19.35	14.5	0	0
25	6277	12	Azithromycin	0	57	6/21/2016	102	HEIGHT	15.9	15.5	1	0
25	6277	60	Azithromycin	0	100	3/16/2020	118.7	HEIGHT	22.55	16.5	0	0
25	6278	0	Azithromycin	1	2	6/16/2015	68.1	LENGTH	6.9	13	0	0
25	6278	12	Azithromycin	1	9	6/21/2016	78.6	LENGTH	8.9	13	1	0
25	6278	24	Azithromycin	1	23	5/21/2017	89.4	HEIGHT	10.86363636	13.5	0	0
25	6278	48	Azithromycin	1	52	5/16/2019	107.1	HEIGHT	14.35	13.5	1	0
25	6280	0	Azithromycin	0	48	3/13/2015	96.3	HEIGHT	15.75	15.5	1	0
25	6280	12	Azithromycin	0	66	6/27/2016	104.6	HEIGHT	16.35	16	0	0
25	6280	24	Azithromycin	0	80	5/21/2017	110.4	HEIGHT	17.45454545	15.5	0	0
25	6280	36	Azithromycin	0	92	6/21/2018	115.7	HEIGHT	18.95	15.5	0	0
25	6280	48	Azithromycin	0	103	5/16/2019	119.8	HEIGHT	21.75	16	0	0
25	6280	60	Azithromycin	0	112	2/11/2020	123.3	HEIGHT	22.35	15	0	0
25	6281	12	Azithromycin	0	36	6/21/2016	90	HEIGHT	11.9	14	1	0
25	6281	24	Azithromycin	0	47	5/21/2017	98.5	HEIGHT	13.45454545	14.5	1	0
25	6281	36	Azithromycin	0	59	6/21/2018	104.4	HEIGHT	14.75	12.5	1	0
25	6281	48	Azithromycin	0	70	5/16/2019	110	HEIGHT	16.25	14.5	0	0
25	6281	60	Azithromycin	0	79	2/11/2020	112.6	HEIGHT	17.95454545	14.2	0	0
25	6282	0	Azithromycin	1	18	3/13/2015	75.4	HEIGHT	8.85	13.5	1	0
25	6282	12	Azithromycin	1	31	6/27/2016	84.6	HEIGHT	10.85	14	0	0
25	6282	36	Azithromycin	1	57	6/21/2018	95.4	HEIGHT	13.55	14	1	0
25	6282	48	Azithromycin	1	71	5/16/2019	100.3	HEIGHT	14.25	14	0	0
25	6283	0	Azithromycin	1	54	6/9/2015	108.7	HEIGHT	18.48	16	1	0
25	6285	12	Azithromycin	1	54	6/21/2016	103.7	HEIGHT	14.85	15	1	0
25	6285	48	Azithromycin	1	112	5/16/2019	116.8	HEIGHT	19.35	15	0	0

25	6285	60	Azithromycin	1	121	2/11/2020	119.9	HEIGHT	25.3	15.5		0	0
25	6290	0	Azithromycin	0	36	6/16/2015	89.6	HEIGHT	12.15	14	12	0	0
25	6293	0	Azithromycin	0	30	3/13/2015	96.2	HEIGHT	12.95	14.5		0	0
25	6293	12	Azithromycin	0	43	6/21/2016	104	HEIGHT	15	14		1	0
25	6293	24	Azithromycin	0	57	5/21/2017	110.5	HEIGHT	16.18181818	13.5		1	0
25	6293	36	Azithromycin	0	87	6/21/2018	119.5	HEIGHT	18.1	13.5		0	0
25	6293	48	Azithromycin	0	98	5/16/2019	122.9	HEIGHT	19.45	14		0	0
25	6293	60	Azithromycin	0	107	2/11/2020	126.8	HEIGHT	22.40909091	14.5		0	0
25	6295	0	Azithromycin	0	36	3/13/2015	90.4	HEIGHT	12.75	15		0	0
25	6295	12	Azithromycin	0	51	6/27/2016	100.2	HEIGHT	14.45	14		0	0
25	6295	24	Azithromycin	0	63	5/21/2017	106.4	HEIGHT	16.22727273	14.5		0	0
25	6295	48	Azithromycin	0	86	5/16/2019	114.9	HEIGHT	18.85	14		0	0
25	6295	60	Azithromycin	0	95	2/11/2020	118.4	HEIGHT	20	14.6		0	0
25	6297	24	Azithromycin	1	12	5/21/2017	70.6	HEIGHT	7.681818182	13		1	0
25	6297	48	Azithromycin	1	35	5/16/2019	84.2	HEIGHT	10.5	13.5		1	0
25	6297	60	Azithromycin	1	44	2/11/2020	90.9	HEIGHT	13.2	14.3		0	0
25	6298	0	Azithromycin	0	36	3/13/2015	81.7	HEIGHT	10.25	14		1	0
25	6302	12	Azithromycin	0	1	6/27/2016	63.5	LENGTH	5.95	13.5	18	0	0
25	6303	0	Azithromycin	1	48	6/9/2015	104.8	HEIGHT	17.5	16		1	0
25	6304	0	Azithromycin	0	48	6/16/2015	99.9	HEIGHT	15.65	14.5		0	0
25	6304	24	Azithromycin	0	56	5/21/2017	113.8	HEIGHT	18.90909091	14		1	0
25	6304	36	Azithromycin	0	68	6/21/2018	118.6	HEIGHT	20.55	14.5		0	0
25	6304	48	Azithromycin	0	79	5/16/2019	122.3	HEIGHT	21.8	15		0	0
25	6304	60	Azithromycin	0	88	2/11/2020	126.1	HEIGHT	24.9	15		0	0
25	6305	0	Azithromycin	0	24	6/16/2015	79.1	LENGTH	9.3	13.5		1	0
25	6308	12	Azithromycin	1	54	6/21/2016	103.3	HEIGHT	15.6	15.5		1	0
25	6308	60	Azithromycin	1	100	2/11/2020	120.3	HEIGHT	20.3	15.9		0	0
25	6312	12	Azithromycin	1	4	6/21/2016	61.2	HEIGHT	5.8	13		1	0
25	6312	24	Azithromycin	1	12	5/21/2017	70.2	HEIGHT	7.318181818	12.5		1	0
25	6312	48	Azithromycin	1	38	5/16/2019	89.5	HEIGHT	11.85	15		1	0
25	6312	60	Azithromycin	1	47	2/11/2020	92.9	HEIGHT	13.5	15		1	0
25	6318	0	Azithromycin	1	24	6/16/2015	82.6	LENGTH	11.35	15		1	0
25	6318	12	Azithromycin	1	45	6/21/2016	91.6	HEIGHT	13.15	15.5		1	0
25	6318	24	Azithromycin	1	56	5/21/2017	98.4	HEIGHT	14.36363636	15.5		1	0
25	6318	36	Azithromycin	1	77	6/21/2018	104.2	HEIGHT	14.9	14		0	0
25	6318	48	Azithromycin	1	87	5/16/2019	108.3	HEIGHT	16.8	14		0	0
25	6318	60	Azithromycin	1	97	2/11/2020	112	HEIGHT	18.31818182	14.5		0	0
25	6323	0	Azithromycin	1	54	6/16/2015	96.7	HEIGHT	13.75	15		0	0
25	6323	12	Azithromycin	1	67	6/27/2016	103.2	HEIGHT	15.5	15.5		0	0
25	6323	24	Azithromycin	1	81	5/21/2017	109.3	HEIGHT	17.40909091	15		0	0

25	6323	36	Azithromycin	1	93	6/21/2018	114	HEIGHT	18.1	15	0	0
25	6323	48	Azithromycin	1	104	5/16/2019	118	HEIGHT	19.4	15	0	0
25	6323	60	Azithromycin	1	113	2/11/2020	121.9	HEIGHT	22	15.6	0	0
25	6324	12	Azithromycin	1	42	6/21/2016	99.4	HEIGHT	14.55	15	1	0
25	6324	24	Azithromycin	1	56	5/21/2017	107.2	HEIGHT	16.54545455	15	1	0
25	6324	48	Azithromycin	1	79	5/16/2019	118.3	HEIGHT	20.3	15	0	0
25	6324	60	Azithromycin	1	88	2/11/2020	121.1	HEIGHT	21.95454545	15.6	0	0
25	6326	0	Azithromycin	1	48	3/13/2015	111.2	HEIGHT	17.2	15	1	0
25	6326	12	Azithromycin	1	61	6/27/2016	119.2	HEIGHT	20.15	15	0	0
25	6326	24	Azithromycin	1	75	5/21/2017	125.2	HEIGHT	21.63636364	15	0	0
25	6326	36	Azithromycin	1	87	6/21/2018	129.2	HEIGHT	23.4	15	0	0
25	6326	48	Azithromycin	1	98	5/16/2019	132.5	HEIGHT	24.8	15.5	0	0
25	6326	60	Azithromycin	1	107	3/16/2020	137.1	HEIGHT	27.65	16.6	0	0
25	6328	0	Azithromycin	0	48	3/13/2015	104.7	HEIGHT	18.45	14	1	0
25	6328	12	Azithromycin	0	61	6/27/2016	122.3	HEIGHT	21.7	14	0	0
25	6328	24	Azithromycin	0	75	5/21/2017	126.7	HEIGHT	22.95454545	14.5	0	0
25	6328	36	Azithromycin	0	87	6/21/2018	130	HEIGHT	24.85	15	0	0
25	6328	48	Azithromycin	0	98	5/16/2019	133.6	HEIGHT	25.55	15	0	0
25	6328	60	Azithromycin	0	107	2/11/2020	137.8	HEIGHT	27	15	0	0
25	6329	0	Azithromycin	0	36	3/13/2015	92.9	HEIGHT	13.45	15	0	0
25	6329	24	Azithromycin	0	63	5/21/2017	113.2	HEIGHT	18.5	15	0	0
25	6329	36	Azithromycin	0	75	6/21/2018	119.5	HEIGHT	21.2	16	0	0
25	6329	48	Azithromycin	0	86	5/16/2019	125	HEIGHT	23.25	16	0	0
25	6333	0	Azithromycin	1	48	3/13/2015	102.2	HEIGHT	14.35	14.5	1	0
25	6333	12	Azithromycin	1	66	6/27/2016	110.8	HEIGHT	15.85	14.5	0	0
25	6333	24	Azithromycin	1	80	5/21/2017	115.3	HEIGHT	17.45454545	14.5	0	0
25	6333	36	Azithromycin	1	92	6/21/2018	119.5	HEIGHT	19.5	15	0	0
25	6333	48	Azithromycin	1	103	5/16/2019	125.2	HEIGHT	21.6	15.5	0	0
25	6333	60	Azithromycin	1	112	2/11/2020	128.6	HEIGHT	23.68181818	15.5	0	0
25	6334	0	Azithromycin	0	36	3/13/2015	91.5	HEIGHT	12.6	15	1	0
25	6334	36	Azithromycin	0	80	6/21/2018	106.9	HEIGHT	16.8	14.5	0	0
25	6334	48	Azithromycin	0	91	5/16/2019	110.3	HEIGHT	18.05	15	0	0
25	6337	0	Azithromycin	1	36	3/13/2015	101.7	HEIGHT	13.8	14	0	0
25	6337	12	Azithromycin	1	49	6/27/2016	112.7	HEIGHT	16.35	14	0	0
25	6337	24	Azithromycin	1	63	5/21/2017	120.2	HEIGHT	18	13.5	0	0
25	6337	36	Azithromycin	1	75	6/21/2018	126	HEIGHT	21.05	14.5	0	0
25	6337	48	Azithromycin	1	86	5/16/2019	130.6	HEIGHT	22.2	15	0	0
25	6337	60	Azithromycin	1	95	2/11/2020	136.1	HEIGHT	24.85	14.6	0	0
25	6339	0	Azithromycin	0	12	3/13/2015	71.2	HEIGHT	7.95	14	1	0
25	6339	12	Azithromycin	0	18	6/21/2016	82.4	HEIGHT	10.15	15	1	0

25	6339	24	Azithromycin	0	32	5/21/2017	91.1	HEIGHT	12.31818182	15	1	0
25	6339	36	Azithromycin	0	44	6/21/2018	98.8	HEIGHT	14.2	15	1	0
25	6339	48	Azithromycin	0	55	5/16/2019	103.4	HEIGHT	15	14	1	0
25	6339	60	Azithromycin	0	64	2/11/2020	107.3	HEIGHT	16.05	14	0	0
25	6341	0	Azithromycin	1	48	3/13/2015	93.1	HEIGHT	14.75	16	1	0
25	6341	12	Azithromycin	1	54	6/27/2016	103.2	HEIGHT	15.85	16	0	0
25	6341	36	Azithromycin	1	80	6/21/2018	119.4	HEIGHT	19.55	15	0	0
25	6341	48	Azithromycin	1	91	5/16/2019	124.6	HEIGHT	20.35	15	0	0
25	6341	60	Azithromycin	1	100	2/11/2020	128.5	HEIGHT	21.65	14.4	0	0
25	6342	0	Azithromycin	1	48	3/13/2015	100.1	HEIGHT	16.15	16	1	0
25	6342	12	Azithromycin	1	69	6/27/2016	109.2	HEIGHT	18.5	16	0	0
25	6342	24	Azithromycin	1	70	5/21/2017	116.6	HEIGHT	20.09090909	16.5	0	0
25	6342	36	Azithromycin	1	82	6/21/2018	121.4	HEIGHT	22.4	16	0	0
25	6345	0	Azithromycin	1	48	3/13/2015	91.9	HEIGHT	13.2	14	1	0
25	6345	12	Azithromycin	1	54	6/27/2016	100.4	HEIGHT	14.85	14.5	0	0
25	6345	24	Azithromycin	1	68	5/21/2017	106.6	HEIGHT	16.54545455	14.5	0	0
25	6345	36	Azithromycin	1	85	6/21/2018	113.3	HEIGHT	18.4	14.5	0	0
25	6346	0	Azithromycin	1	36	3/13/2015	87.6	HEIGHT	13.4	15.5	0	0
25	6346	12	Azithromycin	1	49	6/27/2016	97.6	HEIGHT	15.25	15	0	0
25	6347	0	Azithromycin	0	18	6/9/2015	83.7	HEIGHT	11.95	16	0	0
25	6347	12	Azithromycin	0	31	6/21/2016	92.4	HEIGHT	14.15	16.5	1	0
25	6347	36	Azithromycin	0	57	6/21/2018	107.2	HEIGHT	17.6	16	1	0
25	6347	48	Azithromycin	0	68	5/16/2019	112.4	HEIGHT	19.8	16	0	0
25	6347	60	Azithromycin	0	77	2/11/2020	116.5	HEIGHT	20.65	15.5	0	0
25	6349	0	Azithromycin	0	24	3/13/2015	77	HEIGHT	9.05	13	1	0
25	6349	12	Azithromycin	1	30	6/27/2016	89.2	HEIGHT	11.95	14.5	0	0
25	6349	24	Azithromycin	1	44	5/21/2017	96.3	HEIGHT	14.22727273	15	1	0
25	6351	0	Azithromycin	0	18	3/13/2015	81.6	HEIGHT	11.85	16.5	0	0
25	6351	12	Azithromycin	0	30	6/21/2016	89.6	HEIGHT	14.75	17	1	0
25	6351	36	Azithromycin	0	56	6/21/2018	104.5	HEIGHT	17.95	16	1	0
25	6351	48	Azithromycin	0	57	5/16/2019	108.6	HEIGHT	19.25	17	1	0
25	6351	60	Azithromycin	0	66	2/11/2020	112	HEIGHT	20.25	15.7	0	0
25	6353	0	Azithromycin	1	48	3/13/2015	83.4	HEIGHT	9.65	13.5	1	0
25	6353	12	Azithromycin	1	42	6/21/2016	94.9	HEIGHT	12.2	14.5	1	0
25	6353	24	Azithromycin	1	56	5/21/2017	100.6	HEIGHT	13.18181818	14	1	0
25	6353	36	Azithromycin	1	68	6/21/2018	106.6	HEIGHT	14.65	14	0	0
25	6353	60	Azithromycin	1	88	2/11/2020	114.3	HEIGHT	16.45	13.3	0	0
25	6354	0	Azithromycin	0	18	3/13/2015	72	HEIGHT	8.85	15	1	0
25	6355	0	Azithromycin	0	42	3/13/2015	87.3	HEIGHT	12.15	16	1	0
25	6355	12	Azithromycin	0	57	6/21/2016	93.2	HEIGHT	13.45	15.5	1	0

25	6355	24	Azithromycin	0	69	5/21/2017	98.9	HEIGHT	14.40909091	15		0	0
25	6355	48	Azithromycin	0	92	5/16/2019	106.4	HEIGHT	16.95	15		0	0
25	6355	60	Azithromycin	0	101	2/11/2020	109.8	HEIGHT	18.22727273	15.2		0	0
25	6356	0	Azithromycin	1	3	3/13/2015	58.3	LENGTH	4.45	10.5		1	0
25	6356	12	Azithromycin	1	12	6/21/2016	75.5	HEIGHT	9.35	15		1	0
25	6356	24	Azithromycin	1	26	5/21/2017	83.6	HEIGHT	11.22727273	15		1	0
25	6356	36	Azithromycin	1	43	6/21/2018	90.7	HEIGHT	12.4	15		1	0
25	6356	48	Azithromycin	1	54	5/16/2019	94.5	HEIGHT	13.25	15		1	0
25	6356	60	Azithromycin	1	63	2/11/2020	100.3	HEIGHT	15.05	14.8		0	0
25	6357	24	Azithromycin	0	3	5/21/2017	61.8	LENGTH	6	14	60	1	0
25	6357	36	Azithromycin	0	15	6/21/2018	73.9	LENGTH	8.6	16	60	1	0
25	6360	24	Azithromycin	0	26	5/21/2017	81.7	HEIGHT	10.45454545	13.5		1	0
25	6360	36	Azithromycin	0	38	6/21/2018	88	HEIGHT	12.5	14		1	0
25	6360	48	Azithromycin	0	49	5/16/2019	94.6	HEIGHT	14.15	15		1	0
25	6360	60	Azithromycin	0	58	2/11/2020	102.2	HEIGHT	15.72727273	14.9		1	0
25	6363	0	Azithromycin	1	36	3/13/2015	99	HEIGHT	16.35	17		1	0
25	6364	12	Azithromycin	0	51	6/21/2016	95.8	HEIGHT	13.5	14.5		1	0
25	6364	24	Azithromycin	0	63	5/21/2017	101.9	HEIGHT	15.27272727	13.5		0	0
25	6364	36	Azithromycin	0	75	6/21/2018	109.5	HEIGHT	16.75	13.5		0	0
25	6364	48	Azithromycin	0	86	5/16/2019	113.2	HEIGHT	16.95	14		0	0
25	6364	60	Azithromycin	0	95	2/11/2020	116.5	HEIGHT	18.8	13.5		0	0
25	6365	0	Azithromycin	0	54	3/13/2015	102	HEIGHT	16.05	16		1	0
25	6365	12	Azithromycin	0	67	6/27/2016	108.1	HEIGHT	18.5	15.5		0	0
25	6365	36	Azithromycin	0	93	6/21/2018	117.5	HEIGHT	21.6	16		0	0
25	6366	0	Azithromycin	1	36	3/13/2015	92.3	HEIGHT	12.1	14		1	0
25	6366	12	Azithromycin	1	54	6/27/2016	101.8	HEIGHT	13.45	14.5		0	0
25	6366	24	Azithromycin	1	72	5/21/2017	105.7	HEIGHT	14.31818182	14		0	0
25	6366	36	Azithromycin	1	84	6/21/2018	110.9	HEIGHT	16.15	14		0	0
25	6366	48	Azithromycin	1	95	5/16/2019	115.4	HEIGHT	17.4	14		0	0
25	6366	60	Azithromycin	1	104	2/11/2020	118.3	HEIGHT	18.9	14.6		0	0
25	6367	24	Azithromycin	1	23	5/21/2017	81.1	HEIGHT	10.72727273	15		1	0
25	6367	60	Azithromycin	1	55	3/16/2020	98.1	HEIGHT	15.75	15.5		1	0
25	6368	0	Azithromycin	1	48	6/16/2015	98.6	HEIGHT	13.55	14.5		0	0
25	6368	12	Azithromycin	1	54	6/21/2016	106	HEIGHT	15.7	15		1	0
25	6368	36	Azithromycin	1	80	6/21/2018	117.3	HEIGHT	19.8	15.5		0	0
25	6368	48	Azithromycin	1	91	5/16/2019	121.6	HEIGHT	22	16		0	0
25	6368	60	Azithromycin	1	100	2/11/2020	124.4	HEIGHT	22.55	15.5		0	0
25	6371	0	Azithromycin	1	12	6/16/2015	73.6	HEIGHT	7.85	14		0	0
25	6371	12	Azithromycin	1	30	6/21/2016	78.8	HEIGHT	9.35	14		1	0
25	6371	24	Azithromycin	1	44	5/21/2017	88.3	HEIGHT	11.13636364	14.5		1	0

25	6371	36	Azithromycin	1	56	6/21/2018	96	HEIGHT	13.35	14.5		1	0
25	6371	48	Azithromycin	1	57	5/16/2019	102.3	HEIGHT	14.55	14.5		1	0
25	6371	60	Azithromycin	1	66	2/11/2020	107.6	HEIGHT	15.95	14.4		0	0
25	6372	24	Azithromycin	1	44	5/21/2017	88.7	HEIGHT	12.22727273	15		1	0
25	6372	60	Azithromycin	1	60	2/11/2020	104.7	HEIGHT	15.36363636	13.6		0	0
25	8069	36	Azithromycin	0	10	6/21/2018	71.5	LENGTH	7.35	12		1	0
25	8069	48	Azithromycin	0	16	5/16/2019	82.3	HEIGHT	8.9	13		1	0
25	8069	60	Azithromycin	0	25	3/16/2020	86.7	HEIGHT	9.65	11.7		1	0
25	8118	60	Azithromycin	1	5	2/11/2020	60.4	LENGTH	5.863636364	11.7		1	0
25	8125	36	Azithromycin	1	21	6/21/2018	75	HEIGHT	9.4	14		1	0
25	8125	60	Azithromycin	1	41	3/16/2020	89.9	HEIGHT	13.55	15.2		1	0
25	8189	60	Azithromycin	1	25	2/11/2020	76.8	HEIGHT	9.136363636	12.5		1	0
25	8287	60	Azithromycin	0	42	2/11/2020	85.7	HEIGHT	11.04545455	12.2		1	0
25	8291	36	Azithromycin	1	9	6/21/2018	75.5	LENGTH	9.5	15		1	0
25	8291	48	Azithromycin	1	20	5/16/2019	81.8	LENGTH	11.3	14		1	0
25	8291	60	Azithromycin	1	30	2/11/2020	87.7	HEIGHT	14.2	14.7		1	0
25	8318	60	Azithromycin	0	5	3/16/2020	69.7	LENGTH	8.65	15		1	0
25	8320	48	Azithromycin	1	6	5/16/2019	66.5	LENGTH	7.65	14		1	0
25	8337	60	Azithromycin	1	43	3/22/2020	89.8	HEIGHT	13.13636364	14.7		1	0
25	8399	36	Azithromycin	1	56	6/21/2018	94.6	HEIGHT	13.95	15		1	0
25	8399	48	Azithromycin	1	60	5/16/2019	101.4	HEIGHT	15.3	14.5		0	0
25	8399	60	Azithromycin	1	69	3/16/2020	108.2	HEIGHT	17.3	15.4		0	0
25	8454	36	Azithromycin	1	15	6/21/2018	76.2	LENGTH	9.55	12.5		1	0
25	8454	48	Azithromycin	1	29	5/16/2019	80.7	HEIGHT	9.9	12.5		1	0
25	8454	60	Azithromycin	1	39	3/16/2020	89.4	HEIGHT	12.55	14		1	0
25	8470	60	Azithromycin	1	12	2/11/2020	75.9	LENGTH	8.9	13		1	0
25	8494	36	Azithromycin	1	10	6/21/2018	72.7	LENGTH	7.8	12	48	1	0
25	8527	60	Azithromycin	0	12	2/11/2020	78.1	LENGTH	11.59090909	16		1	0
25	8649	60	Azithromycin	1	12	2/11/2020	69.1	LENGTH	8.5	13.4		1	0
25	8680	36	Azithromycin	1	6	6/21/2018	60.5	LENGTH	5.55	11		1	0
25	8680	48	Azithromycin	1	17	5/16/2019	70.3	LENGTH	7.95	13		1	0
25	8680	60	Azithromycin	1	26	3/16/2020	76.3	HEIGHT	9.9	12.5		1	0
25	8686	36	Azithromycin	1	15	6/21/2018	73.1	HEIGHT	9.2	14.5		1	0
25	8746	48	Azithromycin	1	32	5/16/2019	82.6	HEIGHT	10.5	14		1	0
25	8746	60	Azithromycin	1	41	3/16/2020	91.2	HEIGHT	13.55	15.7		1	0
25	8767	48	Azithromycin	1	12	5/16/2019	70.5	LENGTH	6.7	11.5		1	0
25	8767	60	Azithromycin	1	21	2/11/2020	76.6	LENGTH	9.9	13.9		1	0
25	8790	36	Azithromycin	1	1	6/21/2018	53.5	LENGTH	3.45	10		1	0
25	8790	48	Azithromycin	1	12	5/16/2019	70.7	LENGTH	6.7	12.5		1	0
25	8790	60	Azithromycin	1	21	2/11/2020	77.4	HEIGHT	7.85	12.3		1	0

25	8794	36	Azithromycin	1	1	6/21/2018	55.8	LENGTH	5.2	12		1	0
25	8794	48	Azithromycin	1	12	5/16/2019	68.8	LENGTH	7.65	14		1	0
25	8794	60	Azithromycin	1	21	2/11/2020	74.2	HEIGHT	8.5	12.6		1	0
25	8820	60	Azithromycin	1	54	2/11/2020	103.5	HEIGHT	15	14		1	0
25	8855	36	Azithromycin	0	2	6/21/2018	58.7	LENGTH	5.55	12		1	0
25	8855	48	Azithromycin	0	13	5/16/2019	69.7	LENGTH	7.65	13		1	0
25	8855	60	Azithromycin	0	22	2/11/2020	77.6	HEIGHT	9	13		1	0
25	8903	36	Azithromycin	0	17	6/21/2018	79.4	HEIGHT	10.65	14.5	48	1	0
25	8914	60	Azithromycin	0	42	3/16/2020	97.1	HEIGHT	14.15	13.9		1	0
25	8981	36	Azithromycin	1	27	6/21/2018	81.8	HEIGHT	10.15	12.5		1	0
25	8981	48	Azithromycin	1	38	5/16/2019	89.2	HEIGHT	11.55	13		1	0
25	9172	48	Azithromycin	0	40	5/16/2019	86.2	HEIGHT	11.1	13.5		1	0
25	9172	60	Azithromycin	0	49	2/11/2020	91.1	HEIGHT	13.36363636	14.1		1	0
25	9174	36	Azithromycin	0	15	6/21/2018	79.5	LENGTH	11.1	14.5	42	1	0
25	9243	48	Azithromycin	1	48	5/16/2019	110	HEIGHT	18.05	15		1	0
25	9243	60	Azithromycin	1	57	2/11/2020	114.2	HEIGHT	20.2	15.5		1	0
25	9249	36	Azithromycin	1	8	6/21/2018	71.3	LENGTH	8.35	14		1	0
25	9249	48	Azithromycin	1	18	5/16/2019	81.7	HEIGHT	9.65	13.5		1	0
25	9249	60	Azithromycin	1	28	2/11/2020	83.5	HEIGHT	8.7	11.2		1	0
25	9331	36	Azithromycin	1	12	6/21/2018	71.4	LENGTH	7.5	13		1	0
25	9331	48	Azithromycin	1	23	5/16/2019	79.1	HEIGHT	9.4	14		1	0
25	9331	60	Azithromycin	1	33	2/11/2020	84.1	HEIGHT	11.18181818	14.2		1	0
25	9352	36	Azithromycin	0	11	6/21/2018	69.9	LENGTH	7.75	12.5		1	0
25	9371	48	Azithromycin	1	3	5/16/2019	85.6	HEIGHT	10.35	13		1	0
25	9371	60	Azithromycin	1	45	2/11/2020	91.7	HEIGHT	12.15	13.5		1	0
25	9509	36	Azithromycin	1	10	6/21/2018	68.7	LENGTH	7.3	12		1	0
25	9509	48	Azithromycin	1	21	5/16/2019	76	HEIGHT	9.75	14		1	0
25	9509	60	Azithromycin	1	31	3/22/2020	82.9	HEIGHT	11.04545455	13.5		1	0
25	9536	48	Azithromycin	0	4	5/16/2019	67.2	LENGTH	7.8	14		1	0
25	9536	60	Azithromycin	0	12	2/11/2020	77.8	LENGTH	10.36363636	14.5		1	0
26	6383	24	Azithromycin	0	1	5/17/2017	57.1	LENGTH	5.7	13.5		1	0
26	6383	60	Azithromycin	0	34	2/12/2020	89.4	HEIGHT	12.77272727	15		0	0
26	6384	24	Azithromycin	0	52	5/17/2017	94.1	HEIGHT	12.05	14.5		1	0
26	6384	48	Azithromycin	0	76	5/19/2019	103.8	HEIGHT	14.55	13.5		0	0
26	6386	24	Azithromycin	0	22	5/20/2017	79.5	HEIGHT	9.590909091	13.5	60	1	0
26	6386	36	Azithromycin	0	38	6/1/2018	88.4	HEIGHT	11.7	14.5	60	1	0
26	6391	24	Azithromycin	0	5	5/17/2017	63.9	LENGTH	7.2	14.5		1	0
26	6391	36	Azithromycin	0	15	6/1/2018	75.6	LENGTH	8.7	13.5		1	0
26	6391	48	Azithromycin	0	26	5/19/2019	81.9	HEIGHT	9.95	14		1	0
26	6391	60	Azithromycin	0	35	2/12/2020	87.3	HEIGHT	11.45454545	13.5		1	0

26	6398	0	Azithromycin	0	7	3/16/2015	69.8	LENGTH	8.25	14.5		1	0
26	6399	0	Azithromycin	1	54	3/16/2015	109.1	HEIGHT	18.2	16.5		1	0
26	6399	36	Azithromycin	1	104	6/1/2018	127.1	HEIGHT	24.75	16.5		0	0
26	6399	48	Azithromycin	1	115	5/19/2019	131.3	HEIGHT	27.95	18		0	0
26	6399	60	Azithromycin	1	124	2/12/2020	136.7	HEIGHT	28.36363636	16.8		0	0
26	6404	0	Azithromycin	0	36	3/16/2015	80.4	HEIGHT	8.95	13.5		1	0
26	6404	12	Azithromycin	0	41	2/12/2016	86.1	HEIGHT	10.8	13.5		1	0
26	6404	24	Azithromycin	0	56	5/17/2017	95.8	HEIGHT	12.05	13.5		1	0
26	6404	36	Azithromycin	0	69	6/1/2018	102.5	HEIGHT	12.6	13		0	0
26	6404	48	Azithromycin	0	80	5/19/2019	107.7	HEIGHT	15	13.5		0	0
26	6404	60	Azithromycin	0	89	2/12/2020	111.3	HEIGHT	16.40909091	13.9		0	0
26	6406	12	Azithromycin	0	1	2/11/2016	48.3	LENGTH	2.6	9		1	0
26	6406	24	Azithromycin	0	15	5/17/2017	71.3	HEIGHT	8.05	13.5		1	0
26	6406	36	Azithromycin	0	28	6/1/2018	83.9	HEIGHT	10.4	14.5		1	0
26	6406	48	Azithromycin	0	36	5/19/2019	91.5	HEIGHT	12.55	15		1	0
26	6406	60	Azithromycin	0	45	2/12/2020	97.8	HEIGHT	14.45454545	14.7		1	0
26	6410	24	Azithromycin	0	6	5/17/2017	63.7	LENGTH	5.85	13.5		1	0
26	6410	36	Azithromycin	0	19	6/1/2018	74.9	HEIGHT	7.4	13.5		1	0
26	6410	48	Azithromycin	0	30	5/19/2019	82.3	LENGTH	9.4	13.5		1	0
26	6410	60	Azithromycin	0	39	2/12/2020	89.5	HEIGHT	10.63636364	13.5		1	0
26	6412	24	Azithromycin	1	52	5/17/2017	103.3	HEIGHT	13.8	14.5		1	0
26	6412	48	Azithromycin	1	76	5/19/2019	118.3	HEIGHT	17.7	14.5		0	0
26	6416	0	Azithromycin	0	24	3/16/2015	86	HEIGHT	11.05	15.5		1	0
26	6416	12	Azithromycin	0	26	2/11/2016	92.4	HEIGHT	13.35	15		1	0
26	6416	24	Azithromycin	0	38	5/17/2017	102.6	HEIGHT	14.9	15		1	0
26	6416	36	Azithromycin	0	62	6/1/2018	111.1	HEIGHT	16.3	14.5		0	0
26	6416	48	Azithromycin	0	73	5/19/2019	115.7	HEIGHT	17.65	14.5		0	0
26	6416	60	Azithromycin	0	82	2/12/2020	120.9	HEIGHT	20.18181818	14.9		0	0
26	6417	12	Azithromycin	0	56	2/11/2016	108.7	HEIGHT	16.4	16		1	0
26	6421	0	Azithromycin	1	54	3/16/2015	83.7	HEIGHT	10.7	14.5		1	0
26	6423	24	Azithromycin	1	17	5/17/2017	72.9	HEIGHT	8.05	13.5		1	0
26	6423	48	Azithromycin	1	40	5/19/2019	87.8	HEIGHT	10.55	13.5		1	0
26	6423	60	Azithromycin	1	49	2/12/2020	94.9	HEIGHT	12.59090909	13.8		1	0
26	6424	0	Azithromycin	0	24	3/16/2015	84	HEIGHT	10.4	14.4	6	1	0
26	6425	12	Azithromycin	0	38	2/12/2016	98.7	HEIGHT	13.45	14.5		1	0
26	6425	24	Azithromycin	0	52	5/17/2017	109.5	HEIGHT	14.9	14.5		1	0
26	6425	48	Azithromycin	0	76	5/19/2019	122	HEIGHT	18	14		0	0
26	6426	24	Azithromycin	1	44	5/20/2017	89.2	HEIGHT	11.59090909	14		1	0
26	6426	36	Azithromycin	1	57	6/1/2018	96.9	HEIGHT	12.75	13.5		1	0
26	6426	48	Azithromycin	1	68	5/19/2019	103.1	HEIGHT	13.35	13.5		0	0

26	6427	12	Azithromycin	0	62	2/12/2016	110.2	HEIGHT	18.2	16		0	0
26	6429	0	Azithromycin	0	24	3/16/2015	63.9	LENGTH	6.7	13.1	18	1	0
26	6430	12	Azithromycin	0	53	2/12/2016	100.6	HEIGHT	14.55	15.5		1	0
26	6431	12	Azithromycin	1	17	2/11/2016	73.9	LENGTH	8.65	14		1	0
26	6431	24	Azithromycin	1	32	5/20/2017	87.2	HEIGHT	10.59090909	13		1	0
26	6431	36	Azithromycin	1	45	6/1/2018	94.7	HEIGHT	11.95	13		1	0
26	6431	48	Azithromycin	1	56	5/19/2019	101.7	HEIGHT	13.65	14		1	0
26	6432	12	Azithromycin	1	20	2/11/2016	72.8	LENGTH	8.35	14		1	0
26	6432	24	Azithromycin	1	34	5/17/2017	82.5	HEIGHT	10.3	15		1	0
26	6432	36	Azithromycin	1	47	6/1/2018	91.2	HEIGHT	11.75	14		1	0
26	6432	48	Azithromycin	1	58	5/19/2019	97.3	HEIGHT	13.65	14.5		1	0
26	6432	60	Azithromycin	1	56	2/20/2020	104.5	HEIGHT	15	14.3		1	0
26	6433	0	Azithromycin	0	48	3/16/2015	103.2	HEIGHT	15.45	15.7		1	0
26	6433	12	Azithromycin	0	53	2/12/2016	109.6	HEIGHT	17.7	16		1	0
26	6433	24	Azithromycin	0	68	5/17/2017	118.5	HEIGHT	19.35	14.5		0	0
26	6433	36	Azithromycin	0	81	6/1/2018	125.2	HEIGHT	21.75	16		0	0
26	6433	48	Azithromycin	0	92	5/19/2019	128.4	HEIGHT	23.55	16		0	0
26	6433	60	Azithromycin	0	101	2/12/2020	134.5	HEIGHT	26.13636364	15.5		0	0
26	6434	0	Azithromycin	0	48	3/16/2015	104	HEIGHT	16	15.4		1	0
26	6435	24	Azithromycin	1	34	5/17/2017	85.1	LENGTH	10.25	13		1	0
26	6435	36	Azithromycin	1	47	6/1/2018	95.5	HEIGHT	12.55	14		1	0
26	6435	48	Azithromycin	1	55	5/19/2019	103.3	HEIGHT	14.55	14.5		0	0
26	6436	12	Azithromycin	0	53	2/11/2016	89.4	HEIGHT	11.75	15		1	0
26	6437	0	Azithromycin	1	24	3/16/2015	70.4	LENGTH	7.25	13.1		1	0
26	6438	12	Azithromycin	1	3	2/12/2016	58.2	HEIGHT	4.95	13		1	0
26	6438	24	Azithromycin	1	17	5/17/2017	73.1	LENGTH	7.95	14.5		1	0
26	6439	0	Azithromycin	1	12	3/16/2015	71.9	LENGTH	7.7	12.7		1	0
26	6439	12	Azithromycin	1	29	2/11/2016	79.3	LENGTH	9.55	14.5		1	0
26	6439	24	Azithromycin	1	44	5/17/2017	87.6	HEIGHT	11.75	14.5		1	0
26	6439	36	Azithromycin	1	56	6/1/2018	96.2	HEIGHT	12.8	14		1	0
26	6439	48	Azithromycin	1	67	5/19/2019	103.4	HEIGHT	14.1	14		0	0
26	6439	60	Azithromycin	1	61	2/12/2020	107.9	HEIGHT	15.72727273	14		0	0
26	6442	12	Azithromycin	0	20	2/12/2016	76.9	HEIGHT	9.1	14.5		1	0
26	6442	24	Azithromycin	0	34	5/17/2017	86.6	HEIGHT	11	14		1	0
26	6442	36	Azithromycin	0	47	6/1/2018	95.1	HEIGHT	13.25	14.5		1	0
26	6442	48	Azithromycin	0	58	5/19/2019	101.8	HEIGHT	14.3	14.5		1	0
26	6443	12	Azithromycin	0	96	2/12/2016	71.2	LENGTH	8.95	15.5		1	0
26	6443	36	Azithromycin	0	34	6/1/2018	92.6	HEIGHT	13.35	15		1	0
26	6443	48	Azithromycin	0	45	5/19/2019	99.9	HEIGHT	13.95	14.5		1	0
26	6443	60	Azithromycin	0	55	2/12/2020	105.8	HEIGHT	15.59090909	14		1	0

26	6445	12	Azithromycin	1	29	2/11/2016	71.8	LENGTH	7.4	13		1	0
26	6445	24	Azithromycin	1	44	5/17/2017	83.8	HEIGHT	9.35	13		1	0
26	6445	36	Azithromycin	1	56	6/1/2018	104.3	HEIGHT	13.6	14		1	0
26	6445	48	Azithromycin	1	67	5/19/2019	108.8	HEIGHT	14.5	14		0	0
26	6447	0	Azithromycin	1	36	3/16/2015	88	LENGTH	12.1	16		1	0
26	6447	12	Azithromycin	1	44	2/12/2016	95.3	HEIGHT	14	16		0	0
26	6450	12	Azithromycin	0	12	2/12/2016	71.5	LENGTH	8.1	13.5	42	1	0
26	6452	12	Azithromycin	0	29	2/12/2016	81.8	LENGTH	9.3	13.5	48	1	0
26	6452	36	Azithromycin	0	57	6/1/2018	101.4	HEIGHT	13.2	14	48	1	0
26	6458	24	Azithromycin	0	17	5/17/2017	80.3	HEIGHT	10.9	14.5		1	0
26	6458	36	Azithromycin	0	30	6/1/2018	88.5	HEIGHT	13.15	15		1	0
26	6459	0	Azithromycin	0	24	3/16/2015	72.8	LENGTH	8.3	14	6	1	0
26	6461	24	Azithromycin	0	12	5/17/2017	73.5	HEIGHT	8.3	14		1	0
26	6461	36	Azithromycin	0	25	6/1/2018	82.5	HEIGHT	10.85	15		1	0
26	6461	48	Azithromycin	0	36	5/19/2019	91.1	HEIGHT	12.85	14.5		1	0
26	6461	60	Azithromycin	0	45	2/12/2020	97.1	HEIGHT	14.27272727	14.5		1	0
26	6463	0	Azithromycin	0	12	3/16/2015	67.1	LENGTH	7.7	14.5		1	0
26	6464	0	Azithromycin	0	48	3/16/2015	96.8	HEIGHT	13.2	15.1		1	0
26	6464	12	Azithromycin	0	53	2/12/2016	103.1	HEIGHT	15	14.5		1	0
26	6464	24	Azithromycin	0	68	5/17/2017	109.3	HEIGHT	16.7	15		0	0
26	6464	36	Azithromycin	0	80	6/1/2018	114.7	HEIGHT	19.15	15.5		0	0
26	6464	48	Azithromycin	0	91	5/19/2019	118.7	HEIGHT	20.45	16		0	0
26	6464	60	Azithromycin	0	100	2/12/2020	122.6	HEIGHT	22.77272727	16		0	0
26	6467	24	Azithromycin	1	52	5/17/2017	101.8	HEIGHT	14.95	16		1	0
26	6467	48	Azithromycin	1	76	5/19/2019	114.1	HEIGHT	19.55	17		0	0
26	6470	12	Azithromycin	1	53	2/12/2016	96	HEIGHT	13.75	15.5		1	0
26	6471	24	Azithromycin	1	50	5/17/2017	95.2	HEIGHT	12.8	14		1	0
26	6471	48	Azithromycin	1	74	5/19/2019	110	HEIGHT	15.8	14.5		0	0
26	6472	0	Azithromycin	1	36	3/16/2015	79.2	HEIGHT	11.35	16		1	0
26	6472	24	Azithromycin	1	68	5/17/2017	95.2	HEIGHT	15.45	15.5		0	0
26	6472	36	Azithromycin	1	80	6/1/2018	100	HEIGHT	17	16		0	0
26	6472	48	Azithromycin	1	91	5/19/2019	105.1	HEIGHT	18.25	16.5		0	0
26	6472	60	Azithromycin	1	100	2/12/2020	109.2	HEIGHT	19.81818182	16		0	0
26	6473	24	Azithromycin	1	56	5/17/2017	103.2	HEIGHT	13.6	14		1	0
26	6473	48	Azithromycin	1	79	5/19/2019	114.9	HEIGHT	18.05	15		0	0
26	6474	0	Azithromycin	0	48	3/16/2015	102.3	HEIGHT	15.85	15.5		1	0
26	6474	12	Azithromycin	1	77	2/12/2016	108.2	HEIGHT	17.8	15.6		0	0
26	6476	24	Azithromycin	0	62	5/17/2017	91.9	HEIGHT	11.95	14		1	0
26	6480	12	Azithromycin	1	26	2/11/2016	89.8	HEIGHT	12.1	16.8		1	0
26	6480	24	Azithromycin	1	37	5/17/2017	98.2	HEIGHT	14.65	17.5		1	0

26	6480	36	Azithromycin	1	49	6/1/2018	105.5	HEIGHT	16.05	17		1	0
26	6480	48	Azithromycin	1	60	5/19/2019	112	HEIGHT	17.9	17		0	0
26	6481	0	Azithromycin	0	24	3/16/2015	78.7	HEIGHT	9	14		1	0
26	6481	12	Azithromycin	0	41	2/12/2016	83.1	HEIGHT	11.65	15		0	0
26	6483	24	Azithromycin	0	28	5/20/2017	85.4	HEIGHT	11.72727273	16		1	0
26	6483	36	Azithromycin	0	40	6/1/2018	94	HEIGHT	13.2	15		1	0
26	6483	48	Azithromycin	0	51	5/19/2019	101.4	HEIGHT	14.95	14.5		1	0
26	6484	12	Azithromycin	1	53	2/12/2016	115.4	HEIGHT	17.75	14.5		1	0
26	6484	48	Azithromycin	1	91	5/19/2019	130.3	HEIGHT	22.7	16		0	0
26	6486	24	Azithromycin	0	15	5/20/2017	71.4	LENGTH	8.818181818	15		1	0
26	6486	36	Azithromycin	0	27	6/1/2018	81.1	HEIGHT	11.05	15		1	0
26	6486	48	Azithromycin	0	38	5/19/2019	88.8	HEIGHT	11.5	14		1	0
26	6486	60	Azithromycin	0	47	2/12/2020	93.7	HEIGHT	13.09090909	14.5		1	0
26	6487	12	Azithromycin	0	50	2/11/2016	96.2	HEIGHT	13.1	15.5		1	0
26	6488	12	Azithromycin	0	53	2/12/2016	99.4	HEIGHT	13.4	13.3		1	0
26	6488	48	Azithromycin	0	91	5/19/2019	115.4	HEIGHT	16.9	13		0	0
26	6492	0	Azithromycin	1	48	3/16/2015	101	HEIGHT	13.9	15		1	0
26	6492	12	Azithromycin	1	50	2/12/2016	105.2	HEIGHT	15.75	15.5		1	0
26	6492	24	Azithromycin	1	62	5/17/2017	111.8	HEIGHT	16.3	15		0	0
26	6492	36	Azithromycin	1	74	6/1/2018	118.4	HEIGHT	18.4	15.5		0	0
26	6492	48	Azithromycin	1	85	5/19/2019	122.7	HEIGHT	20	16		0	0
26	6492	60	Azithromycin	1	94	2/12/2020	125.9	HEIGHT	22.31818182	16		0	0
26	6493	24	Azithromycin	1	44	5/20/2017	104	HEIGHT	14.22727273	15.5		1	0
26	6495	24	Azithromycin	1	17	5/17/2017	68.8	LENGTH	6.4	11.5		1	0
26	6497	0	Azithromycin	1	48	3/16/2015	88.6	HEIGHT	11.85	13.9	12	1	0
26	6501	12	Azithromycin	0	5	2/12/2016	63.5	LENGTH	6.55	13.5		1	0
26	6501	24	Azithromycin	0	17	5/17/2017	79.7	HEIGHT	9.15	13.5		1	0
26	6501	36	Azithromycin	0	29	6/1/2018	88.8	HEIGHT	10.85	13.5		1	0
26	6501	48	Azithromycin	0	40	5/19/2019	96	HEIGHT	12.1	13		1	0
26	6501	60	Azithromycin	0	50	2/12/2020	101.7	HEIGHT	13.63636364	13.5		1	0
26	6503	24	Azithromycin	1	10	5/17/2017	66.1	HEIGHT	6.95	13.5		1	0
26	6503	36	Azithromycin	1	22	6/1/2018	76.3	LENGTH	8.75	13.5		1	0
26	6503	60	Azithromycin	1	42	2/12/2020	90.2	HEIGHT	10.09090909	13.7		1	0
26	6504	12	Azithromycin	1	29	2/12/2016	83.5	HEIGHT	10.95	14		1	0
26	6504	36	Azithromycin	1	56	6/1/2018	104	HEIGHT	15.35	14.5		1	0
26	6504	48	Azithromycin	1	57	5/19/2019	110.2	HEIGHT	17.1	15.5		1	0
26	6508	0	Azithromycin	1	36	3/16/2015	90.7	HEIGHT	11.9	14.6		1	0
26	6508	36	Azithromycin	1	75	6/1/2018	110.3	HEIGHT	16.65	15.5		0	0
26	6509	0	Azithromycin	1	54	3/16/2015	98.3	HEIGHT	14.15	15.5		1	0
26	6509	12	Azithromycin	1	53	2/12/2016	103.5	HEIGHT	15.3	15		1	0

26	6509	24	Azithromycin	1	76	5/17/2017	111.1	HEIGHT	18	16		0	0
26	6509	36	Azithromycin	1	89	6/1/2018	117.5	HEIGHT	19.35	15.5		0	0
26	6509	48	Azithromycin	1	100	5/19/2019	123	HEIGHT	21.2	16		0	0
26	6509	60	Azithromycin	1	109	2/12/2020	127.1	HEIGHT	24.36363636	16.8		0	0
26	6511	24	Azithromycin	0	17	5/17/2017	79.7	HEIGHT	10.2	14		1	0
26	6511	36	Azithromycin	0	33	6/1/2018	89.5	HEIGHT	11.85	14		1	0
26	6511	48	Azithromycin	0	44	5/19/2019	96.5	HEIGHT	13.35	14		1	0
26	6511	60	Azithromycin	0	54	2/12/2020	101.2	HEIGHT	14.09090909	13.2		1	0
26	6512	0	Azithromycin	1	12	3/16/2015	65.7	LENGTH	5.6	12		1	0
26	6512	12	Azithromycin	1	17	2/12/2016	74	HEIGHT	6.95	11.5		1	0
26	6512	24	Azithromycin	1	37	5/17/2017	82.8	HEIGHT	8.65	12.5		1	0
26	6512	36	Azithromycin	1	49	6/1/2018	89.8	HEIGHT	10.1	12.5		1	0
26	6512	48	Azithromycin	1	60	5/19/2019	96.4	HEIGHT	11.45	14		0	0
26	6512	60	Azithromycin	1	69	2/12/2020	101.5	HEIGHT	12.63636364	13		0	0
26	6513	0	Azithromycin	0	42	3/16/2015	91.3	HEIGHT	11.95	15		1	0
26	6513	12	Azithromycin	0	56	2/11/2016	94.9	HEIGHT	13.75	15		1	0
26	6513	24	Azithromycin	0	70	5/17/2017	103.3	HEIGHT	14.45	14.5		0	0
26	6513	36	Azithromycin	0	83	6/1/2018	108.8	HEIGHT	15.65	14		0	0
26	6513	48	Azithromycin	0	94	5/19/2019	116.1	HEIGHT	17.7	15		0	0
26	6515	24	Azithromycin	0	15	5/17/2017	70.9	HEIGHT	8.35	14		1	0
26	6515	36	Azithromycin	0	28	6/1/2018	81.6	LENGTH	10.25	14.5		1	0
26	6515	48	Azithromycin	0	36	5/19/2019	88.2	HEIGHT	12	15		1	0
26	6515	60	Azithromycin	0	45	2/12/2020	95.2	HEIGHT	14.09090909	14.9		1	0
26	6516	12	Azithromycin	0	41	2/11/2016	99.9	HEIGHT	14.95	15.5	42	1	0
26	6518	0	Azithromycin	1	48	3/16/2015	103.1	HEIGHT	16.2	16		1	0
26	6519	24	Azithromycin	0	13	5/17/2017	69.3	LENGTH	7.95	14		1	0
26	6519	48	Azithromycin	0	37	5/19/2019	85.7	HEIGHT	11.45	14.5		0	0
26	6525	12	Azithromycin	0	23	2/11/2016	79.7	LENGTH	8.7	12.8		1	0
26	6525	24	Azithromycin	0	38	5/17/2017	87.4	HEIGHT	10.05	13		1	0
26	6525	36	Azithromycin	0	50	6/1/2018	93.7	HEIGHT	10.75	12.5		1	0
26	6525	48	Azithromycin	0	61	5/19/2019	99.3	HEIGHT	12.35	12.5		0	0
26	6525	60	Azithromycin	0	56	2/20/2020	103.1	HEIGHT	13.31818182	13		1	0
26	6526	24	Azithromycin	0	12	5/17/2017	73.1	LENGTH	6.65	11.5		1	0
26	6526	36	Azithromycin	0	24	6/1/2018	84.9	HEIGHT	10.15	13.5		1	0
26	6526	48	Azithromycin	0	35	5/19/2019	92.2	HEIGHT	12.05	14		1	0
26	6526	60	Azithromycin	0	44	2/12/2020	97.5	HEIGHT	13.31818182	14.2		1	0
26	6529	24	Azithromycin	0	38	5/17/2017	101.8	HEIGHT	14.65	15	48	1	0
26	6529	36	Azithromycin	0	51	6/1/2018	107.7	HEIGHT	15.4	14	48	1	0
26	6531	24	Azithromycin	1	10	5/17/2017	67.9	LENGTH	6.7	13.5		1	0
26	6531	36	Azithromycin	1	23	6/1/2018	78.6	HEIGHT	9.2	14		1	0

26	6531	48	Azithromycin	1	33	5/19/2019	87.4	HEIGHT	10.35	14		1	0
26	6531	60	Azithromycin	1	43	2/12/2020	92.7	HEIGHT	11.81818182	13.9		0	0
26	6532	12	Azithromycin	0	2	2/11/2016	59.2	LENGTH	6.15	14.5		0	0
26	6532	48	Azithromycin	0	35	5/19/2019	94.4	LENGTH	12.2	14		1	0
26	6532	60	Azithromycin	0	44	2/12/2020	99.1	HEIGHT	13.40909091	13.4		1	0
26	6533	0	Azithromycin	1	36	3/16/2015	90.9	HEIGHT	12.3	14.5		1	0
26	6533	12	Azithromycin	1	53	2/12/2016	97.3	HEIGHT	13.35	14.5		1	0
26	6533	24	Azithromycin	1	68	5/17/2017	106	HEIGHT	14.65	13.5		0	0
26	6533	36	Azithromycin	1	80	6/1/2018	113.4	HEIGHT	16.55	14		0	0
26	6533	60	Azithromycin	1	100	2/12/2020	122.8	HEIGHT	20.27272727	15		0	0
26	8002	48	Azithromycin	0	33	5/19/2019	85.2	HEIGHT	11.3	14.5		1	0
26	8002	60	Azithromycin	0	43	2/12/2020	91.7	HEIGHT	13.36363636	15		1	0
26	8005	36	Azithromycin	0	12	6/1/2018	74.9	LENGTH	7.5	12		1	0
26	8005	48	Azithromycin	0	22	5/19/2019	85.4	HEIGHT	9.15	12.5		1	0
26	8005	60	Azithromycin	0	32	2/12/2020	89.9	HEIGHT	10.81818182	12.5		1	0
26	8009	48	Azithromycin	0	5	5/19/2019	68.5	LENGTH	6.8	12		1	0
26	8038	60	Azithromycin	0	31	2/12/2020	89.3	HEIGHT	12.68181818	14.4		1	0
26	8091	48	Azithromycin	0	12	5/19/2019	71.4	LENGTH	8.45	14.5		1	0
26	8091	60	Azithromycin	0	21	2/12/2020	79.4	HEIGHT	9.136363636	14		1	0
26	8137	36	Azithromycin	0	8	6/1/2018	75.6	LENGTH	9.55	15		1	0
26	8137	48	Azithromycin	0	15	5/19/2019	85.8	HEIGHT	11.05	13.5		1	0
26	8137	60	Azithromycin	0	24	2/12/2020	92.2	HEIGHT	12.59090909	13.7		1	0
26	8155	48	Azithromycin	0	26	5/19/2019	81.5	HEIGHT	9.3	13.5		1	0
26	8300	60	Azithromycin	0	5	2/20/2020	65.4	LENGTH	7.136363636	12.8		1	0
26	8341	48	Azithromycin	1	16	5/19/2019	77.5	LENGTH	8.65	13		1	0
26	8341	60	Azithromycin	1	25	2/12/2020	82.6	HEIGHT	10.09090909	13.3		1	0
26	8349	48	Azithromycin	1	3	5/19/2019	55.8	LENGTH	4.8	11		1	0
26	8385	36	Azithromycin	0	14	6/1/2018	74	HEIGHT	8.65	15.5		1	0
26	8418	60	Azithromycin	1	9	2/20/2020	66.9	LENGTH	6.681818182	11.6		1	0
26	8525	48	Azithromycin	0	8	5/19/2019	68.3	HEIGHT	6.3	12		1	0
26	8525	60	Azithromycin	0	12	2/12/2020	75.5	LENGTH	7.5	11.5		1	0
26	8617	48	Azithromycin	0	28	5/19/2019	81.5	LENGTH	9.7	14		1	0
26	8617	60	Azithromycin	0	37	2/12/2020	86.5	HEIGHT	11.86363636	14.2		1	0
26	8719	60	Azithromycin	0	14	2/12/2020	71.7	HEIGHT	7.727272727	13		1	0
26	8738	48	Azithromycin	1	6	5/19/2019	65.3	LENGTH	6.95	12.5		1	0
26	8738	60	Azithromycin	1	14	2/12/2020	76.6	HEIGHT	8.727272727	13.5		1	0
26	8749	60	Azithromycin	1	36	2/12/2020	83.6	HEIGHT	10.09090909	12.5		1	0
26	8773	60	Azithromycin	0	11	2/12/2020	72.7	LENGTH	9.863636364	14.8		1	0
26	8817	60	Azithromycin	1	39	2/12/2020	92.5	HEIGHT	12.31818182	14		1	0
26	8857	36	Azithromycin	1	4	6/1/2018	59.7	LENGTH	5.65	13.5		1	0

26	8857	48	Azithromycin	1	15	5/19/2019	74.7	HEIGHT	7.8	13		1	0
26	8857	60	Azithromycin	1	24	2/12/2020	81.1	HEIGHT	10.27272727	14		1	0
26	9045	48	Azithromycin	0	40	5/19/2019	81.8	HEIGHT	10.2	13		1	0
26	9045	60	Azithromycin	0	49	2/12/2020	86.9	HEIGHT	13.04545455	14.3		1	0
26	9096	48	Azithromycin	1	36	5/19/2019	89	HEIGHT	12.4	15		1	0
26	9118	60	Azithromycin	0	14	2/12/2020	77.6	HEIGHT	10.13636364	14		1	0
26	9125	60	Azithromycin	0	38	2/12/2020	90.9	HEIGHT	12.81818182	14.5		1	0
26	9152	36	Azithromycin	0	7	6/1/2018	67.4	LENGTH	8.15	14.5		1	0
26	9152	48	Azithromycin	0	18	5/19/2019	77.8	LENGTH	10.25	14.5		1	0
26	9152	60	Azithromycin	0	27	2/12/2020	84.9	HEIGHT	12.45454545	15.2		1	0
26	9180	48	Azithromycin	0	4	5/19/2019	62.9	LENGTH	6.25	12.5		1	0
26	9180	60	Azithromycin	0	13	2/12/2020	73.6	LENGTH	8.136363636	12.5		1	0
26	9185	48	Azithromycin	0	50	5/19/2019	87.2	HEIGHT	13.4	14.5	54	1	0
26	9192	36	Azithromycin	0	12	6/1/2018	73.1	LENGTH	8.5	14		1	0
26	9192	48	Azithromycin	0	23	5/19/2019	81.3	HEIGHT	9.7	14		1	0
26	9192	60	Azithromycin	0	32	2/12/2020	85.6	HEIGHT	11.68181818	15.2		0	0
26	9214	60	Azithromycin	1	20	2/12/2020	84.8	HEIGHT	11.27272727	13.5		1	0
26	9223	48	Azithromycin	0	16	5/19/2019	74.5	LENGTH	8.95	14		1	0
26	9241	48	Azithromycin	0	56	5/19/2019	106.1	HEIGHT	16.75	15		1	0
26	9241	60	Azithromycin	0	56	2/12/2020	109.7	HEIGHT	17.40909091	14.5		1	0
26	9242	36	Azithromycin	0	7	6/1/2018	64	LENGTH	7.35	14		1	0
26	9242	48	Azithromycin	0	18	5/19/2019	73.4	LENGTH	8.75	13		1	0
26	9242	60	Azithromycin	0	27	2/12/2020	78.8	HEIGHT	10.22727273	13		0	0
26	9267	60	Azithromycin	1	8	2/12/2020	69.5	LENGTH	9.363636364	14		1	0
26	9301	48	Azithromycin	1	52	5/19/2019	100.2	HEIGHT	16.3	17		1	0
26	9467	48	Azithromycin	0	57	5/19/2019	101.4	HEIGHT	15.35	14.5		1	0
26	9472	48	Azithromycin	1	12	5/19/2019	76.7	LENGTH	9.4	14		1	0
26	9472	60	Azithromycin	1	20	2/12/2020	81.6	HEIGHT	12.09090909	14.4		1	0
26	9493	48	Azithromycin	1	12	5/19/2019	72.4	LENGTH	7.6	12		1	0
27	6548	0	Azithromycin	1	42	6/2/2015	108.9	HEIGHT	15.5	14.1		1	1
27	6548	36	Azithromycin	1	124	5/11/2018	126.1	HEIGHT	20.75	15		0	1
27	6548	48	Azithromycin	1	134	4/13/2019	132.4	HEIGHT	23.7	16		0	1
27	6548	60	Azithromycin	1	144	2/4/2020	135.1	HEIGHT	25.65	16		0	1
27	6549	0	Azithromycin	1	3	3/12/2015	56.1	LENGTH	5.05	13.6		1	1
27	6549	36	Azithromycin	1	44	5/11/2018	86.5	HEIGHT	10.55	14.2		1	1
27	6549	48	Azithromycin	1	55	4/13/2019	93.4	HEIGHT	11.85	13.9		1	1
27	6549	60	Azithromycin	1	65	2/4/2020	98.3	HEIGHT	13.4	13.7		0	1
27	6550	12	Azithromycin	1	17	6/13/2016	86.5	HEIGHT	12.4	16		1	1
27	6552	0	Azithromycin	1	30	3/12/2015	94.4	HEIGHT	13.55	16.9		1	1
27	6552	12	Azithromycin	1	66	6/13/2016	103.5	HEIGHT	15.25	15		0	1

27	6552	48	Azithromycin	1	90	4/13/2019	121	HEIGHT	18.6	16.3	0	1
27	6554	0	Azithromycin	1	48	3/12/2015	95.7	HEIGHT	12.6	14.5	0	1
27	6554	12	Azithromycin	1	61	5/14/2016	103.9	HEIGHT	14	14	0	1
27	6554	24	Azithromycin	1	72	3/18/2017	109.5	HEIGHT	14.75	14	0	1
27	6554	36	Azithromycin	1	86	5/11/2018	115.6	HEIGHT	16.6	14	0	1
27	6554	48	Azithromycin	1	97	4/13/2019	120.1	HEIGHT	18.3	14.2	0	1
27	6555	12	Azithromycin	0	44	5/14/2016	92.9	HEIGHT	11.4	14.5	1	1
27	6556	0	Azithromycin	0	52	3/12/2015	118.3	HEIGHT	19.7	15.3	0	1
27	6556	36	Azithromycin	0	120	5/11/2018	134.5	HEIGHT	24.9	15	0	1
27	6556	48	Azithromycin	0	131	4/13/2019	138.4	HEIGHT	28.3	17	0	1
27	6557	12	Azithromycin	0	6	5/14/2016	67.3	LENGTH	6.45	11.7	1	1
27	6557	24	Azithromycin	0	14	3/18/2017	74.3	LENGTH	8.8	13	0	1
27	6557	36	Azithromycin	0	24	5/11/2018	82.3	LENGTH	10.15	14	0	1
27	6557	48	Azithromycin	0	34	4/13/2019	90.5	HEIGHT	11.75	13.6	0	1
27	6557	60	Azithromycin	0	44	2/4/2020	97.1	HEIGHT	13.4	13.7	1	1
27	6559	0	Azithromycin	0	30	3/12/2015	74.8	LENGTH	9	14.4	0	1
27	6559	12	Azithromycin	0	33	5/14/2016	86	HEIGHT	10.95	14	1	1
27	6559	24	Azithromycin	0	44	3/18/2017	92	HEIGHT	13.05	14	0	1
27	6559	36	Azithromycin	0	60	5/11/2018	101.5	HEIGHT	14.85	14	0	1
27	6559	48	Azithromycin	0	70	4/13/2019	107.5	HEIGHT	15.75	14.7	0	1
27	6559	60	Azithromycin	0	80	2/4/2020	112.7	HEIGHT	17.85	15	0	1
27	6561	0	Azithromycin	1	36	3/12/2015	87.3	HEIGHT	11.4	13.2	1	1
27	6561	12	Azithromycin	1	36	6/13/2016	96.2	HEIGHT	12.9	13	0	1
27	6561	24	Azithromycin	1	47	3/18/2017	102.2	HEIGHT	14.55	13.5	1	1
27	6561	36	Azithromycin	1	72	5/11/2018	108.5	HEIGHT	16.2	14	0	1
27	6561	48	Azithromycin	1	82	4/13/2019	115.3	HEIGHT	16.95	13.9	0	1
27	6565	0	Azithromycin	0	49	3/12/2015	107.8	HEIGHT	17.85	15.2	1	1
27	6565	24	Azithromycin	0	83	3/18/2017	117.1	HEIGHT	20.55	14.5	0	1
27	6565	36	Azithromycin	0	98	5/11/2018	122.5	HEIGHT	20.75	15	0	1
27	6565	48	Azithromycin	0	109	4/13/2019	127	HEIGHT	23.85	16	0	1
27	6566	0	Azithromycin	0	36	3/12/2015	93.6	HEIGHT	13.1	15	1	1
27	6566	12	Azithromycin	0	45	5/15/2016	101.5	HEIGHT	15.35	15	0	1
27	6566	24	Azithromycin	0	67	3/18/2017	108.5	HEIGHT	17.1	15	0	1
27	6566	36	Azithromycin	0	80	5/11/2018	114.9	HEIGHT	19.3	15	0	1
27	6566	48	Azithromycin	0	91	4/13/2019	119.3	HEIGHT	20.18181818	14.6	0	1
27	6567	0	Azithromycin	0	9	3/12/2015	58.6	LENGTH	5.2	12.4	1	1
27	6567	12	Azithromycin	0	15	5/14/2016	71.3	LENGTH	7.3	12.2	0	1
27	6568	0	Azithromycin	1	36	3/12/2015	101.5	HEIGHT	16.15	17	0	1
27	6570	0	Azithromycin	1	7	3/12/2015	59.6	LENGTH	5.55	13.5	0	1
27	6570	24	Azithromycin	1	26	3/18/2017	84.3	HEIGHT	11.6	14.5	1	1

27	6570	36	Azithromycin	1	41	5/11/2018	96	HEIGHT	14.2	15	0	1
27	6570	48	Azithromycin	1	51	4/13/2019	104.3	HEIGHT	15.9	15.5	0	1
27	6570	60	Azithromycin	1	61	2/4/2020	111.6	HEIGHT	18.2	15.4	0	1
27	6571	24	Azithromycin	1	23	3/18/2017	80	HEIGHT	9.3	12.4	1	1
27	6571	36	Azithromycin	1	37	5/11/2018	89.1	HEIGHT	10.6	12	1	1
27	6571	48	Azithromycin	1	48	4/13/2019	95.5	HEIGHT	11.8	12.5	1	1
27	6573	0	Azithromycin	1	48	3/12/2015	104.5	HEIGHT	15.1	15.5	0	1
27	6573	36	Azithromycin	1	86	5/11/2018	124.5	HEIGHT	21.7	16	0	1
27	6573	48	Azithromycin	1	97	4/13/2019	132.4	HEIGHT	24.65	17.2	0	1
27	6574	0	Azithromycin	0	19	6/2/2015	76.5	LENGTH	8.3	12.5	1	1
27	6574	36	Azithromycin	0	56	5/11/2018	95.1	HEIGHT	13.45	14	0	1
27	6574	48	Azithromycin	0	67	4/13/2019	101.2	HEIGHT	14.55	14.6	0	1
27	6575	0	Azithromycin	1	24	3/12/2015	96.8	HEIGHT	14.25	15.1	1	1
27	6575	12	Azithromycin	1	54	5/14/2016	103.4	HEIGHT	16.5	15.5	0	1
27	6575	24	Azithromycin	1	65	3/18/2017	109	HEIGHT	18	15.5	0	1
27	6575	36	Azithromycin	1	79	5/11/2018	113.5	HEIGHT	20.05	16	0	1
27	6575	48	Azithromycin	1	90	4/13/2019	118.5	HEIGHT	21.6	16.6	0	1
27	6575	60	Azithromycin	1	100	2/4/2020	122.6	HEIGHT	22.95	17	0	1
27	6576	0	Azithromycin	0	36	3/12/2015	91.9	HEIGHT	14.3	17.1	1	1
27	6576	12	Azithromycin	0	49	5/15/2016	101.9	HEIGHT	15.65	15	0	1
27	6576	24	Azithromycin	0	60	3/18/2017	106.5	HEIGHT	17	15	0	1
27	6576	36	Azithromycin	0	78	5/11/2018	112.5	HEIGHT	19.1	15	0	1
27	6576	60	Azithromycin	0	99	2/4/2020	121.6	HEIGHT	23.4	17.2	0	1
27	6577	0	Azithromycin	1	5	3/12/2015	66.9	LENGTH	7.6	15.3	0	1
27	6577	12	Azithromycin	1	18	5/15/2016	79.6	LENGTH	10.2	14.5	0	1
27	6577	24	Azithromycin	1	29	3/18/2017	85	HEIGHT	12.35	15.5	0	1
27	6577	36	Azithromycin	1	44	5/11/2018	93.9	HEIGHT	15.1	16	0	1
27	6577	48	Azithromycin	1	55	4/13/2019	102.3	HEIGHT	16.15	15.2	1	1
27	6577	60	Azithromycin	1	64	2/4/2020	108.9	HEIGHT	18.5	16	0	1
27	6580	0	Azithromycin	0	48	3/12/2015	105.5	HEIGHT	15.9	15.7	1	1
27	6580	12	Azithromycin	0	61	5/15/2016	114.3	HEIGHT	17.4	14.5	0	1
27	6580	24	Azithromycin	0	72	3/18/2017	120.2	HEIGHT	20.05	15	0	1
27	6580	48	Azithromycin	0	97	4/13/2019	131.3	HEIGHT	25.2	15.6	0	1
27	6580	60	Azithromycin	0	107	2/4/2020	134.2	HEIGHT	27.3	17	0	1
27	6582	12	Azithromycin	0	54	5/15/2016	106.3	HEIGHT	15.7	13.8	1	1
27	6583	0	Azithromycin	0	42	3/12/2015	90.8	HEIGHT	12.4	15.2	1	1
27	6583	12	Azithromycin	0	45	5/15/2016	99	HEIGHT	14.35	14.7	1	1
27	6583	24	Azithromycin	0	56	3/18/2017	104	HEIGHT	15.35	14.5	1	1
27	6584	24	Azithromycin	1	5	3/18/2017	67.3	LENGTH	7.05	13.5	1	1
27	6584	48	Azithromycin	1	31	4/13/2019	84.9	HEIGHT	11.75	15	0	1

27	6584	60	Azithromycin	1	40	2/4/2020	91.2	HEIGHT	12.75	14.5		1	1
27	6585	0	Azithromycin	0	54	3/12/2015	103.1	HEIGHT	15.6	15.4		1	1
27	6585	12	Azithromycin	0	57	6/13/2016	112.5	HEIGHT	17.45	14.5		0	1
27	6585	24	Azithromycin	0	68	3/18/2017	116.2	HEIGHT	19.6	15		0	1
27	6585	36	Azithromycin	0	89	5/11/2018	123.2	HEIGHT	22.35	16		0	1
27	6585	48	Azithromycin	0	100	4/13/2019	128.2	HEIGHT	23.3	15.6		0	1
27	6586	24	Azithromycin	1	10	3/18/2017	74.8	LENGTH	8.45	14		1	1
27	6587	0	Azithromycin	0	11	3/12/2015	66	LENGTH	8.2	16.2		0	1
27	6587	24	Azithromycin	0	26	3/18/2017	83.1	HEIGHT	12.3	16		1	1
27	6587	36	Azithromycin	0	47	5/11/2018	91.1	HEIGHT	13.95	16.5		1	1
27	6587	48	Azithromycin	0	58	4/13/2019	98.2	HEIGHT	15.15	15.5		1	1
27	6587	60	Azithromycin	0	68	2/4/2020	103.4	HEIGHT	16.95	14.7		0	1
27	6589	12	Azithromycin	1	30	5/14/2016	83.3	HEIGHT	10.55	14		1	1
27	6589	24	Azithromycin	1	41	3/18/2017	91	HEIGHT	13.05	15		1	1
27	6590	0	Azithromycin	0	48	3/12/2015	115.4	HEIGHT	18.9	16.6		0	1
27	6590	36	Azithromycin	0	110	5/11/2018	132	HEIGHT	26.4	18		0	1
27	6590	48	Azithromycin	0	121	4/13/2019	136.5	HEIGHT	27.5	18		0	1
27	6591	0	Azithromycin	0	9	3/12/2015	60.3	LENGTH	5.25	12		0	1
27	6591	12	Azithromycin	0	15	5/14/2016	74.4	HEIGHT	7.9	12.2		0	1
27	6593	0	Azithromycin	1	8	3/12/2015	59.8	LENGTH	5.65	13.7		0	1
27	6593	24	Azithromycin	0	23	3/18/2017	79.5	HEIGHT	9.8	14		1	1
27	6593	36	Azithromycin	0	37	5/11/2018	87.5	HEIGHT	11.5	14		0	1
27	6593	48	Azithromycin	0	48	4/13/2019	92	HEIGHT	12.25	14		1	1
27	6593	60	Azithromycin	0	58	2/4/2020	98.8	HEIGHT	13.9	15		1	1
27	6594	0	Azithromycin	0	54	3/12/2015	92.6	HEIGHT	12.8	14	12	0	1
27	6595	0	Azithromycin	1	12	3/12/2015	65.3	LENGTH	6.65	13.2		0	1
27	6595	12	Azithromycin	1	15	5/14/2016	74.8	LENGTH	8.6	13		1	1
27	6595	24	Azithromycin	1	33	3/18/2017	82.1	HEIGHT	11.05	14.5		0	1
27	6595	36	Azithromycin	1	48	5/11/2018	91.4	HEIGHT	13.9	16		0	1
27	6595	48	Azithromycin	1	59	4/13/2019	97.6	HEIGHT	15.45	16		0	1
27	6596	12	Azithromycin	0	45	5/14/2016	90.4	HEIGHT	11.9	13.3		1	1
27	6597	0	Azithromycin	0	13	6/2/2015	70.9	LENGTH	8.5	15.6		1	1
27	6597	12	Azithromycin	0	24	6/13/2016	81	HEIGHT	11.7	17.5		0	1
27	6597	24	Azithromycin	0	34	3/18/2017	86	HEIGHT	13.85	17.5		0	1
27	6597	48	Azithromycin	0	59	4/13/2019	106	HEIGHT	17.15	16.5		0	1
27	6597	60	Azithromycin	0	69	2/4/2020	105.1	HEIGHT	17.6	15.5		0	1
27	6602	0	Azithromycin	1	24	3/12/2015	79.6	HEIGHT	9.5	13.6		0	1
27	6602	24	Azithromycin	1	47	3/18/2017	95	HEIGHT	13.65	14.5		0	1
27	6602	36	Azithromycin	1	62	5/11/2018	104	HEIGHT	14.9	14.2		0	1
27	6602	48	Azithromycin	1	73	4/13/2019	110.6	HEIGHT	16.7	14.6		0	1

27	6602	60	Azithromycin	1	83	2/4/2020	116.9	HEIGHT	19	15.2		0	1
27	6603	0	Azithromycin	0	54	3/12/2015	104	HEIGHT	15.75	14.9		0	1
27	6603	12	Azithromycin	0	66	6/13/2016	110.5	HEIGHT	18.15	15.5		0	1
27	6603	48	Azithromycin	0	103	4/13/2019	122.3	HEIGHT	21.45	15.8		0	1
27	6604	0	Azithromycin	1	30	3/12/2015	92	HEIGHT	13.75	16.8		1	1
27	6604	12	Azithromycin	1	44	5/14/2016	104	HEIGHT	15.85	16		1	1
27	6604	24	Azithromycin	1	62	3/18/2017	106.4	HEIGHT	17.05	15.5		0	1
27	6604	36	Azithromycin	1	77	5/11/2018	113.5	HEIGHT	19.35	15.5		0	1
27	6604	60	Azithromycin	1	97	2/4/2020	120.5	HEIGHT	22.1	15.9		0	1
27	6605	0	Azithromycin	0	36	3/12/2015	80.6	HEIGHT	10.5	14.5		1	1
27	6605	12	Azithromycin	0	39	6/13/2016	92.5	HEIGHT	13.25	15		0	1
27	6605	24	Azithromycin	0	50	3/18/2017	98.5	HEIGHT	15.4	15		0	1
27	6605	36	Azithromycin	0	64	5/11/2018	108	HEIGHT	17.4	14.8		0	1
27	6605	48	Azithromycin	0	75	4/13/2019	114.5	HEIGHT	18.95	15		0	1
27	6605	60	Azithromycin	0	85	2/4/2020	119.6	HEIGHT	19.9	15		0	1
27	6610	12	Azithromycin	1	4	5/15/2016	63.8	LENGTH	5.85	13	42	1	1
27	6614	0	Azithromycin	0	5	3/12/2015	64.1	LENGTH	6.65	13		0	1
27	6614	24	Azithromycin	0	29	3/18/2017	85	HEIGHT	11.85	15		0	1
27	6614	36	Azithromycin	0	44	5/11/2018	95	HEIGHT	14.35	15		0	1
27	6614	48	Azithromycin	0	55	4/13/2019	103	HEIGHT	15.5	14.9		0	1
27	6615	12	Azithromycin	0	21	5/14/2016	77.7	LENGTH	9.5	13.5		1	1
27	6615	24	Azithromycin	0	32	3/18/2017	82.1	HEIGHT	10.7	14		1	1
27	6615	36	Azithromycin	0	47	5/11/2018	88.8	LENGTH	12.9	14.5		1	1
27	6618	12	Azithromycin	1	6	5/14/2016	63.8	LENGTH	5.4	11.2		1	1
27	6618	24	Azithromycin	1	14	3/18/2017	78	HEIGHT	9.1	14.5		1	1
27	6618	36	Azithromycin	1	28	5/11/2018	86.4	LENGTH	11.15	16		0	1
27	6619	0	Azithromycin	0	30	3/12/2015	73.2	LENGTH	8.35	14.4		1	1
27	6619	24	Azithromycin	0	44	3/18/2017	83.2	HEIGHT	12.9	16		0	1
27	6619	36	Azithromycin	0	59	5/11/2018	92.8	LENGTH	15.1	16.4		1	1
27	6619	48	Azithromycin	0	70	4/13/2019	101.2	HEIGHT	16.4	15.5		0	1
27	6619	60	Azithromycin	0	79	2/4/2020	106.3	HEIGHT	19	16		0	1
27	6620	0	Azithromycin	1	2	3/12/2015	57.9	LENGTH	5.8	13.2		1	1
27	6620	24	Azithromycin	1	26	3/18/2017	85	HEIGHT	10.8	15.5		0	1
27	6620	36	Azithromycin	1	41	5/11/2018	95	HEIGHT	14.15	16		0	1
27	6620	48	Azithromycin	1	52	4/13/2019	102.4	HEIGHT	15.3	14.9		1	1
27	6620	60	Azithromycin	1	61	2/4/2020	107.4	HEIGHT	16.5	14.3		0	1
27	6622	0	Azithromycin	1	36	3/12/2015	87.2	HEIGHT	11.7	14		0	1
27	6622	12	Azithromycin	1	49	5/14/2016	95.4	HEIGHT	12.1	13		0	1
27	6622	24	Azithromycin	1	60	3/18/2017	100.5	HEIGHT	13.85	13		0	1
27	6622	36	Azithromycin	1	74	5/11/2018	106.9	HEIGHT	15.5	14		0	1

27	6622	48	Azithromycin	1	85	4/13/2019	112.3	HEIGHT	16.7	14.5		0	1
27	6622	60	Azithromycin	1	95	2/4/2020	115.5	HEIGHT	18.45	15.3		0	1
27	6625	0	Azithromycin	1	24	3/12/2015	75.7	LENGTH	8.45	14	12	0	1
27	6626	24	Azithromycin	0	50	3/18/2017	113.5	HEIGHT	18.8	15		1	1
27	6629	24	Azithromycin	1	53	3/18/2017	96	HEIGHT	13.25	14.5		1	1
27	6631	12	Azithromycin	1	3	5/14/2016	63.7	LENGTH	6.1	13		0	1
27	6631	24	Azithromycin	1	20	3/18/2017	73.8	LENGTH	8.5	14		0	1
27	6631	36	Azithromycin	1	34	5/11/2018	86	HEIGHT	10.65	14		0	1
27	6631	48	Azithromycin	1	45	4/13/2019	92.2	HEIGHT	13.85	16.3		1	1
27	6631	60	Azithromycin	1	55	2/4/2020	98.5	HEIGHT	15.57	17.2		1	1
27	6634	0	Azithromycin	1	54	3/12/2015	100.2	HEIGHT	14.4	16.4		0	1
27	6634	36	Azithromycin	1	104	5/11/2018	120.5	HEIGHT	20.4	15.5		0	1
27	6634	48	Azithromycin	1	115	4/13/2019	123.6	HEIGHT	21.31818182	15.6		0	1
27	6635	0	Azithromycin	1	42	3/12/2015	87.4	HEIGHT	10.7	13.4		1	1
27	6635	12	Azithromycin	1	50	5/14/2016	96.4	HEIGHT	11.95	13		1	1
27	6635	24	Azithromycin	1	62	3/18/2017	103.1	HEIGHT	14.9	13.5		0	1
27	6635	36	Azithromycin	1	76	5/11/2018	110	HEIGHT	16.5	14		0	1
27	6635	48	Azithromycin	1	87	4/13/2019	117	HEIGHT	18.15	15		0	1
27	6635	60	Azithromycin	1	97	2/4/2020	122	HEIGHT	20.1	15.4		0	1
27	6637	0	Azithromycin	1	24	3/12/2015	84.4	HEIGHT	11.6	16		1	1
27	6637	12	Azithromycin	1	37	5/15/2016	91.6	HEIGHT	11.7	14		1	1
27	6637	24	Azithromycin	1	48	3/18/2017	96	HEIGHT	13.7	14.5		0	1
27	6637	36	Azithromycin	1	62	5/11/2018	103.4	HEIGHT	15.3	14.9		0	1
27	6637	48	Azithromycin	1	73	4/13/2019	110	HEIGHT	16.85	14.6		0	1
27	6637	60	Azithromycin	1	83	2/4/2020	114.6	HEIGHT	18.9	17		0	1
27	6638	12	Azithromycin	0	11	5/14/2016	75	LENGTH	8.65	15		0	1
27	6638	24	Azithromycin	0	20	3/18/2017	83.5	HEIGHT	10.45	14		0	1
27	6638	48	Azithromycin	0	48	4/13/2019	98	HEIGHT	13.2	13.8		1	1
27	6639	12	Azithromycin	0	11	5/14/2016	70	HEIGHT	6.15	11.8		0	1
27	6639	24	Azithromycin	0	23	3/18/2017	75.5	HEIGHT	7.9	11.5		0	1
27	6639	36	Azithromycin	0	37	5/11/2018	81.5	HEIGHT	9.2	10.6		0	1
27	6639	60	Azithromycin	0	58	2/4/2020	96.5	HEIGHT	12.95	13.4		1	1
27	6640	0	Azithromycin	0	48	3/12/2015	106.5	HEIGHT	16.9	15.7		1	1
27	6640	36	Azithromycin	0	98	5/11/2018	123	HEIGHT	22.45	15.2		0	1
27	6640	48	Azithromycin	0	109	4/13/2019	127.6	HEIGHT	22.55	15.6		0	1
27	6640	60	Azithromycin	0	119	2/4/2020	130.5	HEIGHT	25.35	16.2		0	1
27	6641	12	Azithromycin	1	4	5/14/2016	60.8	LENGTH	5.15	12.5		0	1
27	6641	24	Azithromycin	1	14	3/18/2017	73.1	HEIGHT	8.35	16		0	1
27	6641	36	Azithromycin	1	28	5/11/2018	83.5	LENGTH	11.6	15.5		0	1
27	6641	60	Azithromycin	1	49	2/4/2020	98.9	HEIGHT	15.4	16		0	1

27	6642	0	Azithromycin	0	48	6/2/2015	108.5	HEIGHT	15.45	15.1		1	1
27	6642	12	Azithromycin	0	59	5/15/2016	112.4	HEIGHT	16.75	14.9		0	1
27	6642	36	Azithromycin	0	98	5/11/2018	122	HEIGHT	20.1	15.3		0	1
27	6642	48	Azithromycin	0	109	4/13/2019	126.6	HEIGHT	20.95	16		0	1
27	6642	60	Azithromycin	0	119	2/4/2020	131.2	HEIGHT	23.1	16.5		0	1
27	6643	0	Azithromycin	1	36	3/12/2015	83.6	HEIGHT	11.1	16.2		0	1
27	6644	12	Azithromycin	1	8	5/14/2016	60.3	LENGTH	5.5	11.7		0	1
27	6644	24	Azithromycin	1	14	3/18/2017	70.5	LENGTH	8.15	13.5		0	1
27	6644	36	Azithromycin	1	34	5/11/2018	81	HEIGHT	10.9	15.5		1	1
27	6644	60	Azithromycin	1	55	2/4/2020	94	HEIGHT	13.9	14.5		0	1
27	6645	0	Azithromycin	0	36	3/12/2015	88.5	LENGTH	13.8	17.3		1	1
27	6645	12	Azithromycin	0	39	5/14/2016	97.2	HEIGHT	16.1	16.3		1	1
27	6645	24	Azithromycin	0	56	3/18/2017	103	HEIGHT	17.5	18.5		0	1
27	6645	36	Azithromycin	0	70	5/11/2018	108.9	HEIGHT	19.35	16		0	1
27	6645	60	Azithromycin	0	91	2/4/2020	117.2	HEIGHT	21.5	16		0	1
27	6646	12	Azithromycin	1	7	5/15/2016	65.9	LENGTH	6.05	12.5	30	0	1
27	6646	24	Azithromycin	1	18	3/18/2017	75.1	HEIGHT	8.05	11.5	30	0	1
27	6650	12	Azithromycin	0	13	5/15/2016	71.9	LENGTH	7.75	13.5		1	1
27	6650	24	Azithromycin	0	24	3/18/2017	77	HEIGHT	9.45	13		1	1
27	6652	12	Azithromycin	0	24	5/15/2016	80.1	HEIGHT	9.4	12.7		0	1
27	6652	24	Azithromycin	0	33	3/18/2017	87.3	HEIGHT	12.45	15		0	1
27	6652	36	Azithromycin	0	48	5/11/2018	94.7	HEIGHT	13.8	14		1	1
27	6652	48	Azithromycin	0	59	4/13/2019	102	HEIGHT	15.75	15		0	1
27	6652	60	Azithromycin	0	69	2/4/2020	108.2	HEIGHT	17.55	14.7		0	1
27	6653	12	Azithromycin	0	27	6/13/2016	90	HEIGHT	11.7	13.5		0	1
27	6654	0	Azithromycin	1	36	3/12/2015	82.5	HEIGHT	10.75	15.2		1	1
27	6654	12	Azithromycin	1	39	5/15/2016	89.5	HEIGHT	11.95	14.5		1	1
27	6654	24	Azithromycin	1	50	3/18/2017	96.1	HEIGHT	13.3	15.5		0	1
27	6654	36	Azithromycin	1	65	5/11/2018	103.2	HEIGHT	15.05	15.7		0	1
27	6654	60	Azithromycin	1	85	2/4/2020	109.8	HEIGHT	17.3	15		0	1
27	6656	0	Azithromycin	0	9	3/12/2015	64.3	LENGTH	7.1	14.6		1	1
27	6656	12	Azithromycin	0	12	5/14/2016	77.5	LENGTH	8.95	14		0	1
27	6656	24	Azithromycin	0	36	3/18/2017	81.3	HEIGHT	10.5	13		0	1
27	6656	36	Azithromycin	0	50	5/11/2018	87.5	HEIGHT	11.7	13.2		0	1
27	6656	60	Azithromycin	0	63	2/4/2020	100.5	HEIGHT	14.45	13.6		0	1
27	6657	24	Azithromycin	1	48	3/18/2017	93	HEIGHT	11.8	14.5		1	1
27	6660	0	Azithromycin	0	12	3/12/2015	66.2	LENGTH	6.85	13.9		0	1
27	6661	12	Azithromycin	1	2	5/15/2016	60.5	HEIGHT	5.35	12.5		0	1
27	6661	24	Azithromycin	1	14	3/18/2017	70.4	LENGTH	7.75	13		0	1
27	6661	36	Azithromycin	1	28	5/11/2018	89	HEIGHT	9.95	13.2		0	1

27	6663	12	Azithromycin	1	1	5/14/2016	52.6	HEIGHT	3.9	11.5		1	1
27	6663	24	Azithromycin	1	12	3/18/2017	64.6	LENGTH	6.25	12		0	1
27	6663	36	Azithromycin	1	26	5/11/2018	76.4	LENGTH	7.75	12.3		1	1
27	6663	48	Azithromycin	1	37	4/13/2019	83.6	LENGTH	8.85	12		0	1
27	6663	60	Azithromycin	1	47	2/4/2020	89.3	HEIGHT	11.4	14		1	1
27	6664	0	Azithromycin	0	54	3/12/2015	108.4	HEIGHT	16.8	15.4		1	1
27	6664	12	Azithromycin	0	67	5/14/2016	114.6	HEIGHT	18	14		0	1
27	6664	24	Azithromycin	0	96	3/18/2017	118.2	HEIGHT	20.55	15		0	1
27	6664	36	Azithromycin	0	110	5/11/2018	125.3	HEIGHT	22.65	15		0	1
27	6665	24	Azithromycin	1	41	3/18/2017	91.5	HEIGHT	12.7	15	30	1	1
27	6666	0	Azithromycin	1	9	3/12/2015	61.2	LENGTH	6.1	13.4		0	1
27	6666	12	Azithromycin	1	18	5/14/2016	76.4	LENGTH	8.4	13		1	1
27	6666	24	Azithromycin	1	29	3/18/2017	82.3	HEIGHT	9.45	12		0	1
27	6666	36	Azithromycin	1	43	5/11/2018	92	HEIGHT	12.25	13.5		1	1
27	6669	0	Azithromycin	1	8	3/12/2015	57.3	LENGTH	5.95	14.8		0	1
27	6669	24	Azithromycin	1	23	3/18/2017	78	HEIGHT	9.1	13.8		1	1
27	6669	36	Azithromycin	1	37	5/11/2018	85.1	HEIGHT	11.5	16		0	1
27	6669	48	Azithromycin	1	48	4/13/2019	92.1	HEIGHT	12.25	15.4		0	1
27	6669	60	Azithromycin	1	58	2/4/2020	99	HEIGHT	14.2	15.5		0	1
27	6670	0	Azithromycin	1	3	6/2/2015	95.5	HEIGHT	12.8	15.1		0	1
27	6670	12	Azithromycin	1	41	5/15/2016	102.4	HEIGHT	13.65	14.3		0	1
27	6670	24	Azithromycin	1	60	3/18/2017	108.5	HEIGHT	15.05	14		0	1
27	6670	36	Azithromycin	1	74	5/11/2018	115.1	HEIGHT	17.45	14		0	1
27	6670	48	Azithromycin	1	85	4/13/2019	121.1	HEIGHT	18.15	14		0	1
27	6670	60	Azithromycin	1	95	2/4/2020	127.7	HEIGHT	20.7	15.3		0	1
27	6672	12	Azithromycin	1	5	5/15/2016	63.6	LENGTH	6	12.5	18	0	1
27	6673	0	Azithromycin	0	52	3/12/2015	98.7	HEIGHT	14.25	14.9		1	1
27	6673	12	Azithromycin	0	61	5/14/2016	105.4	HEIGHT	16.25	14		0	1
27	6674	0	Azithromycin	1	48	3/12/2015	101.1	HEIGHT	15.1	15.8		1	1
27	6674	12	Azithromycin	1	54	6/13/2016	109	HEIGHT	17.3	15		0	1
27	6674	24	Azithromycin	1	65	3/18/2017	113.6	HEIGHT	18.5	16		0	1
27	6674	36	Azithromycin	1	96	5/11/2018	119	HEIGHT	21.05	15.2		0	1
27	6674	48	Azithromycin	1	107	4/13/2019	124.6	HEIGHT	23.45	16.6		0	1
27	6674	60	Azithromycin	1	117	2/4/2020	128.4	HEIGHT	25.25	17		0	1
27	6675	12	Azithromycin	1	15	5/15/2016	80.3	LENGTH	11.7	16.5		1	1
27	6675	48	Azithromycin	1	56	4/13/2019	99.6	HEIGHT	18.45	19		1	1
27	6676	0	Azithromycin	1	31	3/12/2015	84.1	LENGTH	10.1	14		0	1
27	6676	36	Azithromycin	1	56	5/11/2018	107.5	HEIGHT	14.3	14		0	1
27	6676	48	Azithromycin	1	67	4/13/2019	111.5	HEIGHT	15.81818182	14.3		0	1
27	6676	60	Azithromycin	1	76	2/4/2020	116	HEIGHT	18.15	16		0	1

27	6678	12	Azithromycin	0	36	5/15/2016	96	HEIGHT	13.4	15	1	1
27	6679	12	Azithromycin	1	37	5/15/2016	81.3	LENGTH	10	14	1	1
27	6679	24	Azithromycin	1	48	3/18/2017	89.5	HEIGHT	11.9	14.5	1	1
27	6679	36	Azithromycin	1	51	5/11/2018	100.3	LENGTH	14.05	14.8	1	1
27	6683	0	Azithromycin	0	10	3/12/2015	67.7	LENGTH	7.7	14.9	0	1
27	6683	12	Azithromycin	0	24	6/13/2016	77	HEIGHT	10.8	15	0	1
27	6683	24	Azithromycin	0	33	3/18/2017	85.1	HEIGHT	12.15	15	0	1
27	6683	36	Azithromycin	0	47	5/11/2018	93	HEIGHT	13.95	15	1	1
27	6683	48	Azithromycin	0	58	4/13/2019	99.5	HEIGHT	14.85	14.5	1	1
27	6683	60	Azithromycin	0	68	2/4/2020	105.4	HEIGHT	17	14.6	0	1
27	6684	0	Azithromycin	1	48	3/12/2015	93	HEIGHT	13.05	15.2	1	1
27	6684	12	Azithromycin	1	61	5/15/2016	100.4	HEIGHT	14.45	14.5	0	1
27	6684	24	Azithromycin	1	72	3/18/2017	106.3	HEIGHT	16.25	15	0	1
27	6684	36	Azithromycin	1	86	5/11/2018	112.6	HEIGHT	18.9	15.4	0	1
27	6684	48	Azithromycin	1	97	4/13/2019	117.2	HEIGHT	21.31818182	15.5	0	1
27	6684	60	Azithromycin	1	107	2/4/2020	120.9	HEIGHT	22.9	16.5	0	1
27	6685	0	Azithromycin	0	54	3/12/2015	108.6	HEIGHT	16	15.4	0	1
27	6685	24	Azithromycin	0	77	3/18/2017	122.1	HEIGHT	20.8	16	0	1
27	6685	36	Azithromycin	0	98	5/11/2018	128.6	HEIGHT	22.55	15.7	0	1
27	6685	48	Azithromycin	0	109	4/13/2019	135	HEIGHT	24.3	16.6	0	1
27	6686	0	Azithromycin	1	42	3/12/2015	94.5	HEIGHT	13.65	15.5	0	1
27	6686	12	Azithromycin	1	54	6/13/2016	104.8	HEIGHT	16.3	15	1	1
27	6686	24	Azithromycin	1	65	3/18/2017	110.1	HEIGHT	17.55	15	0	1
27	6686	36	Azithromycin	1	80	5/11/2018	117.1	HEIGHT	19.3	15	0	1
27	6686	48	Azithromycin	1	91	4/13/2019	121.5	HEIGHT	20.65	15	0	1
27	6686	60	Azithromycin	1	100	2/4/2020	125.7	HEIGHT	22.25	15.7	0	1
27	6688	0	Azithromycin	0	18	3/12/2015	66.8	LENGTH	6.65	13.2	0	1
27	6688	12	Azithromycin	1	28	6/13/2016	79.3	LENGTH	9.05	13.5	0	1
27	6688	24	Azithromycin	1	26	3/18/2017	88.1	HEIGHT	11	14.5	0	1
27	6688	36	Azithromycin	1	48	5/11/2018	96.5	HEIGHT	12.55	13.5	0	1
27	6688	48	Azithromycin	1	58	4/13/2019	103.7	HEIGHT	13.5	14.5	1	1
27	6689	0	Azithromycin	0	42	3/12/2015	86.4	HEIGHT	13.35	17.3	0	1
27	6689	12	Azithromycin	0	52	6/13/2016	96.5	HEIGHT	15.25	16	0	1
27	6689	24	Azithromycin	0	62	3/18/2017	102	HEIGHT	16.85	16	0	1
27	6689	36	Azithromycin	0	72	5/11/2018	110.4	HEIGHT	18.75	15.6	0	1
27	6689	48	Azithromycin	0	83	4/13/2019	117	HEIGHT	20.2	15.9	0	1
27	6691	12	Azithromycin	1	9	5/14/2016	67.8	LENGTH	6.65	12.9	0	1
27	6691	24	Azithromycin	1	20	3/18/2017	77.3	HEIGHT	9.75	14.5	0	1
27	6691	36	Azithromycin	1	35	5/11/2018	86.2	HEIGHT	12	16	0	1
27	6691	48	Azithromycin	1	46	4/13/2019	92.6	HEIGHT	12.4	15.5	1	1

27	6691	60	Azithromycin	1	56	2/4/2020	97.5	HEIGHT	14.5	15.6		1	1
27	6692	12	Azithromycin	0	10	5/14/2016	71.4	LENGTH	6.7	12		0	1
27	6692	24	Azithromycin	0	20	3/18/2017	78.8	LENGTH	8.5	11.5		0	1
27	6692	36	Azithromycin	0	34	5/11/2018	88.1	HEIGHT	11.2	14		0	1
27	6692	60	Azithromycin	0	55	2/4/2020	98.6	HEIGHT	14.3	14		1	1
27	6695	0	Azithromycin	1	11	3/12/2015	72.3	LENGTH	7.85	13.5		0	1
27	6695	12	Azithromycin	1	24	5/15/2016	84.6	LENGTH	11.2	15.5		1	1
27	6695	24	Azithromycin	1	35	3/18/2017	93.5	HEIGHT	13.2	14.5		1	1
27	6695	36	Azithromycin	1	52	5/11/2018	102.9	HEIGHT	16.1	15.2		0	1
27	6695	48	Azithromycin	1	63	4/13/2019	111	HEIGHT	17.65	16.3		0	1
27	6695	60	Azithromycin	1	73	2/4/2020	116.6	HEIGHT	19.95	16.2		0	1
27	6697	12	Azithromycin	0	6	5/15/2016	67.3	LENGTH	6.45	13		0	1
27	6697	24	Azithromycin	0	18	3/18/2017	74.5	HEIGHT	8.4	14.5		1	1
27	6697	48	Azithromycin	0	43	4/13/2019	90.1	HEIGHT	11.35	13.6		1	1
27	6698	24	Azithromycin	0	11	3/18/2017	71.3	HEIGHT	7	13.7	42	1	1
27	6698	36	Azithromycin	0	26	5/11/2018	82.3	HEIGHT	9.45	14	42	0	1
27	6699	0	Azithromycin	1	10	6/2/2015	71.9	LENGTH	7.55	13.5		1	1
27	6699	24	Azithromycin	1	42	3/18/2017	83	HEIGHT	10.25	13.6		0	1
27	6699	36	Azithromycin	1	56	5/11/2018	93	HEIGHT	12.5	15		0	1
27	6699	48	Azithromycin	1	67	4/13/2019	102.3	HEIGHT	15.1	15.7		0	1
27	6699	60	Azithromycin	1	77	2/4/2020	108.3	HEIGHT	17.35	15.6		0	1
27	6701	24	Azithromycin	0	25	3/18/2017	85	HEIGHT	10.1	13		1	1
27	6703	24	Azithromycin	1	3	3/18/2017	62.3	LENGTH	6.4	13	42	1	1
27	6703	36	Azithromycin	1	18	5/11/2018	77.3	LENGTH	8.5	11.6	42	0	1
27	6705	12	Azithromycin	1	9	5/15/2016	71.7	HEIGHT	7.65	12.5		0	1
27	6705	36	Azithromycin	1	35	5/11/2018	88.6	HEIGHT	13.4	15		0	1
27	6705	60	Azithromycin	1	56	2/4/2020	106	HEIGHT	17.5	15.7		0	1
27	6706	0	Azithromycin	0	6	6/2/2015	65.7	LENGTH	6.5	13.3		0	1
27	6706	12	Azithromycin	0	18	5/15/2016	75.2	LENGTH	7.55	13		0	1
27	6706	24	Azithromycin	0	28	3/18/2017	81.2	HEIGHT	9.95	14.5		0	1
27	6706	36	Azithromycin	0	42	5/11/2018	89	HEIGHT	12.2	14.5		0	1
27	6706	48	Azithromycin	0	53	4/13/2019	95.2	HEIGHT	13.25	14.9		0	1
27	6707	12	Azithromycin	1	49	5/15/2016	99.3	HEIGHT	14.3	15.5		0	1
27	6707	24	Azithromycin	1	51	3/18/2017	105	HEIGHT	16.15	15.5		1	1
27	6709	0	Azithromycin	0	48	3/12/2015	88.4	LENGTH	12.35	15.5		0	1
27	6709	12	Azithromycin	0	38	5/14/2016	96.1	HEIGHT	14.5	14.5		1	1
27	6709	36	Azithromycin	0	62	5/11/2018	109.4	HEIGHT	17.55	15		0	1
27	6709	48	Azithromycin	0	73	4/13/2019	113.6	HEIGHT	18.36363636	14.6		0	1
27	6715	0	Azithromycin	0	12	3/12/2015	68.3	LENGTH	6.1	11.8		0	1
27	6715	12	Azithromycin	0	18	5/14/2016	79.1	HEIGHT	9.85	13.7		1	1

27	6715	24	Azithromycin	0	34	3/18/2017	85.4	HEIGHT	14.05	16			1	1
27	6715	36	Azithromycin	0	48	5/11/2018	95	HEIGHT	16.65	15.5			1	1
27	6715	48	Azithromycin	0	59	4/13/2019	104.5	HEIGHT	18.3	15.7			0	1
27	6715	60	Azithromycin	0	69	2/4/2020	109	HEIGHT	20.9	16			0	1
27	6719	24	Azithromycin	0	10	3/18/2017	71.4	LENGTH	8.1	13.5			1	1
27	6719	36	Azithromycin	0	23	5/11/2018	79.5	HEIGHT	9.2	12			1	1
27	6719	48	Azithromycin	0	34	4/13/2019	87.2	HEIGHT	12.15	14			0	1
27	6719	60	Azithromycin	0	44	2/4/2020	94.3	HEIGHT	15.75	16			0	1
27	6722	0	Azithromycin	0	30	6/2/2015	87.4	HEIGHT	10.2	13.5			1	1
27	6722	12	Azithromycin	0	33	6/13/2016	96	HEIGHT	13.25	14			0	1
27	6722	24	Azithromycin	0	44	3/18/2017	102.1	HEIGHT	14.6	14.5			0	1
27	6722	36	Azithromycin	0	78	5/11/2018	111.2	HEIGHT	17.1	15.2			0	1
27	6722	48	Azithromycin	0	88	4/13/2019	117	HEIGHT	18.65	15			0	1
27	6722	60	Azithromycin	0	98	2/4/2020	121.7	HEIGHT	21.45	13.3			0	1
27	6723	0	Azithromycin	0	36	3/12/2015	85.6	LENGTH	11.8	15			0	1
27	6723	12	Azithromycin	0	36	5/15/2016	91.3	HEIGHT	13.35	14			1	1
27	6723	24	Azithromycin	0	55	3/18/2017	96	HEIGHT	14.15	14			1	1
27	6723	36	Azithromycin	0	69	5/11/2018	103.3	LENGTH	16.35	14.2			0	1
27	6723	48	Azithromycin	0	80	4/13/2019	107.7	HEIGHT	17.63636364	13.7			0	1
27	6725	12	Azithromycin	1	10	5/15/2016	89.6	HEIGHT	12.1	14			1	1
27	6725	36	Azithromycin	1	37	5/11/2018	90.5	HEIGHT	11.7	13.4			0	1
27	6725	48	Azithromycin	1	48	4/13/2019	101	HEIGHT	13.4	15.2			0	1
27	6725	60	Azithromycin	1	58	2/4/2020	105.9	HEIGHT	14.7	14.5			0	1
27	6730	12	Azithromycin	0	24	6/13/2016	81	HEIGHT	7.35	13.5			1	1
27	6731	12	Azithromycin	0	2	5/14/2016	64.1	HEIGHT	6.85	14.5			0	1
27	6731	24	Azithromycin	0	13	3/18/2017	74.1	HEIGHT	9.25	15			0	1
27	6731	36	Azithromycin	0	28	5/11/2018	84.1	HEIGHT	10.8	14.5			1	1
27	6731	48	Azithromycin	0	39	4/13/2019	89.2	HEIGHT	11.95	14.6			1	1
27	6731	60	Azithromycin	0	49	2/4/2020	95.4	HEIGHT	13.8	14.7			0	1
27	6733	0	Azithromycin	0	4	3/12/2015	54.8	LENGTH	4.7	12.3			0	1
27	6733	12	Azithromycin	0	12	6/13/2016	72.5	LENGTH	8.05	14			0	1
27	6733	24	Azithromycin	0	23	3/18/2017	78	HEIGHT	8.9	14.5			0	1
27	6733	48	Azithromycin	0	50	4/13/2019	88.3	HEIGHT	12.55	15.2			0	1
27	6733	60	Azithromycin	0	60	2/4/2020	96.2	HEIGHT	14	16			0	1
27	6735	0	Azithromycin	0	36	6/2/2015	92.5	HEIGHT	14.35	15.5			1	1
27	6735	12	Azithromycin	0	49	5/14/2016	100.1	HEIGHT	16.05	15			0	1
27	6735	24	Azithromycin	0	72	3/18/2017	106.4	HEIGHT	17.75	15.5			0	1
27	6735	36	Azithromycin	0	86	5/11/2018	113	HEIGHT	19.4	15			0	1
27	6736	0	Azithromycin	1	48	3/12/2015	95.7	HEIGHT	12.15	13.2			0	1
27	6736	24	Azithromycin	1	72	3/18/2017	105.1	HEIGHT	14.55	13			0	1

27	6736	36	Azithromycin	1	86	5/11/2018	110.9	HEIGHT	15.7	12.6		0	1
27	6736	48	Azithromycin	1	97	4/13/2019	114	HEIGHT	18.09090909	14.3		0	1
27	6737	0	Azithromycin	0	36	3/12/2015	96.5	HEIGHT	14.6	16		0	1
27	6737	12	Azithromycin	0	45	5/15/2016	105.7	HEIGHT	16.5	15		0	1
27	6737	24	Azithromycin	0	56	3/18/2017	110.5	HEIGHT	17.4	14.5		1	1
27	6737	36	Azithromycin	0	84	5/11/2018	116	HEIGHT	19.1	14		0	1
27	6738	0	Azithromycin	1	42	3/12/2015	90.9	HEIGHT	13.45	16.5		1	1
27	6738	12	Azithromycin	1	45	5/15/2016	99.3	HEIGHT	14.85	14.6		0	1
27	6738	24	Azithromycin	0	56	3/18/2017	104	HEIGHT	16.8	15.5		0	1
27	6738	36	Azithromycin	0	71	5/11/2018	112	HEIGHT	18.15	15		0	1
27	6738	48	Azithromycin	0	82	4/13/2019	116.5	HEIGHT	19.2	14.8		0	1
27	6738	60	Azithromycin	0	91	2/4/2020	120.8	HEIGHT	21.65	15.7		0	1
27	6741	0	Azithromycin	0	48	3/12/2015	102.2	HEIGHT	15.35	16.1		0	1
27	6741	12	Azithromycin	0	62	5/15/2016	107.8	HEIGHT	17.75	15		1	1
27	6741	36	Azithromycin	0	96	5/11/2018	119.5	HEIGHT	20.6	15		0	1
27	6742	0	Azithromycin	1	30	3/12/2015	87	HEIGHT	9.3	13.2		1	1
27	6742	12	Azithromycin	0	33	6/13/2016	98	HEIGHT	11.6	13		0	1
27	6742	24	Azithromycin	0	44	3/18/2017	103.1	HEIGHT	12.6	13.2		0	1
27	6742	36	Azithromycin	0	59	5/11/2018	109.9	HEIGHT	13.7	12.7		0	1
27	6742	48	Azithromycin	0	70	4/13/2019	115.4	HEIGHT	15.05	13.2		0	1
27	6742	60	Azithromycin	0	79	2/4/2020	119.7	HEIGHT	17.15	14		0	1
27	6743	12	Azithromycin	1	9	5/15/2016	67.4	LENGTH	6.4	12.5		0	1
27	6743	24	Azithromycin	1	20	3/18/2017	77.1	HEIGHT	9.45	14		0	1
27	6743	36	Azithromycin	1	34	5/11/2018	88.1	HEIGHT	10.85	13.9		1	1
27	6743	48	Azithromycin	1	45	4/13/2019	94.7	HEIGHT	13	13.9		1	1
27	6743	60	Azithromycin	1	55	2/4/2020	102.5	HEIGHT	14.85	14.3		1	1
27	6745	0	Azithromycin	1	54	3/12/2015	101.4	HEIGHT	16.6	16		0	1
27	6745	12	Azithromycin	1	56	5/14/2016	106.9	HEIGHT	18.3	15		1	1
27	6745	48	Azithromycin	1	117	4/13/2019	119.1	HEIGHT	23.36363636	17.4		0	1
27	6745	60	Azithromycin	1	127	2/4/2020	123.8	HEIGHT	25.65	17		0	1
27	6746	12	Azithromycin	1	6	5/15/2016	71.1	HEIGHT	8.2	14	30	0	1
27	6746	24	Azithromycin	1	14	3/18/2017	77.1	HEIGHT	9.5	14	30	1	1
27	6747	24	Azithromycin	0	38	3/18/2017	79.1	HEIGHT	12.5	18.4		1	1
27	6748	24	Azithromycin	0	48	3/18/2017	95	HEIGHT	12.45	14		1	1
27	6750	12	Azithromycin	0	9	5/15/2016	72.6	HEIGHT	8.75	15		0	1
27	6750	24	Azithromycin	0	19	3/18/2017	80	HEIGHT	10.4	15		0	1
27	6750	36	Azithromycin	0	34	5/11/2018	87.5	HEIGHT	12.6	15		1	1
27	6750	60	Azithromycin	0	54	2/4/2020	103.3	HEIGHT	15.8	15.3		0	1
27	6753	0	Azithromycin	0	48	3/12/2015	99.6	HEIGHT	16.65	18.1		0	1
27	6753	12	Azithromycin	0	61	5/14/2016	110.8	HEIGHT	19	17.5		0	1

27	6753	24	Azithromycin	0	72	3/18/2017	118	HEIGHT	20.95	17.5		0	1
27	6753	36	Azithromycin	0	90	5/11/2018	125.6	HEIGHT	23.2	16.5		0	1
27	6753	48	Azithromycin	0	101	4/13/2019	131.2	HEIGHT	24.9	16.6		0	1
27	6753	60	Azithromycin	0	110	2/4/2020	135.3	HEIGHT	27.2	17		0	1
27	6754	0	Azithromycin	0	12	3/12/2015	80.2	LENGTH	10.05	14.7		0	1
27	6754	12	Azithromycin	0	21	5/15/2016	90.4	HEIGHT	13	14.5		1	1
27	6754	24	Azithromycin	0	32	3/18/2017	97	HEIGHT	14.2	15.5		0	1
27	6754	36	Azithromycin	0	57	5/11/2018	103.5	HEIGHT	15.35	14.3		0	1
27	6755	0	Azithromycin	0	54	6/2/2015	104.2	HEIGHT	13.4	13.9		1	1
27	6755	12	Azithromycin	0	56	6/13/2016	108.5	HEIGHT	15.2	15		0	1
27	6755	24	Azithromycin	0	68	3/18/2017	113.4	HEIGHT	16.4	13.5		0	1
27	6755	36	Azithromycin	0	82	5/11/2018	117.9	HEIGHT	18	14		0	1
27	6755	48	Azithromycin	0	93	4/13/2019	122.4	HEIGHT	19.75	14.9		0	1
27	6756	0	Azithromycin	1	16	3/12/2015	72.3	LENGTH	7.95	14.2		1	1
27	6756	24	Azithromycin	1	35	3/18/2017	92.5	HEIGHT	12.8	14.5		1	1
27	6756	36	Azithromycin	1	51	5/11/2018	102	HEIGHT	15.1	15		1	1
27	6756	48	Azithromycin	1	62	4/13/2019	109.8	HEIGHT	16.8	15.4		0	1
27	6756	60	Azithromycin	1	60	2/4/2020	116.1	HEIGHT	19.5	16.2		0	1
27	6757	0	Azithromycin	1	48	3/12/2015	91.2	HEIGHT	11.7	14.8		0	1
27	6757	24	Azithromycin	1	62	3/18/2017	105	HEIGHT	14.95	14		0	1
27	6757	36	Azithromycin	1	84	5/11/2018	110.4	HEIGHT	16.85	15		0	1
27	6757	48	Azithromycin	1	94	4/13/2019	115.4	HEIGHT	17.4	14.7		0	1
27	6757	60	Azithromycin	1	104	2/4/2020	118.3	HEIGHT	18.4	15		0	1
27	6758	0	Azithromycin	1	18	3/12/2015	72.8	LENGTH	7.35	12.9		0	1
27	6758	24	Azithromycin	1	14	3/18/2017	90	HEIGHT	11.6	13.5		1	1
27	6758	36	Azithromycin	1	53	5/11/2018	98.6	HEIGHT	13.5	13.8		0	1
27	6758	48	Azithromycin	1	64	4/13/2019	106.9	HEIGHT	15.5	15		0	1
27	6758	60	Azithromycin	1	73	2/4/2020	111	HEIGHT	17.4	15		0	1
27	6760	0	Azithromycin	0	12	3/12/2015	72.7	LENGTH	7.5	13.4	12	0	1
27	6763	0	Azithromycin	1	24	3/12/2015	83.7	HEIGHT	11.3	14.8		0	1
27	6764	12	Azithromycin	1	56	5/15/2016	96.8	HEIGHT	15.55	15.5		1	1
27	6764	24	Azithromycin	1	61	3/18/2017	105.9	HEIGHT	16.65	15.5		0	1
27	6764	36	Azithromycin	1	76	5/11/2018	113.1	HEIGHT	19.15	15.8		0	1
27	6764	48	Azithromycin	1	87	4/13/2019	118.3	HEIGHT	21.1	16		0	1
27	6764	60	Azithromycin	1	96	2/4/2020	122.4	HEIGHT	22.1	15.9		0	1
27	6765	0	Azithromycin	0	36	3/12/2015	87.5	HEIGHT	12.5	15.5		0	1
27	6765	12	Azithromycin	0	62	6/13/2016	96	HEIGHT	14.3	15		0	1
27	6765	24	Azithromycin	0	62	3/18/2017	105.1	HEIGHT	15.4	14		0	1
27	6765	36	Azithromycin	0	76	5/11/2018	106.5	HEIGHT	16.9	14.3		0	1
27	6765	48	Azithromycin	0	87	4/13/2019	111.2	HEIGHT	17.7	16		0	1

27	6765	60	Azithromycin	0	97	2/4/2020	115.3	HEIGHT	19.55	15.8	0	1
27	6767	0	Azithromycin	0	48	3/12/2015	100.2	HEIGHT	13.2	14.8	1	1
27	6767	12	Azithromycin	0	63	6/13/2016	105.6	HEIGHT	14.8	14	0	1
27	6767	36	Azithromycin	0	89	5/11/2018	114	HEIGHT	17.25	14.5	0	1
27	6767	48	Azithromycin	0	100	4/13/2019	117.5	HEIGHT	18.59090909	14.7	0	1
27	6767	60	Azithromycin	0	109	2/4/2020	120.9	HEIGHT	20.4	15.5	0	1
27	6769	24	Azithromycin	1	83	3/18/2017	118	HEIGHT	19.65	14.5	0	1
27	6772	0	Azithromycin	0	12	3/12/2015	74.8	LENGTH	8.5	14.5	1	1
27	6772	12	Azithromycin	0	30	6/13/2016	84.4	HEIGHT	10.7	14	0	1
27	6772	24	Azithromycin	0	41	3/18/2017	89.5	HEIGHT	11.5	13.3	1	1
27	6772	36	Azithromycin	0	56	5/11/2018	97.1	HEIGHT	13.55	13.8	0	1
27	6772	48	Azithromycin	0	67	4/13/2019	103.5	HEIGHT	14.25	13.6	0	1
27	6775	0	Azithromycin	0	36	3/12/2015	84.8	HEIGHT	11.5	15.7	0	1
27	6775	24	Azithromycin	0	54	3/18/2017	103.5	HEIGHT	16.5	16.5	1	1
27	6775	36	Azithromycin	0	68	5/11/2018	111.4	HEIGHT	18.25	16	0	1
27	6775	48	Azithromycin	0	79	4/13/2019	116.9	HEIGHT	18.7	15.6	0	1
27	6775	60	Azithromycin	0	89	2/4/2020	121.8	HEIGHT	21	15	0	1
27	6776	0	Azithromycin	1	48	3/12/2015	90.2	HEIGHT	12.7	15.1	1	1
27	6776	12	Azithromycin	1	42	5/14/2016	98.1	HEIGHT	14.6	15	0	1
27	6776	24	Azithromycin	1	72	3/18/2017	104	HEIGHT	16.95	15.5	0	1
27	6776	36	Azithromycin	1	86	5/11/2018	109.6	HEIGHT	17.95	15	0	1
27	6776	60	Azithromycin	1	107	2/4/2020	118.5	HEIGHT	21.55	16	0	1
27	6778	12	Azithromycin	1	9	5/14/2016	67.7	LENGTH	6.6	13.8	0	1
27	6778	24	Azithromycin	1	20	3/18/2017	76.5	HEIGHT	9.1	14.5	0	1
27	6778	36	Azithromycin	1	35	5/11/2018	86.5	HEIGHT	12.4	15	1	1
27	6778	60	Azithromycin	1	56	2/4/2020	97.6	HEIGHT	14.9	15.8	0	1
27	6779	0	Azithromycin	1	48	3/12/2015	105.2	HEIGHT	15.05	15	0	1
27	6779	36	Azithromycin	1	98	5/11/2018	124.4	HEIGHT	20.35	14.4	0	1
27	6779	48	Azithromycin	1	109	4/13/2019	129	HEIGHT	22	15.6	0	1
27	6779	60	Azithromycin	1	119	2/4/2020	132.3	HEIGHT	24.15	15	0	1
27	6780	0	Azithromycin	1	36	3/12/2015	95	HEIGHT	13.1	15.6	0	1
27	6780	12	Azithromycin	1	47	5/14/2016	102	HEIGHT	13.55	14.2	0	1
27	6780	24	Azithromycin	1	56	3/18/2017	107.4	HEIGHT	15.8	15	1	1
27	6780	36	Azithromycin	1	71	5/11/2018	115	HEIGHT	17.35	14.5	0	1
27	6780	48	Azithromycin	1	82	4/13/2019	119.4	HEIGHT	19.68181818	14	0	1
27	6780	60	Azithromycin	1	91	2/4/2020	124.9	HEIGHT	22.2	16	0	1
27	6781	24	Azithromycin	1	28	3/18/2017	82.4	HEIGHT	10.75	14	1	1
27	6782	24	Azithromycin	0	40	3/18/2017	90	HEIGHT	11.6	13.8	1	1
27	6782	36	Azithromycin	0	55	5/11/2018	98.5	HEIGHT	13.55	14	1	1
27	6783	0	Azithromycin	0	36	3/12/2015	96.7	HEIGHT	15	15.7	0	1

27	6783	12	Azithromycin	0	47	5/14/2016	102	HEIGHT	15.15	14		0	1
27	6783	24	Azithromycin	0	56	3/18/2017	108.1	HEIGHT	17.45	14.5		0	1
27	6783	36	Azithromycin	0	71	5/11/2018	115.1	HEIGHT	18.95	15		0	1
27	6783	48	Azithromycin	0	82	4/13/2019	119.5	HEIGHT	20.18181818	14		0	1
27	6783	60	Azithromycin	0	91	2/4/2020	123.5	HEIGHT	21.6	15.5		0	1
27	6785	0	Azithromycin	0	54	3/12/2015	115.7	HEIGHT	19.2	15.5		1	1
27	6785	12	Azithromycin	0	67	5/15/2016	123.3	HEIGHT	21.2	14.5		0	1
27	6785	24	Azithromycin	0	78	3/18/2017	128	HEIGHT	24.35	15.5		0	1
27	6785	48	Azithromycin	0	103	4/13/2019	138.5	HEIGHT	28.05	17		0	1
27	6786	0	Azithromycin	1	24	3/12/2015	69.8	LENGTH	6.95	11.9		1	1
27	6786	12	Azithromycin	1	30	6/13/2016	81.5	HEIGHT	9.45	12		0	1
27	6786	24	Azithromycin	1	41	3/18/2017	88	HEIGHT	10.25	12		0	1
27	6788	0	Azithromycin	0	36	3/12/2015	79	LENGTH	9.1	13.5		0	1
27	6788	24	Azithromycin	0	53	3/18/2017	88.4	HEIGHT	11.9	14.5		1	1
27	6789	0	Azithromycin	0	30	3/12/2015	79.5	HEIGHT	9.35	13.5		0	1
27	6789	12	Azithromycin	0	36	5/15/2016	86.7	HEIGHT	10.9	13.5		1	1
27	6789	24	Azithromycin	0	47	3/18/2017	93.6	HEIGHT	13.15	14		1	1
27	6789	36	Azithromycin	0	62	5/11/2018	100.3	HEIGHT	14.75	13.3		0	1
27	6789	48	Azithromycin	0	73	4/13/2019	106.6	HEIGHT	16	14.3		0	1
27	6789	60	Azithromycin	0	82	2/4/2020	111.6	HEIGHT	17.55	15.7		0	1
27	6790	0	Azithromycin	0	30	3/12/2015	84.3	HEIGHT	11.35	15		1	1
27	6790	12	Azithromycin	0	42	6/13/2016	93	HEIGHT	13.25	14		0	1
27	6790	24	Azithromycin	0	53	3/18/2017	98.4	HEIGHT	14.25	14.5		0	1
27	6790	36	Azithromycin	0	68	5/11/2018	104	HEIGHT	15.4	13.6		0	1
27	6790	48	Azithromycin	0	79	4/13/2019	110.1	HEIGHT	16.15	14.2		0	1
27	6790	60	Azithromycin	0	88	2/4/2020	114.9	HEIGHT	18.55	14.4		0	1
27	6791	12	Azithromycin	1	1	5/14/2016	50.7	LENGTH	3.4	10.5	30	1	1
27	6792	0	Azithromycin	1	54	3/12/2015	107.4	HEIGHT	17.35	17.3		1	1
27	6792	12	Azithromycin	1	63	5/14/2016	114.3	HEIGHT	19.25	16.2		0	1
27	6792	24	Azithromycin	1	74	3/18/2017	119.3	HEIGHT	21.1	17		0	1
27	6792	36	Azithromycin	1	98	5/11/2018	124.8	HEIGHT	23.9	18		0	1
27	6792	48	Azithromycin	1	109	4/13/2019	128.9	HEIGHT	26.15	18		0	1
27	6792	60	Azithromycin	1	119	2/4/2020	132.2	HEIGHT	27.25	19		0	1
27	6794	0	Azithromycin	1	36	3/12/2015	93	HEIGHT	13.1	15		0	1
27	6794	24	Azithromycin	1	83	3/18/2017	104	HEIGHT	15.15	14		0	1
27	6794	36	Azithromycin	1	97	5/11/2018	110.1	HEIGHT	16.7	14		0	1
27	6794	48	Azithromycin	1	108	4/13/2019	115	HEIGHT	18.4	14.5		0	1
27	6794	60	Azithromycin	1	118	2/4/2020	118.9	HEIGHT	20	15.6		0	1
27	6795	0	Azithromycin	1	36	6/2/2015	87.2	HEIGHT	10.95	14.5		1	1
27	6795	12	Azithromycin	1	49	5/15/2016	92.5	HEIGHT	12.15	15		1	1

27	6795	24	Azithromycin	1	60	3/18/2017	100	HEIGHT	14.1	14.5		0	1
27	6795	36	Azithromycin	1	74	5/11/2018	106.5	HEIGHT	16.4	15.1		0	1
27	6796	0	Azithromycin	1	30	3/12/2015	88.9	HEIGHT	11.95	15.3		0	1
27	6796	36	Azithromycin	1	88	5/11/2018	112.7	HEIGHT	17.65	15		0	1
27	6796	48	Azithromycin	1	98	4/13/2019	117.7	HEIGHT	19.2	11.4		0	1
27	6796	60	Azithromycin	1	108	2/4/2020	123	HEIGHT	20.95	15.7		0	1
27	6798	24	Azithromycin	0	9	3/18/2017	74.3	LENGTH	9.5	15		1	1
27	6798	36	Azithromycin	0	24	5/11/2018	82.5	HEIGHT	10.75	14		0	1
27	6798	48	Azithromycin	0	35	4/13/2019	88.5	HEIGHT	12.75	15.5		0	1
27	6801	0	Azithromycin	1	30	3/12/2015	86.2	HEIGHT	12.8	16.7		0	1
27	6802	0	Azithromycin	1	12	3/12/2015	70.7	LENGTH	8.2	14.5		1	1
27	6802	12	Azithromycin	1	24	5/14/2016	80.4	LENGTH	10.3	14.6		0	1
27	6802	24	Azithromycin	1	36	3/18/2017	88	HEIGHT	12.75	15.4		0	1
27	6802	36	Azithromycin	1	50	5/11/2018	97	HEIGHT	15.25	15.3		1	1
27	6802	48	Azithromycin	1	61	4/13/2019	104.3	HEIGHT	16.85	15.5		0	1
27	6806	0	Azithromycin	0	54	3/12/2015	104.1	HEIGHT	17.75	17.4		1	1
27	6806	24	Azithromycin	0	68	3/18/2017	119.1	HEIGHT	21.9	17.3		0	1
27	6806	36	Azithromycin	0	83	5/11/2018	124.5	HEIGHT	24.35	17		0	1
27	6806	48	Azithromycin	0	94	4/13/2019	129.5	HEIGHT	25.55	18		0	1
27	6806	60	Azithromycin	0	103	2/4/2020	134.2	HEIGHT	28.4	16.4		0	1
27	6808	12	Azithromycin	0	42	5/14/2016	93.5	HEIGHT	12.55	14.3		1	1
27	6809	12	Azithromycin	0	24	5/14/2016	90.1	HEIGHT	12.7	14		1	1
27	6810	0	Azithromycin	0	12	3/12/2015	68.5	LENGTH	7	13.3		0	1
27	6810	12	Azithromycin	0	18	6/13/2016	79.4	HEIGHT	10.3	15.5		0	1
27	6810	24	Azithromycin	0	34	3/18/2017	86	HEIGHT	12.25	16		1	1
27	6810	36	Azithromycin	0	48	5/11/2018	95	HEIGHT	14.75	16.5		0	1
27	6810	48	Azithromycin	0	59	4/13/2019	103	HEIGHT	16.4	16		1	1
27	6810	60	Azithromycin	0	69	2/4/2020	107.7	HEIGHT	17.75	15.5		0	1
27	6816	12	Azithromycin	0	0	5/14/2016	55.4	LENGTH	5.35	12.5	24	1	1
27	8056	60	Azithromycin	0	36	2/4/2020	88.2	HEIGHT	12.8	12.5		1	1
27	8066	36	Azithromycin	1	5	5/11/2018	62.4	LENGTH	5.5	11.7		1	1
27	8066	48	Azithromycin	1	16	4/13/2019	71.9	HEIGHT	8	13.3		0	1
27	8066	60	Azithromycin	1	26	2/4/2020	80.1	HEIGHT	9.6	13		0	1
27	8071	60	Azithromycin	0	1	2/4/2020	51.9	LENGTH	4.3	11		1	1
27	8073	60	Azithromycin	1	44	2/4/2020	92.4	HEIGHT	13.7	16.5		1	1
27	8087	48	Azithromycin	1	7	4/13/2019	64.4	LENGTH	7.85	14.3		1	1
27	8087	60	Azithromycin	1	16	2/4/2020	73.2	LENGTH	9.15	15.2		0	1
27	8131	60	Azithromycin	0	34	2/4/2020	81.4	HEIGHT	9.65	13.7		1	1
27	8154	36	Azithromycin	0	56	5/11/2018	110.9	HEIGHT	18.55	16.5		1	1
27	8214	60	Azithromycin	0	14	2/4/2020	72.5	LENGTH	7.35	12		1	1

27	8226	60	Azithromycin	0	39	2/4/2020	96.3	HEIGHT	14.4	16	1	1
27	8254	48	Azithromycin	0	2	4/13/2019	57.5	LENGTH	5.1	12	1	1
27	8254	60	Azithromycin	0	12	2/4/2020	69.8	LENGTH	7.25	12.5	1	1
27	8255	36	Azithromycin	0	44	5/11/2018	92	HEIGHT	11.8	14	1	1
27	8257	36	Azithromycin	1	17	5/11/2018	76.2	HEIGHT	10	16.4	1	1
27	8257	60	Azithromycin	1	38	2/4/2020	89.2	HEIGHT	14.6	17.2	1	1
27	8307	48	Azithromycin	0	34	4/13/2019	88.2	HEIGHT	12.75	15	1	1
27	8312	36	Azithromycin	0	40	5/11/2018	87.1	HEIGHT	11.5	14.5	1	1
27	8334	60	Azithromycin	1	9	2/4/2020	69.5	HEIGHT	8.4	15	1	1
27	8414	36	Azithromycin	0	13	5/11/2018	79	HEIGHT	9.25	15.2	1	1
27	8414	60	Azithromycin	0	34	2/4/2020	88.9	HEIGHT	12.25	15.3	0	1
27	8452	36	Azithromycin	1	17	5/11/2018	75	HEIGHT	8.6	14	1	1
27	8478	36	Azithromycin	0	48	5/11/2018	88.4	HEIGHT	12.3	13.5	1	1
27	8485	48	Azithromycin	0	12	4/13/2019	72.8	LENGTH	8.45	14.5	1	1
27	8485	60	Azithromycin	0	21	2/4/2020	77.5	HEIGHT	10.85	17.3	1	1
27	8515	48	Azithromycin	1	36	4/13/2019	84.2	HEIGHT	12.1	15	1	1
27	8529	48	Azithromycin	0	36	4/13/2019	88	HEIGHT	13.3	16	1	1
27	8529	60	Azithromycin	0	46	2/4/2020	94.3	HEIGHT	14.9	15.5	1	1
27	8574	48	Azithromycin	0	109	4/13/2019	119.4	HEIGHT	21.1	15.7	0	1
27	8579	48	Azithromycin	0	7	4/13/2019	68.3	LENGTH	7.7	14	1	1
27	8579	60	Azithromycin	0	17	2/4/2020	77.5	HEIGHT	9.45	15	1	1
27	8590	48	Azithromycin	0	22	4/13/2019	81.2	HEIGHT	10	14	1	1
27	8590	60	Azithromycin	0	32	2/4/2020	87.2	HEIGHT	12	14	1	1
27	8644	48	Azithromycin	0	4	4/13/2019	64.5	LENGTH	6.95	13.9	1	1
27	8644	60	Azithromycin	0	12	2/4/2020	76.2	HEIGHT	8.8	14.3	0	1
27	8710	48	Azithromycin	0	15	4/13/2019	74.3	LENGTH	8.85	13.5	1	1
27	8710	60	Azithromycin	0	25	2/4/2020	79.8	HEIGHT	10.15	13.3	1	1
27	8732	48	Azithromycin	0	9	4/13/2019	73.4	LENGTH	7.5	13.5	1	1
27	8732	60	Azithromycin	0	19	2/4/2020	79.7	HEIGHT	9.8	14.5	0	1
27	8803	60	Azithromycin	1	45	2/4/2020	97	HEIGHT	13.7	14	1	1
27	8807	36	Azithromycin	0	10	5/11/2018	70.1	HEIGHT	7.1	14	1	1
27	8807	48	Azithromycin	0	21	4/13/2019	75.3	HEIGHT	8.65	14.3	0	1
27	8807	60	Azithromycin	0	31	2/4/2020	85	HEIGHT	11.1	14	1	1
27	8815	36	Azithromycin	1	36	5/11/2018	89.5	HEIGHT	12.3	13.5	1	1
27	8815	60	Azithromycin	1	57	2/4/2020	101.2	HEIGHT	14.65	13.1	1	1
27	8904	36	Azithromycin	0	7	5/11/2018	66.8	LENGTH	6.9	13	1	1
27	8924	36	Azithromycin	1	26	5/11/2018	85.5	HEIGHT	10.5	14.3	1	1
27	8924	48	Azithromycin	1	37	4/13/2019	92.1	HEIGHT	12.15	14.3	1	1
27	8928	48	Azithromycin	0	4	4/13/2019	62.1	LENGTH	6	12.5	1	1
27	8928	60	Azithromycin	0	13	2/4/2020	74.3	LENGTH	8.7	13.8	0	1

27	8936	36	Azithromycin	0	39	5/11/2018	100.1	HEIGHT	14.65	17	1	1
27	8940	36	Azithromycin	0	25	5/11/2018	80.5	HEIGHT	10.25	14	1	1
27	8940	48	Azithromycin	0	36	4/13/2019	69.1	HEIGHT	5.727272727	15.4	1	1
27	8956	36	Azithromycin	1	29	5/11/2018	88.5	HEIGHT	11.85	14.2	1	1
27	8956	60	Azithromycin	1	50	2/4/2020	103.6	HEIGHT	17.3	17	1	1
27	8960	48	Azithromycin	0	35	4/13/2019	86	HEIGHT	12.05	14.7	1	1
27	8997	36	Azithromycin	1	27	5/11/2018	81.6	HEIGHT	9.9	13.8	1	1
27	8997	60	Azithromycin	1	48	2/4/2020	94.5	HEIGHT	13.75	15	1	1
27	9016	36	Azithromycin	0	25	5/11/2018	82.3	LENGTH	10.6	14.8	1	1
27	9026	60	Azithromycin	1	27	2/4/2020	80.6	HEIGHT	9.4	13.3	1	1
27	9040	36	Azithromycin	1	14	5/11/2018	71.4	LENGTH	7.45	14	1	1
27	9040	60	Azithromycin	1	35	2/4/2020	86.4	HEIGHT	10.65	13.5	1	1
27	9061	48	Azithromycin	0	18	4/13/2019	76.9	HEIGHT	8.6	14.6	1	1
27	9068	48	Azithromycin	1	27	4/13/2019	84.3	LENGTH	9.95	13.5	1	1
27	9069	60	Azithromycin	1	4	2/4/2020	64	LENGTH	7.4	13.5	1	1
27	9151	60	Azithromycin	0	11	2/4/2020	70.1	HEIGHT	7.7	14	1	1
27	9156	48	Azithromycin	1	5	4/13/2019	69.3	LENGTH	9.25	16	1	1
27	9156	60	Azithromycin	1	18	2/4/2020	73.3	HEIGHT	10.25	15.8	0	1
27	9169	48	Azithromycin	0	10	4/13/2019	72.9	HEIGHT	8.55	15.3	1	1
27	9169	60	Azithromycin	0	20	2/4/2020	82.3	HEIGHT	11.1	15.5	1	1
27	9195	48	Azithromycin	0	7	4/13/2019	65.8	LENGTH	6.3	13	1	1
27	9285	60	Azithromycin	1	5	2/4/2020	63.3	LENGTH	7.05	13.4	1	1
27	9306	60	Azithromycin	1	14	2/4/2020	74.4	HEIGHT	9.3	15	1	1
27	9335	36	Azithromycin	0	8	5/11/2018	68.3	LENGTH	8.2	14.4	1	1
27	9350	48	Azithromycin	1	23	4/13/2019	73.8	LENGTH	8.4	13.2	1	1
27	9385	60	Azithromycin	0	44	2/4/2020	96.4	HEIGHT	14.3	14.3	1	1
27	9417	36	Azithromycin	1	3	5/11/2018	59.3	LENGTH	5.8	13	1	1
27	9417	60	Azithromycin	1	24	2/4/2020	78.5	HEIGHT	9.1	13	1	1
27	9440	48	Azithromycin	1	20	4/13/2019	77.9	LENGTH	8.75	13.9	1	1
27	9479	48	Azithromycin	0	24	4/13/2019	81.6	LENGTH	6.35	15	1	1
27	9481	48	Azithromycin	0	15	4/13/2019	72.3	HEIGHT	8.25	13	1	1
27	9481	60	Azithromycin	0	25	2/4/2020	78.2	LENGTH	9.2	13	1	1
27	9511	48	Azithromycin	0	22	4/13/2019	82.8	LENGTH	10.95	15.5	1	1
27	9524	36	Azithromycin	1	6	5/11/2018	62.3	LENGTH	5.7	12	1	1
27	9524	48	Azithromycin	1	17	4/13/2019	76.4	HEIGHT	9.909090909	13.7	0	1
27	9524	60	Azithromycin	1	27	2/4/2020	86.4	HEIGHT	12.8	15	0	1
27	9569	48	Azithromycin	0	6	4/13/2019	69.3	LENGTH	9.1	15.6	1	1
27	9571	48	Azithromycin	0	4	4/13/2019	62.3	LENGTH	6.45	13	1	1
27	9571	60	Azithromycin	0	14	2/4/2020	71.8	LENGTH	8.3	14.9	1	1
28	6829	0	Azithromycin	0	58	3/13/2015	103.4	HEIGHT	14.4	15.7	1	0

28	6829	24	Azithromycin	0	71	3/19/2017	115.1	HEIGHT	17.7	15		0	0
28	6829	36	Azithromycin	0	86	5/13/2018	121	HEIGHT	19.25	15.6		0	0
28	6829	48	Azithromycin	0	97	4/11/2019	126.1	HEIGHT	21.2	15.5		0	0
28	6829	60	Azithromycin	0	107	2/7/2020	129.1	HEIGHT	23.6	16.7		0	0
28	6830	12	Azithromycin	1	5	5/12/2016	65.3	LENGTH	7.5	14.5		1	0
28	6833	0	Azithromycin	0	48	3/13/2015	94.3	HEIGHT	13.45	16.2		1	0
28	6833	36	Azithromycin	0	86	5/13/2018	114	HEIGHT	17.7	15.5		0	0
28	6833	48	Azithromycin	0	97	4/11/2019	117.5	HEIGHT	20.35	16.1		0	0
28	6834	0	Azithromycin	0	12	3/13/2015	68.5	LENGTH	7.5	13.7		1	0
28	6834	12	Azithromycin	0	21	5/12/2016	79.1	HEIGHT	9.45	13		1	0
28	6837	0	Azithromycin	0	40	3/13/2015	107.1	HEIGHT	17.9	17		1	0
28	6841	12	Azithromycin	0	32	5/12/2016	93.1	HEIGHT	12.15	15		1	0
28	6842	12	Azithromycin	0	38	5/12/2016	92	HEIGHT	12.75	15.1		1	0
28	6844	12	Azithromycin	0	24	5/12/2016	84.5	HEIGHT	12.2	14.5		1	0
28	6844	36	Azithromycin	0	47	5/13/2018	100	HEIGHT	15.7	15.5		1	0
28	6845	0	Azithromycin	0	12	3/13/2015	67.4	LENGTH	7.85	14		1	0
28	6845	24	Azithromycin	0	33	3/19/2017	85.1	HEIGHT	11	13.5		1	0
28	6845	48	Azithromycin	0	58	4/11/2019	100	HEIGHT	14.1	15		1	0
28	6845	60	Azithromycin	0	68	2/7/2020	106.4	HEIGHT	16.5	15.2		0	0
28	6846	24	Azithromycin	0	16	3/19/2017	80.1	HEIGHT	11.35	15		1	0
28	6846	36	Azithromycin	0	30	5/13/2018	91.7	HEIGHT	15.05	15.5		1	0
28	6846	48	Azithromycin	0	41	4/11/2019	98.5	HEIGHT	17.2	15.5		1	0
28	6848	0	Azithromycin	1	5	3/13/2015	56.4	LENGTH	4.7	12.4		1	0
28	6848	12	Azithromycin	1	12	5/12/2016	76.4	LENGTH	9.05	14.7		1	0
28	6848	24	Azithromycin	1	23	3/19/2017	87	HEIGHT	12	15		1	0
28	6848	36	Azithromycin	1	39	5/13/2018	95	HEIGHT	13.9	14.7		1	0
28	6848	48	Azithromycin	1	50	4/11/2019	105	HEIGHT	15.9	14.7		1	0
28	6849	12	Azithromycin	1	44	5/12/2016	102	HEIGHT	14.55	15.4		1	0
28	6852	12	Azithromycin	1	8	5/12/2016	69.3	LENGTH	8.02	14.5		1	0
28	6852	36	Azithromycin	1	28	5/13/2018	87.5	HEIGHT	11.75	15		1	0
28	6854	0	Azithromycin	0	31	3/13/2015	86.7	HEIGHT	11.15	15		1	0
28	6854	12	Azithromycin	0	48	5/12/2016	95.4	HEIGHT	12.8	13.5		1	0
28	6854	36	Azithromycin	0	118	5/13/2018	107.5	HEIGHT	14.35	13		0	0
28	6854	48	Azithromycin	0	129	4/11/2019	112.4	HEIGHT	16.25	14		0	0
28	6858	0	Azithromycin	0	36	3/13/2015	84.8	HEIGHT	10.5	15		1	0
28	6858	12	Azithromycin	0	42	5/12/2016	93.8	HEIGHT	12.05	14		1	0
28	6858	36	Azithromycin	0	68	5/13/2018	106.5	HEIGHT	14.7	15		0	0
28	6859	0	Azithromycin	0	42	3/13/2015	101.1	HEIGHT	14.45	16		1	0
28	6859	36	Azithromycin	0	92	5/13/2018	119.5	HEIGHT	19.85	16.5		0	0
28	6859	48	Azithromycin	0	103	4/11/2019	125.5	HEIGHT	22.25	17		0	0

28	6860	0	Azithromycin	1	13	3/13/2015	71.1	LENGTH	8.85	15		1	0
28	6861	12	Azithromycin	0	42	5/12/2016	95.4	HEIGHT	12.5	14		1	0
28	6861	24	Azithromycin	0	53	3/19/2017	102.1	HEIGHT	13.6	14		1	0
28	6861	36	Azithromycin	0	68	5/13/2018	109	HEIGHT	15.2	14		0	0
28	6861	48	Azithromycin	0	82	4/11/2019	113.9	HEIGHT	16.9	14.1		0	0
28	6861	60	Azithromycin	0	91	2/7/2020	119.5	HEIGHT	17.8	14.5		0	0
28	6862	0	Azithromycin	0	12	3/13/2015	67.9	LENGTH	8.2	14.3		1	0
28	6862	12	Azithromycin	0	20	5/12/2016	80	HEIGHT	11.65	15.5		1	0
28	6862	24	Azithromycin	0	34	3/19/2017	89.1	HEIGHT	13.45	15		1	0
28	6862	36	Azithromycin	0	48	5/13/2018	99	HEIGHT	16.3	16		1	0
28	6862	48	Azithromycin	0	59	4/11/2019	107.1	HEIGHT	17.25	15		1	0
28	6864	0	Azithromycin	1	10	6/4/2015	73.4	LENGTH	8.2	14		1	0
28	6864	12	Azithromycin	1	30	5/12/2016	84	HEIGHT	9.9	14		1	0
28	6864	36	Azithromycin	1	56	5/13/2018	100.4	HEIGHT	13.5	14		1	0
28	6864	48	Azithromycin	1	74	4/11/2019	107.8	HEIGHT	15.8	14.5		0	0
28	6864	60	Azithromycin	1	84	2/7/2020	111.9	HEIGHT	17.35	15		0	0
28	6867	24	Azithromycin	0	4	3/19/2017	67.6	HEIGHT	9.05	15.5	30	1	0
28	6871	12	Azithromycin	0	7	5/12/2016	69.3	LENGTH	7	13.5		1	0
28	6871	24	Azithromycin	0	18	3/19/2017	79.1	HEIGHT	8.15	11.5		1	0
28	6871	36	Azithromycin	0	33	5/13/2018	88	HEIGHT	11.3	13.3		1	0
28	6871	48	Azithromycin	0	44	4/11/2019	96.1	HEIGHT	12.7	13		1	0
28	6871	60	Azithromycin	0	54	2/7/2020	100.3	HEIGHT	13.3	12.7		1	0
28	6872	0	Azithromycin	1	8	3/13/2015	61.3	LENGTH	6.9	15.5		1	0
28	6872	12	Azithromycin	1	12	5/12/2016	76.3	LENGTH	9.1	15.5		1	0
28	6872	24	Azithromycin	1	23	3/19/2017	84.5	HEIGHT	11.55	15.5		1	0
28	6872	36	Azithromycin	1	41	5/13/2018	96.5	HEIGHT	13.7	15.5		1	0
28	6872	48	Azithromycin	1	52	4/11/2019	106	HEIGHT	16.15	15.5		1	0
28	6872	60	Azithromycin	1	62	2/7/2020	111.8	HEIGHT	17.2	16.2		0	0
28	6874	0	Azithromycin	0	36	3/13/2015	93.2	HEIGHT	13.85	16.5		1	0
28	6874	24	Azithromycin	0	64	3/19/2017	106.1	HEIGHT	15.85	15.5		0	0
28	6874	36	Azithromycin	0	79	5/13/2018	112	HEIGHT	17.95	15.9		0	0
28	6874	48	Azithromycin	0	90	4/11/2019	116.5	HEIGHT	19.45	15.8		0	0
28	6874	60	Azithromycin	0	99	2/7/2020	121.9	HEIGHT	21.8	16.5		0	0
28	6875	0	Azithromycin	1	48	3/13/2015	98.2	HEIGHT	13.5	15.6		1	0
28	6876	0	Azithromycin	1	42	3/13/2015	92	HEIGHT	13.3	16.3		1	0
28	6876	12	Azithromycin	1	54	5/12/2016	101.6	HEIGHT	14.65	15		1	0
28	6876	24	Azithromycin	1	65	3/19/2017	109	HEIGHT	16.3	15		0	0
28	6876	36	Azithromycin	1	80	5/13/2018	117	HEIGHT	18.6	14.7		0	0
28	6876	48	Azithromycin	1	91	4/11/2019	120	HEIGHT	20.15	15		0	0
28	6881	0	Azithromycin	0	36	3/13/2015	97.3	HEIGHT	15.05	17		1	0

28	6881	12	Azithromycin	0	54	5/12/2016	104.4	HEIGHT	16.6	16.2	1	0
28	6881	24	Azithromycin	0	64	3/19/2017	109.6	HEIGHT	16.9	16	0	0
28	6881	36	Azithromycin	0	79	5/13/2018	115	HEIGHT	19.1	16.5	0	0
28	6881	48	Azithromycin	0	90	4/11/2019	121.4	HEIGHT	21.35	16.5	0	0
28	6881	60	Azithromycin	0	99	2/7/2020	126.4	HEIGHT	23.5	17.7	0	0
28	6882	12	Azithromycin	0	5	5/12/2016	70.5	HEIGHT	7.4	13.3	1	0
28	6882	24	Azithromycin	0	16	3/19/2017	79	HEIGHT	10.8	15	1	0
28	6882	36	Azithromycin	0	30	5/13/2018	89.5	HEIGHT	12.8	14	1	0
28	6882	48	Azithromycin	0	41	4/11/2019	99.9	HEIGHT	15.3	14.5	1	0
28	6882	60	Azithromycin	0	51	2/7/2020	105.8	HEIGHT	17.2	17	1	0
28	6883	12	Azithromycin	0	32	5/12/2016	105.7	HEIGHT	16.55	15.1	1	0
28	6884	12	Azithromycin	1	8	5/12/2016	68.7	LENGTH	7.45	14	1	0
28	6887	0	Azithromycin	1	54	3/13/2015	99	HEIGHT	14.3	16.4	1	0
28	6887	12	Azithromycin	1	66	5/12/2016	105.8	HEIGHT	14.6	15.7	0	0
28	6887	24	Azithromycin	1	78	3/19/2017	111	HEIGHT	16.4	15.6	0	0
28	6887	36	Azithromycin	1	92	5/13/2018	116.5	HEIGHT	17.65	15.5	0	0
28	6887	48	Azithromycin	1	103	4/11/2019	120.9	HEIGHT	19.55	15.5	0	0
28	6887	60	Azithromycin	1	113	2/7/2020	123.7	HEIGHT	21	16.2	0	0
28	6890	0	Azithromycin	1	54	3/13/2015	103.1	HEIGHT	16.35	17.3	1	0
28	6890	12	Azithromycin	1	36	5/12/2016	110.6	HEIGHT	18.15	16.5	1	0
28	6890	24	Azithromycin	1	56	3/19/2017	117.5	HEIGHT	20.3	17	1	0
28	6890	36	Azithromycin	1	92	5/13/2018	122.5	HEIGHT	21.45	16.5	0	0
28	6891	0	Azithromycin	0	18	3/13/2015	74.4	LENGTH	8.25	14	1	0
28	6891	12	Azithromycin	0	27	5/12/2016	86.5	HEIGHT	10.9	14.4	1	0
28	6891	24	Azithromycin	0	38	3/19/2017	94.5	HEIGHT	12.4	14	1	0
28	6891	36	Azithromycin	0	53	5/13/2018	101.5	HEIGHT	13.6	13.5	1	0
28	6891	48	Azithromycin	0	64	4/11/2019	108	HEIGHT	15.35	13.5	0	0
28	6891	60	Azithromycin	0	74	2/7/2020	111.6	HEIGHT	17.2	14.3	0	0
28	6892	0	Azithromycin	0	48	3/13/2015	82.5	LENGTH	10.25	14.5	1	0
28	6892	36	Azithromycin	0	98	5/13/2018	119	HEIGHT	18.55	14.8	0	0
28	6892	48	Azithromycin	0	112	4/11/2019	123.1	HEIGHT	19.75	14.9	0	0
28	6895	12	Azithromycin	1	24	5/12/2016	85	HEIGHT	11.35	16	1	0
28	6895	24	Azithromycin	1	42	3/19/2017	90	HEIGHT	12.5	15.3	1	0
28	6896	0	Azithromycin	0	24	3/13/2015	82.1	LENGTH	11.75	16	1	0
28	6897	0	Azithromycin	0	7	3/13/2015	69.1	LENGTH	6.8	12.6	1	0
28	6897	12	Azithromycin	0	24	5/12/2016	81.6	HEIGHT	9.85	13.7	1	0
28	6897	24	Azithromycin	0	33	3/19/2017	89.5	HEIGHT	11.8	14	1	0
28	6897	36	Azithromycin	0	49	5/13/2018	97.5	HEIGHT	12.7	13.9	1	0
28	6897	48	Azithromycin	0	59	4/11/2019	102.7	HEIGHT	13.6	13.2	1	0
28	6898	0	Azithromycin	0	24	3/13/2015	84.6	HEIGHT	11.05	15.5	1	0

28	6898	12	Azithromycin	0	42	5/12/2016	95.4	HEIGHT	12.7	16	1	0
28	6898	24	Azithromycin	0	53	3/19/2017	101.1	HEIGHT	13.95	14.9	1	0
28	6900	0	Azithromycin	1	48	3/13/2015	103.1	HEIGHT	16.3	17	1	0
28	6900	24	Azithromycin	1	71	3/19/2017	116.1	HEIGHT	20.35	16.5	0	0
28	6900	36	Azithromycin	1	86	5/13/2018	122.5	HEIGHT	22.55	16.5	0	0
28	6900	48	Azithromycin	1	97	4/11/2019	127.4	HEIGHT	25.5	17.3	0	0
28	6902	0	Azithromycin	1	18	3/13/2015	77.3	LENGTH	11.3	16	1	0
28	6902	24	Azithromycin	1	29	3/19/2017	97.1	HEIGHT	15.35	16.5	1	0
28	6904	0	Azithromycin	1	36	3/13/2015	91.2	HEIGHT	11.75	14	1	0
28	6904	12	Azithromycin	1	36	5/12/2016	100.2	HEIGHT	13.35	13.8	1	0
28	6904	24	Azithromycin	1	47	3/19/2017	107	HEIGHT	15	13	1	0
28	6904	36	Azithromycin	1	71	5/13/2018	114.9	HEIGHT	15.65	13	0	0
28	6904	48	Azithromycin	1	82	4/11/2019	120.6	HEIGHT	18.8	14	0	0
28	6904	60	Azithromycin	1	92	2/7/2020	127.2	HEIGHT	23.8	14.9	0	0
28	6909	12	Azithromycin	1	4	5/12/2016	64.8	LENGTH	7.1	13.5	1	0
28	6909	24	Azithromycin	1	16	3/19/2017	75.1	HEIGHT	9.3	15	1	0
28	6909	36	Azithromycin	1	30	5/13/2018	85	HEIGHT	11.95	14	1	0
28	6909	48	Azithromycin	1	41	4/11/2019	91.5	HEIGHT	12.85	14.5	1	0
28	6910	0	Azithromycin	0	18	6/4/2015	78.1	LENGTH	9.95	15.6	1	0
28	6911	0	Azithromycin	1	42	3/13/2015	92.1	HEIGHT	13.95	16.5	1	0
28	6911	12	Azithromycin	1	42	5/12/2016	102.6	HEIGHT	15.99	16	1	0
28	6911	24	Azithromycin	1	53	3/19/2017	109.5	HEIGHT	16.85	15.5	1	0
28	6912	0	Azithromycin	0	42	3/13/2015	96	HEIGHT	16.15	18	1	0
28	6912	12	Azithromycin	0	54	5/12/2016	104.6	HEIGHT	17.7	17	1	0
28	6912	24	Azithromycin	0	65	3/19/2017	110	HEIGHT	19.05	16.5	0	0
28	6912	36	Azithromycin	0	80	5/13/2018	116.1	HEIGHT	20.85	16.5	0	0
28	6912	48	Azithromycin	0	91	4/11/2019	120.5	HEIGHT	22.5	17	0	0
28	6912	60	Azithromycin	0	100	2/7/2020	125.6	HEIGHT	24.9	18	0	0
28	6914	0	Azithromycin	1	18	3/13/2015	75.6	LENGTH	9.8	15.4	1	0
28	6914	12	Azithromycin	1	36	5/12/2016	84.4	HEIGHT	12	15	1	0
28	6917	12	Azithromycin	1	54	5/12/2016	118.7	HEIGHT	19.1	15.5	1	0
28	6917	60	Azithromycin	1	125	2/7/2020	140.9	HEIGHT	34.4	19.3	0	0
28	6918	0	Azithromycin	0	48	3/13/2015	85.2	HEIGHT	13.15	16.7	1	0
28	6921	0	Azithromycin	1	42	3/13/2015	108.2	HEIGHT	18.15	17	1	0
28	6922	0	Azithromycin	0	18	3/13/2015	76.1	LENGTH	10	14.8	1	0
28	6922	12	Azithromycin	0	32	5/12/2016	87.5	HEIGHT	11.8	15	1	0
28	6923	0	Azithromycin	1	54	3/13/2015	109	HEIGHT	16.95	16	1	0
28	6923	24	Azithromycin	1	77	3/19/2017	122.1	HEIGHT	20.05	15.5	0	0
28	6924	0	Azithromycin	0	9	3/13/2015	76	LENGTH	9.4	14.8	1	0
28	6925	12	Azithromycin	1	54	5/12/2016	116.1	HEIGHT	17.55	15.5	1	0

28	6925	60	Azithromycin	1	124	2/7/2020	135.9	HEIGHT	28	18.5		0	0
28	6926	12	Azithromycin	1	54	5/12/2016	131	HEIGHT	30.75	19.5		1	0
28	6927	0	Azithromycin	0	36	3/13/2015	91.8	HEIGHT	12.3	15		1	0
28	6927	24	Azithromycin	0	59	3/19/2017	107	HEIGHT	15.95	15		1	0
28	6929	0	Azithromycin	1	48	6/4/2015	86.9	LENGTH	11.6	15.2		1	0
28	6929	24	Azithromycin	1	71	3/19/2017	98.1	HEIGHT	13.15	14		0	0
28	6929	36	Azithromycin	1	86	5/13/2018	112.8	LENGTH	14.2	13.7		0	0
28	6929	48	Azithromycin	1	86	4/11/2019	108.1	HEIGHT	15.85	14.2		0	0
28	6929	60	Azithromycin	1	96	2/7/2020	112.3	HEIGHT	17.8	14.8		0	0
28	6930	24	Azithromycin	0	12	3/19/2017	71.1	HEIGHT	8.75	14		1	0
28	6930	36	Azithromycin	0	26	5/13/2018	86.5	HEIGHT	11.6	14.5		1	0
28	6930	48	Azithromycin	0	37	4/11/2019	94.4	HEIGHT	13.6	15		1	0
28	6932	12	Azithromycin	1	30	5/12/2016	89.4	HEIGHT	13.8	16.3		1	0
28	6932	36	Azithromycin	1	56	5/13/2018	105.5	HEIGHT	17.8	16		1	0
28	6934	24	Azithromycin	0	4	3/19/2017	62.3	LENGTH	6.35	14		1	0
28	6934	48	Azithromycin	0	29	4/11/2019	82.5	HEIGHT	10.7	14		1	0
28	6939	0	Azithromycin	1	7	3/13/2015	57.4	LENGTH	5.55	12.7		1	0
28	6939	12	Azithromycin	1	18	5/12/2016	73.3	LENGTH	8.3	14		1	0
28	6939	24	Azithromycin	1	29	3/19/2017	79.1	HEIGHT	11.05	14.6		1	0
28	6939	36	Azithromycin	1	40	5/13/2018	90.5	HEIGHT	13.1	15.5		1	0
28	6939	48	Azithromycin	1	51	4/11/2019	98.2	HEIGHT	14.7	14.8		1	0
28	6939	60	Azithromycin	1	61	2/7/2020	104.6	HEIGHT	16.3	16.3		0	0
28	6941	24	Azithromycin	1	2	3/19/2017	58.8	LENGTH	5.25	12.5		1	0
28	6941	36	Azithromycin	1	17	5/13/2018	75.5	HEIGHT	8.95	14.5		1	0
28	6941	48	Azithromycin	1	28	4/11/2019	83.5	HEIGHT	10.05	14		1	0
28	8110	36	Azithromycin	1	22	5/13/2018	75.5	HEIGHT	9	13.7		1	0
28	8141	36	Azithromycin	1	28	5/13/2018	83.9	HEIGHT	10.95	14.4		1	0
28	8141	48	Azithromycin	1	43	4/11/2019	92.1	HEIGHT	12.95	14.8		1	0
28	8141	60	Azithromycin	1	53	2/7/2020	97.3	HEIGHT	14.3	14.7		1	0
28	8319	36	Azithromycin	0	18	5/13/2018	75	HEIGHT	10.4	15		1	0
28	8319	48	Azithromycin	0	29	4/11/2019	84.1	HEIGHT	12.25	16		1	0
28	8319	60	Azithromycin	0	39	2/7/2020	90.2	HEIGHT	13.6	15.9		1	0
28	8351	48	Azithromycin	0	4	4/11/2019	64.6	LENGTH	6.65	11.7		1	0
28	8351	60	Azithromycin	0	13	2/7/2020	74.9	HEIGHT	9.65	13.7		1	0
28	8462	36	Azithromycin	1	11	5/13/2018	75	HEIGHT	8.45	13		1	0
28	8462	48	Azithromycin	1	22	4/11/2019	83.2	HEIGHT	10.5	13.5		1	0
28	8462	60	Azithromycin	1	32	2/7/2020	90.2	HEIGHT	12.7	15.3		1	0
28	8645	36	Azithromycin	1	20	5/13/2018	74.4	HEIGHT	8.45	13.5	42	1	0
28	8704	48	Azithromycin	0	3	4/11/2019	59.7	LENGTH	6.9	15.5		1	0
28	8704	60	Azithromycin	0	12	2/7/2020	71.8	LENGTH	9.5	14.2		1	0

28	8733	36	Azithromycin	1	10	5/13/2018	71.3	LENGTH	7.05	12		1	0
28	8733	48	Azithromycin	1	21	4/11/2019	77.4	HEIGHT	8.3	13		1	0
28	8894	48	Azithromycin	0	4	4/11/2019	63.5	LENGTH	6.05	12		1	0
28	8894	60	Azithromycin	0	13	2/7/2020	74.8	HEIGHT	8.2	12.9		1	0
28	8900	60	Azithromycin	1	10	2/7/2020	69.5	HEIGHT	8.8	15.6		1	0
28	8932	48	Azithromycin	0	5	4/11/2019	63.3	LENGTH	6.65	13.5		1	0
28	8932	60	Azithromycin	0	13	2/7/2020	74.7	LENGTH	10.5	15.4		1	0
28	9066	36	Azithromycin	1	15	5/13/2018	70.3	LENGTH	7.25	13		1	0
28	9115	48	Azithromycin	0	103	4/11/2019	127.5	HEIGHT	23.2	17.5		0	0
28	9228	36	Azithromycin	0	5	5/13/2018	65.7	LENGTH	8.35	16		1	0
28	9295	48	Azithromycin	1	25	4/11/2019	72.4	HEIGHT	7.4	11.5		1	0
28	9406	36	Azithromycin	1	20	5/13/2018	76.5	HEIGHT	9.5	14		1	0
28	9508	48	Azithromycin	0	1	4/11/2019	56.4	LENGTH	5.4	12		1	0
28	9508	60	Azithromycin	0	11	2/7/2020	72.7	LENGTH	8.85	14		1	0
29	6953	12	Azithromycin	0	10	6/24/2016	74.2	HEIGHT	8.15	13.5		1	0
29	6956	24	Azithromycin	0	24	4/28/2017	84.7	HEIGHT	11.3	14		1	0
29	6956	60	Azithromycin	0	58	2/14/2020	105	HEIGHT	16.75	15.1		1	0
29	6958	12	Azithromycin	0	11	6/25/2016	74	HEIGHT	8.05	14.5		0	0
29	6958	24	Azithromycin	0	21	4/29/2017	81.4	HEIGHT	9.409090909	13.5		1	0
29	6958	36	Azithromycin	0	34	5/19/2018	88.8	HEIGHT	11	14		0	0
29	6962	24	Azithromycin	0	1	4/28/2017	57.7	LENGTH	5.909090909	13		1	0
29	6962	36	Azithromycin	0	14	5/19/2018	77.9	LENGTH	8.95	13.5		0	0
29	6962	48	Azithromycin	0	25	4/19/2019	85.7	HEIGHT	12.75	16.5		0	0
29	6965	12	Azithromycin	0	114	6/25/2016	126.2	HEIGHT	18.9	14.5		0	0
29	6966	12	Azithromycin	1	48	6/25/2016	111.9	HEIGHT	16.75	15		1	0
29	6967	12	Azithromycin	1	15	6/24/2016	75.5	LENGTH	8.2	14		1	0
29	6969	0	Azithromycin	0	24	4/1/2015	75.4	LENGTH	8.1	13		0	0
29	6969	24	Azithromycin	0	55	4/28/2017	94.5	HEIGHT	12.45454545	13		1	0
29	6969	36	Azithromycin	0	68	5/19/2018	98.6	HEIGHT	14.85	13.5		0	0
29	6970	0	Azithromycin	0	36	4/1/2015	86.8	HEIGHT	11.55	14		1	0
29	6970	12	Azithromycin	0	30	6/24/2016	97.6	HEIGHT	13.1	14		1	0
29	6970	24	Azithromycin	0	43	4/28/2017	102	HEIGHT	15	14		0	0
29	6970	36	Azithromycin	0	56	5/19/2018	106.4	HEIGHT	16.2	14		1	0
29	6970	48	Azithromycin	0	67	4/19/2019	111.4	HEIGHT	17.25	14		0	0
29	6970	60	Azithromycin	0	76	2/14/2020	114.5	LENGTH	19.1	14.7		0	0
29	6973	24	Azithromycin	1	28	4/29/2017	84.5	HEIGHT	9.954545455	13.5	30	1	0
29	6974	12	Azithromycin	0	48	6/25/2016	99.1	HEIGHT	12.4	13		1	0
29	6976	0	Azithromycin	1	9	4/1/2015	68.2	LENGTH	7.35	13.5	30	0	0
29	6976	12	Azithromycin	1	30	6/25/2016	82	HEIGHT	9.7	14.5	30	0	0
29	6976	24	Azithromycin	1	43	4/28/2017	85.2	HEIGHT	11.68181818	14.5	30	1	0

29	6978	0	Azithromycin	0	36	5/9/2015	96.1	LENGTH	13.2	15.5		0	0
29	6978	12	Azithromycin	0	54	6/24/2016	105.1	HEIGHT	14	15.5		1	0
29	6978	24	Azithromycin	0	64	4/28/2017	107.5	LENGTH	15.45	15.5		0	0
29	6983	0	Azithromycin	0	36	4/1/2015	89.4	HEIGHT	11.9	15	48	1	0
29	6983	12	Azithromycin	0	60	6/25/2016	97.5	HEIGHT	13.4	14.5	48	0	0
29	6983	24	Azithromycin	0	73	4/28/2017	101.9	HEIGHT	14.40909091	14.5	48	0	0
29	6986	0	Azithromycin	1	9	4/1/2015	74.7	LENGTH	8.45	13.5		1	0
29	6986	12	Azithromycin	1	30	6/24/2016	81.6	HEIGHT	9.4	14		1	0
29	6986	24	Azithromycin	1	43	4/28/2017	87.7	HEIGHT	12.18181818	14.5		0	0
29	6986	36	Azithromycin	1	56	5/19/2018	99.7	HEIGHT	14.95	15		1	0
29	6986	48	Azithromycin	1	67	4/19/2019	106.1	HEIGHT	15.75	14.5		0	0
29	6987	0	Azithromycin	0	24	5/9/2015	78.5	LENGTH	9.75	14	18	0	0
29	6989	24	Azithromycin	1	66	4/29/2017	113.3	HEIGHT	18.22727273	14		1	0
29	6994	0	Azithromycin	0	36	4/1/2015	93.8	HEIGHT	12.6	14.5		1	0
29	6994	12	Azithromycin	0	51	6/24/2016	100.6	HEIGHT	14	13.5		1	0
29	6994	24	Azithromycin	0	64	4/28/2017	105.6	HEIGHT	15.72727273	14.5		0	0
29	6994	36	Azithromycin	0	77	5/19/2018	109.6	HEIGHT	16.1	14		0	0
29	6994	48	Azithromycin	0	88	4/19/2019	112.1	HEIGHT	17.5	14.5		0	0
29	6994	60	Azithromycin	0	98	2/14/2020	117.4	HEIGHT	19.85	14.9		0	0
29	6995	24	Azithromycin	0	31	4/28/2017	86.9	HEIGHT	10.68181818	14		1	0
29	6995	36	Azithromycin	0	44	5/19/2018	91.7	HEIGHT	12.35	14.5		1	0
29	6995	48	Azithromycin	0	55	4/22/2019	79.3	LENGTH	9.25	12.5		1	0
29	6996	0	Azithromycin	1	4	5/9/2015	69.2	LENGTH	6.7	12.5	6	1	0
29	6997	0	Azithromycin	1	48	5/9/2015	83.1	HEIGHT	10.55	13.5		1	0
29	6997	12	Azithromycin	1	42	6/25/2016	94.3	HEIGHT	12.2	13.5		0	0
29	6997	24	Azithromycin	1	55	4/29/2017	98.7	HEIGHT	14.04545455	14		0	0
29	6997	36	Azithromycin	1	68	5/19/2018	103.4	HEIGHT	15.6	14		0	0
29	6997	48	Azithromycin	1	79	4/19/2019	108.3	HEIGHT	16.15	13.5		0	0
29	6997	60	Azithromycin	1	88	2/14/2020	114.7	HEIGHT	17.7	14.2		0	0
29	6998	0	Azithromycin	1	24	4/1/2015	87.5	HEIGHT	11.15	14		1	0
29	6998	12	Azithromycin	1	39	6/25/2016	97.5	HEIGHT	12.7	14.5		0	0
29	6998	24	Azithromycin	1	52	4/28/2017	103.1	HEIGHT	14.3	14.5		0	0
29	6998	36	Azithromycin	1	65	5/19/2018	108.5	HEIGHT	16.1	14.5		0	0
29	6998	48	Azithromycin	1	76	4/19/2019	113.1	HEIGHT	17.15	14.5		0	0
29	6999	12	Azithromycin	1	45	6/24/2016	99.2	HEIGHT	13.7	15.5		1	0
29	7003	0	Azithromycin	0	24	4/1/2015	86.8	LENGTH	10.3	13		1	0
29	7003	12	Azithromycin	0	39	6/25/2016	96.6	LENGTH	12.45	13.5		0	0
29	7003	24	Azithromycin	0	52	4/28/2017	102.6	HEIGHT	14	14		0	0
29	7003	36	Azithromycin	0	65	5/19/2018	110.2	HEIGHT	16.25	14.5		0	0
29	7003	48	Azithromycin	0	76	4/19/2019	130.6	HEIGHT	22.35	15		0	0

29	7004	12	Azithromycin	1	36	6/24/2016	78.2	HEIGHT	7.95	11.5	30	1	0
29	7005	0	Azithromycin	1	36	5/9/2015	98.4	HEIGHT	15	15.5	18	1	0
29	7006	12	Azithromycin	0	9	6/25/2016	69.1	LENGTH	6.5	12.5		0	0
29	7006	36	Azithromycin	0	28	5/19/2018	84.9	HEIGHT	10.7	13.5		0	0
29	7006	60	Azithromycin	0	48	2/14/2020	98	HEIGHT	13.1	13.3		0	0
29	7007	0	Azithromycin	0	12	4/1/2015	73.5	LENGTH	7.2	12.5	24	0	0
29	7007	12	Azithromycin	0	27	6/25/2016	86.1	HEIGHT	10.15	14.5	24	1	0
29	7008	0	Azithromycin	1	36	4/1/2015	89.5	HEIGHT	11.65	14.5		0	0
29	7008	12	Azithromycin	1	51	6/25/2016	99.9	HEIGHT	13.25	14.5		0	0
29	7008	24	Azithromycin	1	64	4/28/2017	107.8	HEIGHT	15.68181818	14		0	0
29	7008	36	Azithromycin	1	77	5/19/2018	112.1	HEIGHT	16.1	14		0	0
29	7008	48	Azithromycin	1	88	4/19/2019	116.5	HEIGHT	18.4	14.5		0	0
29	7012	0	Azithromycin	1	12	4/1/2015	74.1	LENGTH	8.85	14	24	1	0
29	7014	0	Azithromycin	0	24	4/1/2015	88.3	HEIGHT	10.9	13.5		0	0
29	7014	12	Azithromycin	0	39	6/25/2016	98.8	HEIGHT	13.45	14		0	0
29	7014	24	Azithromycin	0	52	4/28/2017	106.7	HEIGHT	14.59090909	13.5		0	0
29	7014	36	Azithromycin	0	65	5/19/2018	113.9	HEIGHT	16.3	13.5		0	0
29	7015	0	Azithromycin	0	48	4/1/2015	111.8	HEIGHT	17.15	14.5		1	0
29	7015	24	Azithromycin	0	76	4/28/2017	123	HEIGHT	21.27272727	15		0	0
29	7015	36	Azithromycin	0	89	5/19/2018	126.7	HEIGHT	23.35	16		0	0
29	7015	48	Azithromycin	0	100	4/19/2019	129.3	HEIGHT	25.75	16.5		0	0
29	7016	12	Azithromycin	0	21	6/27/2016	78	HEIGHT	9.2	13.5		1	0
29	7016	24	Azithromycin	0	31	4/29/2017	86.3	HEIGHT	10.77272727	13.5		1	0
29	7016	48	Azithromycin	0	55	4/19/2019	97.3	HEIGHT	13.3	13		1	0
29	7020	0	Azithromycin	0	6	4/1/2015	70.6	LENGTH	6.5	11.5		1	0
29	7020	12	Azithromycin	0	21	6/28/2016	81.2	HEIGHT	9.9	12.5		0	0
29	7020	24	Azithromycin	0	34	4/28/2017	88.6	HEIGHT	12.25	14.5		1	0
29	7020	36	Azithromycin	0	47	5/19/2018	95.8	HEIGHT	13.45	14		1	0
29	7020	48	Azithromycin	0	58	4/19/2019	100.6	HEIGHT	14.55	13.5		1	0
29	7021	0	Azithromycin	0	48	6/12/2015	96.2	HEIGHT	13.8	15.5		1	0
29	7021	36	Azithromycin	0	89	5/19/2018	111.7	HEIGHT	18.6	14.5		0	0
29	7021	48	Azithromycin	0	100	4/19/2019	114.5	HEIGHT	20.65	15		0	0
29	7022	12	Azithromycin	1	33	6/25/2016	86.5	HEIGHT	10	13.5		1	0
29	7022	24	Azithromycin	1	43	4/28/2017	90.3	HEIGHT	11.81818182	14		1	0
29	7022	36	Azithromycin	1	56	5/19/2018	96.5	HEIGHT	12.7	14		1	0
29	7024	24	Azithromycin	0	24	4/28/2017	82.5	HEIGHT	10.22727273	13		1	0
29	7024	60	Azithromycin	0	58	2/14/2020	103.9	HEIGHT	16.45	14		1	0
29	7025	0	Azithromycin	0	48	4/1/2015	94.7	HEIGHT	12.15	14		0	0
29	7025	12	Azithromycin	0	42	6/24/2016	102.5	HEIGHT	13.15	13.5		1	0
29	7025	24	Azithromycin	0	55	4/28/2017	107.4	HEIGHT	15	14		1	0

29	7025	36	Azithromycin	0	68	5/19/2018	114.7	HEIGHT	16.35	13.5	0	0
29	7025	48	Azithromycin	0	79	4/19/2019	118.6	HEIGHT	18	14	0	0
29	7027	0	Azithromycin	1	3	4/1/2015	66.2	LENGTH	7.35	13.5	0	0
29	7027	12	Azithromycin	1	15	6/25/2016	77.6	HEIGHT	8.55	13.5	0	0
29	7027	24	Azithromycin	1	28	4/28/2017	87	HEIGHT	10.72727273	14	0	0
29	7027	36	Azithromycin	1	41	5/19/2018	93.7	HEIGHT	13.05	14	1	0
29	7027	48	Azithromycin	1	52	4/19/2019	97.3	HEIGHT	14.05	14.5	1	0
29	7028	0	Azithromycin	1	48	5/9/2015	99.4	HEIGHT	12.3	12.5	1	0
29	7028	12	Azithromycin	1	63	6/25/2016	105.1	HEIGHT	13.55	14	0	0
29	7028	24	Azithromycin	1	76	4/29/2017	110.7	HEIGHT	15.09090909	13	0	0
29	7028	36	Azithromycin	1	89	5/19/2018	116.5	HEIGHT	16.8	14	0	0
29	7029	0	Azithromycin	1	48	6/12/2015	93.3	HEIGHT	11.85	13	0	0
29	7029	12	Azithromycin	1	57	6/25/2016	100.2	HEIGHT	12.6	13.5	1	0
29	7029	24	Azithromycin	1	67	4/29/2017	104.8	HEIGHT	13.90909091	12.5	0	0
29	7029	36	Azithromycin	1	80	5/19/2018	109	HEIGHT	14.85	13	0	0
29	7029	48	Azithromycin	1	91	4/19/2019	113.5	HEIGHT	17.2	14	0	0
29	7031	0	Azithromycin	1	24	6/12/2015	89.1	HEIGHT	12.05	15.5	0	0
29	7031	12	Azithromycin	1	39	6/25/2016	98.4	HEIGHT	14.2	15	0	0
29	7031	24	Azithromycin	1	52	4/28/2017	103.1	HEIGHT	14.86363636	15	1	0
29	7031	36	Azithromycin	1	65	5/19/2018	109.4	HEIGHT	17.2	15	0	0
29	7031	48	Azithromycin	1	76	4/19/2019	113.3	HEIGHT	18.05	15	0	0
29	7034	0	Azithromycin	0	12	5/9/2015	76.3	LENGTH	9.1	15.5	1	0
29	7037	24	Azithromycin	1	21	4/29/2017	81	HEIGHT	8.863636364	12.5	1	0
29	7038	12	Azithromycin	1	51	6/25/2016	95	HEIGHT	12.9	15	1	0
29	7039	0	Azithromycin	1	48	4/1/2015	97.2	HEIGHT	12.1	13	1	0
29	7039	12	Azithromycin	1	63	6/25/2016	107.5	HEIGHT	13.8	13.5	0	0
29	7039	24	Azithromycin	1	76	4/28/2017	112.3	HEIGHT	15.59090909	13.5	0	0
29	7039	36	Azithromycin	1	89	5/19/2018	117.9	HEIGHT	18.6	14.5	0	0
29	7039	48	Azithromycin	1	100	4/19/2019	122.8	HEIGHT	19.85	14.5	0	0
29	7043	0	Azithromycin	0	1	6/12/2015	68.7	LENGTH	7.1	12.5	1	0
29	7043	12	Azithromycin	0	18	6/28/2016	80.2	HEIGHT	8.85	13.5	0	0
29	7043	24	Azithromycin	0	28	4/28/2017	88.3	HEIGHT	10.8	14	1	0
29	7043	36	Azithromycin	0	41	5/19/2018	97.6	HEIGHT	13.35	14	0	0
29	7046	0	Azithromycin	0	36	5/9/2015	81	LENGTH	10	13	1	0
29	7046	12	Azithromycin	0	51	6/25/2016	88.7	HEIGHT	11.4	13	0	0
29	7046	24	Azithromycin	0	57	4/28/2017	93.4	HEIGHT	12.4	13.5	0	0
29	7046	48	Azithromycin	0	81	4/19/2019	103.8	HEIGHT	14.45	12.5	0	0
29	7046	60	Azithromycin	0	91	2/14/2020	107.8	HEIGHT	14.85	12.6	0	0
29	7047	0	Azithromycin	1	24	4/1/2015	84.2	HEIGHT	12.05	14.5	1	0
29	7047	12	Azithromycin	1	42	6/25/2016	93.7	HEIGHT	14.15	16	0	0

29	7047	36	Azithromycin	1	65	5/19/2018	111.7	HEIGHT	19.05	16	0	0
29	7047	48	Azithromycin	1	76	4/19/2019	117.4	HEIGHT	21.3	16	0	0
29	7047	60	Azithromycin	1	85	2/14/2020	122.2	HEIGHT	22.6	16.4	0	0
29	7048	12	Azithromycin	0	57	6/24/2016	104	HEIGHT	12.15	12.5	1	0
29	7053	0	Azithromycin	0	12	5/9/2015	70.6	HEIGHT	6.45	11.5	1	0
29	7053	12	Azithromycin	0	27	6/24/2016	80.8	HEIGHT	8.95	13	1	0
29	7053	24	Azithromycin	0	40	4/28/2017	86	HEIGHT	10	13	1	0
29	7053	36	Azithromycin	0	53	5/19/2018	94.3	HEIGHT	11.25	12.5	1	0
29	7053	48	Azithromycin	0	64	4/19/2019	99.8	HEIGHT	12.1	12.5	0	0
29	7055	0	Azithromycin	0	1	4/1/2015	63	LENGTH	6.6	13.5	0	0
29	7055	24	Azithromycin	0	28	4/28/2017	85.9	HEIGHT	9.9	13	0	0
29	7056	24	Azithromycin	1	28	4/29/2017	76.9	HEIGHT	8.909090909	13.5	1	0
29	7056	36	Azithromycin	1	41	5/19/2018	86.5	HEIGHT	12.35	16.5	1	0
29	7056	48	Azithromycin	1	52	4/19/2019	90.7	HEIGHT	14.05	15.5	1	0
29	7058	0	Azithromycin	0	24	5/9/2015	88	HEIGHT	11.25	14	1	0
29	7058	12	Azithromycin	0	39	6/28/2016	97.4	HEIGHT	13.45	14.5	0	0
29	7058	24	Azithromycin	0	52	4/28/2017	102.3	HEIGHT	14.77272727	14.5	0	0
29	7058	36	Azithromycin	0	65	5/19/2018	108.5	HEIGHT	16.2	14	0	0
29	7058	48	Azithromycin	0	76	4/19/2019	111.1	HEIGHT	17.05	13.5	0	0
29	7059	24	Azithromycin	0	30	4/28/2017	82.2	HEIGHT	11.72727273	14	1	0
29	7061	0	Azithromycin	1	48	4/1/2015	100.7	HEIGHT	15.5	15	1	0
29	7061	12	Azithromycin	1	63	6/25/2016	108.5	HEIGHT	16.6	14.5	0	0
29	7061	24	Azithromycin	1	76	4/28/2017	115.3	HEIGHT	19.09090909	14.5	0	0
29	7061	36	Azithromycin	1	89	5/19/2018	122.6	HEIGHT	20.95	15	0	0
29	7062	0	Azithromycin	1	48	4/1/2015	100.8	HEIGHT	14.15	14.5	0	0
29	7062	12	Azithromycin	1	66	6/28/2016	108.5	HEIGHT	15.4	15	0	0
29	7062	24	Azithromycin	1	76	4/28/2017	113.4	HEIGHT	17.40909091	14.5	0	0
29	7062	36	Azithromycin	1	89	5/19/2018	118.4	HEIGHT	18.8	15	0	0
29	7062	48	Azithromycin	1	100	4/19/2019	122.9	HEIGHT	20.6	15.5	0	0
29	7062	60	Azithromycin	1	109	2/14/2020	127.3	HEIGHT	21.35	15	0	0
29	7063	12	Azithromycin	0	7	6/27/2016	72.5	HEIGHT	8.15	14.5	0	0
29	7063	24	Azithromycin	0	15	4/28/2017	81	HEIGHT	10	13.5	0	0
29	7066	0	Azithromycin	1	48	5/9/2015	124.3	HEIGHT	25.15	16	0	0
29	7066	24	Azithromycin	1	76	4/28/2017	133.1	HEIGHT	28.75	17	0	0
29	7066	36	Azithromycin	1	89	5/19/2018	137.5	HEIGHT	30.2	18	0	0
29	7070	0	Azithromycin	1	48	6/12/2015	101.1	HEIGHT	15.2	15.5	1	0
29	7070	12	Azithromycin	1	66	6/25/2016	110.5	HEIGHT	16.95	15	0	0
29	7074	24	Azithromycin	0	34	4/28/2017	82.5	HEIGHT	12.22727273	16	1	0
29	7074	48	Azithromycin	0	58	4/19/2019	95.3	HEIGHT	15.5	15.5	1	0
29	7075	24	Azithromycin	0	2	4/29/2017	59.4	LENGTH	4.954545455	12	1	0

29	7075	60	Azithromycin	0	35	3/10/2020	88.4	HEIGHT	10.25	13		1	0
29	7079	0	Azithromycin	0	36	4/1/2015	79.6	HEIGHT	8.7	12.5	24	1	0
29	7079	12	Azithromycin	0	51	6/25/2016	88.5	HEIGHT	11.2	14	24	0	0
29	7081	0	Azithromycin	1	1	5/9/2015	62.9	LENGTH	5.75	12	42	0	0
29	7081	24	Azithromycin	1	28	4/28/2017	85.2	HEIGHT	10.85	14	42	0	0
29	7081	36	Azithromycin	1	41	5/19/2018	94	HEIGHT	12.75	14	42	0	0
29	7082	0	Azithromycin	0	48	5/9/2015	102.2	HEIGHT	15.1	14.5		1	0
29	7082	12	Azithromycin	0	54	6/25/2016	108.2	HEIGHT	16.8	14		0	0
29	7082	24	Azithromycin	0	67	4/28/2017	114.1	HEIGHT	17.65	13.5		0	0
29	7082	36	Azithromycin	0	80	5/19/2018	118.4	HEIGHT	19.8	13.5		0	0
29	7082	48	Azithromycin	0	91	4/19/2019	122.5	HEIGHT	21.95	14		0	0
29	7082	60	Azithromycin	0	100	2/14/2020	126.1	HEIGHT	23.1	14.5		0	0
29	7083	0	Azithromycin	1	48	4/1/2015	100.6	HEIGHT	14.7	14.5		0	0
29	7083	12	Azithromycin	1	66	6/27/2016	108.1	HEIGHT	17.4	11.5		0	0
29	7083	24	Azithromycin	1	76	4/28/2017	113.8	HEIGHT	18.86363636	15		0	0
29	7087	0	Azithromycin	1	24	5/9/2015	85.5	HEIGHT	10.25	14		1	0
29	7087	12	Azithromycin	1	39	6/27/2016	93.4	HEIGHT	12.15	15		0	0
29	7087	24	Azithromycin	1	52	4/28/2017	98.6	HEIGHT	13.65	14.5		1	0
29	7087	36	Azithromycin	1	65	5/19/2018	105	HEIGHT	14.8	14.5		0	0
29	7087	48	Azithromycin	1	76	4/19/2019	108.2	HEIGHT	15.65	14		0	0
29	7087	60	Azithromycin	1	86	2/14/2020	114.9	HEIGHT	17.8	13.5		0	0
29	7089	0	Azithromycin	1	36	4/1/2015	83.6	HEIGHT	10.35	13		1	0
29	7089	12	Azithromycin	1	51	6/24/2016	89.2	HEIGHT	12.55	14.5		1	0
29	7089	24	Azithromycin	1	64	4/28/2017	94.3	HEIGHT	13.4	14		0	0
29	7089	36	Azithromycin	1	65	5/19/2018	99.8	HEIGHT	15.7	14.5		0	0
29	7089	48	Azithromycin	1	76	4/19/2019	104.6	HEIGHT	16.25	14.2		0	0
29	7090	12	Azithromycin	1	39	6/25/2016	95.2	HEIGHT	12.45	13		1	0
29	7091	0	Azithromycin	0	24	6/12/2015	83	HEIGHT	10.95	15.5		0	0
29	7091	12	Azithromycin	0	45	6/25/2016	91.5	HEIGHT	12.05	15		0	0
29	7091	24	Azithromycin	0	55	4/29/2017	98.9	HEIGHT	14.36363636	15		0	0
29	7091	36	Azithromycin	0	68	5/19/2018	106.2	HEIGHT	16.2	15		0	0
29	7091	48	Azithromycin	0	79	4/19/2019	112.1	HEIGHT	18.1	14.5		0	0
29	7094	24	Azithromycin	1	12	4/28/2017	72.8	LENGTH	8.045454545	13.5		1	0
29	7094	36	Azithromycin	1	24	5/19/2018	81.8	HEIGHT	10.4	15		0	0
29	7094	48	Azithromycin	1	35	4/19/2019	89.1	HEIGHT	12.2	14.5		0	0
29	7095	0	Azithromycin	1	24	6/12/2015	86.2	HEIGHT	11.65	14.5		1	0
29	7095	12	Azithromycin	1	48	6/25/2016	94.2	HEIGHT	14.1	16		0	0
29	7095	24	Azithromycin	1	61	4/28/2017	102.5	HEIGHT	16	14.5		0	0
29	7095	36	Azithromycin	1	74	5/19/2018	109.4	HEIGHT	17.75	15.5		0	0
29	7095	48	Azithromycin	1	85	4/19/2019	112.2	HEIGHT	17.25	14.5		0	0

29	7095	60	Azithromycin	1	94	2/14/2020	115.7	HEIGHT	19.02	14.5		0	0
29	7096	0	Azithromycin	1	12	5/9/2015	72.4	LENGTH	7.85	13		0	0
29	7096	12	Azithromycin	1	27	6/25/2016	84	HEIGHT	11.75	15		0	0
29	7096	24	Azithromycin	1	40	4/28/2017	88.2	HEIGHT	13.4	14.5		0	0
29	7096	36	Azithromycin	1	53	5/19/2018	99.4	HEIGHT	14.95	15		1	0
29	7096	48	Azithromycin	1	64	4/19/2019	104.9	LENGTH	15.6	15.5		0	0
29	7096	60	Azithromycin	1	73	2/14/2020	111.5	HEIGHT	17.65	14.5		0	0
29	7100	12	Azithromycin	1	3	6/25/2016	59.7	HEIGHT	5.75	12.5		0	0
29	7100	24	Azithromycin	1	12	4/29/2017	72.9	LENGTH	7.363636364	13		0	0
29	7100	36	Azithromycin	1	24	5/19/2018	82.5	HEIGHT	8.65	12.5		1	0
29	7100	60	Azithromycin	1	45	2/14/2020	74.1	HEIGHT	11.8	13.9		0	0
29	7101	24	Azithromycin	0	4	4/28/2017	60.8	LENGTH	5.45	12		1	0
29	7101	36	Azithromycin	0	15	5/19/2018	72.8	LENGTH	7.55	12		0	0
29	7101	48	Azithromycin	0	26	4/19/2019	76.5	LENGTH	8.05	12		0	0
29	7103	0	Azithromycin	1	7	4/1/2015	67.3	LENGTH	7.15	14	30	0	0
29	7103	12	Azithromycin	1	21	6/25/2016	80.7	HEIGHT	10.65	15.5	30	0	0
29	7103	24	Azithromycin	1	34	4/28/2017	87	HEIGHT	13.05	17.5	30	0	0
29	7106	0	Azithromycin	1	48	4/1/2015	98.2	HEIGHT	13.45	15		0	0
29	7107	0	Azithromycin	1	24	6/12/2015	74.8	LENGTH	8.45	13.5	24	0	0
29	7107	12	Azithromycin	0	39	6/24/2016	83.4	HEIGHT	11.4	14.5	24	1	0
29	7109	24	Azithromycin	0	52	4/29/2017	94.8	HEIGHT	13.81818182	15		1	0
29	7110	0	Azithromycin	0	36	5/9/2015	83.4	HEIGHT	10.25	13.5		1	0
29	7110	12	Azithromycin	0	51	6/25/2016	90	HEIGHT	12.6	15		1	0
29	7110	36	Azithromycin	0	77	5/19/2018	101.7	HEIGHT	13.75	14		0	0
29	7110	60	Azithromycin	0	97	2/14/2020	110.8	HEIGHT	16.95	13.7		0	0
29	7111	0	Azithromycin	0	12	4/1/2015	72.8	LENGTH	8.4	14		1	0
29	7111	12	Azithromycin	0	27	6/28/2016	81.2	HEIGHT	10.35	14.5		0	0
29	7111	24	Azithromycin	0	40	4/28/2017	87.7	HEIGHT	11.95	15		0	0
29	7111	36	Azithromycin	0	53	5/19/2018	93.1	HEIGHT	13.05	15		1	0
29	7111	48	Azithromycin	0	64	4/19/2019	99	HEIGHT	14	15		0	0
29	7112	24	Azithromycin	0	15	4/29/2017	74.8	LENGTH	5.454545455	8.5	30	1	0
29	7113	0	Azithromycin	0	12	6/12/2015	73.3	LENGTH	8.15	13		1	0
29	7113	12	Azithromycin	0	27	6/25/2016	83.5	HEIGHT	10.1	14.5		0	0
29	7113	24	Azithromycin	0	40	4/28/2017	91.2	HEIGHT	12.55	14		0	0
29	7113	36	Azithromycin	0	53	5/19/2018	98.5	HEIGHT	13.7	14		1	0
29	7113	48	Azithromycin	0	64	4/19/2019	103.4	HEIGHT	15.1	13.5		0	0
29	7113	60	Azithromycin	0	73	2/14/2020	109.4	HEIGHT	16.6	14		0	0
29	7117	12	Azithromycin	0	42	6/25/2016	100.4	HEIGHT	15.15	15		1	0
29	7119	0	Azithromycin	0	36	6/12/2015	84	LENGTH	11.15	15		1	0
29	7119	12	Azithromycin	1	30	6/28/2016	92.5	LENGTH	12.75	15.5		0	0

29	7119	24	Azithromycin	1	43	4/29/2017	99	HEIGHT	14	15		1	0
29	7119	36	Azithromycin	1	56	5/19/2018	106.7	HEIGHT	16.5	15		1	0
29	7119	48	Azithromycin	1	67	4/19/2019	111.3	HEIGHT	17.15	15		0	0
29	7119	60	Azithromycin	1	76	2/14/2020	118.2	HEIGHT	19.15	14.6		0	0
29	7121	0	Azithromycin	1	48	5/9/2015	93.8	HEIGHT	12.95	15		1	0
29	7121	12	Azithromycin	1	54	6/24/2016	102.8	HEIGHT	14.6	15		1	0
29	7121	24	Azithromycin	1	67	4/28/2017	108.3	HEIGHT	15.18181818	14.5		0	0
29	7121	48	Azithromycin	1	91	4/19/2019	118	HEIGHT	20.4	16		0	0
29	7121	60	Azithromycin	1	100	2/14/2020	122.7	HEIGHT	21.6	16.5		0	0
29	7125	0	Azithromycin	0	36	5/9/2015	89.5	HEIGHT	10.95	13.5		1	0
29	7125	12	Azithromycin	0	54	6/28/2016	98.1	HEIGHT	11.9	13.5		0	0
29	7125	24	Azithromycin	0	67	4/29/2017	103.5	HEIGHT	13.63636364	13.5		0	0
29	7125	36	Azithromycin	0	80	5/19/2018	109	HEIGHT	13.9	13		0	0
29	7125	60	Azithromycin	0	100	2/14/2020	116.5	HEIGHT	17.5	14		0	0
29	7126	12	Azithromycin	1	15	6/25/2016	70.4	HEIGHT	7.45	14		1	0
29	7126	24	Azithromycin	1	28	4/29/2017	80.3	LENGTH	8.954545455	14.5		1	0
29	7126	36	Azithromycin	1	41	5/19/2018	87.8	HEIGHT	10.85	14.5		1	0
29	7132	0	Azithromycin	1	36	4/1/2015	95.6	HEIGHT	10.25	12.5		1	0
29	7133	0	Azithromycin	1	24	6/12/2015	84.7	HEIGHT	10.4	13.5	54	1	0
29	7133	12	Azithromycin	1	30	6/25/2016	94	HEIGHT	12.75	14	54	0	0
29	7133	24	Azithromycin	1	43	4/28/2017	100	HEIGHT	14.04545455	14	54	0	0
29	7133	36	Azithromycin	1	56	5/19/2018	108.4	HEIGHT	15.75	14	54	0	0
29	7134	0	Azithromycin	0	48	4/1/2015	97.6	HEIGHT	13.1	13		0	0
29	7134	12	Azithromycin	0	63	6/25/2016	106.9	HEIGHT	15	14		0	0
29	7134	24	Azithromycin	0	76	4/28/2017	111.2	HEIGHT	16.6	14		0	0
29	7136	12	Azithromycin	1	12	6/25/2016	67.1	LENGTH	5.85	12.5	30	1	0
29	7136	24	Azithromycin	1	22	4/29/2017	69.5	LENGTH	6.318181818	11.5	30	1	0
29	7137	0	Azithromycin	0	12	5/9/2015	70.8	LENGTH	7.75	13		0	0
29	7137	12	Azithromycin	0	27	6/25/2016	80.9	HEIGHT	10.55	14.5		1	0
29	7137	24	Azithromycin	0	40	4/29/2017	86.7	HEIGHT	13.04545455	15		1	0
29	7137	36	Azithromycin	0	53	5/19/2018	96.8	HEIGHT	15.65	15.5		1	0
29	7143	24	Azithromycin	0	43	4/28/2017	84.8	HEIGHT	10.95454545	14		1	0
29	7146	0	Azithromycin	1	36	4/1/2015	92.4	HEIGHT	12.5	15		0	0
29	7146	24	Azithromycin	1	64	4/28/2017	104.5	HEIGHT	15.6	15		0	0
29	7146	36	Azithromycin	1	77	5/19/2018	109.8	HEIGHT	15.95	14.5		0	0
29	7146	48	Azithromycin	1	88	4/19/2019	113.3	HEIGHT	17.4	14.5		0	0
29	7147	0	Azithromycin	1	36	4/1/2015	88.5	HEIGHT	12.65	15		1	0
29	7147	12	Azithromycin	1	51	6/24/2016	97.3	HEIGHT	13.85	15		1	0
29	7147	24	Azithromycin	1	64	4/28/2017	100.7	HEIGHT	14.6	15		0	0
29	7147	36	Azithromycin	1	77	5/19/2018	105.4	HEIGHT	15.3	14		0	0

29	7147	48	Azithromycin	1	88	4/19/2019	109.2	HEIGHT	17.15	14.5		0	0
29	7147	60	Azithromycin	1	97	2/14/2020	114.3	HEIGHT	18.85	14.9		0	0
29	7148	0	Azithromycin	0	36	5/9/2015	96.7	HEIGHT	13.5	14		1	0
29	7148	12	Azithromycin	0	51	6/28/2016	103.2	HEIGHT	14.95	14.6		0	0
29	7148	24	Azithromycin	0	64	4/28/2017	106	HEIGHT	16.95	15		0	0
29	7149	0	Azithromycin	1	24	5/9/2015	80.6	HEIGHT	9.35	13	30	1	0
29	7149	12	Azithromycin	1	39	6/27/2016	88.5	HEIGHT	10.7	15	30	1	0
29	7149	24	Azithromycin	1	52	4/28/2017	95.9	HEIGHT	12.25	13.5	30	1	0
29	7150	24	Azithromycin	1	12	4/28/2017	72.8	LENGTH	7.727272727	13		1	0
29	7150	36	Azithromycin	1	24	5/19/2018	80.4	HEIGHT	10.7	14		0	0
29	7152	12	Azithromycin	1	42	6/24/2016	89.5	HEIGHT	9.2	13.5		1	0
29	7153	0	Azithromycin	0	36	6/12/2015	90.1	HEIGHT	12.45	15.5		1	0
29	7153	12	Azithromycin	0	51	6/24/2016	96.6	HEIGHT	14.5	14		1	0
29	7153	24	Azithromycin	0	57	4/29/2017	101.6	HEIGHT	15.77272727	14		0	0
29	7153	36	Azithromycin	0	70	5/19/2018	106.2	HEIGHT	16.65	13.5		0	0
29	7153	48	Azithromycin	0	81	4/19/2019	110.7	HEIGHT	18.9	14		0	0
29	7153	60	Azithromycin	0	91	2/14/2020	116.2	HEIGHT	21.1	14.5		0	0
29	7155	24	Azithromycin	0	4	4/28/2017	63.6	LENGTH	5.818181818	13		1	0
29	7155	48	Azithromycin	0	28	4/19/2019	82	HEIGHT	9.55	13.5		1	0
29	7157	12	Azithromycin	0	6	6/27/2016	63.8	LENGTH	5.7	11.5	18	1	0
29	7159	12	Azithromycin	1	45	6/25/2016	96.9	HEIGHT	12.5	14		1	0
29	7159	24	Azithromycin	1	58	4/28/2017	101.9	HEIGHT	14.4	13.5		1	0
29	7161	12	Azithromycin	1	8	6/25/2016	67	LENGTH	6.35	12		0	0
29	7161	24	Azithromycin	1	15	4/28/2017	77.4	HEIGHT	8.045454545	12		1	0
29	7161	36	Azithromycin	1	28	5/19/2018	85.5	HEIGHT	9.4	12.5		1	0
29	7161	48	Azithromycin	1	39	4/19/2019	92.6	HEIGHT	11.35	13		1	0
29	7161	60	Azithromycin	1	49	2/14/2020	82.9	HEIGHT	12.1	15.2		1	0
29	7162	12	Azithromycin	1	6	6/28/2016	68	LENGTH	7.05	14.5	24	0	0
29	7163	0	Azithromycin	0	36	4/1/2015	86.5	HEIGHT	11.35	14.5		0	0
29	7165	12	Azithromycin	0	8	6/25/2016	71.3	HEIGHT	8.75	14.5		0	0
29	7165	24	Azithromycin	0	15	4/28/2017	79.5	HEIGHT	10.3	14.5		1	0
29	7166	24	Azithromycin	0	5	4/28/2017	64.8	LENGTH	5.863636364	12.5		1	0
29	7166	36	Azithromycin	0	15	5/19/2018	77.9	LENGTH	8.95	13.5		1	0
29	7166	60	Azithromycin	0	35	2/14/2020	91	LENGTH	11.85	13.5		0	0
29	7167	12	Azithromycin	1	7	6/27/2016	62.4	LENGTH	5.55	11.5		1	0
29	7167	24	Azithromycin	1	15	4/29/2017	73.5	LENGTH	8.045454545	12		1	0
29	7167	36	Azithromycin	1	28	5/19/2018	82.5	HEIGHT	10.6	14.5		1	0
29	7167	60	Azithromycin	1	49	3/10/2020	92.5	HEIGHT	12.6	13		1	0
29	7168	0	Azithromycin	0	1	4/1/2015	65.8	LENGTH	6.15	11.5		1	0
29	7168	12	Azithromycin	0	15	6/25/2016	77.9	HEIGHT	8.05	12		1	0

29	7168	24	Azithromycin	0	28	4/28/2017	86.3	HEIGHT	11.36363636	13.5		0	0
29	7168	36	Azithromycin	0	41	5/19/2018	97.4	HEIGHT	13.15	13		1	0
29	7172	12	Azithromycin	0	11	6/24/2016	68.5	LENGTH	6.6	11.5		1	0
29	7172	24	Azithromycin	0	15	4/28/2017	75.8	HEIGHT	7.454545455	11.5		1	0
29	7172	48	Azithromycin	0	39	4/19/2019	93.2	HEIGHT	12.55	12.5		1	0
29	7172	60	Azithromycin	0	49	2/14/2020	101	HEIGHT	13.95	12.8		0	0
29	7174	24	Azithromycin	1	49	4/29/2017	105.3	HEIGHT	13.54545455	13		1	0
29	7175	0	Azithromycin	0	36	4/1/2015	94.5	HEIGHT	12.8	13.5		1	0
29	7175	12	Azithromycin	0	51	6/25/2016	102.1	HEIGHT	14.55	14		1	0
29	7175	24	Azithromycin	0	64	4/28/2017	108.1	HEIGHT	15.09090909	13		0	0
29	7175	36	Azithromycin	0	77	5/19/2018	111.9	HEIGHT	16.05	13		0	0
29	7175	48	Azithromycin	0	88	4/19/2019	115.7	HEIGHT	18.4	13.5		0	0
29	7179	24	Azithromycin	0	12	4/29/2017	74.5	LENGTH	9.909090909	15.5		1	0
29	7179	36	Azithromycin	0	24	5/19/2018	84.5	HEIGHT	11.15	15		1	0
29	7179	48	Azithromycin	0	35	4/19/2019	88.4	HEIGHT	12.55	14.5		1	0
29	7180	0	Azithromycin	0	48	6/12/2015	102.1	HEIGHT	12.8	14		1	0
29	7181	12	Azithromycin	0	51	6/24/2016	92	HEIGHT	12.85	15		1	0
29	7183	12	Azithromycin	1	57	6/25/2016	101.5	HEIGHT	15.65	14.5		0	0
29	7183	24	Azithromycin	1	67	4/28/2017	105.4	HEIGHT	17.04545455	14		0	0
29	7183	36	Azithromycin	1	80	5/19/2018	113.2	HEIGHT	19.3	15		0	0
29	7183	48	Azithromycin	1	91	4/19/2019	116.8	HEIGHT	20.45	14.5		0	0
29	7187	0	Azithromycin	1	48	5/9/2015	104.5	HEIGHT	14.45	13.5		0	0
29	7187	24	Azithromycin	1	76	4/29/2017	116.5	HEIGHT	17.90909091	14		0	0
29	7187	36	Azithromycin	1	89	5/19/2018	120.5	HEIGHT	19.45	14.5		0	0
29	7188	12	Azithromycin	1	2	6/24/2016	60.8	LENGTH	5.4	12		1	0
29	7190	12	Azithromycin	1	45	6/24/2016	96.8	HEIGHT	13.2	14		1	0
29	7190	24	Azithromycin	1	55	4/29/2017	101.4	HEIGHT	14.22727273	13.5		1	0
29	7191	12	Azithromycin	0	10	6/25/2016	72.6	LENGTH	8.1	13.5		0	0
29	7191	24	Azithromycin	0	21	4/28/2017	80	HEIGHT	9.181818182	14		0	0
29	7191	36	Azithromycin	0	30	5/19/2018	88.4	HEIGHT	11.55	13.5		0	0
29	7191	60	Azithromycin	0	50	2/14/2020	101.8	HEIGHT	14.6	14.2		0	0
29	7193	0	Azithromycin	0	12	4/1/2015	72.8	LENGTH	7.3	12	6	0	0
29	7195	0	Azithromycin	0	48	6/12/2015	91	HEIGHT	12.55	15	12	1	0
29	8003	36	Azithromycin	0	5	5/19/2018	66.7	LENGTH	6.55	14	42	1	0
29	8023	60	Azithromycin	0	12	2/21/2020	74	LENGTH	6.82	13		1	0
29	8026	60	Azithromycin	1	44	3/10/2020	98.9	HEIGHT	15.15	14.8		1	0
29	8032	36	Azithromycin	0	12	5/19/2018	74.5	LENGTH	8.4	13.5		1	0
29	8061	48	Azithromycin	0	22	4/22/2019	100	HEIGHT	13.8	14.5		1	0
29	8062	48	Azithromycin	0	12	4/19/2019	94.6	LENGTH	8.85	15.5		1	0
29	8062	60	Azithromycin	0	21	2/21/2020	83.9	LENGTH	10.7	14.2		1	0

29	8100	36	Azithromycin	1	44	5/19/2018	84.3	HEIGHT	10.3	13	1	0
29	8109	36	Azithromycin	0	47	5/19/2018	94.2	HEIGHT	12.45	13.5	1	0
29	8119	48	Azithromycin	1	10	4/19/2019	70.4	LENGTH	7.75	13	1	0
29	8119	60	Azithromycin	1	20	2/14/2020	82.7	LENGTH	9.95	15.9	1	0
29	8130	48	Azithromycin	0	32	4/19/2019	91.2	HEIGHT	13	14	1	0
29	8130	60	Azithromycin	0	41	2/14/2020	98.6	HEIGHT	14.25	14	1	0
29	8161	36	Azithromycin	0	47	5/19/2018	84.9	HEIGHT	9.95	11.5	1	0
29	8186	36	Azithromycin	0	18	5/19/2018	75.9	LENGTH	8.65	14	1	0
29	8186	48	Azithromycin	0	28	4/19/2019	83.2	HEIGHT	10.15	14.5	1	0
29	8216	48	Azithromycin	0	15	4/19/2019	79.8	LENGTH	10.45	14	1	0
29	8216	60	Azithromycin	0	25	2/14/2020	87.5	HEIGHT	13.35	15.5	1	0
29	8271	48	Azithromycin	1	35	4/22/2019	85.9	HEIGHT	10.95	13.5	1	0
29	8274	60	Azithromycin	1	30	2/21/2020	83.6	LENGTH	10.25	14	1	0
29	8321	60	Azithromycin	1	55	2/21/2020	110.8	HEIGHT	19.3	16.5	1	0
29	8326	60	Azithromycin	1	32	3/10/2020	83.4	HEIGHT	10.05	13.2	1	0
29	8364	48	Azithromycin	0	52	4/19/2019	97.3	HEIGHT	14.2	14	1	0
29	8406	48	Azithromycin	0	14	4/19/2019	77.3	LENGTH	9.85	14.5	1	0
29	8409	48	Azithromycin	1	15	4/22/2019	76.4	LENGTH	9.85	14	1	0
29	8496	48	Azithromycin	0	48	4/19/2019	92.6	HEIGHT	10.75	12	1	0
29	8509	48	Azithromycin	1	26	4/19/2019	80.4	HEIGHT	10.2	14	1	0
29	8536	48	Azithromycin	1	51	4/19/2019	96.1	HEIGHT	13.2	13.5	1	0
29	8548	48	Azithromycin	1	15	4/19/2019	75.2	LENGTH	8.7	14.5	1	0
29	8548	60	Azithromycin	1	25	3/10/2020	82.2	HEIGHT	11.85	14	1	0
29	8556	36	Azithromycin	0	12	5/19/2018	76.2	LENGTH	8.5	13	1	0
29	8556	48	Azithromycin	0	22	4/19/2019	82.8	HEIGHT	10.85	13.5	0	0
29	8604	36	Azithromycin	0	5	5/19/2018	55	LENGTH	3.1	9	1	0
29	8604	48	Azithromycin	0	15	4/19/2019	65.8	LENGTH	5.75	10.5	0	0
29	8622	48	Azithromycin	0	45	4/19/2019	96	HEIGHT	13.2	13.5	1	0
29	8657	48	Azithromycin	1	17	4/19/2019	72.2	LENGTH	8.2	12.5	1	0
29	8687	60	Azithromycin	0	9	2/21/2020	74.8	LENGTH	10.05	14.4	1	0
29	8741	60	Azithromycin	1	37	3/10/2020	87.7	HEIGHT	11.7	13.8	1	0
29	8804	60	Azithromycin	1	36	2/21/2020	91.7	LENGTH	12.3	14.5	1	0
29	8841	48	Azithromycin	1	12	4/19/2019	76.9	HEIGHT	9	13.5	1	0
29	8841	60	Azithromycin	1	21	2/14/2020	84.5	HEIGHT	11	14	1	0
29	8849	36	Azithromycin	0	7	5/19/2018	65.7	LENGTH	7	13	1	0
29	8849	60	Azithromycin	0	27	3/10/2020	75.7	LENGTH	8.6	11	1	0
29	8861	60	Azithromycin	0	26	2/14/2020	75.6	LENGTH	9.2	13.5	1	0
29	8915	60	Azithromycin	0	13	2/14/2020	75.6	LENGTH	8.05	13	1	0
29	8927	60	Azithromycin	1	21	2/21/2020	78.6	LENGTH	8.65	13	1	0
29	8950	48	Azithromycin	0	15	4/19/2019	73.5	LENGTH	8.25	14	1	0

29	8952	48	Azithromycin	0	12	4/19/2019	67.8	LENGTH	8.4	14.5		1	0
29	8952	60	Azithromycin	0	21	2/21/2020	78.3	LENGTH	10.25	15		1	0
29	8958	60	Azithromycin	0	21	2/21/2020	74.9	LENGTH	8.5	13.5		1	0
29	8970	48	Azithromycin	0	36	4/19/2019	89	HEIGHT	11.65	14.5		1	0
29	8987	36	Azithromycin	0	12	5/19/2018	74.2	LENGTH	8.6	13		1	0
29	8987	48	Azithromycin	0	22	4/19/2019	82.6	LENGTH	10.15	13.5		1	0
29	8987	60	Azithromycin	0	32	2/14/2020	90.3	HEIGHT	13.4	14.2		0	0
29	8998	36	Azithromycin	0	41	5/19/2018	83.7	HEIGHT	12.1	14	54	1	0
29	9042	36	Azithromycin	0	12	5/19/2018	68.5	LENGTH	5.95	11		1	0
29	9042	48	Azithromycin	0	23	4/19/2019	77.2	HEIGHT	7.4	12		0	0
29	9042	60	Azithromycin	0	32	2/21/2020	83.3	HEIGHT	10.2	13.8		0	0
29	9056	36	Azithromycin	0	82	5/19/2018	111.1	LENGTH	16.45	15.5		0	0
29	9056	48	Azithromycin	0	91	4/19/2019	116	LENGTH	17.8	15.5		0	0
29	9056	60	Azithromycin	0	100	2/14/2020	122.8	LENGTH	18.15	15.4		0	0
29	9065	36	Azithromycin	0	12	5/19/2018	82.1	LENGTH	11.6	16		1	0
29	9065	48	Azithromycin	0	22	4/19/2019	91.4	LENGTH	13.85	16		1	0
29	9065	60	Azithromycin	0	32	2/14/2020	99.2	HEIGHT	16.15	16.1		0	0
29	9077	36	Azithromycin	0	3	5/19/2018	62.6	LENGTH	5.95	12.5		1	0
29	9077	48	Azithromycin	0	14	4/19/2019	75.8	HEIGHT	8.25	14		0	0
29	9089	60	Azithromycin	1	25	2/21/2020	80.2	HEIGHT	9.65	13.8		1	0
29	9117	60	Azithromycin	0	24	2/21/2020	88.9	HEIGHT	12	13.9		1	0
29	9146	60	Azithromycin	0	8	3/10/2020	69.1	LENGTH	7.95	13.7		1	0
29	9148	36	Azithromycin	1	12	5/19/2018	71	LENGTH	7.4	12.5		1	0
29	9148	60	Azithromycin	1	32	3/10/2020	83.8	HEIGHT	9.55	11.8		1	0
29	9196	36	Azithromycin	0	28	5/19/2018	76.1	LENGTH	7.8	13		1	0
29	9196	48	Azithromycin	0	39	4/22/2019	81.7	HEIGHT	8.8	12		1	0
29	9212	60	Azithromycin	0	24	2/21/2020	84.8	LENGTH	11	13.5		1	0
29	9329	48	Azithromycin	1	46	4/19/2019	96.5	HEIGHT	14.2	15		1	0
29	9341	48	Azithromycin	0	48	4/19/2019	104.1	HEIGHT	14.2	13		0	0
29	9354	48	Azithromycin	0	48	4/19/2019	101.7	HEIGHT	15.25	15		1	0
29	9360	36	Azithromycin	0	12	5/19/2018	79.2	LENGTH	9.7	15		1	0
29	9360	48	Azithromycin	0	22	4/19/2019	87.3	LENGTH	11.9	14		0	0
29	9360	60	Azithromycin	0	32	2/14/2020	93.6	LENGTH	14.05	15.3		1	0
29	9370	36	Azithromycin	0	56	5/19/2018	109.3	HEIGHT	16.75	14.5		1	0
29	9375	60	Azithromycin	0	26	2/21/2020	83	HEIGHT	9.9	12		1	0
29	9403	48	Azithromycin	0	48	4/19/2019	103.7	HEIGHT	14.55	13.5		1	0
29	9446	48	Azithromycin	1	24	4/19/2019	79.3	LENGTH	8.05	12		1	0
29	9455	48	Azithromycin	1	10	4/19/2019	69.4	HEIGHT	6.75	11.5		1	0
29	9455	60	Azithromycin	1	14	2/14/2020	81	LENGTH	9.4	13		0	0
29	9469	48	Azithromycin	1	14	4/19/2019	75.2	HEIGHT	7.95	13		1	0

29	9525	60	Azithromycin	1	12	2/21/2020	72.9	LENGTH	7.7	12.5	1	0
29	9541	60	Azithromycin	1	21	2/14/2020	79.8	LENGTH	9.35	13	1	0
29	9546	48	Azithromycin	1	12	4/19/2019	74.7	LENGTH	10.25	16	1	0
29	9546	60	Azithromycin	1	21	2/14/2020	99.3	HEIGHT	13.55	14	1	0
29	9549	48	Azithromycin	0	22	4/19/2019	83.3	HEIGHT	10.5	15	1	0
29	9562	36	Azithromycin	1	13	5/19/2018	71.9	LENGTH	7.7	13.5	1	0
29	9562	48	Azithromycin	1	24	4/19/2019	81	LENGTH	9.3	13.5	0	0
29	9562	60	Azithromycin	1	33	2/14/2020	88.3	LENGTH	11.1	13.5	1	0
29	9577	36	Azithromycin	1	53	5/19/2018	93.9	HEIGHT	11.7	13.5	1	0
30	7222	0	Azithromycin	1	54	3/15/2015	94.3	HEIGHT	13.75	16	1	0
30	7222	12	Azithromycin	1	78	6/27/2016	103.5	HEIGHT	15.8	15.5	0	0
30	7224	0	Azithromycin	1	36	6/16/2015	91.2	HEIGHT	11.7	13.5	1	0
30	7224	12	Azithromycin	1	42	6/27/2016	97.2	HEIGHT	13.15	13.5	0	0
30	7225	12	Azithromycin	0	12	6/22/2016	75.1	HEIGHT	10.75	14	1	0
30	7226	0	Azithromycin	1	24	3/15/2015	68.6	HEIGHT	7.1	12.5	1	0
30	7227	0	Azithromycin	0	48	3/15/2015	97.7	HEIGHT	12.4	13.5	1	0
30	7227	12	Azithromycin	0	57	6/27/2016	106.7	HEIGHT	13.95	13	0	0
30	7227	24	Azithromycin	0	72	5/23/2017	112.9	HEIGHT	14.75	12.5	0	0
30	7232	0	Azithromycin	1	52	6/16/2015	104.5	HEIGHT	15.55	14	1	0
30	7233	12	Azithromycin	0	11	6/27/2016	71	LENGTH	7.75	13.5	0	0
30	7234	0	Azithromycin	1	54	6/16/2015	107.7	HEIGHT	14.6	13	1	0
30	7234	24	Azithromycin	1	75	5/23/2017	117.6	HEIGHT	18.40909091	13.5	0	0
30	7235	0	Azithromycin	0	36	3/15/2015	95.3	HEIGHT	13	14.5	1	0
30	7235	12	Azithromycin	0	54	6/22/2016	105.2	HEIGHT	14	13.5	1	0
30	7235	24	Azithromycin	0	68	5/23/2017	112.1	HEIGHT	15.65	14	0	0
30	7236	24	Azithromycin	1	17	5/23/2017	74.5	LENGTH	8.6	13.5	0	0
30	7239	24	Azithromycin	0	50	5/23/2017	102.5	HEIGHT	14.95	16	0	0
30	7241	0	Azithromycin	0	48	3/15/2015	84.5	HEIGHT	10.85	14.5	1	0
30	7241	12	Azithromycin	0	57	6/22/2016	92.8	HEIGHT	12.9	15	1	0
30	7241	24	Azithromycin	0	72	5/23/2017	99.3	HEIGHT	13.95	14.5	0	0
30	7242	0	Azithromycin	1	48	3/15/2015	98.7	HEIGHT	13.2	14	1	0
30	7245	0	Azithromycin	0	54	6/16/2015	103.2	HEIGHT	14.7	14.5	1	0
30	7245	12	Azithromycin	0	66	6/27/2016	109.3	HEIGHT	16.25	14.5	0	0
30	7246	24	Azithromycin	1	22	5/24/2017	78.5	HEIGHT	10.2	14.5	1	0
30	7248	0	Azithromycin	0	24	3/15/2015	95.7	HEIGHT	13.7	15	1	0
30	7248	12	Azithromycin	0	39	6/28/2016	103.1	HEIGHT	14.9	15	0	0
30	7248	24	Azithromycin	0	54	5/23/2017	107.5	HEIGHT	15.86363636	14.5	0	0
30	7249	24	Azithromycin	1	5	5/23/2017	57.1	LENGTH	3.590909091	8.5	1	0
30	7250	12	Azithromycin	0	48	6/22/2016	94.6	HEIGHT	10.9	11.5	1	0
30	7255	0	Azithromycin	0	9	6/16/2015	72.7	LENGTH	8.65	14.5	1	0

30	7257	0	Azithromycin	0	5	3/15/2015	63.8	LENGTH	5.8	12.5	1	0
30	7257	12	Azithromycin	0	18	6/27/2016	75.3	HEIGHT	7.35	12.5	0	0
30	7258	12	Azithromycin	1	18	6/22/2016	73.5	HEIGHT	9.15	15.5	1	0
30	7258	24	Azithromycin	1	30	5/23/2017	82.3	HEIGHT	10.7	14.5	1	0
30	7259	24	Azithromycin	0	11	5/23/2017	74.2	HEIGHT	9.95	15.5	1	0
30	7260	0	Azithromycin	0	36	3/15/2015	84.5	HEIGHT	10.75	14	0	0
30	7260	12	Azithromycin	0	42	6/22/2016	96.3	HEIGHT	13.5	14	1	0
30	7260	24	Azithromycin	0	56	5/23/2017	103.2	HEIGHT	14.90909091	14	1	0
30	7261	24	Azithromycin	0	26	5/24/2017	90	HEIGHT	11.7	14.5	1	0
30	7264	0	Azithromycin	1	24	3/15/2015	81	HEIGHT	9	13.5	1	0
30	7264	12	Azithromycin	1	31	6/22/2016	90.1	HEIGHT	10.6	14	1	0
30	7264	24	Azithromycin	1	45	5/23/2017	95.2	HEIGHT	11.77272727	13.5	1	0
30	7265	24	Azithromycin	1	12	5/23/2017	72.3	LENGTH	7.590909091	12.5	1	0
30	7267	0	Azithromycin	1	36	3/15/2015	85.5	HEIGHT	10.85	14.5	1	0
30	7267	12	Azithromycin	1	42	6/27/2016	95.4	HEIGHT	13	14	0	0
30	7267	24	Azithromycin	1	56	5/23/2017	102.3	HEIGHT	14.63636364	13.5	1	0
30	7268	0	Azithromycin	0	36	3/15/2015	89.9	HEIGHT	13.2	15	0	0
30	7269	0	Azithromycin	0	18	6/16/2015	64	LENGTH	6.65	13.5	1	0
30	7269	12	Azithromycin	0	30	6/22/2016	88.4	HEIGHT	10.45	15.5	1	0
30	7271	0	Azithromycin	1	54	3/15/2015	116.2	HEIGHT	19.35	15	1	0
30	7272	0	Azithromycin	0	12	6/16/2015	75.3	HEIGHT	9.35	14	1	0
30	7274	0	Azithromycin	0	54	3/15/2015	114.3	HEIGHT	15.85	13	1	0
30	7276	0	Azithromycin	0	54	3/15/2015	121.4	HEIGHT	19.85	15.5	1	0
30	7276	12	Azithromycin	0	66	6/27/2016	125.4	HEIGHT	22	16	0	0
30	7276	24	Azithromycin	0	80	5/23/2017	132.5	HEIGHT	24.18181818	16.5	0	0
30	7277	0	Azithromycin	0	36	3/15/2015	89.6	HEIGHT	11.55	15.5	0	0
30	7278	12	Azithromycin	0	57	6/22/2016	112	HEIGHT	15.95	14.5	1	0
30	7279	0	Azithromycin	0	54	3/15/2015	124.3	HEIGHT	21	15	1	0
30	7279	12	Azithromycin	0	111	6/28/2016	129	HEIGHT	21.9	16	0	0
30	7280	0	Azithromycin	0	54	3/15/2015	124.2	HEIGHT	23.2	16	1	0
30	7284	0	Azithromycin	1	12	3/15/2015	69.7	LENGTH	7.55	13.5	1	0
30	7284	12	Azithromycin	1	30	6/22/2016	84	HEIGHT	9.55	14	1	0
30	7284	24	Azithromycin	1	44	5/23/2017	90.9	HEIGHT	11.27272727	13.5	0	0
30	7285	0	Azithromycin	1	36	6/16/2015	101.6	HEIGHT	16.05	15.5	1	0
30	7286	24	Azithromycin	0	26	5/23/2017	87.8	HEIGHT	11.86363636	14.5	1	0
30	7289	0	Azithromycin	1	54	3/15/2015	93.6	HEIGHT	13.15	14.5	0	0
30	7289	12	Azithromycin	1	66	6/27/2016	103.2	HEIGHT	14.8	14.5	0	0
30	7289	24	Azithromycin	1	77	5/23/2017	109.1	HEIGHT	16.09090909	15	0	0
30	7290	12	Azithromycin	0	4	6/22/2016	67.3	LENGTH	7.05	13.5	1	0
30	7292	12	Azithromycin	0	54	6/22/2016	91.5	HEIGHT	11.45	14	1	0

30	7294	0	Azithromycin	0	48	3/15/2015	110	HEIGHT	17.15	15.5	0	0
30	7294	12	Azithromycin	0	72	6/27/2016	116.7	HEIGHT	19.25	15.5	0	0
30	7295	0	Azithromycin	1	36	3/15/2015	82.1	HEIGHT	10	13	1	0
30	7295	12	Azithromycin	1	48	6/27/2016	92.4	HEIGHT	11.65	14	0	0
30	7295	24	Azithromycin	1	63	5/23/2017	97.7	HEIGHT	12.90909091	13.5	0	0
30	7296	0	Azithromycin	0	5	3/15/2015	58.3	LENGTH	5.4	13	1	0
30	7296	12	Azithromycin	0	12	6/28/2016	72.6	LENGTH	7.9	13	1	0
30	7296	24	Azithromycin	0	26	5/23/2017	79.3	HEIGHT	10.27272727	14.5	0	0
30	7297	0	Azithromycin	0	36	3/15/2015	81.7	HEIGHT	10.1	14.5	1	0
30	7297	12	Azithromycin	0	42	6/27/2016	93.5	HEIGHT	12.65	14	0	0
30	7297	24	Azithromycin	0	56	5/23/2017	100.6	HEIGHT	14.36363636	14	1	0
30	7298	24	Azithromycin	1	32	5/23/2017	91.3	HEIGHT	13	14.5	1	0
30	7301	12	Azithromycin	1	51	6/22/2016	91.8	HEIGHT	12.15	15	1	0
30	7302	12	Azithromycin	1	15	6/28/2016	74.7	HEIGHT	7.9	14	1	0
30	7303	0	Azithromycin	0	18	3/15/2015	77.5	LENGTH	8.05	12.5	1	0
30	7303	24	Azithromycin	0	42	5/23/2017	94.8	HEIGHT	12.25	13.5	1	0
30	7305	12	Azithromycin	0	5	6/22/2016	63.3	LENGTH	6	12	1	0
30	7305	24	Azithromycin	0	17	5/23/2017	73.5	HEIGHT	7.227272727	12	1	0
30	7306	0	Azithromycin	0	24	3/15/2015	80.1	HEIGHT	9.5	12.5	1	0
30	7306	12	Azithromycin	0	36	6/27/2016	88.5	HEIGHT	12.4	14.5	0	0
30	7306	24	Azithromycin	0	51	5/23/2017	95.9	HEIGHT	14.15	14	0	0
30	7308	0	Azithromycin	0	30	6/16/2015	82.3	HEIGHT	9.55	14	1	0
30	7309	0	Azithromycin	0	54	3/15/2015	105	HEIGHT	14.35	14	1	0
30	7309	12	Azithromycin	1	78	6/27/2016	111.9	HEIGHT	15.65	14.5	0	0
30	7311	24	Azithromycin	0	44	5/23/2017	99.2	HEIGHT	13.40909091	13.5	1	0
30	7313	0	Azithromycin	0	48	3/15/2015	105	HEIGHT	16.6	15	1	0
30	7313	24	Azithromycin	0	74	5/23/2017	117.7	HEIGHT	19.25	15.5	0	0
30	7314	0	Azithromycin	0	48	3/15/2015	117.2	HEIGHT	19.1	15.5	0	0
30	7314	12	Azithromycin	1	66	6/28/2016	123.8	HEIGHT	20.85	16.5	0	0
30	7314	24	Azithromycin	1	80	5/23/2017	129	HEIGHT	23.27272727	17	0	0
30	7316	12	Azithromycin	0	49	6/22/2016	98	HEIGHT	14.5	15.5	1	0
30	7321	12	Azithromycin	0	54	6/22/2016	104.5	HEIGHT	16.1	15.5	1	0
30	7322	0	Azithromycin	0	36	3/15/2015	102.1	HEIGHT	17.2	16	1	0
30	7322	12	Azithromycin	0	43	6/28/2016	109.9	HEIGHT	19.3	15.5	1	0
30	7323	0	Azithromycin	1	12	3/15/2015	71.2	LENGTH	7.4	13	1	0
30	7323	24	Azithromycin	1	44	5/23/2017	89.7	HEIGHT	10.45	14	0	0
30	7325	0	Azithromycin	1	54	3/15/2015	93.3	HEIGHT	12.4	15	0	0
30	7325	12	Azithromycin	1	63	7/11/2016	102.8	HEIGHT	14.75	15	0	0
30	7327	0	Azithromycin	1	48	3/15/2015	99.3	HEIGHT	13.9	14	0	0
30	7328	12	Azithromycin	0	2	6/22/2016	67.5	LENGTH	7.2	13.5	1	0

30	7329	0	Azithromycin	1	34	6/16/2015	83.1	HEIGHT	11.8	16	1	0
30	7332	0	Azithromycin	1	54	3/15/2015	106.4	HEIGHT	16.3	16	0	0
30	7334	12	Azithromycin	0	45	6/22/2016	100.1	HEIGHT	13.6	14.5	1	0
30	7334	24	Azithromycin	0	56	5/23/2017	105.7	HEIGHT	14.90909091	14.5	1	0
30	7335	0	Azithromycin	0	48	3/15/2015	96.4	HEIGHT	13.55	14.5	1	0
30	7335	24	Azithromycin	0	68	5/23/2017	111.1	HEIGHT	15.5	13.5	0	0
30	7337	0	Azithromycin	0	48	3/15/2015	96.4	HEIGHT	11.75	13.5	0	0
30	7337	12	Azithromycin	0	66	6/27/2016	104.4	HEIGHT	12.75	13	0	0
30	7337	24	Azithromycin	0	80	5/23/2017	109.1	HEIGHT	14.04545455	14	0	0
30	7338	0	Azithromycin	1	6	3/15/2015	63.6	LENGTH	5.95	14	1	0
30	7338	12	Azithromycin	1	18	6/27/2016	74.7	HEIGHT	8	13	0	0
30	7338	24	Azithromycin	1	30	5/23/2017	82.5	HEIGHT	9.636363636	13	1	0
30	7339	0	Azithromycin	1	24	6/16/2015	86.3	HEIGHT	13	16	1	0
30	7339	24	Azithromycin	1	56	5/23/2017	100	HEIGHT	15.2	15.5	0	0
30	7340	12	Azithromycin	0	30	6/22/2016	81.8	HEIGHT	11	14	1	0
30	7340	24	Azithromycin	0	44	5/23/2017	89.4	HEIGHT	13.3	15	1	0
30	7343	0	Azithromycin	1	48	6/16/2015	109.9	HEIGHT	17.1	15	1	0
30	7344	0	Azithromycin	1	54	3/15/2015	98.4	HEIGHT	13.65	14.5	0	0
30	7344	24	Azithromycin	1	78	5/23/2017	110.6	HEIGHT	16.95454545	15.5	0	0
30	7345	0	Azithromycin	0	7	3/15/2015	69.5	LENGTH	7.6	14	1	0
30	7345	12	Azithromycin	0	15	6/27/2016	82.4	HEIGHT	10.1	13.5	0	0
30	7347	0	Azithromycin	1	5	3/15/2015	61	LENGTH	6.7	13.5	0	0
30	7347	12	Azithromycin	1	15	6/22/2016	72.8	HEIGHT	7.55	12	1	0
30	7347	24	Azithromycin	1	30	5/23/2017	78.6	HEIGHT	9.55	13	1	0
30	7348	0	Azithromycin	0	24	3/15/2015	74.1	HEIGHT	7.2	12	0	0
30	7349	0	Azithromycin	0	36	3/15/2015	96.9	HEIGHT	14.5	15	1	0
30	7349	12	Azithromycin	0	45	6/22/2016	108	HEIGHT	16.85	15.5	1	0
30	7353	12	Azithromycin	0	57	6/22/2016	109	HEIGHT	17.75	16	1	0
30	7355	0	Azithromycin	0	54	6/16/2015	105	HEIGHT	14.55	13.5	1	0
30	7358	12	Azithromycin	0	42	6/22/2016	97.1	HEIGHT	12.8	13	1	0
30	7358	24	Azithromycin	0	56	5/23/2017	104	HEIGHT	14.85	13.5	1	0
30	7359	0	Azithromycin	1	24	3/15/2015	80.6	HEIGHT	11.95	16.5	1	0
30	7359	12	Azithromycin	1	54	6/22/2016	91.3	HEIGHT	14.65	16	1	0
30	7359	24	Azithromycin	1	48	5/23/2017	99.2	HEIGHT	15.90909091	16	1	0
30	7360	0	Azithromycin	1	5	3/15/2015	61.8	LENGTH	5.65	12	0	0
30	7361	0	Azithromycin	1	48	3/15/2015	92.1	HEIGHT	12.85	14.5	0	0
30	7361	12	Azithromycin	1	57	6/22/2016	100.6	HEIGHT	15.15	14.5	1	0
30	7361	24	Azithromycin	1	72	5/23/2017	106	HEIGHT	16.86363636	14.5	0	0
30	7364	0	Azithromycin	0	10	6/16/2015	82.1	LENGTH	8.95	13	1	0
30	7365	0	Azithromycin	1	48	6/16/2015	98.3	HEIGHT	12.55	14	1	0

30	7367	24	Azithromycin	0	26	5/23/2017	81.7	HEIGHT	10.22727273	13.5		1	0
30	7371	0	Azithromycin	0	30	6/16/2015	88.5	HEIGHT	13.05	15		1	0
30	7371	24	Azithromycin	0	53	5/23/2017	102	HEIGHT	15.63636364	14		1	0
30	7372	0	Azithromycin	1	24	6/16/2015	87	HEIGHT	11.5	14		1	0
30	7372	12	Azithromycin	1	33	6/22/2016	93.6	HEIGHT	12.45	14.5		1	0
30	7372	24	Azithromycin	1	48	5/23/2017	102.1	HEIGHT	14.4	14		0	0
30	7374	0	Azithromycin	0	54	6/16/2015	122.4	HEIGHT	22.25	15		1	0
30	7375	24	Azithromycin	1	26	5/23/2017	83	LENGTH	10.2	13		1	0
30	7378	0	Azithromycin	0	42	3/15/2015	116	HEIGHT	20.35	15.5		0	0
30	7378	12	Azithromycin	0	54	6/28/2016	121.2	HEIGHT	22.5	16.5		0	0
30	7378	24	Azithromycin	0	68	5/23/2017	126	HEIGHT	24.1	16		0	0
30	7379	0	Azithromycin	1	48	6/16/2015	101.3	HEIGHT	14.25	15.5		1	0
30	7379	24	Azithromycin	1	56	5/23/2017	110.1	HEIGHT	17.5	15.5		1	0
30	7380	0	Azithromycin	0	54	6/16/2015	100.2	HEIGHT	12.6	14.5	24	1	0
30	7381	0	Azithromycin	0	12	3/15/2015	69.9	LENGTH	6.7	12	12	1	0
30	7382	12	Azithromycin	0	31	6/28/2016	93.6	HEIGHT	12.25	13.5		1	0
30	7382	24	Azithromycin	0	45	5/23/2017	95.7	HEIGHT	13.1	13		1	0
30	7383	0	Azithromycin	1	36	6/16/2015	88.6	HEIGHT	11.5	13.5		1	0
30	7383	12	Azithromycin	1	45	6/22/2016	96.5	HEIGHT	12.85	14		1	0
30	7383	24	Azithromycin	1	71	5/23/2017	105.9	HEIGHT	15.05	14		0	0
30	7384	0	Azithromycin	1	12	3/15/2015	65	LENGTH	5.5	11.5		1	0
30	7384	12	Azithromycin	1	30	6/22/2016	78.5	HEIGHT	8.95	13.5		1	0
30	7384	24	Azithromycin	1	44	5/23/2017	84.2	HEIGHT	10.18181818	13.5		1	0
30	7387	24	Azithromycin	1	6	5/23/2017	58	LENGTH	5.1	11.5		1	0
30	7388	24	Azithromycin	0	32	5/23/2017	93.3	HEIGHT	12.31818182	14		1	0
30	7390	0	Azithromycin	0	54	3/15/2015	111.4	HEIGHT	18.25	15		1	0
30	7390	12	Azithromycin	0	63	6/28/2016	118.3	HEIGHT	20	16		0	0
30	7391	0	Azithromycin	1	48	3/15/2015	99.7	HEIGHT	14.45	15.5		0	0
30	7391	12	Azithromycin	1	57	6/27/2016	107.7	HEIGHT	15.4	13		0	0
30	7391	24	Azithromycin	1	72	5/23/2017	113.3	HEIGHT	16.85	15.5		0	0
30	7392	12	Azithromycin	1	12	6/22/2016	71.2	HEIGHT	7.1	13		1	0
30	7392	24	Azithromycin	1	26	5/23/2017	79.6	LENGTH	8.909090909	13		0	0
30	7394	24	Azithromycin	1	32	5/23/2017	95.5	HEIGHT	13.77272727	15		1	0
30	7396	24	Azithromycin	1	10	5/23/2017	76	LENGTH	9.681818182	15.5		1	0
30	7399	0	Azithromycin	0	12	3/15/2015	73.5	LENGTH	7.95	12.5		1	0
30	7399	12	Azithromycin	0	24	6/27/2016	86.3	HEIGHT	10.3	12.5		0	0
30	7399	24	Azithromycin	0	38	5/23/2017	94.2	HEIGHT	12.3	13		0	0
30	7401	24	Azithromycin	0	26	5/23/2017	85.6	HEIGHT	11.59090909	14.5		1	0
30	7402	0	Azithromycin	1	54	3/15/2015	116.5	HEIGHT	24.3	19		1	0
30	7402	12	Azithromycin	1	93	6/27/2016	123.5	HEIGHT	26.7	20		0	0

30	7403	24	Azithromycin	0	17	5/23/2017	79	HEIGHT	9.363636364	13.5		0	0
30	7407	0	Azithromycin	0	24	3/15/2015	82.8	HEIGHT	9.55	13.5		1	0
30	7408	0	Azithromycin	1	12	3/15/2015	73.1	LENGTH	7.75	12.5		0	0
30	7409	0	Azithromycin	1	8	6/16/2015	76	LENGTH	8.5	13.5	30	1	0
30	7409	12	Azithromycin	1	24	6/22/2016	87.1	HEIGHT	11	14	30	1	0
30	7411	0	Azithromycin	1	34	6/16/2015	98.2	HEIGHT	14.65	15.5		1	0
30	7411	24	Azithromycin	1	61	5/23/2017	108.6	HEIGHT	17.5	16		0	0
30	7412	12	Azithromycin	1	10	6/27/2016	68.7	LENGTH	6.95	12		0	0
30	7412	24	Azithromycin	1	23	5/23/2017	78.8	HEIGHT	8.3	12		1	0
30	7413	12	Azithromycin	0	9	6/28/2016	78.2	LENGTH	8.75	13		0	0
30	7413	24	Azithromycin	0	17	5/23/2017	89.3	HEIGHT	11.25	12.5		0	0
30	7414	24	Azithromycin	1	17	5/23/2017	73	HEIGHT	7.3	12.5		1	0
30	7415	12	Azithromycin	0	4	6/22/2016	72.9	LENGTH	8.05	13.5		1	0
30	7415	24	Azithromycin	0	17	5/23/2017	78.8	HEIGHT	8.55	12.5		1	0
30	7417	0	Azithromycin	1	42	3/15/2015	114.8	HEIGHT	18.8	15.5		1	0
30	7417	12	Azithromycin	1	69	6/27/2016	123.2	HEIGHT	21.2	16.5		0	0
30	7417	24	Azithromycin	1	80	5/23/2017	127.7	HEIGHT	22.85	17		0	0
30	7419	0	Azithromycin	0	46	6/16/2015	101.8	HEIGHT	14.6	14.5		1	0
30	7420	12	Azithromycin	1	54	6/22/2016	103.9	HEIGHT	15.85	15.5		1	0
30	7421	0	Azithromycin	1	48	3/15/2015	85.4	HEIGHT	11.3	14		0	0
30	7422	0	Azithromycin	1	24	3/15/2015	79.3	HEIGHT	9.5	13		1	0
30	7425	0	Azithromycin	0	12	3/15/2015	69.5	LENGTH	7.1	13		0	0
30	7427	24	Azithromycin	0	44	5/23/2017	97.7	HEIGHT	13.22727273	13.5		1	0
30	7428	24	Azithromycin	1	22	5/23/2017	77.6	HEIGHT	10.31818182	14.5		1	0
30	7429	0	Azithromycin	1	36	3/15/2015	83	HEIGHT	10.8	14.5		1	0
30	7429	12	Azithromycin	1	42	6/27/2016	93.9	HEIGHT	13.2	15.5		0	0
30	7429	24	Azithromycin	1	56	5/23/2017	100.2	HEIGHT	14.31818182	15		1	0
30	7430	0	Azithromycin	1	48	3/15/2015	92.5	HEIGHT	13.7	15		1	0
30	7430	12	Azithromycin	1	57	6/27/2016	102.9	HEIGHT	15.2	15		0	0
30	7433	24	Azithromycin	0	15	5/23/2017	60.9	LENGTH	6.2	13		1	0
30	7437	0	Azithromycin	0	18	6/16/2015	76	HEIGHT	8.45	12.5		1	0
30	7437	24	Azithromycin	0	42	5/23/2017	91	HEIGHT	11.81818182	13.5		1	0
